# A Convenient Synthesis of Novel Isoxazolidine and Isoxazole Isoquinolinones Fused Hybrids

**DOI:** 10.3390/molecules29010091

**Published:** 2023-12-22

**Authors:** Konstantinos A. Ouzounthanasis, Stergios R. Rizos, Alexandros E. Koumbis

**Affiliations:** 1 Laboratory of Organic Chemistry, Department of Chemistry, Aristotle University of Thessaloniki, 54124 Thessaloniki, Greece; 2 Department of Chemistry and Chemical Biology, Harvard University, 12 Oxford St., Cambridge, MA 02138, USA; stergiosrizos@fas.harvard.edu

**Keywords:** isoxazole, isoxazolidine, isoquinolinone, indanone, nitrone, nitrile oxide, 1,3-dipolar cycloaddition, Schmidt reaction, Ullmann reaction

## Abstract

Isoxazolidine, isoxazole, and isoquinolinone rings are present in the structure of several natural products and/or pharmaceutically interesting compounds. In this work, facile and efficient pathways have been developed for the preparation of fused frameworks bearing those heterocycles. The successful approaches for both isoxazolidine/isoquinolinone and isoxazole/isoquinolinone hybrid syntheses relied initially on 1,3-dipolar cycloadditions of nitrones and nitrile oxides to indenone and 2-propargylbenzamide, respectively. The construction of the isoquinolinone lactam system followed by performing a selective Schmidt reaction for isoxazolidine derivatives (two steps overall), whereas the isoxazole lactams were reached via an Ullmann-type cyclisation (three steps overall). Key observations were made regarding the stereo- and regioselectivities of the reactions employed, and small libraries of the targeted hybrids were prepared, demonstrating the general applicability of these strategies.

## 1. Introduction

**Figure 1:**
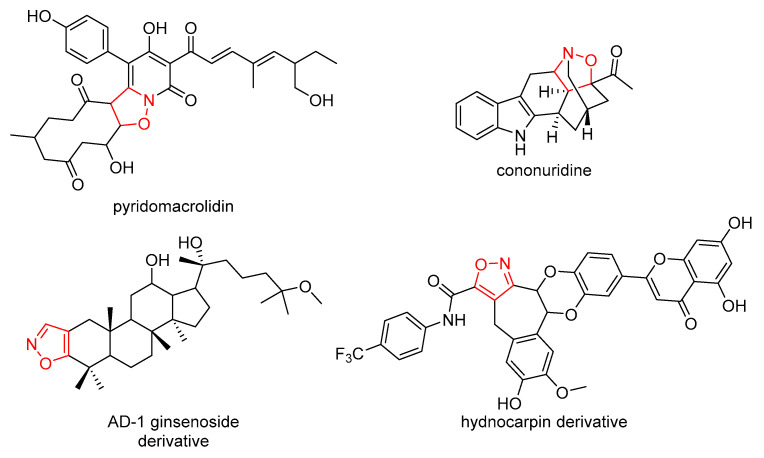
Representative examples of natural products and bioactive derivatives bearing the isoxazolidine or isoxazole moiety [7,8,9,10].

The 1,3-dipolar cycloaddition reaction (Huisgen cycloaddition) is established as a powerful tool for constructing five-membered heterocycles [1]. Among the various heterocyclic rings that are easily accessible via this strategy, isoxazolidines and isoxazoles, the cycloadducts obtained when nitrones or nitrile oxides are employed as 1,3-dipoles in reactions with alkenes and alkynes, respectively, are of great importance [2,3]. These scaffolds are present in the structure of several natural products, and in many instances, they were proved to be crucial for the observed biological activity [4] (Figure 1). A plethora of drugs and other compounds of interest to the pharmaceutical industry also contain those five-membered heterocycles [5,6], thus increasing the synthetic value of the 1,3-dipolar cycloaddition approach not only for the ease of preparing heterocyclic compounds but also for inserting high structural complexity in a straightforward manner.

**Figure 2:**
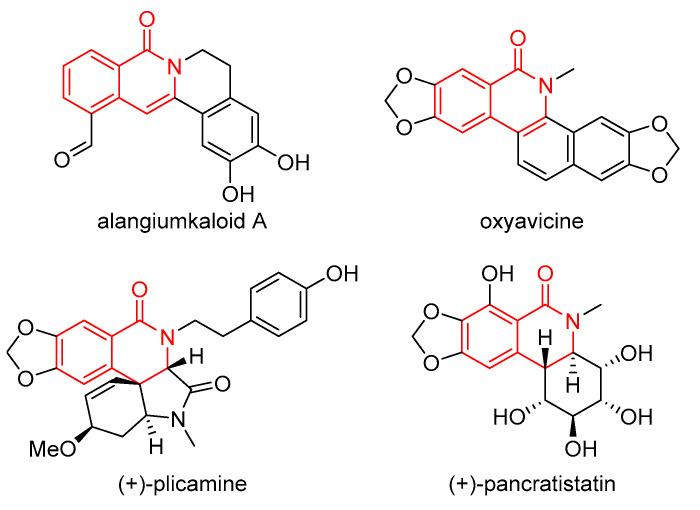
Isoquinolinone or tetrahydroisoquinolinone scaffold in some natural products [15,16,17,18].

Another structural feature commonly appearing in biological active compounds, including either natural products or synthetic drugs, is the isoquinolinone or tetrahydroisoquinolinone moiety [11] (Figure 2). Such ring systems are often found in several pharmacophores, and positive results of Structure–Activity Relationship (SAR) studies support the design of new compounds bearing the aforementioned scaffold [12]. A convenient approach for the construction of the benzolactamic backbone present in those ring systems is the Schmidt reaction [13,14]. Apparently, the harsh acidic/thermal conditions required for the Schmidt protocol could be considered a drawback for the range of desired potential substrates. However, proper modifications may lead to a larger substrate pool being tolerated.

The facile preparation of libraries of novel hybrid compounds combining the two interesting structural features (isoxazolidine or isoxazole heterocycle and (tetra)isoquinolinone ring system) seems to be an appealing and challenging goal. A versatile synthetic methodology could provide potent lead compounds against various biological targets (enzyme inhibition, antifungal activity, etc.). Therefore, development of a concise synthetic sequence that simultaneously maintains a high level of versatility was the task we decided to address. Moreover, observations of the chemical behaviour and reactivity of both the heterocyclic intermediates and the targeted compounds could extract valuable information for the development of future synthetic routes towards other related compounds, so as to avoid unforeseen complications and the formation of unwanted side products in newly designed syntheses.

**Figure 3:**
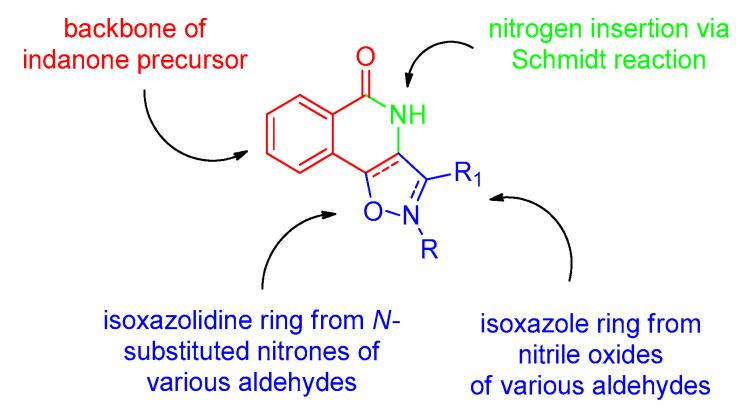
Design of targeted compounds.

**Scheme 1:**
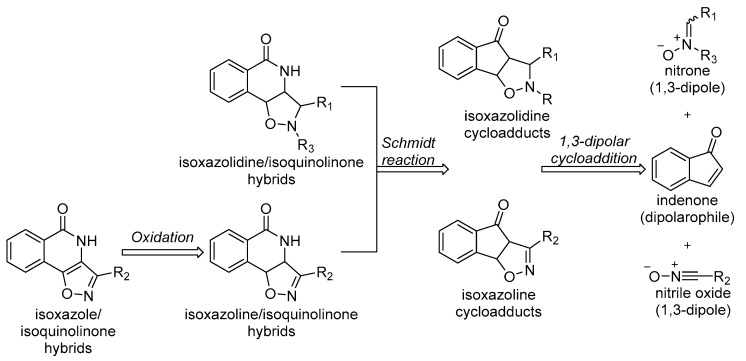
Retrosynthetic plan for the targeted hybrids.

According to our original design, the employment of two key reactions was envisioned for the construction of the main core of the targeted compounds (Figure 3). The introduction of the amide functional group was planned via a Schmidt reaction to a ketone isoxazolidine or isoxazoline cycloadduct. The latter can be prepared utilising an 1,3-dipolar cycloaddition reaction between indenone, serving as the dipolarophile partner, and a nitrone or nitrile oxide 1,3-dipole. In the case of isoxazoline/isoquinolinones, a final oxidation step is required to obtain the target isoxazole derivatives. Both 1,3-dipoles and the dipolarophile can be accessed through known synthetic methodologies from commercially available reagents (Scheme 1).

## 2. Results and Discussion

### 2.1. Synthesis of the Starting Materials

**Scheme 2:**
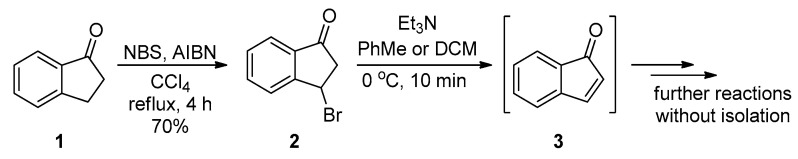
Preparation of dipolarophile **3**.

Due to its instability, indenone (**3**) was prepared from 3-bromoindanone (**2**) upon treatment with Et_3_N, each time prior to its use [19]. Bromide **2** was prepared from 1-indanone (**1**) at a large scale (up to 10 g) following a known procedure [20] and was kept in the freezer for months without any sign of decomposition (Scheme 2).

**Scheme 3:**
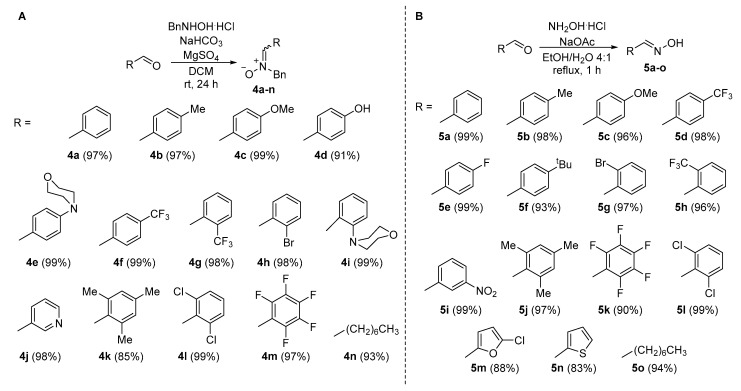
(**A**). Preparation of nitrones **4**. (**B**). Preparation of nitrile oxide precursors **5**.

For the first batch of 1,3-dipoles, we chose to proceed with *N*-benzyl nitrones of various commercially available aldehydes, consisting mostly of benzaldehydes. A modified procedure of a typical one reported in the literature [21] was followed, and, in total, 14 nitrones [22,23,24,25,26,27] were synthesised (Scheme 3A). In a similar manner, a variety of aldehydes were transformed to the corresponding oximes (Scheme 3B), following a known general protocol [28]. These oximes [29,30,31,32,33,34,35,36,37] served as the precursors of the required nitrile oxides.

### 2.2. Synthesis of Isoxazolidine/Isoquinolinone Hybrids

#### 2.2.1. Optimisation Studies for the 1,3-dipolar Cycloaddition Reaction between Indenone and Nitrones

**Scheme 4:**
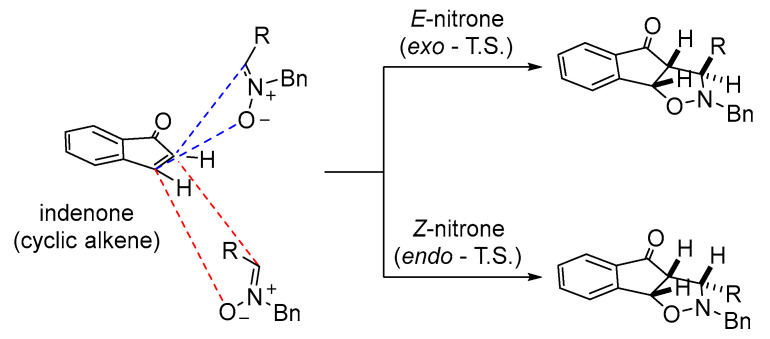
The two possible stereoisomeric adducts for the reaction of indenone **3** and a nitrone.

In principle, four cycloadducts are to be expected as plausible products from the 1,3-dipolar cycloaddition reaction between a nitrone and a cyclic alkene. The *E*/*Z*-nitrone isomerisation in combination with the competing effects of secondary interactions, such as pi stacking and steric hindrance from bulky groups, can play a crucial role, thus rendering it rather hard to predict the stereoselective outcome (Scheme 4). Additionally, the issue of regioselectivity (regioisomers not shown) may complicate the situation even more. However, we presumed that in our case only one regio-orientation would be favoured due to the α,β-unsaturated system present in indenone.

**Figure 4:**
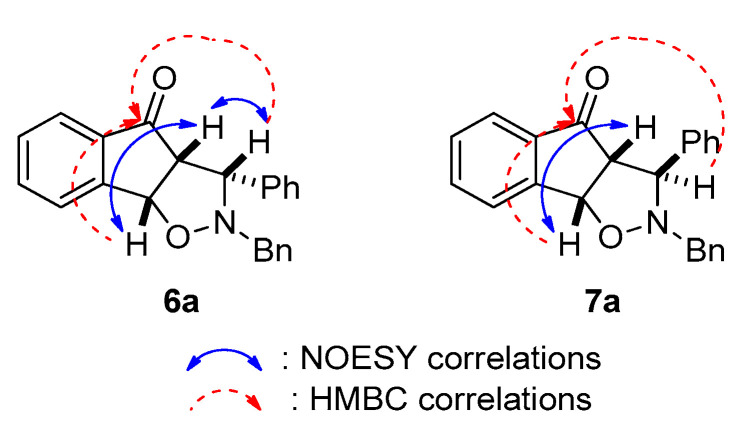
Determining the structure of cycloadducts **6a** and **7a** using 2D NMR spectra.

To identify the preference towards the two competing stereoisomeric cycloadducts, several conditions were examined for the model reaction between *N*-benzyl nitrone of benzaldehyde (**4a**) and the in situ prepared indenone (**3**) (Table 1). Initially, simply refluxing the two partners in toluene afforded only two cycloadducts, **6a** and **7a**, with a good total yield (70%) (entry 1), whereas at lower temperatures the reaction seemed to be sluggish, regardless of the solvent used (only representative entries 1–3 are given in Table 1). The two adducts were successfully separated with column chromatography. 2D NMR spectroscopy (Figure 4) revealed that both cycloadducts emerged from the same regio orientation of the nitrone and were stereoisomers in regard to the relative geometry of the nitrone (*E*/*Z* isomerisation). Next, we settled with conducting a thorough screening in an attempt to determine conditions that may lead to the selective preparation of each of the two cycloadducts and further improve the yield. Metal triflates are widely used in such reactions with nitrones to control stereo- and regioselectivity because a lot of those compounds are commercially available [38,39,40]. Therefore, besides modifying the solvent, temperature, and reaction time, we also checked the influence of a reasonable number of metal triflates in substoichiometrical amounts as additives in order to investigate whether one of the cycloadducts is favoured over the other. Although we have not managed to establish a completely selective protocol, it is worth mentioning that Zn(OTf)_2_ (entries 7 and 8) led to the best ratio (almost 2:1 in favour of *endo*-adduct **6a**) and an overall yield of 80%. The best results in terms of the overall yield of the reaction (90%) were obtained using AgOTf as the additive, but without a significant effect on selectivity regarding the formation of the two cycloadducts (**6a**/**7a** 1.25:1, entry 12). The silver salt counteranion proved to be of little importance because similar results for the stereoselectivity and the yield were observed with all silver salts employed (entries 12, 18, and 19). In an attempt to reverse the regioselectivity by taking advantage of their high affinity towards oxygen, titanium, and tin, Lewis acids were used (entries 16 and 17), but with no success. In general, heating the reaction mixture at 80 °C gave optimum results both in terms of productivity and time of completion.

#### 2.2.2. Synthesis of Diverse Isoxazolidines

We then applied our optimised conditions (entry 12, Table 1) by using as dipoles the *N*-benzyl nitrones **4** shown in Scheme 3A (Table 2). For almost every nitrone examined, the *endo* cycloadduct **6** was the predominant one, in accordance with the optimisation studies. However, in two cases (entries 13 and 14), the *exo* cycloadduct was the main product of the reaction. It should be noted that for the *o*-substituted phenyl nitrones **4g** and **4h** (entries 7 and 8), the formation of the corresponding regioisomers **8** was also verified. 2D NMR studies were again used to prove that the latter originate from an *exo*-T.S. pathway (in a way similar to the analysis shown in Figure 4).

#### 2.2.3. Optimisation of the Schmidt Reaction of Isoxazolidine Adducts

The Schmidt reaction represents a convenient method for the conversion of cyclic ketones to lactams. The nitrogen migration depends strongly on the acidic medium that the reaction is carried in (H_2_SO_4_, PPA, HCl) [13], while the respective substrate holds a decisive role as well (electronic effects of existing substituents may alter the outcome) [41,42,43].

**Figure 5:**
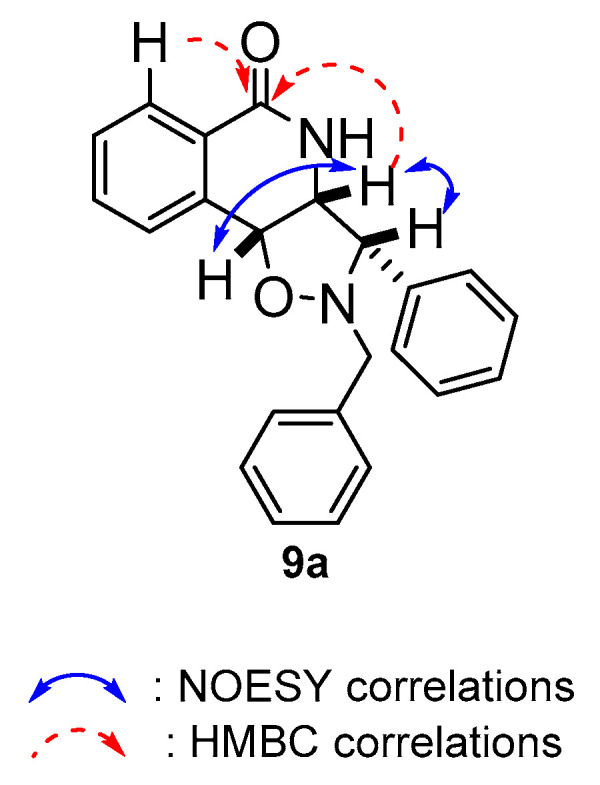
Determining the structure of lactam **9a** using 2D NMR spectra.

To investigate the optimal conditions for the transformation of our ketone cycloadducts (**6** and **7**) to lactams (tetrahydroisoquinolinones), a number of experiments were conducted using *endo* cycloadduct **6a** as the model substrate (Table 3). Interestingly enough, many of the more commonly used acids (HCl, H_2_SO_4_) failed to give any reaction, or the yield was meagre. Trifluoroacetic acid (TFA) and the extremely powerful triflic acid (TfOH) also failed to afford any product, and the starting material was recovered intact in both cases. The first encouraging result was obtained using methanesulfonic acid (MsOH), as shown in entry 9. Ultimately, proper modifications led to a protocol that furnished the desired tetrahydroisoquinolinone **9a**, with a very good yield (entry 11). 2D NMR studies (Figure 5) confirmed the nitrogen migration to the *sp*^3^ (alkyl) carbon and retention of the relative stereochemistry of protons of the starting adduct **6a** in hybrid **9a**. Additionally, the formation of the tetrazole by-product **10a** was observed. In order to test whether amide **9a** could be formed exclusively, the Schmidt reaction was also carried out at a lower temperature (0 °C) in DCM or CHCl_3_, but the starting isoxazolidine **6a** remained intact (experiments not shown in Table 3). 

**Scheme 5:**
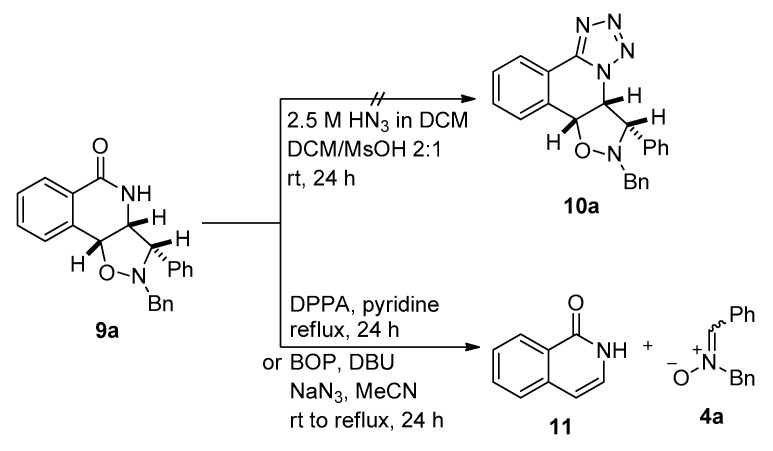
Attempting tetrazole formation from lactam **9a**.

It is known [44,45] that this type of tetrazole by-product may be derived via an alternative pathway of the Schmidt reaction, where the initially formed nitrilium ion is quenched by hydrazoic acid when the latter is present at high concentrations. To exclude the possibility of tetrazole ring formation via a post-amidation reaction, we left pure lactam **9a** to react under our Schmidt protocol conditions, but the latter remained intact. Applying other methods that transform amides to tetrazoles at higher temperatures [46,47] led to the consumption of starting lactam and the formation of parent isoquinolinone **11** and the original nitrone **4a** via an unexpected *retro* 1,3-dipolar cycloaddition reaction (Scheme 5). As it was further concluded, the same *retro* cycloaddition occurs when lactam **9a** is simply heated above 80 °C in an appropriate solvent.

#### 2.2.4. Synthesis of the Targeted Isoxazolidine/Tetrahydroisoquinolinone Hybrids

Having gathered critical insight, we proceeded to prepare a number of isozaxolidine hybrids. The optimised protocol for the Schmidt reaction was applied to both *endo* and *exo* cycloadducts, and the results are summarised in Table 4 and Table 5, respectively. Regardless of the stereoisomer used, this protocol furnished (in most cases, uneventfully) the desired tetrahydroisoquinolinones (**9** and **12**)**,** although some rather distinct differences between the two can be underlined. *Endo* cycloadducts **6** gave the lactams in moderate to very good yields, whereas the *exo* ones (**7**) furnished the analogous lactams in excellent yields in almost every case. The reaction time was also notably shorter for *exo* cycloadducts compared to the *endo* ones. Furthermore, the tetrazole by-product of *exo* analogs was formed in almost every instance. Presumably, the difference in the conformation of the five-membered heterocyclic ring holds the answer to this overall quite different behaviour, but a complete explanation cannot be deduced from the existing data.

### 2.3. Synthesis of Isoxazole/Isoquinolinone Hybrids

#### 2.3.1. Model 1,3-dipolar Cycloaddition Reaction between Indenone and Benzonitrile Oxide and the Following Schmidt Reaction

**Scheme 6:**
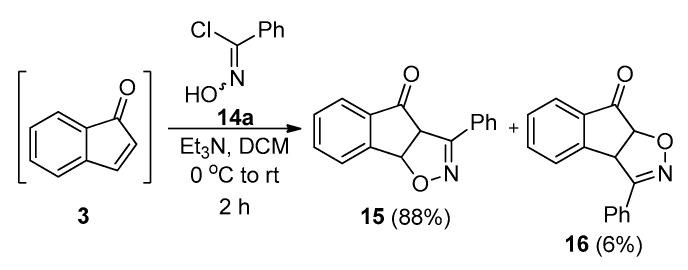
1,3-dipolar cycloaddition between indenone **3** and benzonitrile oxide **14a**.

The 1,3-dipolar cycloaddition reaction between indenone **3** and nitrile oxides was planned as a one-pot procedure. Treating oximes **6** with NCS in DMF furnished the corresponding hydroxamoyl chlorides **14** [48], which were then used without further purification. In the model reaction, one equivalent of Et_3_N was added to the solution of the in situ prepared indenone **3** before the dropwise addition of phenylhydroxamoyl chloride (Scheme 6). This reaction smoothly furnished the two regioisomeric adducts (**15** and **16**), which were isolated and characterised. Once again, the major regioisomer **15** was the one favoured due to the α,β-unsaturated system present in the dipolarophile.

**Scheme 7:**
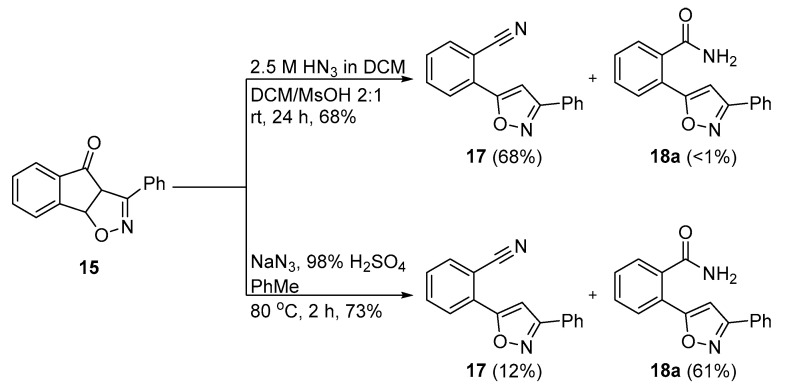
Schmidt reaction on ketone isoxazoline **15**.

Isoxazoline cycloadduct **15** was then submitted to the previously optimised Schmidt reaction protocol (i.e., entry 11, Table 3). To our surprise, this reaction did not furnish the desired lactam, but instead nitrile **17** was isolated as the main product. Traces of the corresponding isoxazole/benzamide **18a** were also identified in the reaction mixture (Scheme 7). Alternatively, heating of **15** in the presence of sodium azide (NaN_3_) and concentrated sulfuric acid gave benzamide **18a** as the major product. Obviously, the latter derived upon hydrolysis of the initially formed nitrile **17**.

**Scheme 8:**
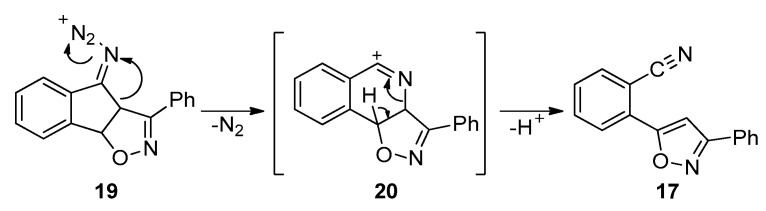
Nitrile/isoxazole formation as a result of a Beckmann fragmentation.

Both the Schmidt reaction and the Beckmann rearrangement share the same mechanistic pathway once the nitrogen migration occurs. Nitriles are common side products generated from the Beckmann rearrangement, and when those are the major products, the transformation is named “Beckmann fragmentation” instead [49]. We assume that in our case, this fragmentation is greatly favoured because the intermediate nitrilium ion **20** readily collapses to the significantly more stable aromatic isoxazole **17** (Scheme 8).

#### 2.3.2. Revision of the Synthetic Plan

**Scheme 9:**
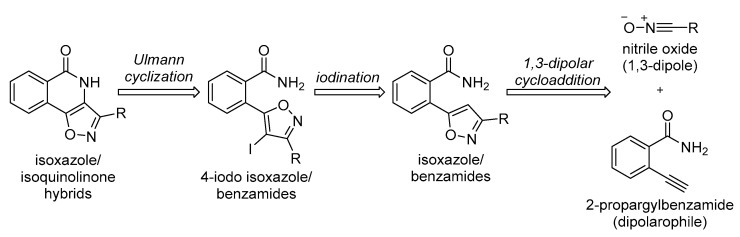
Revised retrosynthetic plan towards isoxazole/isoquinolinone hybrids.

The unforeseen outcome of the Schmidt reaction on isoxazoline adduct **15** made us reconsider our synthetic approach towards the targeted isoxazole/isoquinolinone hybrids. Interestingly, derivative **17a** attracted our attention because it incorporates the isoxazole/benzamide framework, which is ultimately present in our targeted hybrid compounds. Thus, a revised short, synthetic route involving isoxazole/benzamide derivatives was designed. According to this plan, cyclisation of the appropriate 4-iodo isoxazole intermediates via a copper-catalysed Ullmann-type reaction could afford the desired isoxazole/isoquinolinone hybrids. The required iodo derivatives could be prepared via an iodination reaction of the corresponding isoxazole/benzamides, which simply represent the adducts of 1,3-dipolar cycloadditions between 2-propargylbenzamide, now serving as the new dipolarophile, and various in situ prepared nitrile oxides (Scheme 9).

#### 2.3.3. Isoxazole/Benzamides via an 1,3-dipolar Cycloaddition Reaction

A Sonogashira cross coupling reaction to commercially available 2-iodobenzamide and subsequent cleavage of the TMS group, following a typical procedure, afforded 2-propargylbenzamide **21** [50]. A series of in situ generated nitrile oxides (see Scheme 6) reacted with propargyl dipolarophile **21** to give isoxazole/benzamides **18** as single products and in very good yields (Table 6).

#### 2.3.4. Iodination of the Isoxazole Ring

The isoxazole heterocycle is prone to undergoing various transformations, including halogenation. The protocol for the iodination of the 4-position of the isoxazole ring of our derivatives **18** involved treatment with NIS (*N*-iodosuccinimide) in trifluoroacetic acid under microwave irradiation [51]. This afforded the 4-iodoisoxazoles **22** in excellent yields for most of the substrates (Table 7). Exceptions were the analogs bearing the mesityl, furanyl, and thiophene groups (**18j**, **18m**, and **18n**, respectively), which failed to exclusively give the desired products. Instead, complicated mixtures of mono- and polyiodinated products were obtained, regardless of the equivalents of NIS used. It is also worth mentioning that two equivalents of NIS were used for isoxazole **18c**, due to its facile concomitant iodination on the electronically rich p-methoxyphenyl ring.

#### 2.3.5. Optimisation Studies for the Ullmann Reaction

The copper-catalysed C–N bond formation (Ullmann reaction) remains an appealing alternative for the Buchwald–Hartwig reaction, because the latter depends on more expensive and less stable palladium catalysts. A thorough screening to determine the best conditions (copper catalyst, base, solvent, and temperature) was conducted on iodo-isoxazole **22a** (Table 8). Copper(I) thiophene-2-carboxylate (CuTC), being a useful reagent in such reactions, gave inarguably superior results in comparison to the more commonly used copper(I) iodide (CuI) [52]. Typical inorganic bases and solvents were tested, and the best results were obtained upon using K_2_CO_3_ and DMF while maintaining the temperature at 80 °C (entry 10).

#### 2.3.6. Building a Library of Isoxazole/Isoquinolinone Hybrids via the Ullmann-Type Cyclisation

The optimum conditions for Ullmann cyclisation were, thereafter, applied to the rest of the iodo-isoxazoles (Table 9). Analogs bearing a p-substituted aryl group afforded the desired isoquinolinones in very good yields (67–93%). Isoquinolinones where the aryl group is ortho-substituted were isolated in moderate yields (58–61%), whereas analogs with polysubstituted aryl groups furnished the targeted products in generally low yields (29–46%). Nitro analog **22i** failed to give the Ullmann cyclisation because it gradually decomposed during the reaction. Moreover, isoquinolinone derivative **23c** was subjected to a reductive deiodination employing 10% Pd/C to furnish isoquinolinone **24**.

## 3. Experimental Section

### 3.1. General Information

All anhydrous reactions were performed under an argon atmosphere using oven-dried (120 °C) or flame-dried glassware (under vacuum) with dry solvents under anhydrous conditions. THF, 1,4-dioxane, and toluene were distilled over sodium/benzophenone under an argon atmosphere into a dry Schlenk Kjeldahl storage flask containing activated molecular sieves (4 Å), and they were allowed to stand for at least for 24 h. DCM was distilled over calcium hydride (CaH_2_) before use. Carbon tetrachloride was distilled over phosphorus pentoxide before use. All reactions requiring high temperatures were conducted using silicon oil baths as the heating medium. Flash column chromatography was performed by employing silica gel 60 (40–63 μm, Merck). Reactions were monitored through TLC using 0.25 mm silica gel 60 F_254_ plates purchased from Merck. TLC plates were visualised through exposure to ultraviolet light (UV) and/or exposure to an acidic solution of *p*-anisaldehyde or a solution of ninhydrin stain, followed by heating with a heat gun (400 °C). All commercially available reagents and solvents were purchased from Fluorochem, Sigma-Aldrich & Merck, Fischer Scientific, and TCI Chemicals and used as such. Molecular sieves (3 Å and 4 Å) were dried under a high vacuum by being heated with a propane torch in a round-bottom flask for 1–2 min, and the procedure was repeated 2–3 times. Celite^®®^ 545 was purchased from Fluorochem. ^1^H, ^13^C, ^19^F, and 2D NMR spectra were recorded with an Agilent-500/54 spectrometer. Unless otherwise stated, all NMR spectra were recorded at 25 °C. Proton chemical shifts are reported in parts per million (δ scale) and are calibrated relative to a residual nondeuterated solvent as an internal reference (CDCl_3_: δ 7.26, DMSO-*d*_6_: δ 2.5 ppm). Carbon chemical shifts are reported in parts per million (δ scale) and are referenced from the central peak of the carbon resonance of the solvent (CDCl_3_: 77.00, DMSO-*d*_6_: 40.00 ppm). Infrared (IR) data were recorded in a scan range from 400 to 4000 cm^−1^ on a Thermo Scientific Nicolet 6700 FT-IR spectrometer equipped with a diamond attenuated total reflection (ATR) stage. HRMS data were acquired using an Agilent 6540 HRMS-QTOF model equipped with a Dual AJS ESI-MS system or with a Q-TOF (Time of Flight Mass Spectrometry) Maxis Impact (Bruker Daltonics, Bremen, Germany) with ESI source and U-HPLC Thermo Dionex UltiMate 3000 RSLC (ThermoFisher Scientific, Dreieich, Germany) pump and autosampler. Melting points were determined on a A.KRÜSS Optronic Melting Point Meters KSP1N model apparatus. Reactions under microwave irradiation were carried out using a Biotage Initiator+ microwave synthesiser.

### 3.2. Synthesis of 3-Bromo-2,3-dihydro-1H-inden-1-one (***2***)

Compound **2** was prepared following a modified procedure of that reported in the literature [20]. 1-Indanone (10.04 g, 76.0 mmoles, 1.0 equiv.) was dissolved in anhydrous CCl_4_ (150 mL), and then *N*-bromosuccinimide (13.52 g, 76.0 mmoles, 1.0 equiv.) and AIBN (0.125 g, 0.76 mmoles, 0.01 equiv.) were sequentially added at room temperature. The resulting suspension was refluxed for 4 h in an oil bath protected from light, allowed to cool to room temperature, and then filtered through a short pad of Celite. After evaporation of the solvent under reduced pressure, the crude mixture was purified through flash column chromatography (silica gel, *n*-hexane/Et_2_O 35:1 *v*/*v*) to give **2** (11.23 g, 70% yield) as an orange oil, which solidified in the freezer. *R*_f_ = 0.43 (*n*-hexane/EtOAc 4:1 *v*/*v*). All spectroscopic data were in accordance with those reported in the literature [53].

### 3.3. General Procedure for the Preparation of Nitrones ***4***

To a round bottom flask containing *N*-benzylhydroxylamine hydrochloride (2.0 equiv.), anhydrous DCM (40 mL/g) was added under an argon atmosphere. To the resulting suspension of MgSO_4_ (2.0 equiv.), the corresponding aldehyde (1.1 equiv.) and NaHCO_3_ (1.1 equiv.) were added in that order, and the mixture was stirred for 24 h at room temperature. The reaction mixture was then filtered through a short pad of Celite, and the solvent was evaporated under reduced pressure. The crude mixture was purified through flash column chromatography (silica gel, *n*-hexane/EtOAc 5:1 *v*/*v* to 1:1 *v*/*v*) to give the desired nitrones **4**.

### 3.4. General Procedure for the Preparation of Oximes ***5***

Oximes **5** were prepared following a procedure reported previously in the literature [28].

### 3.5. General Procedure for the Preparation of Isoxazolidine Cycloadducts ***6*** and ***7***

3-Bromo-1-indanone (**2**, 0.726 g, 3.44 mmoles, 1.2 equiv.) was dried azeotropically through evaporation with PhMe (3x10 mL) and dissolved in anhydrous PhMe (20 mL) under an argon atmosphere. The mixture was placed in an ice bath, and anhydrous Et_3_N (0.48 mL, 3.44 mmoles, 1.2 equiv.) was added dropwise. After stirring for 15 min at 0 °C, AgOTf (0.185 g, 0.72 mmoles, 0.25 equiv.) and a solution of the corresponding nitrone (2.87 mmoles, 1 equiv.) in anhydrous PhMe (3 mL) were added to the reaction mixture, which was then placed in an oil bath at 80 °C. After stirring for 24 h at that temperature, the reaction mixture was cooled to room temperature, diluted with a small amount of DCM, and filtered through a short pad of Celite. The solvent was evaporated under reduced pressure, and the crude mixture was purified through flash column chromatography (silica gel, *n*-hexane/EtOAc 20:1 *v*/*v* to 12:1 *v*/*v*) to give the corresponding cycloadducts **6** and **7** (and, in some cases, regioisomers **8**).

### 3.6. General Procedure for the Preparation of Hybrids ***9*** and ***12***

A solution of HN_3_ (2.5 M) in DCM was prepared by dissolving NaN_3_ (0.650 g, 10 mmoles) in H_2_O (1 mL), adding the organic solvent (4 mL), cooling the mixture to −10 °C, and slowly adding MsOH (1 mL) over a period of 20 min under vigorous stirring. The mixture was stirred for another 10 min, and the organic layer was then separated. The starting ketone (**6** or **7**, 0.15 mmoles) was dissolved to this solution (3 mL), and the mixture was stirred for 30 min at room temperature. MsOH (1.5 mL) was added dropwise over a period of 3 h. The resulting mixture was stirred for 24–72 h at room temperature, diluted with DCM (5 mL), placed in an ice bath, and slowly quenched through the addition of a saturated aqueous Na_2_CO_3_ solution until effervescence ceased. The organic layer was separated, and the aqueous layer was extracted with DCM (3 × 10 mL). The organic layers were combined, washed with brine (40 mL), and dried over anhydrous Na_2_SO_4_. After evaporation of the solvent under reduced pressure, the crude mixture was purified through flash column chromatography (silica gel, *n*-hexane/EtOAc 5:1 to 1:1 *v*/*v*) to give the desired isoxazolidine/tetrahydroisoquinolinone hybrids **9** and **12** (and tetrazole by-products **10** and **13**, respectively).

### 3.7. Synthesis of Isoxazoline Cycloadducts ***15*** and ***16***

3-Bromo-1-indanone (**2**, 1.00 g, 4.74 mmoles, 1 equiv.) was dissolved in DCM (50 mL), and the mixture was placed in an ice bath. Et_3_N (0.79 mL, 5.69 mmoles, 1.2 equiv.) was added dropwise, and the yellow solution was stirred for 15 min at 0 °C. A DCM solution (100 mL) of phenylhydroxamoyl chloride **14a** (0.811 g, 5.21 mmoles, 1.1 equiv.) was then added via a pressure-equalising dropping funnel over a period of 1 h, while the temperature was maintained at 0 °C. After the addition, the reaction mixture was left stirring for another 1 h, and the reaction temperature was gradually allowed to reach 20 °C. The reaction mixture was quenched through the addition of aqueous semi-saturated aqueous NaCl solution (150 mL), the organic layer was separated, and the aqueous layer was extracted with DCM (3 × 150 mL). The organic layers were combined, washed with brine (500 mL), and dried over anhydrous Na_2_SO_4_. After evaporation of the solvent under reduced pressure, the crude mixture was purified through flash column chromatography (silica gel, *n*-hexane/EtOAc 12:1 to 10:1 *v*/*v*) to give cycloadducts **15** (1.04 g, 88% yield) and **16** (0.071 g, 6% yield).

*3-Phenyl-3a,8b-dihydro-4H-indeno [2,1-d]isoxazol-4-one (**15**)*: white solid; m.p. 153–154 °C; *R*_f_ = 0.31 (*n*–hexane/EtOAc 4:1 *v*/*v*); ^1^H NMR (500 MHz, CDCl_3_): δ = 8.03–7.96 (m, 2H), 7.82 (d, *J* = 7.7 Hz, 1H), 7.79–7.73 (m, 2H), 7.55 (t, *J* = 7.4 Hz, 1H), 7.46–7.41 (m, 3H), 6.31 (d, *J* = 8.3 Hz, 1H), 4.75 (d, *J* = 8.3 Hz, 1H) ppm; ^13^C NMR (125 MHz, CDCl_3_): δ = 197.2, 152.6, 150.7, 136.3, 134.5, 130.6, 130.4, 128.6, 128.0, 127.9, 126.9, 124.2, 83.0, 60.6 ppm; FT-IR (neat): ν = 3057, 2941, 1723, 1604, 1590, 1334, 1269, 884, 761, 696 cm^–1^; HRMS (ESI), *m*/*z*: [M + Na]^+^ calcd for C_16_H_11_NNaO_2_^+^ 272.0682; 272.0679.

*3-Phenyl-3a,8a-dihydro-8H-indeno [1,2-d]isoxazol-8-one (**16**)*: white solid; m.p. 172–173 °C; *R*_f_ = 0.18 (*n*–hexane/EtOAc 4:1 *v*/*v*); ^1^H NMR (500 MHz, CDCl_3_): δ = 7.85 (d, *J* = 7.7 Hz, 1H), 7.83–7.79 (m, 2H), 7.53 (td, *J* = 7.5, 1.3 Hz, 1H), 7.50–7.47 (m, 3H), 7.43 (t, *J* = 7.9 Hz, 2H), 5.42 (d, *J* = 8.6 Hz, 1H), 5.33 (d, *J* = 8.6 Hz, 1H) ppm; ^13^C NMR (125 MHz, CDCl_3_): δ = 198.7, 156.9, 150.3, 136.1, 134.4, 130.6, 129.3, 128.1, 128.0, 127.6, 126.2, 125.7, 84.7, 53.1 ppm; FT-IR (neat): ν = 3058, 2962, 1716, 1592, 1444, 1347, 1251, 881, 759, 694 cm^–1^. HRMS (ESI), *m*/*z*: [M + Na]^+^ calcd for C_16_H_11_NNaO_2_^+^ 272.0682; 272.0686.

### 3.8. Synthesis of Nitrile ***17*** and Benzamide ***18a*** from ***15***

Procedure A: The solution of HN_3_ (2.5 M) in DCM was prepared as described in Section 3.6. Isoxazoline adduct **15** (0.424 g, 1.7 mmoles, 1 equiv.) was dissolved in DCM (6 mL, 2.5 M HN_3_ solution) and left stirring for 30 min at room temperature. MsOH (3 mL) was added dropwise over a period of 3 h. The resulting mixture was stirred for 24 h at room temperature, diluted with DCM (15 mL), placed in an ice bath, and slowly quenched through the addition of saturated aqueous Na_2_CO_3_ solution until effervescence ceased. The organic layer was separated, and the aqueous layer was extracted with DCM (3 × 20 mL). The organic layers were combined, washed with brine (80 mL), and dried over anhydrous Na_2_SO_4_. After evaporation of the solvent under reduced pressure, the crude mixture was purified through flash column chromatography (silica gel, *n*-hexane/EtOAc 10:1 to 1:1 *v*/*v*) to give nitrile **17** (0.247 g, 68% yield) and benzamide **18a** (4 mg, 1%).

Procedure B: Cycloadduct **15** (0.112 g, 0.45 mmoles, 1 equiv.) was dissolved in PhMe (5 mL), and the clear solution was placed in an ice bath. NaN_3_ (0.088 g, 1.35 mmoles, 3 equiv.) and H_2_SO_4_ (98%, 3 µL, 0.045 mmoles, 0.1 equiv.) were sequentially added at 0 °C, and the reaction mixture was then placed in an oil bath at 80 °C. After stirring for 2 h, the mixture was allowed to cool to toom temperature, placed in an ice bath, diluted with a small amount of EtOAc, and slowly quenched through the addition of saturated aqueous Na_2_CO_3_ solution until effervescence ceased. The organic layer was separated, and the aqueous layer was extracted with EtOAc (3 × 10 mL). The organic layers were combined, washed with brine (30 mL), and dried over anhydrous Na_2_SO_4_. After evaporation of the solvent under reduced pressure, the crude mixture was purified through flash column chromatography (silica gel, *n*-hexane/EtOAc 10:1 to 1:1 *v*/*v*) to give nitrile **17** (0.013 g, 12% yield) and benzamide **18a** (0.081 g, 61%).

*2-(3-phenylisoxazol-5-yl)benzonitrile (***17***)*: white solid; *R*_f_ = 0.53 (*n*-hexane/EtOAc 3:1 *v*/*v*). All spectroscopic data are in accordance with those reported in the literature [54].

*2-(3-phenylisoxazol-5-yl)benzamide (***18a***)*: white solid; m.p. 159–160 °C; *R*_f_ = 0.11 (*n*-hexane/EtOAc 1:1 *v*/*v*); ^1^H NMR (500 MHz, DMSO-*d*_6_): δ = 7.99 (br s, 1H), 7.89 (dd, *J* = 7.8, 1.8 Hz, 2H), 7.83–7.79 (m, 1H), 7.63–7.50 (m, 7H), 7.25 (s, 1H) ppm; ^13^C NMR (125 MHz, DMSO-*d*_6_): δ = 170.6, 169.8, 162.6, 137.5, 130.8, 130.7, 130.0, 129.7, 129.0, 128.9, 128.3, 127.0, 124.7, 101.1 ppm. FT-IR (neat): ν = 3332, 3158, 2804, 1663, 1623, 1464, 1401, 1137, 950, 758, 690 cm^–1^. HRMS (ESI), *m*/*z*: [M + Na]^+^ calcd for C_16_H_12_N_2_NaO_2_^+^ 287.0791; found 287.0795.

### 3.9. Synthesis of 2-Propargylbenzamide (***21***)

Compound **21** was prepared following a procedure reported previously in the literature [51].

### 3.10. General Procedure for the Preparation of Hydroxamoyl Chlorides ***14***

Hydroxamoyl chlorides **14** were prepared following a procedure reported previously in the literature [49].

### 3.11. General Procedure for the Preparation of Isoxazole/Benzamides ***18***

2-propargyl benzamide (**21,** (0.164 g, 1.13 mmoles) was dissolved in DCM (6 mL) and placed in an ice bath. Et_3_N (0.19 mL, 1.36 mmoles, 1.2 equiv.) was added, and the reaction mixture was stirred for 10 min at 0 °C. A DCM solution (4 mL) of hydroxamoyl chloride (**14**, 1.36 mmoles, 1.2 equiv.) was then added dropwise over a period of 15 min, and the mixture was left stirring at 0 °C for 1 h. The reaction mixture was gradually allowed to reach room temperature and stirred for another 4 h before it was quenched through the addition of semi-saturated aqueous NaCl solution (20 mL). The organic layer was separated, and the aqueous layer was extracted with DCM (3 × 15 mL). The organic layers were combined, washed with brine (60 mL), and dried over anhydrous Na_2_SO_4_. After evaporation of the solvent under reduced pressure, the crude mixture was purified through flash column chromatography (silica gel, *n*-hexane/EtOAc 5:1 to 1:2 *v*/*v*) to afford the corresponding isoxazole/benzamides **18**.

### 3.12. General Procedure for the Preparation of 4-Iodo Isoxazoles ***22***

To a proper heavy-wall microwave reaction vial containing an isoxazole/benzamide **18** (0.58 mmoles), TFA (2 mL) and *N*-iodosuccinimide (0.137 g, 0.61 mmoles, 1.05 equiv.) were added at room temperature. The vial was then sealed and stirred under microwave irradiation at 80 °C for 10 min. The light purple solution was diluted with DCM (10 mL), placed in an ice bath, and neutralised with a saturated, aqueous NaHCO_3_ solution. The organic layer was separated, and the aqueous layer was extracted with DCM (2 × 15 mL). The organic layers were combined, washed with brine (40 mL), and dried over anhydrous Na_2_SO_4_. After evaporation of the solvent under reduced pressure, the crude mixture was purified through flash column chromatography (silica gel, *n*-hexane/EtOAc 5:1 to 1:2 *v*/*v*) to give the corresponding 4-iodo isoxazole/benzamides **22**.

### 3.13. General Procedure for the Preparation of Isoxazole/Isoquinolinones ***23***

DMF (3 mL) was added to a heavy-wall sealed tube containing a 4-iodo isoxazole/benzamide **22** (0.29 mmoles) at room temperature. K_2_CO_3_ (0.08 g, 0.58 mmoles, 2 equiv.) and CuTC (0.014 g, 0.072 mmoles, 0.25 equiv.) were sequentially added, and the light green suspension was flushed with argon for 2–3 min. The tube was tightly sealed, and the mixture was stirred at 80 °C for 24 h. The dark greenish/brown suspension was diluted with EtOAc (5 mL) and quenched with the addition of an aqueous NH_4_OH-NH_4_Cl solution (10 mL). The organic layer was separated, and the aqueous layer was extracted with EtOAc (3 × 10 mL). The organic layers were combined, washed with brine (40 mL), and dried over anhydrous Na_2_SO_4_. After evaporation of the solvent under reduced pressure, the resulting solid residue was purified through recrystallisation (dissolved in 10% TFA in DCM and crystallised upon adding a few drops of MeOH) to afford the corresponding isoxazole/isoquinolinones **23**.

### 3.14. Synthesis of Isoquinolinone ***24*** via Reductive Deiodination

DMF (1 mL) was added to a Schlenk tube containing isoquinolinone **23c** (21 mg, 0.051 mmoles) at room temperature. Then, 10% Pd/C (3 mg) and a couple of drops of Et_3_N were sequentially added, and the reaction vessel was purged with H_2_ and placed under an atmosphere of H_2_. After stirring for 1 h at room temperature, the resultant slurry was diluted with EtOAc (10 mL), filtered through a short a pad of Celite, and semi-saturated, aqueous NaCl solution (15 mL) was added. The organic layer was separated, and the aqueous layer was extracted with EtOAc (3 × 10 mL). The organic layers were combined, washed with brine (40 mL), and dried over anhydrous Na_2_SO_4_. After evaporation of the solvent under reduced pressure, the resulting solid residue was purified through recrystallisation (dissolved in 10% TFA in DCM and crystallised upon adding a few drops of MeOH) to give pure **24** (15 mg, 98% yield).

*3-(4-methoxyphenyl)isoxazolo [4,5-c]isoquinolin-5(4H)-one (***24***)*: white solid; m.p. > 250 °C (decomposed); *R*_f_ = 0.4 (*n*-hexane/EtOAc 1:1 *v*/*v*); ^1^H NMR (500 MHz, 4% TFA-*d* in CDCl_3_): δ = 8.54 (d, *J* = 8.1 Hz, 1H), 8.19 (d, *J* = 7.9 Hz, 1H), 7.99 (t, *J* = 7.6 Hz, 1H), 7.83–7.77 (m, 3H), 7.11 (d, *J* = 8.3 Hz, 2H), 3.91 (s, 3H) ppm; ^13^C NMR (125 MHz, 4% TFA-*d* in CDCl_3_): δ = 164.4, 161.9, 152.2, 151.4, 135.0, 130.3, 129.4, 129.3, 125.6, 123.9, 121.6, 117.8, 117.3, 115.1, 55.5 ppm; FT-IR (neat): ν = 3089, 2981, 1663, 1600, 1486, 1346, 1259, 1182, 826, 769 cm^–1^. HRMS (ESI), *m*/*z*: [M + Na]^+^ calcd for C_17_H_12_N_2_NaO_3_^+^ 315.0740; found 315.0746.

## 4. Conclusions

Herein, we presented our investigation of syntheses of novel isoxazolidine and isoxazole isoquinolinone hybrids. According to our original retrosynthesis, those fused heterocyclic compounds could be prepared via an 1,3-dipolar cycloaddition of indenone with nitrones and nitrile oxides and a subsequent Schmidt reaction. The cycloaddition with nitrones was found to be regioselective and, to some extent, depending on the reaction conditions, stereoselective, thus favouring the endo-adducts. Both endo- and exo-stereoisomers were uneventfully subjected to the Schmidt reaction to give the corresponding desired lactams (isoxazolidine/isoquinolinone hybrids). Although this scenario proved successful for isoxazolidine derivatives, a Beckmann fragmentation occurred when the Schmidt reaction protocol was applied on the indenone–benzonitrile oxide adduct. Thus, an alternative approach was adopted, which first involved an 1,3-dipolar cycloaddition reaction of an appropriate alkyne with nitrile oxides, and then an Ullmann type cyclisation of the corresponding iodinated isoxazoles, to furnish the desired isoxazole/isoquinolinone hybrids. Differentially substituted dipoles were used to obtain small libraries of each category of hybrids. The overall syntheses represent short and relatively straightforward pathways towards these new classes of compounds, which will be evaluated in the future for their biological activity.

## Figures and Tables

**Table 1:** Optimisation studies for the 1,3-dipolar cycloaddition reaction of **3** with nitrone **4a**.

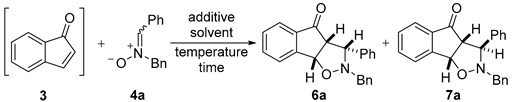									
Entry	3 (equiv.)	4a ^1^ (equiv.)	Solvent ^2^	Temp. (°C)	Additive (0.25 equiv.)	Time (h)	6a ^5^	7a ^5^	Yield ^5^ (comb.)
1	1	1	PhMe	110	-	24	40%	30%	70%
2	1	1	DCM	25	-	72	18%	7%	25%
3	1	1	none	80	-	2	32.5%	15.5%	48%
4	1	1	DCM	25	Cu(OTf)_2_	24	10%	<1%	10%
5	1	1	PhMe	40	Sc(OTf)_3_	72	43%	20%	63%
6	1	1	PhMe	40	Mg(OTf)_2_	72	39.5%	25.5%	65%
7	1.2	1	PhMe	60	Zn(OTf)_2_	24	50%	28%	78.5%
8	1.2	1	PhMe	60	Zn(OTf)_2_	48	54.5%	25.5%	80%
9	1.2	1	PhMe	60	In(OTf)_3_	48	35%	20.5%	55.5%
10	1.2	1	PhMe	60	AgOTf	72	45.5%	36%	81.5%
11 ^3^	1.2	1	PhMe	80	Zn(OTf)_2_	24	50%	34%	84%
12	1.2	1	PhMe	80	AgOTf	24	50%	40%	90%
13	1.2	1	PhMe	80	Cu(OTf)_2_	24	31%	19%	50%
14 ^4^	1.2	1	PhMe	80	Zn(OTf)_2_	3	34.5%	18.5%	53%
15 ^4^	1.2	1	PhMe	100	AgOTf	4	46%	34%	80%
16 ^3^	1	1	PhMe	25	SnCl_4_	24	-	-	-
17 ^3^	1	1	PhMe	25	TiCl_4_	24	-	-	-
18	1.2	1	PhMe	80	Ag_2_CO_3_	24	45%	35.5%	80.5%
19	1.2	1	PhMe	80	Ag_2_O	24	45%	35%	80%

^1^ 1 mmole of **4a** used; ^2^ 7 mL/mmole of **4a**; ^3^ 1 equiv. of the additive was used; ^4^ the reaction was carried out under μW irradiation; ^5^ yield after purification through flash column chromatography based on the amount of nitrone used.

**Table 2:** Synthesis of isoxazolidine cycloadducts using various nitrones.

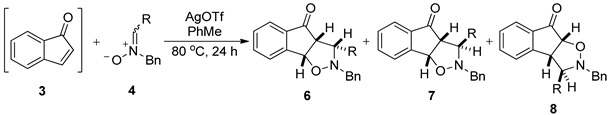					
Entry	R =	*endo* Cycloadduct	*exo* Cycloadduct	Regio Cycloadduct	Yield ^1^ (comb.)
1	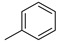	**6a** (50%)	**7a** (40%)	-	90%
2	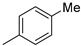	**6b** (40%)	**7b** (37%)	-	77%
3	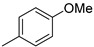	**6c** (47%)	**7c** (24%)	-	71%
4	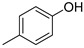	**6d** (45%)	**7d** (26%)	-	71%
5	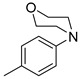	**6e** (43%)	**7e** (26%)	-	69%
6	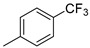	**6f** (54%)	**7f** (24%)	-	78%
7	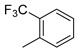	**6g** (31%)	**7g** (24%)	**8g** (16%)	71%
8	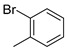	**6h** (28%)	**7h** (20%)	**8h** (23%)	71%
9	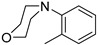	**6i** (42%)	**7i** (34%)	-	76%
10	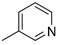	**6j** (55%)	**7j** (27%)	-	82%
11	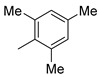	**6k** (32%)	**7k** (20%)	-	52%
12	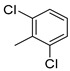	**6l** (40%)	-	-	40%
13	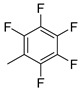	**6m** (18%)	**7m** (37%)	-	55%
14	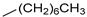	**6n** (21%)	**7n** (55%)	-	76%

^1^ yield after purification through flash column chromatography based on the amount of nitrone used.

**Table 3:** Investigation of the Schmidt reaction conditions on **6a**.

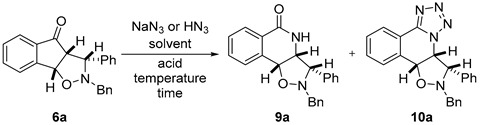								
Entry	Acid	Reagent	Solvent ^4^	Temp. (°C)	Time (h)	9a ^5^	10a ^5^	Yield ^5^ (comb.)
1 ^1^	aq. HCl (37%)	NaN_3_	PhMe	25	24	-	-	rsm
2 ^1^	aq. HCl (37%)	NaN_3_	PhMe	50	24	-	-	rsm
3 ^2^	aq. HCl (37%)	NaN_3_	-	80	24	-	-	rsm
4 ^2^	TFA	NaN_3_	-	25	24	-	-	rsm
5 ^2^	TFA	NaN_3_	-	72	24	-	-	rsm
6 ^1^	TfOH	NaN_3_	PhMe	25	24	-	-	rsm
7 ^2^	TfOH	NaN_3_	-	25	24	-	-	rsm
8 ^1^	H_2_SO_4_	NaN_3_	PhMe	80	24	15%	-	15%
9 ^1^	MsOH	NaN_3_	PhMe	25	24	<10%	-	~10% + rsm
10 ^3^	MsOH	HN_3_	PhMe	25	48	76%	13%	89%
11 ^3^	MsOH	HN_3_	DCM	25	48	78%	14%	92%

^1^ 0.1 mmoles of **6a**, 3 equiv. NaN_3_, 0.1 equiv. of the acid; ^2^ 0.1 mmoles of **6a**, 3 equiv. NaN_3_, acid is used as the solvent; ^3^ 0.1 mmoles of **6a**, 2.5M HN_3_ in solvent, ratio of solvent/MsOH 2:1; ^4^ 20 mL/mmole of **6a**; ^5^ yield after purification through flash column chromatography; rsm = recovered starting material.

**Table 4:** Synthesis of tetrahydroisoquinolinones **9**.

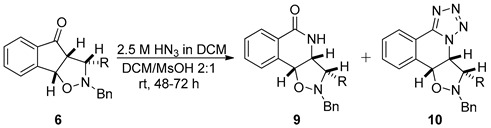				
Entry	R =	Lactam ^1^	Tetrazole ^1^	Combined Yield ^1^
1	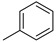	**9a** (78%)	**10a** (14%)	89%
2	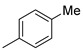	**9b** (59%)	**10b** (19%)	78%
3	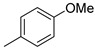	**9c** (62%)	-	62%
4	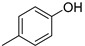	**9d** (54%)	-	54%
5	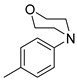	**9e** (81%)	-	81%
6	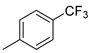	**9f** (70%)	-	70%
7	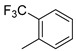	**9g** (65%)	**10g** (12%)	77%
8	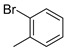	**9h** (65%)	**10h** (17%)	82%
9	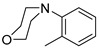	**9i** (51%)	-	51%
10	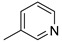	**9j** (53%)	-	53%
11	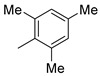	**9k** (90%)	-	90%
12	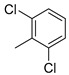	**9l** (72%)	**10l** (13%)	85%
13	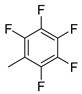	**9m** (65%)	-	65%
14	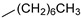	**9n** (69%)	**10n** (11%)	80%

^1^ yield after purification through flash column chromatography.

**Table 5:** Synthesis of tetrahydroisoquinolinones **12**.

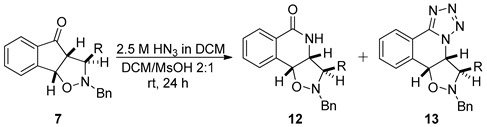				
Entry	R =	Lactam ^1^	Tetrazole ^1^	Combined Yield ^1^
1	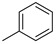	**12a** (82%)	**13a** (14%)	96%
2	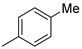	**12b** (72%)	**13b** (14%)	86%
3	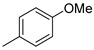	**12c** (83%)	**13c** (14%)	97%
4	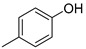	**12d** (75%)	-	75%
5	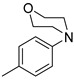	**12e** (84%)	**13e** (14%)	98%
6	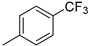	**12f** (85%)	**13f** (14%)	99%
7	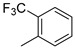	**12g** (82%)	**13g** (14%)	96%
8	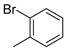	**12h** (87%)	**13h** (12%)	99%
9	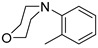	**12i** (84%)	**13i** (8%)	92%
10	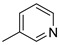	**12j** (64%)	-	64%
11	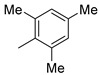	-	-	decomposed
13	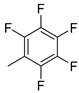	**12m** (81%)	**13m** (12%)	93%
14	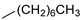	**12n** (90%)	**13n** (9%)	99%

^1^ yield after purification through flash column chromatography.

**Table 6:** Synthesis of isoxazole/benzamides **18**.

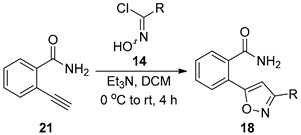					
Entry	R =	Yield ^1^	Entry	R =	Yield ^1^
1	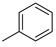	**18a** (84%)	9	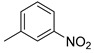	**18i** (83%)
2	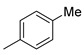	**18b** (89%)	10 ^2^	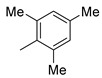	**18j** (90%)
3	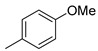	**18c** (87%)	11 ^3^	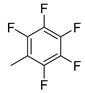	**18k** (84%)
4	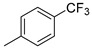	**18d** (86%)	12 ^2^	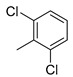	**18l** (93%)
5	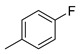	**18e** (88%)	13	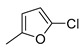	**18m** (80%)
6	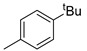	**18f** (91%)	14	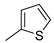	**18n** (79%)
7	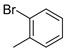	**18g** (92%)	15	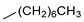	**18o** (92%)
8	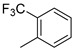	**18h** (89%)			

^1^ yield after purification through flash column chromatography; ^2^ PhMe was used instead of DCM and the reaction mixture was refluxed; ^3^ the reaction mixture was heated to 40 °C.

**Table 7:** Iodination of isoxazole derivatives **18**.

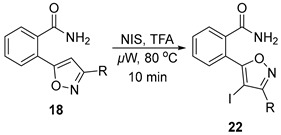					
Entry	R =	Yield ^1^	Entry	R =	Yield ^1^
1	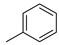	**22a** (97%)	9 ^3^	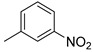	**22i** (99%)
2	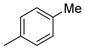	**22b** (99%)	10	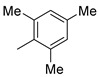	-
3 ^2^	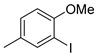	**22c** (88%)	11	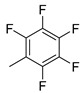	**22k** (99%)
4	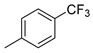	**22d** (99%)	12	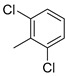	**22l** (98%)
5	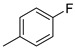	**22e** (97%)	13	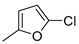	-
6	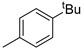	**22f** (97%)	14	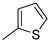	-
7	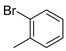	**22g** (99%)	15	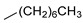	**22o** (98%)
8	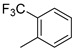	**22h** (99%)			

**^1^** yield after purification through flash column chromatography; ^2^ 2 equiv. of NIS were used; ^3^ yield after purification through trituration with Et_2_O/n-hexane.

**Table 8:** Ullmann cyclisation optimisation studies.

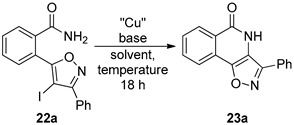					
Entry ^1^	Copper Source (equiv.)	Base (2 equiv.)	Solvent ^2^	Temp. (°C)	Yield ^5^
1	CuI (0.15)	^t^BuONa	^t^BuOH	100	16%
2	CuI (0.15)	^t^BuONa	1,4-dioxane	100	20%
3 ^3^	CuI (0.15)	Cs_2_CO_3_	DMF	100	15%
4	CuI (1)	Cs_2_CO_3_	DMF	100	56%
5	CuTC (0.15)	K_3_PO_4_	DMF	100	50%
6	CuTC (0.25)	K_3_PO_4_	DMF	80	61%
7	CuTC (0.15)	^t^BuONa	DMF	100	55%
8	CuTC (0.15)	Cs_2_CO_3_	DMF	100	56%
9	CuTC (0.25)	Cs_2_CO_3_	DMF	80	73%
10	CuTC (0.25)	K_2_CO_3_	DMF	80	78%
11	CuTC (0.25)	K_2_CO_3_	DMF	120	-
12	CuTC (0.25)	K_2_CO_3_	DMSO	80	31%
13 ^4^	CuTC (0.25)	K_2_CO_3_	DMF	80	48%

^1^ 0.1 mmoles of iodo-benzamide **22a** was used; ^2^ 10 mL/mmole of **22a**; ^3^ 1 equiv. of DMEDA was used as additive; ^4^ 1 equiv. of base was used; ^5^ yield after purification through trituration with Et_2_O/n-hexane.

**Table 9:** Synthesis of isoquinolinones **23**.

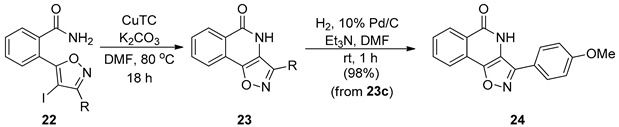					
Entry	R =	Yield ^1^	Entry	R =	Yield ^1^
1	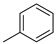	**23a** (78%)	7	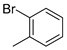	**23g** (58%)
2	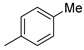	**23b** (67%)	8	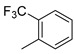	**23h** (61%)
3	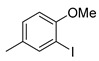	**23c** (93%)	9	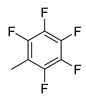	**23k** (29%)
4	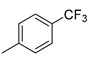	**23d** (81%)	10	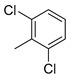	**23l** (46%)
5	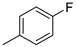	**23e** (89%)	11	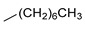	**23o** (80%)
6	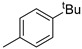	**23f** (78%)			

^1^ yield after purification through recrystallisation (dissolved in 10% TFA in DCM and crystallised upon adding a few drops of MeOH).

## Data Availability

All data supporting the findings of this study are available within the paper and within its Supplementary Materials published online.

## Supplementary Materials

The following supporting information can be downloaded at: https://www.mdpi.com/article/10.3390/molecules29010091/s1. It contains data for compounds **4**–**10**, **12**, **13**, **18b**–**18o**, **22**, and **23** and copies of ^1^H, ^13^C, ^19^F, and 2D NMR spectra of all new compounds.
