# Supplementary material for: A Convenient Synthesis of Novel Isoxazolidine and Isoxazole Isoquinolinones Fused Hybrids
Source: Molecules. 2023 Dec 22;29(1):91. doi: 10.3390/molecules29010091 (PMC10779618; doi:10.3390/molecules29010091)
Supplement: Supplementary file 1 [file molecules-29-00091-s001.zip › molecules-2772781-supplementary.pdf]

A Convenient Synthesis of Novel Isoxazolidine and Isoxazole Isoquinolinones Fused Hybrids

Konstantinos A. Ouzounthanasis [1], Stergios R. Rizos [2] and Alexandros E. Koumbis [1,*]

[1]Laboratory of Organic Chemistry, Department of Chemistry, Aristotle University of Thessaloniki, Thessaloniki 54124, Greece
[2]Department of Chemistry and Chemical Biology, Harvard University, 12 Oxford St., Cambridge, 02138, Massachusetts, USA

*akoumbis@chem.auth.gr

SUPPORTING INFORMATION

Table of Contents

1. Data for compounds **4**……………………………………………………………………S2
2. Data for compounds **5**……………………………………………………………………S4
3. Data for compounds **6**, **7** and **8**……………………………………………………………S7
4. Data for compounds **9** and **10**……………………………………………………………S17
5. Data for compounds **12** and **13**…………………………………………………………...S23
6. Data for compounds **18**……………………………………………………………………S31
7. Data for compounds **22**……………………………………………………………………S34
8. Data for compounds **23**……………………………………………………………………S38
9. Copies of ^{1}H, ^{13}C, ^{19}F and 2D NMR Spectra……………………………………...……S42

1. Data for compounds **4**

(*Z*)-*N*-Benzyl-1-phenylmethanimine oxide (**4a**)

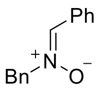

Yield: 97%; white solid; R_f = 0.31 (*n*-hexane/EtOAc 1:1 *v/v*). All spectroscopic data were in accordance with those reported in the literature [**1**].

(*Z*)-*N*-Benzyl-1-(p-tolyl)methanimine oxide (**4b**)

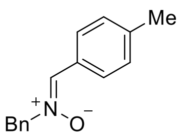

Yield: 97%; white solid; R_f = 0.25 (*n*-hexane/EtOAc 1:1 *v/v*). All spectroscopic data were in accordance with those reported in the literature [**1**].

(*Z*)-*N*-Benzyl-1-(4-methoxyphenyl)methanimine oxide (**4c**)

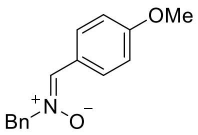

Yield: 99%; white solid; R_f = 0.17 (*n*-hexane/EtOAc 1:1 *v/v*). All spectroscopic data were in accordance with those reported in the literature [**1**].

(*Z*)-*N*-Benzyl-1-(4-hydroxyphenyl)methanimine oxide (**4d**)

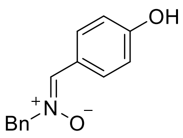

Yield: 91%; white solid; R_f = 0.17 (*n*-hexane/EtOAc 1:3 *v/v*). All spectroscopic data were in accordance with those reported in the literature [**2**].

(*Z*)-*N*-Benzyl-1-(4-morpholinophenyl)methanimine oxide (**4e**)

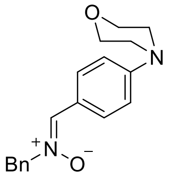

Yield: 99%; m.p. 190–191 °C; white solid; R_f = 0.08 (*n*-hexane/EtOAc 1:2 *v/v*); ^{1}H NMR (500 MHz, $CDCl_3$): δ = 8.16 (d, *J* = 9.1 Hz, 2H), 7.48 – 7.45 (m, 2H), 7.42 – 7.35 (m, 3H), 7.27 (s, 1H), 6.86 (d, *J* = 9.1 Hz, 2H), 5.01 (s, 2H), 3.83 (t, *J* = 4.8 Hz, 4H), 3.24 (t, *J* = 4.8 Hz, 4H) ppm; ^{13}C NMR (125 MHz, $CDCl_3$): δ = 152.2, 134.2, 133.5, 130.3, 129.1, 128.8, 128.7, 121.5, 113.9, 70.4, 66.6, 47.8 ppm; FT-IR (neat): ν = 3067, 2955, 2857, 1601, 1506, 1456, 1229, 1154, 1111, 923, 825, 702 cm^{-1}; HRMS (ESI), *m*/*z*: [M + Na]$^+$ calcd for $C_{18}H_{20}N_2NaO_2^+$ 319.1417; found 319.1410.

(*Z*)-*N*-Benzyl-1-(4-(trifluoromethyl)phenyl)methanimine oxide (**4f**)

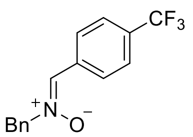

Yield: 99%; white solid; R_f = 0.20 (*n*-hexane/EtOAc 3:1 *v/v*). All spectroscopic data were in accordance with those reported in the literature [**1**].

1 He, C.-T.; Han, X.-L.; Zhang, Y.-X.; Du, Z.-T.; Si, C.-M.; Wei, B.-G. $Sc(OTf)_3$-Catalyzed [3 + 2]-Cycloaddition of Nitrones with Ynones. *Org. Biomol. Chem.* **2021**, *19*, 457–466.
2 Bortolini, o.; Mulani, I.; De Niro, A.; Maiuolo, L.; Nardi, M.; Russo, B.; Avnet, S. Efficient Synthesis of Isoxazolidine-Substituted Bisphosphonates By 1,3-Dipolar Cycloaddition Reactions. *Tetrahedron* **2011**, *67*, 5635–5641.

(*Z*)-*N*-Benzyl-1-(2-(trifluoromethyl)phenyl)methanimine oxide (**4g**)

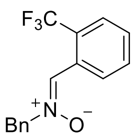

Yield: 98%; white solid; m.p. 54–55 °C; R_f = 0.25 (*n*-hexane/EtOAc 3:1 *v/v*); ^{1}H NMR (500 MHz, $CDCl_3$): δ = 9.40 (d, *J* = 8.1 Hz, 1H), 7.74 (s, 1H), 7.69 (d, *J* = 7.8 Hz, 1H), 7.62 (t, *J* = 7.8 Hz, 1H), 7.50 – 7.47 (m, 3H), 7.46 – 7.41 (m, 3H), 5.14 (s, 2H) ppm; ^{13}C NMR (125 MHz, $CDCl_3$): δ = 133.6, 132.5, 132.1, 129.8, 129.4, 129.3, 129.2, 129.1, 128.8, 127.5, 125.8 (q, *J* = 5.8 Hz), 122.8, 72.2 ppm; ^{19}F NMR (470 MHz, $CDCl_3$): δ = –58.72 ppm; FT-IR (neat): ν = 3078, 3034, 1580, 1424, 1316, 1292, 1153, 1107, 1036, 773, 714 cm^{-1}; HRMS (ESI), *m*/*z*: $[M + Na]^+$ calcd for $C_{15}H_{12}F_3NNaO^+$ 302.0763; found 302.0768.

(*Z*)-N-Benzyl-1-(2-bromophenyl)methanimine oxide (**4h**)

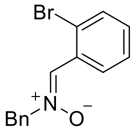

Yield: 98%; white solid; R_f = 0.31 (*n*-hexane/EtOAc 2:1 *v/v*). All spectroscopic data were in accordance with those reported in the literature [**3**].

(Z)-*N*-Benzyl-1-(2-morpholinophenyl)methanimine oxide (**4i**)

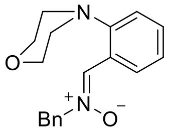

Yield: 99%; white solid; m.p. 144.5–145.5 °C; R_f = 0.20 (*n*-hexane/EtOAc 1:2 *v/v*); ^{1}H NMR (500 MHz, $CDCl_3$): δ = 9.16 (d, *J* = 7.9 Hz, 1H), 7.70 (s, 1H), 7.49 – 7.42 (m, 5H), 7.35 (t, *J* = 7.0 Hz, 1H), 7.15 (t, *J* = 7.6 Hz, 1H), 7.03 (d, *J* = 8.0 Hz, 1H), 5.09 (s, 2H), 3.58 (t, *J* = 3.6 Hz, 4H), 2.78 (t, *J* = 4.5 Hz, 4H) ppm; ^{13}C NMR (125 MHz, $CDCl_3$): δ = 151.3, 133.3, 131.2, 130.9, 129.7, 129.2, 128.8, 124.4, 123.9, 118.7, 71.5, 67.0, 53.4 ppm; FT-IR (neat): ν = 3092, 2947, 2857, 1585, 1455, 1329, 1223, 1125, 1111, 931, 760, 701 cm^{-1}; HRMS (ESI), *m*/*z*: $[M + Na]^+$ calcd for $C_{18}H_{20}N_2NaO_2^+$ 319.1417; found 319.1427.

(*Z*)-*N*-Benzyl-1-(pyridin-3-yl)methanimine oxide (**4j**)

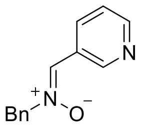

Yield: 98%; white solid; m.p. 95–96 °C; R_f =0.12 (*n*-hexane/EtOAc 1:4 *v/v*). All spectroscopic data were in accordance with those reported in the literature [**4**].

(*Z*)-*N*-Benzyl-1-mesitylmethanimine oxide (**4k**)

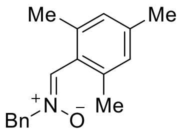

Yield: 85%; white solid; m.p. 144–145 °C; R_f = 0.24 (*n*-hexane/EtOAc 1:1 *v/v*); ^{1}H NMR (500 MHz, $CDCl_3$): δ = 7.62 (s, 1H), 7.54 – 7.50 (m, 2H), 7.45 – 7.34 (m, 3H), 6.83 (s, 2H), 5.12 (s, 2H), 2.25 (s, 3H), 2.16 (s, 6H) ppm; ^{13}C NMR (125 MHz, $CDCl_3$): δ = 139.5, 137.5, 136.4, 133.2, 129.3, 129.0, 128.3, 125.2, 69.9, 21.1, 19.7 ppm; FT-IR (neat): ν = 3041, 2971, 2914, 1567, 1456, 1192, 1163, 951, 696 cm^{-1}; HRMS (ESI), *m*/*z*: $[M + Na]^+$ calcd for $C_{17}H_{19}NNaO^+$ 276.1359; found 276.1360.

---

3 Poulsen, P. H.; Vergura, S.; Monleón, A.; Jørgensen, D. K. B.; Jørgensen, K. A. Controlling Asymmetric Remote and Cascade 1,3-Dipolar Cycloaddition Reactions by Organocatalysis. *J. Am. Chem. Soc.* **2016**, *138*, 6412−6415.
4 Delso. I.; Terejo, T.; ^{1}H–^{15}N HMBC as a Valuable Tool for the Identification and Characterization of Nitrones. *Tetrahedron Lett.* **2007**, *48*, 4101–4104.

(*Z*)-*N*-Benzyl-1-(2,6-dichlorophenyl)methanimine oxide (**4l**)

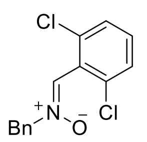

Yield: 99%; white solid; m.p. 164–165 °C; R_f = 0.34 (*n*-hexane/EtOAc 1:1 *v/v*); ^{1}H NMR (500 MHz, $CDCl_3$): δ = 7.55 – 7.51 (m, 3H), 7.46 – 7.38 (m, 2H), 7.31 (d, *J* = 8.0 Hz, 2H), 7.26 – 7.22 (m, 1H), 5.14 (s, 2H) ppm; ^{13}C NMR (125 MHz, $CDCl_3$): δ = 135.4, 132.5, 130.9, 130.6, 129.5, 129.1, 129.0, 128.1, 127.9, 70.3 ppm; FT-IR (neat): ν = 3064, 3028, 2995, 1556, 1430, 1165, 952, 808, 776, 699 cm^{-1}; HRMS (ESI), *m*/*z*: [M + Na]$^+$ calcd for $C_{14}H_{11}Cl_2NNaO^+$ 302.0110; found 302.0101.

(*Z*)-*N*-Benzyl-1-(perfluorophenyl)methanimine oxide (**4m**)

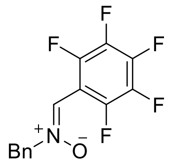

Yield: 97%; white solid; R_f = 0.28 (*n*-hexane/EtOAc 3:1 *v/v*). All spectroscopic data were in accordance with those reported in the literature [**5**].

(*Z*)-*N*-Benzyloctan-1-imine oxide (**4n**)

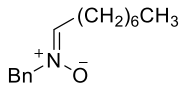

Yield: 93%; white solid; R_f =0.11 (*n*-hexane/EtOAc 1:2 *v/v*). All spectroscopic data were in accordance with those reported in the literature [**6**].

2. Data for compounds **5**

Benzaldehyde oxime (**5a**)

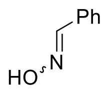

Yield: 99%; white solid; R_f = 0.4 (*n*-hexane/EtOAc 3:1 *v/v*). All spectroscopic data were in accordance with those reported in the literature [**7**].

4-Methylbenzaldehyde oxime (**5b**)

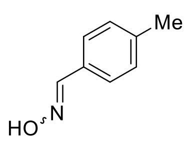

Yield: 98%; R_f = 0.37 (*n*-hexane/EtOAc 5:1 *v/v*). All spectroscopic data were in accordance with those reported in the literature [**8**].

5 Chakraborty, B.; Chettri, E. Synthesis of Some Novel Class of Regioselective Spiro Isoxazolidine Derivatives via 1,3-Dipolar Cycloaddition Reaction of *N*-Benzyl-*C*-fluorosubstituted Phenyl Nitrones in Ionic Liquid. *J. Heterocyclic Chem*. **2018**, *55*, 1157−1165.

6 Katahara, S.; Kobayashi, S.; Fujita, K.; Matsumoto, T.; Sato, T.; Chida, N. An Iridium-Catalyzed Reductive Approach to Nitrones from *N*-Hydroxyamides. *J. Am. Chem. Soc*. **2016**, *138*, 5246−5249.

7 Schierle, S.; Neumann, S.; Heitel, P.; Willems, S.; Kaiser, A.; Pollinger, J.; Merk, D.Design and Structural Optimization of Dual FXR/PPAR*δ* Activators. *J. Med. Chem*. **2020**, *63*, 8369−8379.

8 Di Nunno, L.; Vitale, P.; Scilimati, A.; Simonea, L.; Capitelli, F. Stereoselective Dimerization of 3-Arylisoxazoles to Cage-Shaped Bis-b-lactams *syn* 2,6-Diaryl-3,7-diazatricyclo[4.2.0.02,5]-octan-4,8-diones Induced by Hindered Lithium Amides. *Tetrahedron* **2007**, *63*, 12388−12395.

4-Methoxybenzaldehyde oxime (**5c**)

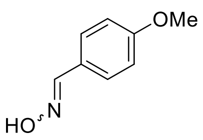

Yield: 96%; white solid; R_f = 0.32 (*n*-hexane/EtOAc 2:1 *v/v*). All spectroscopic data were in accordance with those reported in the literature [**8**].

4-(Trifluoromethyl)benzaldehyde oxime (**5d**)

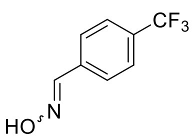

Yield: 98%; white solid; R_f = 0.42 (*n*-hexane/EtOAc 3:1 *v/v*). All spectroscopic data were in accordance with those reported in the literature [**8**].

4-Fluorobenzaldehyde oxime (**5e**)

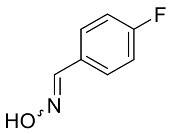

Yield: 99%; white solid; R_f = 0.41 (*n*-hexane/EtOAc 4:1 *v/v*). All spectroscopic data were in accordance with those reported in the literature [**8**].

4-(*t*-Butyl)benzaldehyde oxime (**5f**)

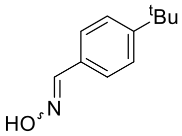

Yield: 93%; white solid; R_f = 0.38 (*n*-hexane/EtOAc 4:1 *v/v*). All spectroscopic data were in accordance with those reported in the literature [**9**].

2-Bromobenzaldehyde oxime (**5g**)

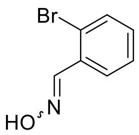

Yield: 97%; white solid; R_f = 0.38 (*n*-hexane/EtOAc 4:1 *v/v*). All spectroscopic data were in accordance with those reported in the literature [**10**].

2-(Trifluoromethyl)benzaldehyde oxime (**5h**)

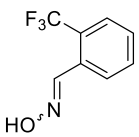

Yield: 96%; white solid; R_f = 0.40 (*n*-hexane/EtOAc 3:1 *v/v*). All spectroscopic data were in accordance with those reported in the literature [**7**].

---

9 Tambara, K.; Dan Pantos, G. Conversion of Aldoximes into Nitriles and Amides Under Mild Conditions. *Org. Biomol. Chem.*, **2013**, *11*, 2466−2472.
10 Steiger, S. A.; Li, C.; Backos, D. S.; Reigan, P.; Natale, N.R. Dimeric Isoxazolyl-1,4-Dihydropyridines Have Enhanced Binding at the Multi-Drug Resistance Transporter. *Bioorg. Med. Chem.* **2017**, *25*, 3223−3234.

3-Nitrobenzaldehyde oxime (**5i**)

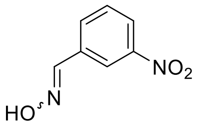

Yield: 99%; white solid; R_f = 0.23 (*n*-hexane/EtOAc 1:1 *v/v*). All spectroscopic data were in accordance with those reported in the literature [**11**].

2,4,6-Trimethylbenzaldehyde oxime (**5j**)

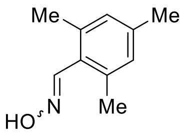

Yield: 97%; white solid; R_f = 0.43 (*n*-hexane/EtOAc 5:1 *v/v*). All spectroscopic data were in accordance with those reported in the literature [**12**].

2,3,4,5,6-Pentafluorobenzaldehyde oxime (**5k**)

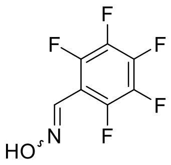

Yield: 90%; white solid; R_f = 0.52 (*n*-hexane/EtOAc 3:1 *v/v*). All spectroscopic data were in accordance with those reported in the literature [**13**].

2,6-Dichlorobenzaldehyde oxime (**5l**)

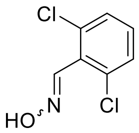

Yield: 99%; white solid; R_f = 0.54 (*n*-hexane/EtOAc 4:1 *v/v*). All spectroscopic data were in accordance with those reported in the literature [**7**].

5-Chlorofuran-2-carbaldehyde oxime (**5m**)

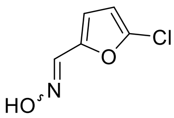

Yield: 88%; white solid; R_f = 0.43 (*n*-hexane/EtOAc 4:1 *v/v*). All spectroscopic data were in accordance with those reported in the literature [**14**].

11 Yu, J.; Lu, M. Metal-Free: A Novel and Efficient Aerobic Oxidation of Primary Amines to Oximes Using *N,N′,N″*-Trihydroxyisocyanuric Acid and Acetaldoxime as Catalysts in Water. *SYNLETT* **2014**, *25*, 1873−1878.

12 McIntosh, M. L.; Naffziger, M. R.; Ashburn, B. O.; Zakharov, L. N.; Carter, R. G. Highly Regioselective Nitrile Oxide Dipolar Cycloadditions with *ortho*-Nitrophenyl Alkynes. *Org. Biomol. Chem.* **2012**, *10*, 9204−9213.

13 Jawalekar, A. M.; Reubsaet, E.; Rutjes F. P. J. T.; van Delft, F. L. Synthesis of Isoxazoles by Hypervalent Iodine-Induced Cycloaddition of Nitrile Oxides to Alkynes. *Chem. Commun.* **2011**, *47*, 3198−3200.

14 Kanemasa, S.; Matsuda, H.; Kamimurac, A.; Kakinami, T. Synthesis of Hydroximoyl Chlorides from Aldoximes and Benzyltrimethylammonium Tetrachloroiodate (BTMA ICl_4). *Tetrahedron* **2000**, *56*, 1057−1064.

Thiophene-2-carbaldehyde oxime (**5n**)

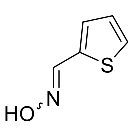

Yield: 83%; white solid; R_f = 0.43 (*n*-hexane/EtOAc 4:1 *v/v*). All spectroscopic data were in accordance with those reported in the literature [**11**].

Octanal oxime (**5o**)

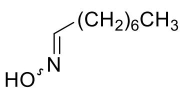

Yield: 94%; white solid; R_f = 0.39 (*n*-hexane/EtOAc 2:1 *v/v*). All spectroscopic data were in accordance with those reported in the literature [**15**].

3. Data for compounds **6**, **8** and **7**

(3*R*,3a*R*,8b*S*)-2-Benzyl-3-phenyl-2,3,3a,8b-tetrahydro-4*H*-indeno[2,1-*d*]isoxazol-4-one (**6a**)

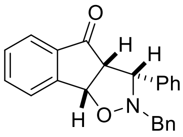

Yield: 50%; white solid; m.p. 159–160 °C; R_f = 0.38 (*n*-hexane/EtOAc 3:1 *v/v*); ^{1}H NMR (500 MHz, DMSO-d_6): δ = 7.79 (tt, *J* = 7.9, 1.9 Hz, 1H), 7.70 (d, *J* = 7.6 Hz, 1H), 7.59 – 7.52 (m, 2H), 7.28 – 7.15 (m, 6H), 7.09 (t, *J* = 6.1 Hz, 4H), 5.75 (d, *J* = 6.3 Hz, 1H), 4.20 (d, *J* = 9.3 Hz, 1H), 3.85 – 3.78 (m, 2H), 3.71 (d, *J* = 15.3 Hz, 1H) ppm; ^{13}C NMR (125 MHz, DMSO-d_6): δ = 201.7, 153.2, 138.7, 137.9, 136.2, 135.8, 130.0, 128.6, 128.6, 128.31, 128.26, 128.1, 127.1, 126.8, 122.1, 78.4, 73.5, 60.1, 58.5 ppm; FT-IR (neat): ν = 3058, 3024, 2855, 1715, 1600, 1495, 1455, 1264, 775, 756, 733, 698 cm^{-1}; HRMS (ESI), *m*/*z*: [M + Na]$^+$ calcd for $C_{23}H_{19}NNaO_2^+$ 364.1308; found 364.1300.

(3*S*,3a*R*,8b*S*)-2-Benzyl-3-phenyl-2,3,3a,8b-tetrahydro-4*H*-indeno[2,1-*d*]isoxazol-4-one (**7a**)

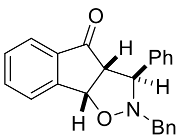

Yield: 40%; white solid; m.p. 190–191 °C; R_f = 0.50 (*n*-hexane/EtOAc 3:1 *v/v*); ^{1}H NMR (500 MHz, DMSO-d_6, 60 °C): δ = 7.78 (t, *J* = 7.4 Hz, 1H), 7.72 (t, *J* = 7.9 Hz, 2H), 7.63 (t, *J* = 7.3 Hz, 1H), 7.53 (d, *J* = 7.4 Hz, 2H), 7.44 (t, *J* = 7.5 Hz, 2H), 7.36 (t, *J* = 7.4 Hz, 1H), 7.25 – 7.16 (m, 3H), 7.13 (d, *J* = 6.8 Hz, 2H), 5.81 (d, *J* = 6.4 Hz, 1H), 4.07 (d, *J* = 5.7 Hz, 1H), 3.81 (t, *J* = 6.1 Hz, 1H), 3.66 (dd, *J* = 14.9, 5.2 Hz, 2H) ppm; ^{13}C NMR (125 MHz, DMSO-d_6, 60 °C): δ = 203.3, 150.8, 138.6, 137.8, 136.5, 135.9, 130.5, 128.8, 128.4, 128.3, 128.2, 128.1, 127.6, 127.0, 123.6, 78.7, 72.2, 63.5, 58.7 ppm; FT-IR (neat): ν = 3022, 2915, 2856, 1714, 1604, 1495, 1456, 1348, 1231, 754, 731, 694 cm^{-1}; HRMS (ESI), *m*/*z*: [M + Na]$^+$ calcd for $C_{23}H_{19}NNaO_2^+$ 364.1308; found 364.1302.

15 Suzuki, K.; Watanabe, T.; Murahashi, S.-I. Oxidation of Primary Amines to Oximes with Molecular Oxygen using 1,1-Diphenyl-2-picrylhydrazyl and WO_3/Al_2O_3 as Catalysts. *J. Org. Chem.* **2013**, *78*, 2301−2310.

(3*R*,3a*R*,8b*S*)-2-Benzyl-3-(p-tolyl)-2,3,3a,8b-tetrahydro-4*H*-indeno[2,1-*d*]isoxazol-4-one (**6b**)

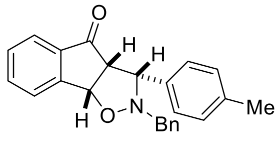

Yield: 40%; white solid; m.p. 145.5–146.5 °C; R_f = 0.70 (*n*-hexane/EtOAc 4:1 *v/v*); ^{1}H NMR (500 MHz, DMSO-d_6): δ = 7.79 (tt, *J* = 8.1, 5.8, 2.5 Hz, 1H), 7.70 (d, *J* = 7.6 Hz, 1H), 7.59 – 7.54 (m, 2H), 7.23 – 7.15 (m, 3H), 7.10 – 7.06 (m, 4H), 6.96 (d, *J* = 8.0 Hz, 2H), 5.73 (d, *J* = 6.3 Hz, 1H), 4.14 (d, *J* = 9.4 Hz, 1H), 3.80 – 3.76 (m, 2H), 3.67 (d, *J* = 15.3 Hz, 1H), 2.26 (s, 3H) ppm; ^{13}C NMR (125 MHz, DMSO-d_6): δ = 201.9, 153.4, 138.8, 138.0, 137.3, 135.9, 133.1, 130.1, 129.3, 128.6, 128.4, 128.3, 127.2, 126.9, 122.2, 78.4, 73.4, 60.1, 58.4, 21.2 ppm; FT-IR (neat): ν = 3024, 2921, 2852, 1712, 1599, 1346, 1213, 1079, 1042, 814, 773, 734, 695 cm^{-1}; HRMS (ESI), *m/z*: [M + Na]$^+$ calcd for $C_{24}H_{21}NNaO_2^+$ 378.1465; found 378.1472.

(3*S*,3a*R*,8b*S*)-2-Benzyl-3-(p-tolyl)-2,3,3a,8b-tetrahydro-4*H*-indeno[2,1-*d*]isoxazol-4-one (**7b**)

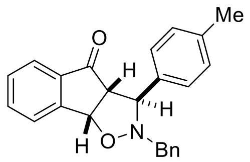

Yield: 37%; white solid; m.p. 159–160 °C; R_f = 0.58 (*n*-hexane/EtOAc 4:1 *v/v*); ^{1}H NMR (500 MHz, DMSO-d_6, 60 °C): δ = 7.78 (t, *J* = 7.4 Hz, 1H), 7.72 (t, *J* = 6.3 Hz, 2H), 7.63 (t, *J* = 7.5 Hz, 1H), 7.40 (d, *J* = 7.9 Hz, 2H), 7.26 – 7.17 (m, 5H), 7.12 (d, *J* = 7.2 Hz, 2H), 5.79 (d, *J* = 6.4 Hz, 1H), 4.01 (d, *J* = 5.8 Hz, 1H), 3.78 (t, *J* = 6.1 Hz, 1H), 3.66 (d, *J* = 14.9 Hz, 1H), 3.61 (d, *J* = 14.7 Hz, 1H), 2.34 (s, 3H) ppm; ^{13}C NMR (125 MHz, DMSO-d_6, 60 °C): δ = 203.3, 150.8, 137.9, 137.5, 136.5, 135.8, 135.4, 130.5, 129.4, 128.4, 128.3, 128.1, 127.6, 127.0, 123.6, 78.6, 72.1, 63.4, 58.5, 20.9 ppm; FT-IR (neat): ν = 3032, 2950, 2843, 1714, 1605, 1350, 1278, 1056, 763, 701 cm^{-1}; HRMS (ESI), *m/z*: [M + Na]$^+$ calcd for $C_{24}H_{21}NNaO_2^+$ 378.1465; found 378.1470.

(3*R*,3a*R*,8b*S*)-2-Benzyl-3-(4-methoxyphenyl)-2,3,3a,8b-tetrahydro-4*H*-indeno[2,1-*d*]isoxazol-4-one (**6c**)

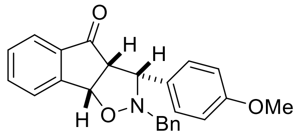

Yield: 47%; white solid; m.p. 130–131 °C; R_f = 0.43 (*n*-hexane/EtOAc 3:1 *v/v*); ^{1}H NMR (500 MHz, DMSO-d_6): δ = 7.82 – 7.77 (m, 1H), 7.71 (d, *J* = 7.6 Hz, 1H), 7.56 (d, *J* = 4.5 Hz, 2H), 7.24 – 7.15 (m, 3H), 7.09 (d, *J* = 8.0 Hz, 2H), 7.00 (d, *J* = 8.7 Hz, 2H), 6.83 (d, *J* = 8.8 Hz, 2H), 5.73 (d, *J* = 6.2 Hz, 1H), 4.13 (d, *J* = 9.5 Hz, 1H), 3.81 – 3.74 (m, 2H), 3.72 (s, 3H), 3.66 (d, *J* = 15.3 Hz, 1H) ppm; ^{13}C NMR (125 MHz, DMSO-d_6): δ = 202.0, 159.2, 153.4, 138.8, 138.0, 135.8, 130.1, 129.8, 128.4, 128.3, 127.8, 127.2, 126.9, 122.2, 114.1, 78.3, 73.2, 60.0, 58.4, 55.4 ppm; FT-IR (neat): ν = 3024, 3007, 2844, 1711, 1613 ,1598, 1514, 1254 ,1032, 836, 774, 735 cm^{-1}; HRMS (ESI), *m/z*: [M + K]$^+$ calcd for $C_{24}H_{21}KNO_3^+$ 433.1045; found 433.1056.

(3*S*,3a*R*,8b*S*)-2-Benzyl-3-(4-methoxyphenyl)-2,3,3a,8b-tetrahydro-4*H*-indeno[2,1-*d*]isoxazol-4-one (**7c**)

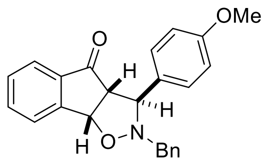

Yield: 24%; white solid; m.p. 127–128 °C; R_f = 0.60 (*n*-hexane/EtOAc 3:1 *v/v*); ^{1}H NMR (500 MHz, DMSO-d_6, 60 °C): δ = 7.78 (t, *J* = 7.4 Hz, 1H), 7.72 (t, *J* = 6.6 Hz, 2H), 7.62 (t, *J* = 7.7 Hz, 1H), 7.43 (d, *J* = 8.6 Hz, 2H), 7.25 – 7.16 (m, 3H), 7.13 (d, *J* = 7.3 Hz, 2H), 6.99 (d, *J* = 8.7 Hz, 2H), 5.79 (d, *J* = 6.4 Hz, 1H), 3.99 (br s, 1H), 3.79 (s, 3H), 3.77 (t, *J* = 6.1 Hz, 1H), 3.63 (dd, *J* = 14.7, 5.3 Hz, 2H) ppm; ^{13}C NMR (125 MHz, DMSO-d_6, 60 °C): δ = 203.3, 159.4, 150.9, 137.9, 136.5, 135.8, 130.5, 130.22, 129.7, 128.3, 128.1, 127.6, 127.0, 123.6, 114.4, 78.6, 71.8, 63.4, 58.4, 55.4 ppm; FT-IR (neat): ν = 3032, 2909, 2835, 1718, 1613, 1514, 1248, 1178, 1035, 829, 770, 731 cm^{-1}; HRMS (ESI), *m/z*: [M + Na]$^+$ calcd for $C_{24}H_{21}NNaO_3^+$ 394.1414; found 394.1422.

(3*R*,3a*R*,8b*S*)-2-Benzyl-3-(4-hydroxyphenyl)-2,3,3a,8b-tetrahydro-4*H*-indeno[2,1-*d*]isoxazol-4-one (**6d**)

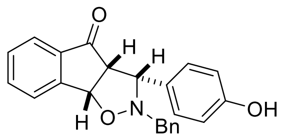

Yield: 45%; white solid; m.p. 202–204 °C; R_f = 0.28 (*n*-hexane/EtOAc 1:1 *v/v*); ^{1}H NMR (500 MHz, DMSO-d_6): δ = 9.36 (br s, 1H), 7.81 – 7.76 (m, 1H), 7.70 (d, *J* = 7.6 Hz, 1H), 7.59 – 7.54 (m, 2H), 7.23 – 7.14 (m, 3H), 7.08 (d, *J* = 6.9 Hz, 2H), 6.86 (d, *J* = 8.5 Hz, 2H), 6.64 (d, *J* = 8.6 Hz, 2H), 5.71 (d, *J* = 6.3 Hz, 1H), 4.07 (d, *J* = 9.3 Hz, 1H), 3.78 (d, *J* = 15.4 Hz, 1H), 3.72 (dd, *J* = 9.3, 6.3 Hz, 1H), 3.64 (d, *J* = 15.4 Hz, 1H) ppm; ^{13}C NMR (125 MHz, DMSO-d_6): δ = 202.1, 157.4, 153.5, 138.9, 138.1, 135.8, 130.1, 129.8, 128.4, 128.3, 127.1, 126.9, 126.0, 122.1, 115.5, 78.3, 73.5, 60.0, 58.3 ppm; FT-IR (neat): ν = 3284, 3024, 2847, 1694, 1600, 1518, 1454, 1274, 1263, 834, 768, 741, 694 cm^{-1}; HRMS (ESI), *m/z*: [M + Na]$^+$ calcd for $C_{23}H_{19}NNaO_3^+$ 380.1257; found 380.1260.

(3*S*,3a*R*,8b*S*)-2-Benzyl-3-(4-hydroxyphenyl)-2,3,3a,8b-tetrahydro-4*H*-indeno[2,1-*d*]isoxazol-4-one (**7d**)

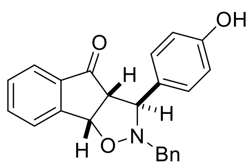

Yield: 26%; white solid; m.p. 189–190 °C; R_f = 0.40 (*n*-hexane/EtOAc 1:1 *v/v*); ^{1}H NMR (500 MHz, DMSO-d_6, 60 °C): δ = 9.33 (br s, 1H), 7.77 (t, *J* = 7.4 Hz, 1H), 7.71 (t, *J* = 7.1 Hz, 2H), 7.62 (t, *J* = 7.5 Hz, 1H), 7.31 (d, *J* = 8.4 Hz, 2H), 7.25 – 7.11 (m, 5H), 6.82 (d, *J* = 8.4 Hz, 2H), 5.77 (d, *J* = 6.4 Hz, 1H), 3.92 (br s, 1H), 3.75 (t, *J* = 6.1 Hz, 1H), 3.66 (d, *J* = 14.8 Hz, 1H), 3.58 (d, *J* = 14.7 Hz, 1H) ppm; ^{13}C NMR (125 MHz, DMSO-d_6, 60 °C): δ = 203.4, 157.5, 150.9, 138.0, 136.5, 135.8, 130.4, 129.7, 128.4, 128.3, 128.1, 127.5, 126.9, 123.6, 115.7, 78.5, 72.0, 63.3, 58.3 ppm; FT-IR (neat): ν = 3482, 3024, 2919, 2849, 1699, 1602, 1518, 1348, 1277, 1233, 843, 833, 733, 694 cm^{-1}; HRMS (ESI), *m/z*: [M + Na]$^+$ calcd for $C_{23}H_{19}NNaO_3^+$ 380.1257; found 380.1251.

(3*R*,3a*R*,8b*S*)-2-Benzyl-3-(4-morpholinophenyl)-2,3,3a,8b-tetrahydro-4*H*-indeno[2,1-*d*]isoxazol-4-one (**6e**)

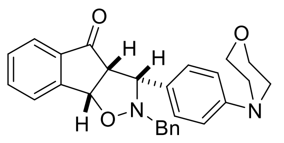

Yield: 43%; white solid; m.p. 151–152 °C; R_f = 0.34 (*n*-hexane/EtOAc 2:1 *v/v*); ^{1}H NMR (500 MHz, DMSO-d_6): δ = 7.79 (ddd, *J* = 8.0, 5.6, 2.7 Hz, 1H), 7.70 (d, *J* = 7.6 Hz, 1H), 7.59 – 7.54 (m, 2H), 7.23 – 7.15 (m, 3H), 7.08 (d, *J* = 6.9 Hz, 2H), 6.93 (d, *J* = 8.6 Hz, 2H), 6.82 (d, *J* = 8.8 Hz, 2H), 5.71 (d, *J* = 6.3 Hz, 1H), 4.08 (d, *J* = 9.3 Hz, 1H), 3.77 (d, *J* = 15.4 Hz, 1H), 3.74 – 3.69 (m, 5H), 3.64 (d, *J* = 15.4 Hz, 1H), 3.11 – 3.04 (m, 4H) ppm; ^{13}C NMR (125 MHz, DMSO-d_6): δ = 202.0, 153.5, 150.8, 138.8, 138.1, 135.8, 130.0, 129.4, 128.4, 128.2, 127.1, 126.9, 126.0, 122.1, 115.0, 78.3, 73.5, 66.6, 60.0, 58.3, 48.5 ppm; FT-IR (neat): ν = 3035, 2950, 2838, 1715, 1612, 1516, 1264, 1234, 1218, 1120, 927, 770, 735 cm^{-1}; HRMS (ESI), *m*/*z*: [M + Na]$^+$ calcd for $C_{27}H_{26}N_2NaO_3^+$ 449.1836; found 449.1832.

(3*S*,3a*R*,8b*S*)-2-Benzyl-3-(4-morpholinophenyl)-2,3,3a,8b-tetrahydro-4*H*-indeno[2,1-*d*]isoxazol-4-one (**7e**)

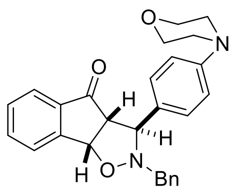

Yield: 26%; white solid; m.p. 219–220 °C; R_f = 0.53 (*n*-hexane/EtOAc 2:1 *v/v*); ^{1}H NMR (500 MHz, DMSO-d_6, 60 °C): δ = 7.78 (t, *J* = 7.4 Hz, 1H), 7.72 (t, *J* = 6.3 Hz, 2H), 7.62 (t, *J* = 7.5 Hz, 1H), 7.37 (d, *J* = 8.6 Hz, 2H), 7.26 – 7.16 (m, 3H), 7.13 (d, *J* = 7.2 Hz, 2H), 6.99 (d, *J* = 8.7 Hz, 2H), 5.78 (d, *J* = 6.4 Hz, 1H), 3.93 (br s, 1H), 3.78 – 3.74 (m, 4H), 3.67 (d, *J* = 14.9 Hz, 1H), 3.59 (d, *J* = 14.8 Hz, 1H), 3.15 (d, *J* = 5.1 Hz, 4H) ppm; ^{13}C NMR (125 MHz, DMSO-d_6, 60 °C): δ = 203.3, 151.0, 150.9, 138.0, 136.5, 135.8, 130.4, 129.2, 128.4, 128.3, 128.1, 127.5, 126.9, 123.6, 115.2, 78.5, 72.0, 66.4, 63.3, 58.4, 48.6 ppm; FT-IR (neat): ν = 3011, 2966, 2837, 1721, 1610, 1521, 1350, 1234, 1122, 926, 821, 742, 688 cm^{-1}; HRMS (ESI), *m*/*z*: [M + H]$^+$ calcd for $C_{27}H_{27}N_2O_3^+$ 427.2016; found 427.2011.

(3*R*,3a*R*,8b*S*)-2-Benzyl-3-(4-(trifluoromethyl)phenyl)-2,3,3a,8b-tetrahydro-4*H*-indeno[2,1-*d*]isoxazol-4-one (**6f**)

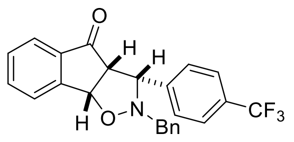

Yield: 54%; white solid; m.p. 145–146 °C; R_f = 0.47 (*n*-hexane/EtOAc 3:1 *v/v*); ^{1}H NMR (500 MHz, DMSO-d_6): δ = 7.80 (ddd, *J* = 7.7, 6.2, 2.1 Hz, 1H), 7.72 (d, *J* = 7.6 Hz, 1H), 7.64 (d, *J* = 8.2 Hz, 2H), 7.58 – 7.53 (m, 2H), 7.33 (d, *J* = 8.1 Hz, 2H), 7.24 – 7.16 (m, 3H), 7.13 – 7.10 (m, 2H), 5.80 (d, *J* = 6.3 Hz, 1H), 4.36 (d, *J* = 9.3 Hz, 1H), 3.92 (dd, *J* = 9.3, 6.4 Hz, 1H), 3.84 (d, *J* = 15.2 Hz, 1H), 3.75 (d, *J* = 15.2 Hz, 1H) ppm; ^{13}C NMR (125 MHz, DMSO-d_6): δ = 201.5, 153.0, 141.3, 138.5, 137.6, 135.9, 130.1, 129.4, 128.8 (q, *J* = 31.6 Hz), 128.5, 128.3, 127.2, 126.9, 125.4 (q, *J* = 3.9 Hz), 124.7 (q, *J* = 272.1 Hz), 122.3, 78.6, 72.7, 60.2, 58.9 ppm; ^{19}F NMR (470 MHz, CDCl3): δ = –62.62 (s, 3F) ppm; FT-IR (neat): ν = 3034, 2953, 2922, 1721, 1602, 1329, 1163, 1122, 1068, 833, 752, 699, 590, 452 cm^{-1}; HRMS (ESI), *m*/*z*: [M + H]$^+$ calcd for $C_{24}H_{19}F_3NO_2^+$ 410.1362; found 410.1356.

(3*S*,3a*R*,8b*S*)-2-Benzyl-3-(4-(trifluoromethyl)phenyl)-2,3,3a,8b-tetrahydro-4*H*-indeno[2,1-*d*]isoxazol-4-one (**7f**)

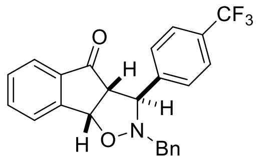

Yield: 24%; white solid; m.p. 170–171 °C; R_f = 0.66 (*n*-hexane/EtOAc 3:1 *v/v*); ^{1}H NMR (500 MHz, DMSO-d_6, 60 °C): δ = 7.83 – 7.72 (m, 7H), 7.65 (t, *J* = 7.4 Hz, 1H), 7.25 – 7.17 (m, 3H), 7.13 (d, *J* = 7.1 Hz, 2H), 5.83 (d, *J* = 6.4 Hz, 1H), 4.24 (d, *J* = 5.4 Hz, 1H), 3.85 (t, *J* = 5.9 Hz, 1H), 3.71 (d, *J* = 14.6 Hz, 1H), 3.67 (d, *J* = 14.8 Hz, 1H) ppm; ^{13}C NMR (125 MHz, DMSO-d_6, 60 °C): δ = 203.0, 150.8, 143.6, 137.5, 136.4, 136.0, 130.6, 129.1, 128.9 (q, *J* = 31.9 Hz), 128.4, 128.2, 127.6, 127.1, 125.7 (q, *J* = 3.9 Hz), 124.4 (q, *J* = 272.2 Hz), 123.7, 78.9, 71.4, 63.6, 59.1 ppm; ^{19}F NMR (470 MHz, CDCl3): δ = –62.61 (s, 3F) ppm; FT-IR (neat): ν = 3035, 2955, 2850, 1715, 1607, 1331, 1159, 1111, 1068, 855, 762, 703, 463 cm^{-1}; HRMS (ESI), *m/z*: [M + Na]$^+$ calcd for $C_{24}H_{18}F_3NNaO_2^+$ 432.1182; found 432.1189.

(3*R*,3a*R*,8b*S*)-2-Benzyl-3-(2-(trifluoromethyl)phenyl)-2,3,3a,8b-tetrahydro-4*H*-indeno[2,1-*d*]isoxazol-4-one (**6g**)

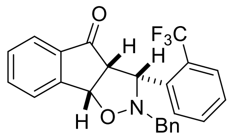

Yield: 31%; white solid; m.p. 155–157 °C; R_f = 0.54 (*n*-hexane/EtOAc 4:1 *v/v*); ^{1}H NMR (500 MHz, DMSO-d_6): δ = 7.81 (td, *J* = 7.5, 7.0, 1.3 Hz, 1H), 7.78 – 7.72 (m, 2H), 7.60 – 7.52 (m, 2H), 7.48 – 7.43 (m, 2H), 7.23 – 7.09 (m, 6H), 5.82 (d, *J* = 6.3 Hz, 1H), 4.40 (d, *J* = 9.2 Hz, 1H), 3.84 – 3.80 (m, 2H), 3.76 (d, *J* = 15.0 Hz, 1H) ppm; ^{13}C NMR (125 MHz, DMSO-d_6): δ = 201.0, 153.0, 138.5, 137.2, 135.7, 134.6 (d, *J* = 1.5 Hz), 132.5, 130.0, 128.9, 128.4, 128.11, 128.09, 128.0 (q, *J* = 29.6 Hz), 127.0, 126.7, 126.0 (q, *J* = 5.7 Hz), 124.6 (q, *J* = 274.0 Hz), 122.0, 78.4, 69.1, 60.0, 58.6 ppm; ^{19}F NMR (470 MHz, CDCl3): δ = –58.78 (s, 3F) ppm; FT-IR (neat): ν = 3021, 2945, 2883, 1716, 1653, 1455, 1313, 1261, 1165, 1103, 1033, 785, 748, 701, 660 cm^{-1}; HRMS (ESI), *m/z*: [M + Na]$^+$ calcd for $C_{24}H_{18}F_3NNaO_2^+$ 432.1182; found 432.1181.

(3*S*,3a*R*,8b*S*)-2-Benzyl-3-(2-(trifluoromethyl)phenyl)-2,3,3a,8b-tetrahydro-4*H*-indeno[2,1-*d*]isoxazol-4-one (**7g**)

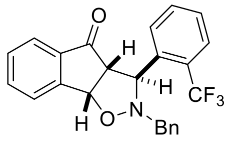

Yield: 24%; white solid; m.p. 152–153 °C; R_f = 0.43 (*n*-hexane/EtOAc 4:1 *v/v*); ^{1}H NMR (500 MHz, DMSO-d_6, 60 °C): δ = 8.07 (d, *J* = 7.9 Hz, 1H), 7.83 – 7.73 (m, 5H), 7.65 (t, *J* = 7.5 Hz, 1H), 7.58 (t, *J* = 7.6 Hz, 1H), 7.24 – 7.16 (m, 3H), 7.07 (d, *J* = 6.8 Hz, 2H), 5.90 (d, *J* = 6.5 Hz, 1H), 4.48 (d, *J* = 3.8 Hz, 1H), 3.92 (t, *J* = 5.8 Hz, 1H), 3.65 (d, *J* = 14.3 Hz, 1H), 3.61 (d, *J* = 14.4 Hz, 1H) ppm; ^{13}C NMR (125 MHz, DMSO-d_6, 60 °C): δ = 202.1, 151.1, 138.1 (d, *J* = 1.8 Hz), 137.7, 136.4, 136.3, 133.4, 130.9, 130.7, 128.9, 128.6, 128.4, 127.9 (q, *J* = 29.6 Hz), 127.8, 127.4, 126.2 (q, *J* = 5.9 Hz), 124.6 (q, *J* = 274.7 Hz), 124.0, 79.7, 67.7, 64.9, 59.7 ppm; ^{19}F NMR (470 MHz, CDCl3): δ = –56.83 (s, 3F) ppm; FT-IR (neat): ν = 3021, 2925, 2874, 1716, 1647, 1456, 1312, 1277, 1162, 1121, 1034, 769, 694, 666 cm^{-1}; HRMS (ESI), *m/z*: [M + Na]$^+$ calcd for $C_{24}H_{18}F_3NNaO_2^+$ 432.1182; found 432.1186.

(3*R*,3a*S*,8a*R*)-2-Benzyl-3-(2-(trifluoromethyl)phenyl)-2,3,3a,8a-tetrahydro-8*H*-indeno[1,2-*d*]isoxazol-8-one (**8g**)

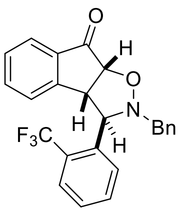

Yield: 16%; white solid; m.p. 165–166 °C; R_f = 0.22 (*n*-hexane/DCM 1:1 *v/v*); ^{1}H NMR (500 MHz, DMSO-d_6, 60 °C): δ = 8.07 (d, *J* = 7.9 Hz, 1H), 7.86 – 7.74 (m, 4H), 7.63 – 7.56 (m, 2H), 7.44 (d, *J* = 7.7 Hz, 1H), 7.25 – 7.17 (m, 3H), 7.09 (d, *J* = 7.1 Hz, 2H), 5.02 (d, *J* = 6.8 Hz, 1H), 4.51 (dd, *J* = 6.8, 4.3 Hz, 1H), 4.40 (d, *J* = 2.8 Hz, 1H), 3.65 (d, *J* = 14.4 Hz, 1H), 3.57 (d, *J* = 14.4 Hz, 1H) ppm; ^{13}C NMR (125 MHz, DMSO-d_6, 60 °C): δ = 201.2, 152.9, 137.9, 137.3, 136.6, 135.1, 133.4, 130.4, 129.4, 128.7, 128.3, 128.2, 127.4 (q, *J* = 29.3 Hz), 127.2, 126.8, 125.9 (q, *J* = 5.8 Hz), 124.5 (q, *J* = 274.4 Hz), 124.2, 81.8, 70.2, 59.3, 57.5 ppm; ^{19}F NMR (470 MHz, CDCl3): δ = –56.71 (s, 3F) ppm; FT-IR (neat): ν = 2934, 1717, 1647, 1541,1465, 1311, 1161, 1121, 825, 779, 697, 668 cm^{-1}; HRMS (ESI), *m*/*z*: [M + K]$^+$ calcd for $C_{24}H_{18}F_3KNO_2^+$ 448.0921; found 448.0930.

(3*R*,3a*R*,8b*S*)-2-Benzyl-3-(2-bromophenyl)-2,3,3a,8b-tetrahydro-4*H*-indeno[2,1-*d*]isoxazol-4-one (**6h**)

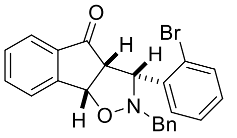

Yield: 28%; white solid; m.p. 139–140 °C; R_f = 0.54 (*n*-hexane/THF 20:1 *v/v*); ^{1}H NMR (500 MHz, DMSO-d_6): δ = 7.79 (t, *J* = 7.4 Hz, 1H), 7.73 (d, *J* = 7.6 Hz, 1H), 7.68 – 7.64 (m, 1H), 7.55 (t, *J* = 7.3 Hz, 1H), 7.49 (d, *J* = 7.5 Hz, 1H), 7.25 – 7.17 (m, 5H), 7.12 (d, *J* = 7.0 Hz, 2H), 6.91 – 6.88 (m, 1H), 5.81 (d, *J* = 6.4 Hz, 1H), 4.43 (d, *J* = 9.3 Hz, 1H), 3.96 – 3.90 (m, 2H), 3.78 (d, *J* = 15.2 Hz, 1H) ppm; ^{13}C NMR (125 MHz, DMSO-d_6): δ = 200.8, 152.9, 138.3, 137.1, 135.6, 135.4, 132.8, 129.9, 129.6, 128.7, 128.3, 128.1, 127.8, 127.0, 126.7, 124.2, 121.9, 78.1, 71.9, 58.5, 57.5 ppm; FT-IR (neat): ν = 3026, 2945, 2868, 1712, 1602, 1260, 1221, 1015, 766, 748, 697, 620 cm^{-1}; HRMS (ESI), *m*/*z*: [M + H]$^+$ calcd for $C_{23}H_{19}BrNO_2^+$ 420.0594; found 420.0596.

(3*S*,3a*R*,8b*S*)-2-Benzyl-3-(2-bromophenyl)-2,3,3a,8b-tetrahydro-4*H*-indeno[2,1-*d*]isoxazol-4-one (**7h**)

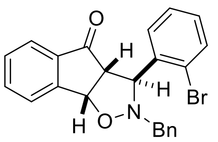

Yield: 20%; white solid; m.p. 148–150 °C; R_f = 0.52 (*n*-hexane/THF 20:1 *v/v*); ^{1}H NMR (500 MHz, DMSO-d_6, 60 °C): δ = 7.82 – 7.72 (m, 4H), 7.68 – 7.62 (m, 2H), 7.48 (t, *J* = 7.5 Hz, 1H), 7.29 (td, *J* = 7.7, 1.7 Hz, 1H), 7.23 – 7.15 (m, 3H), 7.06 (d, *J* = 6.9 Hz, 2H), 5.86 (d, *J* = 6.5 Hz, 1H), 4.70 (d, *J* = 4.6 Hz, 1H), 3.91 (dd, *J* = 6.5, 4.7 Hz, 1H), 3.71 (d, *J* = 14.2 Hz, 1H), 3.64 (d, *J* = 14.4 Hz, 1H). ppm; ^{13}C NMR (125 MHz, DMSO-d_6, 60 °C): δ = 202.7, 151.3, 137.7, 137.4, 136.3, 136.0, 133.1, 130.6, 130.3, 130.0, 128.4, 128.3, 128.1, 127.6, 127.1, 123.8, 123.7, 79.3, 70.5, 63.6, 59.4 ppm; FT-IR (neat): ν = 3028, 2919, 2870, 1715, 1600, 1472, 1347, 1276, 1233, 1022, 844, 752, 743, 698, 618 cm^{-1}; HRMS (ESI), *m*/*z*: [M + H]$^+$ calcd for $C_{23}H_{19}BrNO_2^+$ 420.0594; found 420.0596.

(3*R*,3a*S*,8a*R*)-2-Benzyl-3-(2-bromophenyl)-2,3,3a,8a-tetrahydro-8*H*-indeno[1,2-*d*]isoxazol-8-one (**8h**)

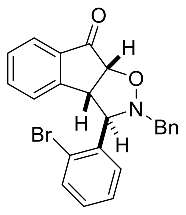

Yield: 23%; white solid; m.p. 123–124 °C; R_f = 0.30 (DCM); ^{1}H NMR (500 MHz, DMSO-d_6, 60 °C): δ = 7.84 – 7.78 (m, 2H), 7.77 – 7.71 (m, 2H), 7.69 (d, *J* = 8.0 Hz, 1H), 7.58 (t, *J* = 7.4 Hz, 1H), 7.51 (t, *J* = 7.5 Hz, 1H), 7.31 (td, *J* = 7.7, 1.7 Hz, 1H), 7.25 – 7.17 (m, 3H), 7.08 (d, *J* = 7.0 Hz, 2H), 4.92 (d, *J* = 6.8 Hz, 1H), 4.66 (d, *J* = 3.6 Hz, 1H), 4.46 (dd, *J* = 6.9, 3.7 Hz, 1H), 3.71 (d, *J* = 14.2 Hz, 1H), 3.62 (d, *J* = 14.2 Hz, 1H) ppm; ^{13}C NMR (125 MHz, DMSO-d_6, 60 °C): δ = 201.6, 153.6, 138.5, 137.2, 136.6, 135.2, 133.0, 130.0, 130.0, 129.3, 128.5, 128.4, 128.2, 127.2, 127.1, 124.2, 123.4, 81.9, 73.0, 59.8, 56.7 ppm; FT-IR (neat): v = 3027, 2934, 2896, 1721, 1603, 1472, 1251, 1020, 764, 752, 697, 634 cm^{-1}; HRMS (ESI), *m*/*z*: [M + Na]$^+$ calcd for $C_{23}H_{18}BrNNaO_2^+$ 442.0413; found 442.0420.

(3*R*,3a*R*,8b*S*)-2-Benzyl-3-(2-morpholinophenyl)-2,3,3a,8b-tetrahydro-4*H*-indeno[2,1-*d*]isoxazol-4-one (**6i**)

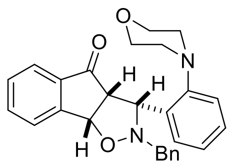

Yield: 42%; white solid; m.p. 186–187 °C; R_f = 0.31 (*n*-hexane/EtOAc 3:1 *v/v*); ^{1}H NMR (500 MHz, DMSO-d_6): δ = 7.80 (td, *J* = 7.3, 1.3 Hz, 1H), 7.73 (d, *J* = 7.6 Hz, 1H), 7.55 (t, *J* = 7.2 Hz, 1H), 7.51 (d, *J* = 7.5 Hz, 1H), 7.29 – 7.17 (m, 5H), 7.10 (d, *J* = 6.6 Hz, 2H), 6.92 (td, *J* = 7.5, 1.3 Hz, 1H), 6.78 (dd, *J* = 7.8, 1.6 Hz, 1H), 5.78 (d, *J* = 6.3 Hz, 1H), 4.69 (d, *J* = 9.6 Hz, 1H), 3.88 (dd, *J* = 9.6, 6.3 Hz, 1H), 3.82 (d, *J* = 15.1 Hz, 1H), 3.80 – 3.72 (m, 4H), 3.67 (d, *J* = 15.1 Hz, 1H), 3.08 – 3.03 (m, 2H), 2.81 – 2.76 (m, 2H) ppm; ^{13}C NMR (125 MHz, DMSO-d_6): δ = 201.8, 153.4, 152.3, 138.7, 137.5, 135.7, 132.5, 129.9, 128.6, 128.5, 128.2, 127.8, 127.1, 126.8, 124.7, 122.1, 121.8, 78.4, 67.2, 67.1, 58.9, 58.3, 53.5 ppm; FT-IR (neat): v = 3030, 2944, 2842, 1715, 1599, 1490, 1454, 1222, 1117, 1080, 928, 742, 704 cm^{-1}; HRMS (ESI), *m*/*z*: [M + Na]$^+$ calcd for $C_{27}H_{26}N_2NaO_3^+$ 449.1836; found 449.1842.

(3*S*,3a*R*,8b*S*)-2-Benzyl-3-(2-morpholinophenyl)-2,3,3a,8b-tetrahydro-4*H*-indeno[2,1-*d*]isoxazol-4-one (**7i**)

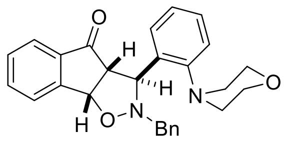

Yield: 34%; white solid; m.p. 183–184 °C; R_f = 0.51 (*n*-hexane/EtOAc 3:1 *v/v*); ^{1}H NMR (500 MHz, DMSO-d_6, 60 °C): δ 7.80 (t, *J* = 7.4 Hz, 1H), 7.77 – 7.71 (m, 3H), 7.64 (t, *J* = 7.4 Hz, 1H), 7.35 (t, *J* = 7.6 Hz, 1H), 7.27 – 7.14 (m, 5H), 7.01 (d, *J* = 6.9 Hz, 2H), 5.86 (d, *J* = 6.4 Hz, 1H), 4.94 (d, *J* = 4.6 Hz, 1H), 3.84 (dd, *J* = 6.5, 4.6 Hz, 1H), 3.74 – 3.59 (m, 6H), 3.06 – 3.02 (m, 2H), 2.68 – 2.63 (m, 2H) ppm; ^{13}C NMR (125 MHz, DMSO-d_6, 60 °C): δ = 203.8, 151.9, 151.5, 137.8, 136.6, 135.9, 134.7, 130.5, 129.1, 128.9, 128.4, 128.1, 127.6, 127.0, 124.9, 123.5, 121.4, 79.2, 66.8, 65.4, 63.6, 59.0, 53.4 ppm; FT-IR (neat): v = 3031, 2892, 2823, 1715, 1598, 1491, 1452, 1294, 1114, 1068, 933, 749, 735 cm^{-1}; HRMS (ESI), *m*/*z*: [M + Na]$^+$ calcd for $C_{27}H_{26}N_2NaO_3^+$ 449.1836; found 449.1833.

(3*R*,3a*R*,8b*S*)-2-Benzyl-3-(pyridin-3-yl)-2,3,3a,8b-tetrahydro-4*H*-indeno[2,1-*d*]isoxazol-4-one (**6j**)

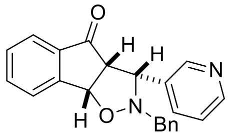

Yield: 55%; white solid; m.p. 149–150 °C; R_f = 0.23 (*n*-hexane/EtOAc 1:2 *v/v*); ^{1}H NMR (500 MHz, DMSO-d_6): δ = 8.44 (dd, *J* = 4.8, 1.6 Hz, 1H), 8.34 (d, *J* = 2.2 Hz, 1H), 7.81 (td, *J* = 7.3, 6.5, 1.9 Hz, 1H), 7.72 (d, *J* = 7.6 Hz, 1H), 7.59 – 7.54 (m, 2H), 7.38 (dt, *J* = 8.0, 1.9 Hz, 1H), 7.29 – 7.16 (m, 4H), 7.11 (d, *J* = 7.0 Hz, 2H), 5.81 (d, *J* = 6.3 Hz, 1H), 4.32 (d, *J* = 9.2 Hz, 1H), 3.92 (dd, *J* = 9.3, 6.7 Hz, 1H), 3.84 (d, *J* = 15.1 Hz, 1H), 3.78 (d, *J* = 15.1 Hz, 1H) ppm; ^{13}C NMR (125 MHz, DMSO-d_6): δ = 202.0, 153.1, 149.9, 149.4, 138.5, 137.6, 136.2, 136.1, 132.2, 130.3, 128.48, 128.45, 127.3, 127.0, 123.8, 122.3, 78.6, 70.9, 60.1, 58.8 ppm; FT-IR (neat): ν = 3029, 2942, 2844, 1715, 1604, 1424, 1338, 1230, 1023, 967, 874, 766, 750, 719, 700 cm^{-1}; HRMS (ESI), *m/z*: [M + K]$^+$ calcd for $C_{22}H_{18}KN_2O_2^+$ 381.1000; found 381.1010.

(3*S*,3a*R*,8b*S*)-2-Benzyl-3-(pyridin-3-yl)-2,3,3a,8b-tetrahydro-4*H*-indeno[2,1-*d*]isoxazol-4-one (**7j**)

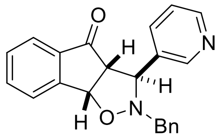

Yield: 27%; white solid; m.p. 172–173 °C; R_f = 0.34 (*n*-hexane/EtOAc 1:2 *v/v*); ^{1}H NMR (500 MHz, DMSO-d_6, 60 °C): δ = 8.69 (d, *J* = 2.2 Hz, 1H), 8.57 (dd, *J* = 4.8, 1.6 Hz, 1H), 7.91 (dt, *J* = 7.9, 2.0 Hz, 1H), 7.80 (td, *J* = 7.4, 1.2 Hz, 1H), 7.77 – 7.73 (m, 2H), 7.64 (t, *J* = 7.5 Hz, 1H), 7.45 (dd, *J* = 7.8, 4.7 Hz, 1H), 7.24 – 7.16 (m, 3H), 7.10 (d, *J* = 7.1 Hz, 2H), 5.86 (d, *J* = 6.4 Hz, 1H), 4.22 (d, *J* = 5.2 Hz, 1H), 3.88 (dd, *J* = 6.5, 5.2 Hz, 1H), 3.70 (d, *J* = 14.5 Hz, 1H), 3.64 (d, *J* = 14.7 Hz, 1H) ppm; ^{13}C NMR (125 MHz, DMSO-d_6, 60 °C): δ = 203.1, 150.9, 149.7, 149.5, 137.5, 136.5, 136.0, 136.0, 134.1, 130.6, 128.4, 128.2, 127.6, 127.1, 123.9, 123.6, 78.8, 69.7, 63.2, 58.7 ppm; FT-IR (neat): ν = 3022, 2918, 2856, 1715, 1604, 1426, 1348, 1277, 1029, 944, 840, 762, 733, 715, 696 cm^{-1}; HRMS (ESI), *m/z*: [M + Na]$^+$ calcd for $C_{22}H_{18}N_2NaO_2^+$ 365.1260; found 365.1267.

(3*R*,3a*R*,8b*S*)-2-Benzyl-3-mesityl-2,3,3a,8b-tetrahydro-4*H*-indeno[2,1-*d*]isoxazol-4-one (**6k**)

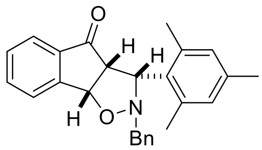

Yield: 32%; white solid; m.p. 177–178 °C; R_f = 0.68 (*n*-hexane/EtOAc 3:1 *v/v*); ^{1}H NMR (500 MHz, DMSO-d_6): δ = 7.78 – 7.70 (m, 3H), 7.63 (t, *J* = 7.3 Hz, 1H), 7.28 – 7.24 (m, 2H), 7.22 – 7.18 (m, 3H), 6.90 (s, 2H), 5.79 (d, *J* = 6.2 Hz, 1H), 4.23 (d, *J* = 8.1 Hz, 1H), 4.01 (dd, *J* = 8.1, 6.2 Hz, 1H), 3.70 (d, *J* = 14.8 Hz, 1H), 3.64 (d, *J* = 14.8 Hz, 1H), 2.54 (br s, 6H), 2.24 (s, 3H) ppm; ^{13}C NMR (125 MHz, DMSO-d_6): δ = 204.0, 149.9 138.2, 137.7, 137.0, 136.3, 135.7, 130.7, 130.3, 129.9, 128.3, 128.2, 127.7, 127.0, 124.0, 78.8, 69.0, 61.2, 59.7, 21.1, 20.6 ppm; FT-IR (neat): ν = 3023, 2926, 2871, 1714, 1605, 1494, 1350, 1278, 1232, 1059, 941, 863, 762, 720 cm^{-1}; HRMS (ESI), *m/z*: [M + Na]$^+$ calcd for $C_{26}H_{25}NNaO_2^+$ 406.1778; found 406.1780.

(3*S*,3a*R*,8b*S*)-2-Benzyl-3-mesityl-2,3,3a,8b-tetrahydro-4*H*-indeno[2,1-*d*]isoxazol-4-one (**7k**)

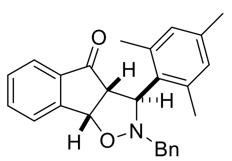

Yield: 20%; white solid; m.p. 191–192 °C; R_f = 0.48 (*n*-hexane/EtOAc 3:1 *v/v*); ^{1}H NMR (500 MHz, DMSO-d_6, 60 °C): δ = 7.81 (td, *J* = 7.4, 1.4 Hz, 1H), 7.74 (d, *J* = 7.6 Hz, 1H), 7.62 – 7.55 (m, 2H), 7.23 – 7.16 (m, 3H), 7.09 (d, *J* = 6.9 Hz, 2H), 6.89 (s, 1H), 6.66 (s, 1H), 5.72 (d, *J* = 6.4 Hz, 1H), 4.33 (d, *J* = 9.1 Hz, 1H), 3.83 (d, *J* = 15.5 Hz, 1H), 3.77 (dd, *J* = 9.1, 6.4 Hz, 1H), 3.60 (d, *J* = 15.6 Hz, 1H), 2.38 (s, 3H), 2.20 (s, 3H), 1.80 (s, 3H) ppm; ^{13}C NMR (125 MHz, DMSO-d_6, 60 °C): δ = 201.3, 153.2, 138.8, 137.5, 137.4, 137.2, 136.2, 135.5, 130.9, 129.8, 129.1, 128.04, 128.00, 127.9, 126.8, 126.7, 121.9, 77.5, 72.0, 58.4, 56.9, 21.9, 20.9, 20.5 ppm; FT-IR (neat): v = 3025, 2916, 2859, 1717, 1653, 1559, 1496, 1464, 1259, 1077, 866, 760, 728 cm^{-1}; HRMS (ESI), *m*/*z*: [M + Na]$^+$ calcd for $C_{26}H_{25}NNaO_2^+$ 406.1778; found 406.1777.

(3*R*,3a*R*,8b*S*)-2-Benzyl-3-(2,6-dichlorophenyl)-2,3,3a,8b-tetrahydro-4*H*-indeno[2,1-*d*]isoxazol-4-one (**6l**)

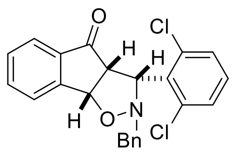

Yield: 40%; white solid; m.p. 193–194 °C; R_f = 0.54 (*n*-hexane/EtOAc 4:1 *v/v*); ^{1}H NMR (500 MHz, DMSO-d_6): δ = 7.80 – 7.73 (m, 3H), 7.65 (td, *J* = 7.5, 1.2 Hz, 1H), 7.52 (d, *J* = 8.0 Hz, 2H), 7.38 (dd, *J* = 8.5, 7.6 Hz, 1H), 7.23 – 7.14 (m, 5H), 5.87 (d, *J* = 6.1 Hz, 1H), 4.66 (d, *J* = 7.5 Hz, 1H), 4.31 (dd, *J* = 7.6, 6.1 Hz, 1H), 3.80 (d, *J* = 14.4 Hz, 1H), 3.70 (d, *J* = 14.3 Hz, 1H) ppm; ^{13}C NMR (125 MHz, DMSO-d_6): δ = 203.5, 149.7, 137.7, 136.4, 136.2, 131.4, 131.3, 131.2, 128.6, 128.4, 128.1, 127.5, 124.5, 79.4, 68.8, 60.9, 60.7 ppm; FT-IR (neat): v = 3031, 2898, 2874, 1713, 1610, 1566, 1428, 1288, 1185, 945, 843, 785, 760, 740, 607 cm^{-1}; HRMS (ESI), *m*/*z*: [M + Na]$^+$ calcd for $C_{23}H_{17}Cl_2NNaO_2^+$ 432.0529; found 432.0536.

(3*R*,3a*R*,8b*S*)-2-Benzyl-3-(perfluorophenyl)-2,3,3a,8b-tetrahydro-4*H*-indeno[2,1-*d*]isoxazol-4-one (**6m**)

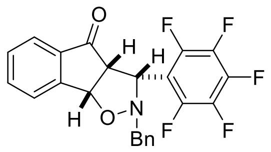

Yield: 18%; white solid; m.p. 163–164 °C; R_f = 0.51 (*n*-hexane/EtOAc 3:1 *v/v*); ^{1}H NMR (500 MHz, DMSO-d_6): δ = 7.82 (t, *J* = 7.4 Hz, 1H), 7.74 (d, *J* = 7.6 Hz, 1H), 7.66 (d, *J* = 7.6 Hz, 1H), 7.59 (t, *J* = 7.4 Hz, 1H), 7.20 – 7.13 (m, 3H), 7.10 – 7.05 (m, 2H), 5.81 (d, *J* = 6.4 Hz, 1H), 4.44 (d, *J* = 8.9 Hz, 1H), 3.95 (d, *J* = 14.4 Hz, 1H), 3.89 – 3.85 (m, 2H) ppm; ^{13}C NMR (125 MHz, DMSO-d_6): δ = 202.1, 152.6, 138.3, 136.8, 136.3, 130.3, 128.8, 128.3, 127.5, 127.3, 122.5, 78.3, 64.8, 59.8, 57.8 ppm; ^{19}F NMR (470 MHz, CDCl3): δ = –130.34 (d, *J* = 21.7 Hz, 1F), –141.56 (d, *J* = 22.5 Hz, 1F), –153.39 (t, *J* = 20.9 Hz, 1F), –161.33 (t, *J* = 18.4 Hz, 1F), –162.11 (t, *J* = 17.7 Hz, 1F) ppm; FT-IR (neat): v = 3025, 2918, 2847, 1706, 1652, 1604, 1522, 1500, 1364, 1270, 1030, 1012, 980, 773, 761, 711, 673 cm^{-1}; HRMS (ESI), *m*/*z*: [M + Na]$^+$ calcd for $C_{23}H_{14}F_5NNaO_2^+$ 454.0837; found 454.0841.

(3*S*,3a*R*,8b*S*)-2-Benzyl-3-(perfluorophenyl)-2,3,3a,8b-tetrahydro-4*H*-indeno[2,1-*d*]isoxazol-4-one (**7m**)

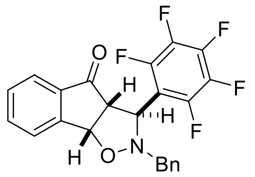

Yield: 37%; white solid; m.p. 131–133 °C; R_f = 0.58 (*n*-hexane/EtOAc 3:1 *v/v*); ^{1}H NMR (500 MHz, DMSO-d_6, 60 °C): δ = 7.81 (t, *J* = 7.3 Hz, 1H), 7.78 – 7.74 (m, 2H), 7.66 (t, *J* = 7.4 Hz, 1H), 7.22 – 7.12 (m, 5H), 5.83 (d, *J* = 6.1 Hz, 1H), 4.36 (d, *J* = 5.5 Hz, 1H), 4.08 (t, *J* = 5.9 Hz, 1H), 3.90 (d, *J* = 13.9 Hz, 1H), 3.79 (d, *J* = 13.8 Hz, 1H) ppm; ^{13}C NMR (125 MHz, DMSO-d_6, 60 °C): δ = 202.9, 150.0, 136.6, 136.3, 136.1, 130.8, 128.5, 128.0, 127.7, 127.3, 123.8, 78.9, 62.2, 61.2, 59.6 ppm; ^{19}F NMR (470 MHz, CDCl3): δ = –139.58 (d, *J* = 16.1 Hz, 2F), –153.31 (t, *J* = 20.8 Hz, 1F), –161.79 (td, *J* = 22.2, 8.0 Hz, 2F) ppm; FT-IR (neat): v = 3087, 3033, 2882, 1718, 1652, 1605, 1524, 1503, 1372, 1274, 1230, 1057, 1007, 969, 954, 756, 696, 670 cm^{-1}; HRMS (ESI), *m*/*z*: [M + Na]$^+$ calcd for $C_{23}H_{14}F_5NNaO_2^+$ 454.0837; found 454.0832.

(3*S*,3a*R*,8b*S*)-2-Benzyl-3-heptyl-2,3,3a,8b-tetrahydro-4*H*-indeno[2,1-*d*]isoxazol-4-one (**6n**)

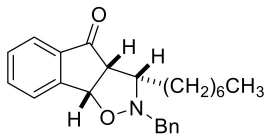

Yield: 21%; white solid; m.p. 59–60 °C; R_f = 0.63 (*n*-hexane/EtOAc 3:1 *v/v*); ^{1}H NMR (500 MHz, DMSO-d_6): δ = 7.74 (t, *J* = 7.8 Hz, 1H), 7.63 (d, *J* = 7.6 Hz, 2H), 7.54 (t, *J* = 7.5 Hz, 1H), 7.26 – 7.16 (m, 5H), 5.67 (d, *J* = 6.6 Hz, 1H), 4.07 (d, *J* = 14.4 Hz, 1H), 3.82 – 3.74 (m, 2H), 3.03 (q, *J* = 7.3 Hz, 1H), 1.62 – 1.52 (m, 1H), 1.40 – 1.14 (m, 10H), 0.85 (t, *J* = 6.9 Hz, 3H) ppm; ^{13}C NMR (125 MHz, DMSO-d_6): δ = 203.3, 152.5, 138.1, 138.0, 135.9, 130.1, 128.9, 128.4, 127.2, 127.1, 122.4, 78.2, 68.8, 59.1, 57.3, 31.7, 29.4, 29.1, 28.2, 26.6, 22.6, 14.4 ppm; FT-IR (neat): v = 3030, 2925, 2855, 1702, 1603, 1466, 1270, 955, 769, 753, 701 cm^{-1}; HRMS (ESI), *m*/*z*: [M + Na]$^+$ calcd for $C_{24}H_{29}NNaO_2^+$ 386.2091; found 386.2090.

(3*R*,3a*R*,8b*S*)-2-Benzyl-3-heptyl-2,3,3a,8b-tetrahydro-4*H*-indeno[2,1-*d*]isoxazol-4-one (**7n**)

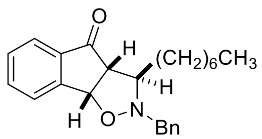

Yield: 55%; white solid; m.p. 64–65 °C; R_f = 0.57 (*n*-hexane/EtOAc 3:1 *v/v*); ^{1}H NMR (500 MHz, DMSO-d_6, 60 °C): δ = 7.74 (t, *J* = 7.3 Hz, 1H), 7.70 – 7.65 (m, 2H), 7.59 (t, *J* = 7.4 Hz, 1H), 7.28 – 7.18 (m, 3H), 7.15 (d, *J* = 7.2 Hz, 2H), 5.60 (d, *J* = 6.4 Hz, 1H), 3.82 (d, *J* = 14.1 Hz, 1H), 3.65 (d, *J* = 14.0 Hz, 1H), 3.53 (dd, *J* = 6.5, 4.8 Hz, 1H), 3.09 (q, *J* = 5.9 Hz, 1H), 1.73 – 1.64 (m, 1H), 1.62 – 1.54 (m, 1H), 1.54 – 1.40 (m, 2H), 1.33 – 1.24 (m, 7H), 0.88 (t, *J* = 6.7 Hz, 3H) ppm; ^{13}C NMR (125 MHz, DMSO-d_6, 60 °C): δ = 204.1, 151.6, 138.0, 136.4, 135.7, 130.3, 128.7, 128.1, 127.5, 127.0, 123.5, 79.0, 68.4, 61.3, 60.0, 32.0, 31.4, 29.1, 28.8, 25.7, 22.2, 14.0 ppm; FT-IR (neat): v = 3034, 2925, 2857, 1708, 1604, 1466, 1352, 1284, 1232, 771, 760, 703 cm^{-1}; HRMS (ESI), *m*/*z*: [M + Na]$^+$ calcd for $C_{24}H_{29}NNaO_2^+$ 386.2091; found 386.2085.

4. Data for compounds **9** and **10**

(3S,3a*R*,9b*R*)-2-Benzyl-3-phenyl-2,3,3a,9b-tetrahydroisoxazolo[4,5-*c*]isoquinolin-5(4*H*)-one (**9a**)

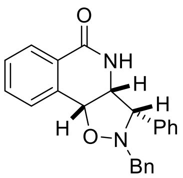

Reaction time: 48 h; yield: 78%; white solid; m.p. 169–170 °C; R_f = 0.14 (*n*-hexane/EtOAc 1:1 *v/v*); ^{1}H NMR (500 MHz, DMSO-*d*$_6$): δ = 7.83 (d, *J* = 7.6 Hz, 1H), 7.59 (td, *J* = 7.5, 1.4 Hz, 1H), 7.45 (t, *J* = 7.8 Hz, 1H), 7.41 (d, *J* = 7.7 Hz, 1H), 7.28 – 7.18 (m, 8H), 7.15 – 7.12 (m, 3H), 5.59 (d, *J* = 7.6 Hz, 1H), 4.76 (td, *J* = 7.7, 3.7 Hz, 1H), 4.28 (d, *J* = 7.7 Hz, 1H), 3.94 (d, *J* = 14.9 Hz, 1H), 3.89 (d, *J* = 15.0 Hz, 1H) ppm; ^{13}C NMR (125 MHz, DMSO-*d*$_6$): δ = 162.6, 138.1, 137.9, 136.3, 132.5, 129.2, 128.7, 128.5, 128.5, 128.34, 128.25, 128.1, 127.8, 127.2, 126.9, 74.6, 71.9, 60.2, 59.6 ppm; FT-IR (neat): v = 3206, 3074, 2855, 1652, 1582, 1412, 1342, 997, 745, 697 cm^{-1}; HRMS (ESI), *m*/*z*: [M + Na]$^+$ calcd for $C_{23}H_{20}N_2NaO_2^+$ 379.1417; found 379.1422.

(4a*R*,5*S*,7a*R*)-6-Benzyl-5-phenyl-4a,5,6,7a-tetrahydroisoxazolo[4,5-*c*]tetrazolo[5,1-*a*]isoquinoline (**10a**)

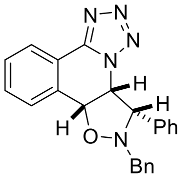

Reaction time: 48 h; yield: 14%; white solid; m.p. 148–150 °C; R_f = 0.36 (*n*-hexane/EtOAc 2:1 *v/v*); ^{1}H NMR (500 MHz, CDCl$_3$): δ = 8.18 (d, *J* = 7.2 Hz, 1H), 7.63 – 7.54 (m, 3H), 7.29 – 7.24 (m, 3H), 7.20 – 7.10 (m, 5H), 6.94 (d, *J* = 6.9 Hz, 2H), 5.91 – 5.85 (m, 2H), 4.44 (d, *J* = 7.8 Hz, 1H), 4.07 (d, *J* = 14.9 Hz, 1H), 3.84 (d, *J* = 14.9 Hz, 1H) ppm; ^{13}C NMR (125 MHz, CDCl$_3$): δ = 149.9, 136.3, 132.3, 129.5, 129.1, 128.7, 128.20, 128.19, 128.1, 128.0, 127.3, 124.9, 119.7, 74.5, 74.3, 63.3, 59.1 ppm; FT-IR (neat): v = 3022, 2859, 1556, 1490, 1447, 983, 788, 738, 702, 694 cm^{-1}; HRMS (ESI), *m*/*z*: [M + Na]$^+$ calcd for $C_{23}H_{19}N_5NaO^+$ 404.1482; found 404.1476.

(3*S*,3a*R*,9b*R*)-2-Benzyl-3-(*p*-tolyl)-2,3,3a,9b-tetrahydroisoxazolo[4,5-*c*]isoquinolin-5(4*H*)-one (**9b**)

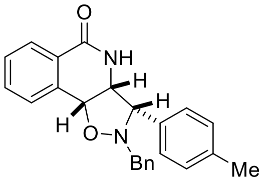

Reaction time: 48 h; yield: 59%; white solid; m.p. 157.5–158.5 °C; R_f = 0.41 (*n*-hexane/EtOAc 1:1 *v/v*); ^{1}H NMR (500 MHz, DMSO-*d*$_6$): δ = 7.83 (d, *J* = 7.3 Hz, 1H), 7.59 (td, *J* = 7.5, 1.4 Hz, 1H), 7.46 (t, *J* = 7.3 Hz, 1H), 7.39 (d, *J* = 7.7 Hz, 1H), 7.27 – 7.15 (m, 6H), 7.07 (d, *J* = 7.9 Hz, 2H), 7.00 (d, *J* = 8.0 Hz, 2H), 5.59 (d, *J* = 7.8 Hz, 1H), 4.70 (td, *J* = 7.8, 3.8 Hz, 1H), 4.20 (d, *J* = 7.8 Hz, 1H), 3.87 (s, 2H), 2.25 (s, 3H) ppm; ^{13}C NMR (125 MHz, DMSO-*d*$_6$): δ = 162.3, 138.0, 137.6, 137.0, 132.9, 132.2, 128.99, 128.95, 128.5, 128.4, 128.2, 128.1, 127.7, 127.0, 126.7, 74.4, 71.8, 60.0, 60.0, 20.9 ppm; FT-IR (neat): v = 3206, 3088, 2895, 1652, 1583, 1409 ,1354, 755, 734 cm^{-1}; HRMS (ESI), *m*/*z*: [M + Na]$^+$ calcd for $C_{24}H_{22}N_2NaO_2^+$ 393.1573; found 393.1573.

(4a*R*,5*S*,7a*R*)-6-Benzyl-5-(*p*-tolyl)-4a,5,6,7a-tetrahydroisoxazolo[4,5-*c*]tetrazolo[5,1-*a*]isoquinoline (**10b**)

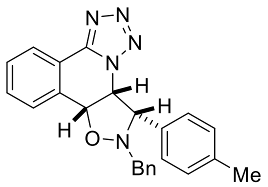

Reaction time: 48 h; yield: 19%; white solid; m.p. 151–152 °C; R_f = 0.57 (*n*-hexane/EtOAc 1:1 *v/v*); ^{1}H NMR (500 MHz, $CDCl_3$): δ = 8.19 (d, *J* = 7.2 Hz, 1H), 7.62 – 7.54 (m, 3H), 7.29 – 7.23 (m, 3H), 7.15 – 7.11 (m, 2H), 6.93 (d, *J* = 7.8 Hz, 2H), 6.82 (d, *J* = 7.7 Hz, 2H), 5.89 – 5.83 (m, 2H), 4.40 (d, *J* = 6.2 Hz, 1H), 4.05 (d, *J* = 14.9 Hz, 1H), 3.81 (d, *J* = 15.0 Hz, 1H), 2.22 (s, 3H) ppm; ^{13}C NMR (125 MHz, $CDCl_3$): δ = 149.9, 138.9, 136.4, 132.3, 129.46, 129.45, 129.2, 129.0, 128.23, 128.21, 128.1, 128.03, 127.99, 127.2, 124.9, 119.8, 74.5, 74.2, 63.3, 59.0, 21.1 ppm; FT-IR (neat): ν = 3067, 3028, 2877, 1557, 1513, 1494 ,1450, 1330, 1111, 1051, 818, 731, 723, 695 cm^{-1}; HRMS (ESI), *m*/*z*: $[M + Na]^+$ calcd for $C_{24}H_{21}N_5NaO^+$ 418.1638; found 418.1640.

3*S*,3a*R*,9b*R*)-2-Benzyl-3-(4-methoxyphenyl)-2,3,3a,9b-tetrahydroisoxazolo[4,5-*c*]isoquinolin-5(4*H*)-one (**9c**)

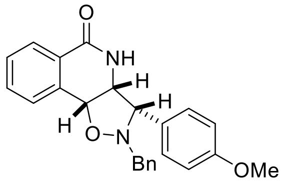

Reaction time: 48 h; yield: 62%; white solid; m.p. 170–171°C; R_f = 0.14 (*n*-hexane/EtOAc 1:1 *v/v*); ^{1}H NMR (500 MHz, DMSO-d_6): δ = 7.86 (d, *J* = 6.9 Hz, 1H), 7.59 (td, *J* = 7.5, 1.4 Hz, 1H), 7.46 (t, *J* = 7.3 Hz, 1H), 7.40 (d, *J* = 7.6 Hz, 1H), 7.27 – 7.16 (m, 6H), 7.04 (d, *J* = 8.7 Hz, 2H), 6.83 (d, *J* = 8.7 Hz, 2H), 5.58 (d, *J* = 7.7 Hz, 1H), 4.68 (td, *J* = 7.7, 3.7 Hz, 1H), 4.19 (d, *J* = 7.7 Hz, 1H), 3.87 (s, 2H), 3.71 (s, 3H) ppm; ^{13}C NMR (125 MHz, DMSO-d_6): δ = 162.6, 159.3, 138.3, 138.2, 132.6, 130.5, 128.7, 128.5, 128.4, 128.3, 127.9, 127.7, 127.2, 126.9, 114.1, 74.3, 71.9, 60.0, 59.4, 55.5 ppm; FT-IR (neat): ν = 3197, 3087, 1652, 1582, 1513, 1251, 1176, 1031, 829, 757 cm^{-1}; HRMS (ESI), *m*/*z*: $[M + Na]^+$ calcd for $C_{24}H_{22}N_2NaO_3^+$ 409.1523; found 409.1526.

(3*S*,3a*R*,9b*R*)-2-Benzyl-3-(4-hydroxyphenyl)-2,3,3a,9b-tetrahydroisoxazolo[4,5-*c*]isoquinolin-5(4*H*)-one (**9d**)

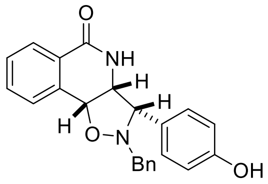

Reaction time: 48 h; yield: 54%; white solid; m.p. 163–164 °C; R_f = 0.12 (*n*-hexane/EtOAc 1:1 *v/v*); ^{1}H NMR (500 MHz, DMSO-d_6): δ = 9.38 (s, 1H), 7.86 (d, *J* = 7.6 Hz, 1H), 7.58 (t, *J* = 7.5 Hz, 1H), 7.46 (t, *J* = 7.5 Hz, 1H), 7.39 (d, *J* = 7.6 Hz, 1H), 7.26 – 7.15 (m, 6H), 6.93 (d, *J* = 8.4 Hz, 2H), 6.66 (d, *J* = 8.5 Hz, 2H), 5.56 (d, *J* = 7.7 Hz, 1H), 4.64 (td, *J* = 7.7, 3.6 Hz, 1H), 4.13 (d, *J* = 7.6 Hz, 1H), 3.86 (d, *J* = 15.2 Hz, 1H), 3.86 (d, *J* = 15.2 Hz, 1H) ppm; ^{13}C NMR (125 MHz, DMSO-d_6): δ = 162.6, 157.4, 138.4, 138.2, 132.6, 130.5, 128.7, 128.5, 128.4, 127.8, 127.2, 126.9, 126.1, 115.5, 74.5, 71.9, 60.0, 59.4 ppm; FT-IR (neat): ν = 3332, 3140, 1645, 1577, 1516, 1454, 1231, 738 cm^{-1}; HRMS (ESI), *m*/*z*: $[M + Na]^+$ calcd for $C_{23}H_{20}N_2NaO_3{}^+$ 395.1366; found 395.1370.

(3*S*,3a*R*,9b*R*)-2-Benzyl-3-(4-morpholinophenyl)-2,3,3a,9b-tetrahydroisoxazolo[4,5-*c*]isoquinolin-5(4*H*)-one (**9e**)

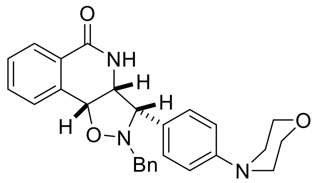

Reaction time: 72 h; yield: 81%; white solid; m.p. 167–168 °C; R_f = 0.25 (*n*-hexane/THF 1:1 *v/v*); ^{1}H NMR (500 MHz, DMSO-d_6): δ = 7.87 (d, *J* = 7.0 Hz, 1H), 7.59 (td, *J* = 7.5, 1.4 Hz, 1H), 7.47 (t, *J* = 7.3 Hz, 1H), 7.39 (d, *J* = 7.6 Hz, 1H), 7.26 – 7.15 (m, 6H), 6.99 (d, *J* = 8.7 Hz, 2H), 6.84 (d, *J* = 8.8 Hz, 2H), 5.56 (d, *J* = 7.7 Hz, 1H), 4.65 (td, *J* = 7.7, 3.7 Hz, 1H), 4.13 (d, *J* = 7.6 Hz, 1H), 3.85 (d, *J* = 15.3 Hz, 1H), 3.84 (d, *J* = 15.1 Hz, 1H), 3.70 (t, *J* = 4.8 Hz, 4H), 3.12 – 3.01 (m, 4H) ppm; ^{13}C NMR (125 MHz, DMSO-d_6): δ = 162.6, 151.0, 138.4, 138.2, 132.5, 130.1, 128.67, 128.4, 128.3, 127.8, 127.2, 126.9, 126.1, 115.1, 74.6, 71.9, 66.6, 59.9, 59.4, 48.6 ppm; FT-IR (neat): ν = 3216, 2963, 2853, 1654, 1583, 1518, 1350, 1239, 1122, 926, 762, 734 cm^{-1}; HRMS (ESI), *m*/*z*: [M + Na]$^+$ calcd for $C_{27}H_{27}N_3NaO_3^+$ 464.1945; found 464.1955.

(3*S*,3a*R*,9b*R*)-2-Benzyl-3-(4-(trifluoromethyl)phenyl)-2,3,3a,9b-tetrahydroisoxazolo[4,5-*c*]isoquinolin-5(4*H*)-one (**9f**)

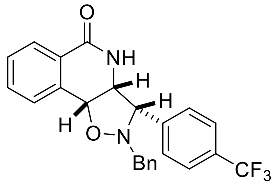

Reaction time: 72 h; yield: 70%; white solid; m.p. 165–166 °C; R_f = 0.39 (*n*-hexane/EtOAc 1:1 *v/v*); ^{1}H NMR (500 MHz, DMSO-d_6): δ = 7.81 (d, *J* = 7.7 Hz, 1H), 7.62 – 7.57 (m, 3H), 7.54 (d, *J* = 3.8 Hz, 1H), 7.48 – 7.40 (m, 2H), 7.32 (d, *J* = 8.1 Hz, 2H), 7.27 – 7.19 (m, 5H), 5.63 (d, *J* = 7.8 Hz, 1H), 4.84 (td, *J* = 7.9, 3.8 Hz, 1H), 4.41 (d, *J* = 7.9 Hz, 1H), 3.98 (d, *J* = 14.8 Hz, 1H), 3.92 (d, *J* = 14.8 Hz, 1H) ppm; ^{13}C NMR (125 MHz, DMSO-d_6): δ = 162.6, 141.5, 137.9, 137.9, 132.6, 130.0, 128.7, 128.6, 128.5 (q, *J* = 31.2 Hz), 128.4, 128.1, 127.7, 127.3, 126.9, 125.3 (q, *J* = 3.8 Hz), 124.7 (q, *J* = 272.0 Hz), 73.9, 71.8, 60.3, 59.6 ppm; ^{19}F NMR (470 MHz, CDCl3): δ = –62.73 (s, 3F) ppm; FT-IR (neat): ν = 3203, 3086, 2899, 1647, 1584, 1410, 1326, 1165, 1132, 1369,, 758, 735 cm^{-1}; HRMS (ESI), *m*/*z*: [M + Na]$^+$ calcd for $C_{24}H_{19}F_3N_2NaO_2^+$ 447.1290; found 447.1286.

(3*S*,3a*R*,9b*R*)-2-Benzyl-3-(2-(trifluoromethyl)phenyl)-2,3,3a,9b-tetrahydroisoxazolo[4,5-*c*]isoquinolin-5(4*H*)-one (**9g**)

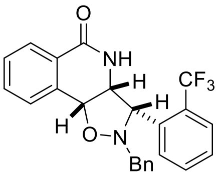

Reaction time: 48 h; yield: 65%; white solid; m.p. 175–176 °C; R_f = 0.26 (*n*-hexane/EtOAc 2:1 *v/v*); ^{1}H NMR (500 MHz, DMSO-d_6): δ = 7.87 (d, *J* = 7.0 Hz, 1H), 7.74 (d, *J* = 7.1 Hz, 1H), 7.62 (td, *J* = 7.5, 1.4 Hz, 1H), 7.55 (d, *J* = 3.8 Hz, 1H), 7.52 – 7.44 (m, 3H), 7.41 (d, *J* = 7.6 Hz, 1H), 7.25 – 7.16 (m, 4H), 7.12 (d, *J* = 6.7 Hz, 2H), 5.66 (d, *J* = 7.8 Hz, 1H), 4.75 (td, *J* = 7.8, 4.0 Hz, 1H), 4.38 (d, *J* = 8.5 Hz, 1H), 3.88 (d, *J* = 14.9 Hz, 1H), 3.82 (d, *J* = 14.9 Hz, 1H) ppm; ^{13}C NMR (125 MHz, DMSO-d_6): δ = 162.5, 137.8, 137.3, 134.5, 132.4, 132.2, 130.7, 129.0 (q, *J* = 34.2 Hz), 128.6, 128.4, 128.3, 128.1, 128.0, 127.6, 127.1, 126.7, 125.9 (q, *J* = 5.7 Hz), 124.5 (q, *J* = 274.0 Hz), 72.0, 71.3, 60.0, 59.9 ppm; ^{19}F NMR (470 MHz, CDCl3): δ = –58.99 (s, 3F) ppm; FT-IR (neat): ν = 3196, 3064, 2922, 1682, 1311, 1164,

1114, 1103, 1033, 778, 737, 696 cm^{-1}; HRMS (ESI), *m/z*: [M + Na]$^+$ calcd for $C_{24}H_{19}F_3N_2NaO_2^+$ 447.1290; found 447.1306.

(4a*R*,5*S*,7a*R*)-6-Benzyl-5-(2-(trifluoromethyl)phenyl)-4a,5,6,7a-tetrahydroisoxazolo[4,5-*c*]tetrazolo[5,1-*a*]isoquinoline (**10g**)

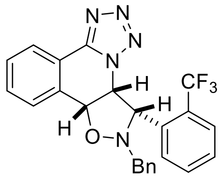

Reaction time: 48 h; yield: 12%; white solid; m.p. 161–162 °C; R_f = 0.51 (*n*-hexane/EtOAc 2:1 *v/v*); ^{1}H NMR (500 MHz, $CDCl_3$): δ = 8.20 (d, *J* = 7.8 Hz, 1H), 7.66 (d, *J* = 7.9 Hz, 1H), 7.63 – 7.55 (m, 3H), 7.29 (t, *J* = 7.7 Hz, 1H), 7.24 – 7.20 (m, 3H), 7.12 (t, *J* = 7.7 Hz, 1H), 7.10 – 7.06 (m, 2H), 6.87 (d, *J* = 7.8 Hz, 1H), 5.95 (t, *J* = 8.5 Hz, 1H), 5.90 (d, *J* = 8.8 Hz, 1H), 4.81 (d, *J* = 7.8 Hz, 1H), 3.97 (d, *J* = 15.0 Hz, 1H), 3.86 (d, *J* = 15.0 Hz, 1H) ppm; ^{13}C NMR (125 MHz, $CDCl_3$): δ = 149.9, 136.2, 135.1, 132.4, 132.1, 131.1 (q, *J* = 1.6 Hz), 129.6, 128.9, 128.2, 128.04, 128.02, 127.9, 127.3, 126.2 (q, *J* = 5.5 Hz), 124.9, 124.1 (q, *J* = 273.7 Hz), 119.5, 74.7, 70.8, 63.6, 59.1 ppm; ^{19}F NMR (470 MHz, $CDCl_3$): δ = –58.25 (s, 3F) ppm; FT-IR (neat): ν = 3072, 3040, 2945, 1455, 1309, 1277, 1176, 1114, 1033, 772, 741, 697 cm^{-1}; HRMS (ESI), *m/z*: [M + Na]$^+$ calcd for $C_{24}H_{18}F_3N_5NaO^+$ 472.1356; found 472.1349.

(3*S*,3a*R*,9b*R*)-2-Benzyl-3-(2-bromophenyl)-2,3,3a,9b-tetrahydroisoxazolo[4,5-*c*]isoquinolin-5(4*H*)-one (**9h**)

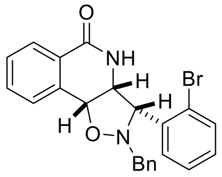

Reaction time: 48 h; yield: 65%; white solid; m.p. 159–161 °C; R_f = 0.40 (*n*-hexane/EtOAc 1:1 *v/v*); ^{1}H NMR (500 MHz, DMSO-d_6): δ = 7.81 (d, *J* = 7.5 Hz, 1H), 7.64 – 7.59 (m, 2H), 7.47 (t, *J* = 7.5 Hz, 1H), 7.43 (d, *J* = 7.7 Hz, 1H), 7.34 (d, *J* = 3.8 Hz, 1H), 7.27 – 7.16 (m, 6H), 7.05 (dd, *J* = 6.0, 3.7 Hz, 1H), 5.65 (d, *J* = 7.3 Hz, 1H), 4.83 (td, *J* = 7.5, 3.9 Hz, 1H), 4.55 (d, *J* = 7.6 Hz, 1H), 3.96 (d, *J* = 14.8 Hz, 1H), 3.91 (d, *J* = 14.8 Hz, 1H) ppm; ^{13}C NMR (125 MHz, DMSO-d_6): δ = 162.7, 137.8, 137.7, 135.5, 132.9, 132.7, 130.7, 130.0, 128.9, 128.7, 128.4, 128.3, 127.88, 127.85, 127.4, 126.9, 124.6, 73.9, 72.2, 60.4, 58.5 ppm; FT-IR (neat): ν = 3190, 3063, 2949, 1675, 1583, 1416, 763, 736 cm^{-1}; HRMS (ESI), *m/z*: [M + Na]$^+$ calcd for $C_{23}H_{19}BrN_2NaO_2^+$ 457.0522; found 457.0531,

(4a*R*,5*S*,7a*R*)-6-Benzyl-5-(2-bromophenyl)-4a,5,6,7a-tetrahydroisoxazolo[4,5-*c*]tetrazolo[5,1-*a*]isoquinoline (**10h**)

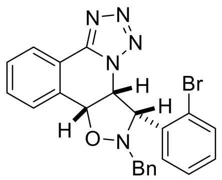

Reaction time: 48 h; yield: 17%; white solid; m.p. 158–159 °C; R_f = 0.74 (*n*-hexane/EtOAc 1:1 *v/v*); ^{1}H NMR (500 MHz, $CDCl_3$): δ = 8.16 (d, *J* = 7.5 Hz, 1H), 7.63 – 7.53 (m, 4H), 7.27 – 7.23 (m, 3H), 7.14 – 7.10 (m, 2H), 7.05 (td, *J* = 7.7, 1.8 Hz, 1H), 6.91 (td, *J* = 7.6, 1.2 Hz, 1H), 6.70 (dd, *J* = 7.8, 1.7 Hz, 1H), 6.02 (t, *J* = 8.4 Hz, 1H), 5.89 (d, *J* = 8.7 Hz, 1H), 4.92 (d, *J* = 8.2 Hz, 1H), 4.06 (d, *J* = 15.0 Hz, 1H), 3.87 (d, *J* = 15.0 Hz, 1H) ppm; ^{13}C NMR (125 MHz, $CDCl_3$): δ = 149.8, 135.9, 135.0, 132.9, 132.3, 132.1, 130.2, 129.6, 128.2, 128.1, 127.6, 127.3, 125.5, 124.9, 119.7, 74.5, 73.2, 62.0, 59.0 ppm; FT-IR

(neat): ν = 3067, 2980, 2879, 1481, 1449, 1435, 1376, 1018, 977, 740, 697, 605 cm^{-1}; HRMS (ESI), *m*/*z*: $[M + Na]^+$ calcd for $C_{23}H_{18}BrN_5NaO^+$ 482.0587; found 482.0577.

(3*S*,3a*R*,9b*R*)-2-Benzyl-3-(2-morpholinophenyl)-2,3,3a,9b-tetrahydroisoxazolo[4,5-*c*]isoquinolin-5(4*H*)-one (**9i**)

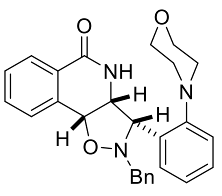

Reaction time: 72 h; yield: 51%; off-white solid; m.p. 162.5–163.5 °C; R_f = 0.20 (*n*-hexane/THF 1:1 *v/v*); ^1H NMR (500 MHz, DMSO-d_6): δ = 7.88 (d, *J* = 6.9 Hz, 1H), 7.61 (td, *J* = 7.5, 1.4 Hz, 1H), 7.49 (t, *J* = 7.5 Hz, 1H), 7.44 (d, *J* = 7.6 Hz, 1H), 7.27 – 7.13 (m, 7H), 7.10 (d, *J* = 3.4 Hz, 1H), 7.04 (dd, *J* = 7.8, 1.7 Hz, 1H), 6.98 (td, *J* = 7.9, 7.4, 1.6 Hz, 1H), 5.63 (d, *J* = 7.3 Hz, 1H), 4.83 (td, *J* = 7.3, 3.5 Hz, 1H), 4.75 (d, *J* = 7.3 Hz, 1H), 3.84 (s, 2H), 3.74 (dtd, *J* = 17.1, 10.9, 2.9 Hz, 4H), 2.89 (ddd, *J* = 11.7, 6.1, 3.1 Hz, 2H), 2.77 (ddd, *J* = 8.5, 6.0, 3.2 Hz, 2H) ppm; ^{13}C NMR (125 MHz, DMSO-d_6): δ = 162.8, 152.6, 137.92, 137.85, 132.6, 131.7, 130.2, 128.88, 128.87, 128.8, 128.4, 128.3, 128.2, 127.3, 126.9, 124.5, 121.4, 72.2, 69.2, 67.3, 60.0, 58.9, 53.8 ppm; FT-IR (neat): ν = 3194, 3062, 2949, 2846, 1675, 1425, 1113, 1031, 741 cm^{-1}; HRMS (ESI), *m*/*z*: $[M + H]^+$ calcd for $C_{27}H_{28}N_3O_3^+$ 442.2125; found 442.2134.

(3*S*,3a*R*,9b*R*)-2-Benzyl-3-(pyridin-3-yl)-2,3,3a,9b-tetrahydroisoxazolo[4,5-*c*]isoquinolin-5(4*H*)-one (**9j**)

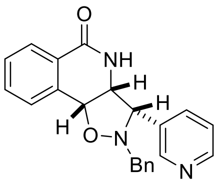

Reaction time: 48 h; yield: 53%; off-white solid; m.p. 164–165 °C; R_f = 0.17 (DCM:acetone 1:1 *v/v*); ^1H NMR (500 MHz, DMSO-d_6): δ = 8.40 (dd, *J* = 4.8, 1.7 Hz, 1H), 8.29 (d, *J* = 2.1 Hz, 1H), 7.80 (dd, *J* = 7.7, 1.4 Hz, 1H), 7.64 (d, *J* = 3.9 Hz, 1H), 7.60 (td, *J* = 7.5, 1.4 Hz, 1H), 7.47 – 7.40 (m, 2H), 7.34 (dt, *J* = 7.9, 2.0 Hz, 1H), 7.27 – 7.18 (m, 6H), 5.64 (d, *J* = 8.0 Hz, 1H), 4.82 (td, *J* = 8.0, 3.9 Hz, 1H), 4.35 (d, *J* = 8.0 Hz, 1H), 3.99 (d, *J* = 14.7 Hz, 1H), 3.92 (d, *J* = 14.7 Hz, 1H) ppm; ^{13}C NMR (125 MHz, DMSO-d_6): δ = 162.6, 150.6, 149.1, 138.0, 137.9, 136.7, 132.7, 132.4, 128.7, 128.6, 128.4, 128.0, 127.6, 127.4, 126.9, 123.5, 72.2, 71.7, 60.3, 59.3 ppm; FT-IR (neat): ν = 3193, 3051, 2958, 1667, 1496, 1417, 1037, 735, 715 cm^{-1}; HRMS (ESI), *m*/*z*: $[M + Na]^+$ calcd for $C_{22}H_{19}N_3NaO_2^+$ 380.1369; found 380.1360.

(3*S*,3a*R*,9b*R*)-2-Benzyl-3-mesityl-2,3,3a,9b-tetrahydroisoxazolo[4,5-*c*]isoquinolin-5(4*H*)-one (**9k**)

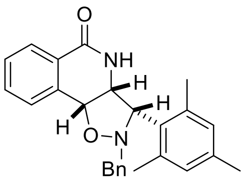

Reaction time: 48 h; yield: 90%; white solid; m.p. 175–176 °C; R_f = 0.34 (*n*-hexane/EtOAc 2:1 *v/v*); ^1H NMR (500 MHz, DMSO-d_6, 60 °C): δ = 8.25 (br s, 1H), 8.04 (d, *J* = 7.7 Hz, 1H), 7.60 (td, *J* = 7.4, 1.5 Hz, 1H), 7.55 – 7.50 (m, 2H), 7.27 – 7.17 (m, 5H), 6.88 (s, 2H), 5.43 (d, *J* = 7.5 Hz, 1H), 4.76 (td, *J* = 7.2, 2.5 Hz, 1H), 4.29 (d, *J* = 6.9 Hz, 1H), 3.72 (d, *J* = 14.7 Hz, 1H), 3.66 (d, *J* = 14.8 Hz, 1H), 2.50 (s, 6H), 2.22 (s, 3H) ppm; ^{13}C NMR (125 MHz, DMSO-d_6, 60 °C): δ = 161.5, 138.3, 138.1, 137.0, 134.6, 132.4, 130.3, 129.9, 129.3, 129.1, 128.4, 128.2, 127.6, 127.3, 127.0, 76.9, 71.8, 60.7,

60.1, 21.2, 20.6 ppm; FT-IR (neat): v = 3179, 3044, 2915, 1679, 1586, 1422, 844, 766, 701 cm^{-1}; HRMS (ESI), *m*/*z*: [M + K]$^+$ calcd for $C_{26}H_{26}KN_2O_2^+$ 437.1626; found 437.1635.

(3*S*,3a*R*,9b*R*)-2-Benzyl-3-(2,6-dichlorophenyl)-2,3,3a,9b-tetrahydroisoxazolo[4,5-*c*]isoquinolin-5(4*H*)-one (**9l**)

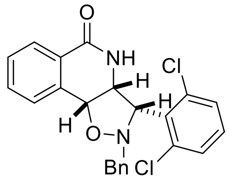

Reaction time: 72 h; yield: 72%; white solid; m.p. 179–180 °C; R_f = 0.43 (*n*-hexane/EtOAc 1:1 *v*/*v*); ^{1}H NMR (500 MHz, DMSO-d_6, 60 °C): δ = 8.48 (br s 1H), 8.05 (d, *J* = 7.4 Hz, 1H), 7.61 (t, *J* = 7.4 Hz, 1H), 7.58 – 7.53 (m, 2H), 7.48 (d, *J* = 8.0 Hz, 2H), 7.35 (t, *J* = 8.1 Hz, 1H), 7.24 – 7.13 (m, 5H), 5.53 (d, *J* = 7.2 Hz, 1H), 5.06 (td, *J* = 6.5, 2.4 Hz, 1H), 4.71 (d, *J* = 6.2 Hz, 1H), 3.85 (d, *J* = 14.3 Hz, 1H), 3.72 (d, *J* = 14.3 Hz, 1H) ppm; ^{13}C NMR (125 MHz, DMSO-d_6, 60 °C): δ = 161.3, 137.4, 136.2, 134.1, 132.4, 130.9, 130.7, 130.0, 129.8, 129.5, 128.3, 128.0, 127.6, 127.4, 127.1, 77.0, 72.0, 60.9, 60.2 ppm; FT-IR (neat): v = 3176, 3035, 2910, 1661, 1582, 1434, 1416, 783, 762, 736 cm^{-1}; HRMS (ESI), *m*/*z*: [M + Na]$^+$ calcd for $C_{23}H_{18}Cl_2N_2NaO_2^+$ 447.0638; found 447.0644.

(4a*R*,5*S*,7a*R*)-6-Benzyl-5-(2,6-dichlorophenyl)-4a,5,6,7a-tetrahydroisoxazolo[4,5-*c*]tetrazolo[5,1-*a*]isoquinoline (**10l**)

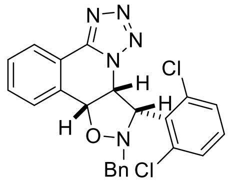

Reaction time: 72 h; yield: 13%; white solid; m.p. 188–189 °C; R_f = 0.68 (*n*-hexane/EtOAc 1:1 *v*/*v*); ^{1}H NMR (500 MHz, $CDCl_3$): δ = 8.34 (dd, *J* = 7.2, 1.7 Hz, 1H), 7.69 (dd, *J* = 7.4, 1.7 Hz, 1H), 7.66 – 7.59 (m, 2H), 7.41 (d, *J* = 8.0 Hz, 2H), 7.31 – 7.16 (m, 6H), 6.02 (dd, *J* = 6.7, 5.3 Hz, 1H), 5.92 (d, *J* = 6.7 Hz, 1H), 5.08 (d, *J* = 5.3 Hz, 1H), 3.94 (d, *J* = 14.4 Hz, 1H), 3.85 (d, *J* = 14.3 Hz, 1H) ppm; ^{13}C NMR (125 MHz, $CDCl_3$): δ = 148.9, 136.5, 132.2, 131.4, 130.9, 130.7, 129.5, 129.1, 128.3, 128.0, 127.3, 126.0, 120.5, 75.53, 75.46, 64.3, 60.1 ppm; FT-IR (neat): v = 3090, 2951, 2919, 1488, 1455, 1435, 1318, 1049, 786, 773, 749, 624 cm^{-1}; HRMS (ESI), *m*/*z*: [M + Na]$^+$ calcd for $C_{23}H_{17}Cl_2N_5NaO^+$ 472.0702; found 472.0711.

(3*S*,3a*R*,9b*R*)-2-Benzyl-3-(perfluorophenyl)-2,3,3a,9b-tetrahydroisoxazolo[4,5-*c*]isoquinolin-5(4*H*)-one (**9m**)

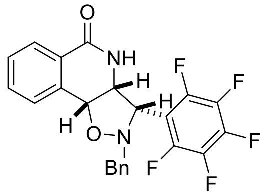

Reaction time: 48 h; yield: 65%; white solid; m.p. 192–193 °C; R_f = 0.18 (*n*-hexane/EtOAc 2:1 *v*/*v*); ^{1}H NMR (500 MHz, DMSO-d_6): δ = 7.94 (d, *J* = 7.5 Hz, 1H), 7.76 (d, *J* = 2.7 Hz, 1H), 7.62 (td, *J* = 7.5, 1.4 Hz, 1H), 7.52 – 7.47 (m, 2H), 7.22 – 7.15 (m, 5H), 5.66 (d, *J* = 7.6 Hz, 1H), 4.85 (td, *J* = 7.5, 3.1 Hz, 1H), 4.59 (d, *J* = 7.4 Hz, 1H), 4.08 (d, *J* = 13.8 Hz, 1H), 4.02 (d, *J* = 13.8 Hz, 1H) ppm; ^{13}C NMR (125 MHz, DMSO-d_6): δ = 162.6, 137.0, 136.3, 132.6, 129.1, 129.0, 128.7, 128.3, 128.1, 127.6, 127.0, 71.8, 68.0, 60.9, 59.3 ppm; ^{19}F NMR (470 MHz, CDCl3): δ = –133.97 (br s, 1F), –140.75 (br s, 1F), –156.44 (t, *J* = 22.2 Hz, 1F), –164.03 (d, *J* = 276.0 Hz, 2F) ppm; FT-IR (neat): v =

3182, 3052, 2928, 1657, 1522, 1499, 1415, 1348, 970, 738, 693 cm^{-1}; HRMS (ESI), *m*/*z*: [M + Na]$^+$ calcd for $C_{23}H_{15}F_5N_2NaO_2^+$ 469.0946; found 464.0960.

3*S*,3a*R*,9b*R*)-2-Benzyl-3-heptyl-2,3,3a,9b-tetrahydroisoxazolo[4,5-*c*]isoquinolin-5(4*H*)-one (**9n**)

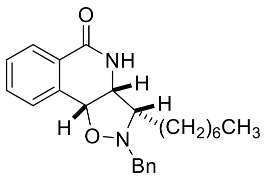

Reaction time: 48 h; yield: 69%; white solid; m.p. 138–139 °C; R_f = 0.37 (*n*-hexane/EtOAc 1:1 *v/v*); ^{1}H NMR (500 MHz, DMSO-d_6): δ = 7.98 – 7.92 (m, 2H), 7.56 (td, *J* = 7.5, 1.4 Hz, 1H), 7.48 (t, *J* = 7.5 Hz, 1H), 7.38 (d, *J* = 7.5 Hz, 1H), 7.33 – 7.21 (m, 5H), 5.41 (d, *J* = 7.1 Hz, 1H), 4.63 (td, *J* = 6.8, 2.4 Hz, 1H), 4.04 (d, *J* = 13.8 Hz, 1H), 3.98 (d, *J* = 13.8 Hz, 1H), 3.23 (q, *J* = 6.8 Hz, 1H), 1.53 – 1.45 (m, 1H), 1.29 – 1.10 (m, 10H), 0.84 (t, *J* = 7.1 Hz, 3H) ppm; ^{13}C NMR (125 MHz, DMSO-d_6): δ = 163.0, 138.5, 135.8, 132.6, 129.3, 129.2, 129.0, 128.5, 128.1, 127.4, 127.3, 71.1, 70.2, 61.2, 57.9, 31.7, 29.4, 29.1, 28.4, 26.7, 22.6, 14.4 ppm; FT-IR (neat): ν = 3209, 2915, 2849, 1664, 1583, 1418, 1339, 761, 727, 694 cm^{-1}; HRMS (ESI), *m*/*z*: [M + Na]$^+$ calcd for $C_{24}H_{30}N_2NaO_2^+$ 401.2199; found 401.2200.

(4a*R*,5*S*,7a*R*)-6-Benzyl-5-heptyl-4a,5,6,7a-tetrahydroisoxazolo[4,5-*c*]tetrazolo[5,1-*a*]isoquinoline (**10n**)

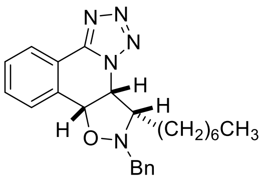

Reaction time: 48 h; yield: 11%; white solid; m.p. 119–120 °C; R_f = 0.80 (*n*-hexane/EtOAc 1:1 *v/v*); ^{1}H NMR (500 MHz, $CDCl_3$): δ = 8.24 (dd, *J* = 7.5, 1.8 Hz, 1H), 7.60 – 7.52 (m, 3H), 7.38 – 7.29 (m, 5H), 5.82 – 5.76 (m, 1H), 4.15 (d, *J* = 13.5 Hz, 1H), 4.09 (d, *J* = 13.6 Hz, 1H), 3.68 (q, *J* = 6.9 Hz, 1H), 1.28 – 1.17 (m, 4H), 1.15 – 1.06 (m, 4H), 1.04 – 0.95 (m, 4H), 0.83 (t, *J* = 7.2 Hz, 3H) ppm; ^{13}C NMR (125 MHz, $CDCl_3$): δ = 150.3, 136.1, 133.1, 132.4, 129.8, 128.8, 128.7, 128.4, 127.7, 125.4, 119.1, 73.8, 69.1, 61.4, 60.4, 31.6, 29.0, 29.0, 27.3, 26.2, 22.5, 14.0 ppm; FT-IR (neat): ν = 3064, 2947, 2926, 2855, 1493, 1455, 1111, 788, 745, 710 cm^{-1}; HRMS (ESI), *m*/*z*: [M + Na]$^+$ calcd for $C_{24}H_{29}N_5NaO^+$ 404.2445; found 404.2435.

5. Data for compounds **12** and **13**

(3*R*,3a*R*,9b*R*)-2-Benzyl-3-phenyl-2,3,3a,9b-tetrahydroisoxazolo[4,5-*c*]isoquinolin-5(4*H*)-one (**12a**)

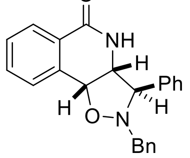

Yield: 82%; white solid; m.p. 160–161 °C; R_f = 0.11 (*n*-hexane/EtOAc 1:1 *v/v*); ^{1}H NMR (500 MHz, DMSO-d_6, 60 °C): δ = 8.36 (br s, 1H), 8.04 (d, *J* = 7.6 Hz, 1H), 7.61 – 7.50 (m, 6H), 7.40 (t, *J* = 7.5 Hz, 2H), 7.33 (t, *J* = 7.3 Hz, 1H), 7.28 – 7.18 (m, 4H), 5.23 (d, *J* = 6.2 Hz, 1H), 4.42 (t, *J* = 4.9 Hz, 1H), 4.17 (d, *J* = 4.0 Hz, 1H), 3.97 (d, *J* = 14.4 Hz, 1H), 3.90 (d, *J* = 14.3 Hz, 1H) ppm; ^{13}C NMR (125 MHz, DMSO-d_6, 60 °C): δ = 161.6, 138.9, 137.8, 133.8, 132.3, 130.0, 130.0, 128.7, 128.6, 128.14, 128.08, 128.06, 128.0, 127.3,

127.1, 78.3, 72.4, 65.0, 60.8 ppm; FT-IR (neat): ν = 3179, 3047, 2934, 1668, 1606, 1586, 1419, 1350, 748, 700 cm^{-1}; HRMS (ESI), *m/z*: [M + Na]$^+$ calcd for $C_{23}H_{20}N_2NaO_2^+$ 379.1417; found 379.1420.

(4a*R*,5*R*,7a*R*)-6-Benzyl-5-phenyl-4a,5,6,7a-tetrahydroisoxazolo[4,5-*c*]tetrazolo[5,1-*a*]isoquinoline (**13a**)

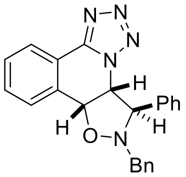

Yield: 14%; white solid; m.p. 170–171 °C; R_f = 0.40 (*n*-hexane/EtOAc 2:1 *v/v*); ^{1}H NMR (500 MHz, $CDCl_3$): δ = 8.32 (dd, *J* = 7.3, 1.6 Hz, 1H), 7.76 (d, *J* = 7.2 Hz, 2H), 7.67 – 7.57 (m, 3H), 7.51 – 7.47 (m, 2H), 7.44 – 7.40 (m, 1H), 7.30 – 7.22 (m, 5H), 5.60 (d, *J* = 6.3 Hz, 1H), 5.24 (dd, *J* = 6.4, 4.4 Hz, 1H), 4.39 (d, *J* = 4.4 Hz, 1H), 3.99 (d, *J* = 14.5 Hz, 1H), 3.77 (d, *J* = 14.5 Hz, 1H) ppm; ^{13}C NMR (125 MHz, $CDCl_3$): δ = 148.8, 136.2, 136.1, 132.2, 131.2, 130.9, 129.2, 129.0, 129.0, 128.9, 128.3, 128.2, 127.6, 126.0, 120.5, 78.52, 75.5, 68.3, 59.7 ppm; FT-IR (neat): ν = 3081, 3022, 2859, 1555, 1490, 1447, 1157, 983, 788, 738, 702 cm^{-1}; HRMS (ESI), *m/z*: [M + Na]$^+$ calcd for $C_{23}H_{19}N_5NaO^+$ 404.1482; found 404.1474.

(3*R*,3a*R*,9b*R*)-2-Benzyl-3-(p-tolyl)-2,3,3a,9b-tetrahydroisoxazolo[4,5-*c*]isoquinolin-5(4*H*)-one (**12b**)

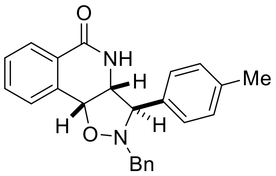

Yield: 72%; white solid; m.p. 156–157 °C; R_f = 0.43 (*n*-hexane/EtOAc 1:1 *v/v*); ^{1}H NMR (500 MHz, DMSO-d_6, 60 °C): δ = 8.33 (br s, 1H), 8.04 (d, *J* = 7.6 Hz, 1H), 7.61 – 7.49 (m, 3H), 7.47 (d, *J* = 7.8 Hz, 2H), 7.28 – 7.17 (m, 7H), 5.22 (d, *J* = 6.2 Hz, 1H), 4.38 (t, *J* = 5.2 Hz, 1H), 4.11 (d, *J* = 4.1 Hz, 1H), 3.95 (d, *J* = 14.5 Hz, 1H), 3.86 (d, *J* = 14.4 Hz, 1H), 2.32 (s, 3H) ppm; ^{13}C NMR (125 MHz, DMSO-d_6, 60 °C): δ = 161.6, 137.9, 137.3, 135.8, 133.9, 132.3, 130.00, 129.45, 129.3, 128.7, 128.2, 128.0, 127.3, 127.1, 78.2, 72.4, 64.9, 60.7, 20.9 ppm; FT-IR (neat): ν = 3186, 3023, 2924, 1667, 1496, 1418, 1026, 762, 741, 695 cm^{-1}; HRMS (ESI), *m/z*: [M + Na]$^+$ calcd for $C_{24}H_{22}N_2NaO_2^+$ 393.1573; found 393.1580.

(4a*R*,5*R*,7a*R*)-6-Benzyl-5-(p-tolyl)-4a,5,6,7a-tetrahydroisoxazolo[4,5-*c*]tetrazolo[5,1-*a*]isoquinoline (**13a**)

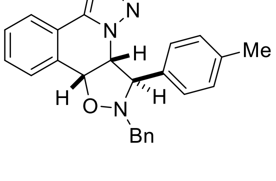

Yield: 14%; white solid; m.p. 162–163 °C; R_f = 0.66 (*n*-hexane/EtOAc 1:1 *v/v*); ^{1}H NMR (500 MHz, $CDCl_3$): δ = 8.31 (dd, *J* = 7.2, 1.6 Hz, 1H), 7.66 – 7.57 (m, 5H), 7.30 (d, *J* = 7.8 Hz, 2H), 7.28 – 7.22 (m, 5H), 5.59 (d, *J* = 6.5 Hz, 1H), 5.22 (dd, *J* = 6.5, 4.6 Hz, 1H), 4.33 (d, *J* = 4.6 Hz, 1H), 3.99 (d, *J* = 14.5 Hz, 1H), 3.73 (d, *J* = 14.5 Hz, 1H), 2.42 (s, 3H) ppm; ^{13}C NMR (125 MHz, $CDCl_3$): δ = 148.8, 138.9, 136.6, 133.4, 132.2, 131.2, 130.8, 129.9, 129.2, 128.8, 128.19, 128.15, 127.4, 126.0, 120.5, 78.5, 75.4, 68.4, 59.6, 21.2 ppm; FT-IR (neat): ν = 3065, 3031, 2896, 1494, 1452, 1430, 1367, 1318, 1056, 964, 817, 757, 696 cm^{-1}; HRMS (ESI), *m/z*: [M + Na]$^+$ calcd for $C_{24}H_{21}N_5NaO^+$ 418.1638; found 418.1644.

(3*R*,3a*R*,9b*R*)-2-Benzyl-3-(4-methoxyphenyl)-2,3,3a,9b-tetrahydroisoxazolo[4,5-*c*]isoquinolin-5(4*H*)-one (**12c**)

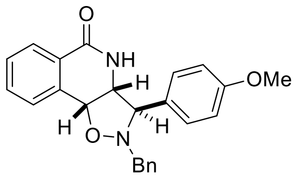

Yield: 83%; white solid; m.p. 151–152 °C; R_f = 0.40 (*n*-hexane/EtOAc 1:1 *v/v*); ^{1}H NMR (500 MHz, DMSO-d_6, 60 °C): δ = 8.31 (br s, 1H), 8.02 (d, *J* = 7.6 Hz, 1H), 7.59 (td, *J* = 7.4, 1.5 Hz, 1H), 7.56 – 7.51 (m, 2H), 7.49 (d, *J* = 8.8 Hz, 2H), 7.28 – 7.18 (m, 5H), 6.96 (d, *J* = 8.7 Hz, 2H), 5.22 (d, *J* = 6.3 Hz, 1H), 4.37 (ddd, *J* = 6.2, 4.2, 1.7 Hz, 1H), 4.06 (d, *J* = 4.2 Hz, 1H), 3.94 (d, *J* = 14.5 Hz, 1H), 3.84 (d, *J* = 14.5 Hz, 1H), 3.77 (s, 3H) ppm; ^{13}C NMR (125 MHz, DMSO-d_6, 60 °C): δ = 161.6, 159.3, 138.0, 133.9, 132.4, 130.6, 130.0, 130.0, 129.3, 128.7, 128.2, 128.1, 127.3, 127.1, 114.2, 78.1, 72.3, 64.8, 60.5, 55.4 ppm; FT-IR (neat): ν = 3175, 3035, 2924, 1672, 1511, 1415, 1249, 1173, 1029, 759 cm^{-1}; HRMS (ESI), *m*/*z*: [M + Na]$^+$ calcd for $C_{24}H_{22}N_2NaO_3^+$ 409.1523; found 409.1529.

(4a*R*,5*R*,7a*R*)-6-Benzyl-5-(4-methoxyphenyl)-4a,5,6,7a-tetrahydroisoxazolo[4,5-*c*]tetrazolo[5,1-*a*]isoquinoline (**13c**)

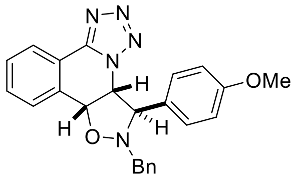

Yield: 14%; white solid; m.p. 139–140 °C; R_f = 0.57 (*n*-hexane/EtOAc 1:1 *v/v*); ^{1}H NMR (500 MHz, $CDCl_3$): δ = 8.31 (dd, *J* = 7.3, 1.7 Hz, 1H), 7.68 – 7.57 (m, 5H), 7.30 – 7.22 (m, 5H), 7.01 (d, *J* = 8.7 Hz, 2H), 5.60 (d, *J* = 6.6 Hz, 1H), 5.22 (dd, *J* = 6.6, 4.6 Hz, 1H), 4.29 (d, *J* = 4.6 Hz, 1H), 3.99 (d, *J* = 14.6 Hz, 1H), 3.86 (s, 3H), 3.73 (d, *J* = 14.5 Hz, 1H) ppm; ^{13}C NMR (125 MHz, $CDCl_3$): δ = 160.1, 148.8, 136.5, 132.2, 131.2, 130.8, 130.0, 129.2, 128.8, 128.20, 128.17, 127.4, 126.0, 120.5, 114.6, 78.3, 75.3, 68.3, 59.5, 55.4 ppm; FT-IR (neat): ν = 3064, 3032, 2891, 2835, 1612, 1516, 1454, 1247, 1182, 1033, 962, 833, 754, 712, 695 cm^{-1}; HRMS (ESI), *m*/*z*: [M + Na]$^+$ calcd for $C_{24}H_{21}N_5NaO_2^+$ 434.1587; found 434.1577.

(3*R*,3a*R*,9b*R*)-2-Benzyl-3-(4-hydroxyphenyl)-2,3,3a,9b-tetrahydroisoxazolo[4,5-*c*]isoquinolin-5(4*H*)-one (**12d**)

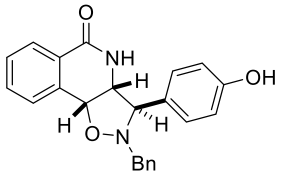

Yield: 75%; white solid; m.p. 161–162.5 °C; R_f = 0.20 (*n*-hexane/EtOAc 1:1 *v/v*); ^{1}H NMR (500 MHz, DMSO-d_6, 60 °C): δ = 9.28 (br s, 1H), 8.28 (br s, 1H), 8.03 (d, *J* = 7.6 Hz, 1H), 7.59 (td, *J* = 7.4, 1.5 Hz, 1H), 7.54 – 7.48 (m, 2H), 7.37 (d, *J* = 8.5 Hz, 2H), 7.28 – 7.17 (m, 5H), 6.81 (d, *J* = 8.4 Hz, 2H), 5.22 (d, *J* = 6.4 Hz, 1H), 4.36 (t, *J* = 4.8 Hz, 2H), 4.00 (d, *J* = 4.3 Hz, 1H), 3.93 (d, *J* = 14.5 Hz, 1H), 3.81 (d, *J* = 14.5 Hz, 1H) ppm; ^{13}C NMR (125 MHz, DMSO-d_6, 60 °C): δ = 161.6, 157.4, 138.1, 134.1, 132.4, 130.0, 129.4, 129.3, 128.71, 128.70, 128.2, 128.0, 127.3, 127.1, 115.6, 78.3, 72.3, 64.7, 60.4 ppm; FT-IR (neat): ν = 3328, 3196, 3047, 1679, 1520, 1419, 1261, 1179, 765, 728, 699 cm^{-1}; HRMS (ESI), *m*/*z*: [M + Na]$^+$ calcd for $C_{23}H_{20}N_2NaO_3^+$ 395.1366; found 395.1371.

(3*R*,3a*R*,9b*R*)-2-Benzyl-3-(4-morpholinophenyl)-2,3,3a,9b-tetrahydroisoxazolo[4,5-*c*]isoquinolin-5(4*H*)-one (**12e**)

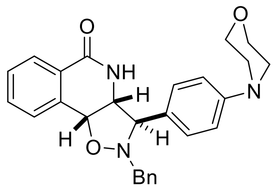

Yield: 84%; white solid; m.p. 158.5–159.5 °C; R_f = 0.17 (*n*-hexane/THF 1:1 *v/v*); ^{1}H NMR (500 MHz, DMSO-d_6, 60 °C): δ = 8.28 (br s, 1H), 8.02 (d, *J* = 7.6 Hz, 1H), 7.59 (td, *J* = 7.4, 1.5 Hz, 1H), 7.55 – 7.49 (m, 2H), 7.42 (d, *J* = 8.6 Hz, 2H), 7.28 – 7.18 (m, 5H), 6.96 (d, *J* = 8.5 Hz, 2H), 5.22 (d, *J* = 6.3 Hz, 1H), 4.37 (t, *J* = 4.6 Hz, 1H), 4.02 (d, *J* = 4.3 Hz, 1H), 3.94 (d, *J* = 14.5 Hz, 1H), 3.82 (d, *J* = 14.5 Hz, 1H), 3.75 (t, *J* = 4.8 Hz, 4H), 3.13 (t, *J* = 4.8 Hz, 4H) ppm; ^{13}C NMR (125 MHz, DMSO-d_6, 60 °C): δ = 161.6, 151.0, 138.1, 134.0, 132.3, 130.0, 129.4, 128.9, 128.8, 128.7, 128.1, 128.0, 127.3, 127.0, 115.2, 78.2, 72.3, 66.4, 64.7, 60.4, 48.7 ppm; FT-IR (neat): v = 3184, 3043, 2954, 1667, 1516, 1417, 1241, 1123, 930, 734 cm^{-1}; HRMS (ESI), *m*/*z*: [M + Na]$^+$ calcd for $C_{27}H_{27}N_3NaO_3^+$ 464.1945; found 464.1949.

(4a*R*,5*R*,7a*R*)-6-Benzyl-5-(4-morpholinophenyl)-4a,5,6,7a-tetrahydroisoxazolo[4,5-*c*]tetrazolo[5,1-*a*]isoquinoline (**13e**)

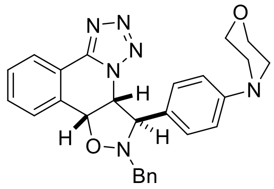

Yield: 14%; white solid; m.p. 164–166 °C; R_f = 0.43 (*n*-hexane/EtOAc 1:1 *v/v*); ^{1}H NMR (500 MHz, $CDCl_3$): δ = 8.31 (dd, *J* = 7.3, 1.7 Hz, 1H), 7.66 – 7.57 (m, 5H), 7.31 – 7.22 (m, 5H), 7.03 (d, *J* = 8.2 Hz, 2H), 5.59 (d, *J* = 6.6 Hz, 1H), 5.22 (dd, *J* = 6.6, 4.6 Hz, 1H), 4.26 (d, *J* = 4.7 Hz, 1H), 3.99 (d, *J* = 14.6 Hz, 1H), 3.90 (t, *J* = 4.8 Hz, 4H), 3.71 (d, *J* = 14.5 Hz, 1H), 3.24 (t, *J* = 4.8 Hz, 4H) ppm; ^{13}C NMR (125 MHz, $CDCl_3$): δ = 148.8, 136.7, 132.2, 131.2, 130.8, 129.3, 129.2, 128.8, 128.2, 127.4, 126.0, 120.5, 116.0, 78.4, 75.3, 68.2, 66.8, 59.5, 49.0 ppm; FT-IR (neat): v = 3034, 2949, 2894, 1608, 1516, 1454, 1235, 1118, 1053, 923, 761, 712, 696 cm^{-1}; HRMS (ESI), *m*/*z*: [M + Na]$^+$ calcd for $C_{27}H_{26}N_6NaO_2^+$ 489.2009; found 489.2011.

(3*R*,3a*R*,9b*R*)-2-Benzyl-3-(4-(trifluoromethyl)phenyl)-2,3,3a,9b-tetrahydroisoxazolo[4,5-*c*]isoquinolin-5(4*H*)-one (**12f**)

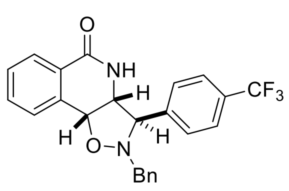

Yield: 85%; white solid; m.p. 158–159 °C; R_f = 0.31 (*n*-hexane/EtOAc 21:1 *v/v*); ^{1}H NMR (500 MHz, DMSO-d_6, 60 °C): δ = 8.39 (br s, 1H), 8.04 (d, *J* = 7.6 Hz, 1H), 7.80 (d, *J* = 8.1 Hz, 2H), 7.74 (d, *J* = 8.2 Hz, 2H), 7.61 (td, *J* = 7.4, 1.5 Hz, 1H), 7.57 – 7.52 (m, 2H), 7.27 – 7.17 (m, 5H), 5.24 (d, *J* = 6.1 Hz, 1H), 4.45 (ddd, *J* = 5.9, 3.9, 1.7 Hz, 1H), 4.31 (d, *J* = 3.8 Hz, 1H), 3.98 (s, 2H) ppm; ^{13}C NMR (125 MHz, DMSO-d_6, 60 °C): δ = 161.8, 144.0, 137.7, 134.0, 132.7, 130.3, 129.8, 129.12, 129.07, 128.9 (q, *J* = 27.5 Hz), 128.4, 128.2, 127.6, 127.4, 125.7 (q, *J* = 3.8 Hz), 124.7 (q, *J* = 272.1 Hz), 77.8, 72.8, 65.3, 61.4 ppm; ^{19}F NMR (470 MHz, DMSO-d_6): δ = –62.69 (s, 3F) ppm; FT-IR (neat): v = 3182, 3046, 1668, 1585, 1421, 1328, 1165, 1113, 1070, 837, 759, 699 cm^{-1}; HRMS (ESI), *m*/*z*: [M + Na]$^+$ calcd for $C_{24}H_{19}F_3N_2NaO_2^+$ 447.1291; found 447.1299.

(4aR,5R,7aR)-6-Benzyl-5-(4-(trifluoromethyl)phenyl)-4a,5,6,7a-tetrahydroisoxazolo[4,5-*c*]tetrazolo[5,1-*a*]isoquinoline (**13f**)

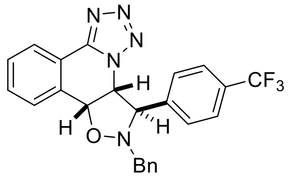

Yield: 14%; white solid; m.p. 164–165 °C; R_f = 0.66 (*n*-hexane/EtOAc 2:1 *v/v*); ^{1}H NMR (500 MHz, $CDCl_3$): δ = 8.33 (dd, J = 7.1, 1.7 Hz, 1H), 7.90 (d, J = 8.0 Hz, 2H), 7.74 (d, J = 8.1 Hz, 2H), 7.69 – 7.60 (m, 3H), 7.30 – 7.22 (m, 5H), 5.60 (d, J = 6.4 Hz, 1H), 5.22 (dd, J = 6.4, 4.4 Hz, 1H), 4.47 (d, J = 4.3 Hz, 1H), 3.94 (d, J = 14.3 Hz, 1H), 3.85 (d, J = 14.3 Hz, 1H) ppm; ^{13}C NMR (125 MHz, $CDCl_3$): δ = 148.9, 140.7, 135.8, 132.4, 131.22, 131.15 (q, J = 32.4 Hz), 131.05, 128.9, 128.8, 128.6, 128.3, 127.7, 126.2 (q, J = 3.7 Hz), 126.1, 123.9 (q, J = 272.3 Hz), 120.4, 77.9, 75.5, 68.4, 60.1 ppm; ^{19}F NMR (470 MHz, $CDCl_3$): δ = –62.72 (s, 3F) ppm; FT-IR (neat): ν = 3036, 2881, 1616, 1455, 1322, 1161, 1122, 1112, 1067, 839, 788, 761, 695 cm^{-1}; HRMS (ESI), *m/z*: [M + Na]$^+$ calcd for $C_{24}H_{18}F_3N_5NaO^+$ 472.1356; found 472.1357.

(3R,3aR,9bR)-2-Benzyl-3-(2-(trifluoromethyl)phenyl)-2,3,3a,9b-tetrahydroisoxazolo[4,5-*c*]isoquinolin-5(4H)-one (**12g**)

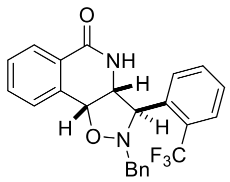

Yield: 82%; white solid; m.p. 158–159 °C; R_f = 0.46 (*n*-hexane/EtOAc 1:1 *v/v*); ^{1}H NMR (500 MHz, DMSO-d_6, 60 °C): δ = 8.08 (d, J = 7.3 Hz, 2H), 8.03 (d, J = 7.7 Hz, 1H), 7.80 – 7.73 (m, 2H), 7.62 (td, J = 7.5, 1.5 Hz, 1H), 7.57 – 7.50 (m, 3H), 7.27 – 7.17 (m, 5H), 5.39 (d, J = 6.8 Hz, 1H), 4.49 (td, J = 6.8, 6.2, 2.4 Hz, 1H), 4.45 (d, J = 4.4 Hz, 2H), 3.86 (s, 2H) ppm; ^{13}C NMR (125 MHz, DMSO-d_6, 60 °C): δ = 161.7, 137.6, 137.0, 135.1, 133.0, 132.6, 130.1, 129.4 (q, J = 31.5 Hz), 129.32, 129.31, 128.63, 128.61, 128.2, 127.4, 127.3, 127.2, 125.9 (q, J = 5.6 Hz), 124.5 (q, J = 274.4 Hz), 73.2, 72.1, 65.1, 61.1 ppm; ^{19}F NMR (470 MHz, DMSO-d_6): δ = –57.04 (s, 3F) ppm; FT-IR (neat): ν = 3193, 3068, 2944, 1675, 1606, 1411, 1311, 1122, 1034, 771, 754, 699 cm^{-1}; HRMS (ESI), *m/z*: [M + K]$^+$ calcd for $C_{24}H_{19}F_3KN_2O_2^+$ 463.1030; found 463.1040.

(4aR,5R,7aR)-6-Benzyl-5-(2-(trifluoromethyl)phenyl)-4a,5,6,7a-tetrahydroisoxazolo[4,5-*c*]tetrazolo[5,1-*a*]isoquinoline (**13g**)

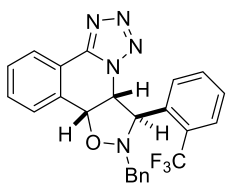

Yield: 14%; white solid; m.p. 178–180 °C; R_f = 0.57 (*n*-hexane/EtOAc 1:1 *v/v*); ^{1}H NMR (500 MHz, $CDCl_3$): δ = 8.33 (d, J = 7.5 Hz, 1H), 8.14 (d, J = 7.9 Hz, 1H), 7.78 (d, J = 7.9 Hz, 1H), 7.74 (t, J = 7.6 Hz, 1H), 7.66 – 7.59 (m, 3H), 7.55 (t, J = 7.7 Hz, 1H), 7.29 – 7.20 (m, 5H), 5.69 (d, J = 6.7 Hz, 1H), 5.42 (dd, J = 6.7, 4.8 Hz, 1H), 4.99 (d, J = 4.7 Hz, 1H), 3.76 (s, 2H) ppm; ^{13}C NMR (125 MHz, $CDCl_3$): δ = 149.0, 136.5, 135.0, 132.6, 132.3, 130.8, 130.6, 130.1, 129.9, 129.6 (q, J = 30.1 Hz), 129.1, 128.7, 128.2, 127.5, 126.6 (q, J = 5.8 Hz), 126.1, 125.0, 120.0, 75.4, 72.7, 68.3, 60.3 ppm; ^{19}F NMR (470 MHz, $CDCl_3$): δ = –57.10 (s, 3F) ppm; FT-IR (neat): ν = 3040, 2916, 1450, 1312, 1283, 1155, 1126, 1035, 970, 786, 727, 695 cm^{-1}; HRMS (ESI), *m/z*: [M + Na]$^+$ calcd for $C_{24}H_{18}F_3N_5NaO^+$ 472.1356; found 472.1364.

(3*R*,3a*R*,9b*R*)-2-Benzyl-3-(2-bromophenyl)-2,3,3a,9b-tetrahydroisoxazolo[4,5-*c*]isoquinolin-5(4*H*)-one (**12h**)

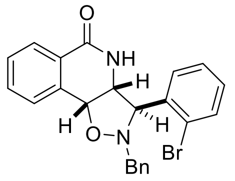

Yield: 87%; white solid; m.p. 150–151 °C; R_f = 0.48 (*n*-hexane/EtOAc 1:1 *v/v*); ^{1}H NMR (500 MHz, DMSO-d_6, 60 °C): δ = 8.55 (br s, 1H), 8.01 (dd, *J* = 7.7, 1.5 Hz, 1H), 7.86 (dd, *J* = 7.8, 1.7 Hz, 1H), 7.64 (dd, *J* = 8.0, 1.2 Hz, 1H), 7.60 (td, *J* = 7.4, 1.5 Hz, 1H), 7.55 (td, *J* = 7.5, 1.5 Hz, 1H), 7.52 – 7.46 (m, 2H), 7.28 (td, *J* = 7.7, 1.7 Hz, 1H), 7.26 – 7.17 (m, 5H), 5.15 (d, *J* = 5.7 Hz, 1H), 4.68 (d, *J* = 3.5 Hz, 1H), 4.47 (ddd, *J* = 5.6, 3.5, 1.7 Hz, 1H), 4.03 (s, 2H) ppm; ^{13}C NMR (125 MHz, DMSO-d_6, 60 °C): δ = 161.9, 137.7, 137.6, 134.3, 132.9, 132.5, 130.1, 129.9, 129.8, 129.5, 128.7, 128.2, 128.1, 127.8, 127.4, 127.2, 123.7, 76.0, 72.6, 64.3, 61.7 ppm; FT-IR (neat): ν = 3176, 3057, 2905, 1668, 1604, 1584, 1406, 1024, 752 cm^{-1}; HRMS (ESI), *m/z*: [M + Na]$^+$ calcd for $C_{23}H_{19}BrN_2NaO_2^+$ 457.0522; found 457.0535.

4a*R*,5*R*,7a*R*)-6-Benzyl-5-(2-bromophenyl)-4a,5,6,7a-tetrahydroisoxazolo[4,5-*c*]tetrazolo[5,1-*a*]isoquinoline (**13h**)

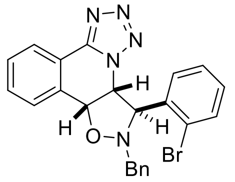

Yield: 12%; white solid; m.p. 174–176 °C; R_f = 0.60 (*n*-hexane/EtOAc 1:1 *v/v*); ^{1}H NMR (500 MHz, $CDCl_3$): δ = 8.34 (d, *J* = 7.4 Hz, 1H), 7.90 (dd, *J* = 7.9, 1.8 Hz, 1H), 7.68 – 7.57 (m, 4H), 7.45 (td, *J* = 7.6, 1.3 Hz, 1H), 7.29 – 7.19 (m, 6H), 5.58 (d, *J* = 6.0 Hz, 1H), 5.38 (dd, *J* = 6.0, 3.7 Hz, 1H), 5.16 (d, *J* = 3.7 Hz, 1H), 3.81 (d, *J* = 13.9 Hz, 1H), 3.78 (d, *J* = 14.1 Hz, 1H) ppm; ^{13}C NMR (125 MHz, $CDCl_3$): δ = 149.1, 136.3, 135.7, 133.7, 132.2, 130.9, 130.4, 130.3, 129.5, 128.8, 128.2, 128.0, 127.5, 126.2, 124.1, 120.4, 75.9, 75.7, 67.50, 60.9 ppm; FT-IR (neat): ν = 3064, 2918, 1490, 1474, 1452, 1308, 1050, 961, 778, 756, 746, 696 cm^{-1}; HRMS (ESI), *m/z*: [M + Na]$^+$ calcd for $C_{23}H_{18}BrN_5NaO^+$ 482.0587; found 482.0576.

(3*R*,3a*R*,9b*R*)-2-Benzyl-3-(2-morpholinophenyl)-2,3,3a,9b-tetrahydroisoxazolo[4,5-*c*]isoquinolin-5(4*H*)-one (**12i**)

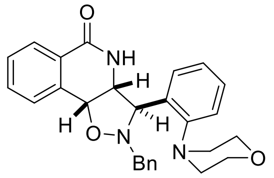

Yield: 84%; white solid; m.p. 140–141 °C; R_f = 0.41 (*n*-hexane/EtOAc 1:1 *v/v*); ^{1}H NMR (500 MHz, DMSO-d_6, 60 °C): δ = 8.09 (br s, 1H), 8.03 (d, *J* = 7.7 Hz, 1H), 7.71 (dd, *J* = 7.7, 1.6 Hz, 1H), 7.62 (td, *J* = 7.5, 1.5 Hz, 1H), 7.56 – 7.51 (m, 2H), 7.36 – 7.29 (m, 2H), 7.27 – 7.17 (m, 6H), 5.27 (d, *J* = 6.5 Hz, 1H), 4.72 (d, *J* = 5.3 Hz, 1H), 4.46 (ddd, *J* = 6.5, 5.3, 2.3 Hz, 1H), 3.94 (d, *J* = 14.3 Hz, 1H), 3.88 (d, *J* = 14.3 Hz, 1H), 3.70 (t, *J* = 4.5 Hz, 4H), 2.86 (dt, *J* = 11.6, 4.5 Hz, 2H), 2.77 (dt, *J* = 11.6, 4.5 Hz, 2H) ppm; ^{13}C NMR (125 MHz, DMSO-d_6, 60 °C): δ = 161.9, 152.3, 138.1, 135.4, 134.5, 132.5, 129.5, 129.2, 128.9, 128.7, 128.4, 128.1, 127.7, 127.3, 127.0, 125.4, 122.2, 72.3, 72.1, 66.8, 64.1, 61.2, 53.6 ppm; FT-IR (neat): ν = 3177, 3032, 2899, 1667, 1584, 1404, 1342, 1113, 931, 771, 755 cm^{-1}; HRMS (ESI), *m/z*: [M + H]$^+$ calcd for $C_{27}H_{28}N_3O_3^+$ 442.2125; found 442.2133.

(4a*R*,5*R*,7a*R*)-6-Benzyl-5-(2-morpholinophenyl)-4a,5,6,7a-tetrahydroisoxazolo[4,5-*c*]tetrazolo[5,1-*a*]isoquinoline (**13i**)

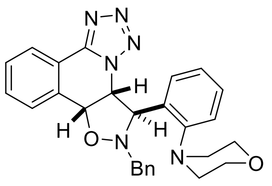

Yield: 8%; white solid; m.p. 168–170 °C; R_f = 0.56 (*n*-hexane/EtOAc 1:1 *v/v*); ^{1}H NMR (500 MHz, $CDCl_3$): δ = 8.34 (d, *J* = 7.4 Hz, 1H), 7.85 (dd, *J* = 8.0, 1.6 Hz, 1H), 7.67 – 7.58 (m, 3H), 7.43 (td, *J* = 7.5, 1.6 Hz, 1H), 7.37 – 7.32 (m, 2H), 7.26 – 7.17 (m, 5H), 5.61 (d, *J* = 5.7 Hz, 1H), 5.38 – 5.34 (m, 2H), 3.89 – 3.77 (m, 5H), 3.69 (d, *J* = 13.8 Hz, 1H), 3.09 – 3.04 (m, 2H), 2.77 (br s, 2H) ppm; ^{13}C NMR (125 MHz, $CDCl_3$): δ = 152.1, 149.1, 136.6, 133.2, 132.2, 130.9, 130.8, 129.8, 129.4, 128.7, 128.2, 127.4, 126.1, 125.9, 122.6, 120.5, 76.0, 71.7, 67.9, 67.3, 60.7, 53.8 ppm; FT-IR (neat): ν = 3063, 2892, 2854, 1491, 1451, 1316, 1223, 1114, 1058, 971, 932, 796, 729, 695 cm^{-1}; HRMS (ESI), *m/z*: [M + H]$^+$ calcd for $C_{27}H_{27}N_6O_2^+$ 467.2190; found 467.2199.

(3*R*,3a*R*,9b*R*)-2-Benzyl-3-(pyridin-3-yl)-2,3,3a,9b-tetrahydroisoxazolo[4,5-*c*]isoquinolin-5(4*H*)-one (**12j**)

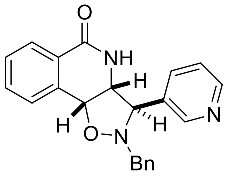

Yield: 64%; off-white solid; m.p. 166–168 °C; R_f = 0.24 (DCM:acetone 3:1 *v/v*); ^{1}H NMR (500 MHz, DMSO-d_6, 60 °C): δ = 8.70 (d, *J* = 2.2 Hz, 1H), 8.51 (dd, *J* = 4.8, 1.7 Hz, 1H), 8.37 (br s, 1H), 8.03 (d, *J* = 7.6 Hz, 1H), 7.93 (dt, *J* = 7.8, 2.0 Hz, 1H), 7.61 (td, *J* = 7.4, 1.5 Hz, 1H), 7.58 – 7.52 (m, 2H), 7.40 (dd, *J* = 7.9, 4.8 Hz, 1H), 7.26 – 7.16 (m, 5H), 5.27 (d, *J* = 6.2 Hz, 1H), 4.47 (ddd, *J* = 6.0, 4.0, 1.7 Hz, 1H), 4.20 (d, *J* = 4.0 Hz, 1H), 3.95 (s, 2H) ppm; ^{13}C NMR (125 MHz, DMSO-d_6, 60 °C): δ = 161.6, 149.6, 149.3, 137.5, 135.8, 134.5, 133.7, 132.5, 130.1, 129.6, 128.9, 128.2, 128.0, 127.4, 127.3, 123.7, 76.3, 72.5, 64.9, 60.9 ppm; FT-IR (neat): ν = 3188, 3061, 2869, 1670, 1418, 1364, 1343, 1028, 763, 733 cm^{-1}; HRMS (ESI), *m/z*: [M + Na]$^+$ calcd for $C_{22}H_{19}N_3NaO_2^+$ 380.1369; found 380.1377.

(3*R*,3a*R*,9b*R*)-2-Benzyl-3-(perfluorophenyl)-2,3,3a,9b-tetrahydroisoxazolo[4,5-*c*]isoquinolin-5(4*H*)-one (**12m**)

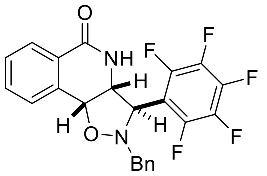

Yield: 81%; white solid; m.p. 182–183 °C; R_f = 0.20 (*n*-hexane/EtOAc 3:1 *v/v*); ^{1}H NMR (500 MHz, DMSO-d_6, 60 °C): δ = 8.36 (br s, 1H), 8.05 (d, *J* = 8.0 Hz, 1H), 7.64 (td, *J* = 7.2, 1.5 Hz, 1H), 7.60 – 7.55 (m, 2H), 7.25 – 7.15 (m, 5H), 5.37 (d, *J* = 6.8 Hz, 1H), 4.77 (ddd, *J* = 7.0, 5.2, 1.9 Hz, 1H), 4.19 (d, *J* = 5.1 Hz, 1H), 4.06 (d, *J* = 13.7 Hz, 1H), 3.90 (d, *J* = 13.6 Hz, 1H) ppm; ^{13}C NMR (125 MHz, DMSO-d_6, 60 °C): δ = 161.2, 136.5, 132.9, 132.5, 130.3, 129.7, 128.8, 128.0, 127.8, 127.4, 127.3, 72.3, 70.9, 61.9, 60.8 ppm; ^{19}F NMR (470 MHz, DMSO-d_6): δ = –139.38 (d, *J* = 14.6 Hz, 2F), –153.11 (t, *J* = 20.9 Hz, 1F), –161.59 (td, *J* = 22.1, 8.0 Hz, 2F) ppm; FT-IR (neat): ν = 3207, 3062, 2956, 1683, 1521, 1504, 1415, 1339, 1131, 1006, 958, 759, 698 cm^{-1}; HRMS (ESI), *m/z*: [M + Na]$^+$ calcd for $C_{23}H_{15}F_5N_2NaO_2^+$ 469.0946; found 469.0947

(4a*R*,5*R*,7a*R*)-6-Benzyl-5-(perfluorophenyl)-4a,5,6,7a-tetrahydroisoxazolo[4,5-*c*]tetrazolo[5,1-*a*]isoquinoline (**13m**)

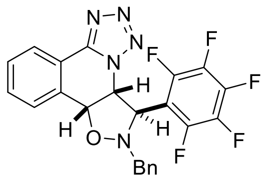

Yield: 12%; white solid; m.p. 175.5–176.5 °C; R_f = 0.33 (*n*–hexane/EtOAc 3:1 *v*/*v*); ^{1}H NMR (500 MHz, $CDCl_3$): δ = 8.34 (dd, *J* = 7.3, 1.8 Hz, 1H), 7.74 – 7.63 (m, 3H), 7.24 – 7.18 (m, 4H), 5.75 (d, *J* = 6.3 Hz, 1H), 5.55 (dd, *J* = 6.3, 4.6 Hz, 1H), 4.58 (d, *J* = 4.6 Hz, 1H), 4.24 (d, *J* = 13.3 Hz, 1H), 3.78 (d, *J* = 13.3 Hz, 1H) ppm; ^{13}C NMR (125 MHz, $CDCl_3$): δ = 148.9, 134.7, 132.5, 131.4, 131.3, 128.7, 128.4, 128.1, 127.9, 126.2, 120.5, 75.5, 69.3, 65.8, 61.0 ppm; ^{19}F NMR (470 MHz, $CDCl_3$): δ = –139.63 (d, *J* = 15.6 Hz, 2F), –151.74 (t, *J* = 20.8 Hz, 1F), –160.75 (td, *J* = 22.1, 21.3, 8.0 Hz, 2F) ppm; FT-IR (neat): ν = 3031, 2913, 1522, 1506, 1455, 1343, 1064, 1012, 959, 948, 755, 710, 699 cm^{-1}; HRMS (ESI), *m*/*z*: $[M + Na]^+$ calcd for $C_{23}H_{14}F_5N_5NaO^+$ 494.1011; found 494.1020.

(3*R*,3a*R*,9b*R*)-2-Benzyl-3-heptyl-2,3,3a,9b-tetrahydroisoxazolo[4,5-*c*]isoquinolin-5(4*H*)-one (**12n**)

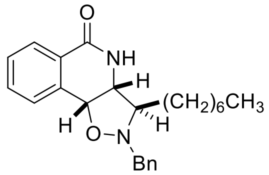

Yield: 90%; white solid; m.p. 119.5–120.5 °C; R_f = 0.54 (*n*-hexane/EtOAc 1:1 *v*/*v*); ^{1}H NMR (500 MHz, DMSO-d_6, 60 °C): δ = 8.12 (br s, 1H), 8.01 (d, *J* = 7.6 Hz, 1H), 7.58 (td, *J* = 7.4, 1.5 Hz, 1H), 7.53 (td, *J* = 7.5, 1.4 Hz, 1H), 7.48 (d, *J* = 7.3 Hz, 1H), 7.31 – 7.21 (m, 5H), 4.99 (d, *J* = 5.4 Hz, 1H), 4.19 (d, *J* = 4.7 Hz, 1H), 4.01 (d, *J* = 13.7 Hz, 1H), 3.93 (d, *J* = 13.7 Hz, 1H), 3.22 (dd, *J* = 6.6, 2.6 Hz, 1H), 1.54 (q, *J* = 7.3 Hz, 2H), 1.42 – 1.21 (m, 10H), 0.87 (t, *J* = 6.9 Hz, 3H) ppm; ^{13}C NMR (125 MHz, DMSO-d_6, 60 °C): δ = 161.8, 138.2, 134.0, 132.2, 130.0, 129.4, 129.0, 128.3, 128.2, 127.3, 127.1, 73.6, 72.6, 62.3, 61.9, 32.5, 31.4, 29.2, 28.7, 25.4, 22.2, 14.0 ppm; FT-IR (neat): ν = 3185, 3034, 2924, 2852, 1667, 1585, 1421, 762, 692 cm^{-1}; HRMS (ESI), *m*/*z*: $[M + Na]^+$ calcd for $C_{24}H_{30}N_2NaO_2^+$ 401.2199; found 401.2190.

(4a*R*,5*R*,7a*R*)-6-Benzyl-5-heptyl-4a,5,6,7a-tetrahydroisoxazolo[4,5-*c*]tetrazolo[5,1-*a*]isoquinoline (**13n**)

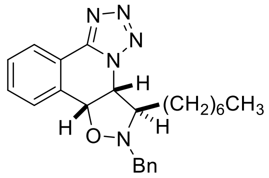

Yield: 9%; white solid; m.p. 126–127 °C; R_f = 0.86 (*n*-hexane/EtOAc 1:1 *v*/*v*); ^{1}H NMR (500 MHz, $CDCl_3$): δ = 8.31 (d, *J* = 7.8 Hz, 1H), 7.65 – 7.56 (m, 3H), 7.33 – 7.24 (m, 5H), 5.34 (d, *J* = 5.4 Hz, 1H), 4.98 (dd, *J* = 5.5, 2.8 Hz, 1H), 3.85 (s, 2H), 3.74 (td, *J* = 6.5, 2.8 Hz, 1H), 1.86 – 1.80 (m, 2H), 1.66 – 1.59 (m, 2H), 1.44 – 1.25 (m, 8H), 0.90 (t, *J* = 6.8 Hz, 3H) ppm; ^{13}C NMR (125 MHz, $CDCl_3$): δ = 149.1, 136.2, 132.1, 131.0, 130.9, 129.4, 129.1, 128.4, 127.6, 126.0, 120.7, 75.5, 73.4, 65.8, 61.7, 32.8, 31.7, 29.5, 29.1, 25.9, 22.6, 14.1 ppm; FT-IR (neat): ν = 3061, 2945, 2923, 2854, 1491, 1455, 1111, 789, 744, 710 cm^{-1}; HRMS (ESI), *m*/*z*: $[M + Na]^+$ calcd for $C_{24}H_{30}N_2NaO_2^+$ 426.2264; found 426.2266.

6. Data for compounds **18**

2-(3-(*p*-Tolyl)isoxazol-5-yl)benzamide (**18b**)

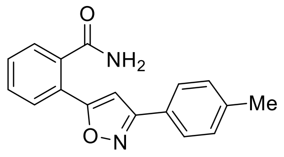

Yield: 89%; white solid; m.p. 176–177 °C; R_f = 0.36 (*n*-hexane/EtOAc 1:2 *v/v*); ^{1}H NMR (500 MHz, DMSO-d_6): δ = 8.00 (br s, 1H), 7.82 – 7.77 (m, 3H), 7.63 – 7.53 (m, 4H), 7.36 (d, *J* = 7.9 Hz, 2H), 7.22 (s, 1H), 2.38 (s, 3H) ppm; ^{13}C NMR (125 MHz, DMSO-d_6): δ = 170.7, 169.6, 162.5, 140.5, 137.4, 130.7, 130.2, 130.0, 128.8, 128.3, 126.9, 126.2, 124.7, 101.0, 21.5 ppm; FT-IR (neat): ν = 3333, 3158, 2920, 1663, 1605, 1427, 1388, 918, 797, 758 cm^{-1}; HRMS (ESI), *m*/*z*: [M + Na]$^+$ calcd for $C_{17}H_{14}N_2NaO_2^+$ 301.0947; found 301.0950.

2-(3-(4-Methoxyphenyl)isoxazol-5-yl)benzamide (**18c**)

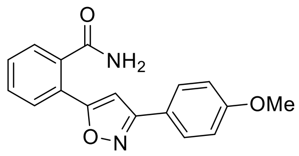

Yield: 87%; white solid; m.p. 175–176 °C; R_f = 0.47 (DCM/acetone 4:1 *v/v*); ^{1}H NMR (500 MHz, DMSO-d_6): δ = 7.98 (br s, 1H), 7.83 (d, *J* = 8.8 Hz, 2H), 7.82 – 7.78 (m, 1H), 7.62 – 7.52 (m, 4H), 7.19 (s, 1H), 7.10 (d, *J* = 8.8 Hz, 2H), 3.83 (s, 3H) ppm; ^{13}C NMR (125 MHz, DMSO-d_6): δ = 170.7, 169.4, 162.2, 161.2, 137.4, 130.7, 130.0, 128.8, 128.5, 128.3, 124.8, 121.4, 115.1, 100.9, 55.8 ppm; FT-IR (neat): ν = 3336, 3164, 1662, 1614, 1433, 1390, 1247, 1026, 832, 799 cm^{-1}; HRMS (ESI), *m*/*z*: [M + Na]$^+$ calcd for $C_{17}H_{14}N_2NaO_3^+$ 317.0897; found 317.0891.

2-(3-(4-(Trifluoromethyl)phenyl)isoxazol-5-yl)benzamide (**18d**)

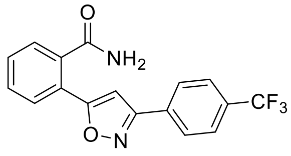

Yield: 86%; white solid; m.p. 178–179 °C; R_f = 0.17 (*n*-hexane/EtOAc 1:1 *v/v*); ^{1}H NMR (500 MHz, DMSO-d_6): δ = 8.13 (d, *J* = 8.1 Hz, 2H), 8.02 (br s, 1H), 7.92 (d, *J* = 8.1 Hz, 2H), 7.83 – 7.80 (m, 1H), 7.64 – 7.55 (m, 4H), 7.38 (s, 1H) ppm; ^{13}C NMR (125 MHz, DMSO-d_6): δ = 170.6, 170.5, 161.7, 137.5, 133.0, 130.99, 130.98 (q, *J* = 32.2 Hz, 1C), 130.2, 129.1, 128.5, 127.9, 126.7 (q, *J* = 4.0 Hz, 1C), 124.6, 124.5 (q, *J* = 272.4 Hz, 1C), 101.5 ppm; ^{19}F NMR (470 MHz, DMSO-d_6) δ = –61.36 (s, 3F) ppm; FT-IR (neat): ν = 3377, 3193, 1652, 1441, 1327, 1167, 1122, 1065, 952, 769 cm^{-1}; HRMS (ESI), *m*/*z*: [M + Na]$^+$ calcd for $C_{17}H_{11}F_3N_2NaO_2^+$ 355.0665; found 355.0666.

2-(3-(4-Fluorophenyl)isoxazol-5-yl)benzamide (**18e**)

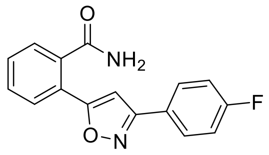

Yield: 88%; white solid; m.p. 175–176 °C; R_f = 0.46 (DCM/acetone 4:1 *v/v*); ^{1}H NMR (500 MHz, DMSO-d_6): δ = 8.00 (br s, 1H), 7.98 – 7.93 (m, 2H), 7.82 – 7.78 (m, 1H), 7.63 – 7.53 (m, 4H), 7.42 – 7.36 (m, 2H), 7.26 (s, 1H) ppm; ^{13}C NMR (125 MHz, DMSO-d_6): δ = 170.6, 170.0, 163.7 (d, *J* = 247.6 Hz, 1C), 161.7, 137.5, 130.8, 130.1, 129.4 (d, *J* = 8.6 Hz, 1C), 128.9, 128.4, 125.6 (d, *J* = 3.2 Hz, 1C), 124.7,

116.7 (d, J = 21.9 Hz, 1C), 101.1 ppm; ^{19}F NMR (470 MHz, DMSO-d_6) δ = –110.59 (ddd, J = 14.2, 8.9, 5.4 Hz, 1F) ppm; FT-IR (neat): ν = 3372, 3181, 1651, 1607, 1438, 1396, 1231, 1159, 950, 784 cm^{-1}; HRMS (ESI), m/z: [M + Na]$^+$ calcd for $C_{16}H_{11}FN_2NaO_2^+$ 305.0697; found 305.0690.

2-(3-(4-(tert-Butyl)phenyl)isoxazol-5-yl)benzamide (**18f**)

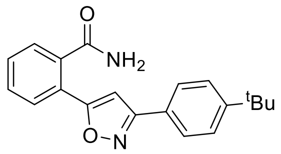

Yield: 91%; white solid; m.p. 165–166 °C; R_f = 0.54 (DCM/acetone 4:1 v/v); ^{1}H NMR (500 MHz, DMSO-d_6): δ = 8.00 (br s, 1H), 7.84 – 7.80 (m, 3H), 7.62 – 7.53 (m, 6H), 7.22 (s, 1H), 1.32 (s, 9H) ppm; ^{13}C NMR (125 MHz, DMSO-d_6): δ = 170.7, 169.6, 162.5, 153.4, 137.4, 130.7, 130.0, 128.8, 128.3, 126.8, 126.4, 126.3, 124.7, 101.1, 35.1, 31.4 ppm; FT-IR (neat): ν = 3334, 3162, 2952, 1666, 1626, 1430, 1389, 952, 839, 759 cm^{-1}; HRMS (ESI), m/z: [M + K]$^+$ calcd for $C_{16}H_{11}FKN_2O_2^+$ 359.1156; found 359.1163.

2-(3-(2-Bromophenyl)isoxazol-5-yl)benzamide (**18g**)

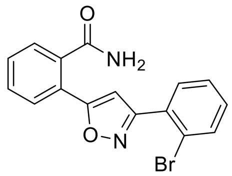

Yiel: 92%; white solid; m.p. 174–175 °C; R_f = 0.18 (n-hexane/EtOAc 1:1 v/v); ^{1}H NMR (500 MHz, DMSO-d_6): δ = 8.02 (br s, 1H), 7.88 – 7.86 (m, 1H), 7.84 (dd, J = 8.0, 1.2 Hz, 1H), 7.69 (dd, J = 7.6, 1.7 Hz, 1H), 7.62 (br s, 1H), 7.61 – 7.58 (m, 2H), 7.58 – 7.51 (m, 2H), 7.48 (td, J = 7.7, 1.8 Hz, 1H), 7.16 (s, 1H) ppm; ^{13}C NMR (125 MHz, DMSO-d_6): δ = 170.8, 168.5, 162.7, 137.5, 134.1, 132.3, 131.8, 130.8, 130.3, 130.0, 128.7, 128.5, 128.2, 124.1, 122.0, 104.2 ppm; FT-IR (neat): ν = 3373, 3189, 1634, 1605, 1451, 1440, 1395, 952, 760 cm^{-1}; HRMS (ESI), m/z: [M + Na]$^+$ calcd for $C_{16}H_{11}BrN_2NaO_2^+$ 364.9896; found 364.9890.

2-(3-(2-(Trifluoromethyl)phenyl)isoxazol-5-yl)benzamide (**18h**)

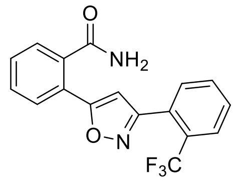

Yield: 89%; white solid; m.p. 153–154 °C; R_f = 0.18 (n-hexane/EtOAc 1:1 v/v); ^{1}H NMR (500 MHz, DMSO-d_6): δ = 8.02 (br s, 1H), 7.96 (d, J = 7.8 Hz, 1H), 7.90 – 7.82 (m, 2H), 7.78 (t, J = 7.6 Hz, 1H), 7.73 (d, J = 7.5 Hz, 1H), 7.64 – 7.57 (m, 3H), 7.54 – 7.51 (m, 1H), 7.00 (s, 1H) ppm; ^{13}C NMR (125 MHz, DMSO-d_6): δ = 170.8, 168.8, 161.8, 137.5, 133.4, 132.3, 131.0, 130.9, 130.0, 128.6, 128.3, 128.0 (q, J = 2.4 Hz, 1C), 127.9 (q, J = 30.7 Hz, 1C), 127.2 (q, J = 5.4 Hz, 1C), 124.2 (q, J = 273.7 Hz, 1C), 123.9, 104.2 (q, J = 2.0 Hz, 1C) ppm; ^{19}F NMR (470 MHz, DMSO-d_6) δ = –56.86 (s, 3F) ppm; FT-IR (neat): ν = 3362, 3186, 1651, 1392, 1309, 1138, 1114, 954, 772 cm^{-1}; HRMS (ESI), m/z: [M + Na]$^+$ calcd for $C_{17}H_{11}F_3N_2NaO_2^+$ 355.0665; found 355.0669.

2-(3-(3-Nitrophenyl)isoxazol-5-yl)benzamide (**18i**)

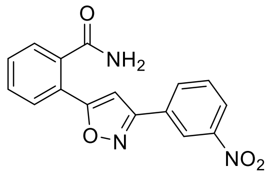

Yield: 83%; white solid; m.p. 247–248 °C; R_f = 0.31 (*n*-hexane/EtOAc 1:2 *v/v*); ^{1}H NMR (500 MHz, DMSO-d_6): δ = 8.66 (br s, 1H), 8.37 (t, *J* = 7.8 Hz, 2H), 8.02 (br s, 1H), 7.89 – 7.80 (m, 2H), 7.64 – 7.56 (m, 4H), 7.48 (s, 1H) ppm; ^{13}C NMR (125 MHz, DMSO-d_6): δ = 170.7, 170.5, 161.1, 148.9, 137.5, 133.3, 131.5, 131.0, 130.6, 130.2, 129.1, 128.4, 125.4, 124.5, 121.5, 101.4 ppm; FT-IR (neat): ν = 3353, 3171, 2363, 2342, 1660, 1542, 1347, 809, 763, 745 cm^{-1}; HRMS (ESI), *m*/*z*: [M + Na]$^+$ calcd for $C_{16}H_{11}N_3NaO_4^+$ 332.0642; found 332.0652.

2-(3-Mesitylisoxazol-5-yl)benzamide (**18j**)

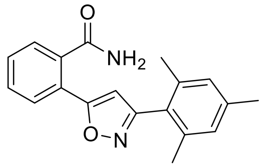

Yield: 90%; white solid; m.p. 177–178 °C; R_f = 0.28 (*n*-hexane/EtOAc 1:1 *v/v*); ^{1}H NMR (500 MHz, CDCl$_3$): δ = 7.86 (dd, *J* = 7.6, 1.7 Hz, 1H), 7.55 (td, *J* = 7.8, 1.4 Hz, 2H), 7.49 (td, *J* = 7.7, 7.2, 1.3 Hz, 1H), 6.94 (s, 2H), 6.58 (s, 1H), 6.16 (br s, 1H), 5.94 (br s, 1H), 2.32 (s, 3H), 2.17 (s, 6H) ppm; ^{13}C NMR (125 MHz, CDCl$_3$): δ = 171.2, 167.4, 162.7, 138.9, 137.2, 135.0, 130.3, 130.1, 128.7, 128.4, 127.8, 125.9, 124.9, 104.8, 21.2, 20.3 ppm; FT-IR (neat): ν = 3348, 3179, 1658, 1581, 1571, 1487, 1377, 841, 774 cm^{-1}; HRMS (ESI), *m*/*z*: [M + Na]$^+$ calcd for $C_{19}H_{18}N_2NaO_2^+$ 329.1260; found 329.1253.

2-(3-(Perfluorophenyl)isoxazol-5-yl)benzamide (**18k**)

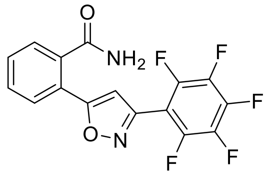

Yield: 84%; white solid; m.p. 218–219 °C; R_f = 0.28 (*n*-hexane/EtOAc 1:1 *v/v*); ^{1}H NMR (500 MHz, DMSO-d_6): δ = 8.05 (br s, 1H), 7.89 – 7.85 (m, 1H), 7.64 (br s, 1H), 7.63 – 7.60 (m, 2H), 7.59 – 7.55 (m, 1H), 7.16 (s, 1H) ppm; ^{13}C NMR (125 MHz, DMSO-d_6): δ = 170.6, 170.0, 152.0, 137.5, 131.2, 130.2, 128.9, 128.4, 123.7, 104.1 (t, *J* = 3.3 Hz, 1C) ppm; ^{19}F NMR (470 MHz, DMSO-d_6) δ = –139.21 (dd, *J* = 22.7, 6.7 Hz, 2F), –151.85 (t, *J* = 22.2 Hz, 1F), –161.56 (td, *J* = 21.7, 5.6 Hz, 2F) ppm; FT-IR (neat): ν = 3359, 3176, 1653, 1534, 1506, 1400, 1092, 997, 815, 764 cm^{-1}; HRMS (ESI), *m*/*z*: [M + Na]$^+$ calcd for $C_{16}H_7F_5N_2NaO_2^+$ 377.0320; found 377.0329.

2-(3-(2,6-Dichlorophenyl)isoxazol-5-yl)benzamide (**18l**)

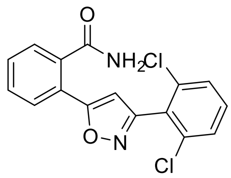

Yield: 93%; white solid; m.p. 197–198 °C; R_f = 0.34 (*n*-hexane/EtOAc 2:3 *v/v*); ^{1}H NMR (500 MHz, DMSO-d_6): δ = 8.02 (br s, 1H), 7.91 – 7.87 (m, 1H), 7.67 (d, *J* = 7.8 Hz, 2H), 7.63 – 7.57 (m, 4H), 7.54 – 7.51 (m, 1H), 6.97 (s, 1H) ppm; ^{13}C NMR (125 MHz, DMSO-d_6): δ = 170.8, 169.2, 159.3, 137.5, 134.9, 132.9, 130.9, 130.0, 129.2, 128.5, 128.3, 128.1, 123.8, 104.5 ppm; FT-IR (neat): ν = 3356, 3169, 1648, 1625, 1433, 1406, 1384, 1193, 1124, 780 cm^{-1}; HRMS (ESI), *m*/*z*: [M + K]$^+$ calcd for $C_{16}H_{10}Cl_2KN_2O_2^+$ 370.9751; found 370.9759.

2-(3-(5-Chlorofuran-2-yl)isoxazol-5-yl)benzamide (**18m**)

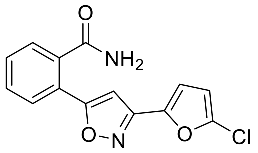

Yield: 80%; white solid; m.p. 172–173 °C; R_f = 0.4 (*n*-hexane/EtOAc 1:2 *v/v*); ^{1}H NMR (500 MHz, DMSO-d_6): δ = 8.00 (br s, 1H), 7.82 – 7.78 (m, 1H), 7.62 – 7.57 (m, 3H), 7.56 – 7.52 (m, 1H), 7.26 (d, *J* = 3.5 Hz, 1H), 7.09 (s, 1H), 6.76 (d, *J* = 3.5 Hz, 1H) ppm; ^{13}C NMR (125 MHz, DMSO-d_6): δ = 170.6, 169.7, 154.2, 143.8, 137.7, 137.5, 131.0, 130.1, 128.9, 128.4, 124.1, 114.3, 109.9, 100.3 ppm; FT-IR (neat): ν = 3380, 3187, 1645, 1620, 1522, 1429, 1397, 1205, 1014, 766 cm^{-1}; HRMS (ESI), *m/z*: $[M + Na]^+$ calcd for $C_{14}H_9ClN_2NaO_3^+$ 311.0194; found 311.0199.

2-(3-(Thiophen-2-yl)isoxazol-5-yl)benzamide (**18n**)

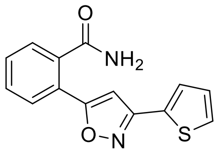

Yield: 79%; white solid; m.p. 171.5–172.5 °C; R_f = 0.34 (*n*-hexane/EtOAc 1:2 *v/v*); ^{1}H NMR (500 MHz, DMSO-d_6): δ = 8.00 (br s, 1H), 7.81 – 7.77 (m, 1H), 7.76 (d, *J* = 4.9 Hz, 1H), 7.71 (d, *J* = 3.4 Hz, 1H), 7.64 – 7.53 (m, 4H), 7.24 (dd, *J* = 5.1, 3.6 Hz, 1H), 7.19 (s, 1H) ppm; ^{13}C NMR (125 MHz, DMSO-d_6): δ = 170.6, 169.7, 158.1, 137.5, 130.9, 130.4, 130.1, 129.2, 129.0, 128.7, 128.4, 124.5, 101.1 ppm; FT-IR (neat): ν = 3370, 3200, 1625, 1602, 1582, 1428, 1394, 914, 762, 706 cm^{-1}; HRMS (ESI), *m/z*: $[M + Na]^+$ calcd for $C_{14}H_{10}N_2NaO_2S^+$ 293.0355; found 293.0354.

2-(3-Heptylisoxazol-5-yl)benzamide (**18o**)

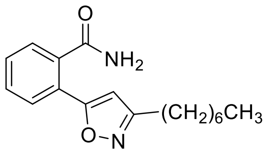

Yield: 92%; white solid; m.p. 105–106 °C; R_f = 0.46 (*n*-hexane/EtOAc 2:3 *v/v*); ^{1}H NMR (500 MHz, DMSO-d_6): δ = 7.93 (br s, 1H), 7.77 – 7.73 (m, 1H), 7.58 – 7.51 (m, 3H), 7.49 – 7.45 (m, 1H), 6.64 (s, 1H), 2.64 (t, *J* = 7.6 Hz, 2H), 1.63 (p, *J* = 7.3 Hz, 2H), 1.37 – 1.21 (m, 8H), 0.86 (t, *J* = 6.8 Hz, 3H) ppm; ^{13}C NMR (125 MHz, DMSO-d_6): δ = 170.9, 168.1, 164.5, 137.4, 130.4, 129.8, 128.5, 128.1, 124.6, 102.7, 31.7, 29.0, 28.9, 28.2, 25.8, 22.6, 14.4 ppm; FT-IR (neat): ν = 3363, 3182, 2920, 2850, 1652, 1602, 1463, 1396, 797, 767 cm^{-1}; HRMS (ESI), *m/z*: $[M + Na]^+$ calcd for $C_{17}H_{22}N_2NaO_2^+$ 309.1573; found 309.1563.

7. Data for compounds **22**

2-(4-Iodo-5-phenylisoxazol-3-yl)benzamide (**22a**)

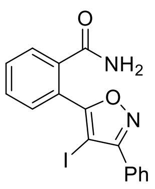

Yield: 97%; white solid; m.p. 177.5–179 °C; R_f = 0.2 (*n*-hexane/EtOAc 1:1 *v/v*); ^{1}H NMR (500 MHz, DMSO-d_6): δ = 8.02 (br s, 1H), 7.81 – 7.78 (m, 2H), 7.76 – 7.73 (m, 1H), 7.71 – 7.64 (m, 3H), 7.62 – 7.56 (m, 3H), 7.46 (br s, 1H) ppm; ^{13}C NMR (125 MHz, DMSO-d_6): δ = 171.9, 169.3, 163.4, 137.9, 131.3, 131.2, 130.7, 130.6, 129.3, 129.1, 128.9,

128.8, 126.2, 61.3 ppm; FT-IR (neat): ν = 3466, 3144, 1673, 1604, 1379, 982, 766, 699, 515 cm^{-1}; HRMS (ESI), *m*/*z*: $[M + Na]^+$ calcd for $C_{16}H_{11}IN_2NaO_2^+$ 412.9757; found 412.9750.

2-(4-Iodo-5-(p-tolyl)isoxazol-3-yl)benzamide (**22b**)

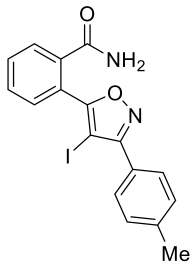

Yield: 99%; white solid; m.p. 184–185 °C; R_f = 0.44 (*n*-hexane/EtOAc 1:2 *v/v*); ^1H NMR (500 MHz, $CDCl_3$): δ = 7.78 – 7.71 (m, 3H), 7.65 (d, *J* = 6.9 Hz, 1H), 7.58 (dtd, *J* = 17.0, 7.4, 1.6 Hz, 2H), 7.30 (d, *J* = 7.8 Hz, 2H), 6.13 (br s, 1H), 5.93 (br s, 1H), 2.42 (s, 3H) ppm; ^{13}C NMR (125 MHz, $CDCl_3$): δ = 170.3, 169.6, 163.4, 140.3, 136.1, 131.04, 130.9, 130.6, 129.2, 128.6, 128.5, 125.7, 125.4, 59.7, 21.4 ppm; FT-IR (neat): ν = 3468, 3153, 1678, 1604, 1379, 1110, 982, 820, 767, 495 cm^{-1}; HRMS (ESI), *m*/*z*: $[M + Na]^+$ calcd for $C_{17}H_{13}IN_2NaO_2^+$ 426.9914; found 426.9907.

2-(4-Iodo-5-(3-iodo-4-methoxyphenyl)isoxazol-3-yl)benzamide (**22c**)

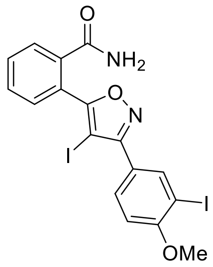

Yield: 88%; white solid; m.p. 195–196 °C; R_f = 0.31 (*n*-hexane/EtOAc 1:1 *v/v*); ^1H NMR (500 MHz, DMSO-*d*6): δ = 8.18 (d, *J* = 2.2 Hz, 1H), 8.02 (br s, 1H), 7.84 (dd, *J* = 8.5, 2.2 Hz, 1H), 7.76 – 7.71 (m, 1H), 7.68 – 7.63 (m, 3H), 7.46 (br s, 1H), 7.21 (d, *J* = 8.6 Hz, 1H), 3.92 (s, 3H) ppm; ^{13}C NMR (125 MHz, DMSO-*d*6): δ = 172.0, 169.3, 161.6, 159.7, 139.1, 137.9, 131.4, 131.2, 130.6, 130.5, 128.8, 126.2, 123.1, 112.2, 86.7, 61.3, 57.2 ppm; FT-IR (neat): ν = 34061, 3208, 2930, 1652, 1600, 1397, 1274, 1258, 1046, 987, 728 cm^{-1}; HRMS (ESI), *m*/*z*: $[M + Na]^+$ calcd for $C_{17}H_{12}I_2N_2NaO_3^+$ 568.8829; found 568.8840.

2-(4-Iodo-5-(4-(trifluoromethyl)phenyl)isoxazol-3-yl)benzamide (**22d**)

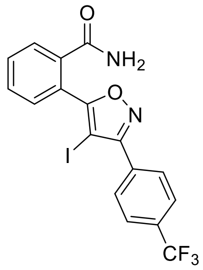

Yield: 99%; white solid; m.p. 174–175 °C; R_f = 0.46 (*n*-hexane/EtOAc 1:2 *v/v*); ^1H NMR (500 MHz, DMSO-*d*6): δ = 8.07 – 8.02 (m, 3H), 7.98 (d, *J* = 8.2 Hz, 2H), 7.78 – 7.74 (m, 1H), 7.71 – 7.64 (m, 3H), 7.48 (br s, 1H) ppm; ^{13}C NMR (125 MHz, DMSO-*d*6): δ = 172.5, 169.2, 162.5, 137.9, 133.1, 131.4, 131.3, 130.9 (q, *J* = 32.3 Hz, 1C), 130.7, 129.9, 128.8, 126.3 (q, *J* = 3.6 Hz, 1C), 126.0, 124.5 (q, *J* = 272.4 Hz, 1C), 61.3 ppm; ^{19}F NMR (470 MHz, DMSO-*d*6) δ = –61.36 (s, 3F) ppm; FT-IR (neat): ν = 3469, 3279, 3167, 1675, 1640, 1600, 1319, 1127, 1110, 1067, 766 cm^{-1}; HRMS (ESI), *m*/*z*: $[M + H]^+$ calcd for $C_{17}H_{11}F_3IN_2O_2^+$ 458.9812; found 458.9821.

2-(5-(4-Fluorophenyl)-4-iodoisoxazol-3-yl)benzamide (**22e**)

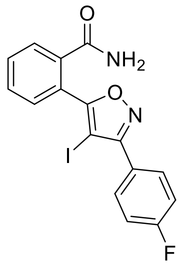

Yield: 97%; white solid; m.p. 151–152 °C; R_f = 0.48 (*n*-hexane/EtOAc 1:2 *v/v*); ^{1}H NMR (500 MHz, DMSO-*d*$_6$): δ = 8.03 (br s, 1H), 7.88 – 7.84 (m, 2H), 7.76 – 7.72 (m, 1H), 7.70 – 7.64 (m, 3H), 7.49 – 7.41 (m, 3H) ppm; ^{13}C NMR (125 MHz, DMSO-*d*$_6$): δ = 172.0, 169.2, 163.6 (d, *J* = 247.9 Hz, 1C), 162.6, 137.9, 131.3 (2C), 131.2, 131.2, 130.5, 128.7, 126.1, 125.5 (d, *J* = 3.3 Hz, 1C), 116.4 (d, *J* = 21.9 Hz, 1C), 61.3 ppm; ^{19}F NMR (470 MHz, DMSO-*d*$_6$) δ = –110.57 (tt, *J* = 9.2, 5.2 Hz, 1F) ppm; FT-IR (neat): ν = 3359, 3151, 1678, 1604, 1482, 1399, 1216, 986, 841, 766 cm^{-1}; HRMS (ESI), *m*/*z*: [M + Na]$^+$ calcd for $C_{16}H_{10}FIN_2NaO_2^+$ 430.9663; found 430.9669.

2-(5-(4-(tert-Butyl)phenyl)-4-iodoisoxazol-3-yl)benzamide (**22f**)

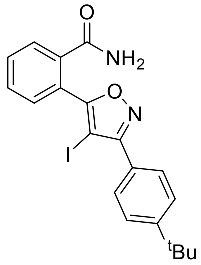

Yield: 97%; white solid; m.p. 215–216 °C; R_f = 0.58 (*n*-hexane/EtOAc 1:2 *v/v*); ^{1}H NMR (500 MHz, DMSO-*d*$_6$): δ = 8.02 (br s, 1H), 7.75 – 7.72 (m, 3H), 7.70 – 7.63 (m, 3H), 7.61 (d, *J* = 8.3 Hz, 2H), 7.47 (br s, 1H), 1.34 (s, 9H) ppm; ^{13}C NMR (125 MHz, DMSO-*d*$_6$): δ = 171.7, 169.3, 163.2, 153.27, 137.9, 131.3, 131.1, 130.5, 128.7, 126.2, 126.2, 126.1, 61.2, 35.1, 31.5 ppm; FT-IR (neat): ν = 3417, 3198, 2961, 1671, 1611, 1590, 1375, 984, 842, 767 cm^{-1}; HRMS (ESI), *m*/*z*: [M + Na]$^+$ calcd for $C_{20}H_{19}IN_2NaO_2^+$ 469.0383; found 469.0392.

2-(5-(2-Bromophenyl)-4-iodoisoxazol-3-yl)benzamide (**22g**)

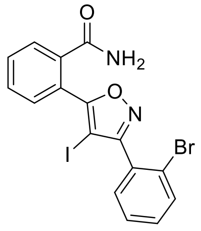

Yield: 99%; white solid; m.p. 224–225 °C; R_f = 0.34 (*n*-hexane/EtOAc 1:2 *v/v*); ^{1}H NMR (500 MHz, DMSO-*d*$_6$): δ = 7.99 (br s, 1H), 7.85 (d, *J* = 7.8 Hz, 1H), 7.75 – 7.71 (m, 1H), 7.71 – 7.63 (m, 3H), 7.58 (t, *J* = 7.4 Hz, 1H), 7.52 (td, *J* = 7.7, 1.9 Hz, 1H), 7.49 (br s, 1H), 7.45 (dd, *J* = 7.4, 1.9 Hz, 1H) ppm; ^{13}C NMR (125 MHz, DMSO-*d*$_6$): δ = 170.8, 169.3, 165.6, 138.0, 133.4, 132.5, 132.3, 131.2, 131.0, 130.7, 130.5, 128.8, 128.4, 125.7, 123.2, 64.1 ppm; FT-IR (neat): ν = 3470, 3313, 3175, 1682, 1662, 1599, 1473, 1379, 982, 760 cm^{-1}; HRMS (ESI), *m*/*z*: [M + Na]$^+$ calcd for $C_{16}H_{10}BrIN_2NaO_2^+$ 490.8863; found 490.8860.

2-(4-Iodo-5-(2-(trifluoromethyl)phenyl)isoxazol-3-yl)benzamide (**22h**)

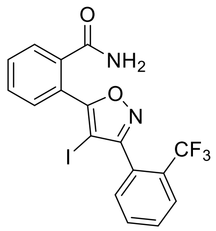

Yield: 99%; white solid; m.p. 190–191 °C; R_f = 0.37 (*n*-hexane/EtOAc 1:2 *v/v*); ^{1}H NMR (500 MHz, DMSO-*d*$_6$): δ = 8.00 – 7.95 (m, 2H), 7.88 (t, *J* = 7.5 Hz, 1H), 7.81 (t, *J* = 7.7 Hz, 1H), 7.75 – 7.72 (m, 1H), 7.70 – 7.65 (m, 3H), 7.54 (d, *J* = 7.7 Hz, 1H), 7.50 (br s, 1H) ppm; ^{13}C NMR (125 MHz, DMSO-*d*$_6$): δ = 170.7, 169.3, 164.2, 138.0, 133.1, 132.6, 131.2, 131.2, 131.0, 130.5, 128.9 (d, *J* = 30.6 Hz, 1C), 128.8, 127.8 (q, *J* = 1.7 Hz, 1C), 127.2 (q, *J* = 4.8 Hz, 1C), 125.7, 124.0 (q, *J* = 274.1 Hz, 1C), 64.1 ppm; ^{19}F NMR (470 MHz, DMSO-*d*$_6$) δ = –57.17 (s, 3F) ppm; FT-IR (neat): ν = 3440, 3115,

1681, 1656, 1610, 1396, 1316, 1174, 1127, 1032, 766 cm^{-1}; HRMS (ESI), *m*/*z*: [M + Na]$^+$ calcd for $C_{17}H_{10}F_3IN_2NaO_2^+$ 480.9631; found 480.9627.

2-(4-Iodo-5-(3-nitrophenyl)isoxazol-3-yl)benzamide (**22i**)

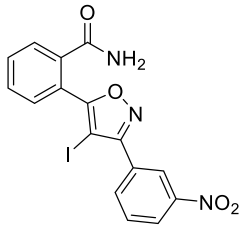

Yield: 99%; white solid; m.p. 222–223 °C; R_f = 0.46 (*n*-hexane/EtOAc 1:2 *v*/*v*); ^{1}H NMR (500 MHz, DMSO-d_6): δ = 8.65 (t, *J* = 2.0 Hz, 1H), 8.45 (ddd, *J* = 8.3, 2.4, 1.1 Hz, 1H), 8.28 (dt, *J* = 7.7, 1.2 Hz, 1H), 8.05 (br s, 1H), 7.92 (t, *J* = 8.0 Hz, 1H), 7.79 – 7.75 (m, 1H), 7.72 – 7.66 (m, 3H), 7.48 (br s, 1H) ppm; ^{13}C NMR (125 MHz, DMSO-d_6): δ = 172.7, 169.1, 161.6, 148.3, 137.8, 135.2, 131.3, 131.3, 131.3, 130.6, 130.5, 128.8, 125.9, 125.5, 123.3, 61.3 ppm; FT-IR (neat): ν = 3458, 3297, 3176, 1660, 1603, 1525, 1386, 1347, 997, 765 cm^{-1}; HRMS (ESI), *m*/*z*: [M + Na]$^+$ calcd for $C_{16}H_{10}IN_3NaO_4^+$ 457.9608; found 457.9603.

2-(4-Iodo-5-(perfluorophenyl)isoxazol-3-yl)benzamide (**22k**)

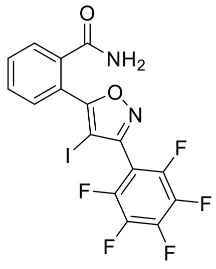

Yield: 99%; white solid; m.p. 178–179 °C; R_f = 0.5 (*n*-hexane/EtOAc 1:2 *v*/*v*); ^{1}H NMR (500 MHz, DMSO-d_6): δ = 8.07 (br s, 1H), 7.79 – 7.75 (m, 1H), 7.73 – 7.67 (m, 3H), 7.53 (br s, 1H) ppm; ^{13}C NMR (125 MHz, DMSO-d_6): δ = 172.8, 169.1, 155.4, 137.8, 131.5, 131.2, 130.7, 128.8, 125.3, 63.9 ppm; ^{19}F NMR (470 MHz, DMSO-d_6) δ = –138.55 – –138.65 (m, 2F), –149.48 (t, *J* = 22.4 Hz, 1F), –160.55 – –160.71 (m, 2F) ppm; FT-IR (neat): ν = 3476, 3200, 1673, 1506, 1494, 1372, 1126, 1021, 990 cm^{-1}; HRMS (ESI), *m*/*z*: [M + Na]$^+$ calcd for $C_{16}H_6F_5IN_2NaO_2^+$ 502.9286; found 502.9271.

2-(5-(2,6-Dichlorophenyl)-4-iodoisoxazol-3-yl)benzamide (**22l**)

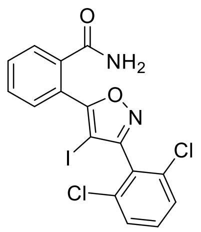

Yield: 98%; white solid; m.p. 264–265 °C; R_f = 0.48 (*n*-hexane/EtOAc 1:2 *v*/*v*); ^{1}H NMR (500 MHz, DMSO-d_6): δ = 7.98 (br s, 1H), 7.76 – 7.61 (m, 7H), 7.51 (br s, 1H) ppm; ^{13}C NMR (125 MHz, DMSO-d_6): δ = 171.4, 169.3, 162.9, 138.0, 135.3, 133.4, 131.3, 130.9, 130.5, 129.0, 128.8, 127.9, 125.5, 63.8 ppm; FT-IR (neat): ν = 3432, 3111, 1680, 1661, 1605, 1429, 1369, 1193, 1112, 985 cm^{-1}; HRMS (ESI), *m*/*z*: [M + Na]$^+$ calcd for $C_{16}H_9Cl_2IN_2NaO_2^+$ 480.8978; found 480.8977.

2-(5-Heptyl-4-iodoisoxazol-3-yl)benzamide (**22o**)

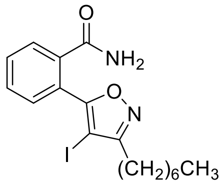

Yield: 98%; white solid; m.p. 84–85 °C; R_f = 0.54 (*n*-hexane/EtOAc 1:2 *v/v*); ^{1}H NMR (500 MHz, $CDCl_3$): δ = 7.79 – 7.73 (m, 1H), 7.62 – 7.54 (m, 3H), 5.85 (br s, 1H), 5.76 (br s, 1H), 2.67 (t, *J* = 7.5 Hz, 2H), 1.76 (p, *J* = 7.6 Hz, 2H), 1.46 – 1.26 (m, 8H), 0.89 (t, *J* = 6.7 Hz, 3H) ppm; ^{13}C NMR (125 MHz, $CDCl_3$): δ = 169.5, 168.8, 165.3, 135.9, 130.7, 130.6, 128.6, 125.6, 61.4, 31.7, 28.9, 27.2, 26.9, 22.6, 14.1 ppm; FT-IR (neat): ν = 3306, 3140, 2926, 2855, 1687, 1667, 1607, 1385, 1047 cm^{-1}; HRMS (ESI), *m*/*z*: $[M + Na]^+$ calcd for $C_{17}H_{21}IN_2NaO_2^+$ 435.0540; found 435.0550.

8. Data for compounds **23**

3-Phenylisoxazolo[4,5-*c*]isoquinolin-5(4*H*)-one (**23a**)

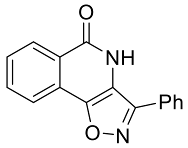

Yield: 78%; white solid; m.p. >250 °C (decomposed); R_f = 0.46 (DCM/acetone 8:1 *v/v*); ^{1}H NMR (500 MHz, DMSO-d_6): δ = 11.88 (br s, 1H), 8.35 (dd, *J* = 8.2, 2.4 Hz, 1H), 8.13 – 8.07 (m, 1H), 7.99 – 7.88 (m, 3H), 7.76 (td, *J* = 7.6, 2.3 Hz, 1H), 7.61 – 7.54 (m, 3H) ppm; ^{13}C NMR (125 MHz, DMSO-d_6): δ = 162.1, 152.7, 150.1, 138.2, 134.0, 130.9, 130.5, 130.0, 129.4, 129.1, 128.6, 127.0, 125.1, 121.3 ppm; FT-IR (neat): ν = 3441, 2924, 1664, 1598, 1346, 769, 689 cm^{-1}; HRMS (ESI), *m*/*z*: $[M + Na]^+$ calcd for $C_{16}H_{10}N_2NaO_2^+$ 285.0634; found 285.0639

3-(*p*-Tolyl)isoxazolo[4,5-*c*]isoquinolin-5(4*H*)-one (**23b**)

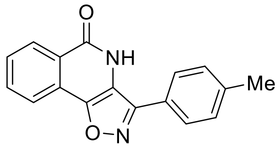

Yield: 67%; white solid; m.p. >250 °C (decomposed); R_f = 0.64 (*n*-hexane/EtOAc 1:1 *v/v*); ^{1}H NMR (500 MHz, 4% TFA-*d* in $CDCl_3$): δ = 8.55 (d, *J* = 8.1 Hz, 1H), 8.19 (d, *J* = 7.9 Hz, 1H), 7.99 (t, *J* = 7.6 Hz, 1H), 7.82 – 7.73 (m, 3H), 7.40 (d, *J* = 7.6 Hz, 2H), 2.47 (s, 3H) ppm; ^{13}C NMR (125 MHz, 4% TFA-*d* in $CDCl_3$): δ = 164.4, 152.5, 151.4, 141.7, 134.9, 130.3, 130.2, 129.4, 127.6, 125.7, 124.0, 122.8, 121.5, 117.4, 21.5 ppm; FT-IR (neat): ν = 3094, 2982, 1646, 1596, 1481, 1339, 821, 766, 725 cm^{-1}; HRMS (ESI), *m*/*z*: $[M + K]^+$ calcd for $C_{17}H_{12}KN_2O_2^+$ 315.0530; found 315.0533.

3-(3-Iodo-4-methoxyphenyl)isoxazolo[4,5-*c*]isoquinolin-5(4*H*)-one (**23c**)

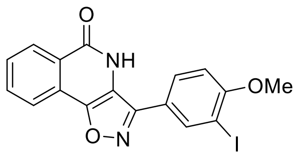

Yield: 93%; white solid; m.p. >260 °C (decomposed); R_f = 0.4 (*n*-hexane/EtOAc 1:1 *v/v*); ^{1}H NMR (500 MHz, 4% TFA-*d* in $CDCl_3$): δ = 8.56 (d, *J* = 8.2 Hz, 1H), 8.26 (d, *J* = 2.1 Hz, 1H), 8.21 (d, *J* = 8.0 Hz, 1H), 8.01 (t, *J* = 7.6 Hz, 1H), 7.86 – 7.78 (m, 2H), 7.01 (d, *J* = 8.5 Hz, 1H), 3.99 (s, 3H) ppm; ^{13}C NMR (125 MHz, 4% TFA-*d* in $CDCl_3$): δ = 164.5, 160.5, 151.6, 151.0, 138.5, 135.0, 130.4, 129.4, 129.4, 125.6, 121.6, 119.7, 117.2, 111.2, 87.1, 56.6 ppm; FT-IR (neat): ν = 3086, 2845, 1652,

1599, 1480, 1393, 1197, 1154, 1044, 779 cm^{-1}; HRMS (ESI), *m/z*: [M + Na]$^+$ calcd for $C_{17}H_{11}IN_2NaO_3^+$ 440.9707; found 440.9716.

3-(4-(Trifluoromethyl)phenyl)isoxazolo[4,5-*c*]isoquinolin-5(4*H*)-one (**23d**)

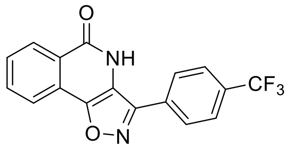

Yield: 81%; white solid; m.p. >255 °C (decomposed); R_f = 0.51 (*n*-hexane/EtOAc 2:1 *v/v*); ^{1}H NMR (500 MHz, 4% TFA-*d* in $CDCl_3$): δ = 8.57 (d, *J* = 8.1 Hz, 1H), 8.24 (d, *J* = 7.9 Hz, 1H), 8.06 – 7.99 (m, 3H), 7.90 – 7.82 (m, 3H) ppm; ^{13}C NMR (125 MHz, 4% TFA-*d* in $CDCl_3$): δ = 164.6, 152.2, 151.4, 135.2, 133.3 (q, *J* = 33.0 Hz, 1C), 130.7, 129.5, 129.1, 128.3, 126.6 (q, *J* = 3.7 Hz, 1C), 125.4, 124.0, 123.6 (q, *J* = 272.5 Hz, 1C), 121.7, 117.3 ppm; ^{19}F NMR (470 MHz, 4% TFA-*d* in $CDCl_3$) δ = –63.18 (s, 3F) ppm; FT-IR (neat): ν = 3086, 1652, 1598, 1327, 1198, 1134, 1111, 1070, 841, 770 cm^{-1}; HRMS (ESI), *m/z*: [M + Na]$^+$ calcd for $C_{17}H_9F_3N_2NaO_2^+$ 353.0508; found 353.0517.

3-(4-Fluorophenyl)isoxazolo[4,5-*c*]isoquinolin-5(4*H*)-one (**23e**)

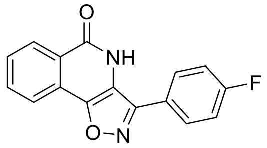

Yield: 89%; white solid; m.p. >270 °C (decomposed); R_f = 0.66 (*n*-hexane/EtOAc 1:1 *v/v*); ^{1}H NMR (500 MHz, 4% TFA-*d* in $CDCl_3$): δ = 8.56 (d, *J* = 8.1 Hz, 1H), 8.23 (d, *J* = 8.0 Hz, 1H), 8.02 (t, *J* = 7.6 Hz, 1H), 7.90 – 7.81 (m, 3H), 7.31 (t, *J* = 8.5 Hz, 2H) ppm; ^{13}C NMR (125 MHz, 4% TFA-*d* in $CDCl_3$): δ = 164.7 (d, *J* = 253.1 Hz, 1C), 164.6, 151.8, 151.8, 135.2, 130.6, 130.0 (d, *J* = 8.7 Hz, 1C), 129.5, 125.5, 124.1, 121.7, 121.5 (d, *J* = 3.4 Hz, 1C), 117.3, 117.0 (d, *J* = 22.0 Hz, 1C) ppm; ^{19}F NMR (470 MHz, 4% TFA-*d* in $CDCl_3$) δ = –107.45 (tt, *J* = 8.9, 5.0 Hz, 1F) ppm; FT-IR (neat): ν = 3090, 2981, 1662, 1600, 1485, 1420, 1344, 1235, 828, 760 cm^{-1}; HRMS (ESI), *m/z*: [M + Na]$^+$ calcd for $C_{16}H_9FN_2NaO_2^+$ 303.0540; found 303.0533.

3-(4-(*tert*-Butyl)phenyl)isoxazolo[4,5-*c*]isoquinolin-5(4*H*)-one (**23f**)

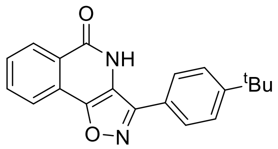

Yield: 78%; white solid; m.p. >265 °C (decomposed); R_f = 0.44 (*n*-hexane/EtOAc 2:1 *v/v*); ^{1}H NMR (500 MHz, 4% TFA-*d* in $CDCl_3$): δ = 8.57 (d, *J* = 8.2 Hz, 1H), 8.22 (d, *J* = 8.0 Hz, 1H), 8.00 (t, *J* = 7.6 Hz, 1H), 7.80 (t, *J* = 7.3 Hz, 3H), 7.64 (d, *J* = 7.9 Hz, 2H), 1.40 (s, 9H) ppm; ^{13}C NMR (125 MHz, 4% TFA-*d* in $CDCl_3$): δ = 164.4, 154.8, 152.5, 151.4, 134.9, 130.2, 129.5, 127.5, 126.6, 125.7, 124.0, 122.8, 121.6, 117.6, 35.0, 31.1 ppm; FT-IR (neat): ν = 3113, 2965, 1645, 1598, 1340, 1134, 834, 769 cm^{-1}; HRMS (ESI), *m/z*: [M + Na]$^+$ calcd for $C_{20}H_{18}N_2NaO_2^+$ 341.1260; found 341.1266.

3-(2-Bromophenyl)isoxazolo[4,5-*c*]isoquinolin-5(4*H*)-one (**23g**)

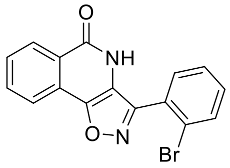

Yield: 58%; white solid; m.p. >240 °C (decomposed); R_f = 0.46 (*n*-hexane/EtOAc 1:1 *v/v*); ^{1}H NMR (500 MHz, 4% TFA-*d* in $CDCl_3$): δ = 8.50 (d, *J* = 8.2 Hz, 1H), 8.21 (d, *J* = 8.0 Hz, 1H), 8.02 – 7.94 (m, 1H), 7.78 (q, *J* = 8.0, 7.4 Hz, 2H), 7.65 – 7.57 (m, 1H), 7.50 (dtd, *J* = 21.4, 7.5, 1.6 Hz, 2H) ppm; ^{13}C NMR (125 MHz, 4% TFA-*d* in $CDCl_3$): δ = 163.6, 152.9, 150.9, 134.5, 133.6, 132.3, 132.1, 129.9, 129.5, 128.0, 127.8, 127.4, 125.7, 122.8, 121.5, 118.8 ppm; FT-IR (neat): ν = 3077, 2971, 1652, 1596, 1339, 855, 760, 730, 700 cm^{-1}; HRMS (ESI), *m*/*z*: [M + Na]$^+$ calcd for $C_{16}H_9BrN_2NaO_2^+$ 362.9740; found 362.9745.

3-(2-(Trifluoromethyl)phenyl)isoxazolo[4,5-*c*]isoquinolin-5(4*H*)-one (**23h**)

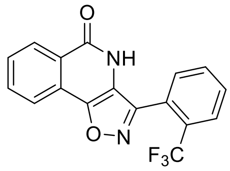

Yield: 61%; white solid; m.p. 238–239 °C; R_f = 0.47 (*n*-hexane/EtOAc 1:1 *v/v*); ^{1}H NMR (500 MHz, DMSO-*d*$_6$): δ = 11.91 (br s, 1H), 8.35 (d, *J* = 8.0 Hz, 1H), 8.14 (d, *J* = 7.8 Hz, 1H), 7.99 – 7.95 (m, 2H), 7.90 – 7.71 (m, 4H) ppm; ^{13}C NMR (125 MHz, DMSO-*d*$_6$): δ = 161.7, 151.7, 148.9, 134.2, 133.1, 133.1, 131.3, 130.2, 129.3, 129.2 (q, *J* = 30.6 Hz, 1C) 126.9 (q, *J* = 4.9 Hz, 1C), 126.2, 125.1 (q, *J* = 4.1 Hz, 1C), 125.1, 124.1 (q, *J* = 273.7 Hz, 1C), 121.5, 121.4 ppm; ^{19}F NMR (470 MHz, 4% TFA-*d* in $CDCl_3$) δ = –57.79 (s, 3F) ppm; FT-IR (neat): ν = 3080, 2981, 1645, 1595, 1341, 1311, 1123, 1068, 1037, 766 cm^{-1}; HRMS (ESI), *m*/*z*: [M + Na]$^+$ calcd for $C_{17}H_9F_3N_2NaO_2^+$ 353.0508; found 353.0517.

3-(Perfluorophenyl)isoxazolo[4,5-*c*]isoquinolin-5(4*H*)-one (**23k**)

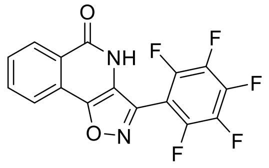

Yield: 29%; white solid; m.p. >260 °C (decomposed); R_f = 0.47 (*n*-hexane/EtOAc 3:1 *v/v*); ^{1}H NMR (500 MHz, 4% TFA-*d* in $CDCl_3$): δ = 8.58 (d, *J* = 8.1 Hz, 1H), 8.27 (d, *J* = 7.9 Hz, 1H), 8.05 (t, *J* = 7.6 Hz, 1H), 7.86 (t, *J* = 7.7 Hz, 1H) ppm; ^{13}C NMR (125 MHz, 4% TFA-*d* in $CDCl_3$): δ = 164.5, 152.1, 141.5, 135.1, 130.7, 129.6, 125.3, 124.1, 121.6, 118.6 ppm; ^{19}F NMR (470 MHz, 4% TFA-*d* in $CDCl_3$) δ = –137.51 – –137.62 (m, 2F), –147.82 (t, *J* = 20.8 Hz, 1F), –159.30 – –159.44 (m, 2F) ppm; FT-IR (neat): ν = 3087, 1649, 1600, 1474, 1342, 1180, 899 cm^{-1}; HRMS (ESI), *m*/*z*: [M + Na]$^+$ calcd for $C_{16}H_5F_5N_2NaO_2^+$ 375.0163; found 375.0163.

3-(2,6-Dichlorophenyl)isoxazolo[4,5-*c*]isoquinolin-5(4*H*)-one (**23l**)

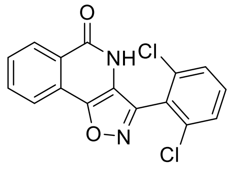

Yield: 46%; white solid; m.p. >270 °C (decomposed); R_f = 0.47 (*n*-hexane/EtOAc 1:1 *v/v*); ^{1}H NMR (500 MHz, 4% TFA-*d* in $CDCl_3$): δ = 8.55 (d, *J* = 8.1 Hz, 1H), 8.26 (d, *J* = 8.0 Hz, 1H), 8.01 (t, *J* = 7.7 Hz, 1H), 7.81 (t, *J* = 7.9 Hz, 1H), 7.58 – 7.47 (m, 3H) ppm; ^{13}C NMR (125 MHz, 4% TFA-*d* in $CDCl_3$): δ = 164.3, 151.3, 149.1, 135.9, 134.8, 132.6, 130.2, 129.5, 128.7, 125.7, 124.2, 124.0, 121.6, 118.9

ppm; FT-IR (neat): ν = 2914, 1652, 1602, 1434, 1339, 1196, 894, 783, 772 cm^{-1}; HRMS (ESI), *m*/*z*: [M + Na]$^+$ calcd for $C_{16}H_8Cl_2N_2NaO_2^+$ 352.9855; found 352.9863.

3-Heptylisoxazolo[4,5-*c*]isoquinolin-5(4*H*)-one (**23o**)

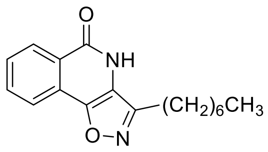

Yield: 80%; white solid; m.p. 177–178 °C; R_f = 0.46 (*n*-hexane/EtOAc 2:1 *v/v*); ^1H NMR (500 MHz, DMSO-d_6): δ = 11.94 (br s, 1H), 8.32 (dd, *J* = 8.2, 1.3 Hz, 1H), 8.01 (d, *J* = 7.9 Hz, 1H), 7.90 (td, *J* = 7.6, 1.3 Hz, 1H), 7.72 (t, *J* = 7.2 Hz, 1H), 2.84 (t, *J* = 7.6 Hz, 2H), 1.67 (p, *J* = 7.4 Hz, 2H), 1.37 – 1.21 (m, 8H), 0.84 (t, *J* = 6.8 Hz, 3H) ppm; ^{13}C NMR (125 MHz, DMSO-d_6): δ = 161.8, 154.1, 148.4, 133.9, 129.6, 129.3, 126.0, 125.4, 121.2, 120.5, 31.6, 29.0, 28.8, 27.7, 24.1, 22.5, 14.4 ppm; FT-IR (neat): ν = 2952, 2924, 2850, 1662, 1602, 1424, 1335, 1241, 859, 764 cm^{-1}; HRMS (ESI), *m*/*z*: [M + Na]$^+$ calcd for $C_{17}H_{20}N_2NaO_2^+$ 307.1417; found 307.1409.

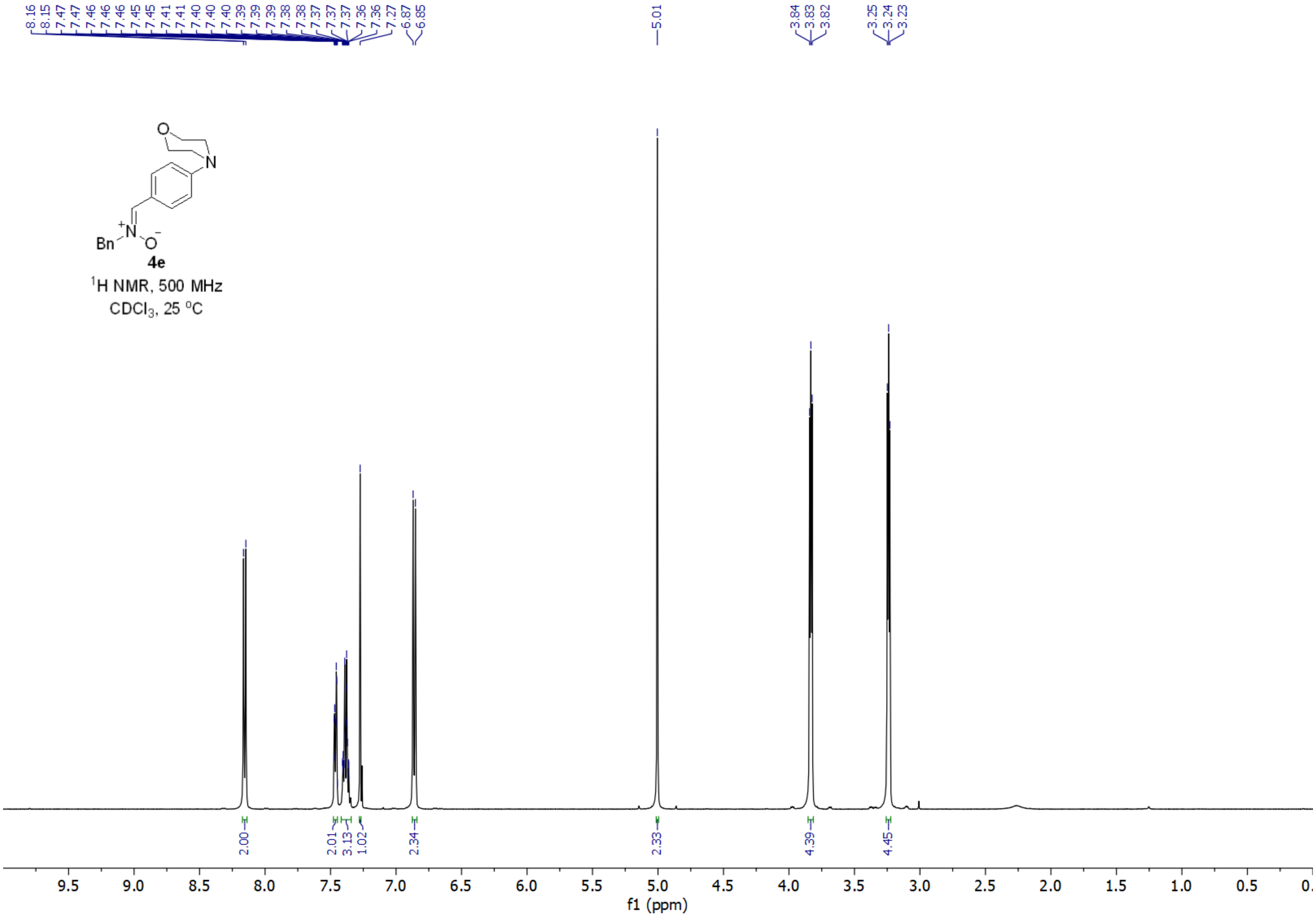
8.16
8.15
7.47
7.47
7.46
7.46
7.45
7.45
7.41
7.40
7.40
7.40
7.39
7.39
7.39
7.38
7.38
7.37
7.37
7.37
7.36
7.36
7.27
6.87
6.85
5.01
3.84
3.83
3.82
3.25
3.24
3.23
O
N
+
N
O
−
Bn
4e
1H NMR, 500 MHz
CDCl3, 25 °C
2.00
2.01
3.13
1.02
2.34
2.33
4.39
4.45
9.5
9.0
8.5
8.0
7.5
7.0
6.5
6.0
5.5
5.0
4.5
4.0
3.5
3.0
2.5
2.0
1.5
1.0
0.5
0.
f1 (ppm)

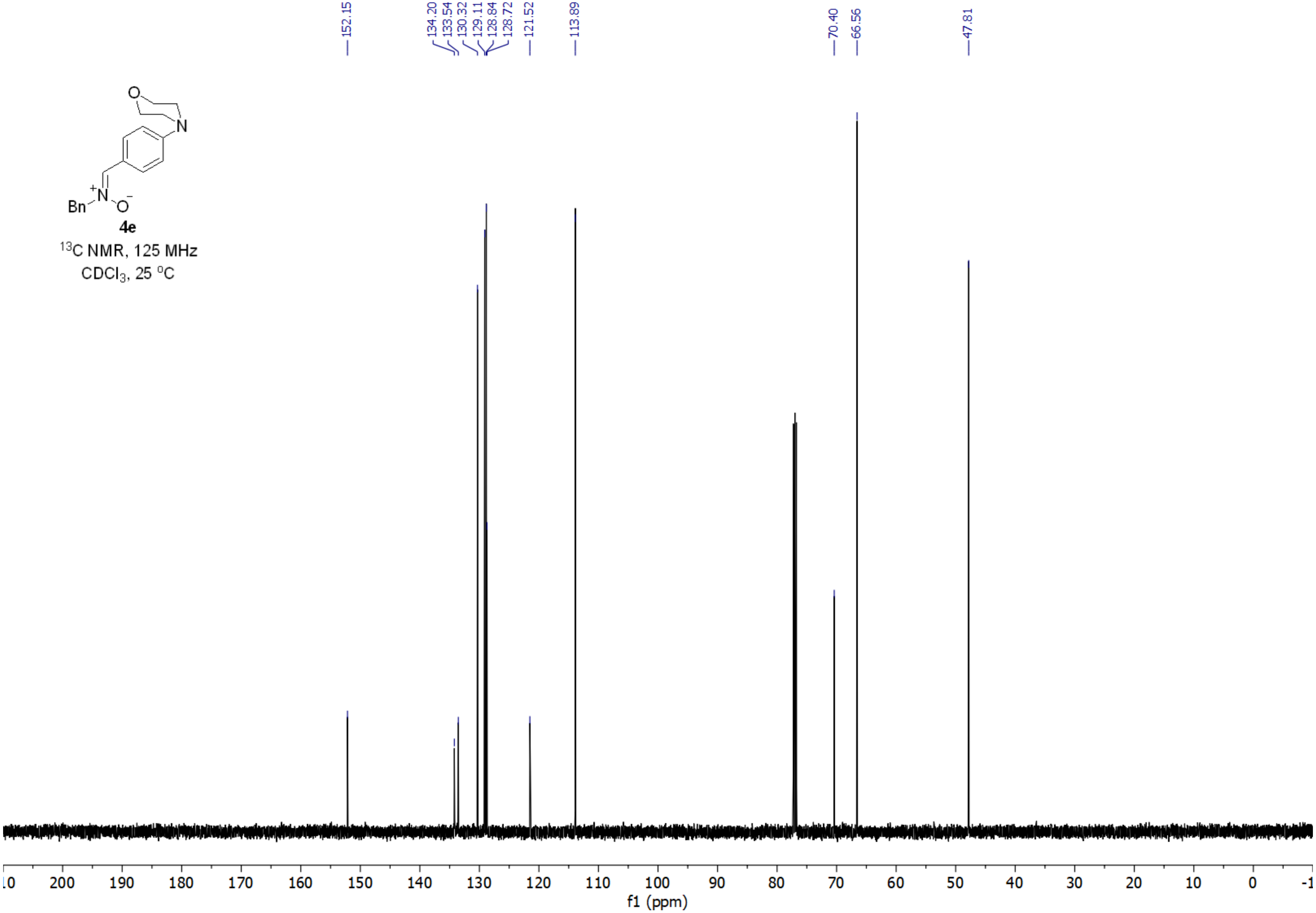
152.15
134.20
133.54
130.32
129.11
128.84
128.72
121.52
113.89
70.40
66.56
47.81
O
N
Bn
N
O
4e
13C NMR, 125 MHz
CDCl3, 25 °C
210 200 190 180 170 160 150 140 130 120 110 100 90 80 70 60 50 40 30 20 10 0 -10
f1 (ppm)

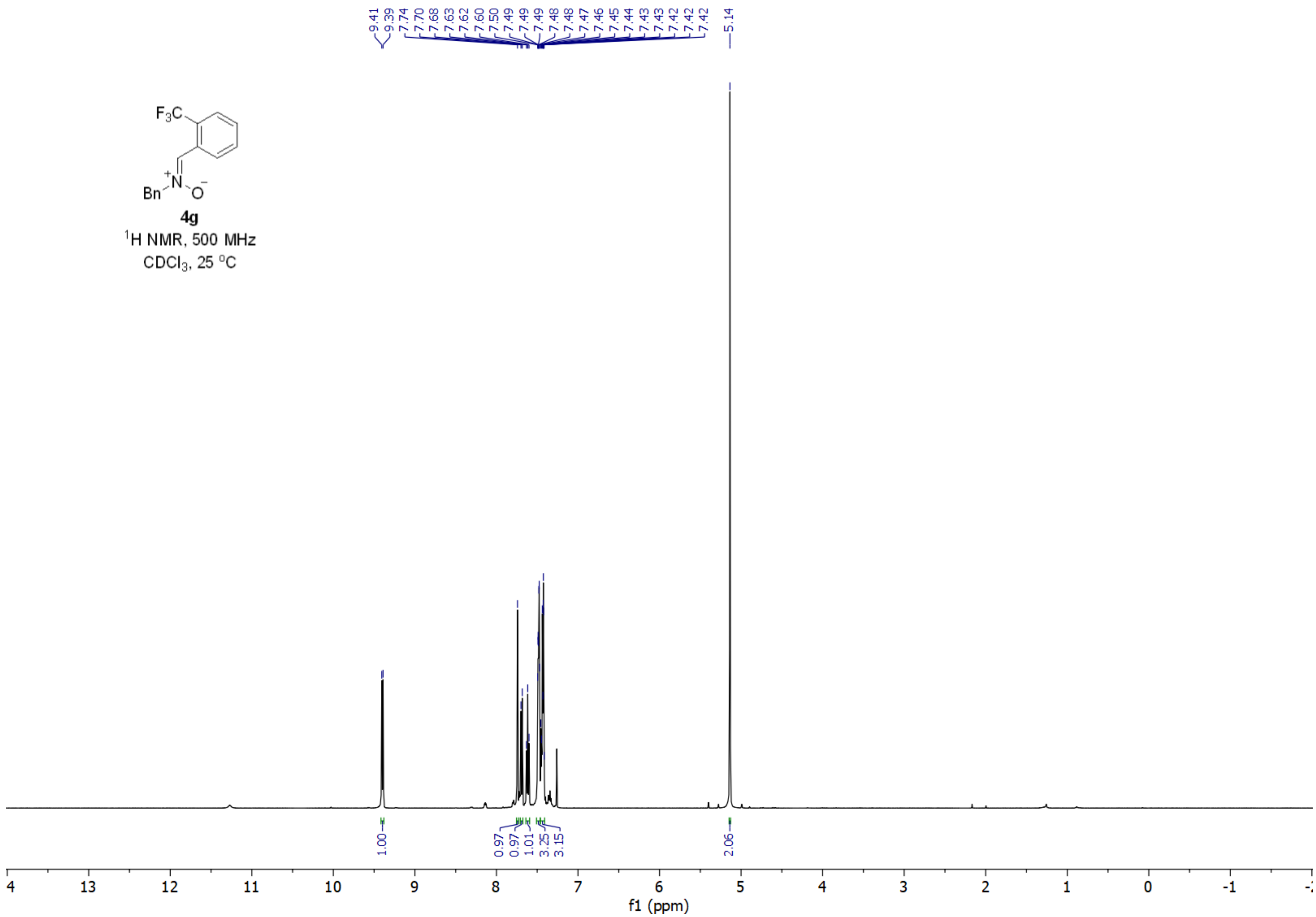

9.41
9.39
7.74
7.70
7.68
7.63
7.62
7.60
7.50
7.49
7.49
7.49
7.48
7.48
7.47
7.46
7.45
7.44
7.43
7.43
7.42
7.42
7.42
5.14
F3C
Bn
N
O
4g
1H NMR, 500 MHz
CDCl3, 25 oC
1.00
0.97
0.97
1.01
3.25
3.15
2.06
14
13
12
11
10
9
8
7
6
5
4
3
2
1
0
-1
-2
f1 (ppm)

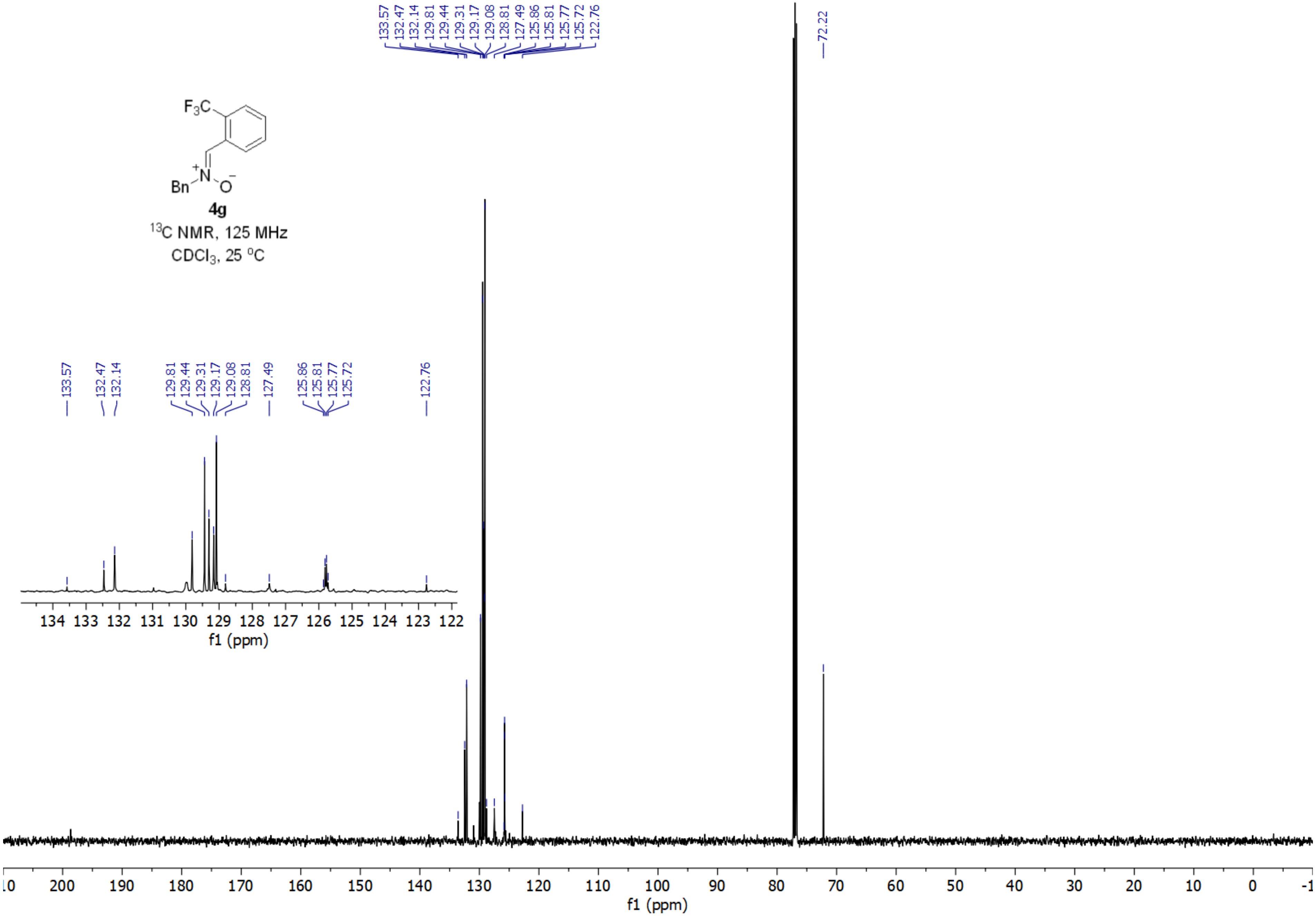
F3C
Bn
N
O
4g
13C NMR, 125 MHz
CDCl3, 25 °C
133.57
132.47
132.14
129.81
129.44
129.31
129.17
129.08
128.81
127.49
125.86
125.81
125.77
125.72
122.76
72.22
f1 (ppm)

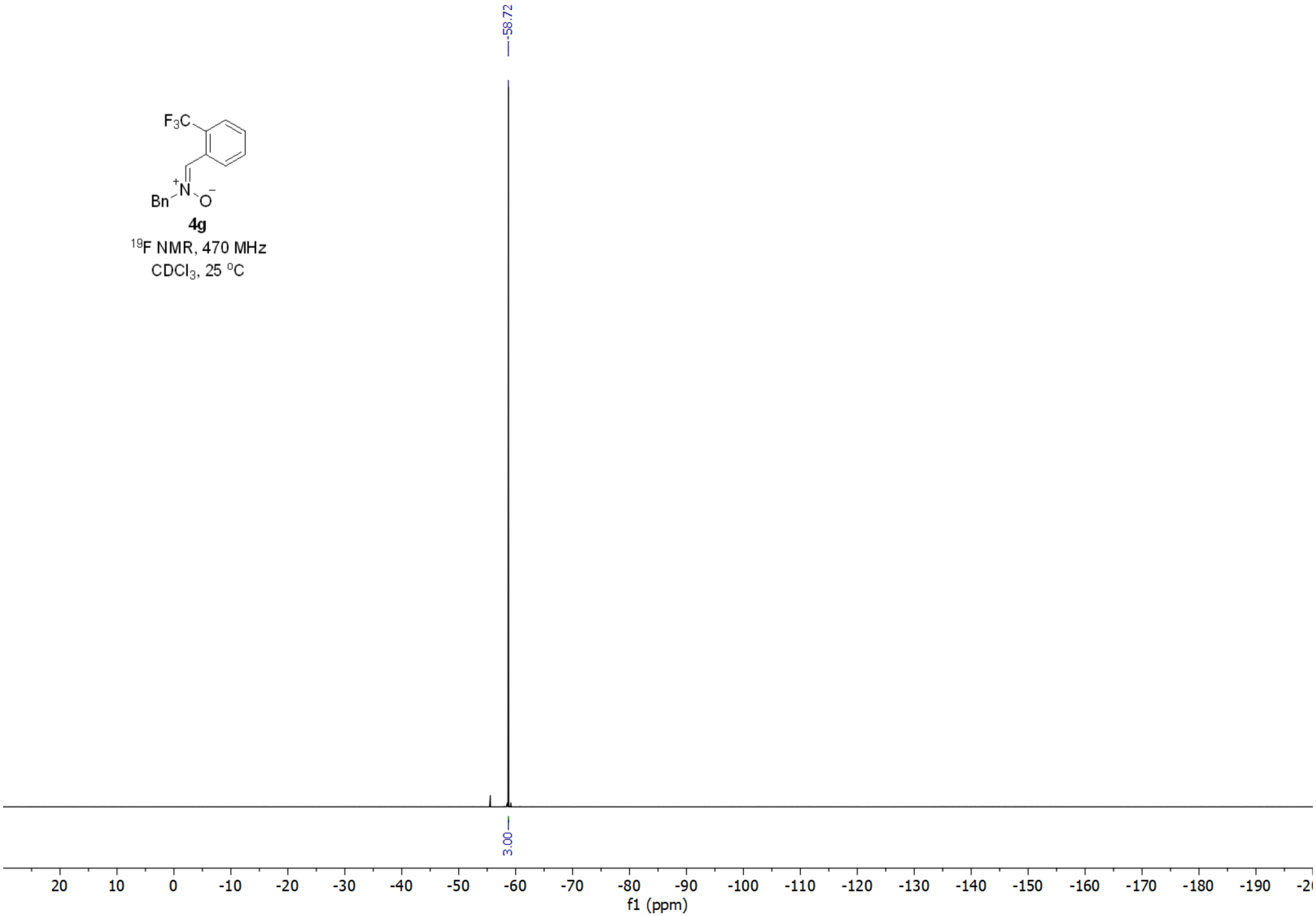

F3C
Bn
N
O
4g
19F NMR, 470 MHz
CDCl3, 25 °C
-58.72
3.00
20
10
0
-10
-20
-30
-40
-50
-60
-70
-80
-90
-100
-110
-120
-130
-140
-150
-160
-170
-180
-190
f1 (ppm)

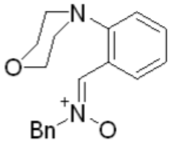

4i

^{1}H NMR, 500 MHz

$CDCl_3$, 25 °C

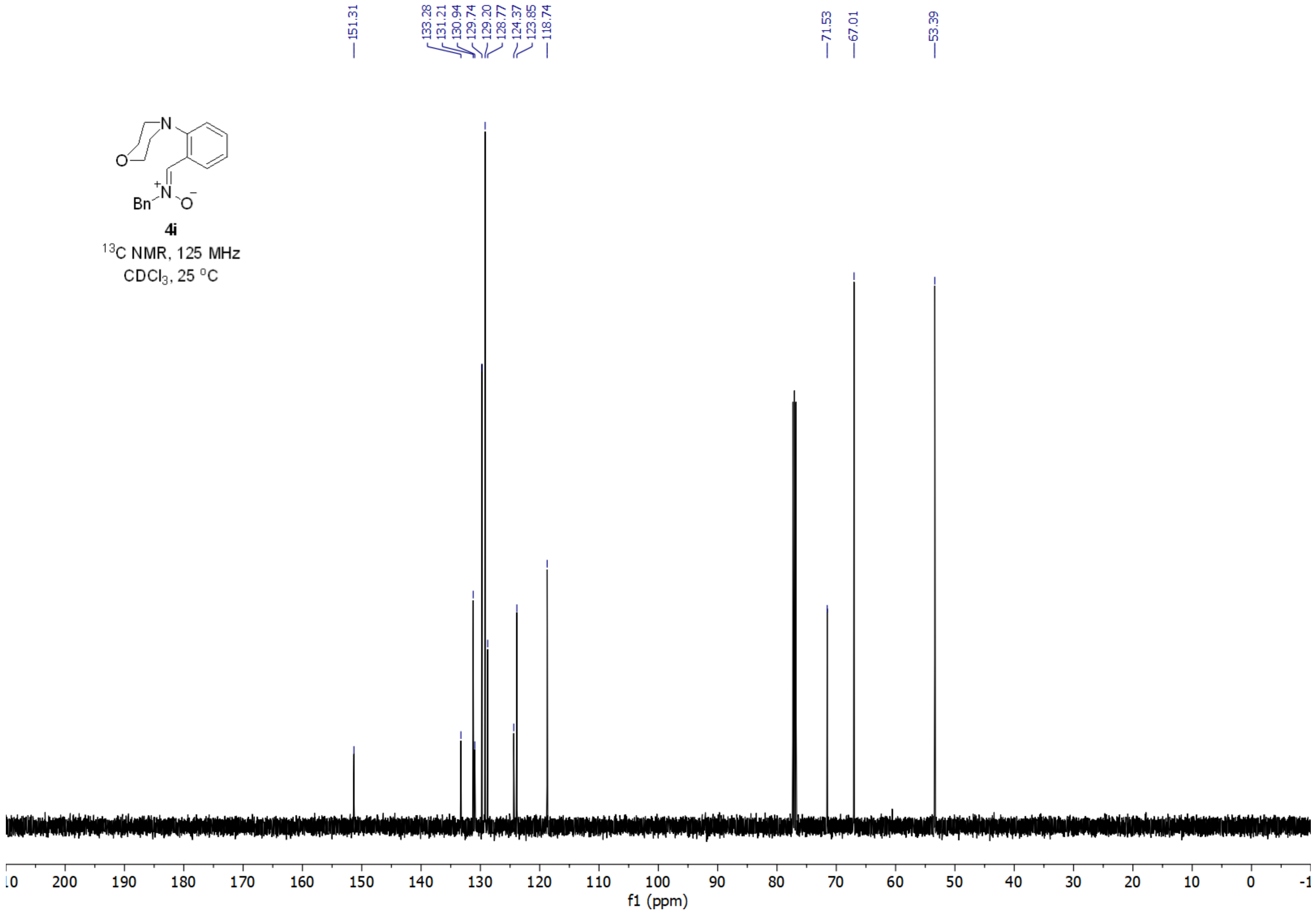

151.31
133.28
131.21
130.94
129.74
129.20
128.77
124.37
123.85
118.74
71.53
67.01
53.39
4i
13C NMR, 125 MHz
CDCl3, 25 °C
f1 (ppm)

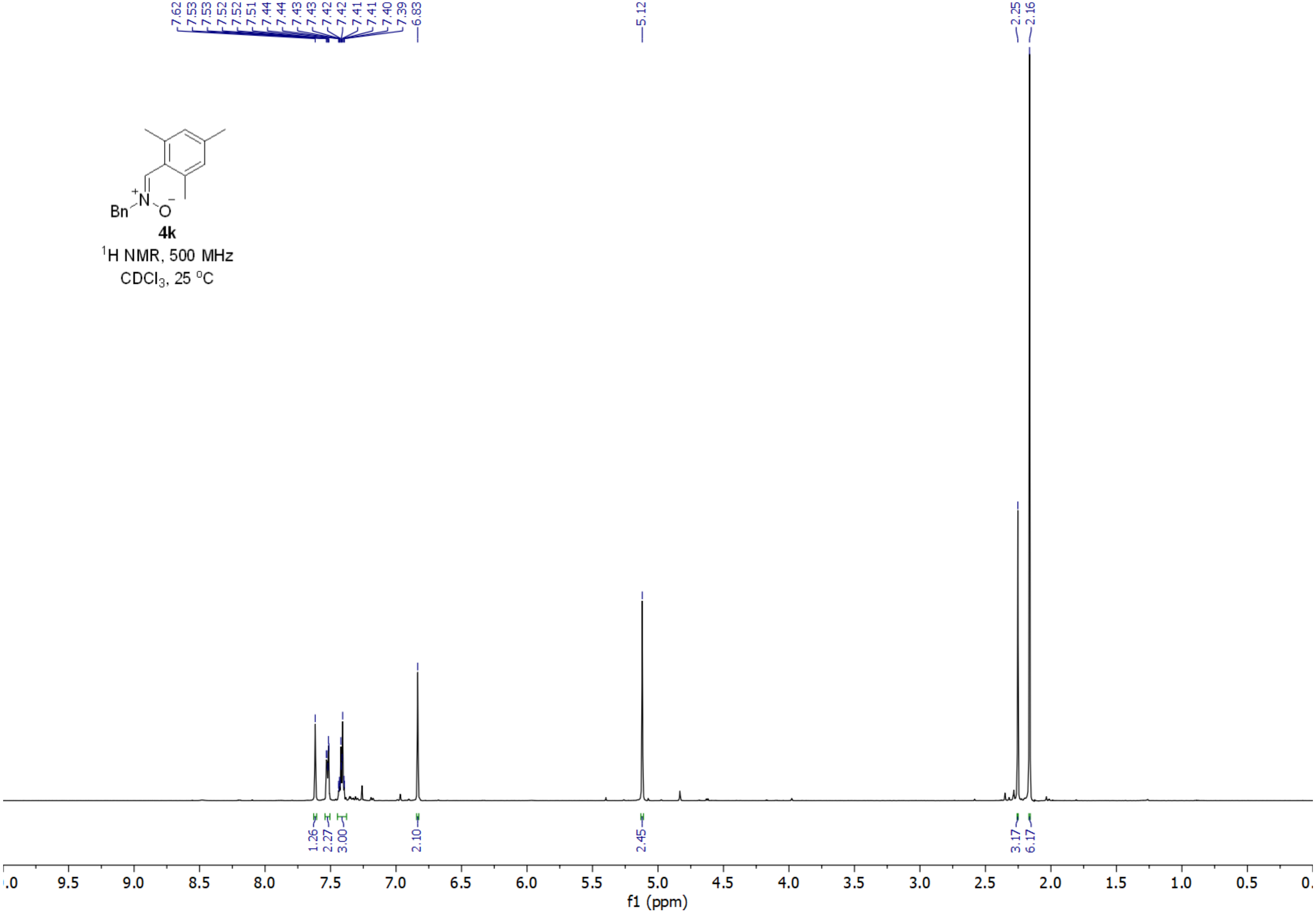
Bn
N
O
4k
1H NMR, 500 MHz
CDCl3, 25 oC
7.62
7.53
7.53
7.52
7.52
7.51
7.44
7.44
7.43
7.43
7.42
7.42
7.41
7.41
7.40
7.39
6.83
5.12
2.25
2.16
1.26
2.27
3.00
2.10
2.45
3.17
6.17
9.5
9.0
8.5
8.0
7.5
7.0
6.5
6.0
5.5
5.0
4.5
4.0
3.5
3.0
2.5
2.0
1.5
1.0
0.5
f1 (ppm)

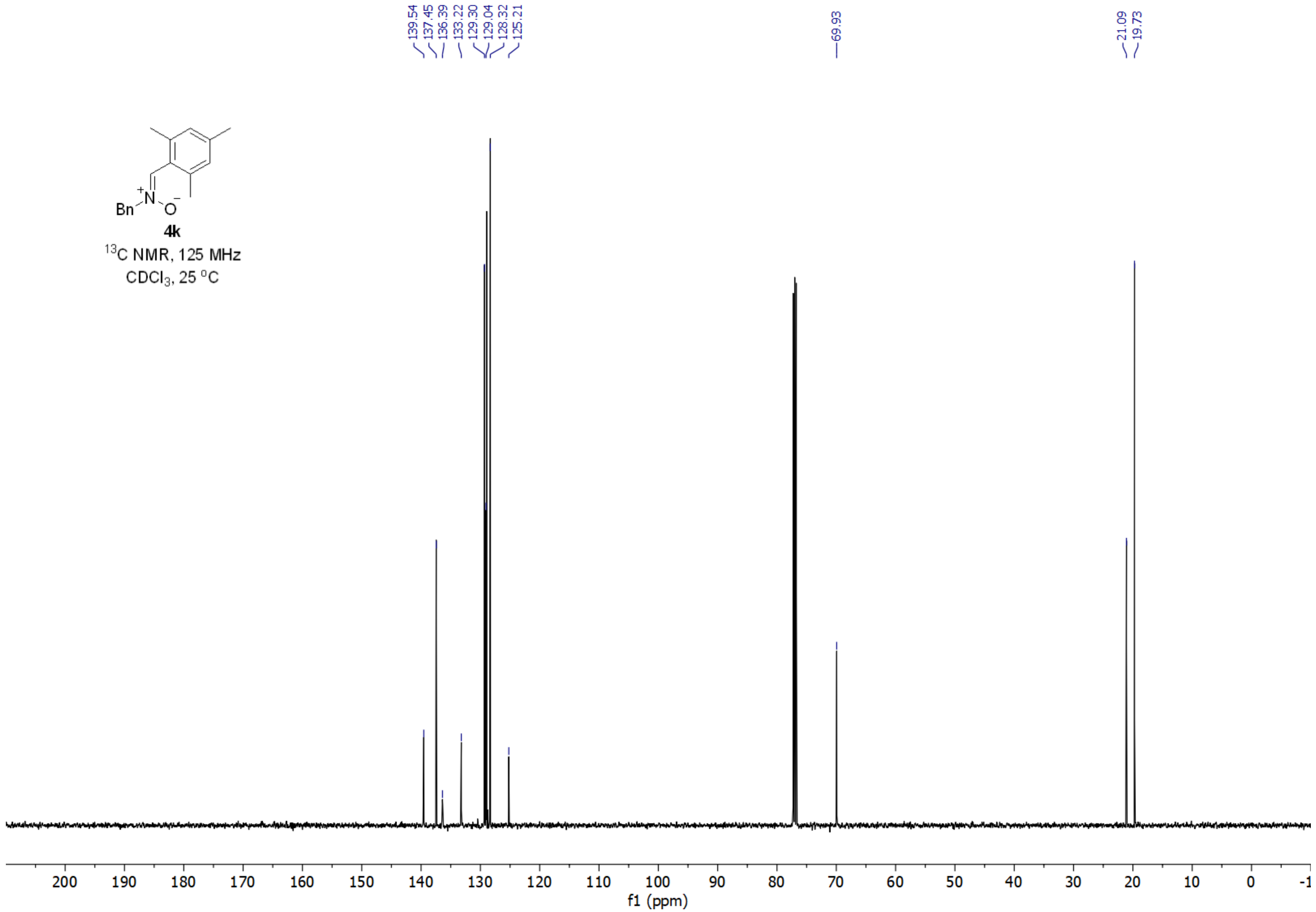
139.54
137.45
136.39
133.22
129.30
129.04
128.32
125.21
69.93
21.09
19.73
Bn
4k
^{13}C NMR, 125 MHz
$CDCl_3$, 25 °C
200 190 180 170 160 150 140 130 120 110 100 90 80 70 60 50 40 30 20 10 0 -1
f1 (ppm)

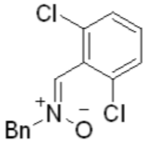

4l

^{1}H NMR, 500 MHz

$CDCl_3$, 25 °C

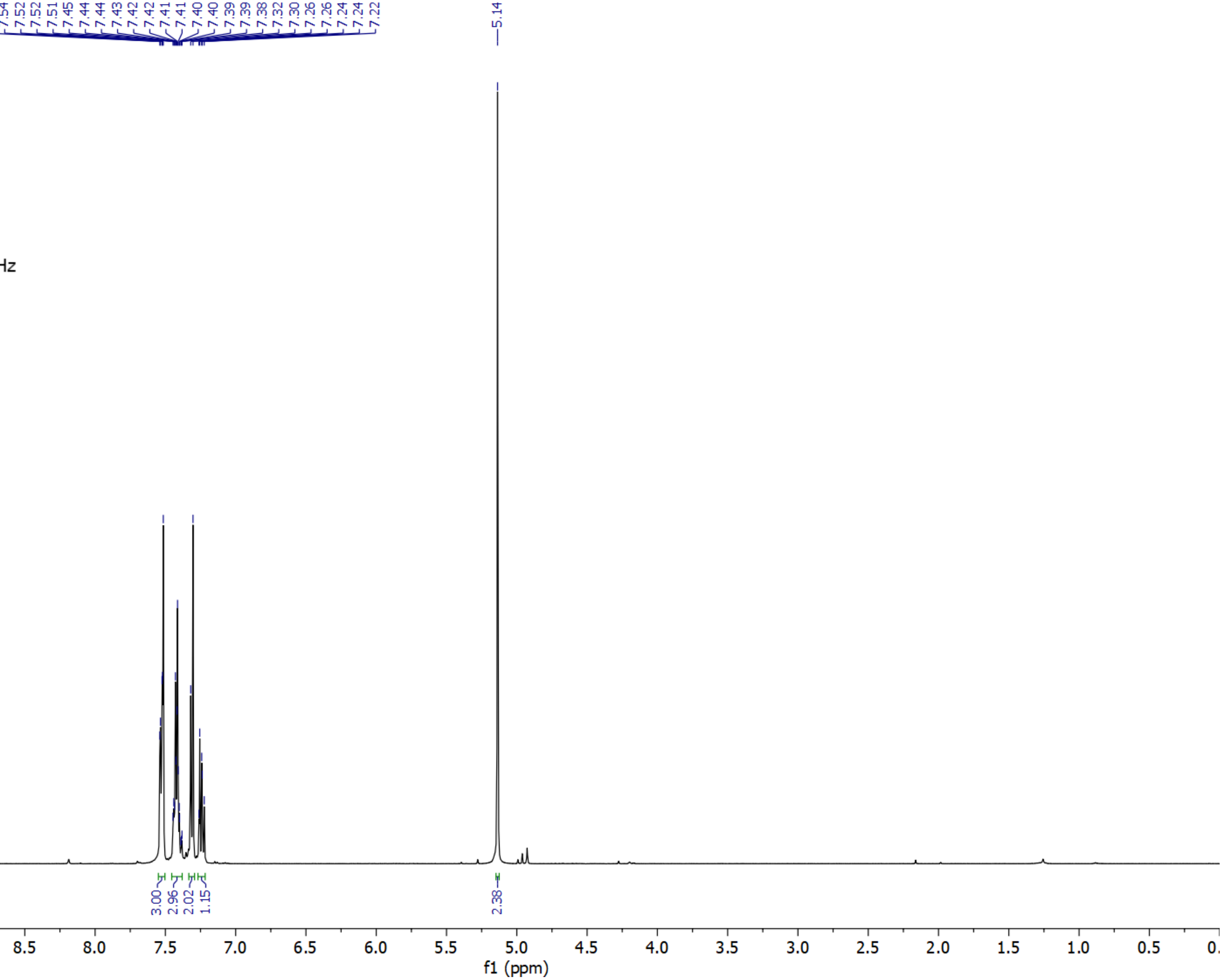

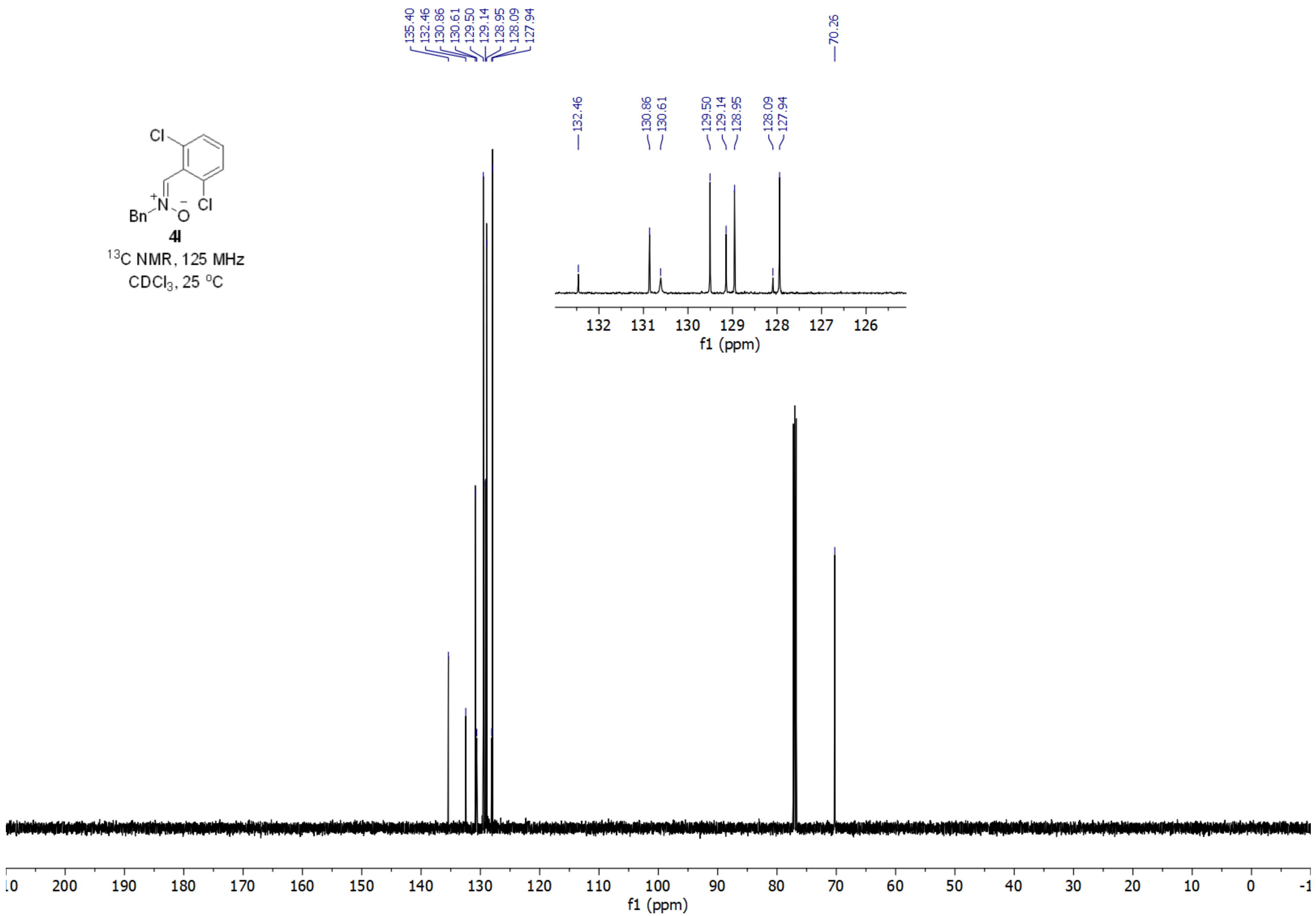
Cl
Cl
Bn
N
O
4l
13C NMR, 125 MHz
CDCl3, 25 °C
135.40
132.46
130.86
130.61
129.50
129.14
128.95
128.09
127.94
70.26
132
131
130
129
128
127
126
f1 (ppm)
200
190
180
170
160
150
140
130
120
110
100
90
80
70
60
50
40
30
20
10
0
f1 (ppm)

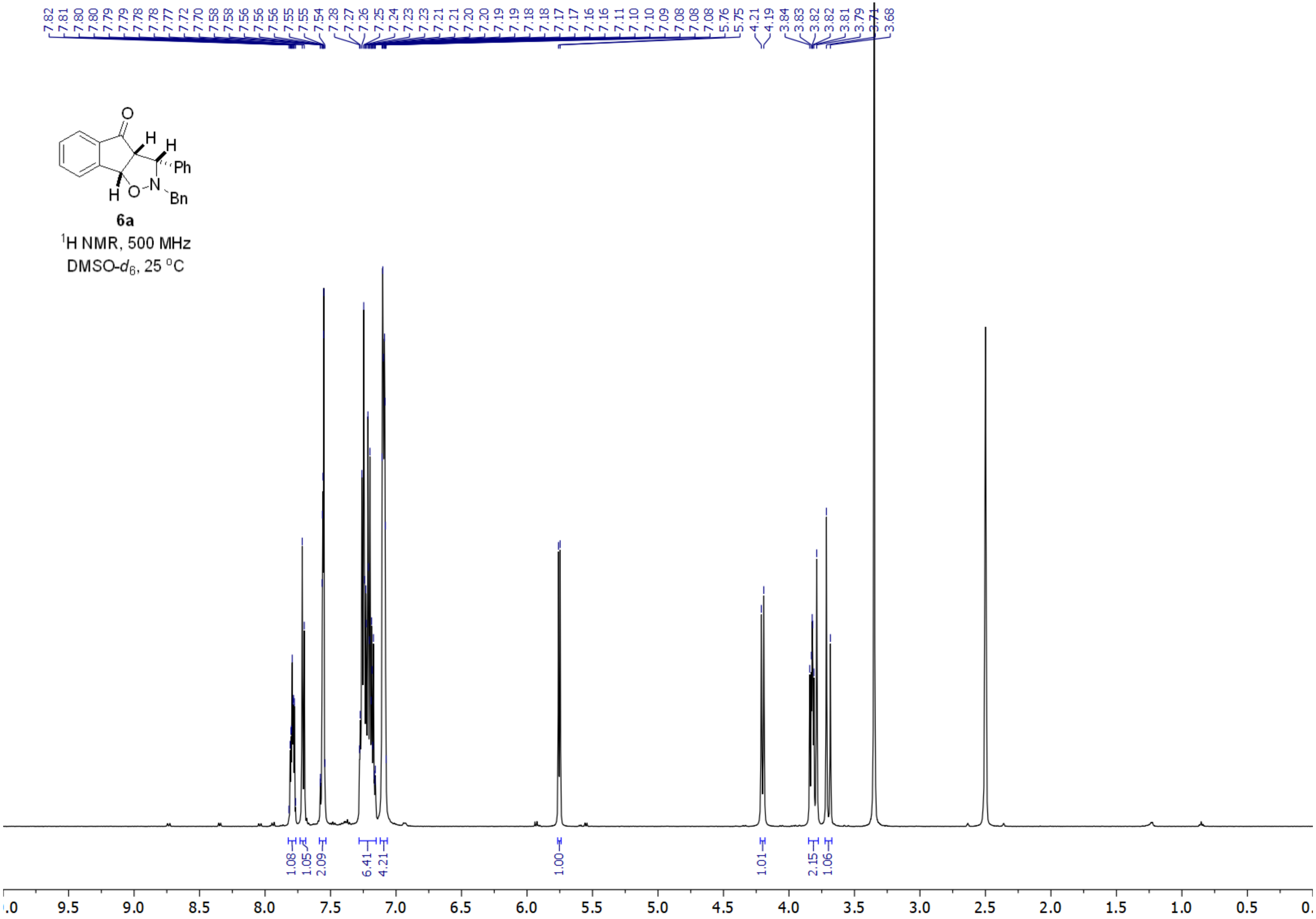
6a
1H NMR, 500 MHz
DMSO-d6, 25 °C
7.82 7.81 7.80 7.80 7.79 7.78 7.78 7.77 7.72 7.70 7.58 7.58 7.56 7.56 7.55 7.55 7.54 7.28 7.27 7.26 7.25 7.24 7.23 7.23 7.21 7.21 7.20 7.19 7.19 7.18 7.18 7.17 7.17 7.16 7.16 7.11 7.10 7.09 7.08 7.08 7.08 5.76 5.75 4.21 4.19 3.84 3.83 3.82 3.82 3.81 3.79 3.71 3.68
1.08 1.05 2.09 6.41 4.21 1.00 1.01 2.15 1.06
9.5 9.0 8.5 8.0 7.5 7.0 6.5 6.0 5.5 5.0 4.5 4.0 3.5 3.0 2.5 2.0 1.5 1.0 0.5

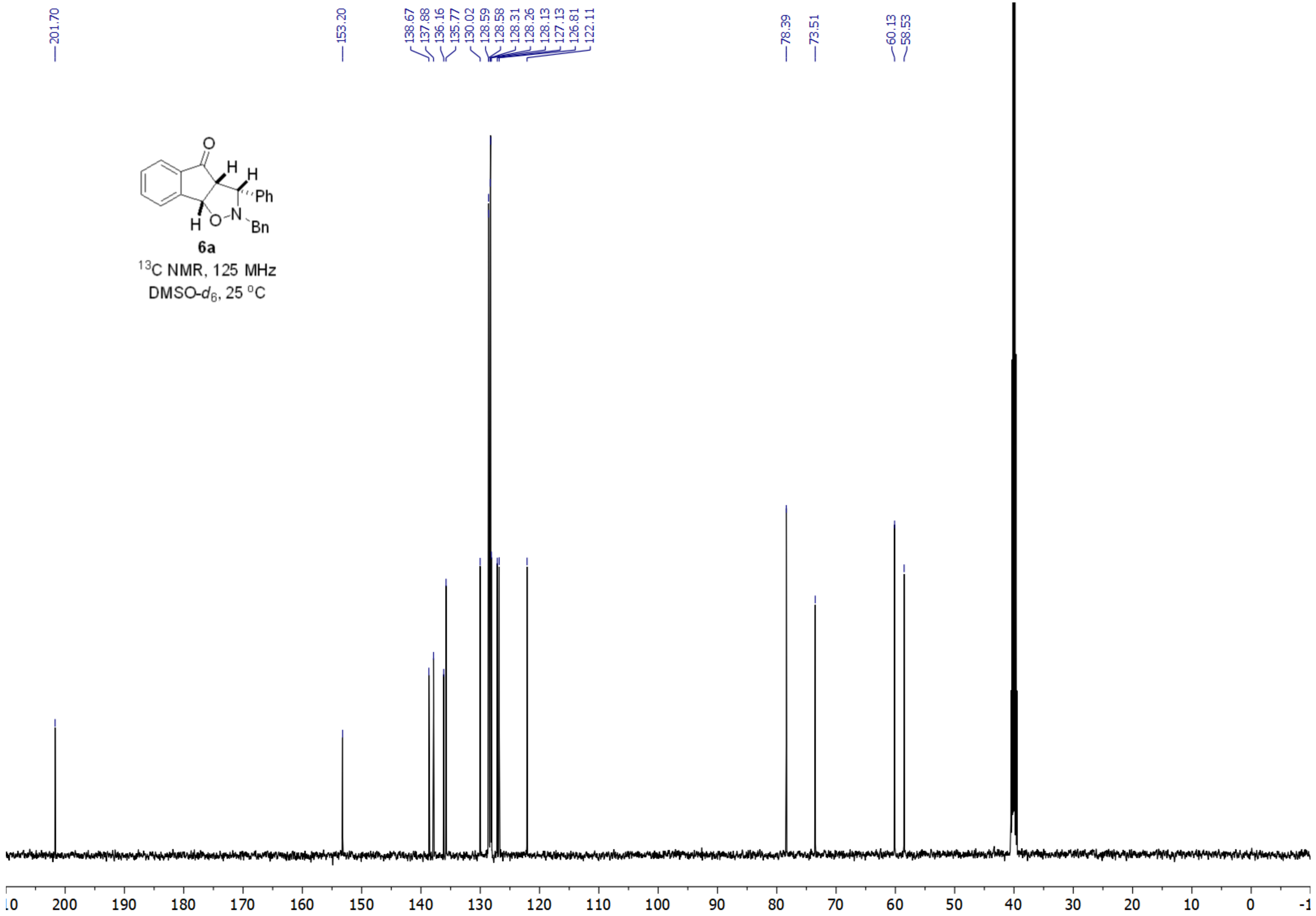
201.70
153.20
138.67
137.88
136.16
135.77
130.02
128.59
128.58
128.31
128.26
128.13
127.13
126.81
122.11
78.39
73.51
60.13
58.53
O
H
H
Ph
H
O
N
Bn
6a
13C NMR, 125 MHz
DMSO-d6, 25 °C
10
200
190
180
170
160
150
140
130
120
110
100
90
80
70
60
50
40
30
20
10
0
-1

6a

COSY ^1H-^1H NMR, 500 MHz

DMSO-d_6, 25 °C

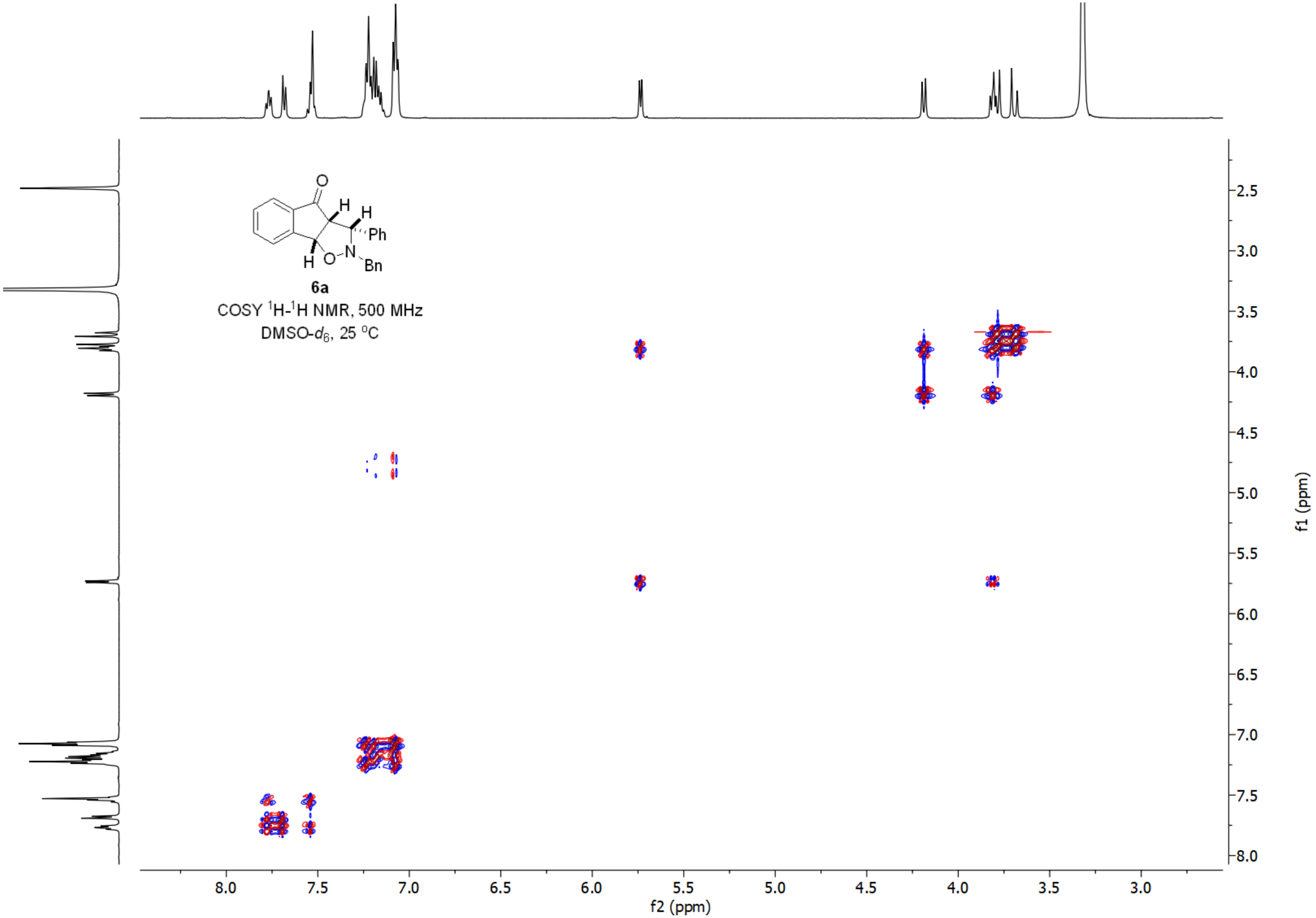

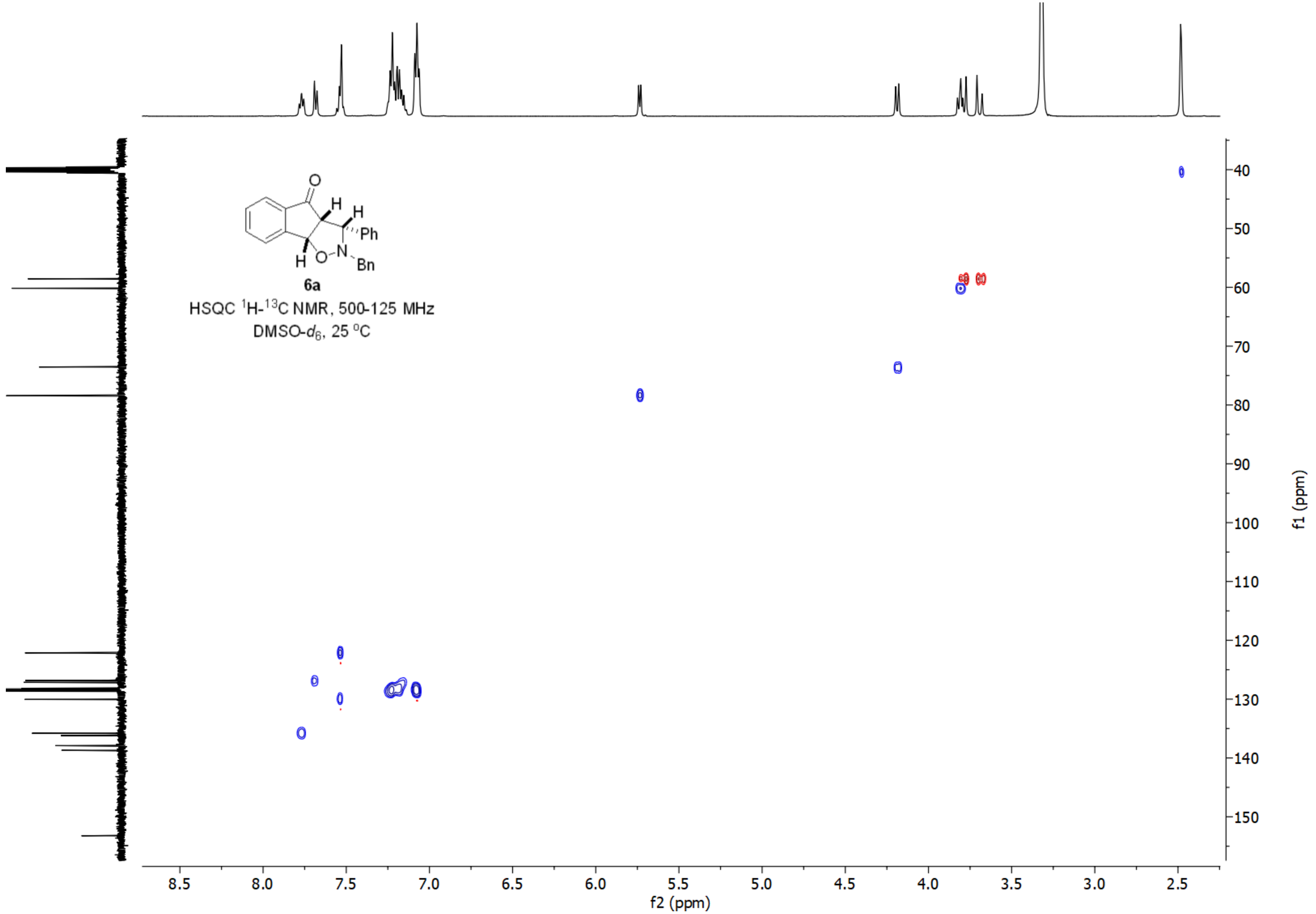
6a
HSQC 1H-13C NMR, 500-125 MHz
DMSO-d6, 25 °C
f2 (ppm)
f1 (ppm)

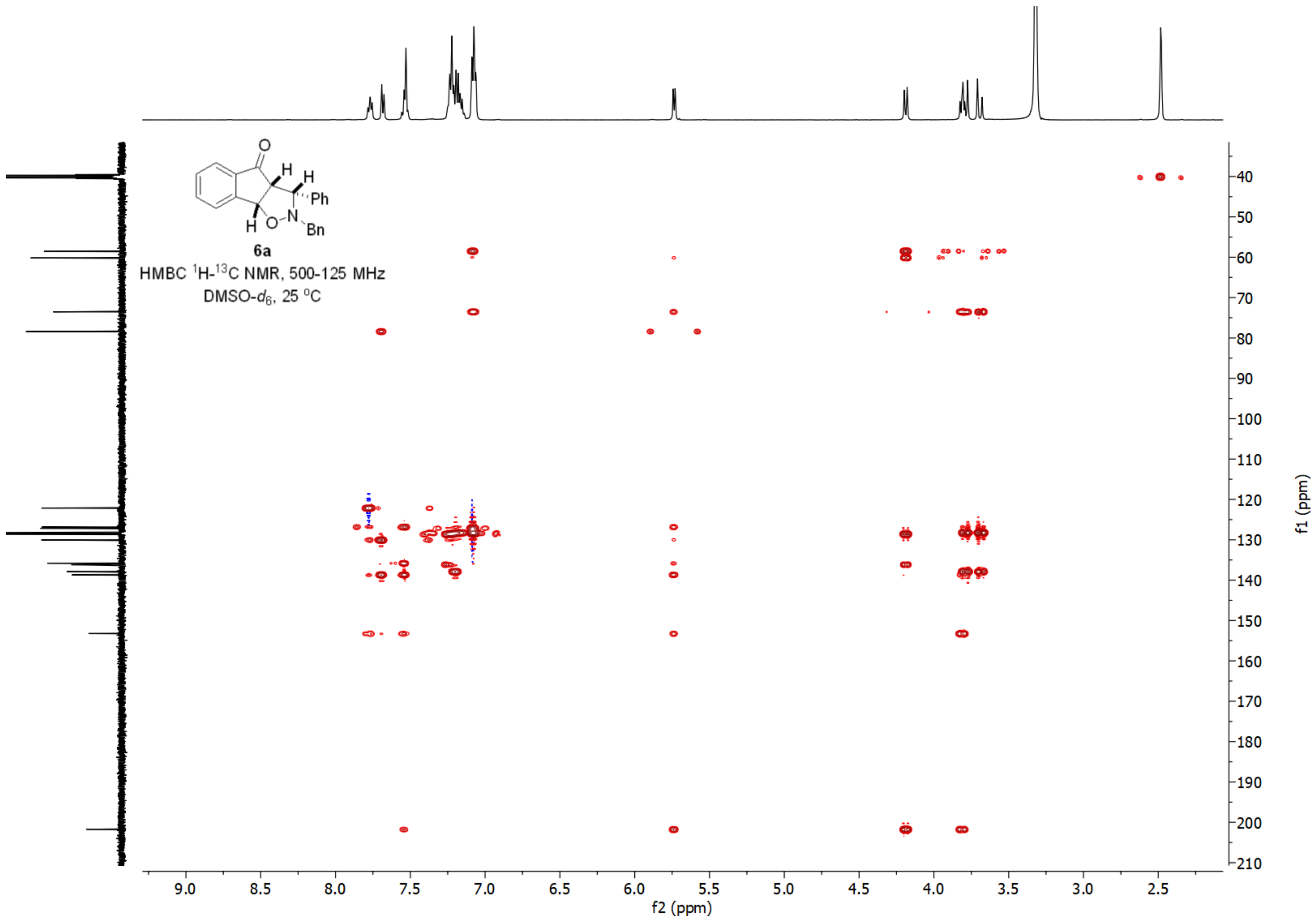

6a
HMBC ^{1}H-^{13}C NMR, 500-125 MHz
DMSO-d_6, 25 °C
f2 (ppm)
f1 (ppm)

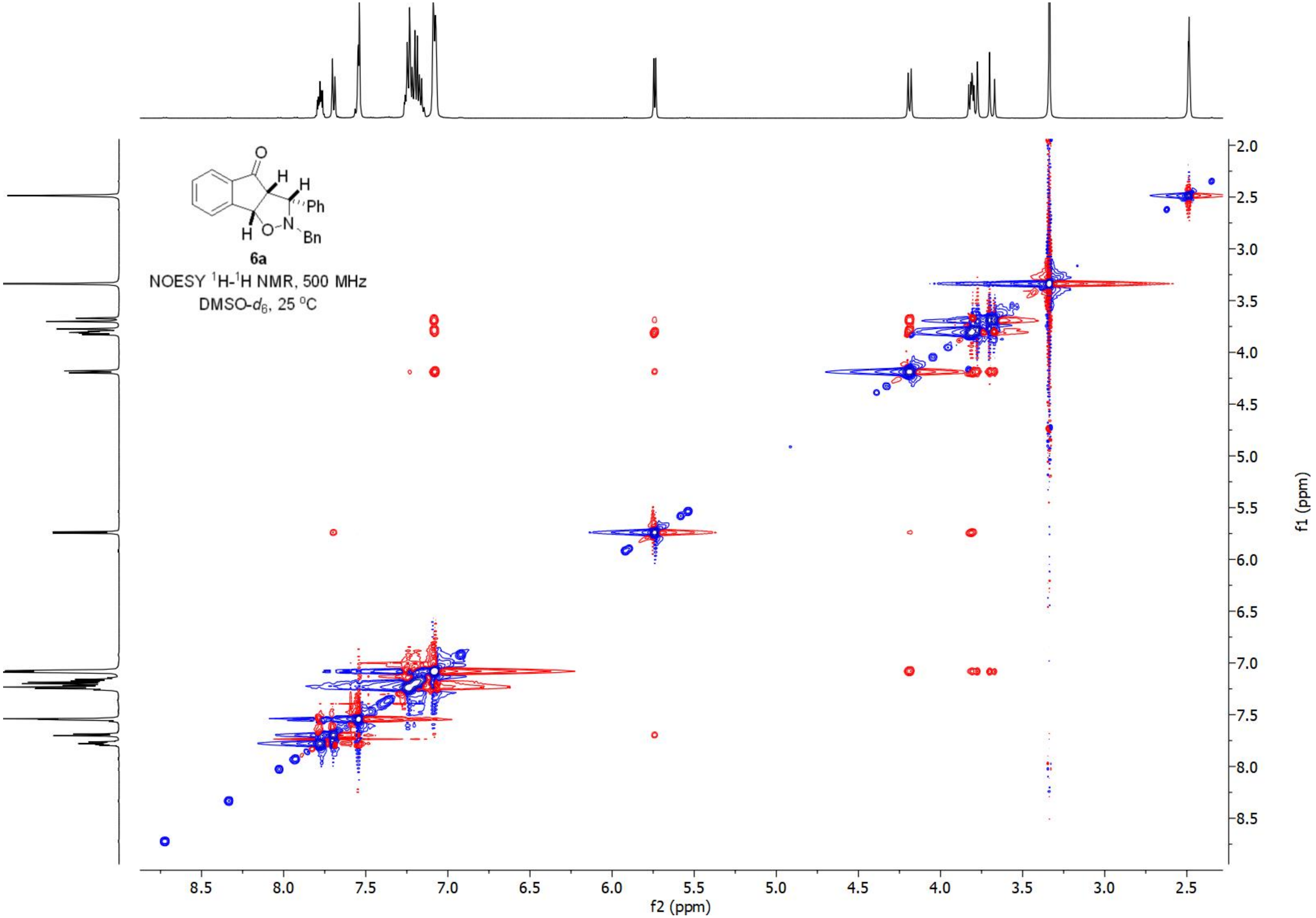

6a
NOESY ¹H-¹H NMR, 500 MHz
DMSO-d6, 25 °C
f2 (ppm)
f1 (ppm)

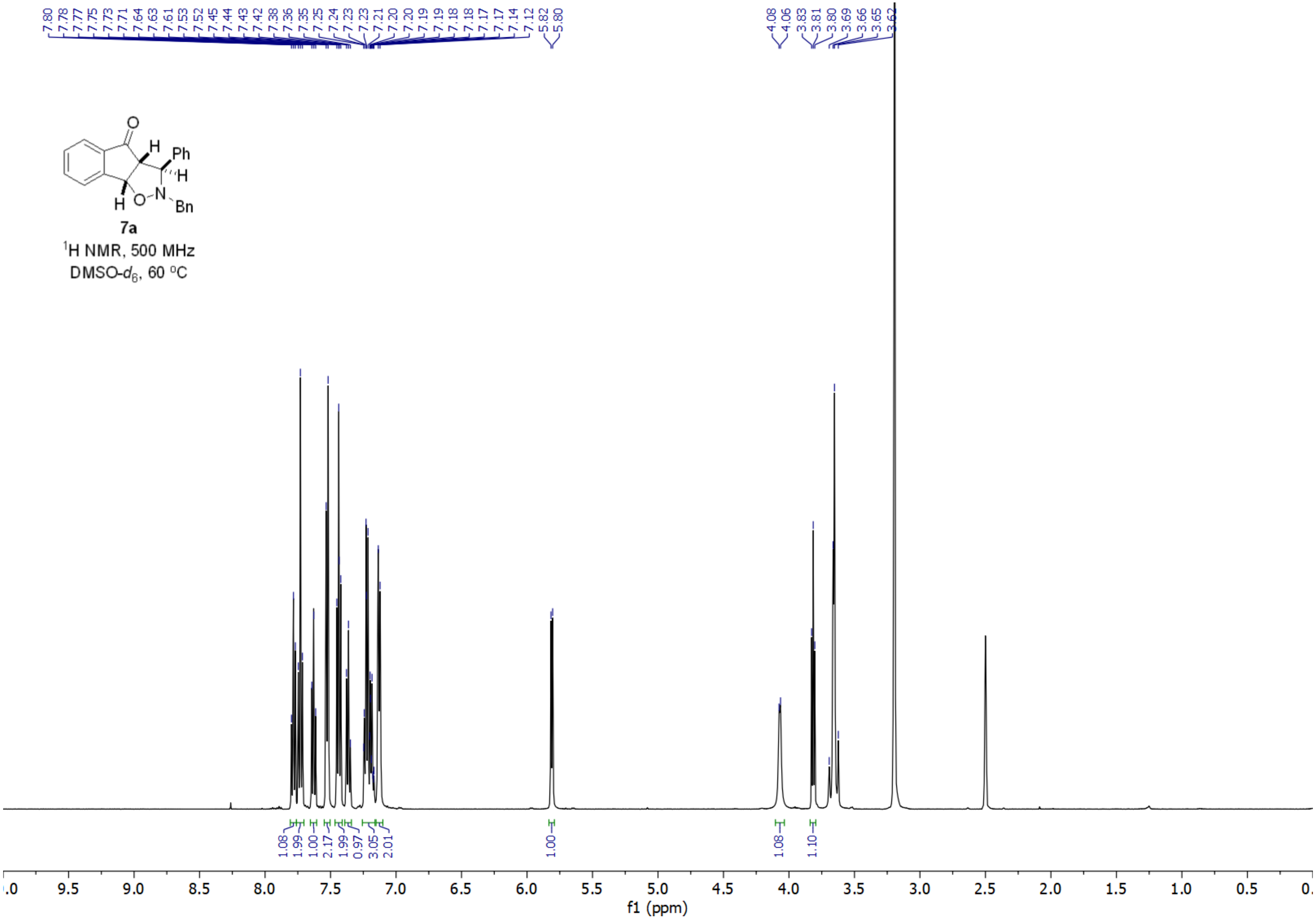

7a
¹H NMR, 500 MHz
DMSO-d6, 60 °C
f1 (ppm)

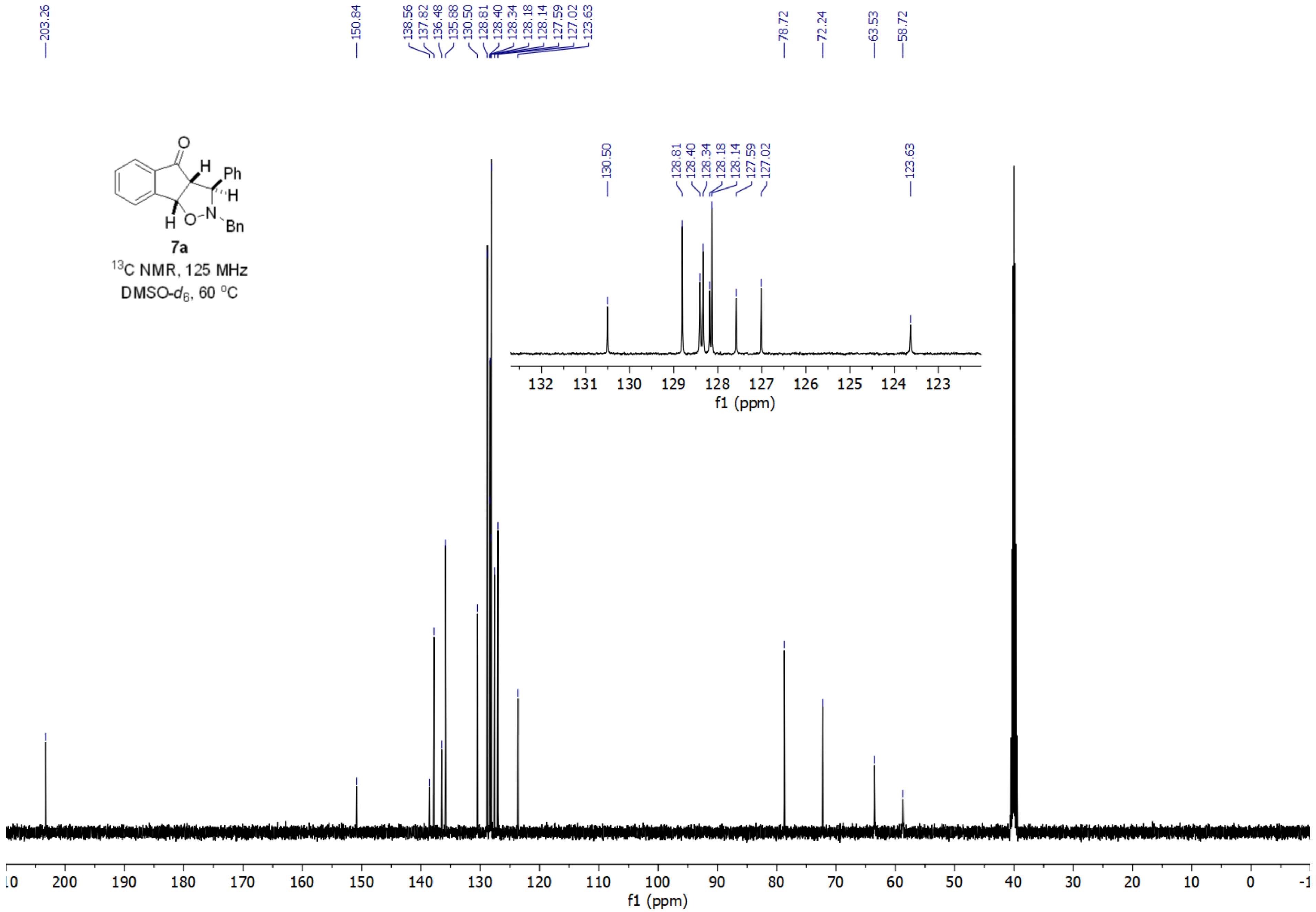
203.26
150.84
138.56
137.82
136.48
135.88
130.50
128.81
128.40
128.34
128.18
128.14
127.59
127.02
123.63
78.72
72.24
63.53
58.72
O
H
Ph
H
H
O
N
Bn
7a
13C NMR, 125 MHz
DMSO-d6, 60 °C
f1 (ppm)

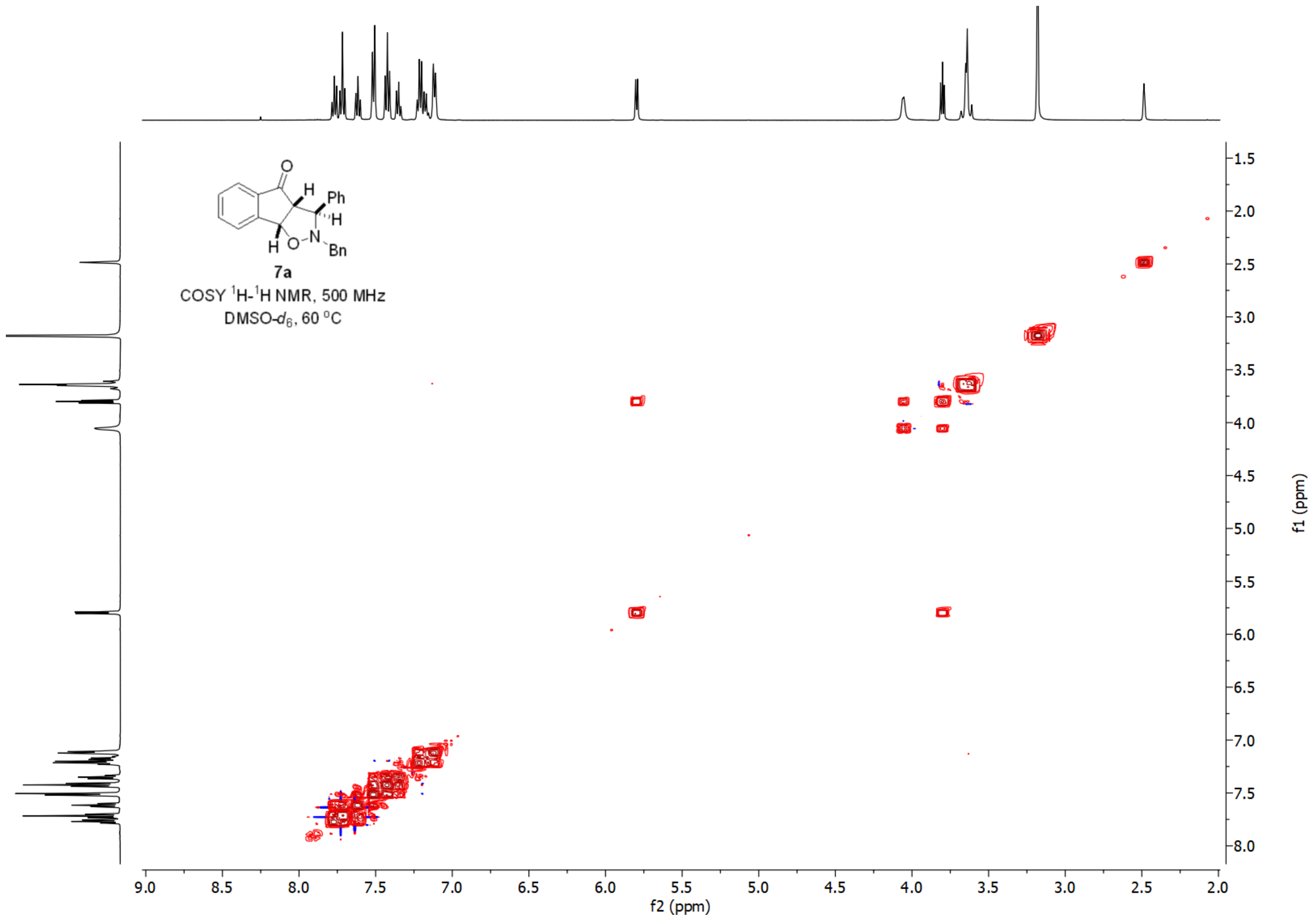
O
H
Ph
H
H
O
N
Bn
7a
COSY 1H-1H NMR, 500 MHz
DMSO-d6, 60 °C
f2 (ppm)
f1 (ppm)

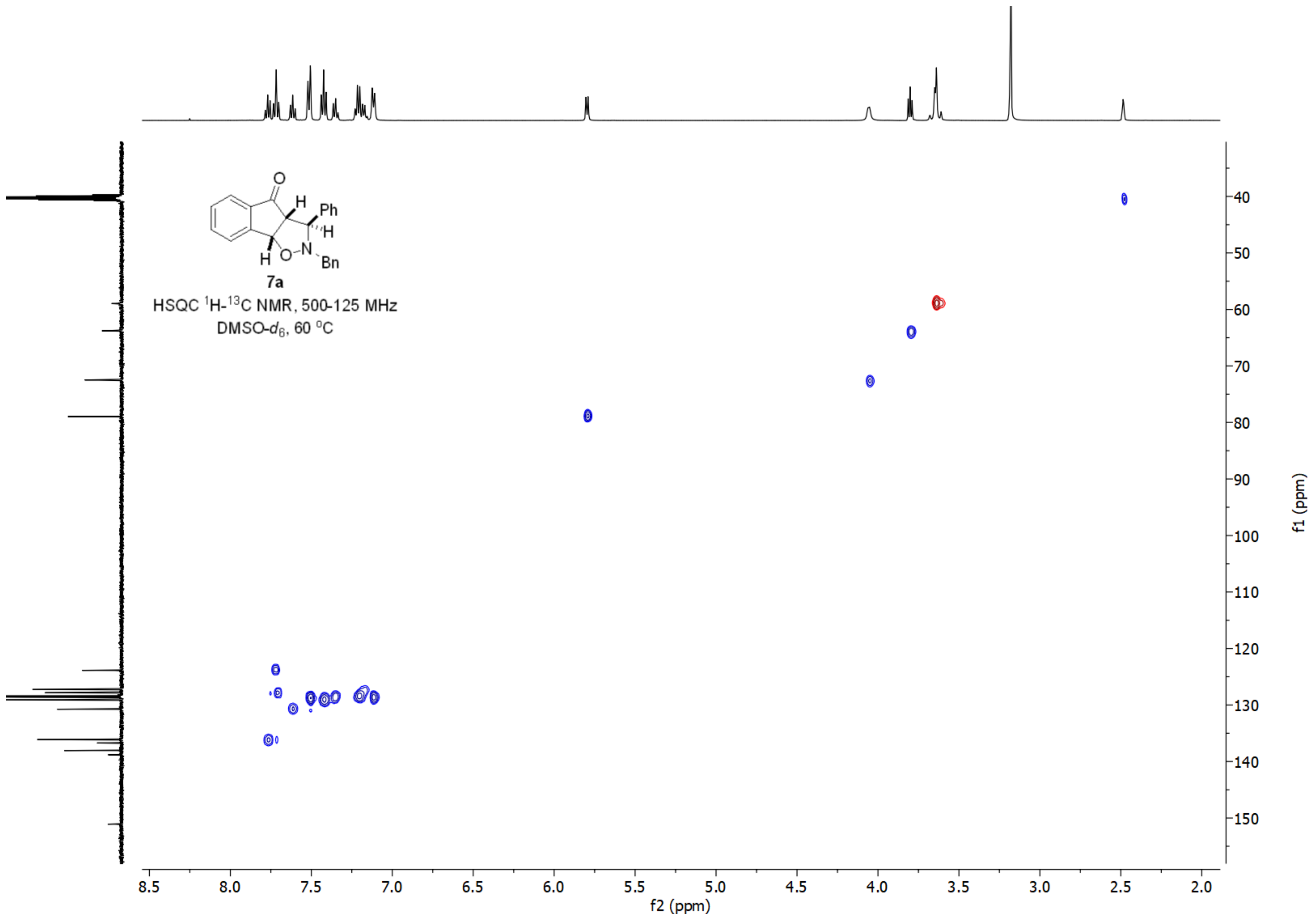

O
H
Ph
H
H
O
N
Bn
7a
HSQC ¹H-¹³C NMR, 500-125 MHz
DMSO-d6, 60 °C
f1 (ppm)
f2 (ppm)

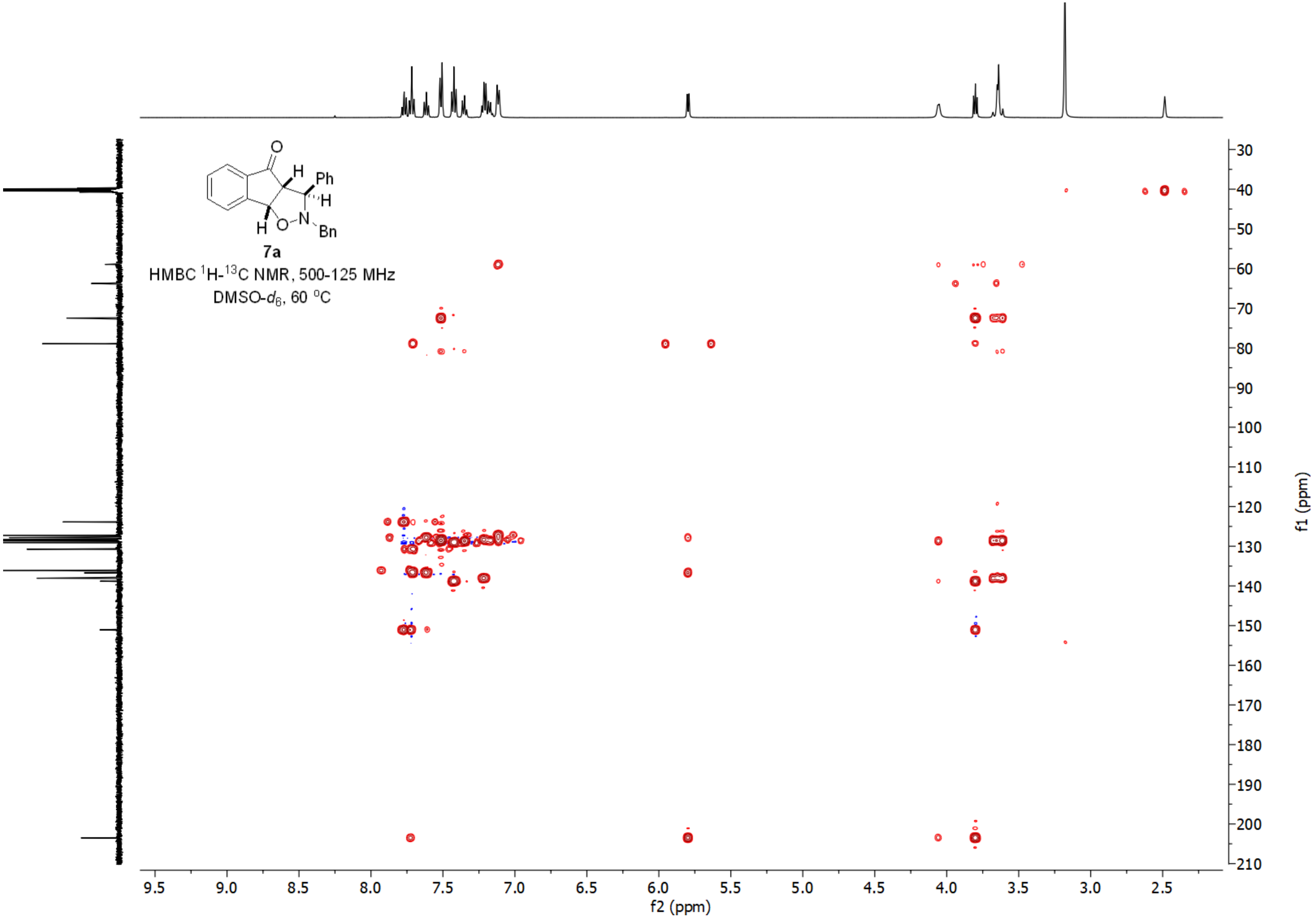
O
H
Ph
H
H
O
N
Bn
7a
HMBC ^{1}H-^{13}C NMR, 500-125 MHz
DMSO-d_6, 60 °C
f2 (ppm)
f1 (ppm)

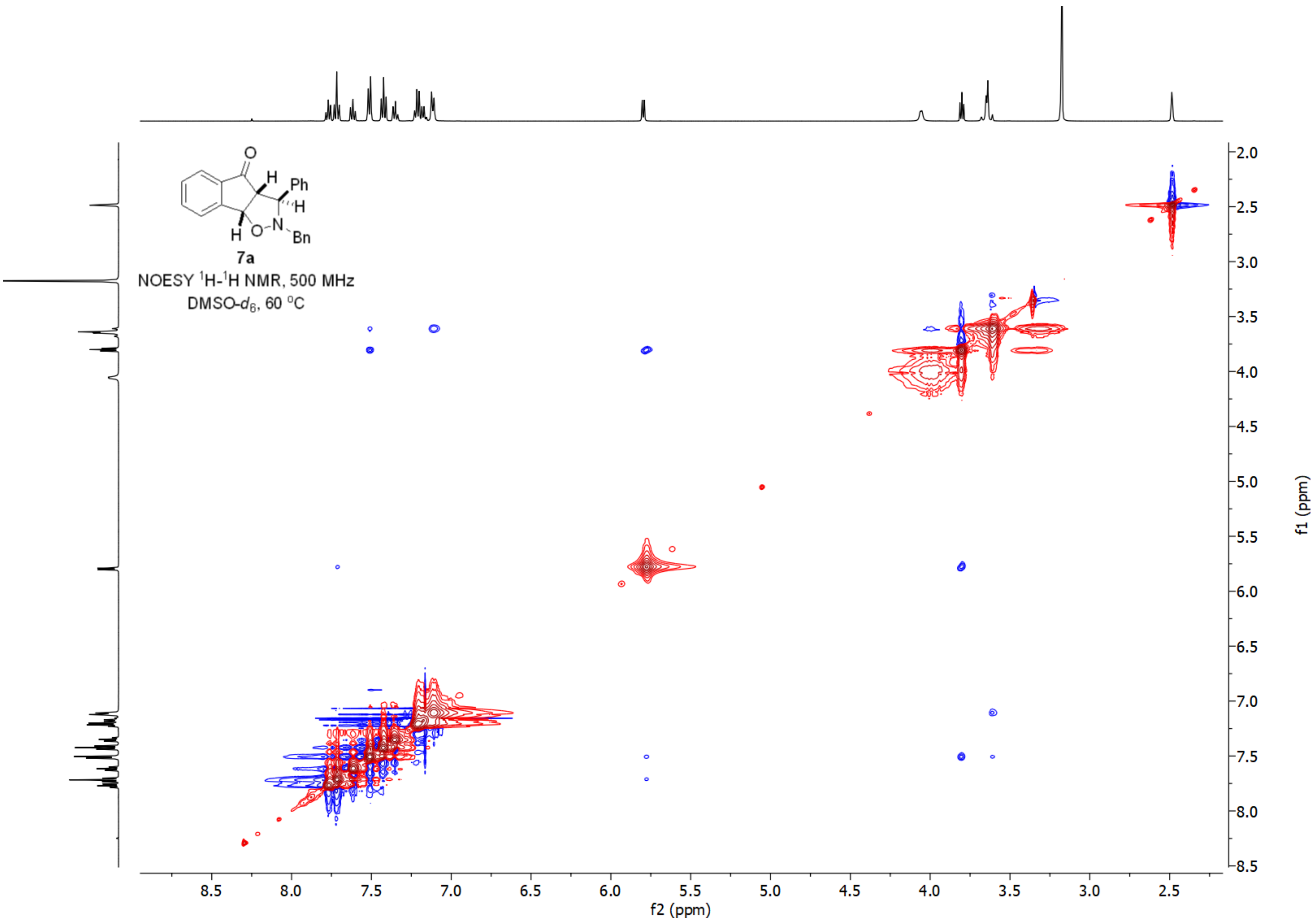
O
H
Ph
H
H
O
N
Bn
7a
NOESY ¹H-¹H NMR, 500 MHz
DMSO-d6, 60 °C
f2 (ppm)
f1 (ppm)

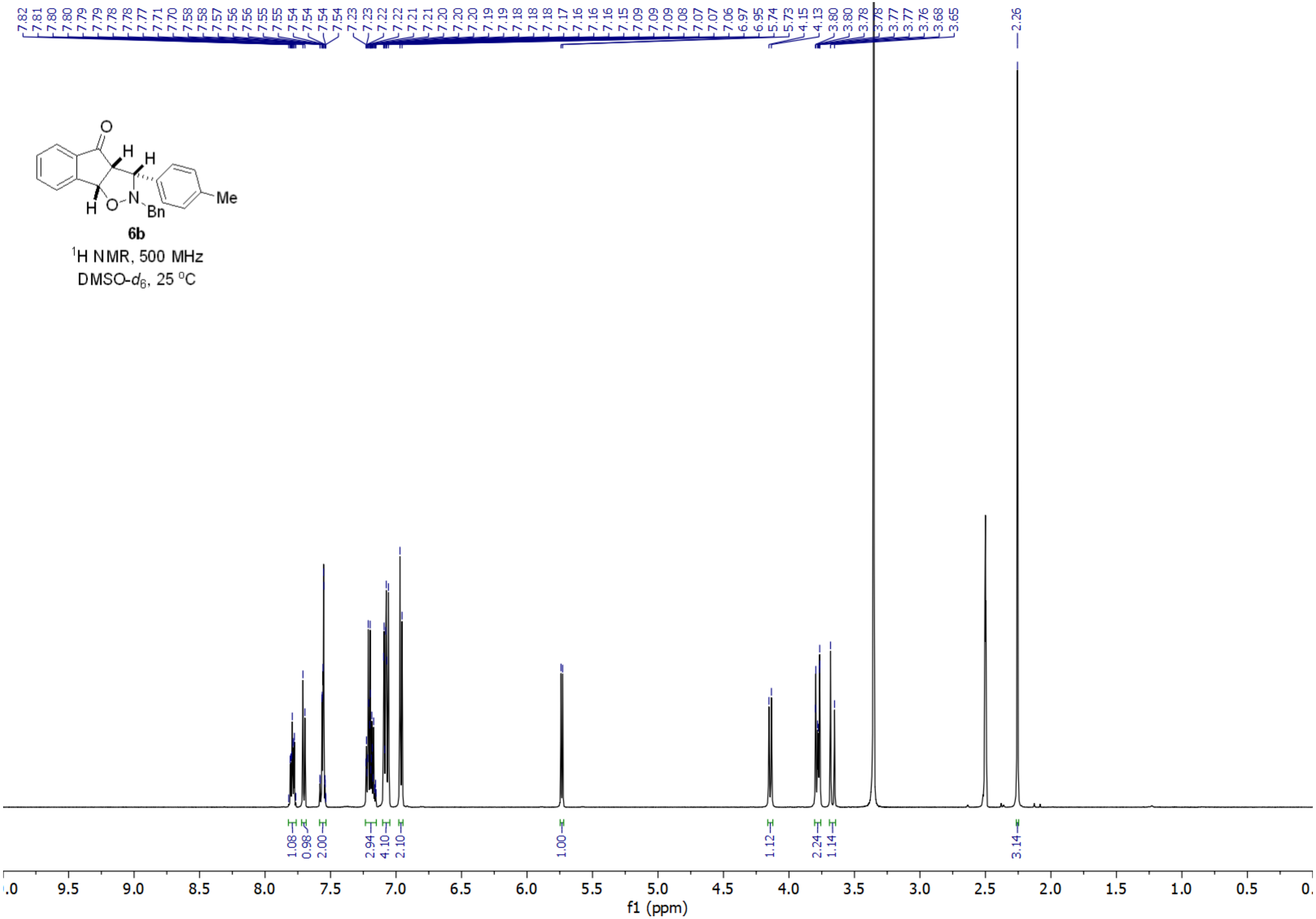
7.82 7.81 7.80 7.79 7.79 7.78 7.78 7.77 7.71 7.70 7.58 7.58 7.57 7.56 7.56 7.55 7.55 7.54 7.54 7.54 7.54 7.23 7.23 7.22 7.22 7.21 7.20 7.20 7.20 7.19 7.18 7.18 7.18 7.17 7.16 7.16 7.15 7.09 7.09 7.08 7.07 7.07 7.06 6.97 6.95 5.74 5.73 4.15 4.13 3.80 3.80 3.78 3.78 3.77 3.77 3.76 3.68 3.65
2.26
6b
1H NMR, 500 MHz
DMSO-d6, 25 °C
1.08 0.98 2.00 2.94 4.10 2.10 1.00 1.12 2.24 1.14 3.14
.0 9.5 9.0 8.5 8.0 7.5 7.0 6.5 6.0 5.5 5.0 4.5 4.0 3.5 3.0 2.5 2.0 1.5 1.0 0.5 0.
f1 (ppm)

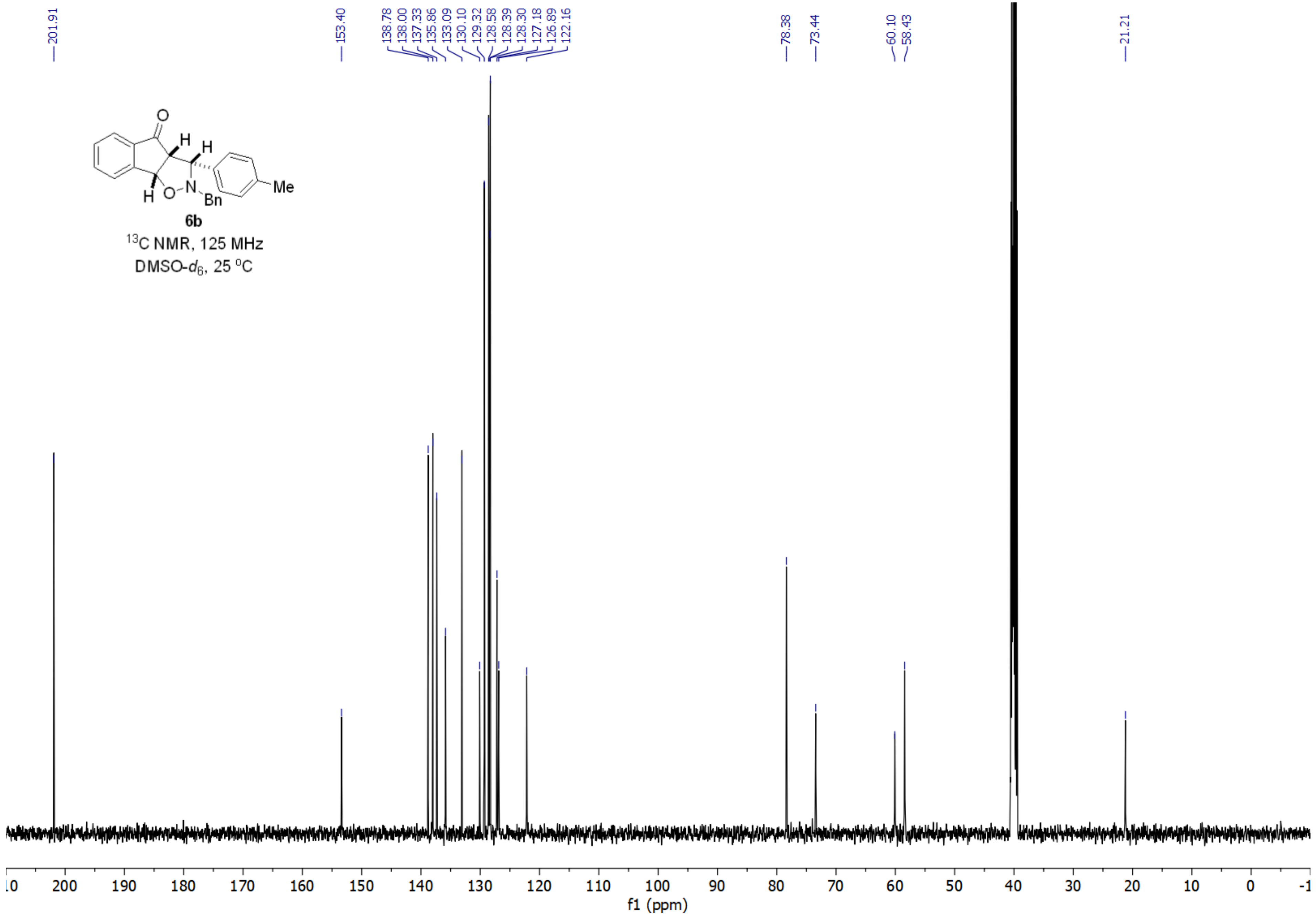
201.91
153.40
138.78
138.00
137.33
135.86
133.09
130.10
129.32
128.58
128.39
128.30
127.18
126.89
122.16
78.38
73.44
60.10
58.43
21.21
O
H
H
Me
H
O
N
Bn
6b
^{13}C NMR, 125 MHz
DMSO-d_6, 25 °C
f1 (ppm)

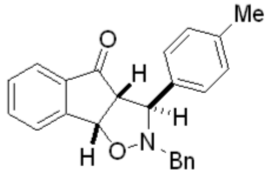

7b

^{1}H NMR, 500 MHz

DMSO-d_6, 60 °C

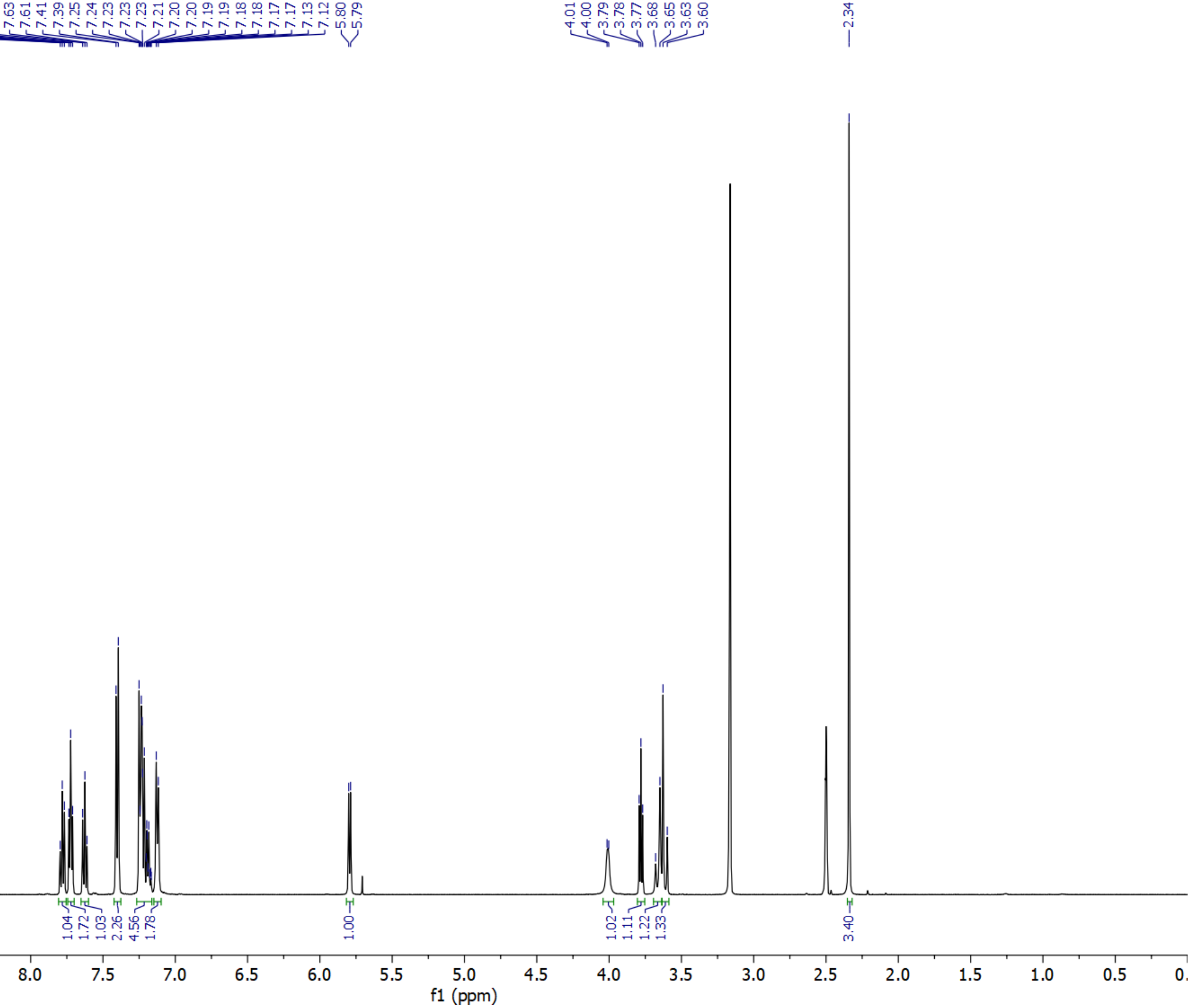

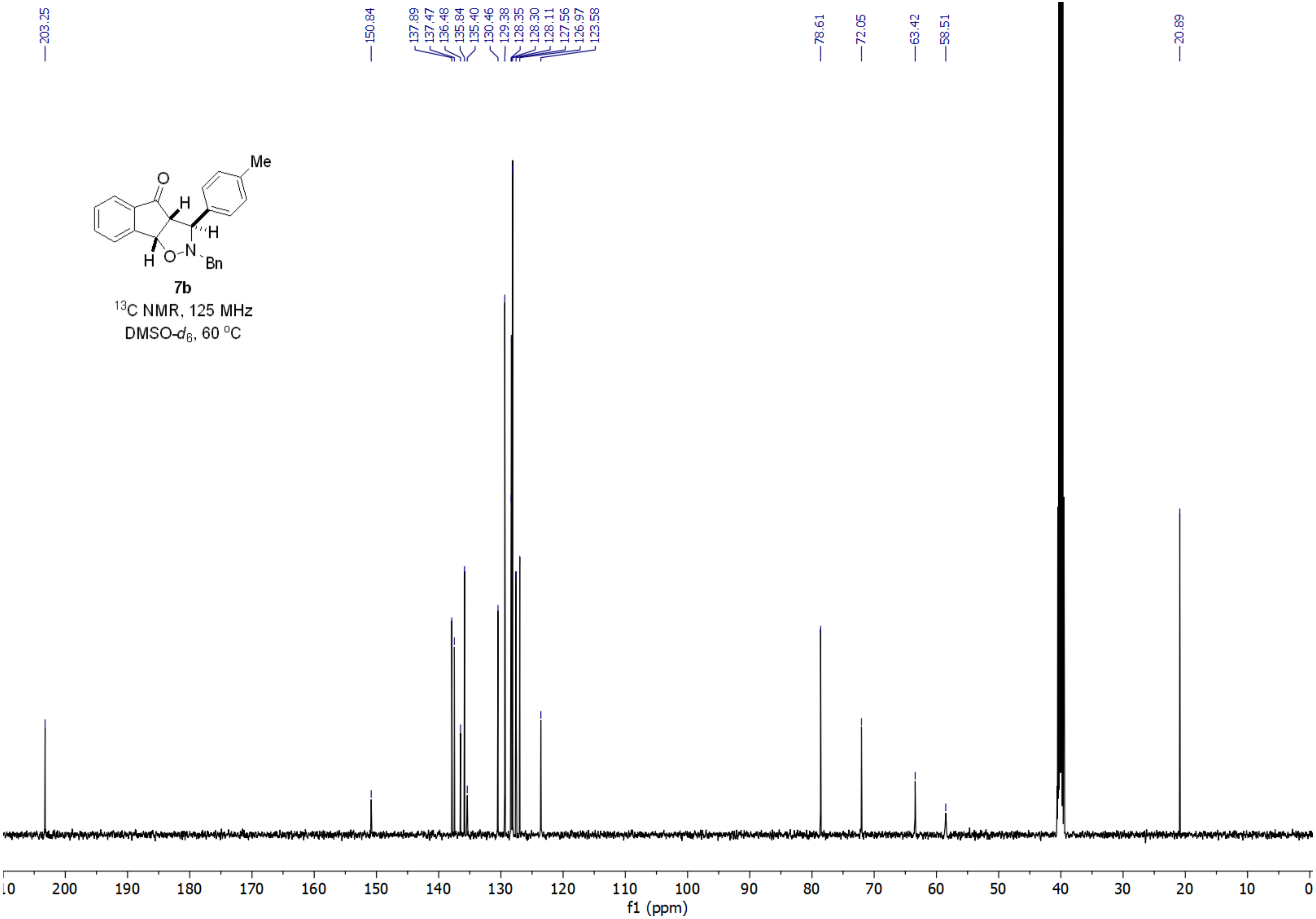

7b

^{13}C NMR, 125 MHz

DMSO-d_6, 60 °C

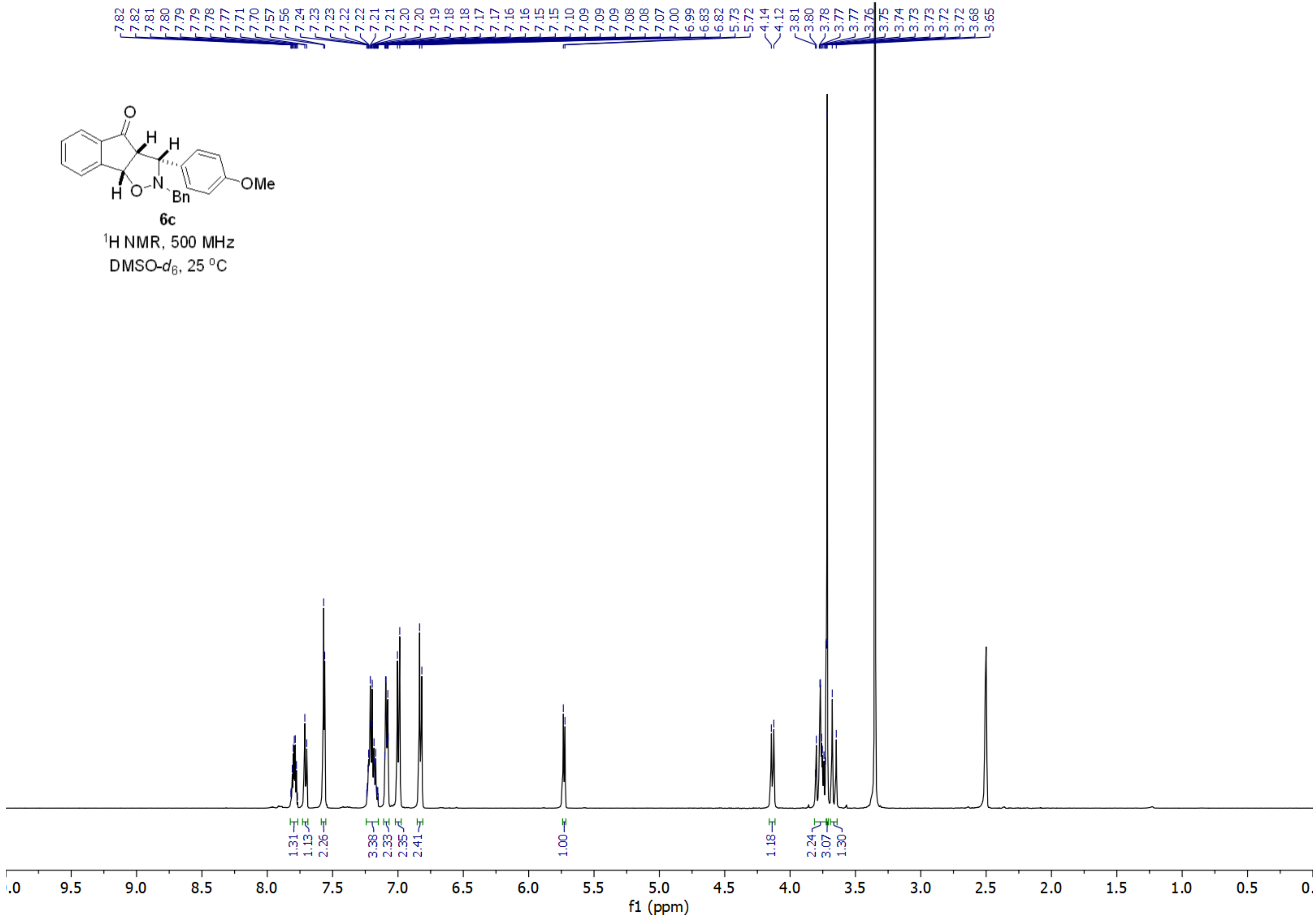
6c
¹H NMR, 500 MHz
DMSO-d_6, 25 °C
OMe
Bn
f1 (ppm)

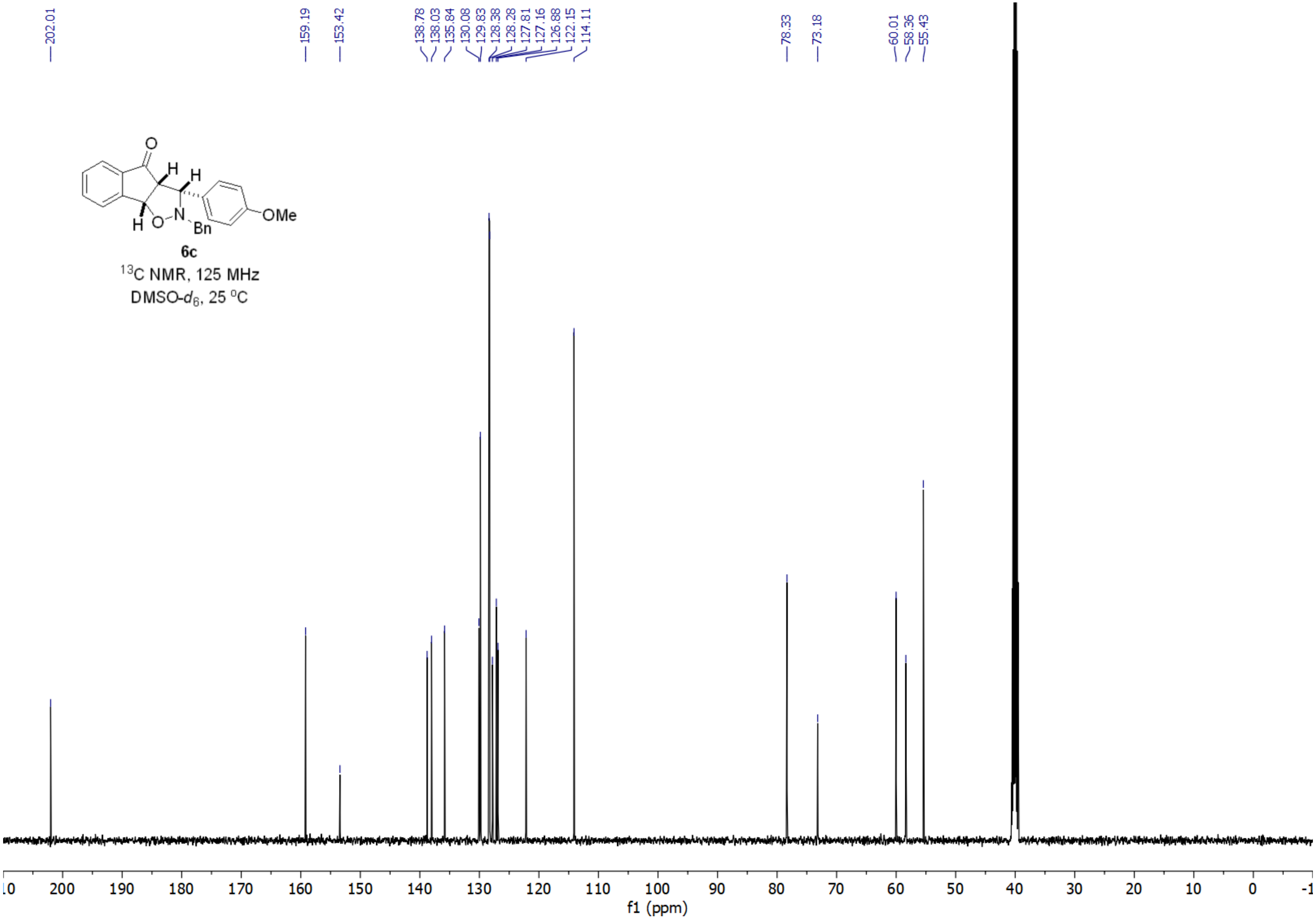
202.01
159.19
153.42
138.78
138.03
135.84
130.08
129.83
128.38
128.28
127.81
127.16
126.88
122.15
114.11
78.33
73.18
60.01
58.36
55.43
O
H
H
OMe
H
O
N
Bn
6c
^{13}C NMR, 125 MHz
DMSO-d_6, 25 °C
f1 (ppm)

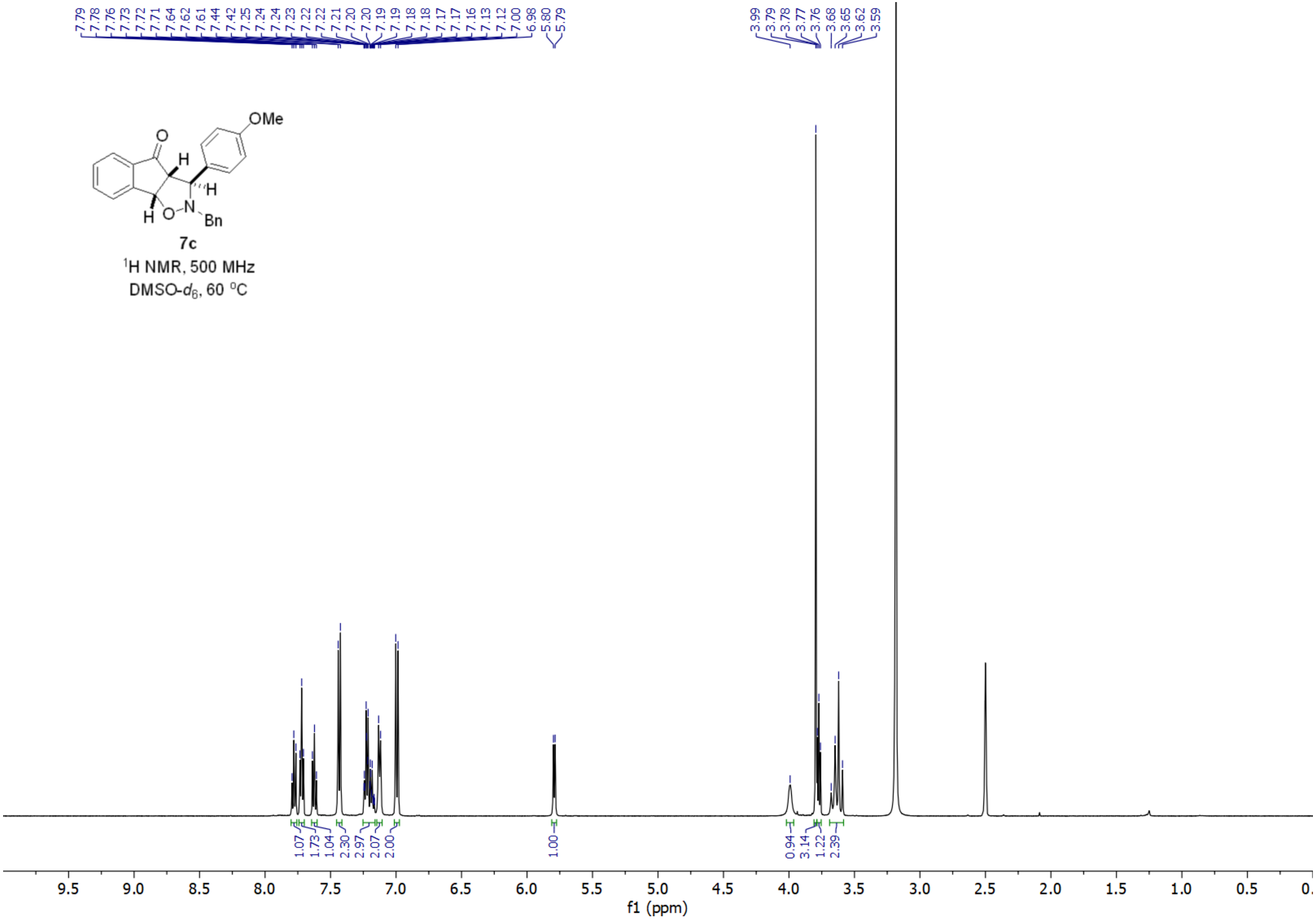

7c

^{1}H NMR, 500 MHz

DMSO-d_6, 60 °C

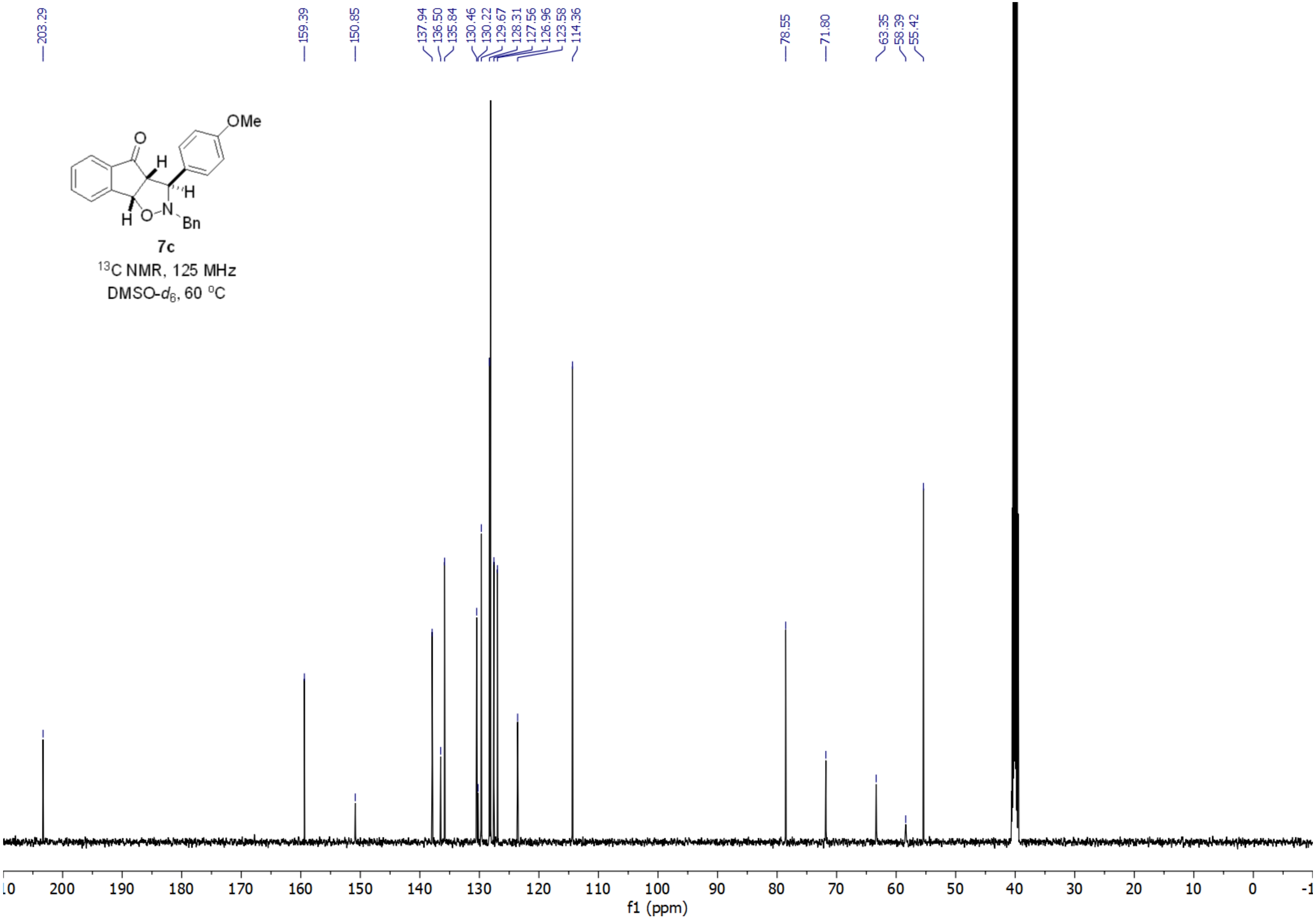
203.29
159.39
150.85
137.94
136.50
135.84
130.46
130.22
129.67
128.31
127.56
126.96
123.58
114.36
78.55
71.80
63.35
58.39
55.42
OMe
O
H
H
H
O
N
Bn
7c
13C NMR, 125 MHz
DMSO-d6, 60 °C
200 190 180 170 160 150 140 130 120 110 100 90 80 70 60 50 40 30 20 10 0
f1 (ppm)

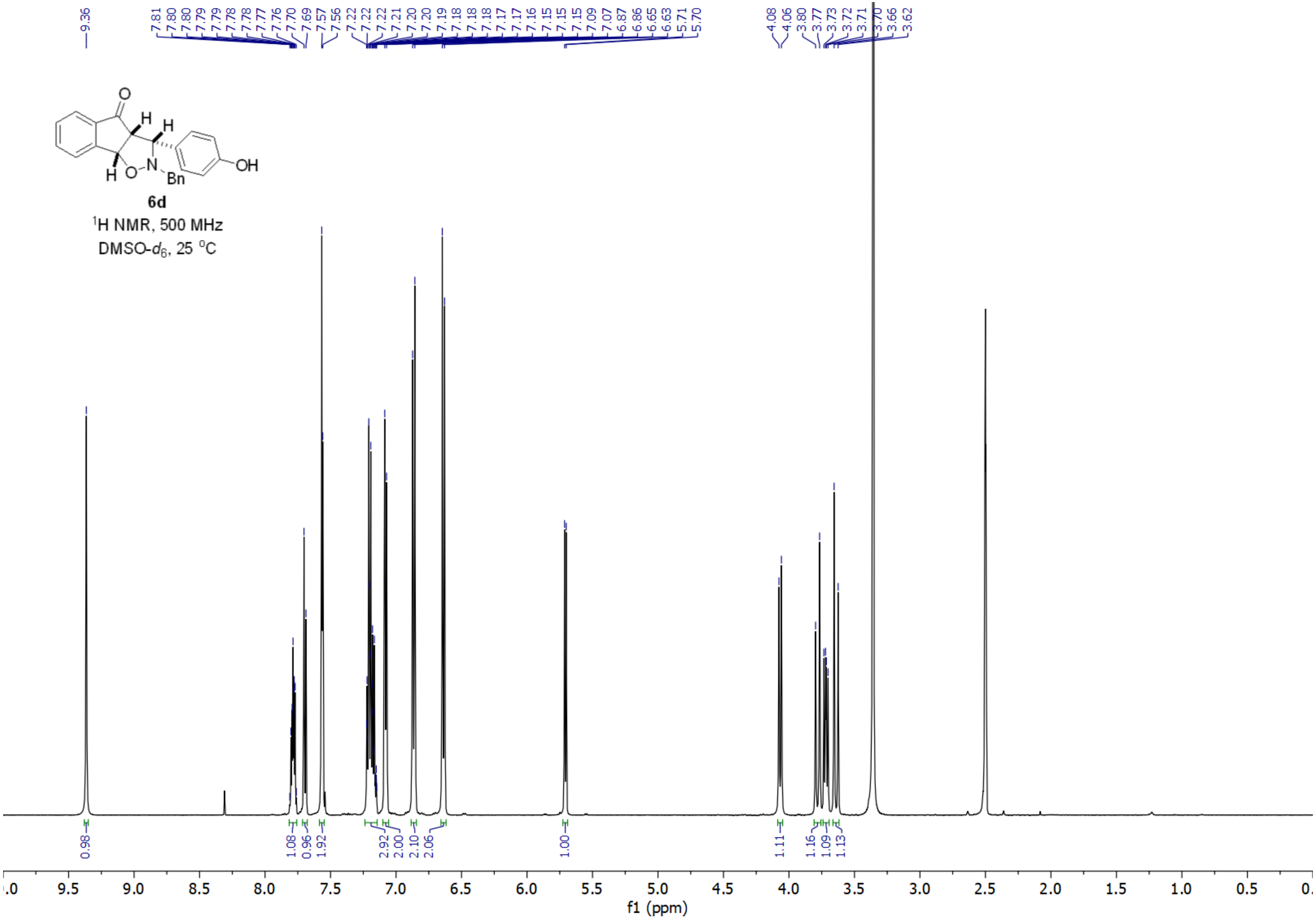
6d
1H NMR, 500 MHz
DMSO-d6, 25 °C
f1 (ppm)

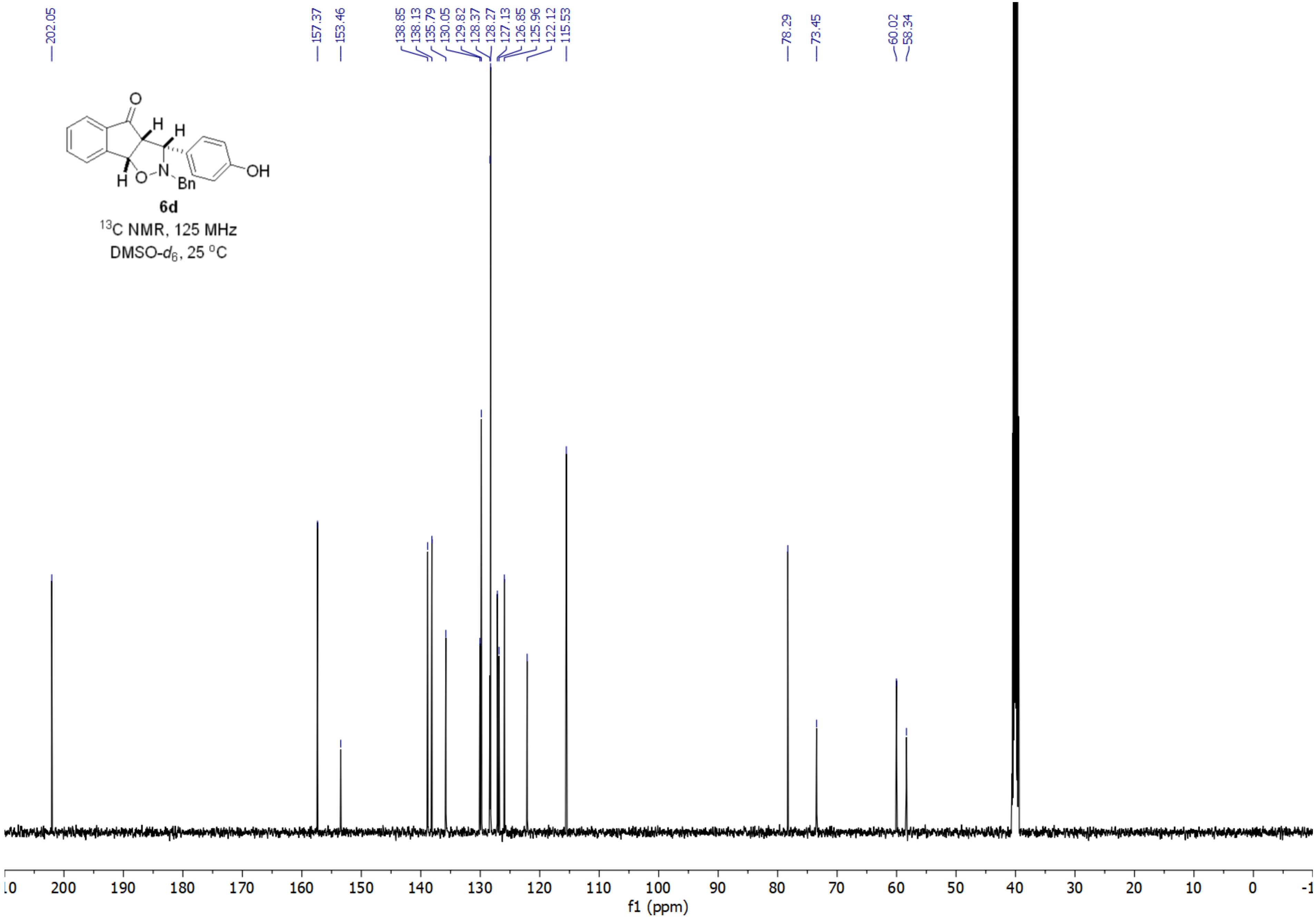

6d

^{13}C NMR, 125 MHz

DMSO-d_6, 25 °C

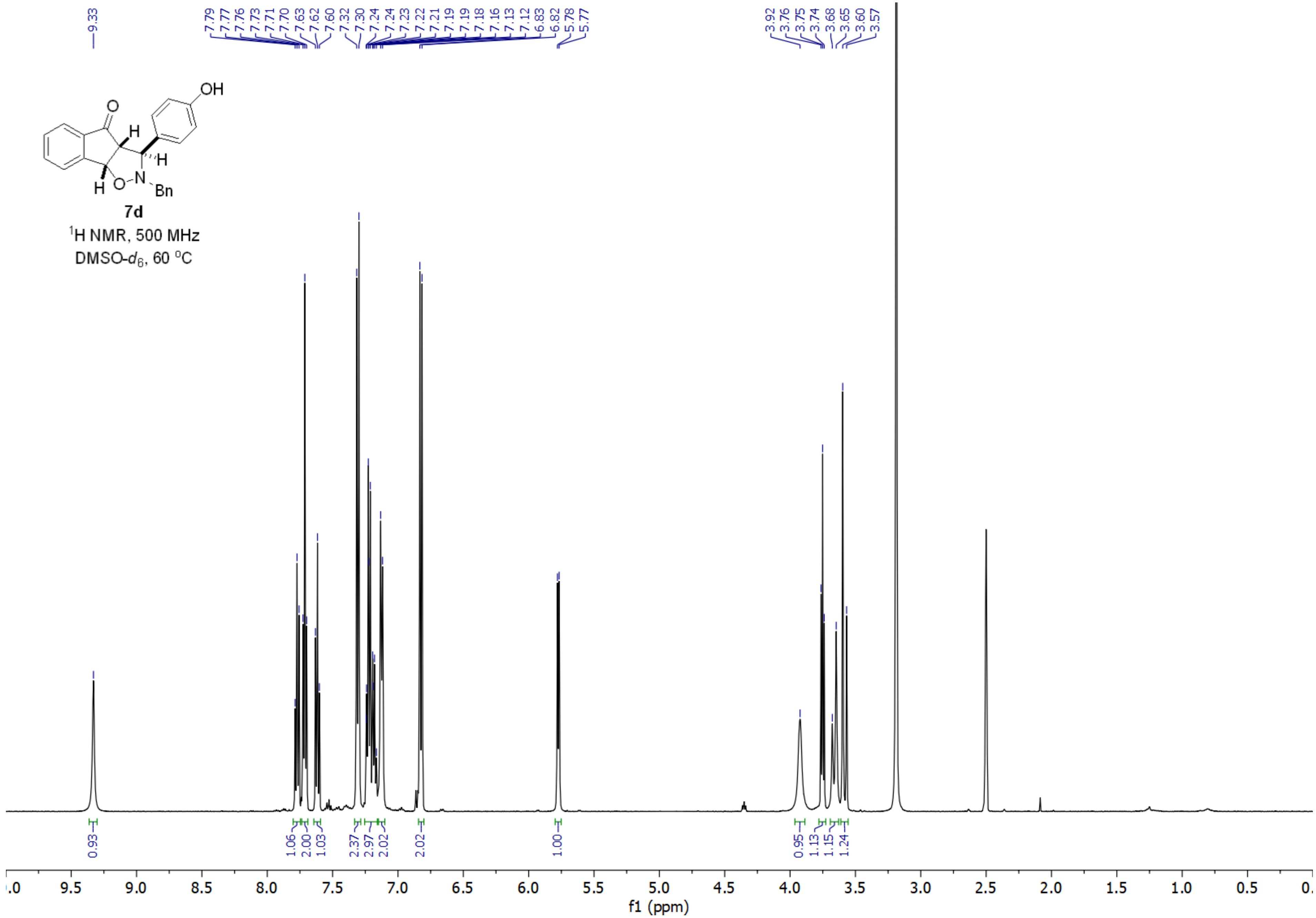
7d
1H NMR, 500 MHz
DMSO-d6, 60 °C
f1 (ppm)

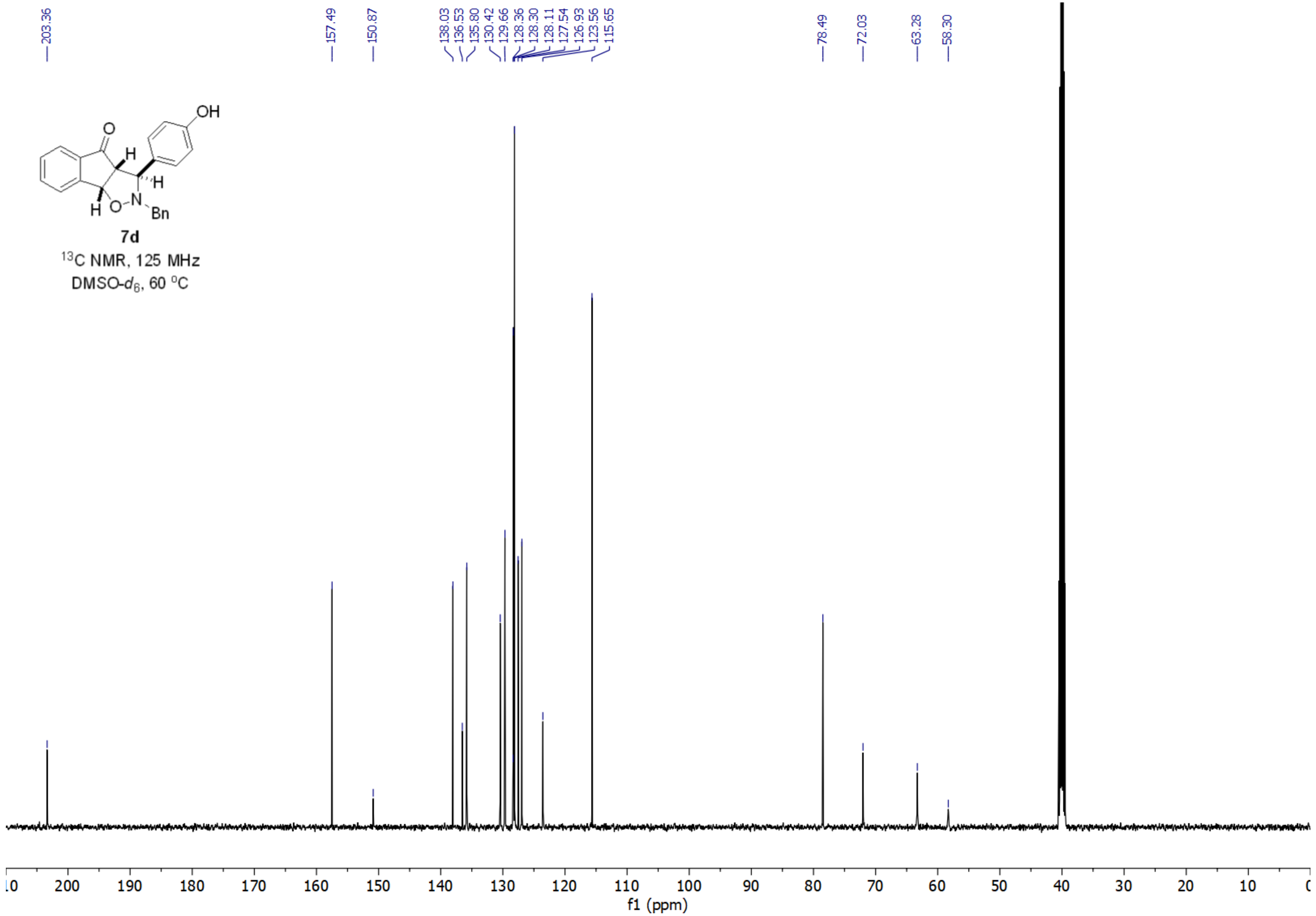
203.36
157.49
150.87
138.03
136.53
135.80
130.42
129.66
128.36
128.30
128.11
127.54
126.93
123.56
115.65
78.49
72.03
63.28
58.30
OH
O
H
H
H
O
N
Bn
7d
^{13}C NMR, 125 MHz
DMSO-d_6, 60 °C
200
190
180
170
160
150
140
130
120
110
100
90
80
70
60
50
40
30
20
10
f1 (ppm)

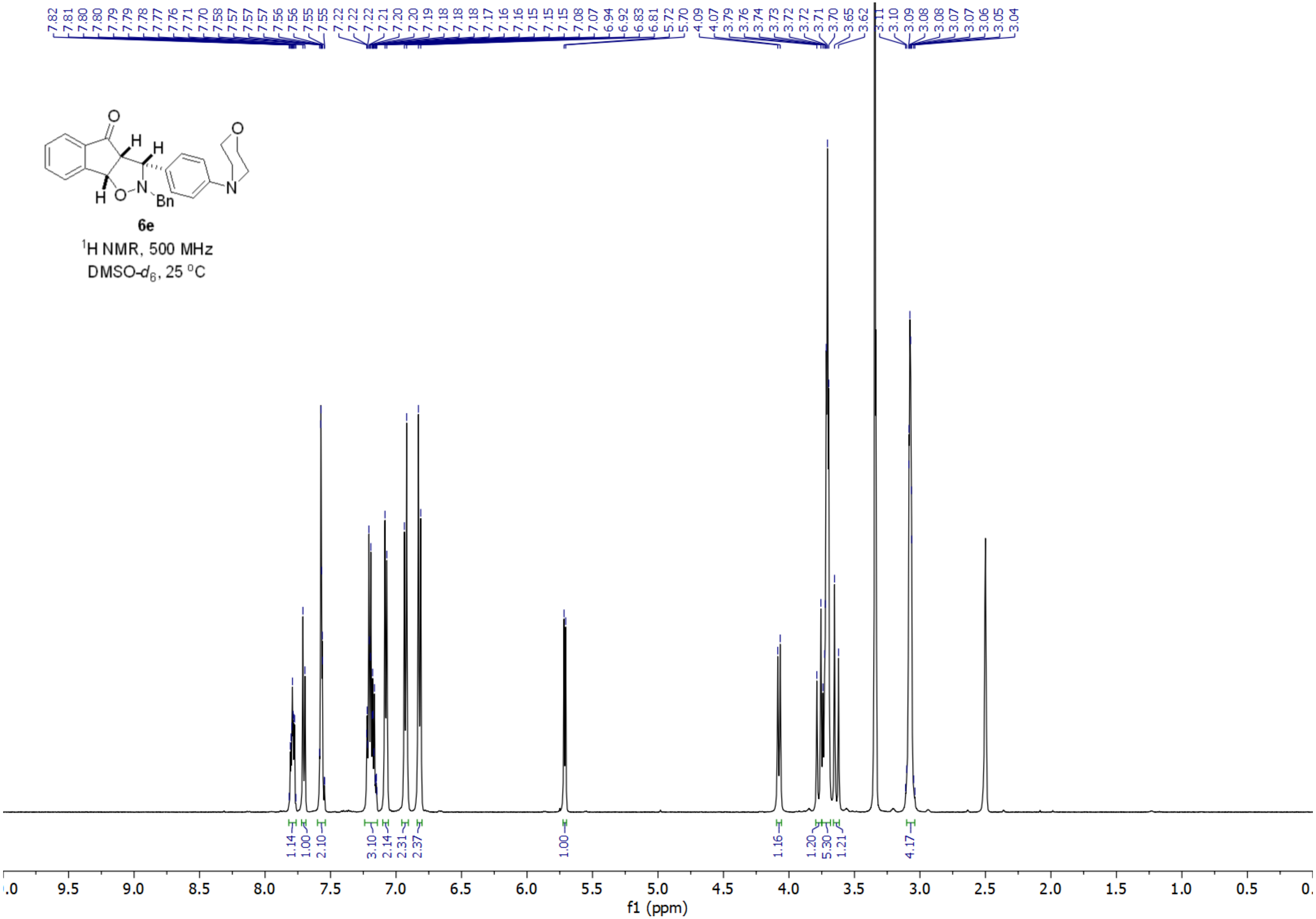
6e
1H NMR, 500 MHz
DMSO-d6, 25 °C
f1 (ppm)

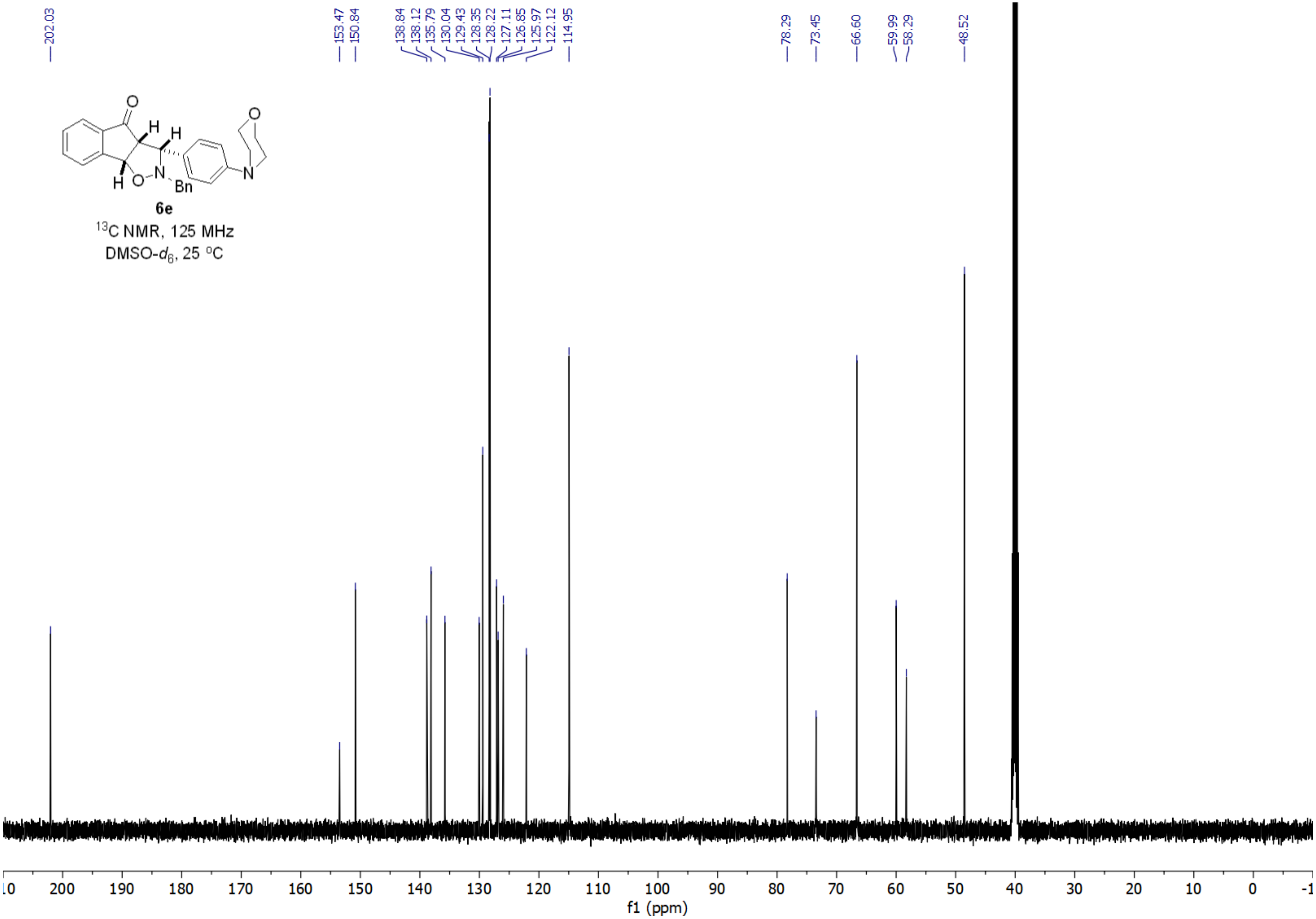
202.03
153.47
150.84
138.84
138.12
135.79
130.04
129.43
128.35
128.22
127.11
126.85
125.97
122.12
114.95
78.29
73.45
66.60
59.99
58.29
48.52
6e
^{13}C NMR, 125 MHz
DMSO-d_6, 25 °C
f1 (ppm)

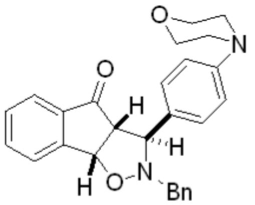

7e

^{1}H NMR, 500 MHz

DMSO-d_6, 60 °C

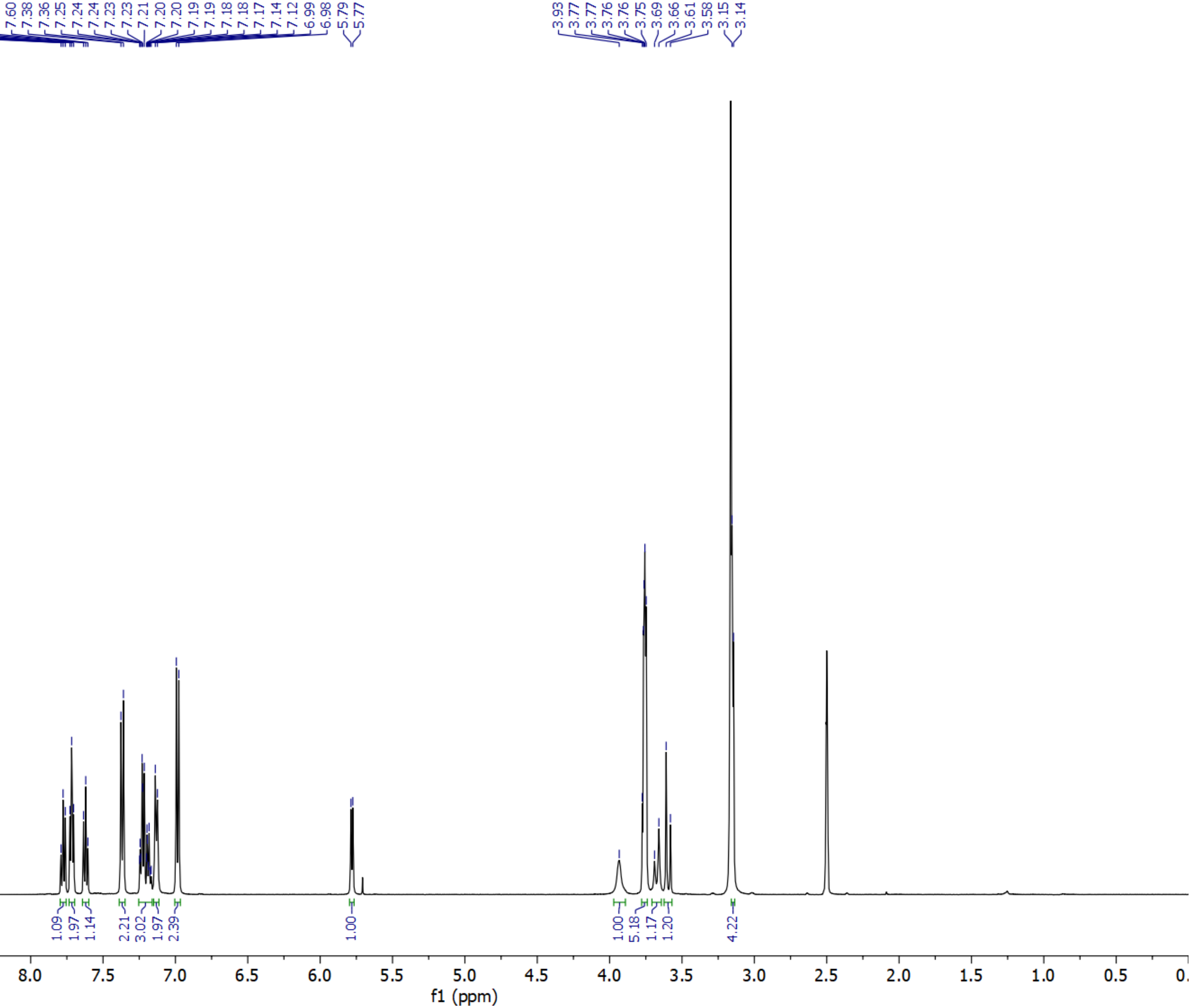

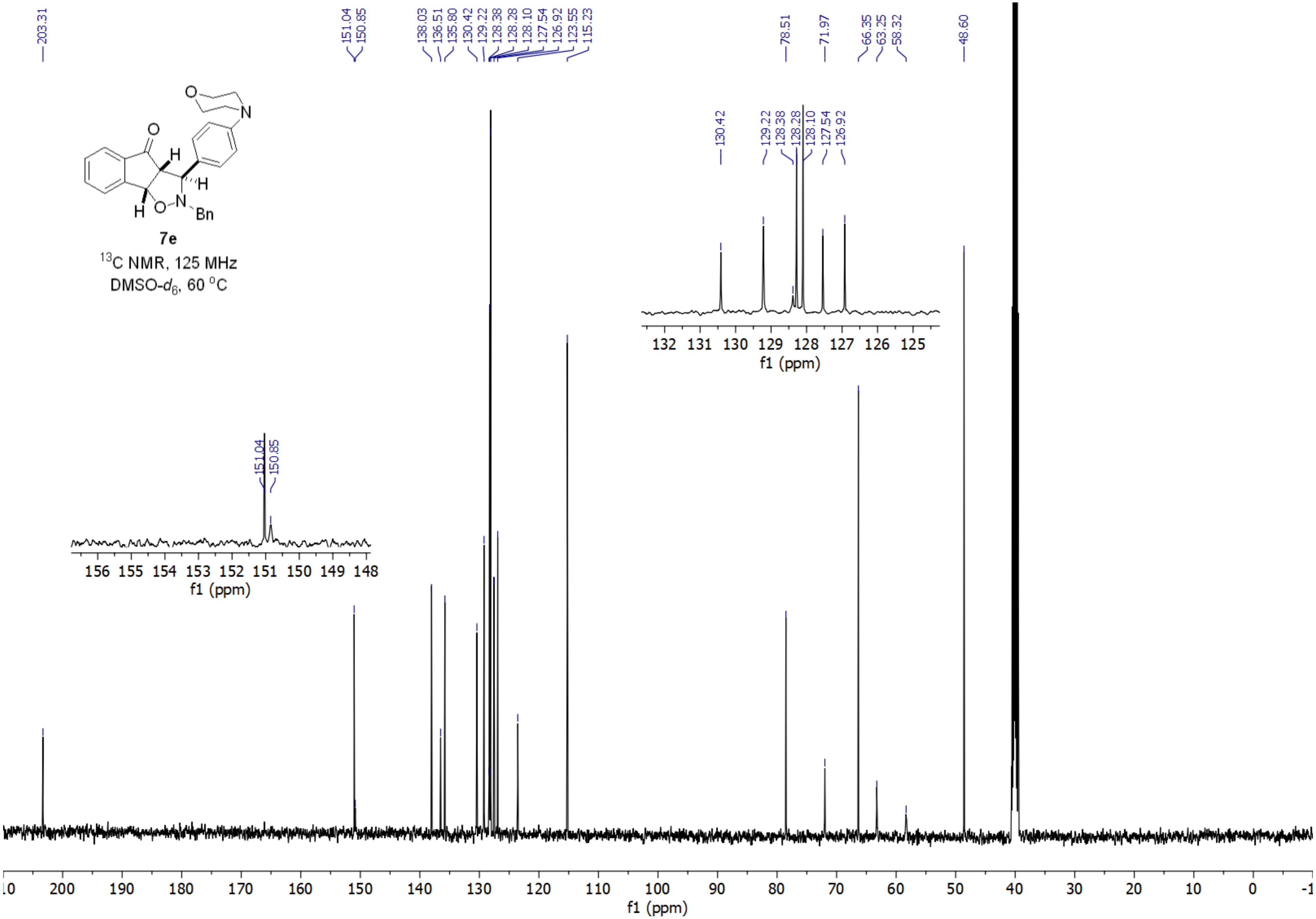
203.31
151.04
150.85
138.03
136.51
135.80
130.42
129.22
128.38
128.28
128.10
127.54
126.92
123.55
115.23
78.51
71.97
66.35
63.25
58.32
48.60
O
N
O
H
H
H
O
N
Bn
7e
^{13}C NMR, 125 MHz
DMSO-d_6, 60 °C
f1 (ppm)

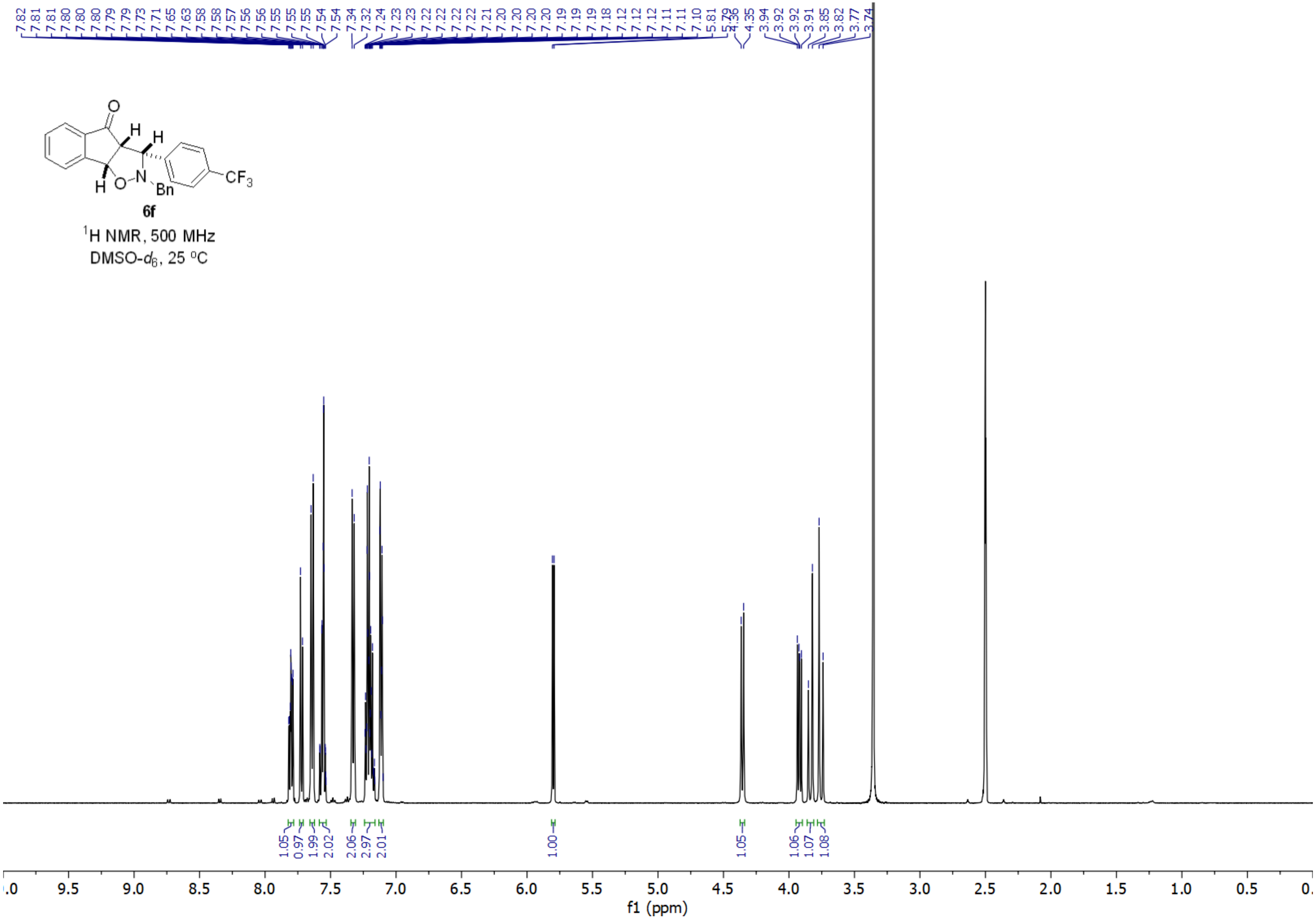
7.82 7.81 7.81 7.80 7.80 7.80 7.79 7.79 7.73 7.71 7.65 7.63 7.58 7.58 7.57 7.56 7.56 7.55 7.55 7.55 7.54 7.54 7.34 7.32 7.24 7.23 7.23 7.22 7.22 7.22 7.22 7.21 7.20 7.20 7.20 7.20 7.19 7.19 7.19 7.18 7.12 7.12 7.12 7.11 7.11 7.10 5.81 5.79 4.36 4.35 3.94 3.92 3.92 3.91 3.85 3.82 3.77 3.74
O
H
H
H
O
N
Bn
CF_3
6f
^{1}H NMR, 500 MHz
DMSO-d_6, 25 °C
1.05 0.97 1.99 2.02 2.06 2.97 2.01 1.00 1.05 1.06 1.07 1.08
.0 9.5 9.0 8.5 8.0 7.5 7.0 6.5 6.0 5.5 5.0 4.5 4.0 3.5 3.0 2.5 2.0 1.5 1.0 0.5 0.
f1 (ppm)

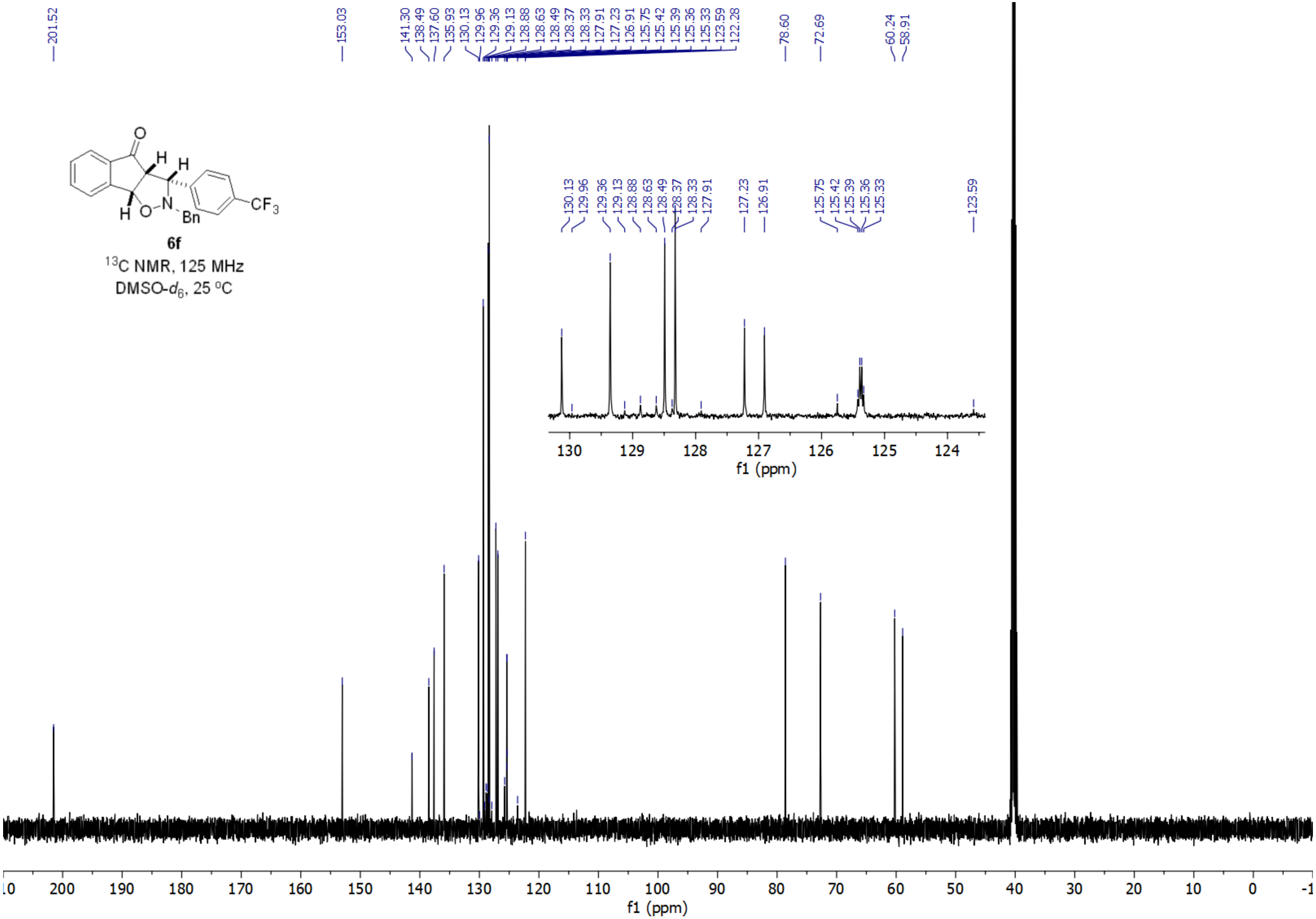

6f

^{13}C NMR, 125 MHz

DMSO-d_6, 25 °C

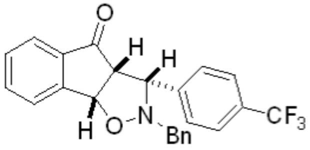

6f

^{19}F NMR, 470 MHz

DMSO-d_6, 25 ^{o}C

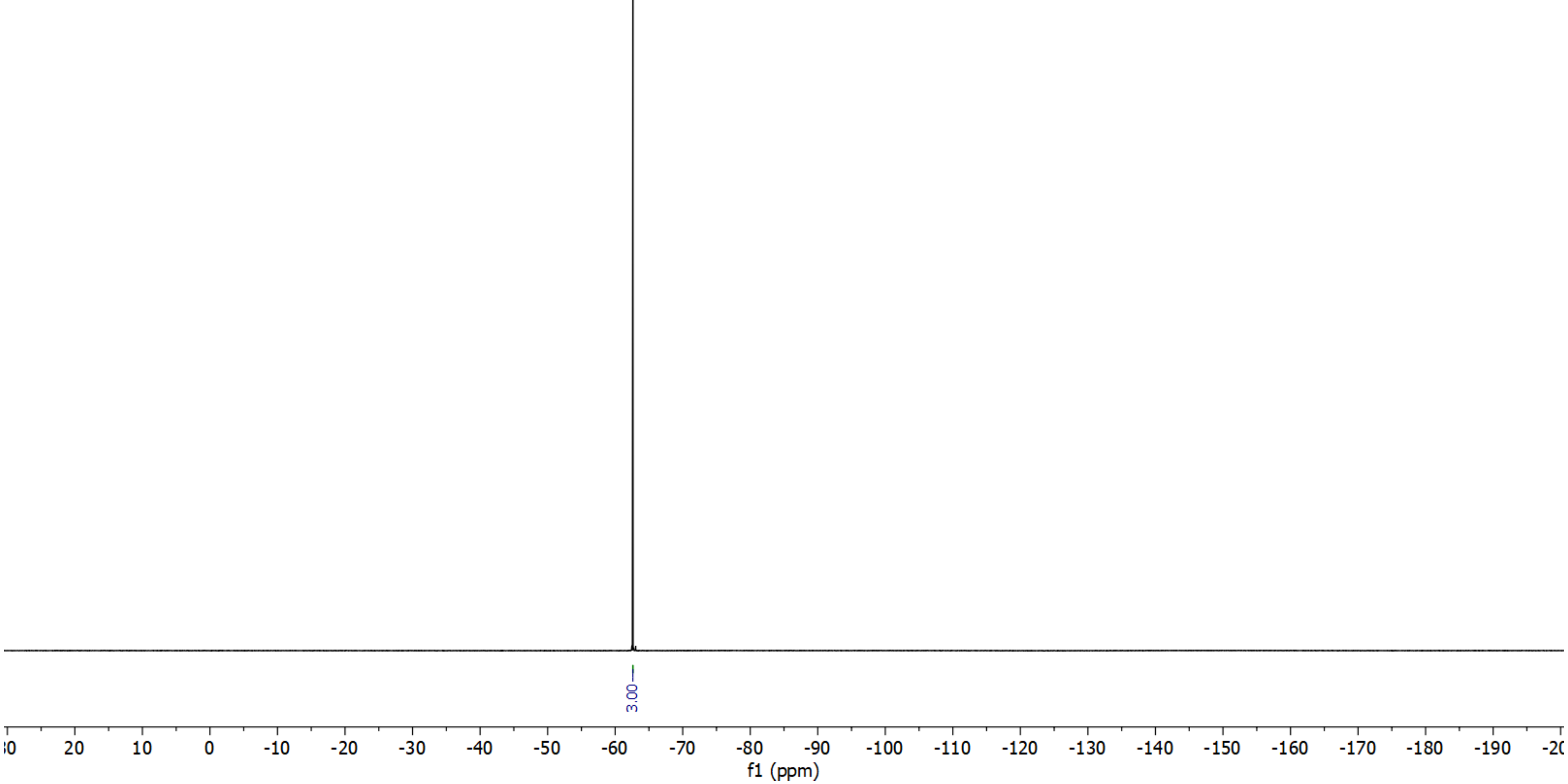

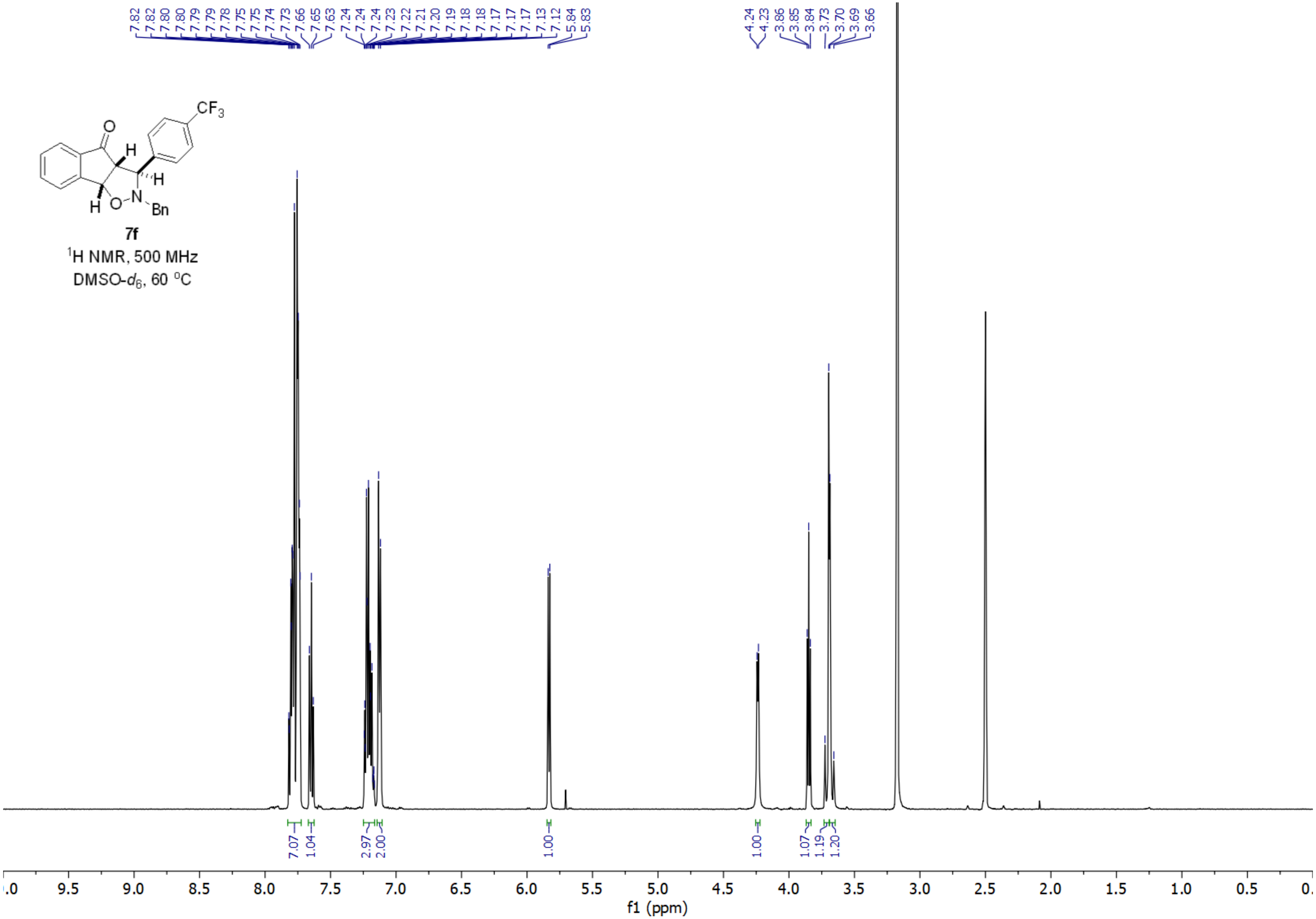

7f
¹H NMR, 500 MHz
DMSO-d6, 60 °C
f1 (ppm)

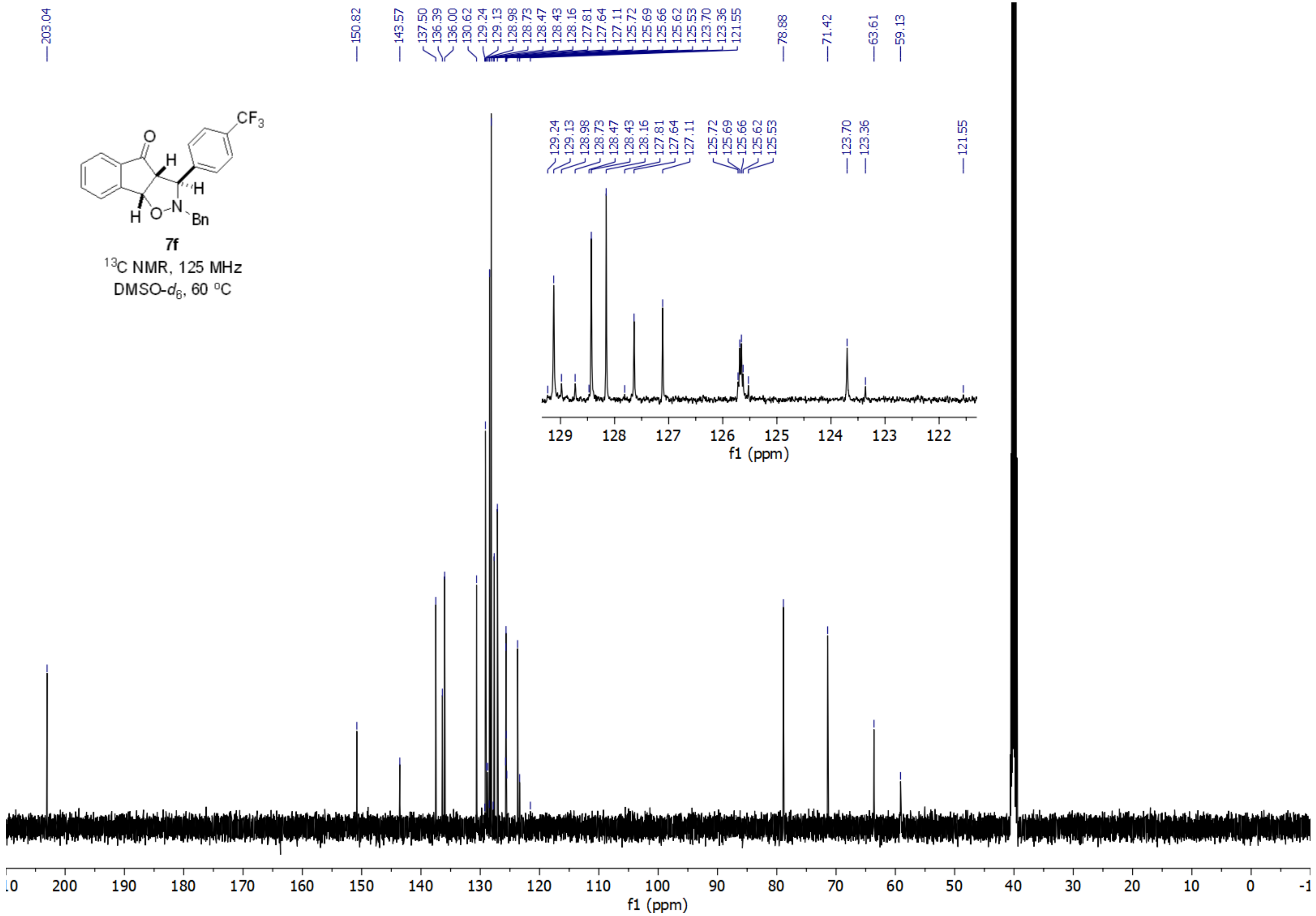

203.04
150.82
143.57
137.50
136.39
136.00
130.62
129.24
129.13
128.98
128.73
128.47
128.43
128.16
127.81
127.64
127.11
125.72
125.69
125.66
125.62
125.53
123.70
123.36
121.55
78.88
71.42
63.61
59.13
CF3
O
H
H
H
O
N
Bn
7f
13C NMR, 125 MHz
DMSO-d6, 60 °C
f1 (ppm)

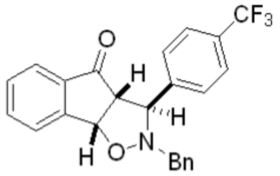

7f

^{19}F NMR, 470 MHz

DMSO-d_6, 25 °C

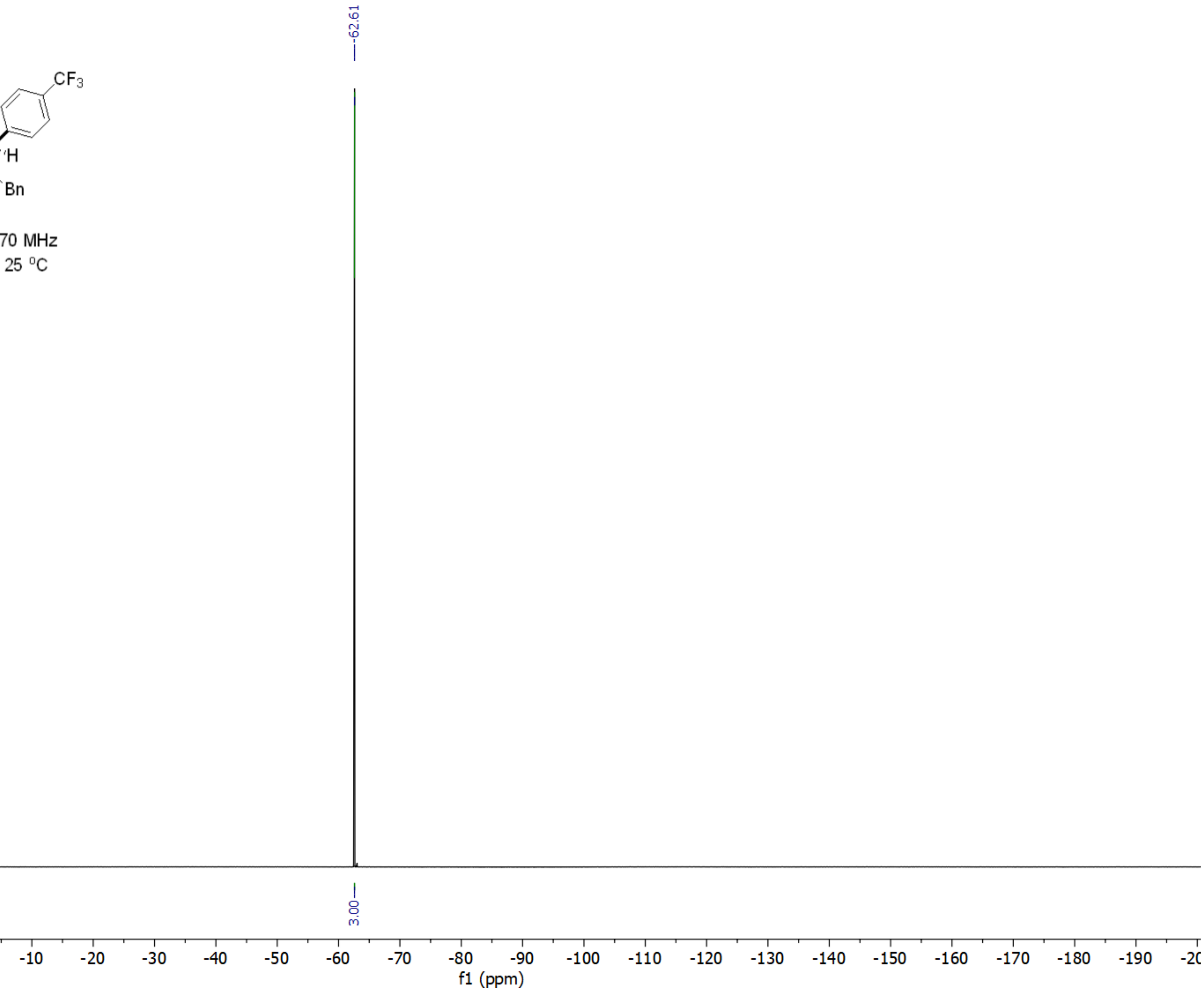

6g

^{1}H NMR, 500 MHz

DMSO-d_6, 25 ^{o}C

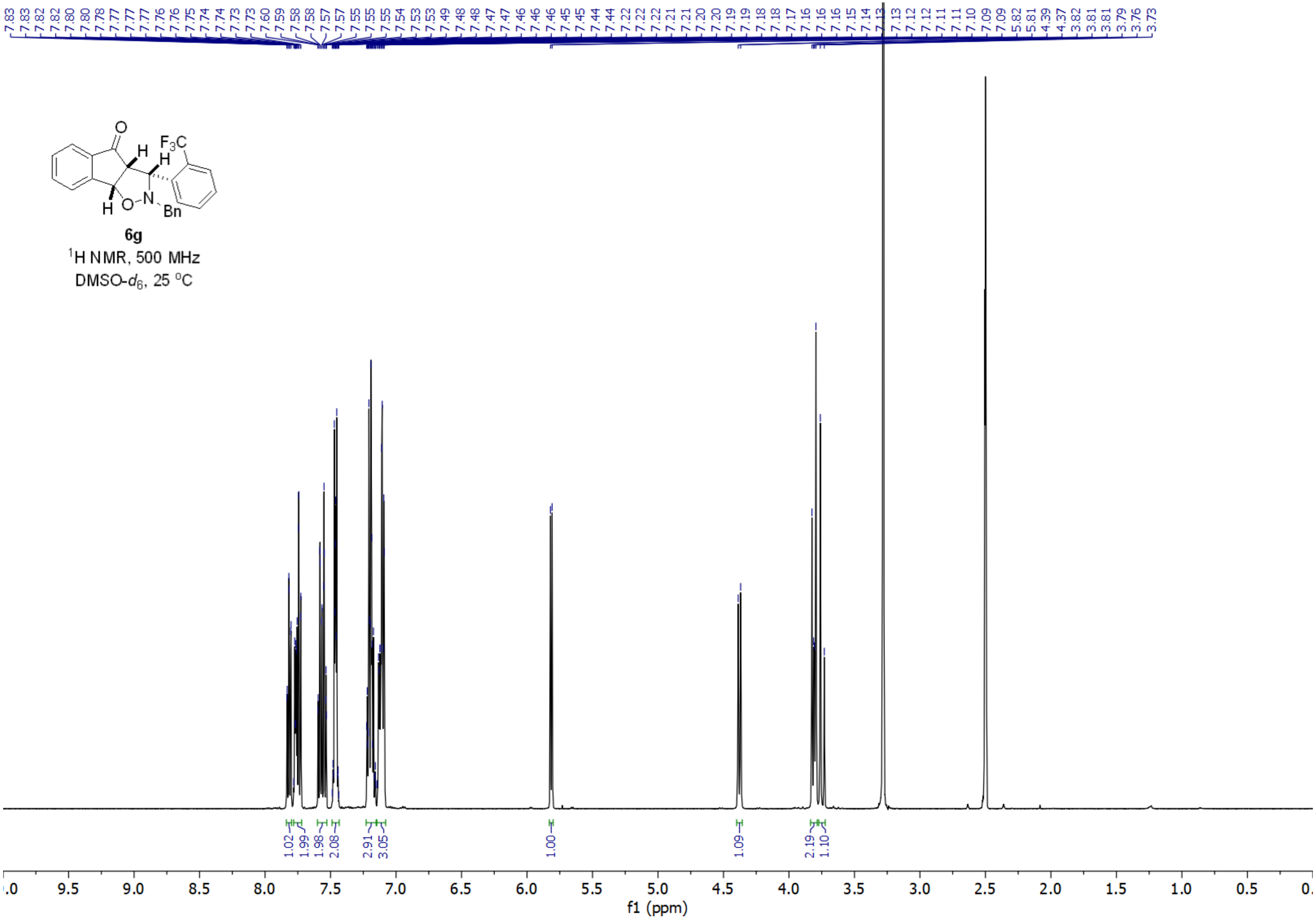

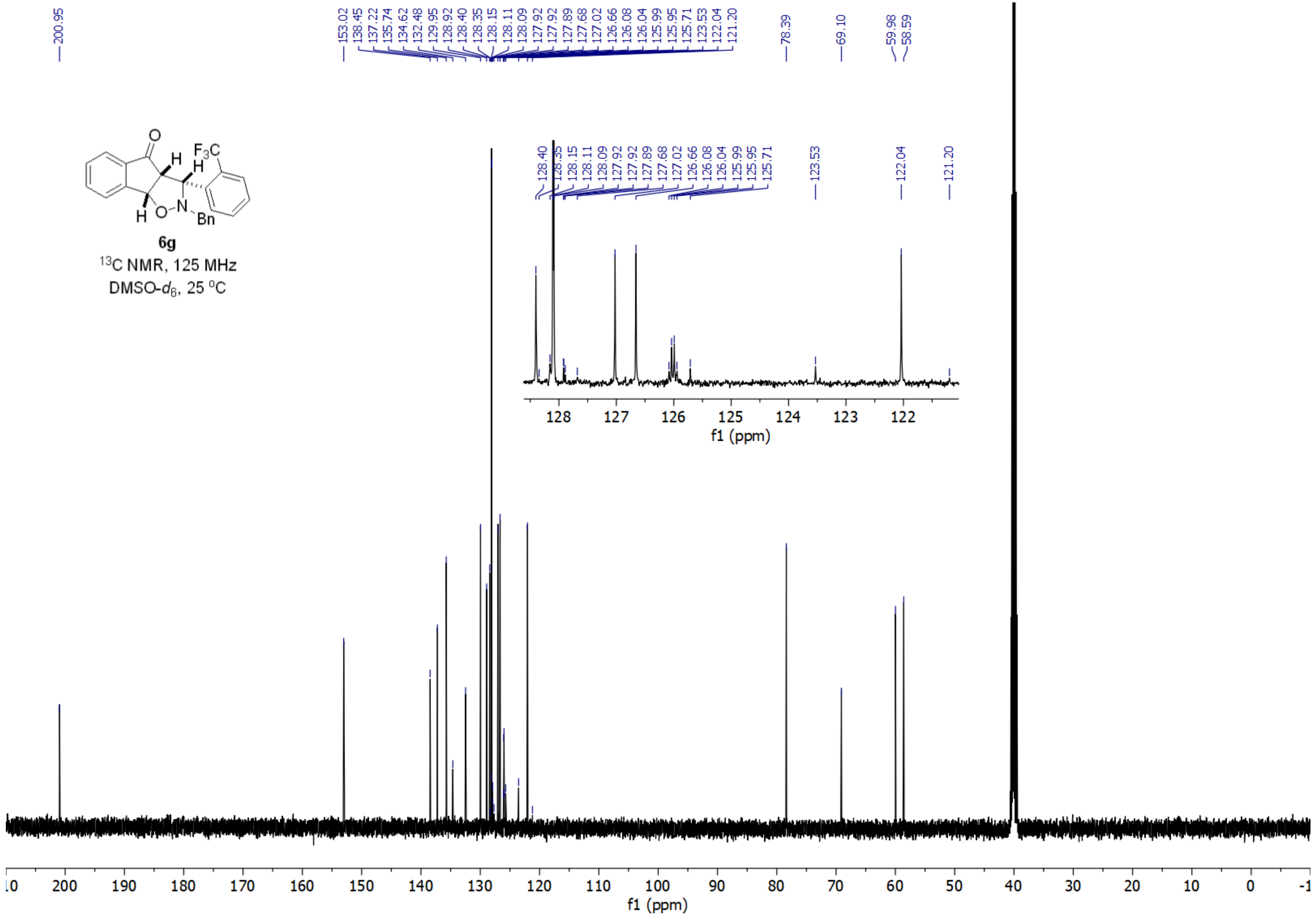

6g

^{13}C NMR, 125 MHz

DMSO-d_6, 25 ^{o}C

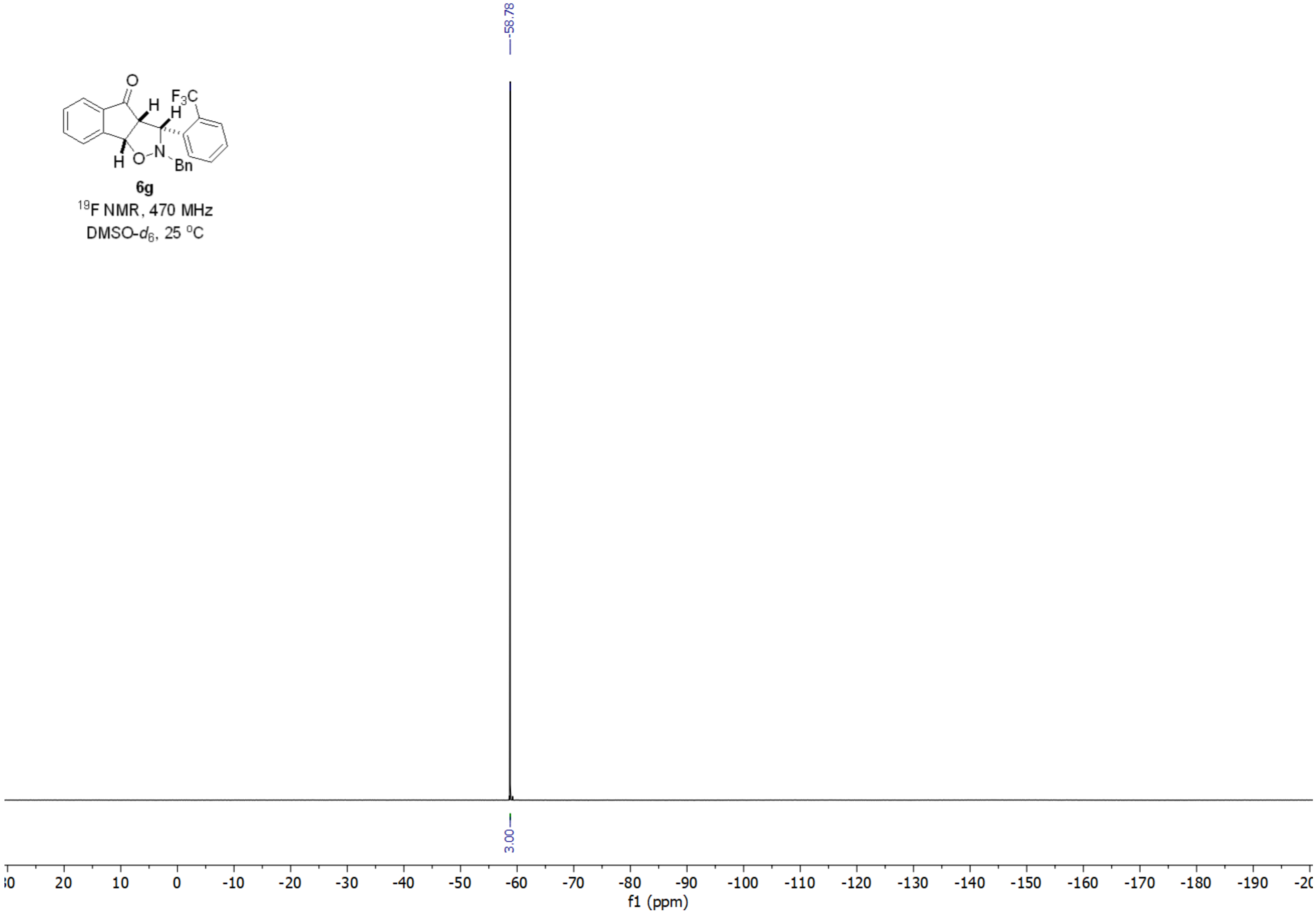

6g

^{19}F NMR, 470 MHz

DMSO-d_6, 25 °C

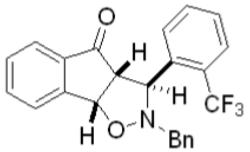

7g

^1H NMR, 500 MHz

DMSO-d_6, 60 °C

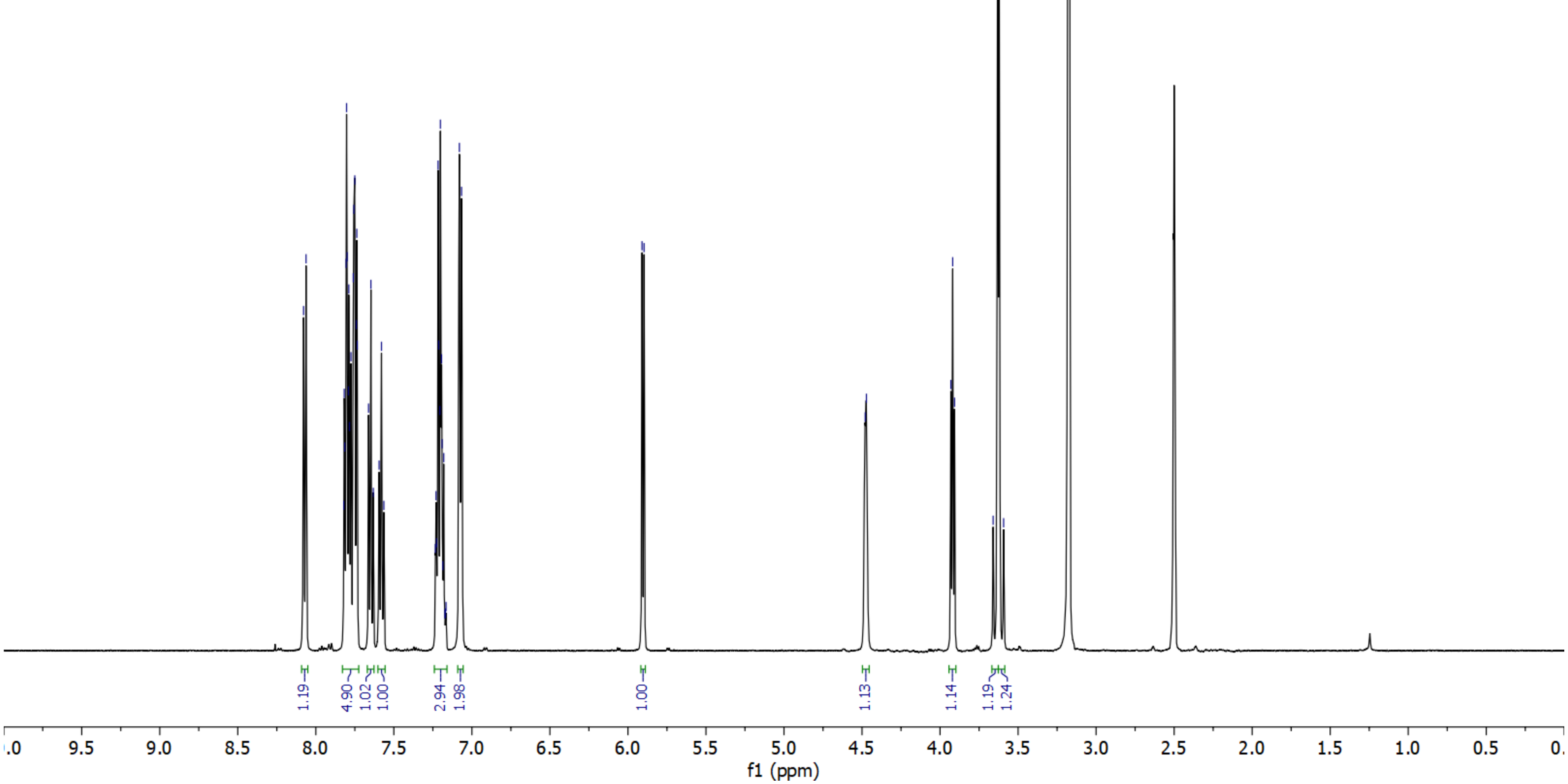

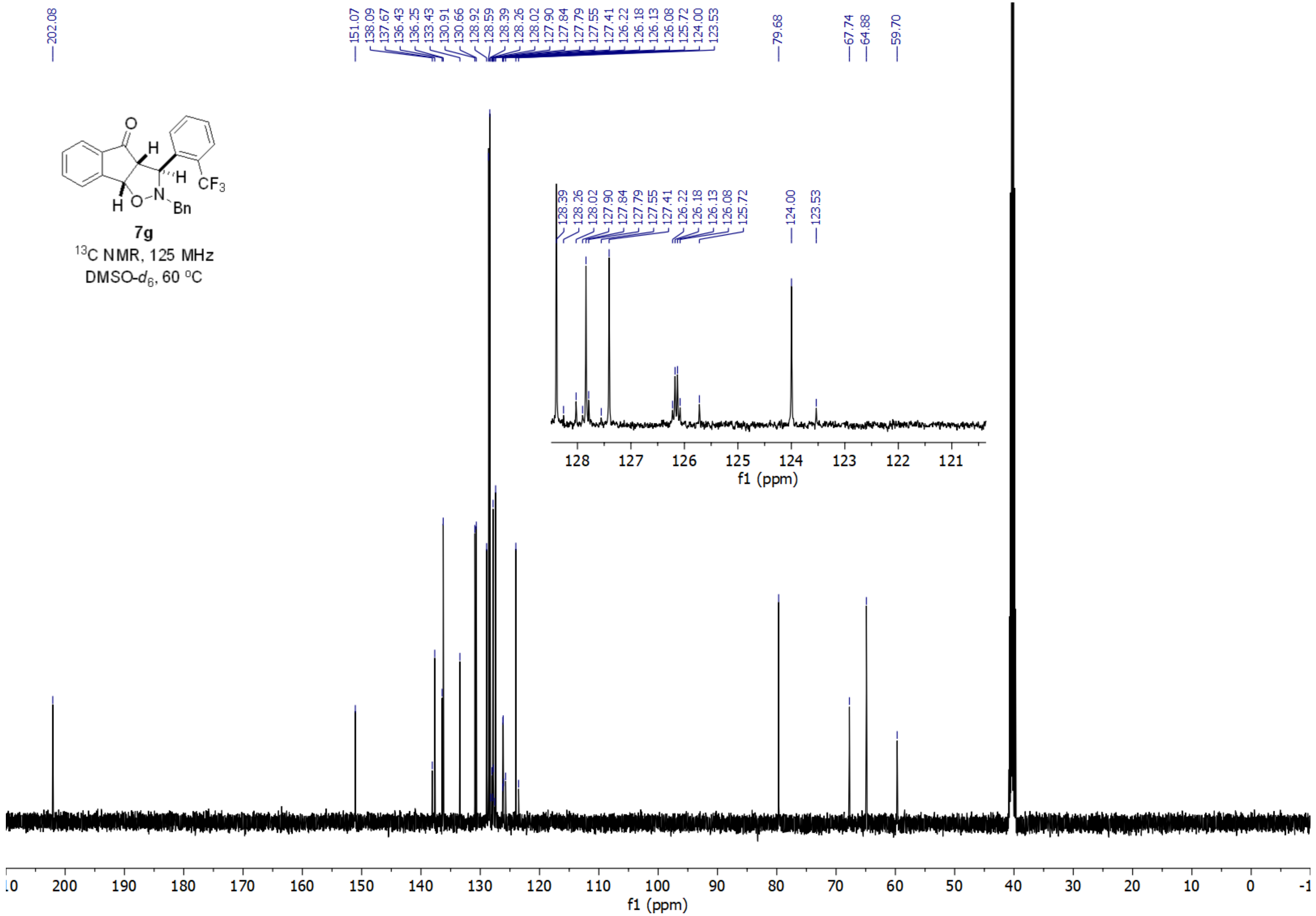

202.08
151.07
138.09
137.67
136.43
136.25
133.43
130.91
130.66
128.92
128.59
128.39
128.26
128.02
127.90
127.84
127.79
127.55
127.41
126.22
126.18
126.13
126.08
125.72
124.00
123.53
79.68
67.74
64.88
59.70
O
H
H
CF3
H
O
N
Bn
7g
13C NMR, 125 MHz
DMSO-d6, 60 °C
f1 (ppm)

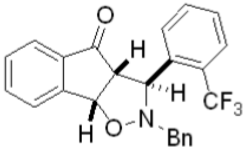

7g

^{19}F NMR, 470 MHz

DMSO-d_6, 25 °C

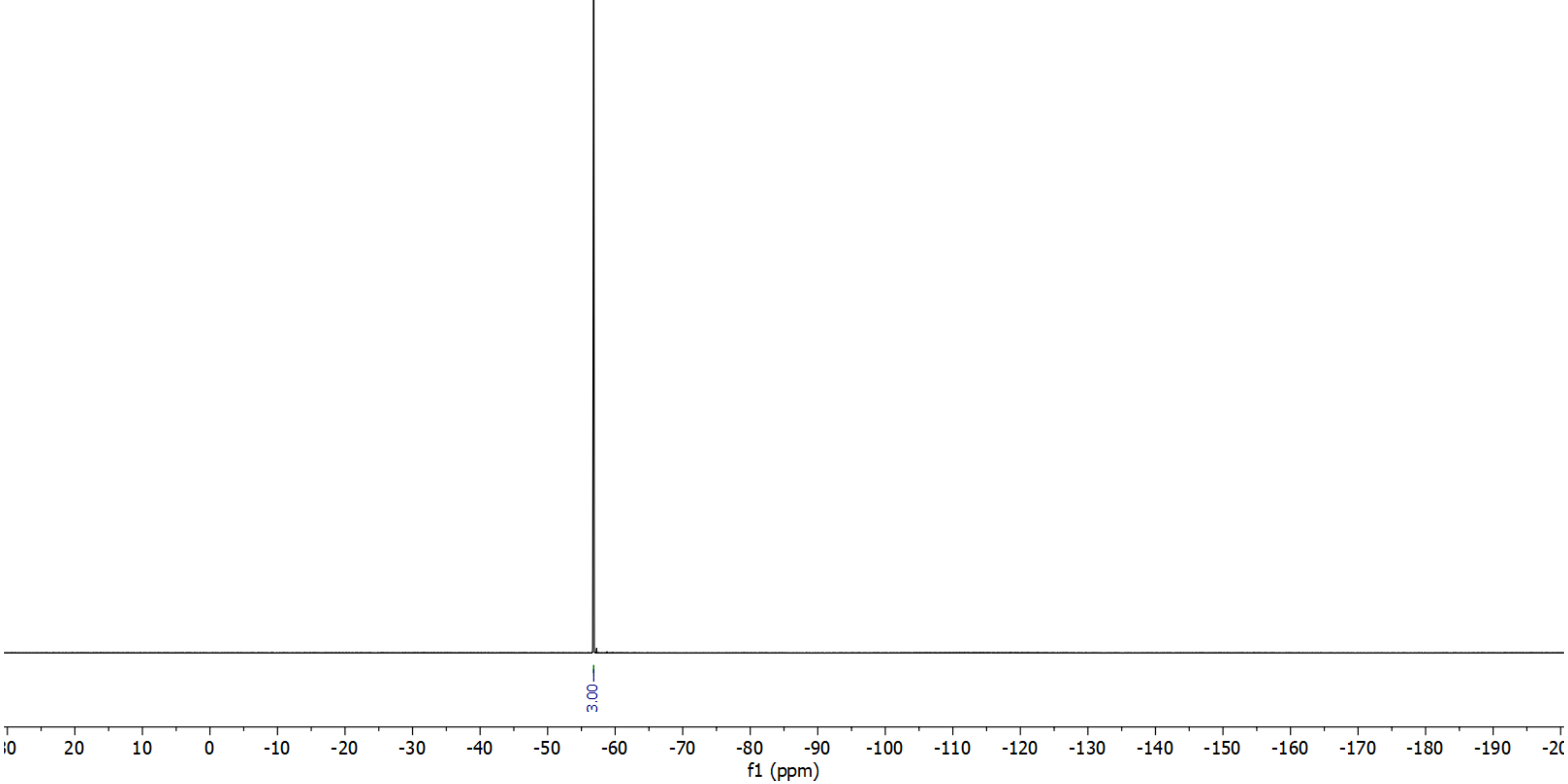

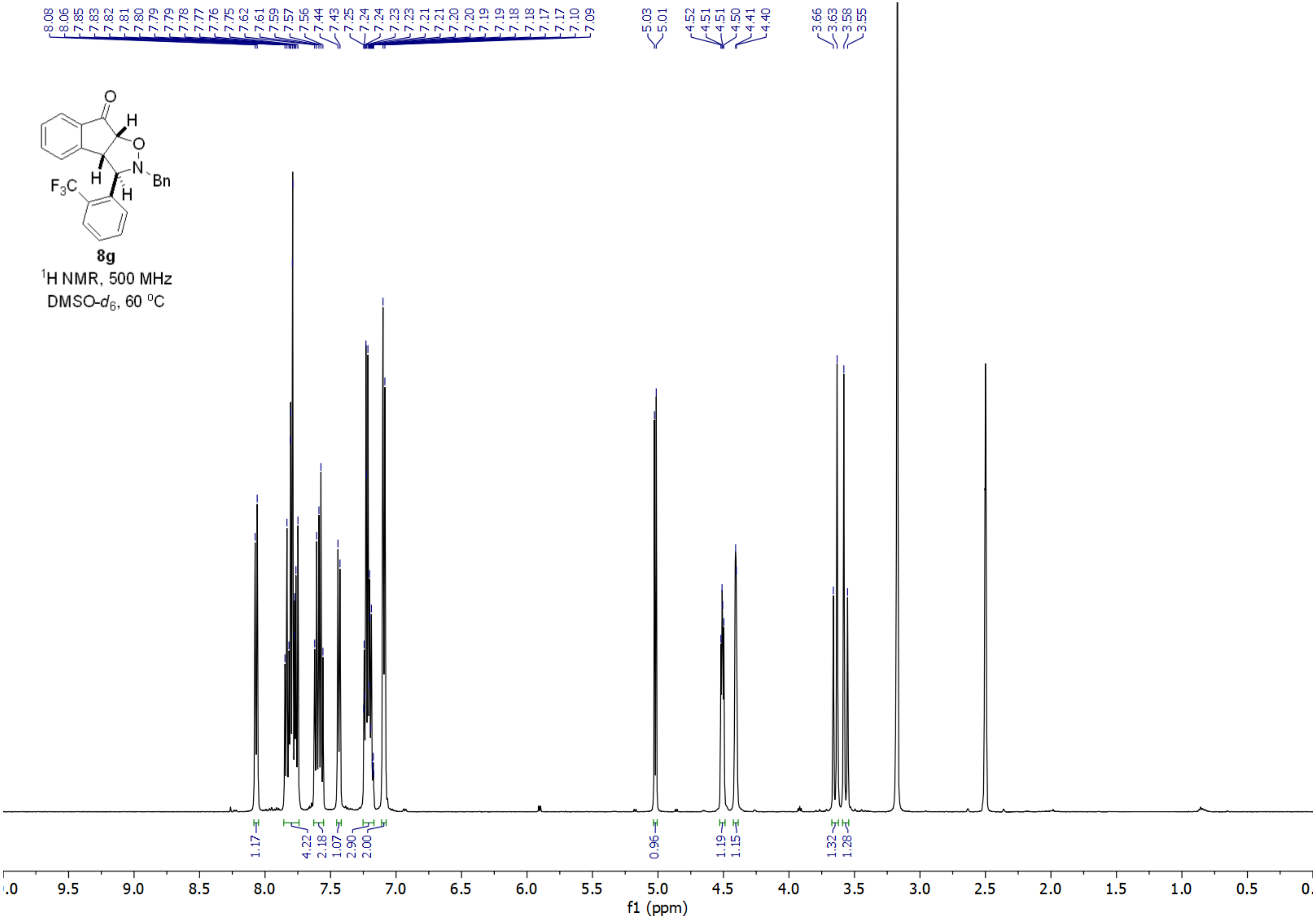

8g

^{1}H NMR, 500 MHz

DMSO-d_6, 60 °C

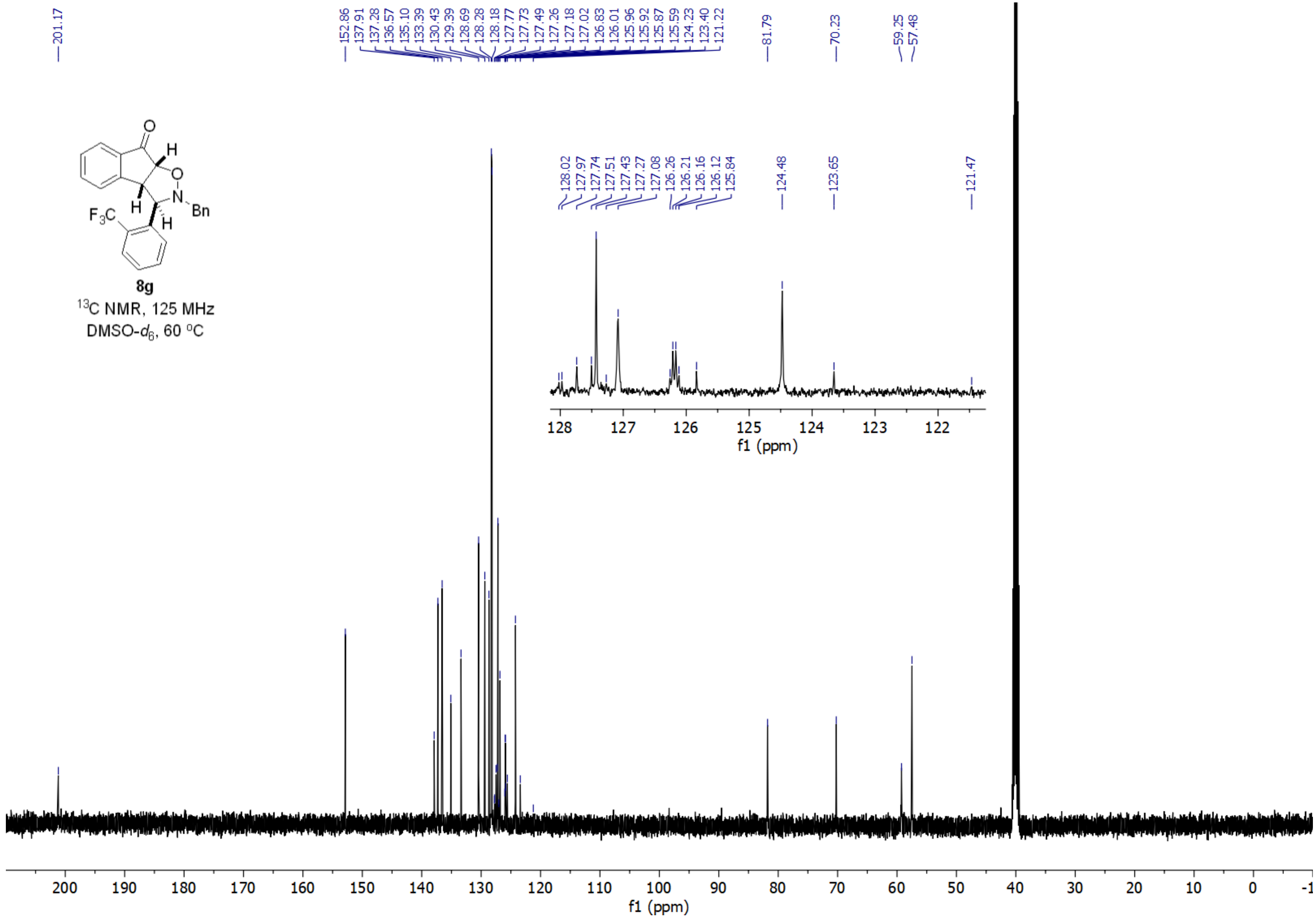

201.17
152.86
137.91
137.28
136.57
135.10
133.39
130.43
129.39
128.69
128.28
128.18
127.77
127.73
127.49
127.26
127.18
127.02
126.83
126.01
125.96
125.92
125.87
125.59
124.23
123.40
121.22
81.79
70.23
59.25
57.48
O
H
O
N
Bn
H
H
F_3C
8g
^{13}C NMR, 125 MHz
DMSO-d_6, 60 °C
128.02
127.97
127.74
127.51
127.43
127.27
127.08
126.26
126.21
126.16
126.12
125.84
124.48
123.65
121.47
128
127
126
125
124
123
122
f1 (ppm)
200
190
180
170
160
150
140
130
120
110
100
90
80
70
60
50
40
30
20
10
0
-1
f1 (ppm)

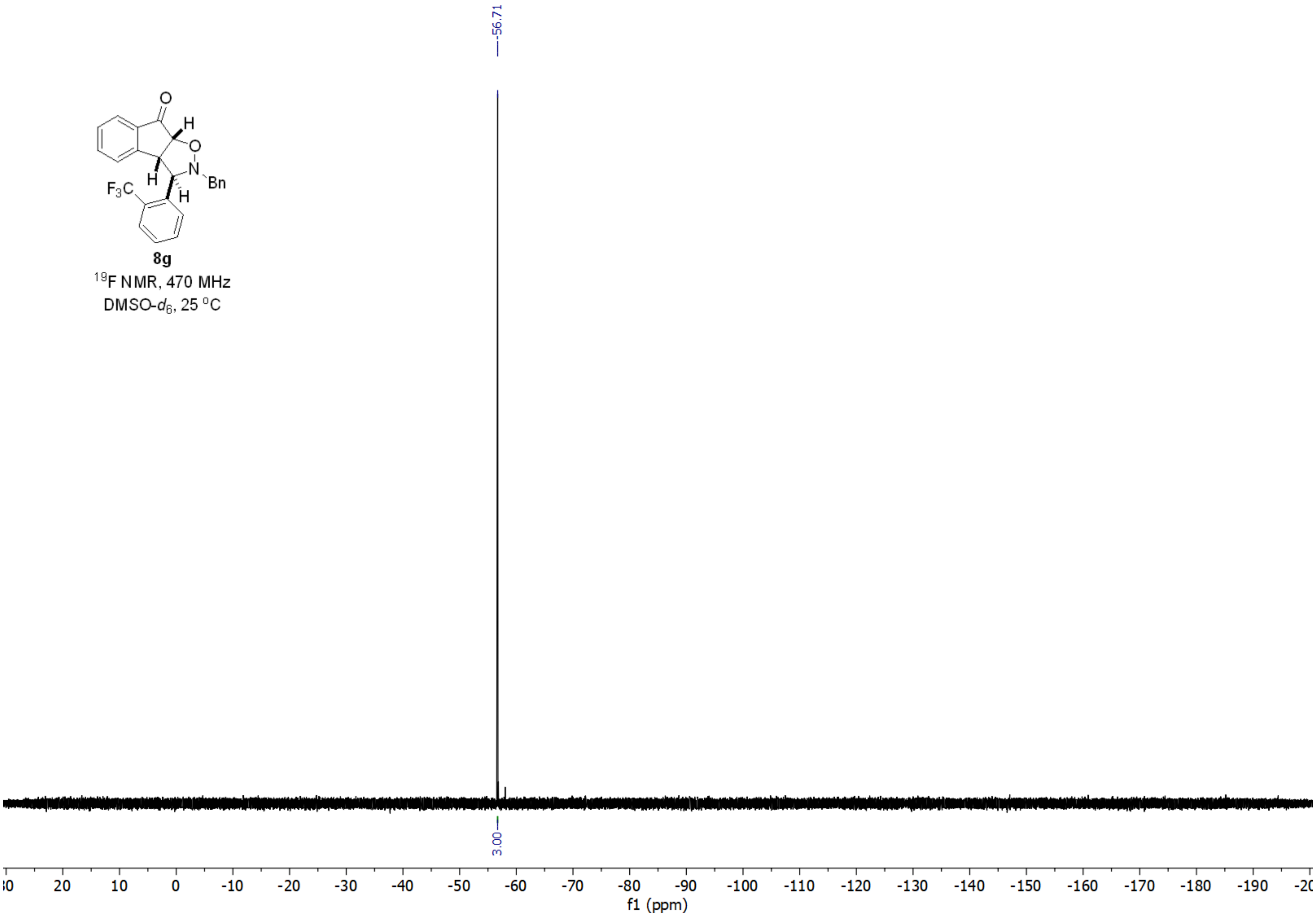
-56.71
8g
^{19}F NMR, 470 MHz
DMSO-d_6, 25 °C
3.00
f1 (ppm)

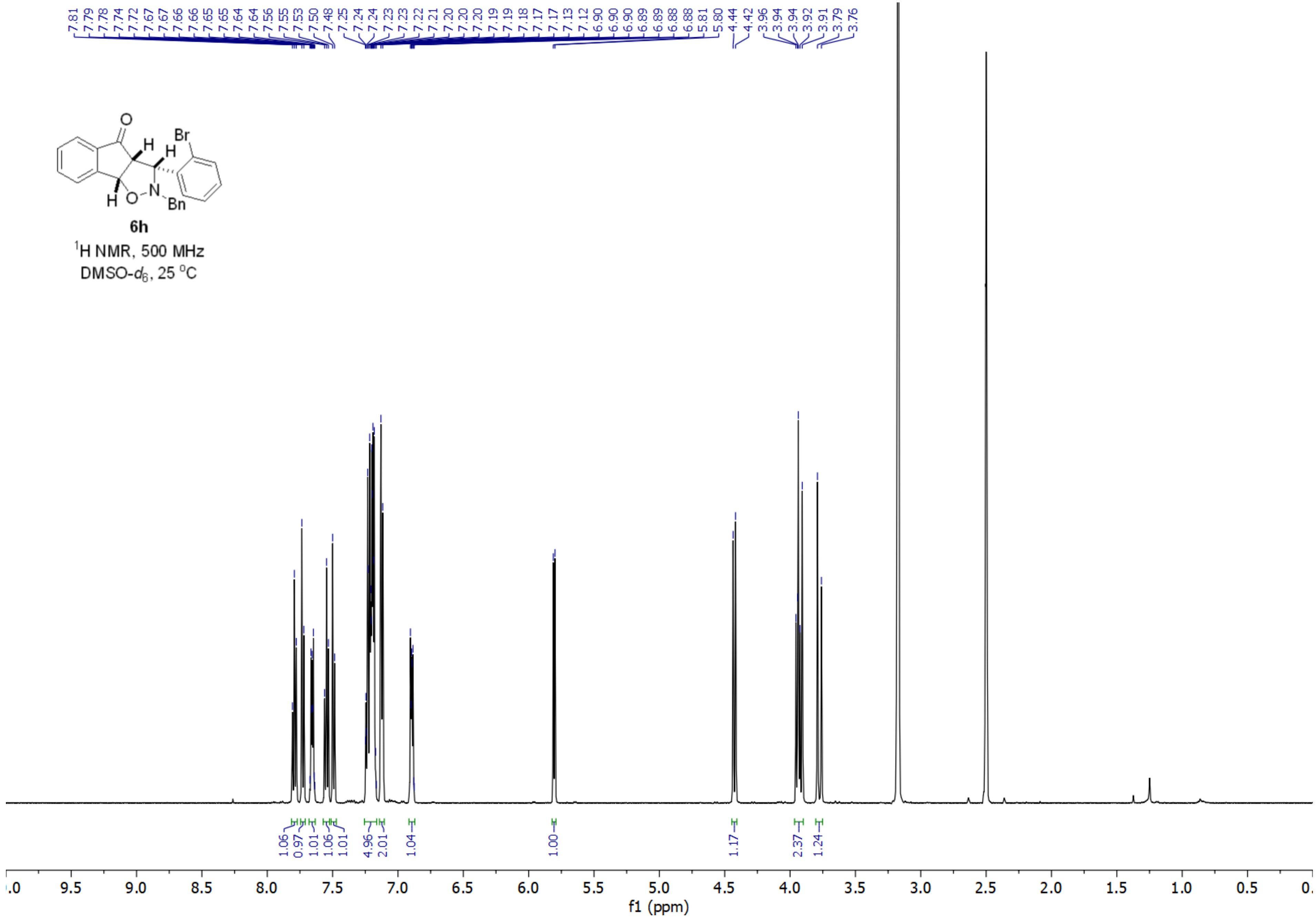
7.81 7.79 7.78 7.74 7.72 7.67 7.67 7.66 7.66 7.65 7.65 7.64 7.64 7.56 7.55 7.53 7.50 7.48 7.25 7.24 7.24 7.23 7.23 7.22 7.21 7.20 7.20 7.20 7.19 7.19 7.18 7.17 7.17 7.13 7.12 6.90 6.90 6.90 6.89 6.89 6.88 6.88 5.81 5.80 4.44 4.42 3.96 3.94 3.94 3.92 3.91 3.79 3.76
O
H
H
Br
H
O
N
Bn
6h
1H NMR, 500 MHz
DMSO-d6, 25 °C
1.06 0.97 1.01 1.06 1.01 4.96 2.01 1.04 1.00 1.17 2.37 1.24
9.5 9.0 8.5 8.0 7.5 7.0 6.5 6.0 5.5 5.0 4.5 4.0 3.5 3.0 2.5 2.0 1.5 1.0 0.5
f1 (ppm)

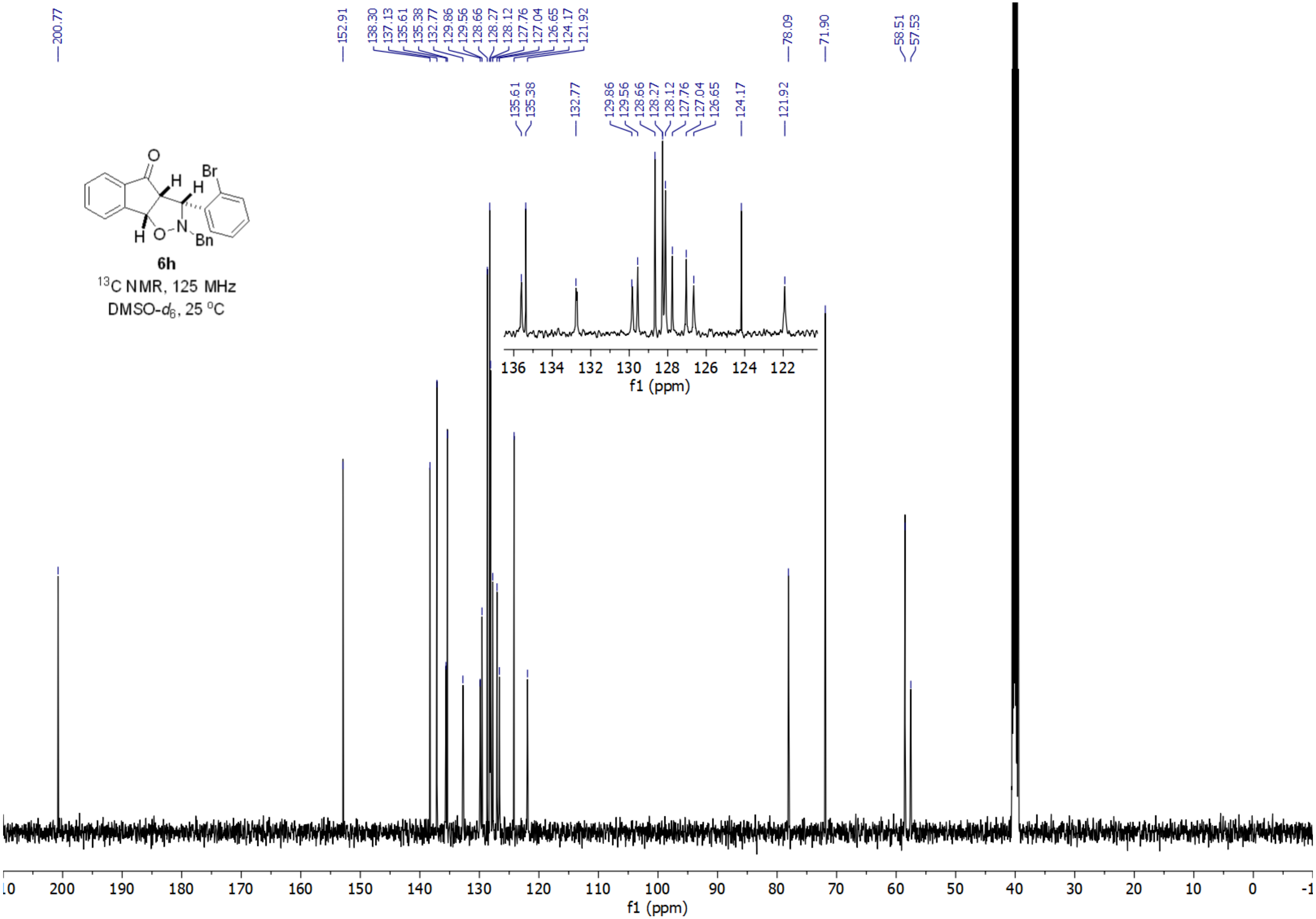

6h

^{13}C NMR, 125 MHz

DMSO-d_6, 25 °C

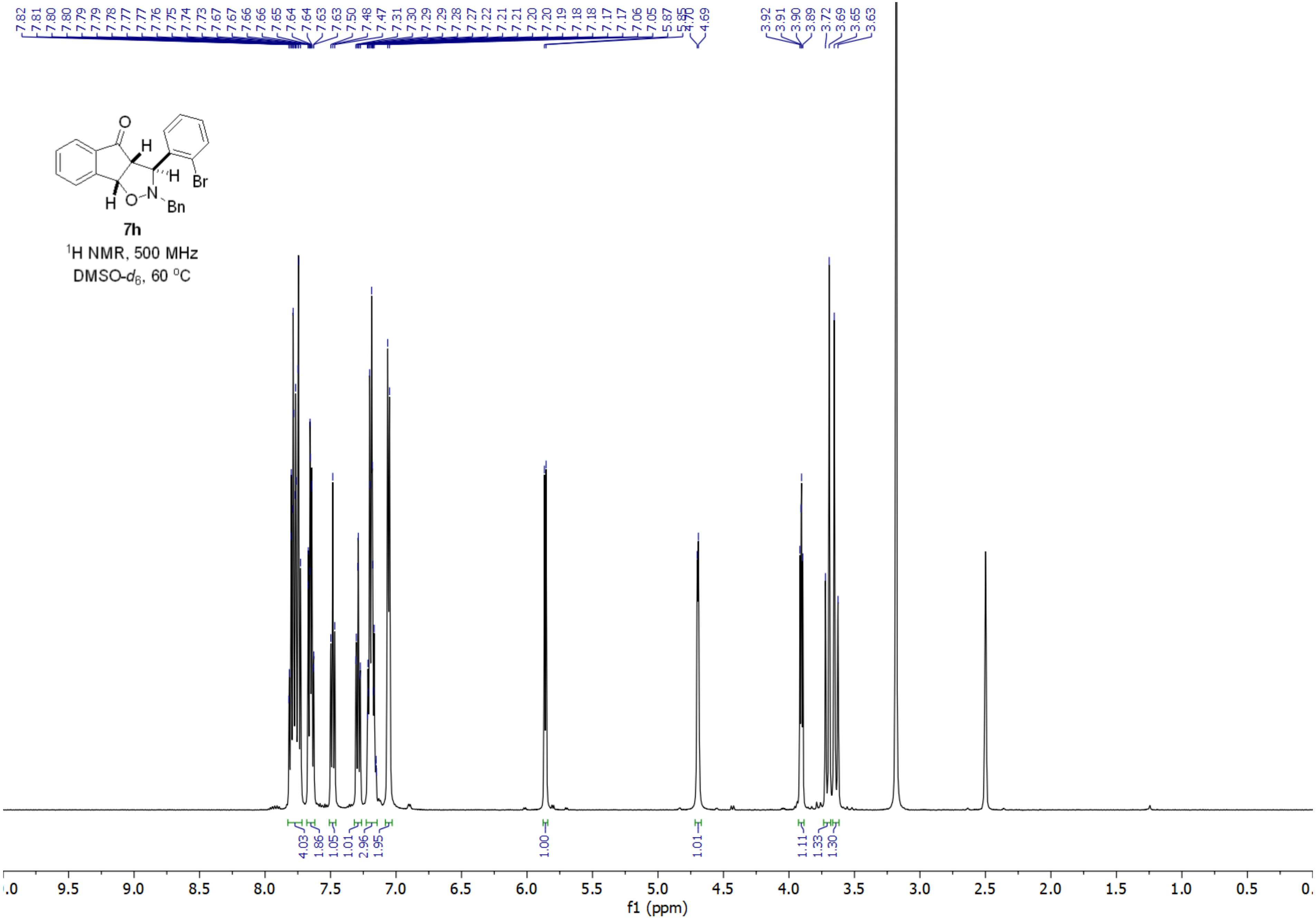
7h
1H NMR, 500 MHz
DMSO-d6, 60 °C
f1 (ppm)

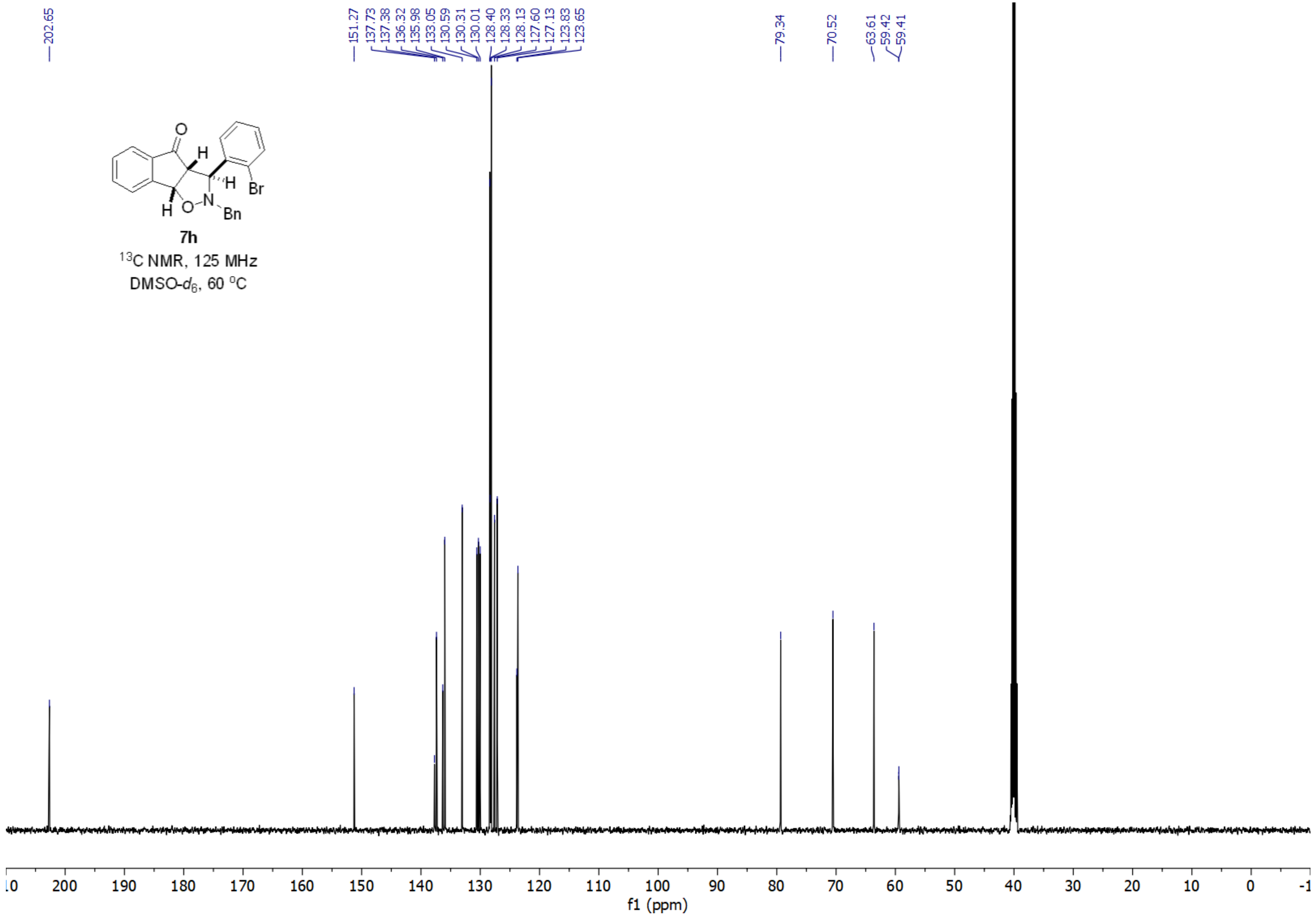

202.65
151.27
137.73
137.38
136.32
135.98
133.05
130.59
130.31
130.01
128.40
128.33
128.13
127.60
127.13
123.83
123.65
79.34
70.52
63.61
59.42
59.41
O
H
H
Br
H
O
N
Bn
7h
^{13}C NMR, 125 MHz
DMSO-d_6, 60 °C
f1 (ppm)

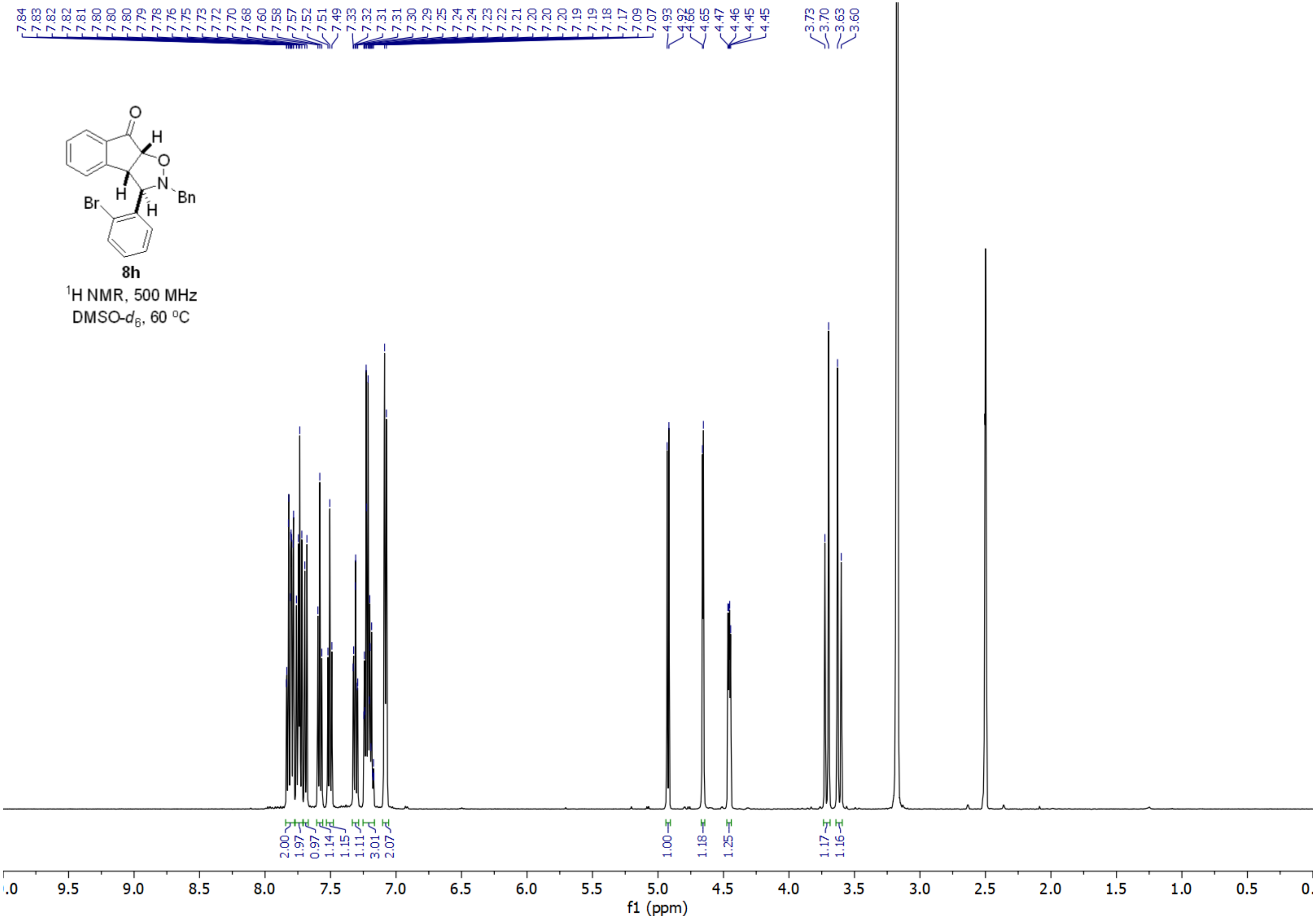
8h
^{1}H NMR, 500 MHz
DMSO-d_6, 60 °C
f1 (ppm)

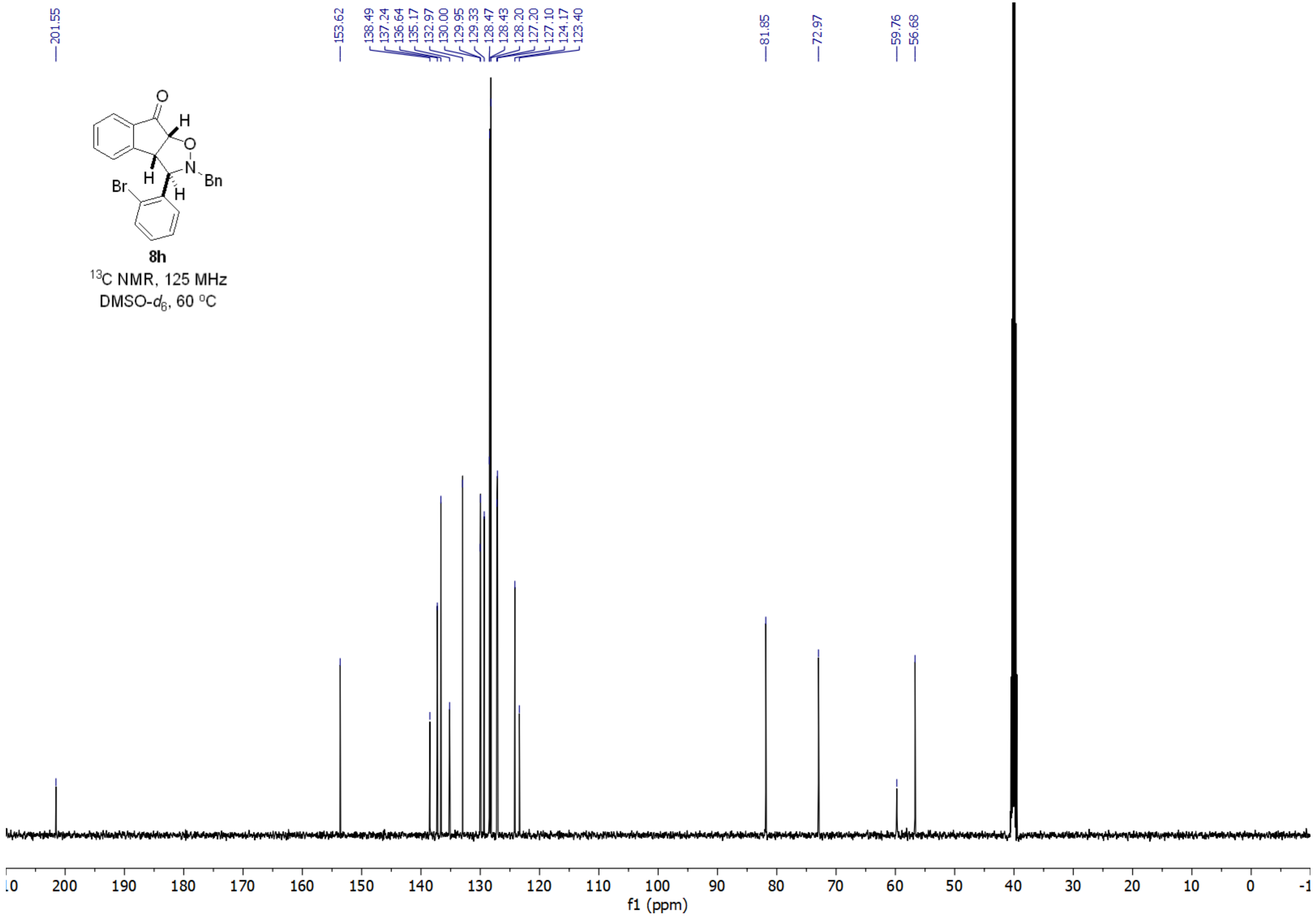

201.55
153.62
138.49
137.24
136.64
135.17
132.97
130.00
129.95
129.33
128.47
128.43
128.20
127.20
127.10
124.17
123.40
81.85
72.97
59.76
56.68
O
H
O
N
Bn
H
H
Br
8h
^{13}C NMR, 125 MHz
DMSO-d_6, 60 °C
f1 (ppm)

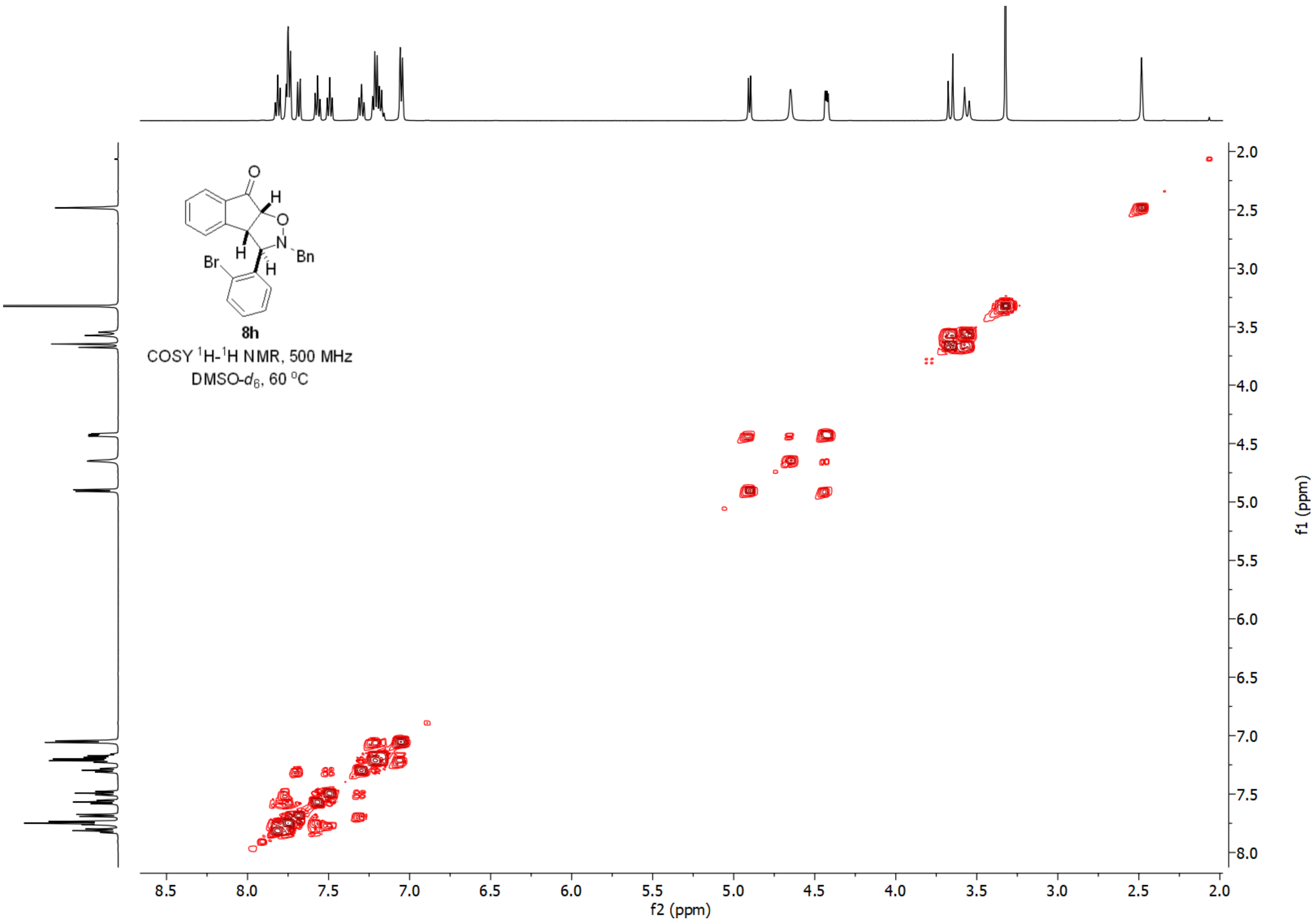
O
H
O
N
Bn
H
Br
H
8h
COSY ¹H-¹H NMR, 500 MHz
DMSO-d6, 60 °C
f2 (ppm)
f1 (ppm)

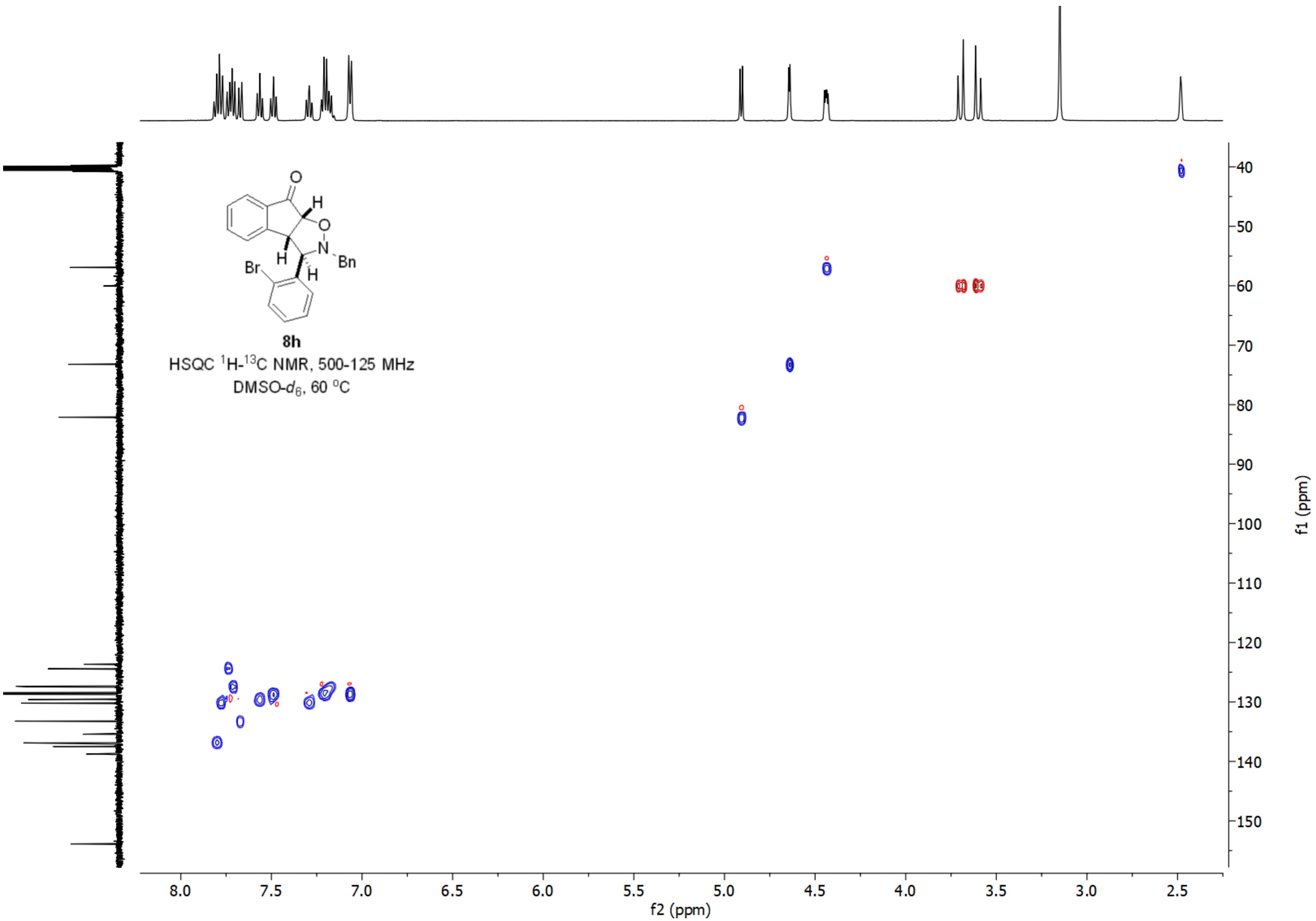

O
H
O
N
Bn
Br
H
H
8h
HSQC ¹H-¹³C NMR, 500-125 MHz
DMSO-d6, 60 °C
f1 (ppm)
f2 (ppm)

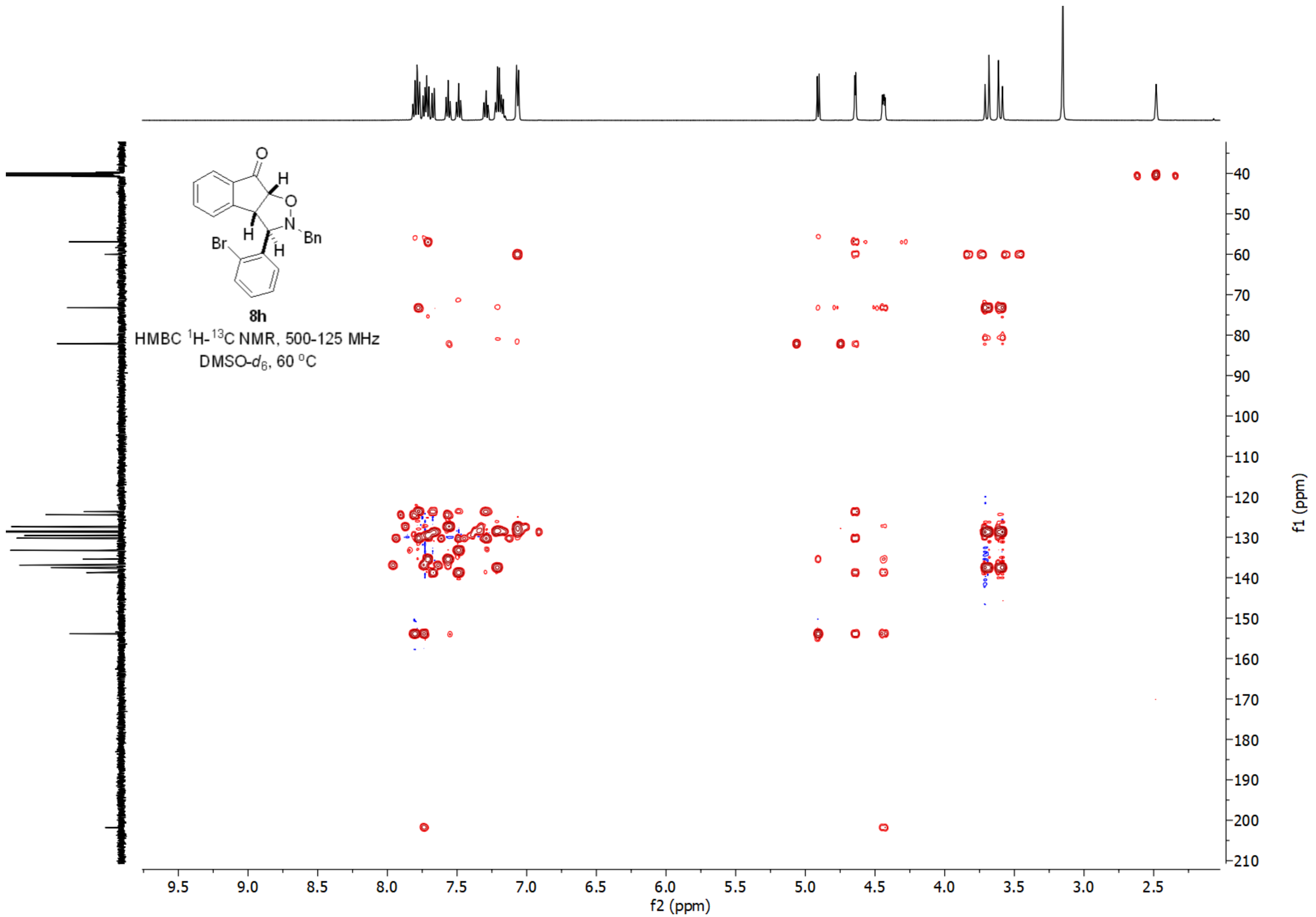

8h

HMBC ^{1}H-^{13}C NMR, 500-125 MHz

DMSO-d_6, 60 °C

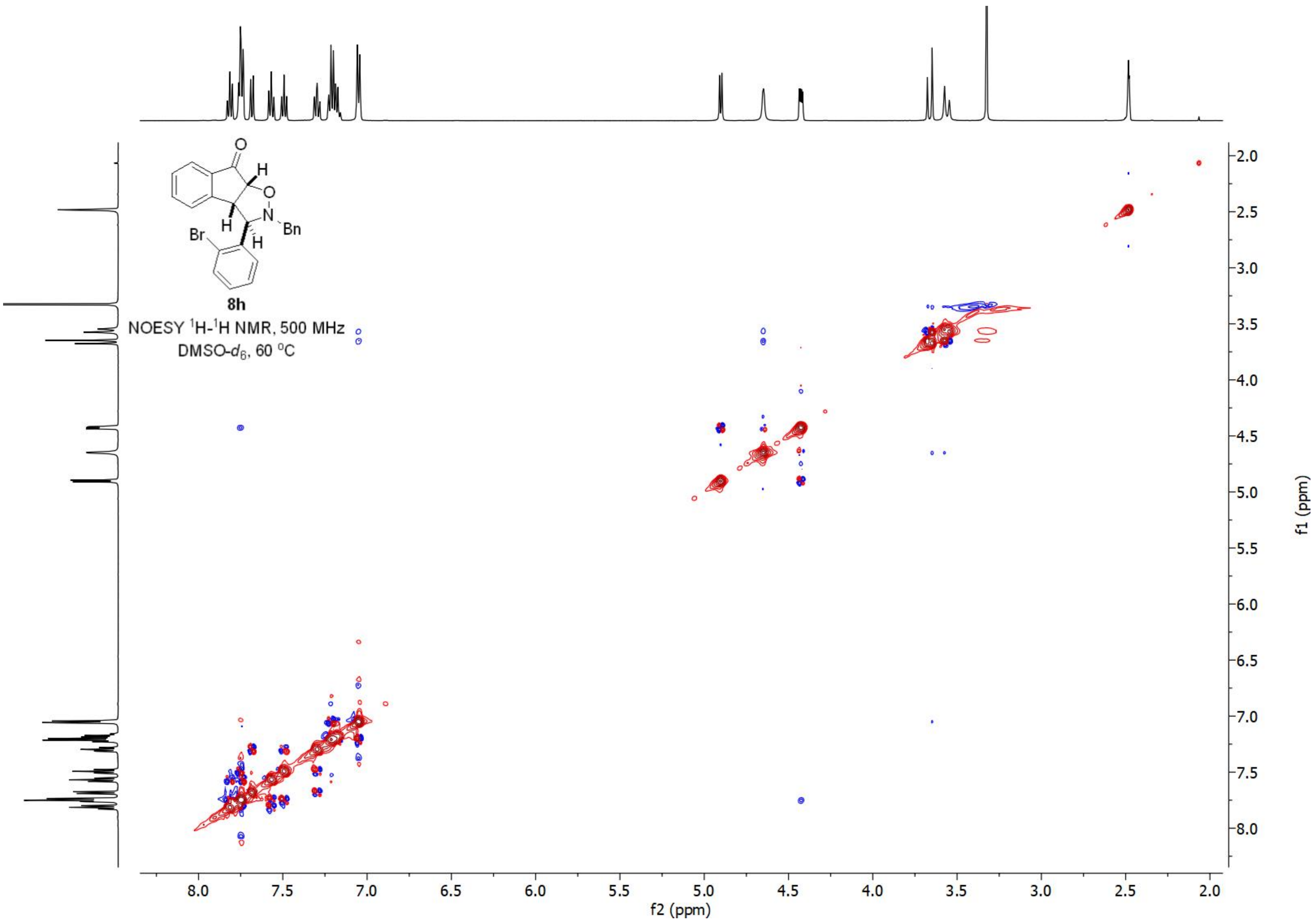
O
H
O
N
Bn
H
H
Br
8h
NOESY 1H-1H NMR, 500 MHz
DMSO-d6, 60 °C
f2 (ppm)
f1 (ppm)
8.0
7.5
7.0
6.5
6.0
5.5
5.0
4.5
4.0
3.5
3.0
2.5
2.0

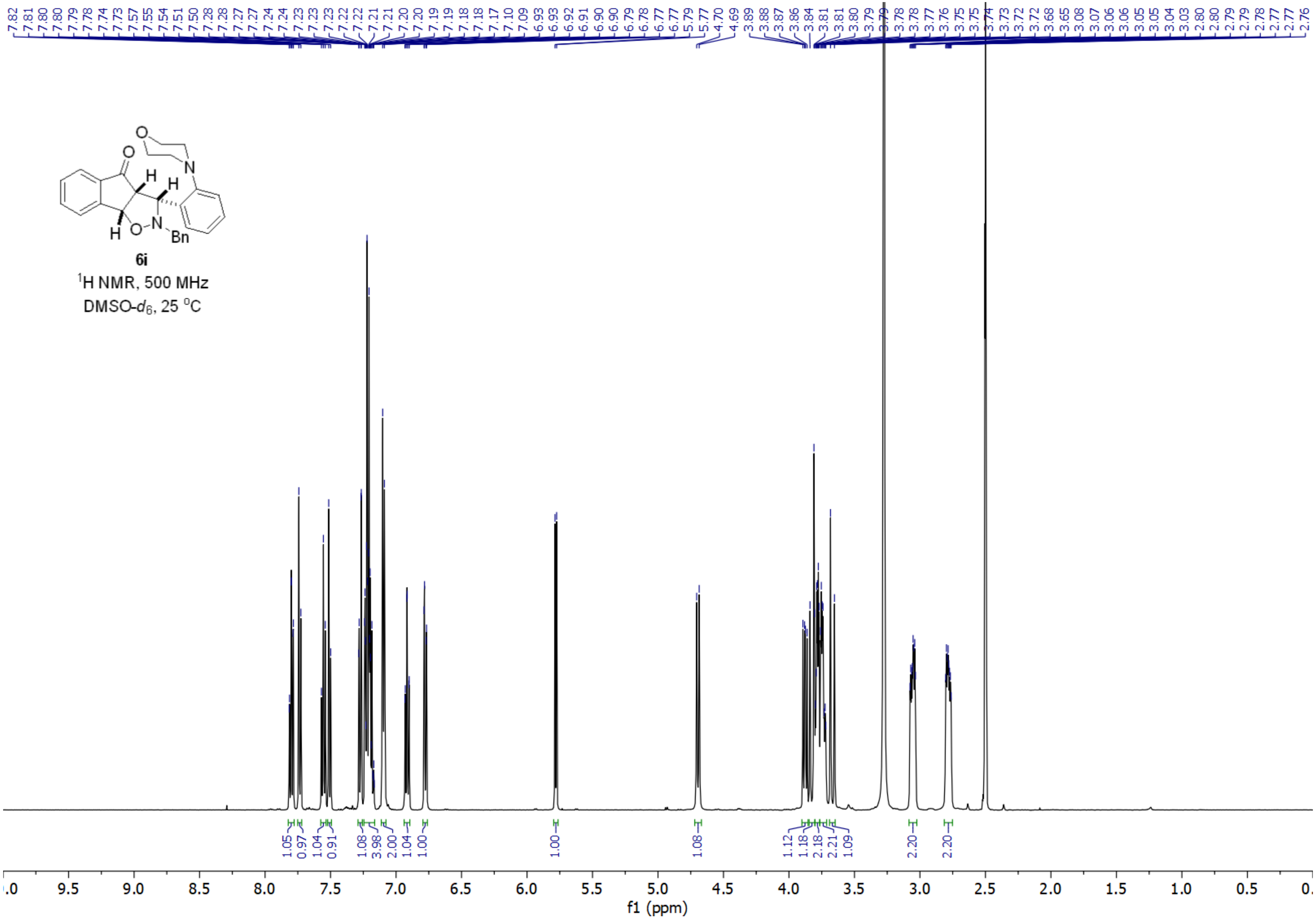
6i
¹H NMR, 500 MHz
DMSO-d6, 25 °C
f1 (ppm)

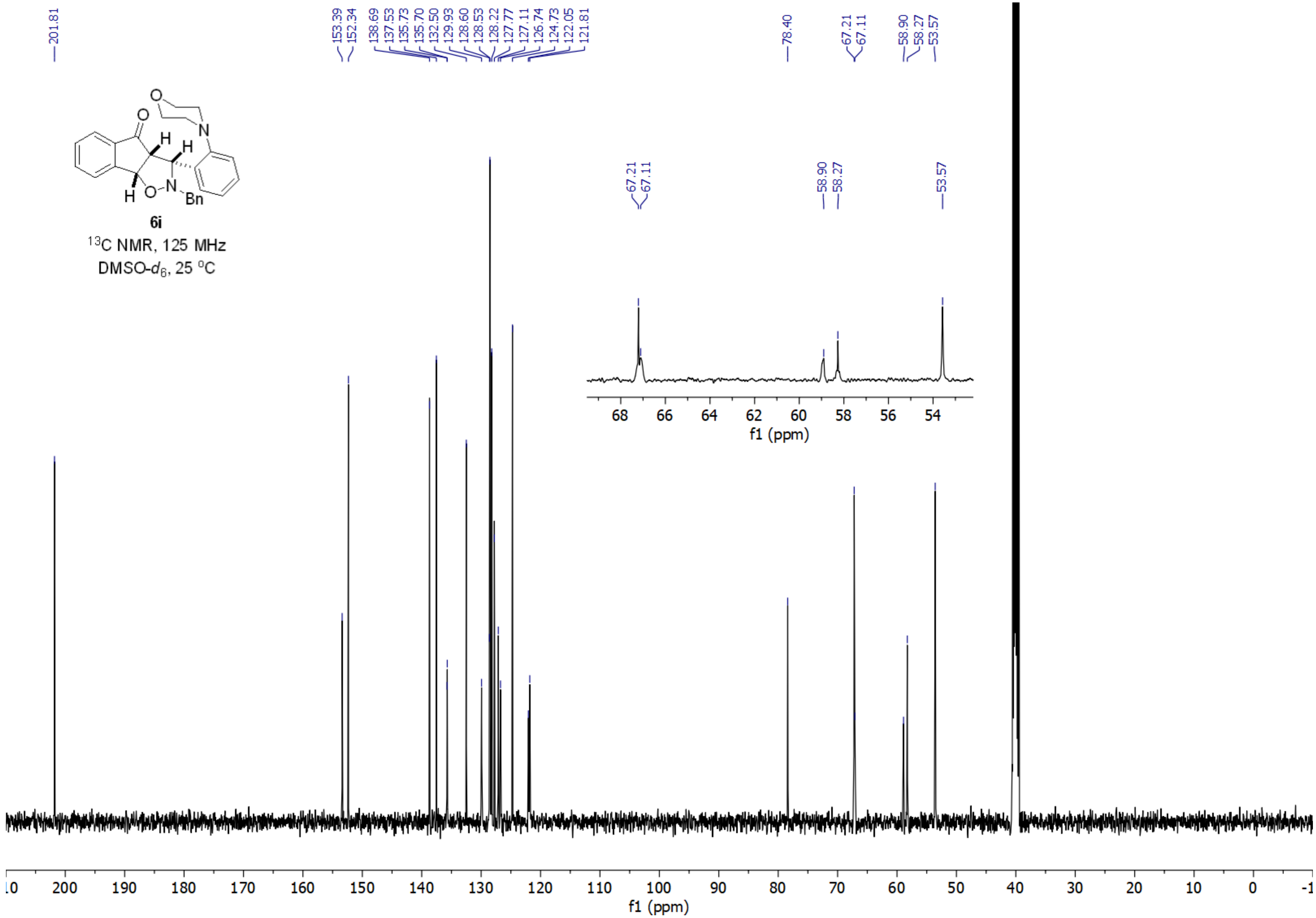

201.81
153.39
152.34
138.69
137.53
135.73
135.70
132.50
129.93
128.60
128.53
128.22
127.77
127.11
126.74
124.73
122.05
121.81
78.40
67.21
67.11
58.90
58.27
53.57
6i
^{13}C NMR, 125 MHz
DMSO-d_6, 25 °C
f1 (ppm)

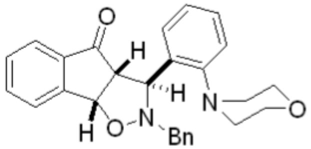

7i

^{1}H NMR, 500 MHz

DMSO-d_6, 60 °C

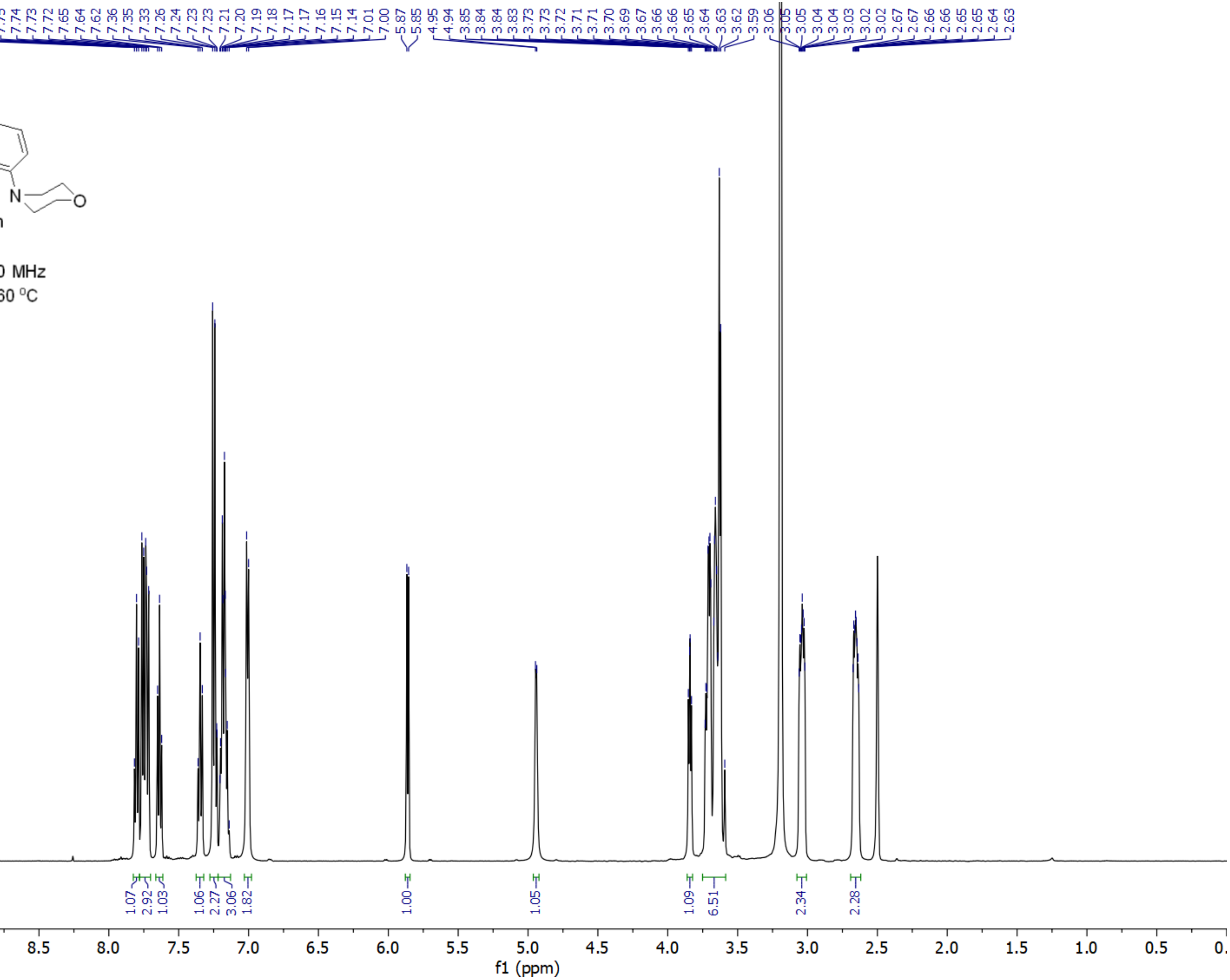

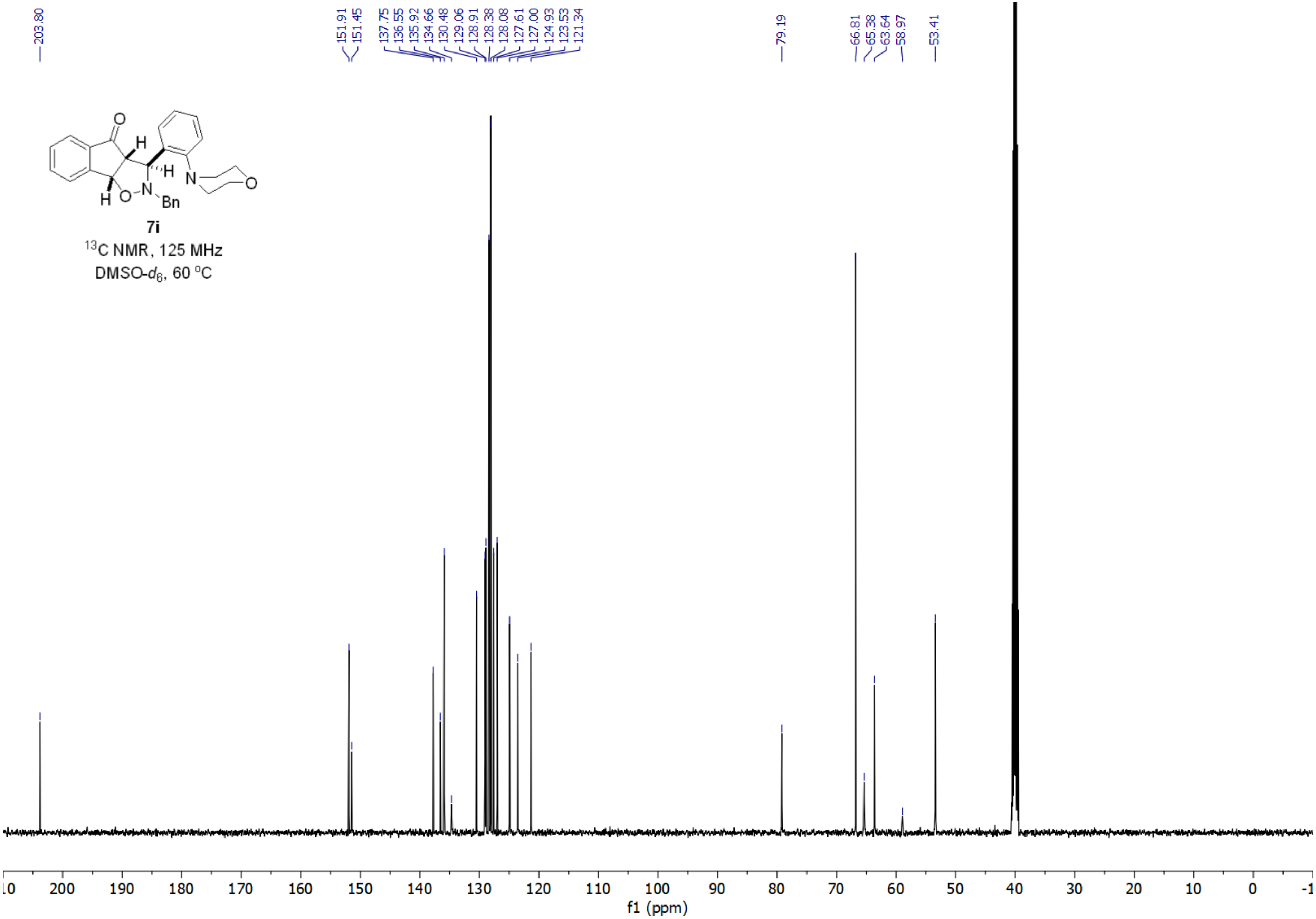
203.80
151.91
151.45
137.75
136.55
135.92
134.66
130.48
129.06
128.91
128.38
128.08
127.61
127.00
124.93
123.53
121.34
79.19
66.81
65.38
63.64
58.97
53.41
7i
^{13}C NMR, 125 MHz
DMSO-d_6, 60 °C
f1 (ppm)

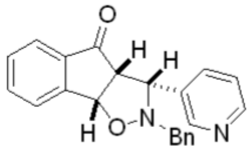

6j

^{1}H NMR, 500 MHz

DMSO-d_6, 25 °C

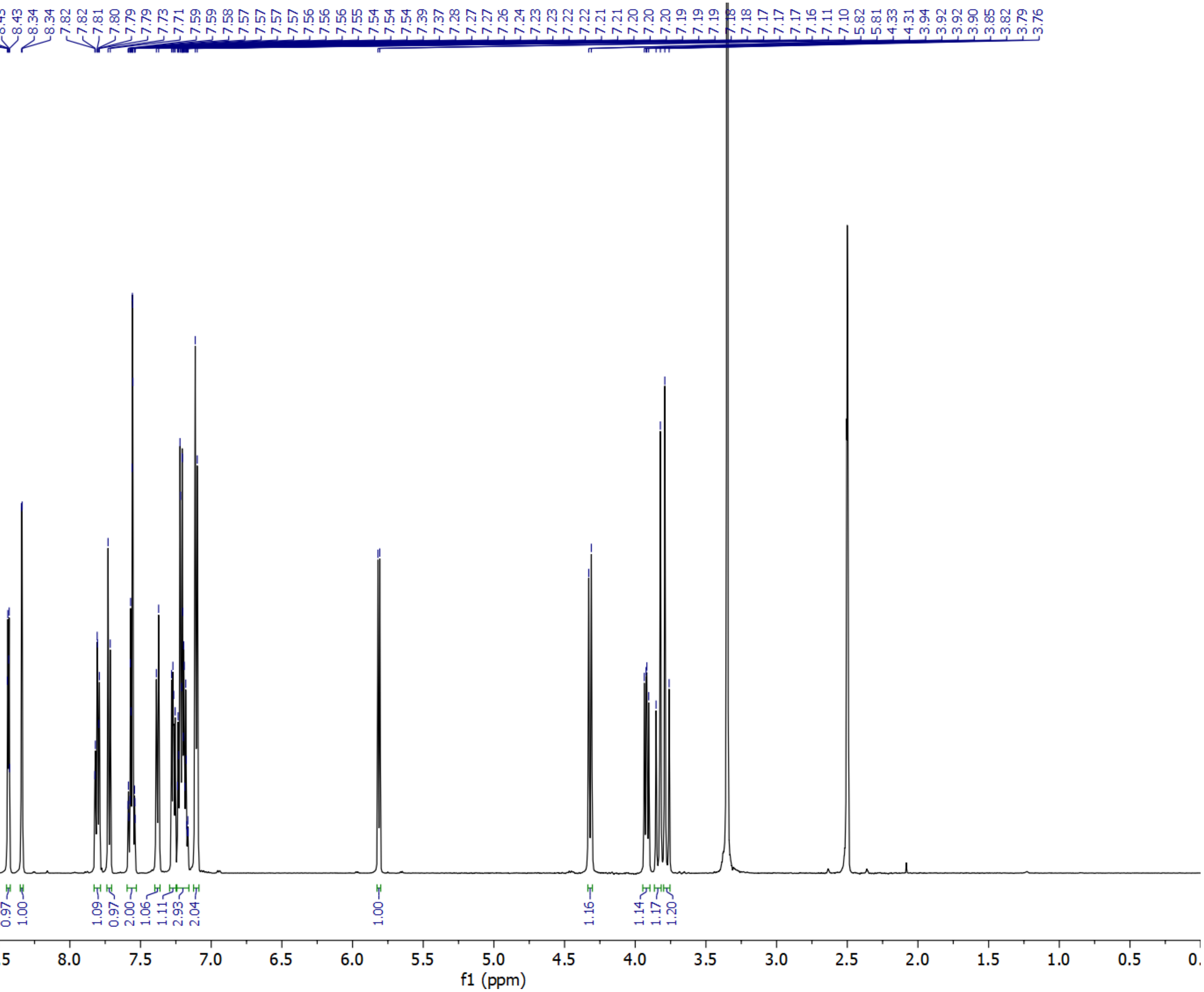

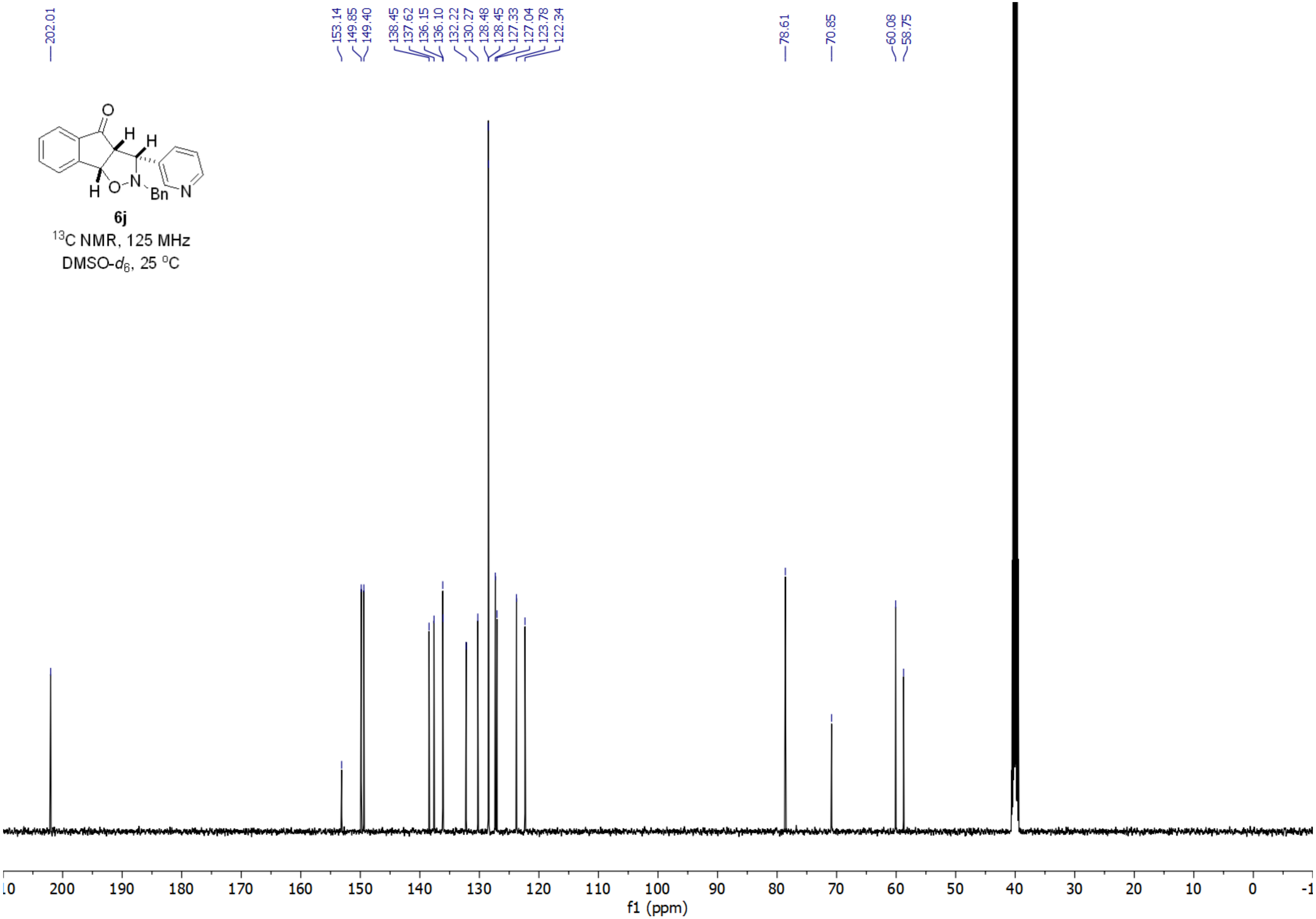

202.01
153.14
149.85
149.40
138.45
137.62
136.15
136.10
132.22
130.27
128.48
128.45
127.33
127.04
123.78
122.34
78.61
70.85
60.08
58.75
6j
^{13}C NMR, 125 MHz
DMSO-d_6, 25 ^{o}C
f1 (ppm)

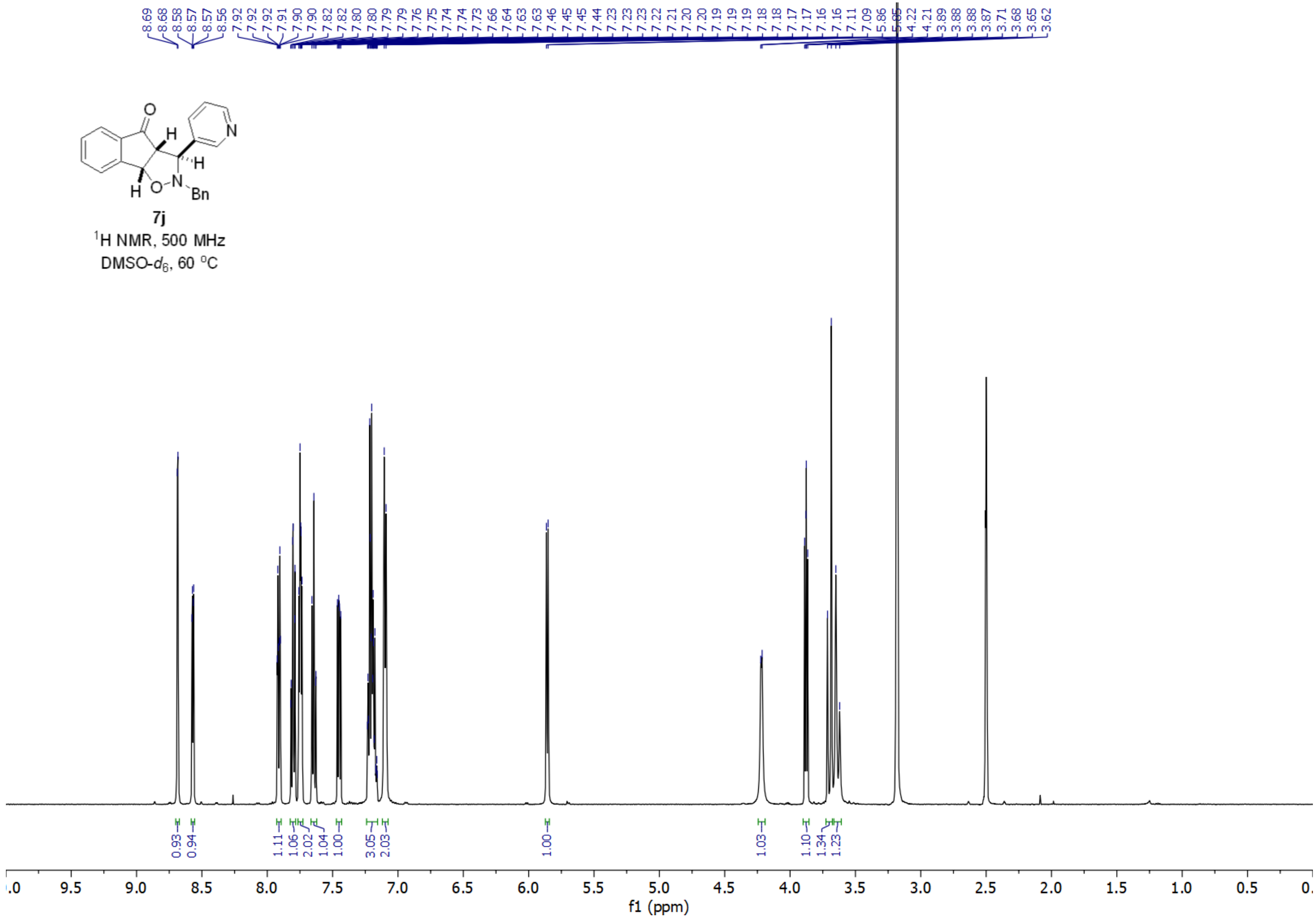

7j
^{1}H NMR, 500 MHz
DMSO-d_6, 60 °C
f1 (ppm)

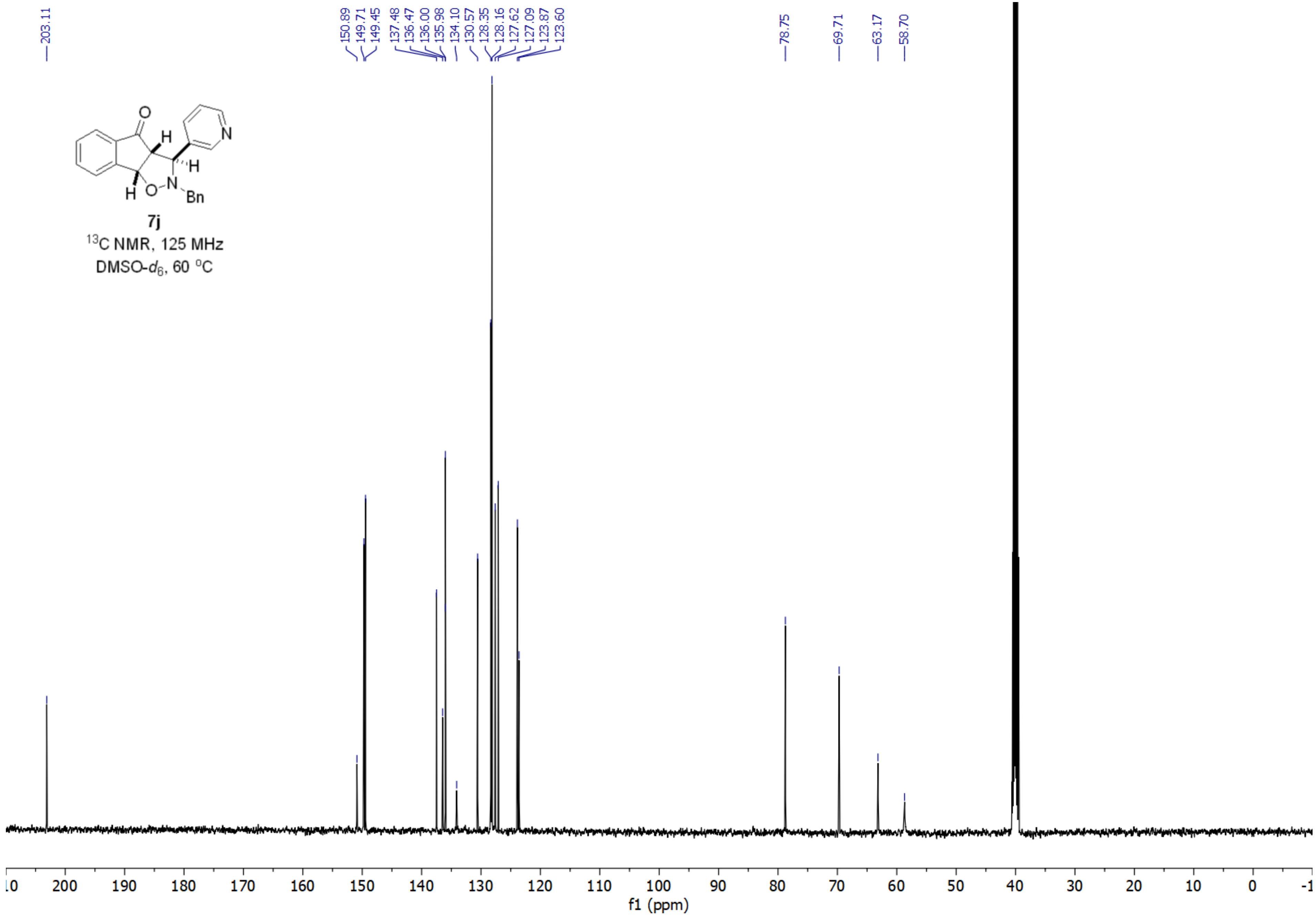
203.11
150.89
149.71
149.45
137.48
136.47
136.00
135.98
134.10
130.57
128.35
128.16
127.62
127.09
123.87
123.60
78.75
69.71
63.17
58.70
O
H
N
H
H
O
N
Bn
7j
^{13}C NMR, 125 MHz
DMSO-d_6, 60 °C
f1 (ppm)

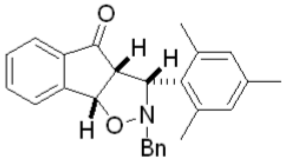

6k

^{1}H NMR, 500 MHz

DMSO-d_6, 25 °C

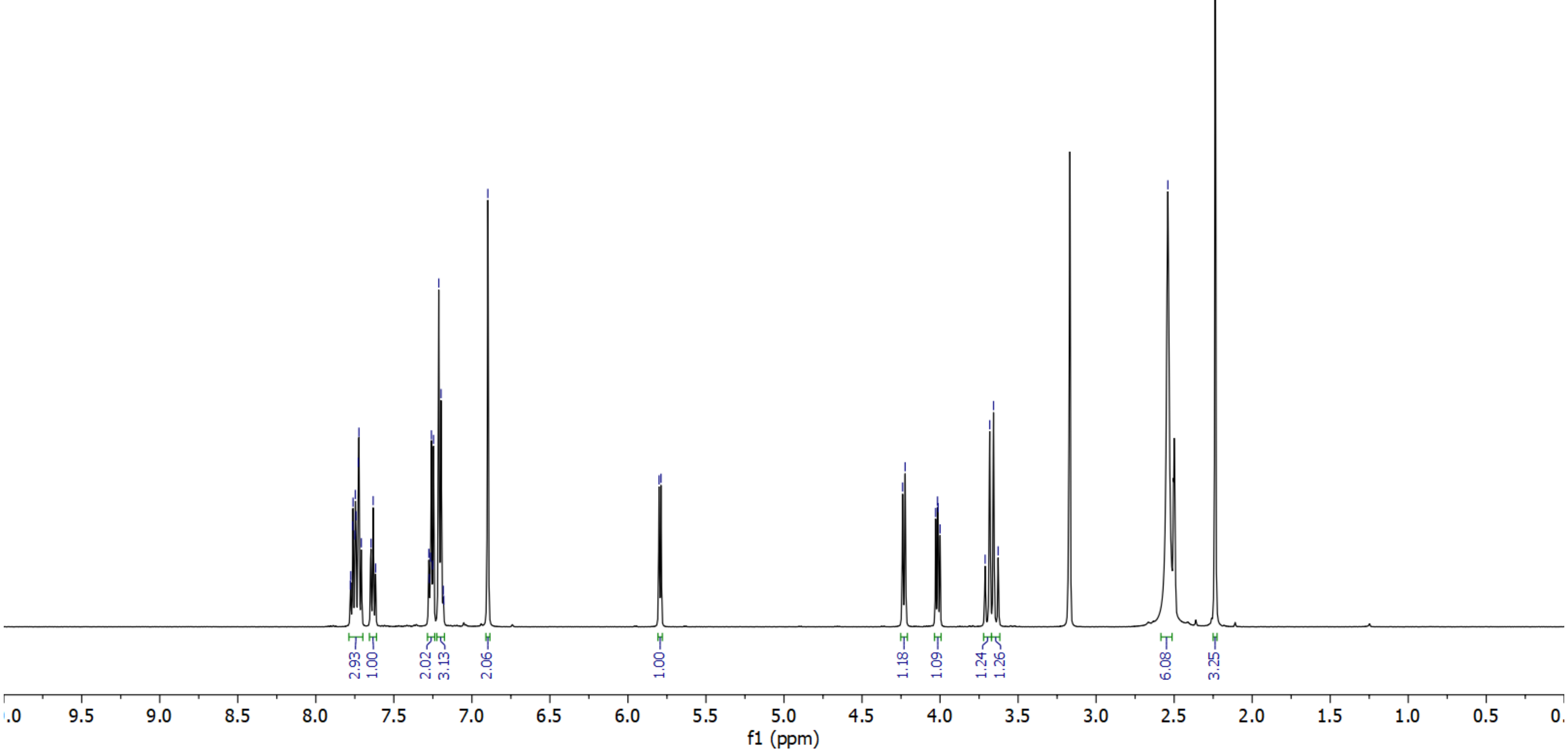

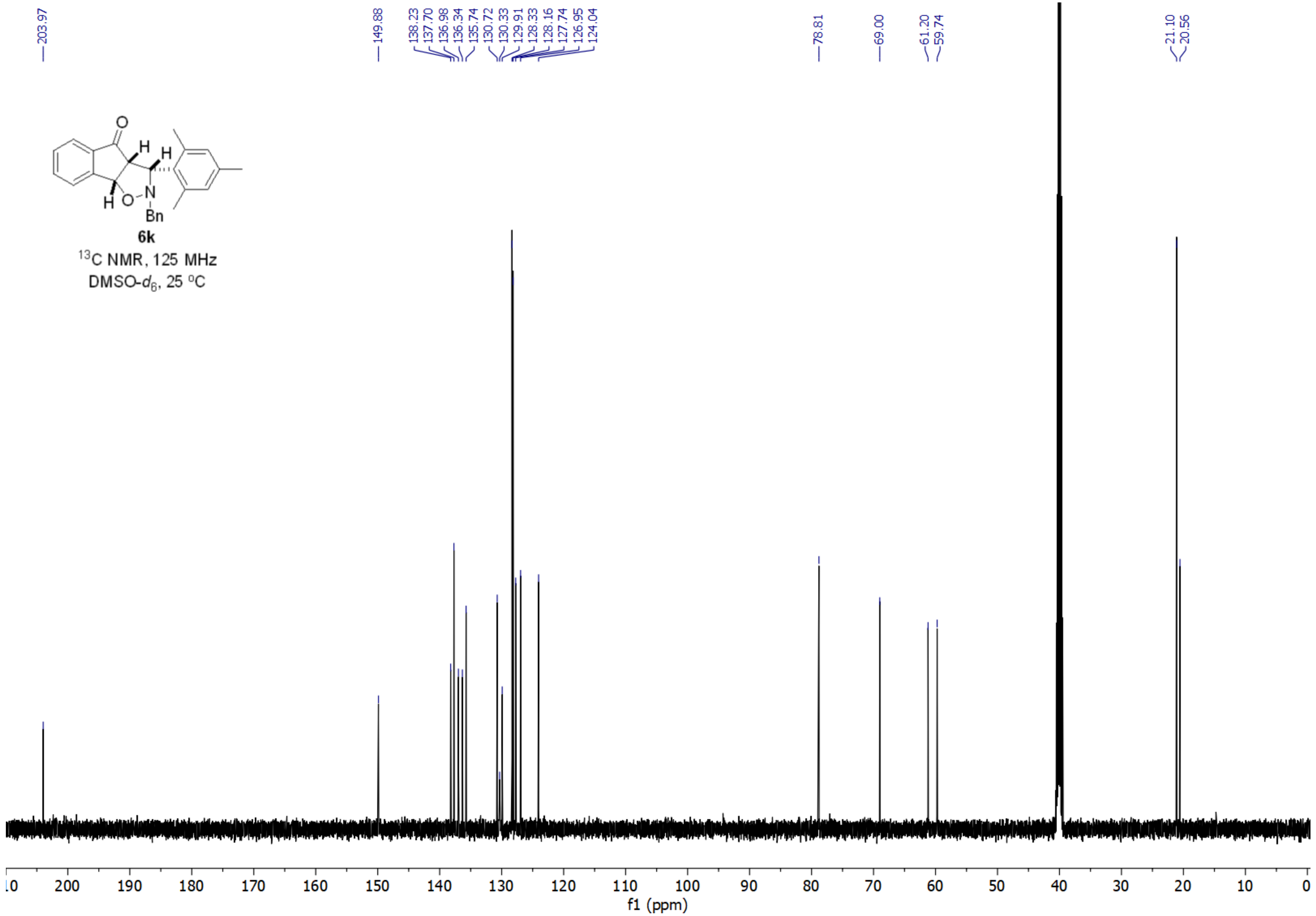

203.97
149.88
138.23
137.70
136.98
136.34
135.74
130.72
130.33
129.91
128.33
128.16
127.74
126.95
124.04
78.81
69.00
61.20
59.74
21.10
20.56
6k
^{13}C NMR, 125 MHz
DMSO-d_6, 25 °C
f1 (ppm)

7k

^{1}H NMR, 500 MHz

DMSO-d_6, 60 °C

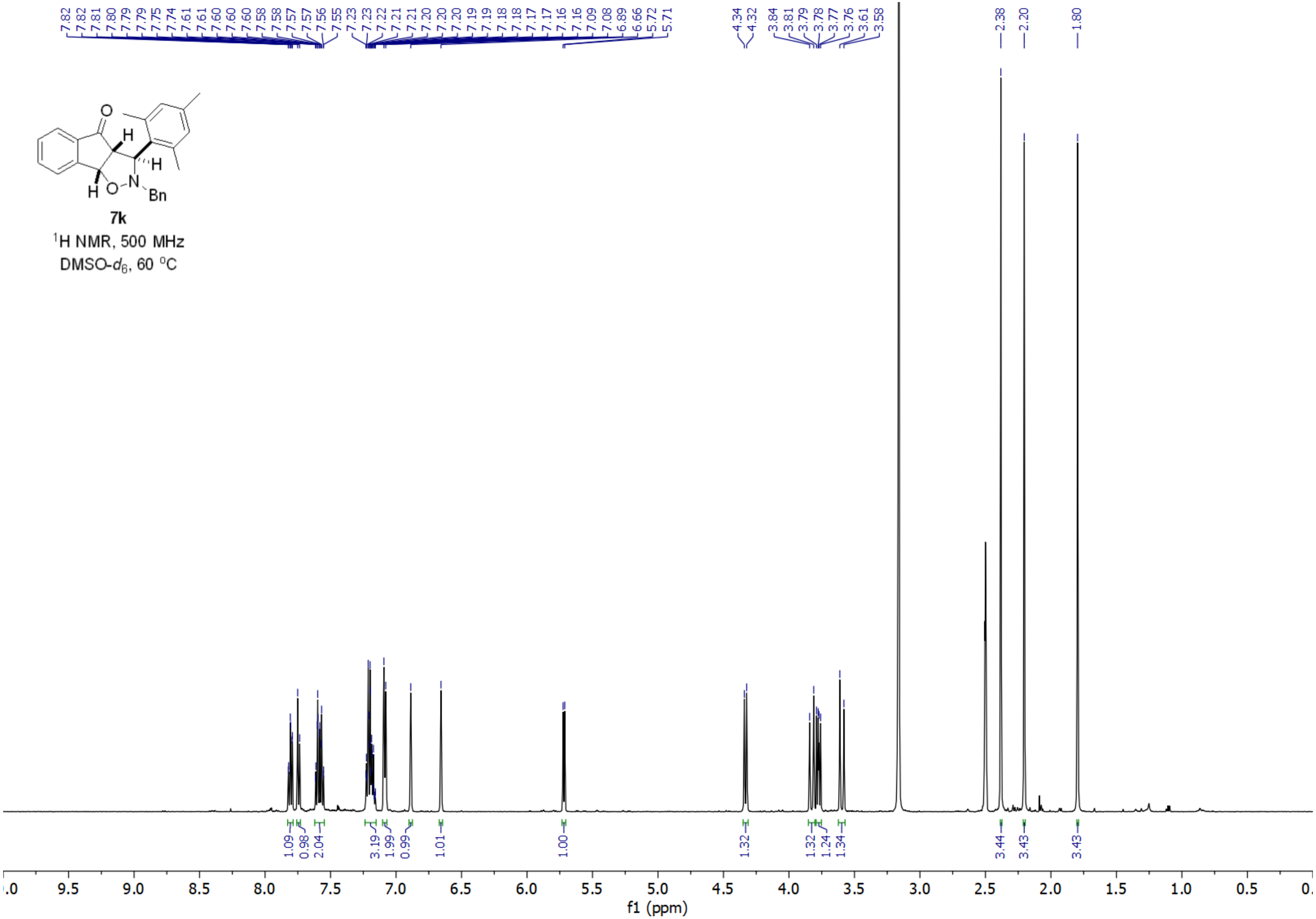

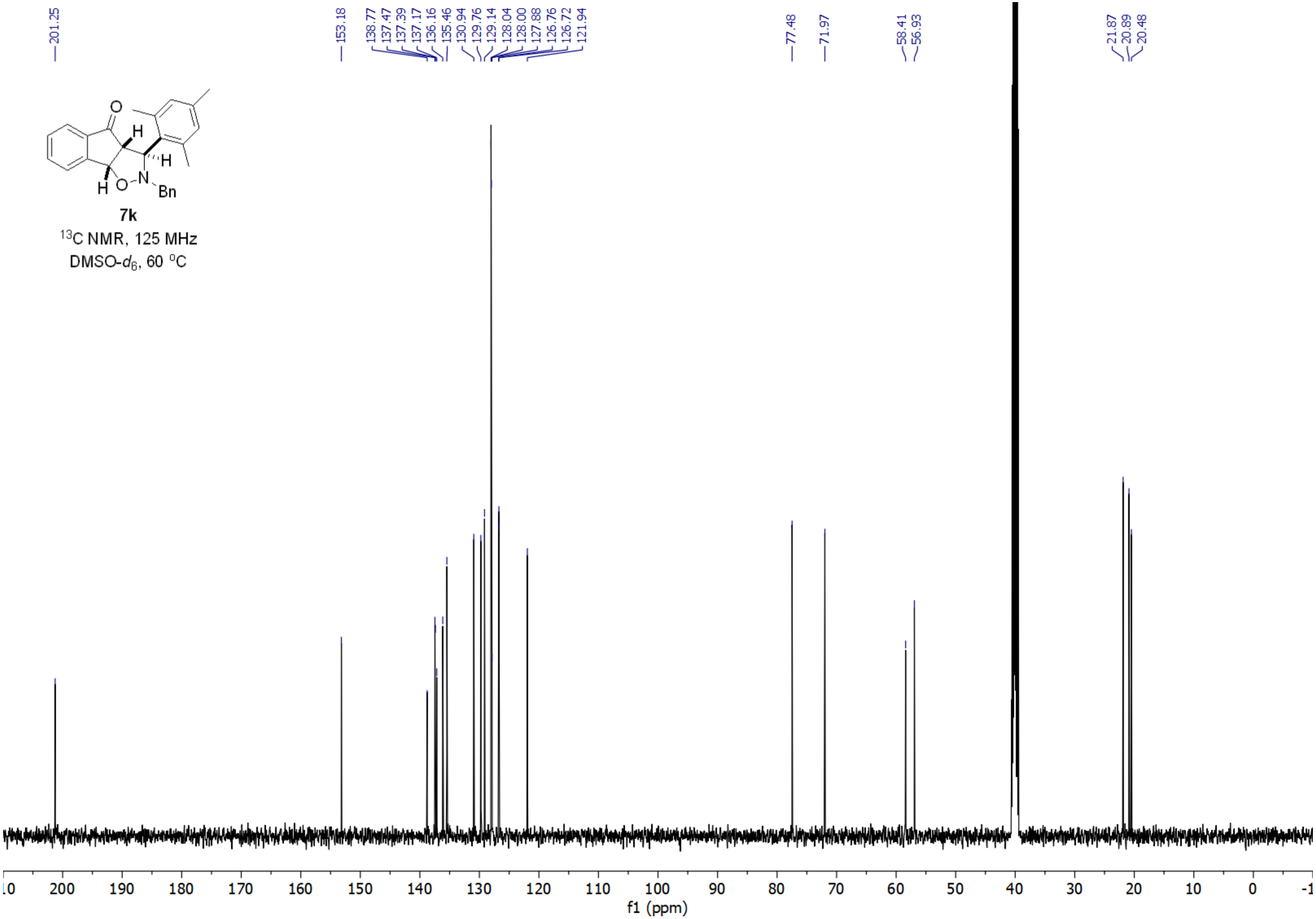

201.25
153.18
138.77
137.47
137.39
137.17
136.16
135.46
130.94
129.76
129.14
128.04
128.00
127.88
126.76
126.72
121.94
77.48
71.97
58.41
56.93
21.87
20.89
20.48
O
H
H
H
O
N
Bn
7k
^{13}C NMR, 125 MHz
DMSO-d_6, 60 °C
f1 (ppm)

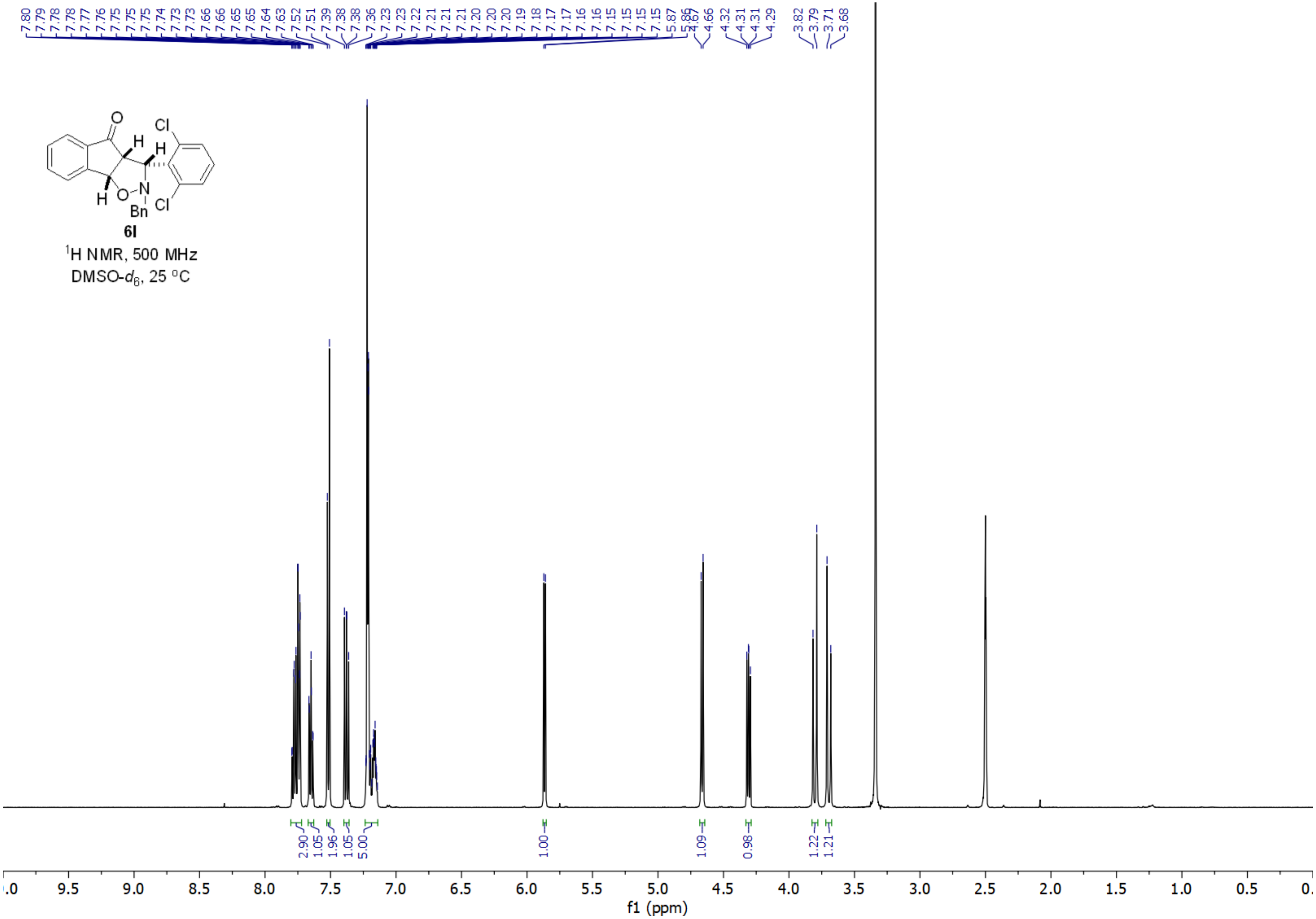

6l
1H NMR, 500 MHz
DMSO-d6, 25 °C
f1 (ppm)

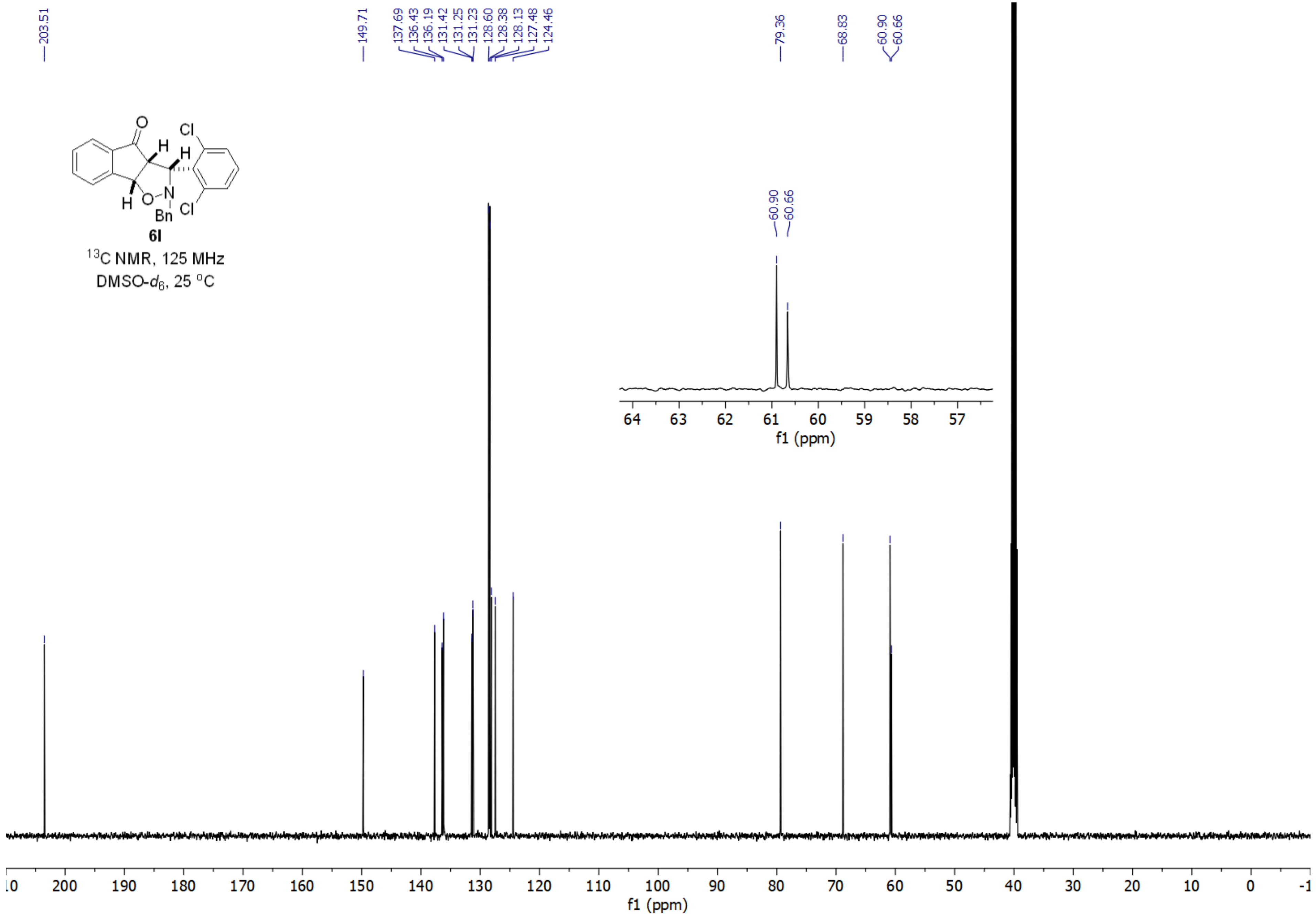
203.51
149.71
137.69
136.43
136.19
131.42
131.25
131.23
128.60
128.38
128.13
127.48
124.46
79.36
68.83
60.90
60.66
6l
^{13}C NMR, 125 MHz
DMSO-d_6, 25 °C
f1 (ppm)

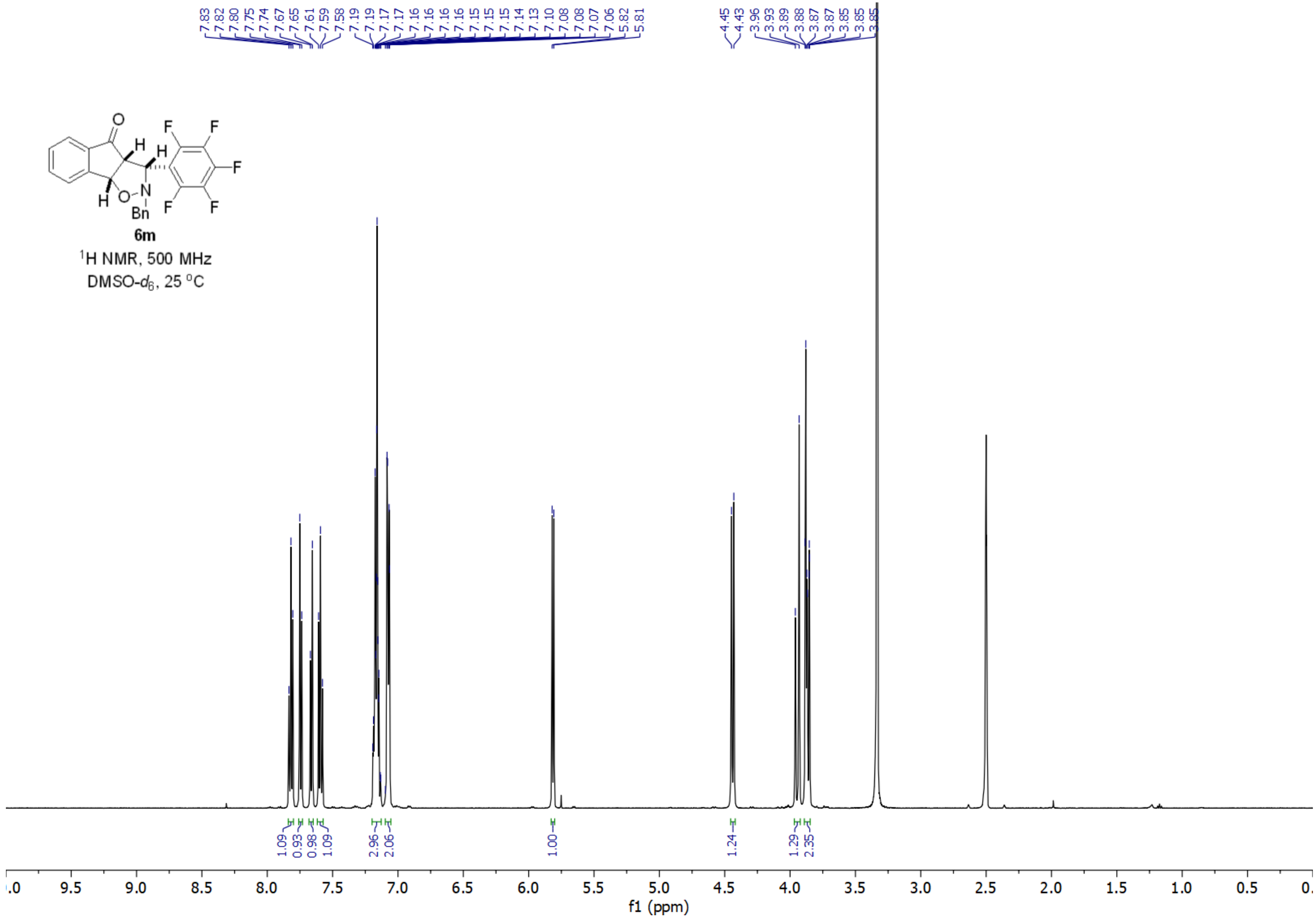
7.83 7.82 7.80 7.75 7.74 7.67 7.65 7.61 7.59 7.58 7.19 7.17 7.17 7.16 7.16 7.16 7.15 7.15 7.15 7.14 7.13 7.10 7.08 7.07 7.06 5.82 5.81
4.45 4.43 3.96 3.93 3.89 3.88 3.87 3.87 3.85 3.85
6m
1H NMR, 500 MHz
DMSO-d6, 25 °C
1.09 0.93 0.98 1.09 2.96 2.06 1.00 1.24 1.29 2.35
f1 (ppm)

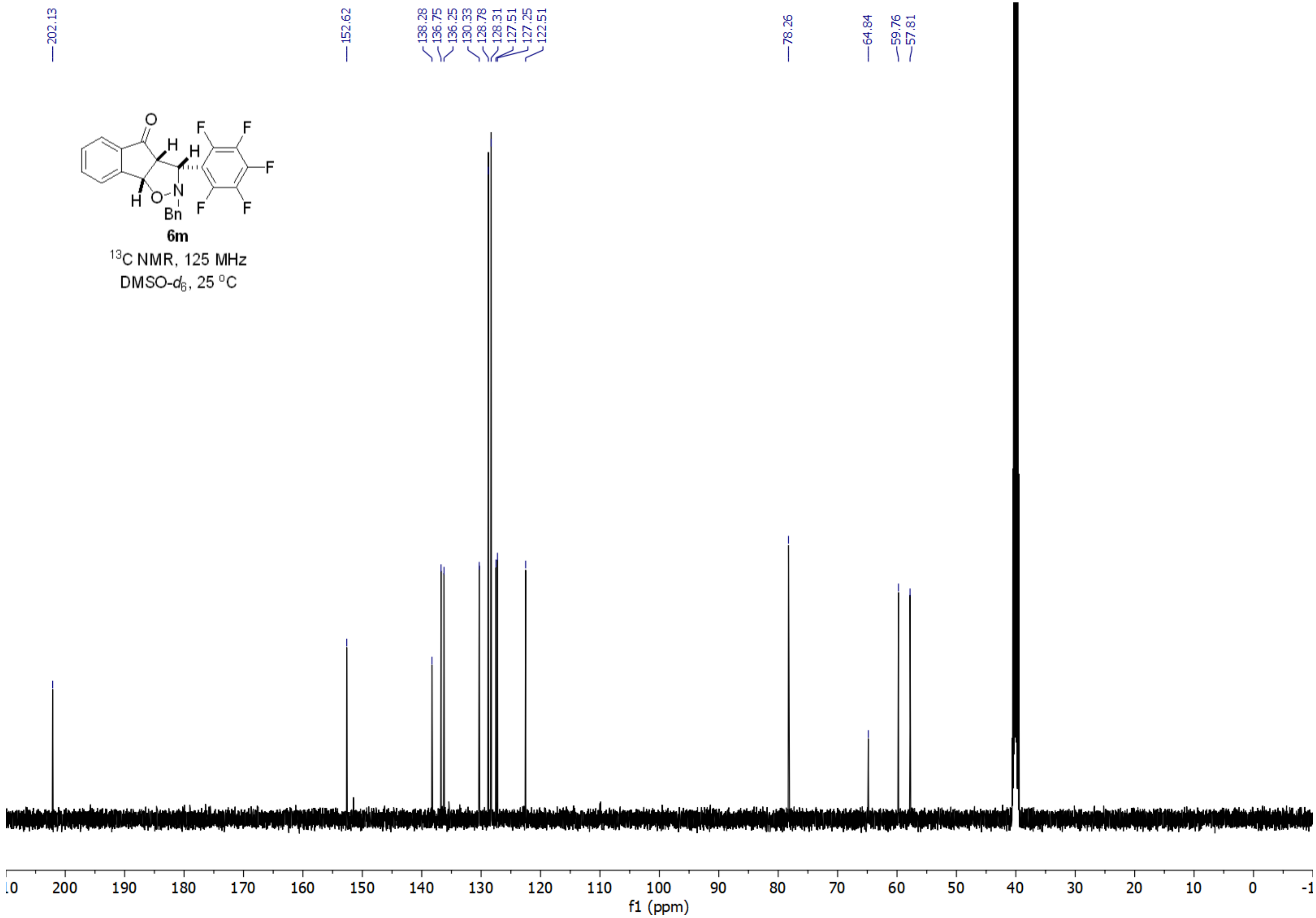
202.13
152.62
138.28
136.75
136.25
130.33
128.78
128.31
127.51
127.25
122.51
78.26
64.84
59.76
57.81
O
H
H
F
F
F
F
F
H
O
N
Bn
6m
13C NMR, 125 MHz
DMSO-d6, 25 °C
f1 (ppm)

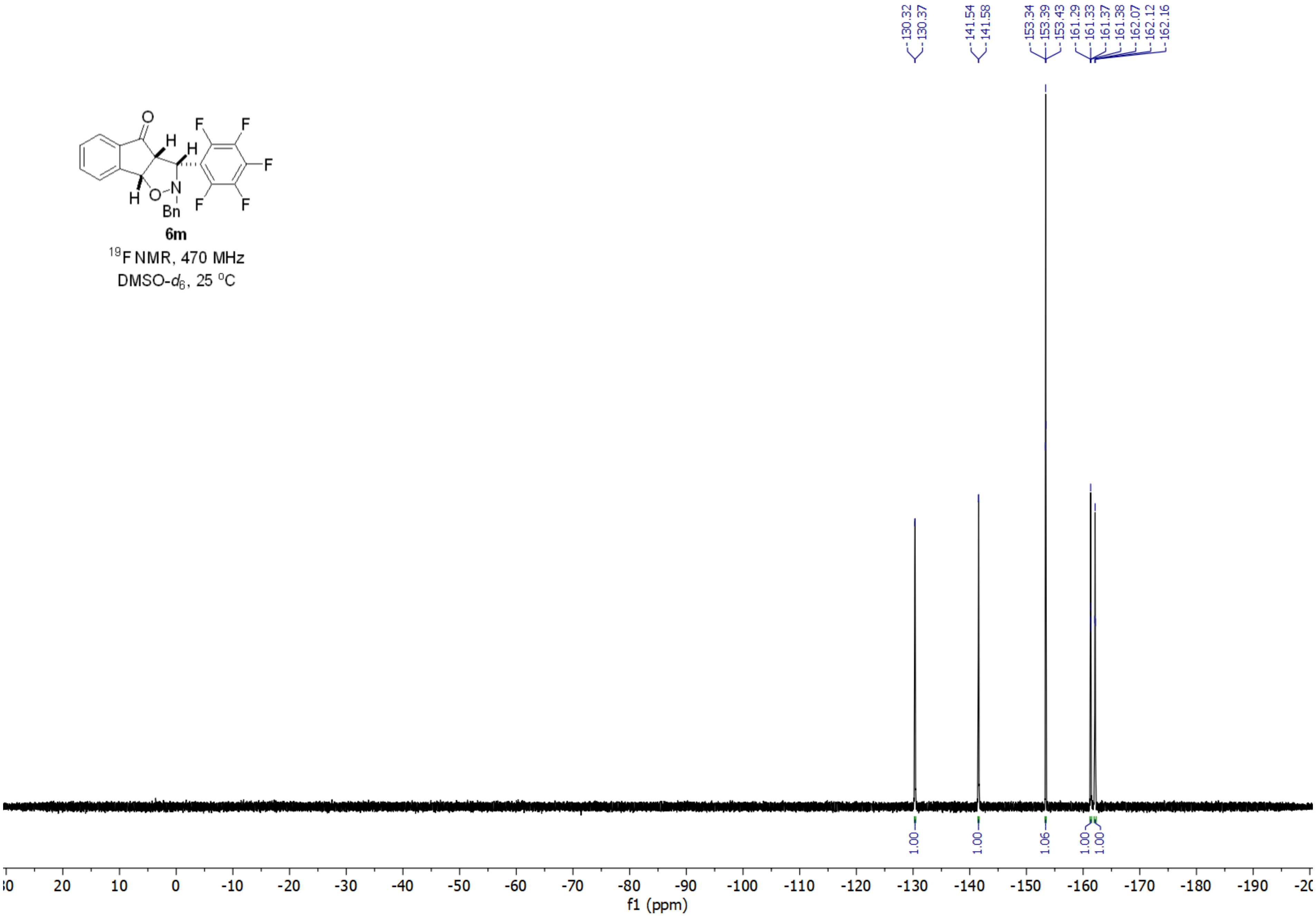

6m
19F NMR, 470 MHz
DMSO-d6, 25 °C
-130.32
-130.37
-141.54
-141.58
-153.34
-153.39
-153.43
-161.29
-161.33
-161.37
-161.38
-162.07
-162.12
-162.16
1.00
1.00
1.06
1.00
1.00
f1 (ppm)

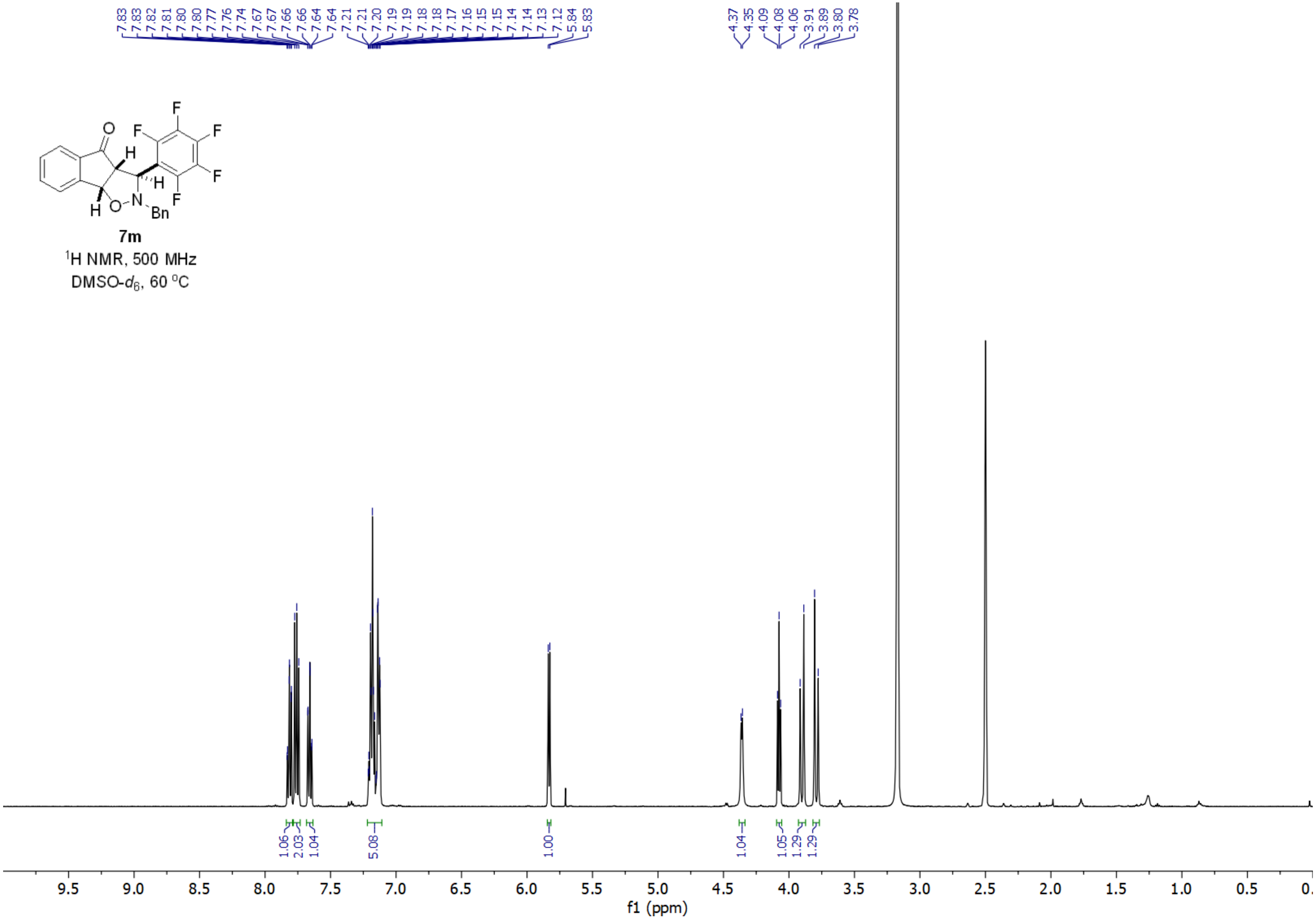

7.83
7.83
7.82
7.81
7.80
7.77
7.76
7.74
7.67
7.67
7.66
7.66
7.64
7.64
7.21
7.21
7.20
7.19
7.19
7.18
7.18
7.17
7.16
7.15
7.15
7.14
7.14
7.13
7.12
5.84
5.83
4.37
4.35
4.09
4.08
4.06
3.91
3.89
3.80
3.78
7m
¹H NMR, 500 MHz
DMSO-d6, 60 °C
1.06
2.03
1.04
5.08
1.00
1.04
1.05
1.29
1.29
9.5
9.0
8.5
8.0
7.5
7.0
6.5
6.0
5.5
5.0
4.5
4.0
3.5
3.0
2.5
2.0
1.5
1.0
0.5
0.
f1 (ppm)

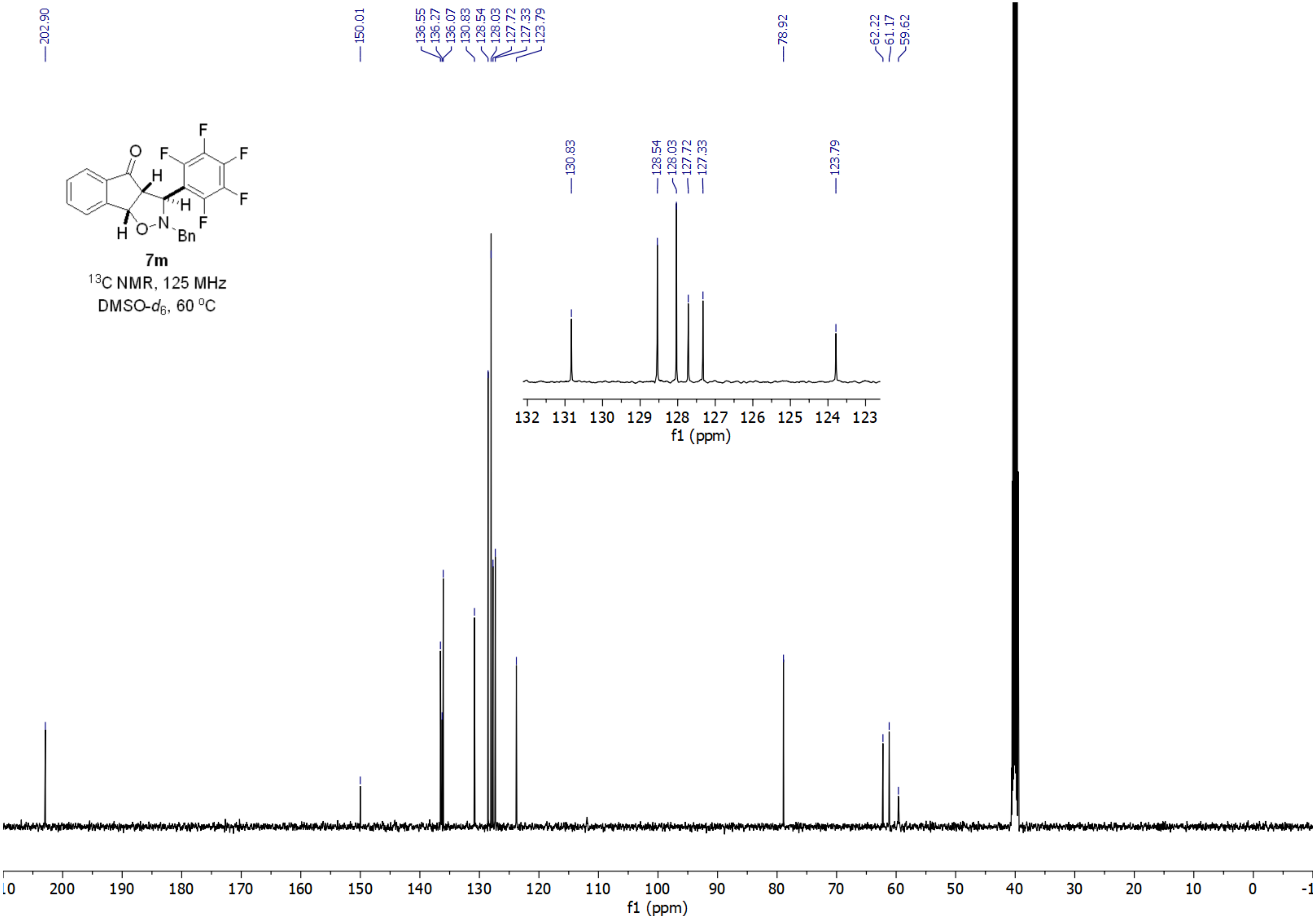
202.90
150.01
136.55
136.27
136.07
130.83
128.54
128.03
127.72
127.33
123.79
78.92
62.22
61.17
59.62
O
H
F
F
F
F
F
H
H
O
N
Bn
7m
^{13}C NMR, 125 MHz
DMSO-d_6, 60 °C
f1 (ppm)

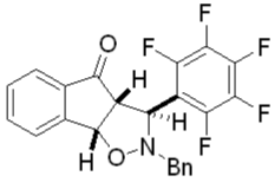

7m
^{19}F NMR, 470 MHz
DMSO-d_6, 25 °C

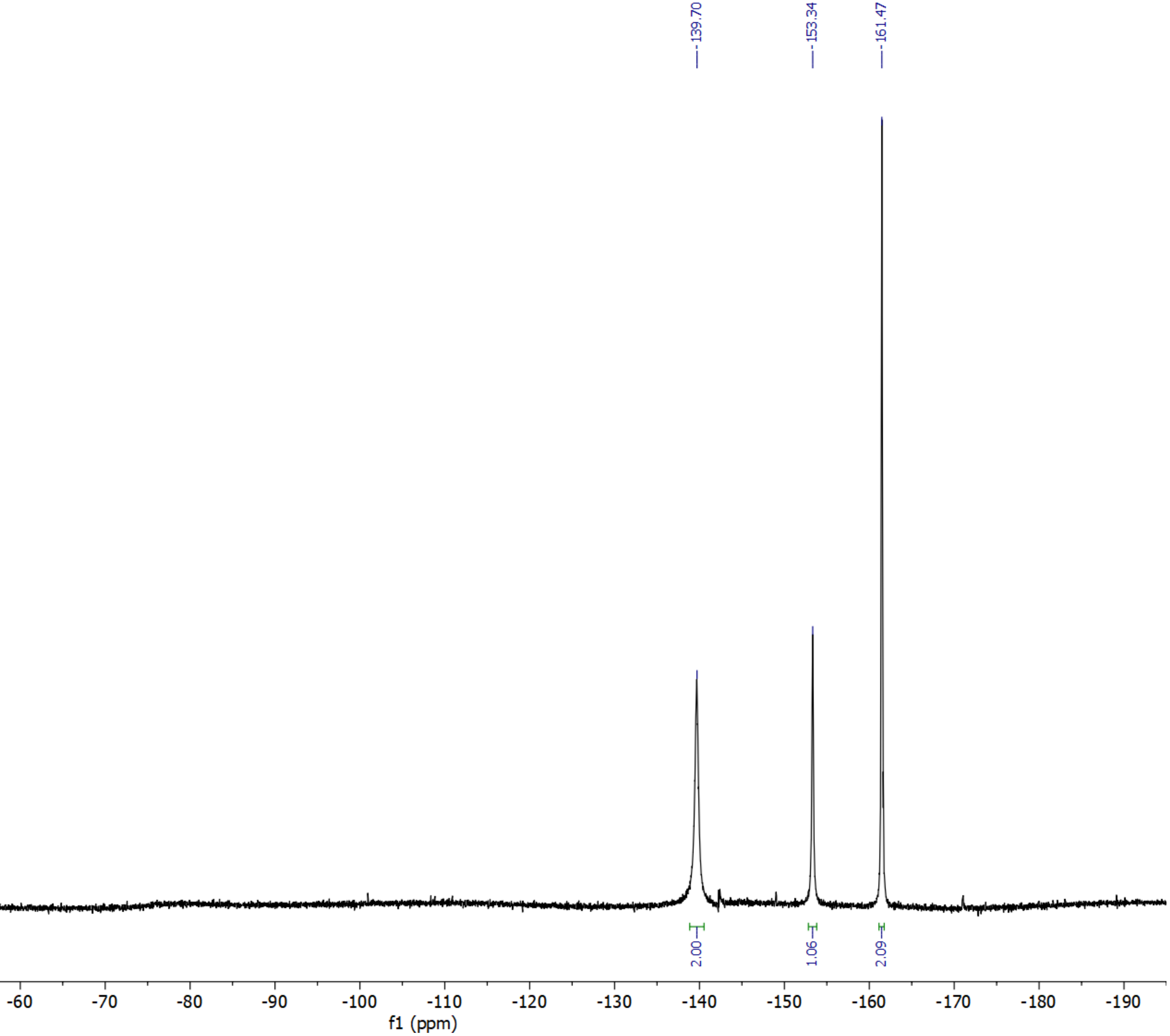

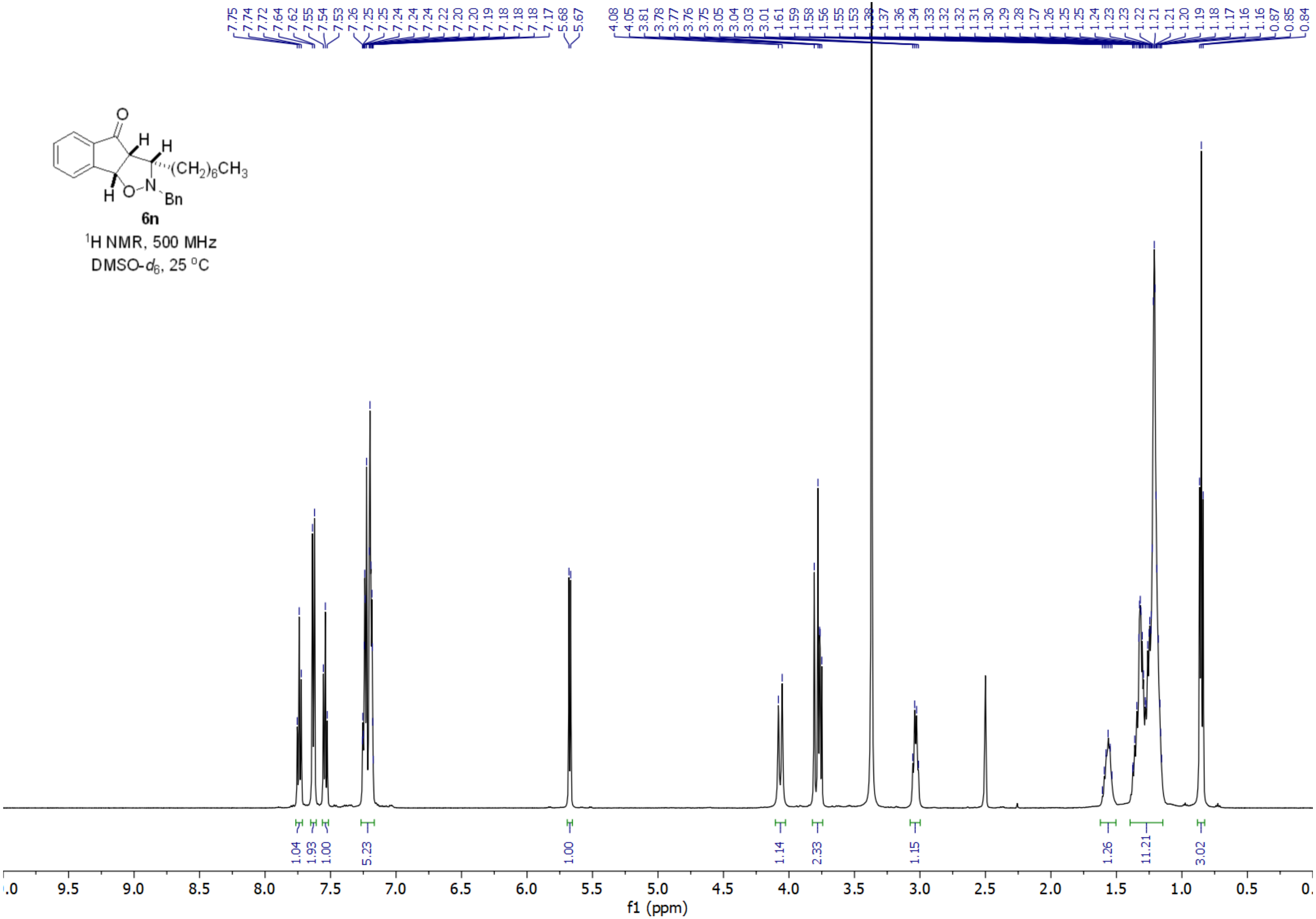

6n

^{1}H NMR, 500 MHz

DMSO-d_6, 25 °C

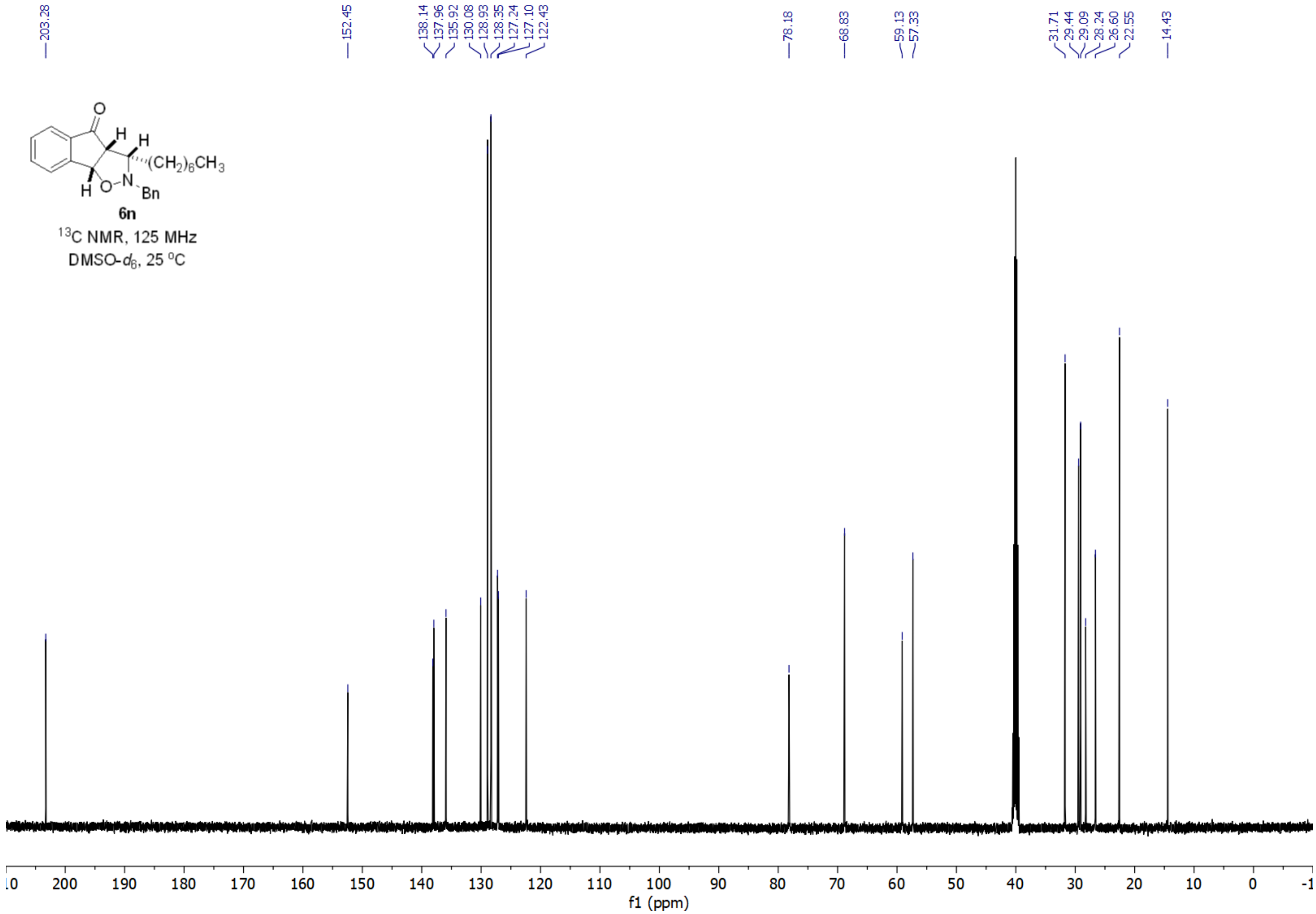

203.28
152.45
138.14
137.96
135.92
130.08
128.93
128.35
127.24
127.10
122.43
78.18
68.83
59.13
57.33
31.71
29.44
29.09
28.24
26.60
22.55
14.43
6n
^{13}C NMR, 125 MHz
DMSO-d_6, 25 °C
f1 (ppm)

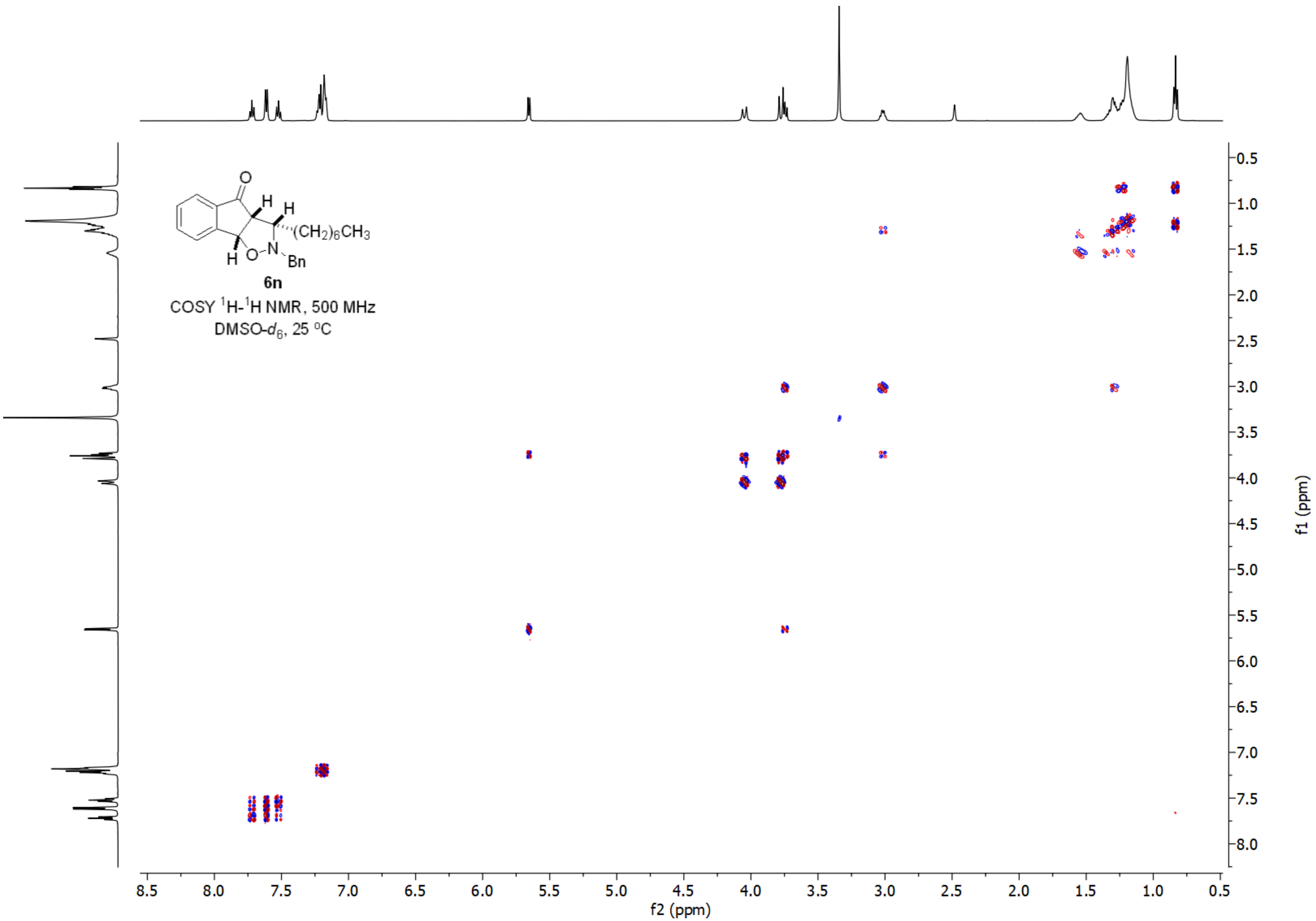

(CH2)6CH3
Bn
6n
COSY 1H-1H NMR, 500 MHz
DMSO-d6, 25 °C
f1 (ppm)
f2 (ppm)

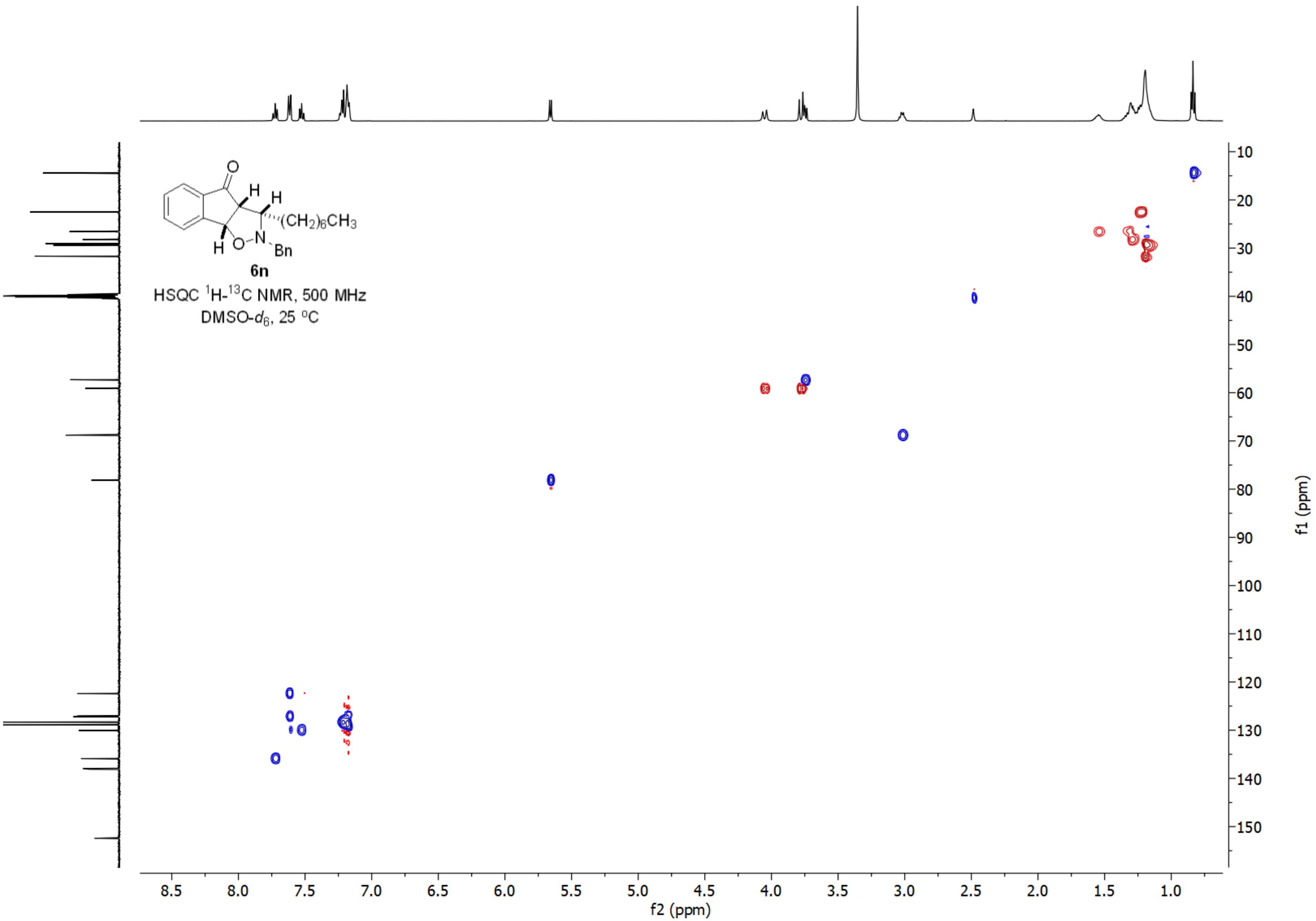
O
H
H
(CH2)6CH3
H
O
N
Bn
6n
HSQC 1H-13C NMR, 500 MHz
DMSO-d6, 25 °C
f2 (ppm)
f1 (ppm)

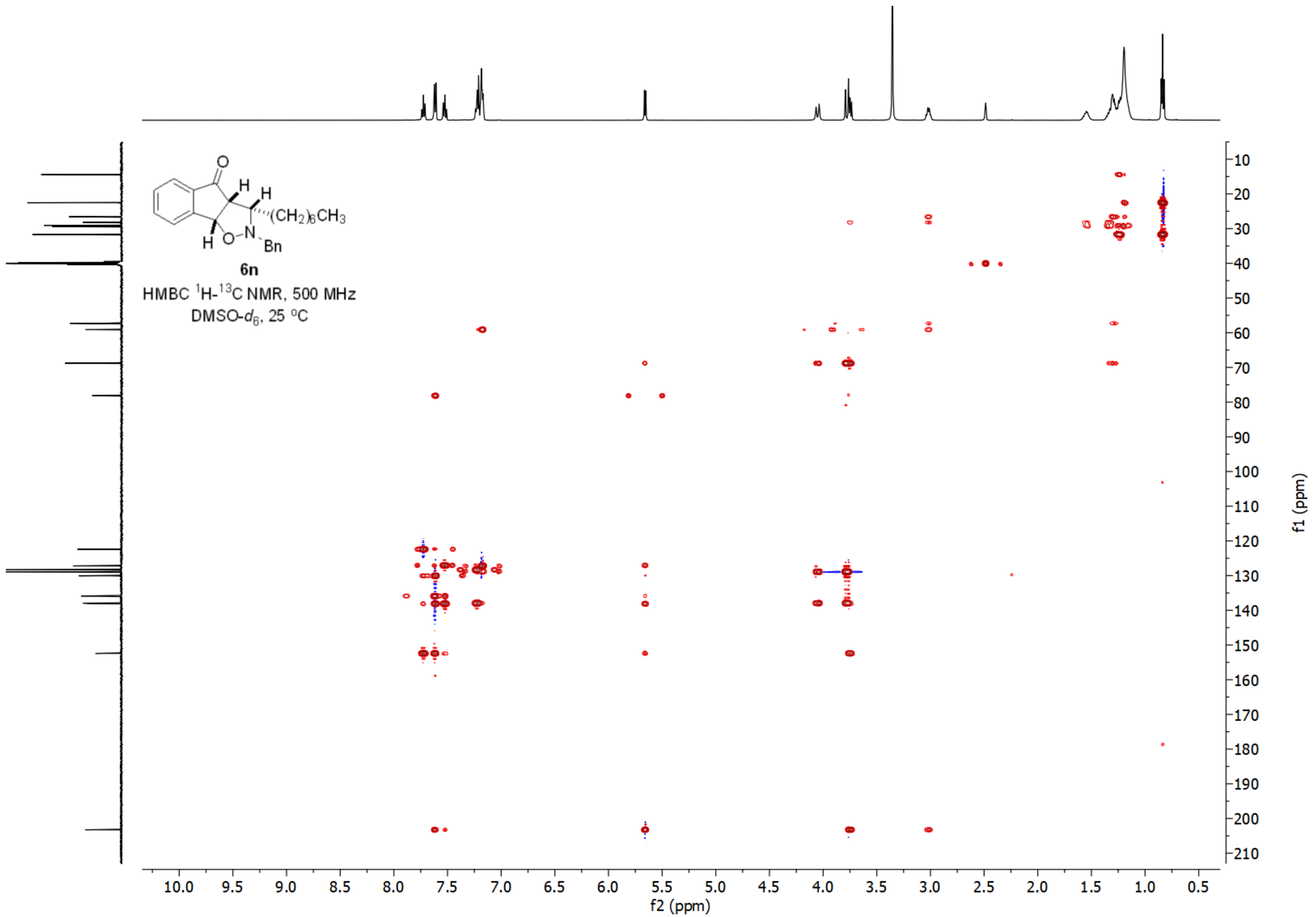

6n
HMBC ^{1}H-^{13}C NMR, 500 MHz
DMSO-d_6, 25 °C
f2 (ppm)
f1 (ppm)

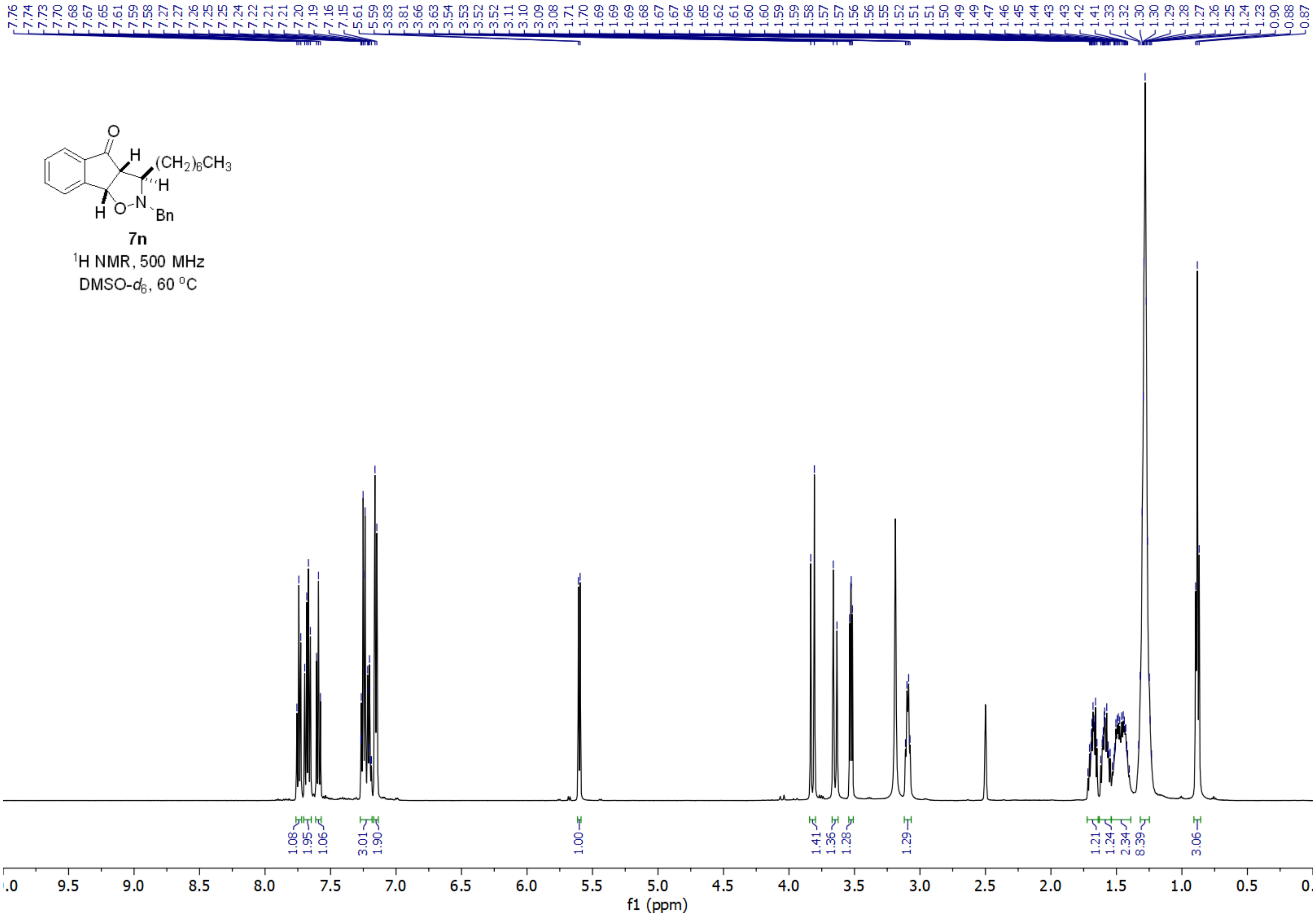
7n
¹H NMR, 500 MHz
DMSO-d6, 60 °C
f1 (ppm)

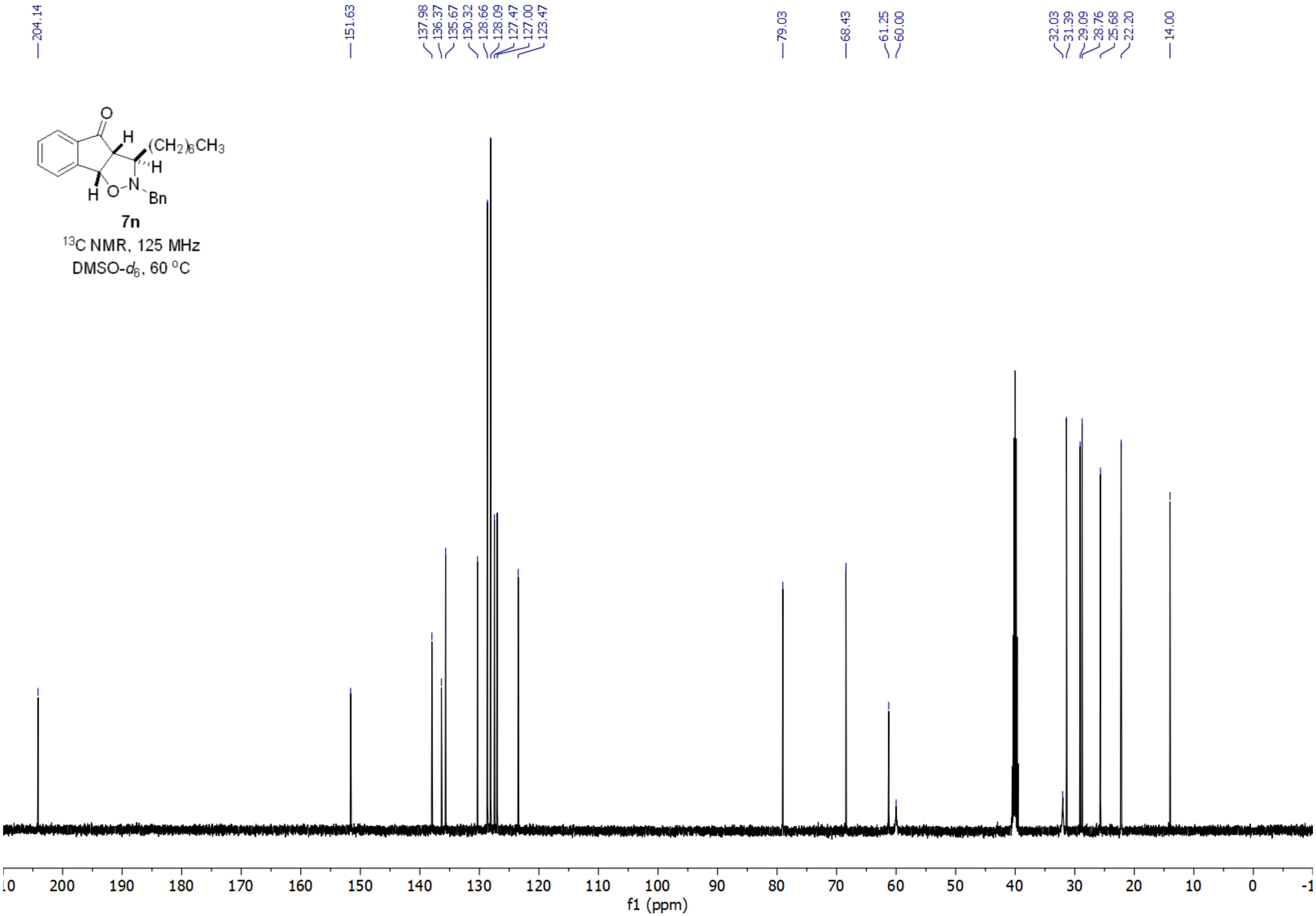
204.14
151.63
137.98
136.37
135.67
130.32
128.66
128.09
127.47
127.00
123.47
79.03
68.43
61.25
60.00
32.03
31.39
29.09
28.76
25.68
22.20
14.00
O
H
(CH2)6CH3
H
H
O
N
Bn
7n
13C NMR, 125 MHz
DMSO-d6, 60 °C
f1 (ppm)

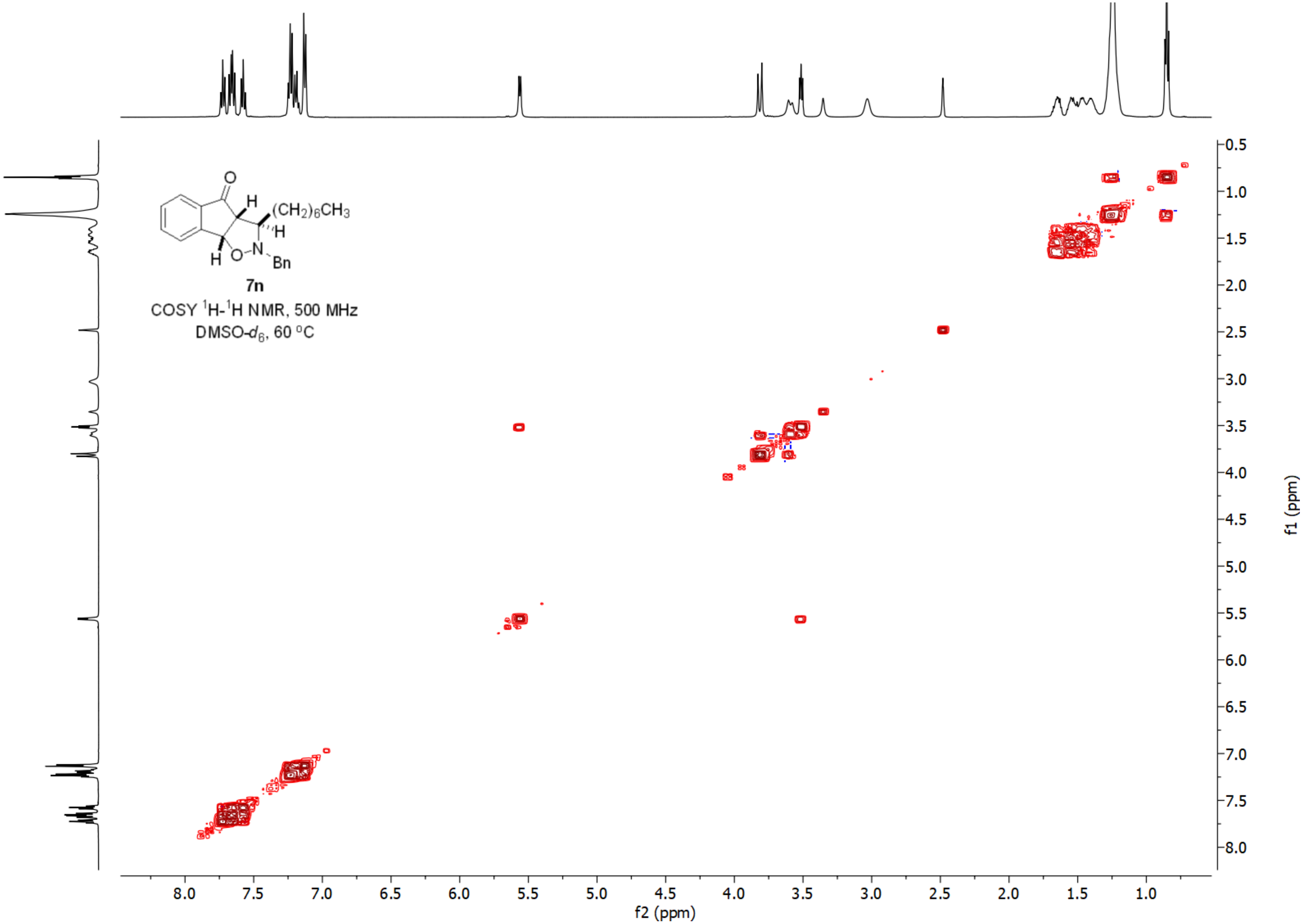

7n
COSY ^1H-^1H NMR, 500 MHz
DMSO-d_6, 60 °C
f2 (ppm)
f1 (ppm)

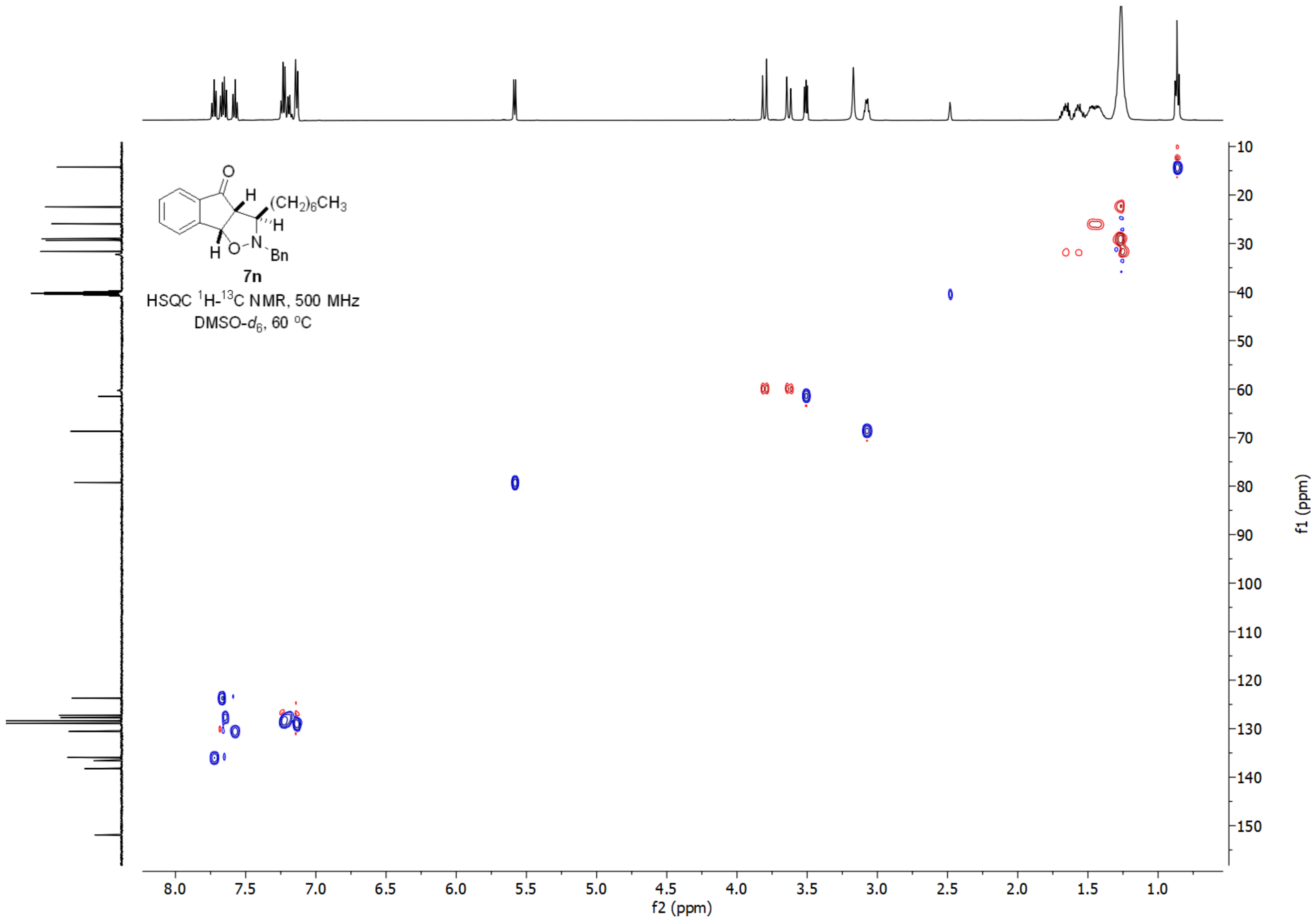

(CH2)6CH3
7n
HSQC 1H-13C NMR, 500 MHz
DMSO-d6, 60 °C
f2 (ppm)
f1 (ppm)

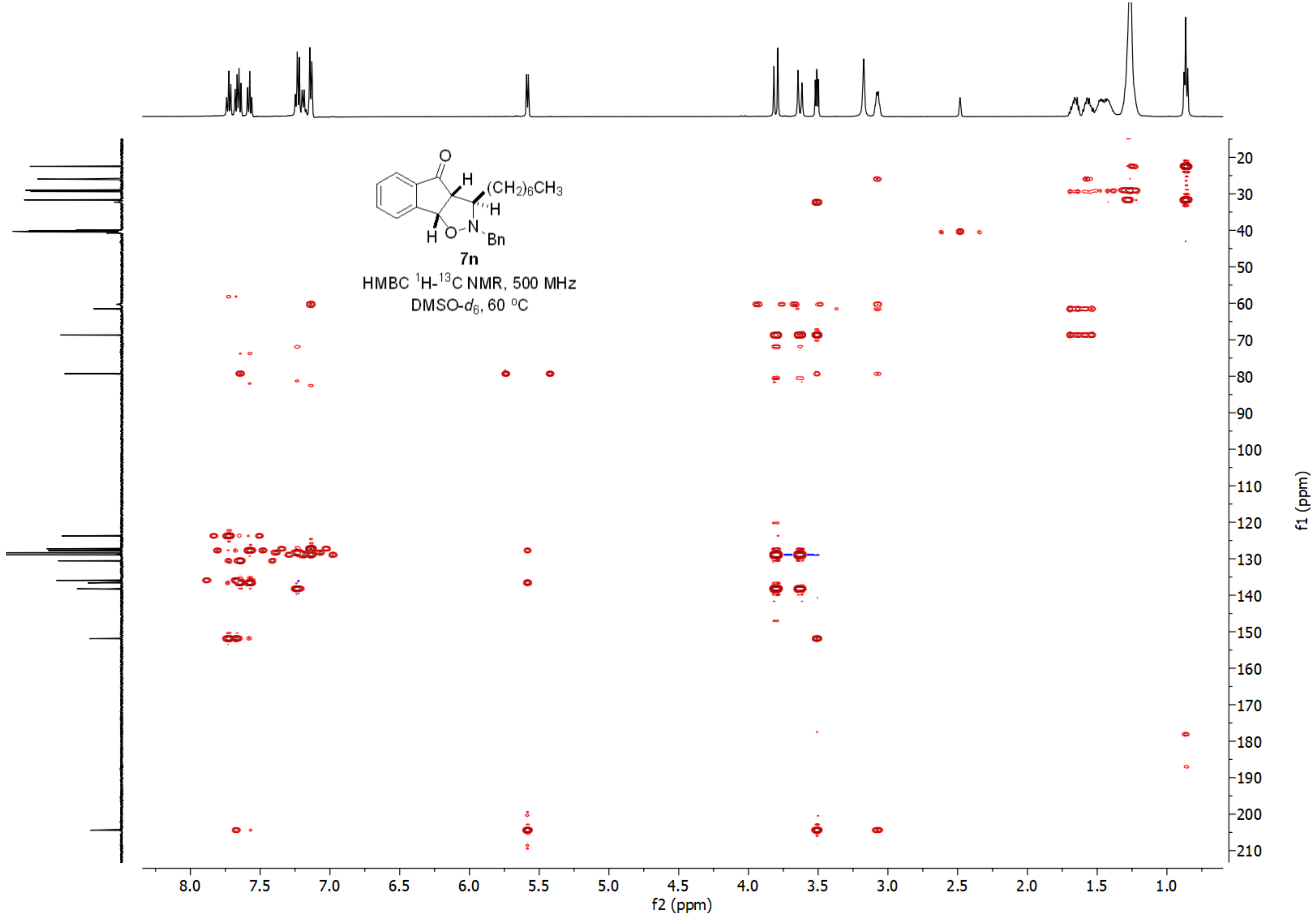

7n
HMBC ^{1}H-^{13}C NMR, 500 MHz
DMSO-d_6, 60 °C
f2 (ppm)
f1 (ppm)

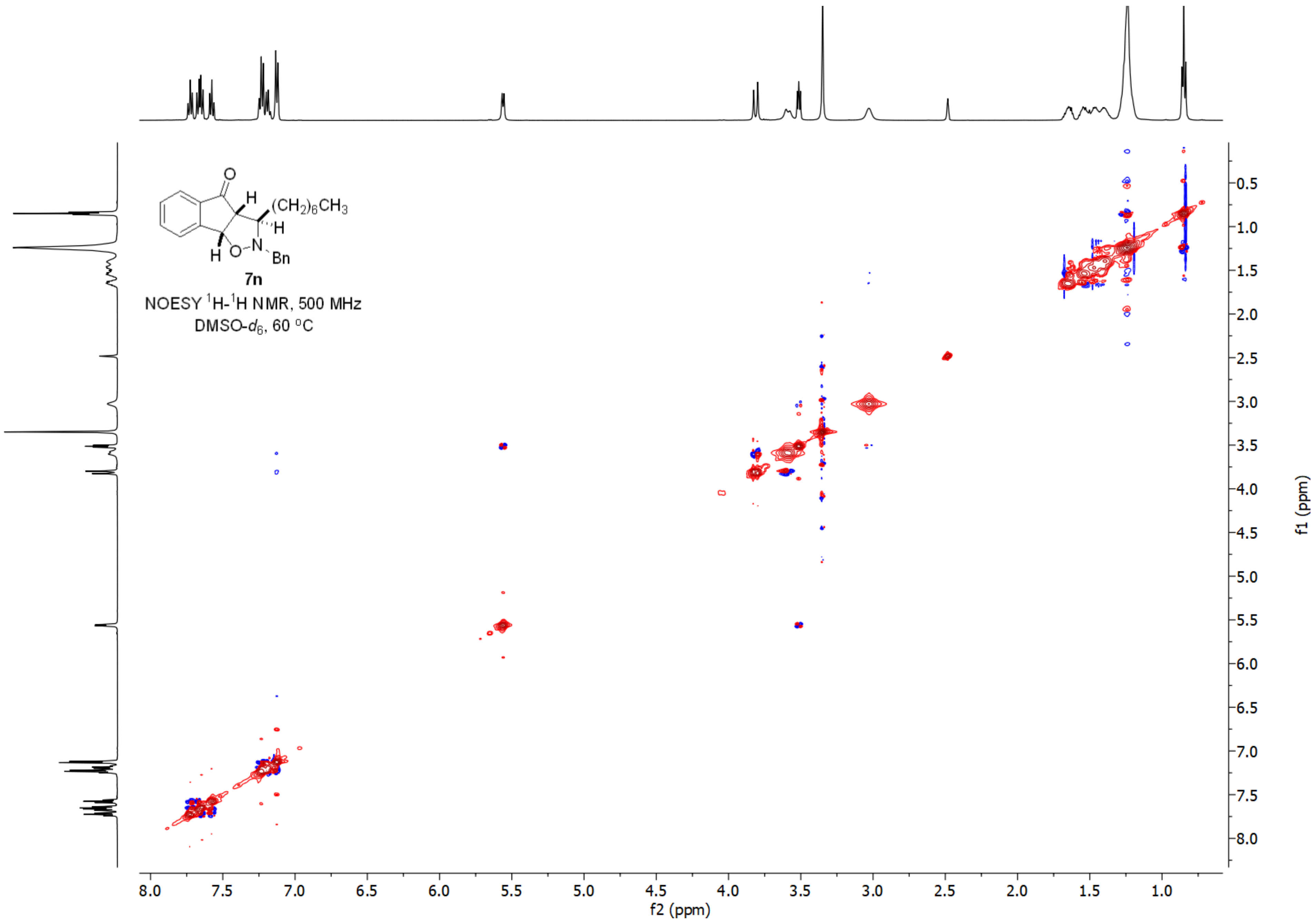
7n
NOESY 1H-1H NMR, 500 MHz
DMSO-d6, 60 °C
f2 (ppm)
f1 (ppm)

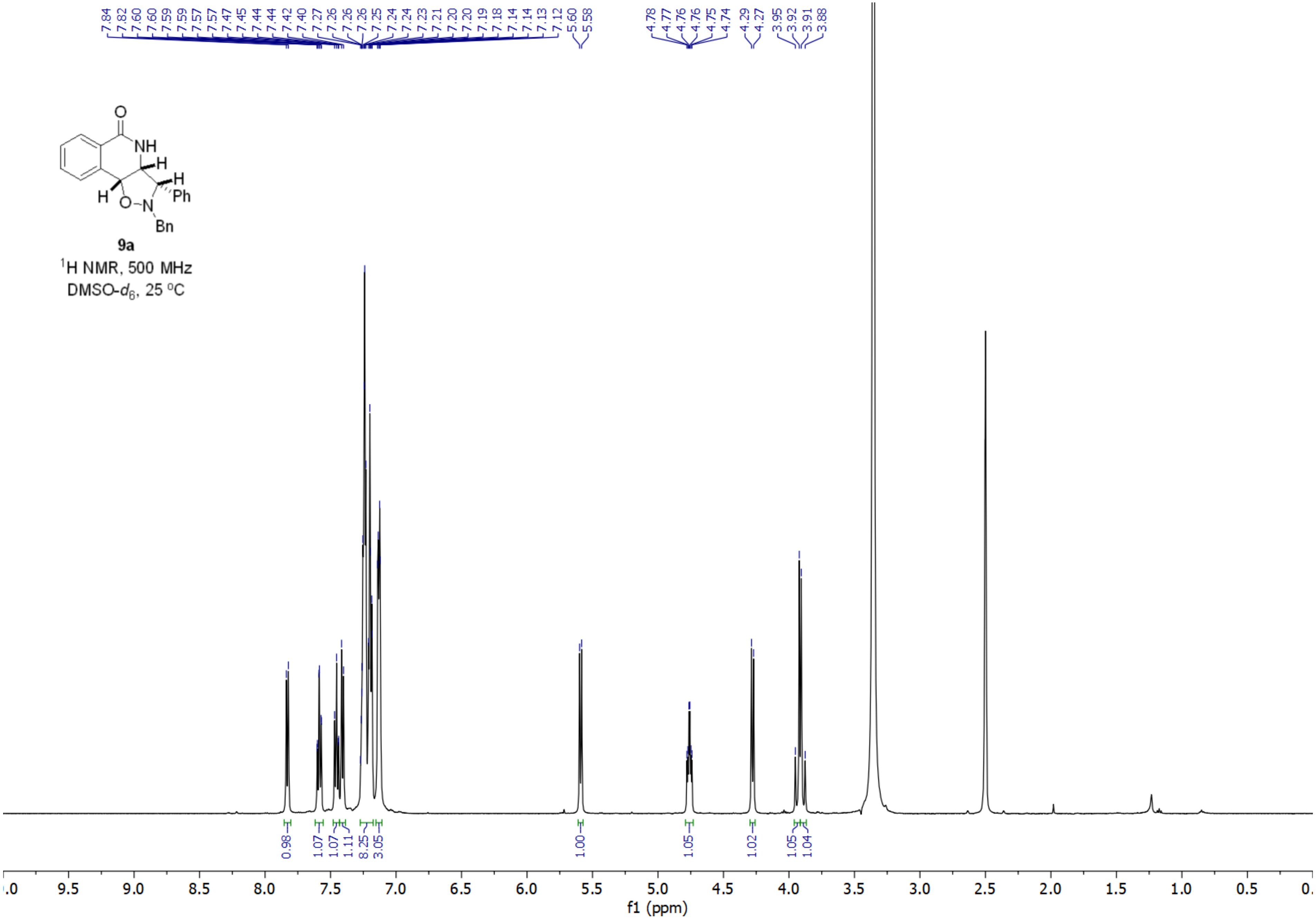
7.84 7.82 7.60 7.60 7.59 7.59 7.57 7.57 7.47 7.45 7.44 7.44 7.42 7.40 7.27 7.26 7.26 7.26 7.25 7.24 7.24 7.23 7.21 7.20 7.20 7.19 7.18 7.14 7.14 7.13 7.12 5.60 5.58
4.78 4.77 4.76 4.76 4.75 4.74 4.29 4.27 3.95 3.92 3.91 3.88
O
NH
H
H
Ph
H
O–N
Bn
9a
^{1}H NMR, 500 MHz
DMSO-d_6, 25 °C
0.98 1.07 1.07 1.11 8.25 3.05 1.00 1.05 1.02 1.05 1.04
9.5 9.0 8.5 8.0 7.5 7.0 6.5 6.0 5.5 5.0 4.5 4.0 3.5 3.0 2.5 2.0 1.5 1.0 0.5
f1 (ppm)

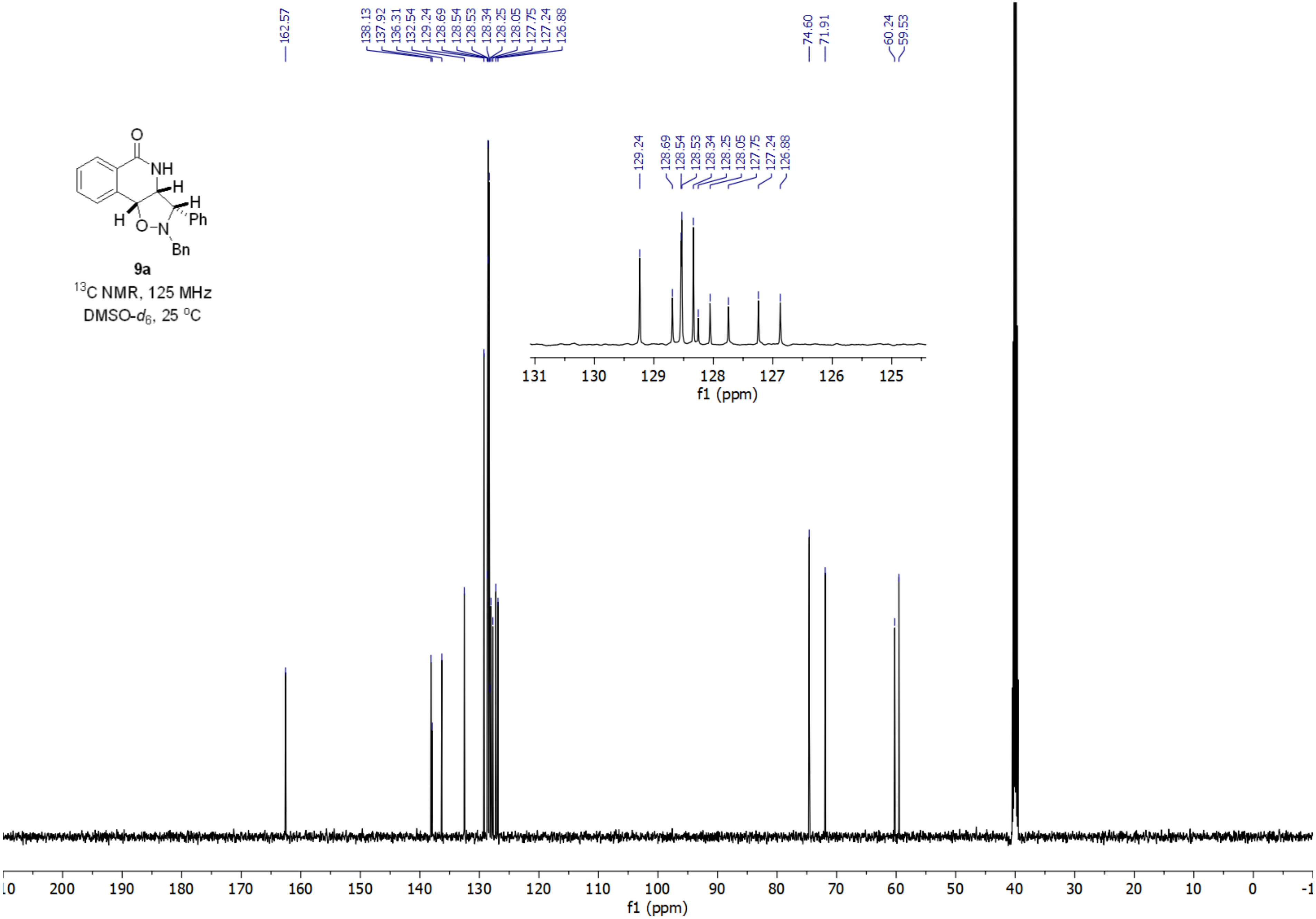

9a

^{13}C NMR, 125 MHz

DMSO-d_6, 25 °C

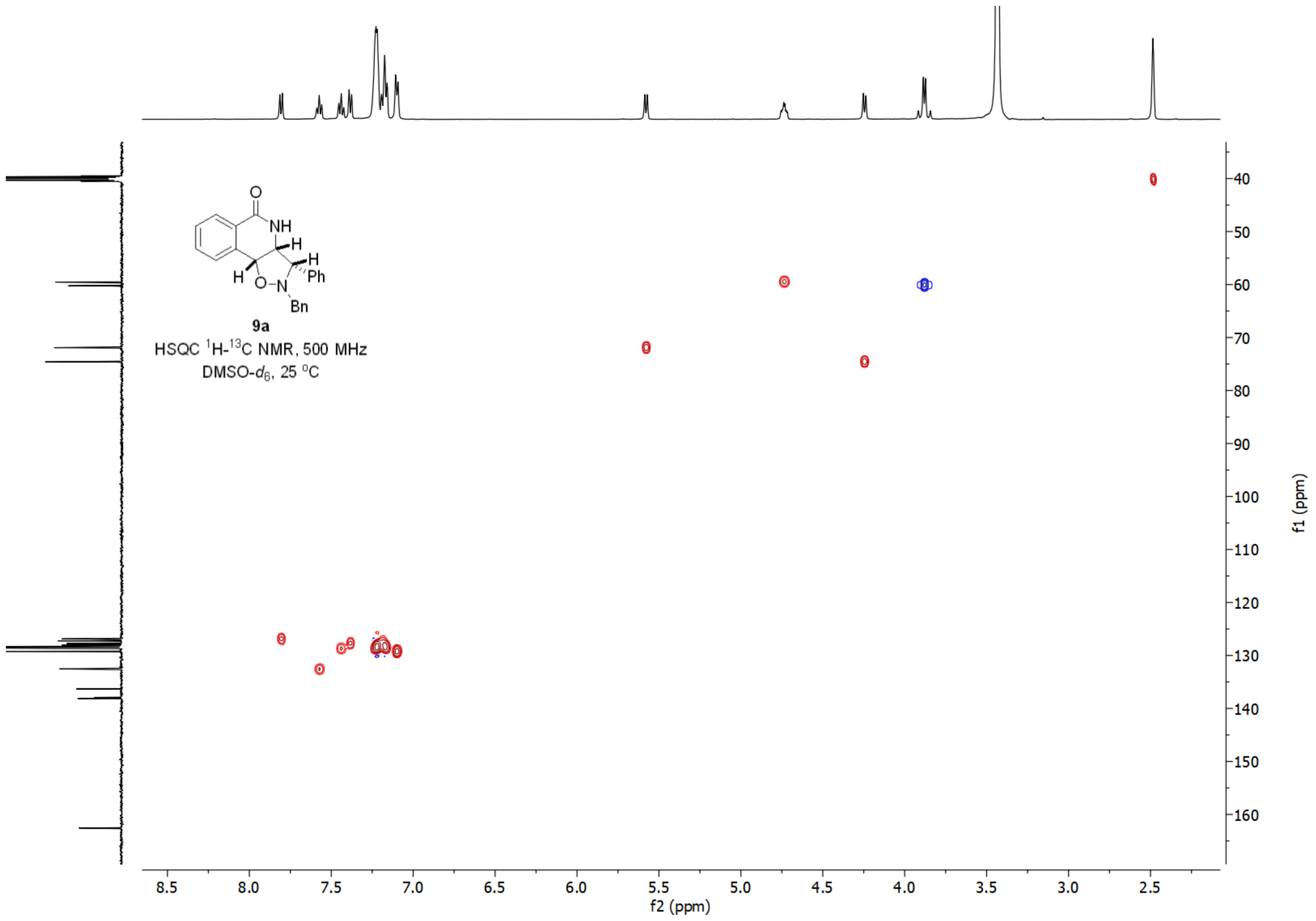
O
NH
H
H
Ph
H
O–N
Bn
9a
HSQC 1H-13C NMR, 500 MHz
DMSO-d6, 25 °C
8.5 8.0 7.5 7.0 6.5 6.0 5.5 5.0 4.5 4.0 3.5 3.0 2.5
f2 (ppm)
40 50 60 70 80 90 100 110 120 130 140 150 160
f1 (ppm)

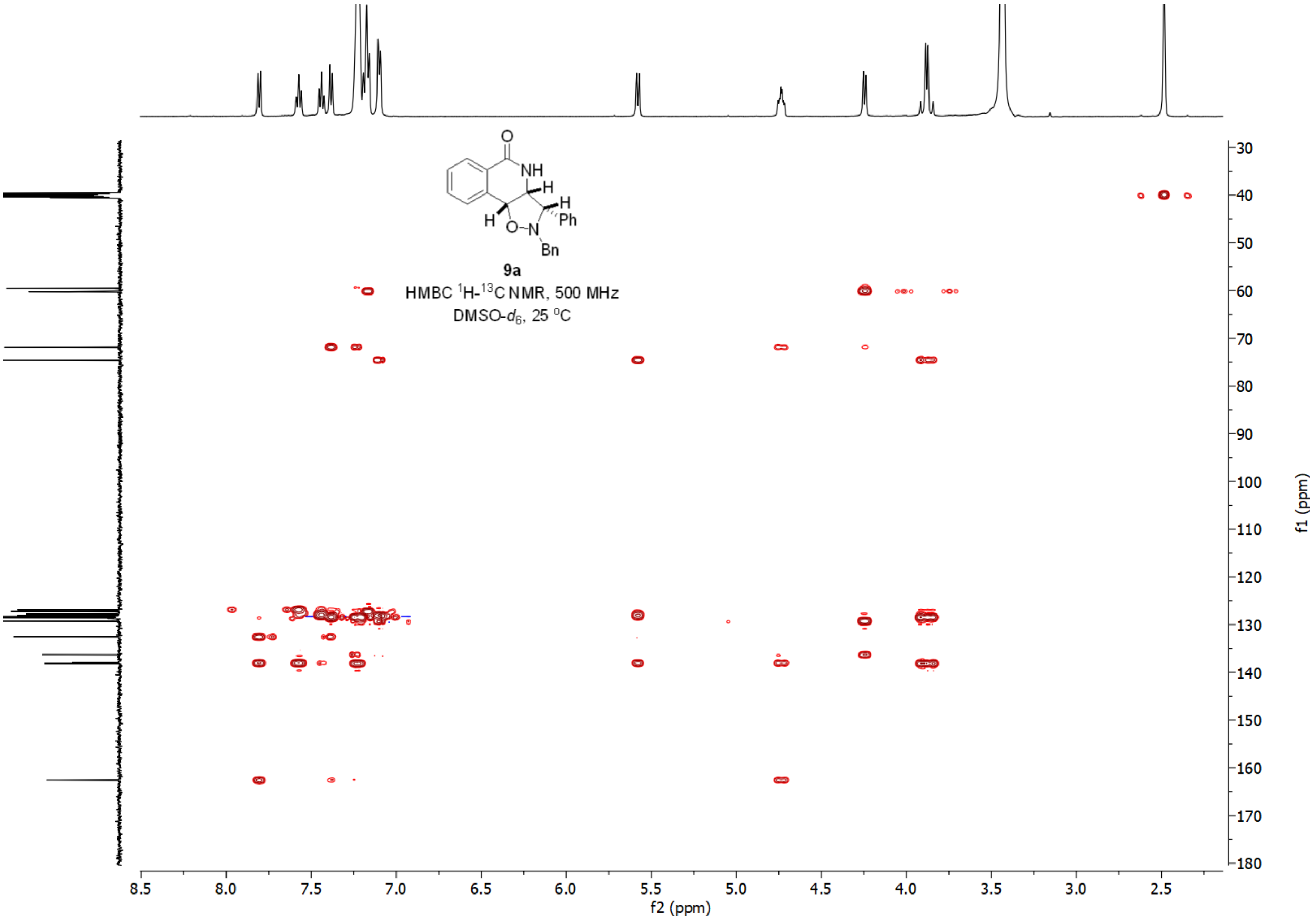

O
NH
H
H
H
Ph
O
N
Bn
9a
HMBC ¹H-¹³C NMR, 500 MHz
DMSO-d6, 25 °C
f2 (ppm)
f1 (ppm)

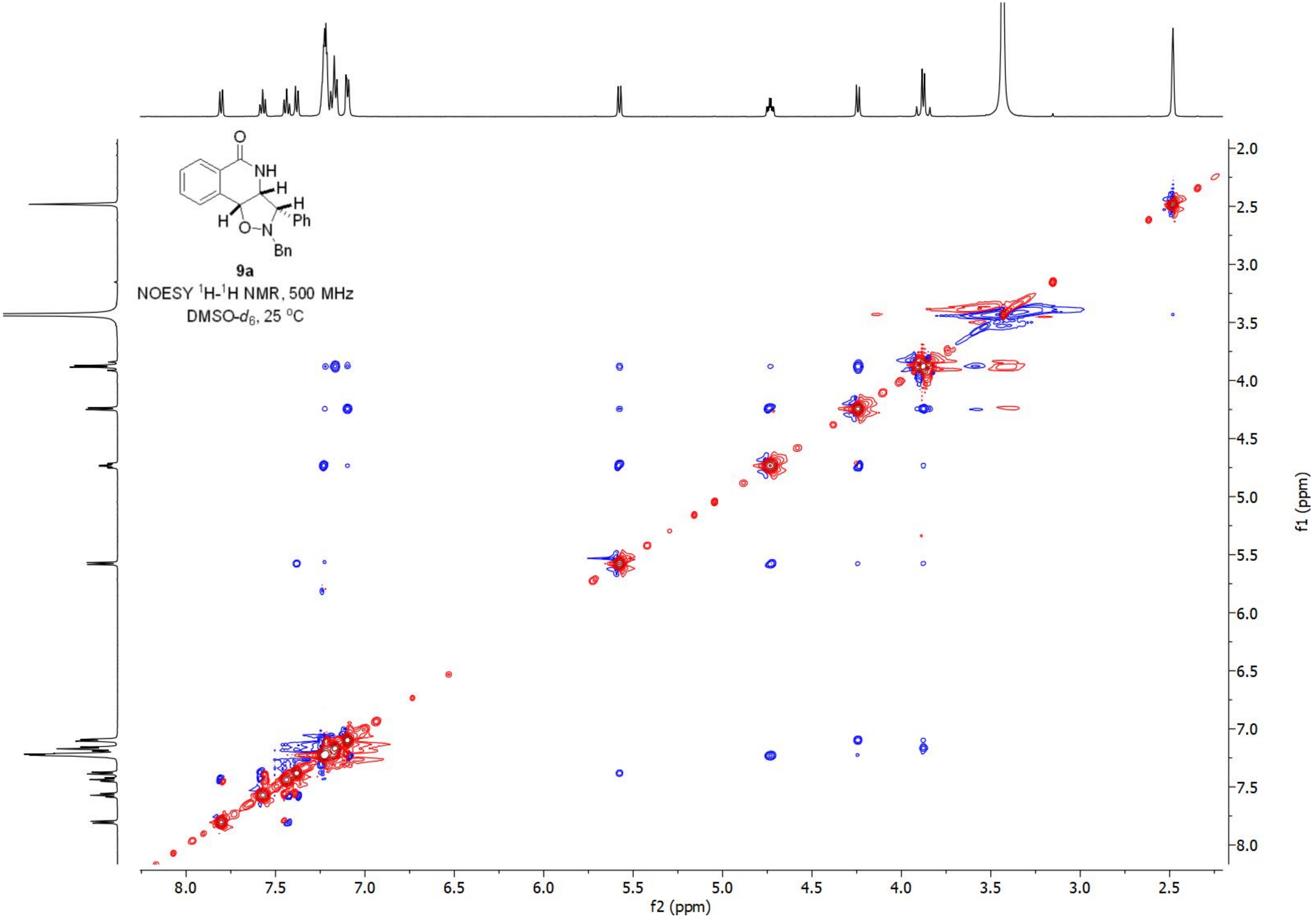

O
NH
H
H
H
Ph
O-N
Bn
9a
NOESY ^{1}H-^{1}H NMR, 500 MHz
DMSO-d_6, 25 ^{o}C
f1 (ppm)
f2 (ppm)

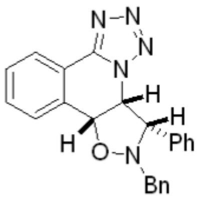

10a

^1H NMR, 500 MHz

$CDCl_3$, 25 °C

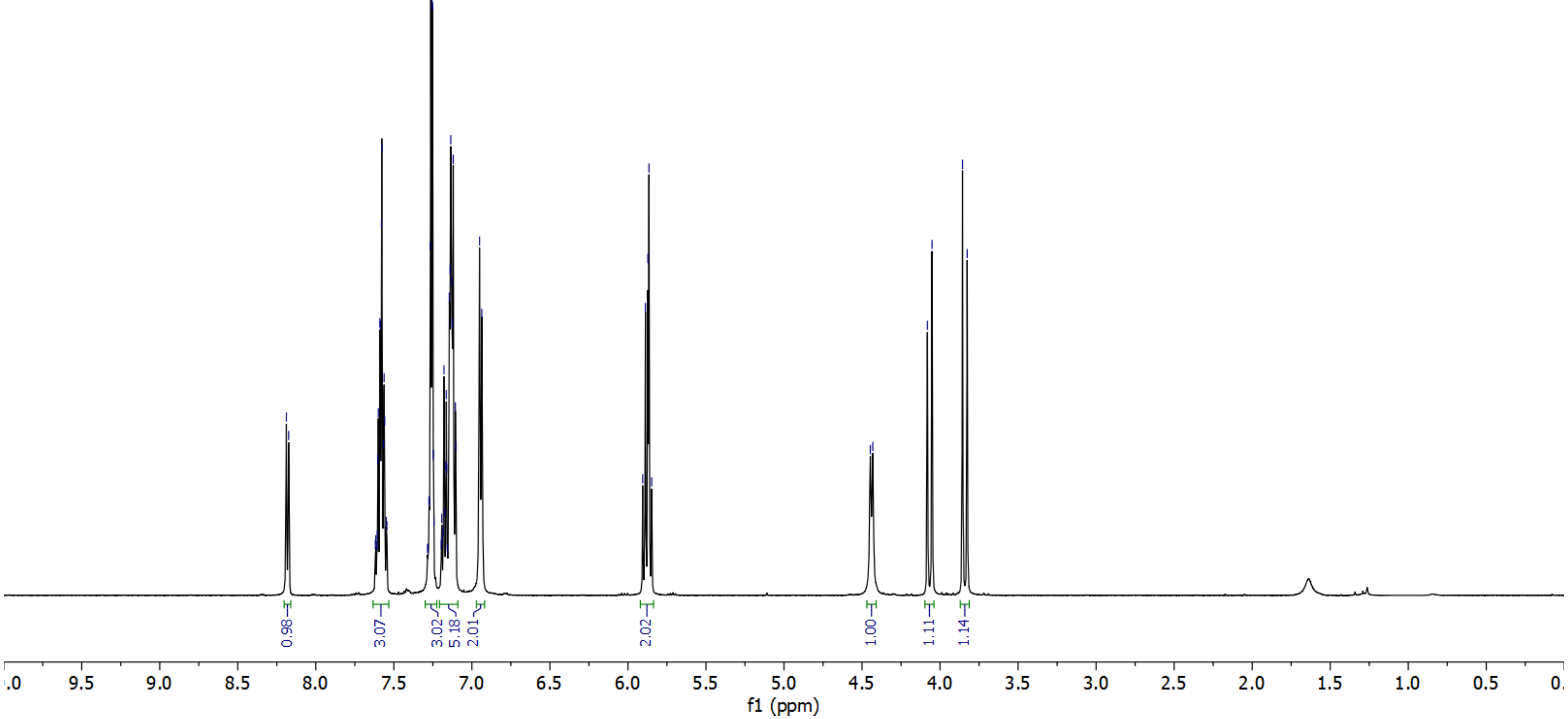

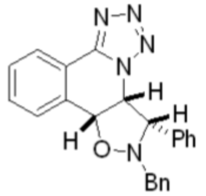

10a
^{13}C NMR, 125 MHz
$CDCl_3$, 25 °C

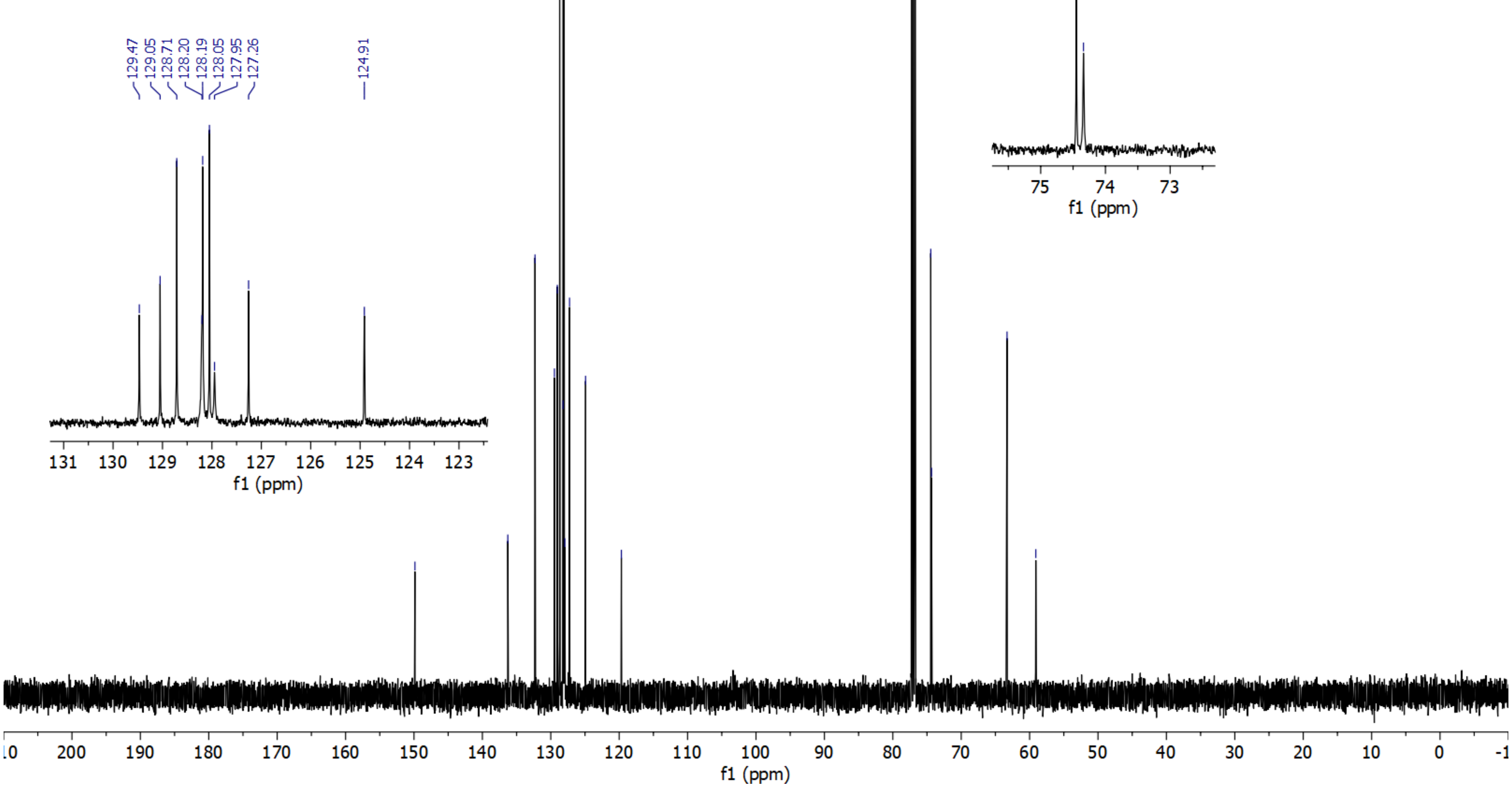

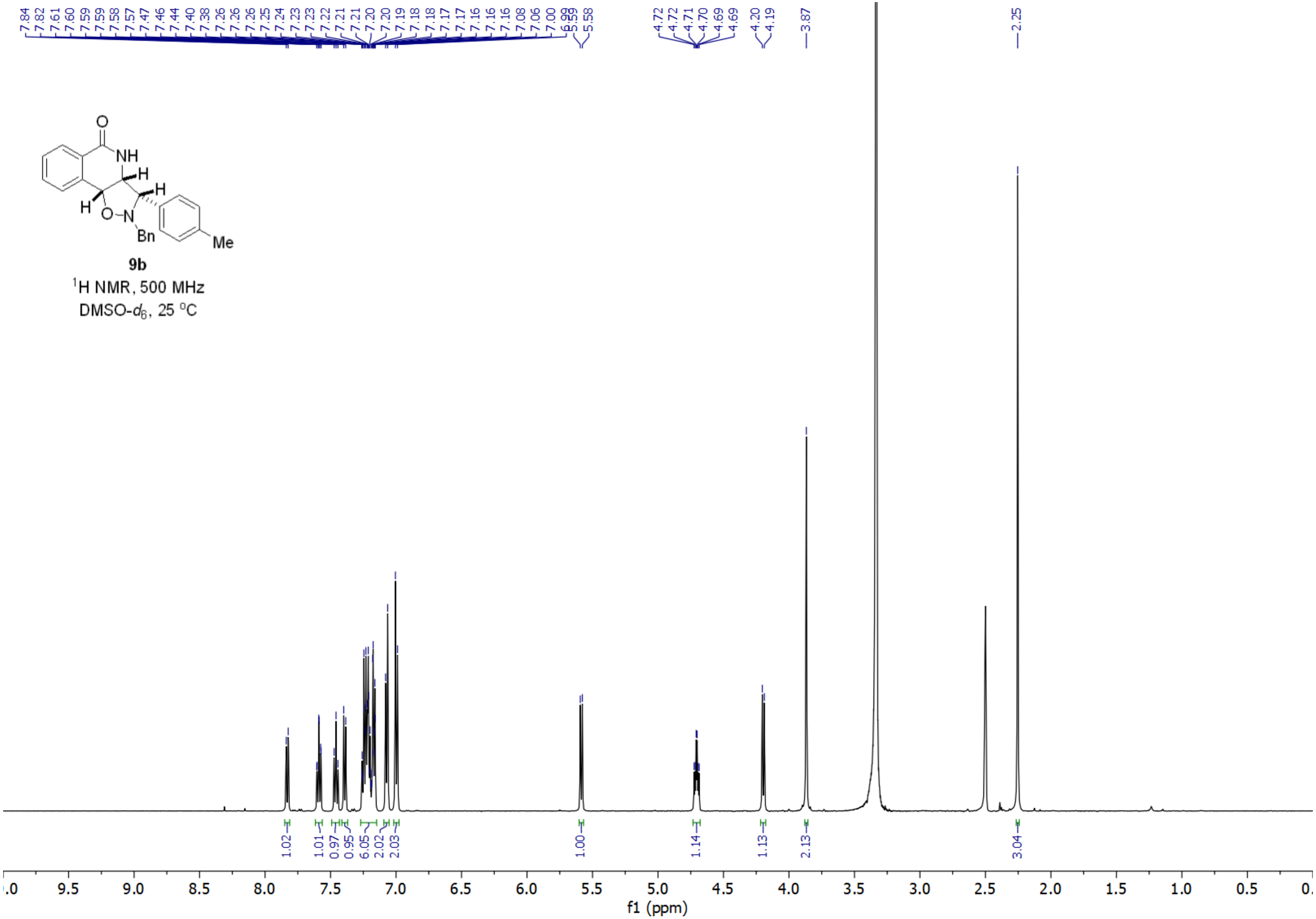

9b

^{1}H NMR, 500 MHz

DMSO-d_6, 25 °C

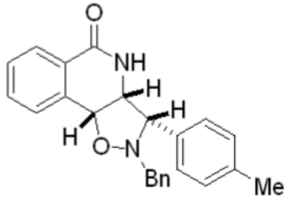

9b

^{13}C NMR, 125 MHz

DMSO-d_6, 25 °C

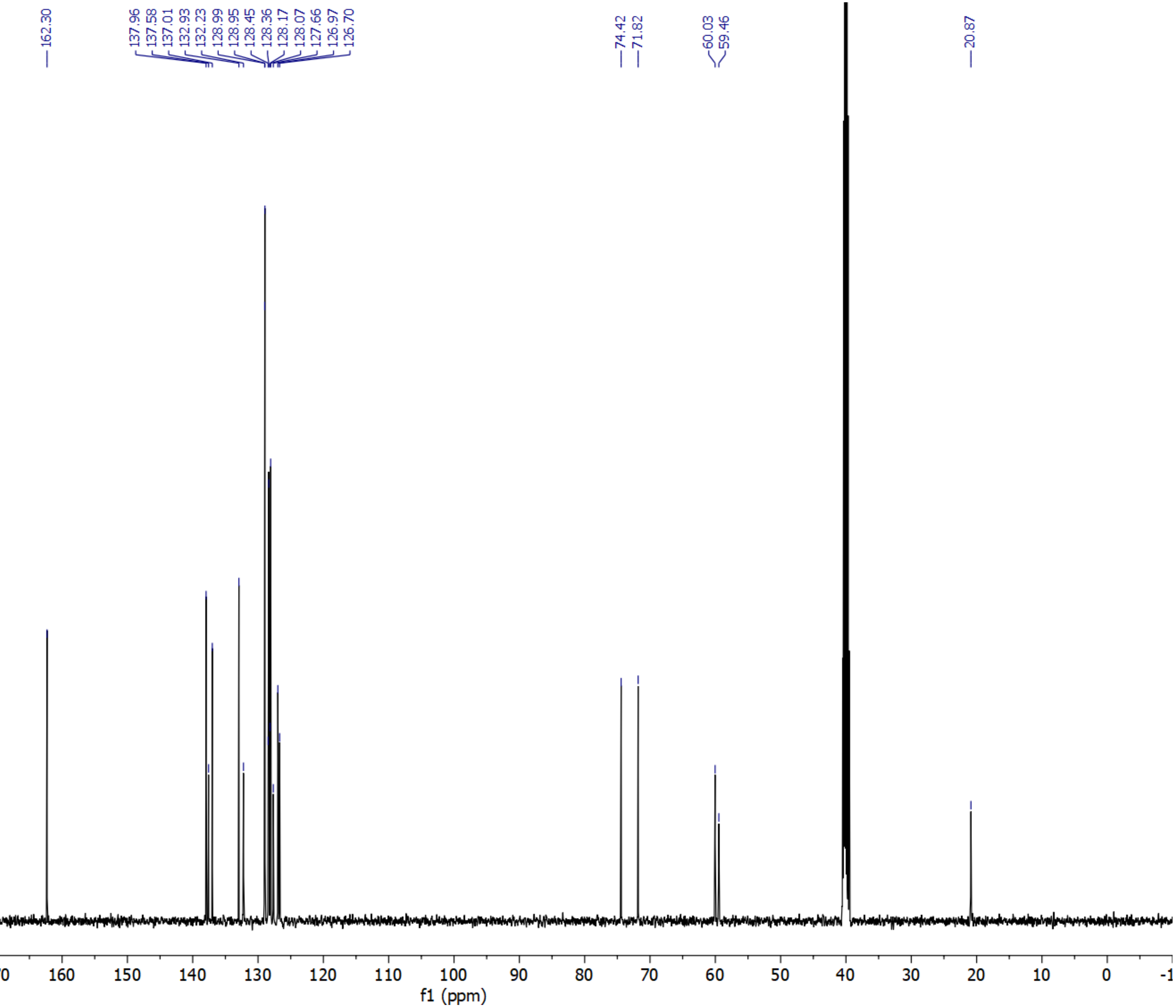

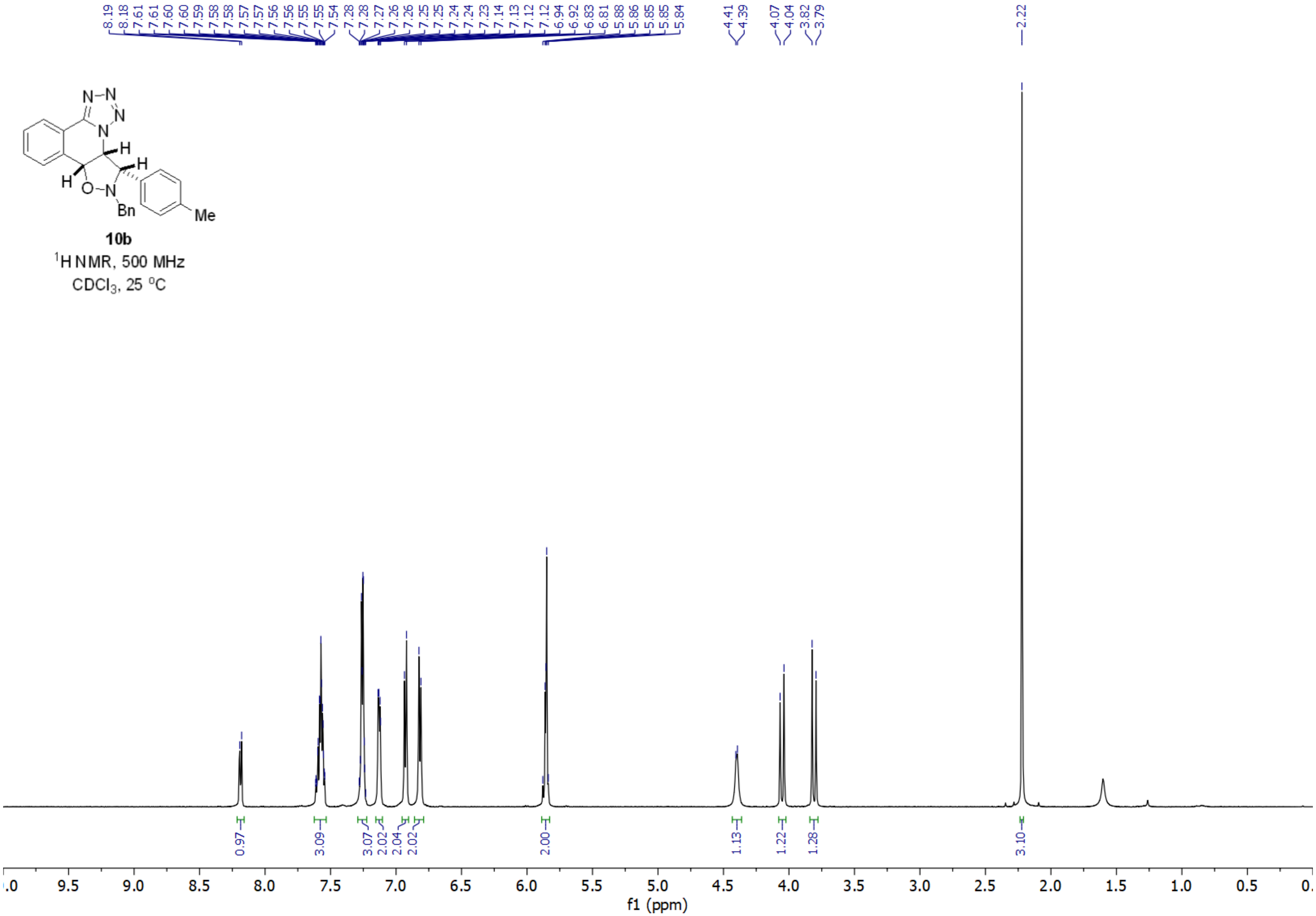

10b

^{1}H NMR, 500 MHz

$CDCl_3$, 25 °C

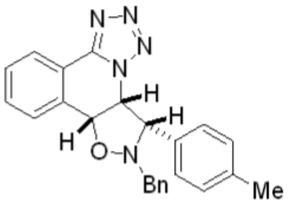

10b

^{13}C NMR, 125 MHz

$CDCl_3$, 25 °C

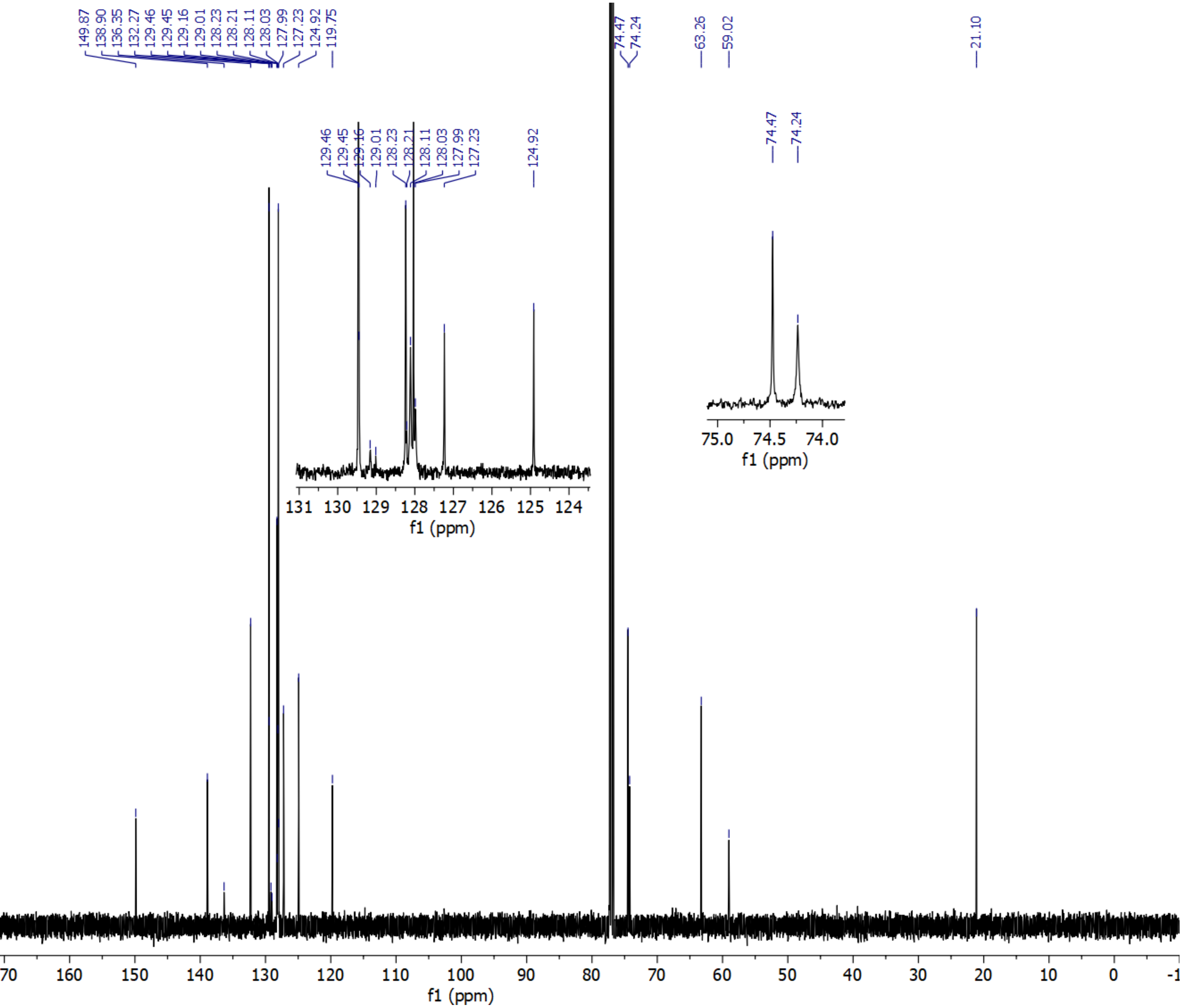

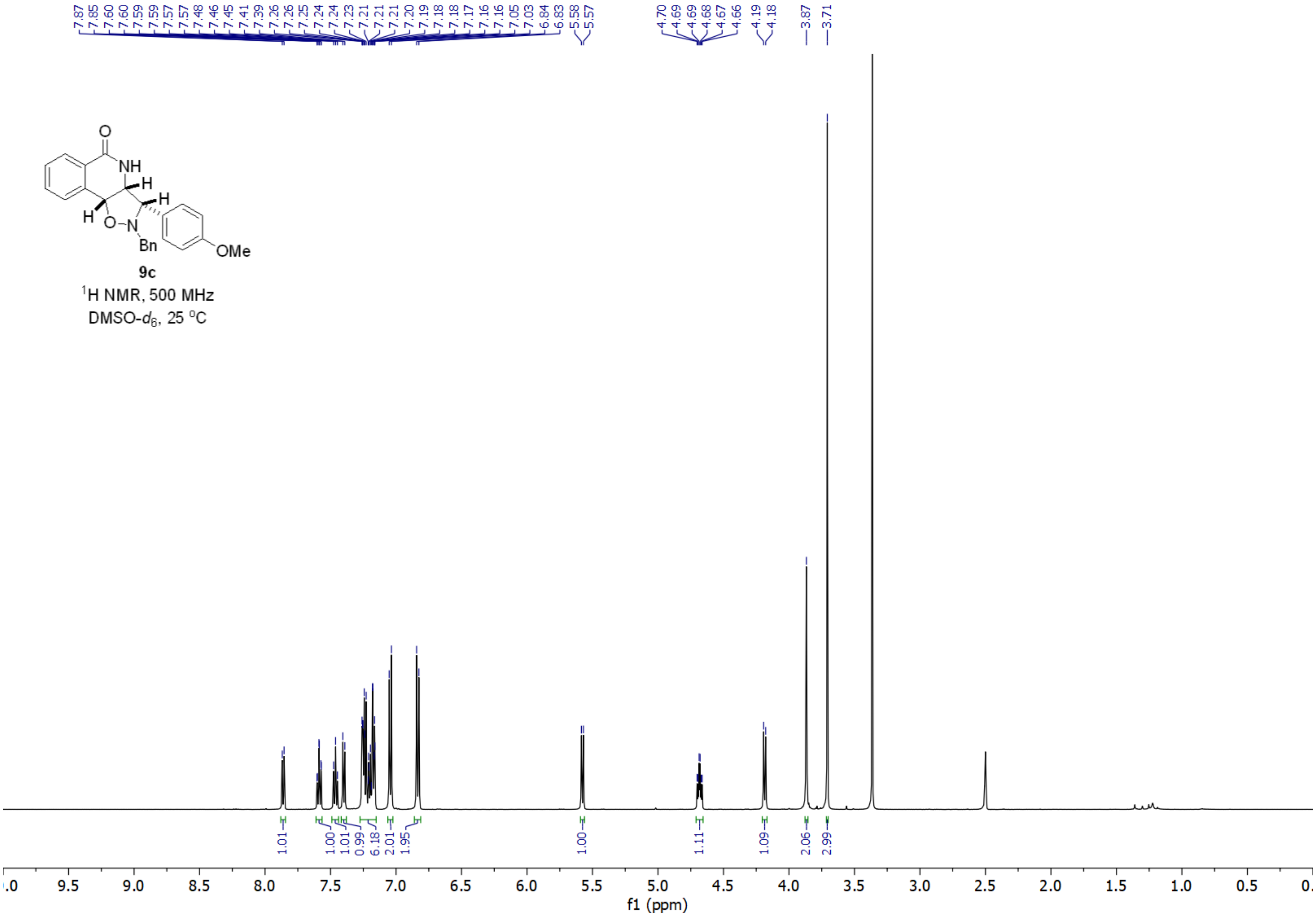

7.87 7.85 7.60 7.59 7.59 7.57 7.57 7.48 7.46 7.45 7.41 7.39 7.26 7.26 7.25 7.24 7.24 7.23 7.21 7.21 7.21 7.20 7.19 7.18 7.18 7.17 7.16 7.16 7.05 7.03 6.84 6.83 5.58 5.57
4.70 4.69 4.69 4.68 4.67 4.66 4.19 4.18
3.87
3.71
O
NH
H
H
H
O
N
Bn
OMe
9c
^{1}H NMR, 500 MHz
DMSO-d_6, 25 ^{o}C
1.01 1.00 1.01 0.99 6.18 2.01 1.95 1.00 1.11 1.09 2.06 2.99
.0 9.5 9.0 8.5 8.0 7.5 7.0 6.5 6.0 5.5 5.0 4.5 4.0 3.5 3.0 2.5 2.0 1.5 1.0 0.5 0.
f1 (ppm)

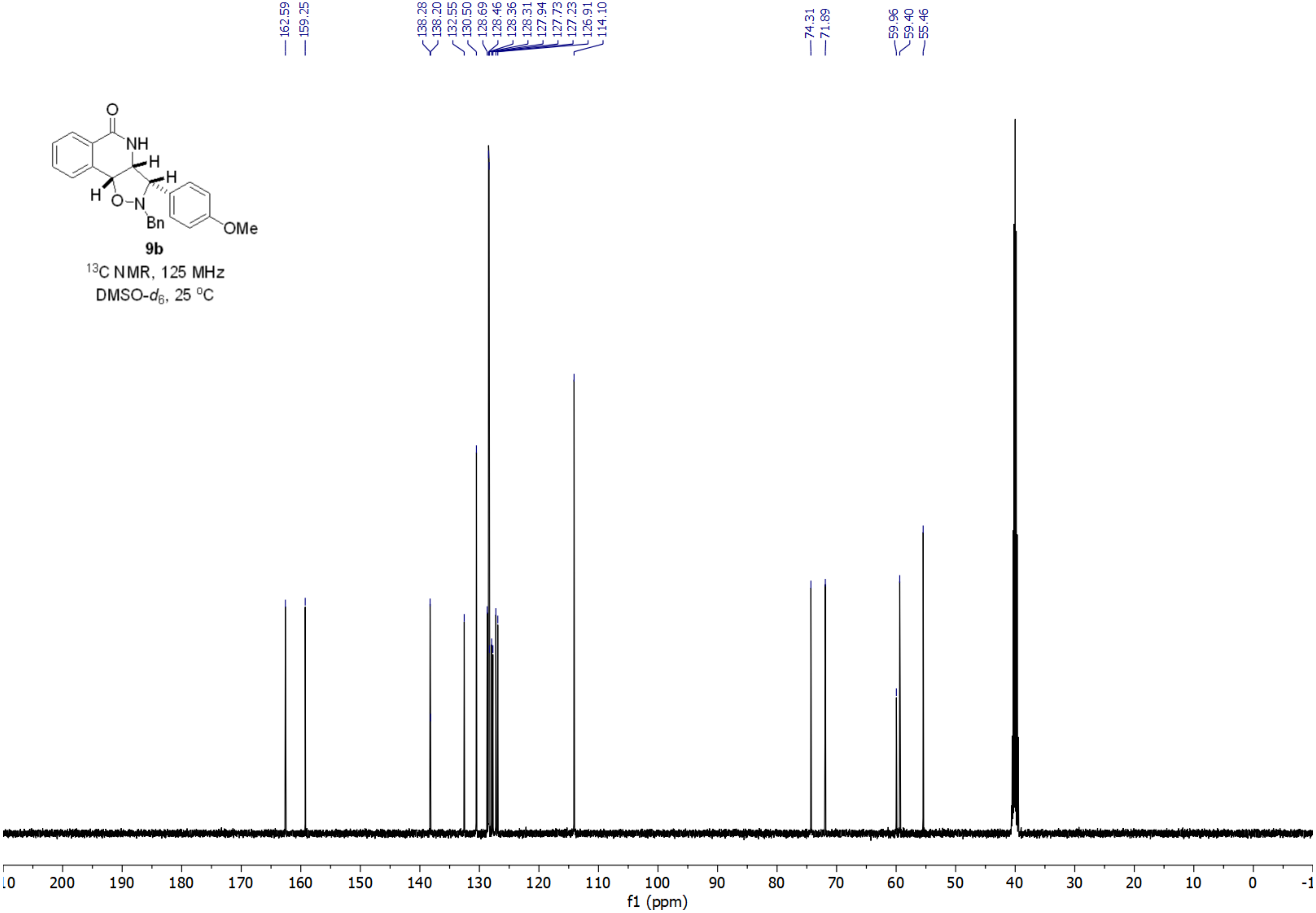
162.59
159.25
138.28
138.20
132.55
130.50
128.69
128.46
128.36
128.31
127.94
127.73
127.23
126.91
114.10
74.31
71.89
59.96
59.40
55.46
O
NH
H
H
H
O
N
Bn
OMe
9b
^{13}C NMR, 125 MHz
DMSO-d_6, 25 °C
f1 (ppm)

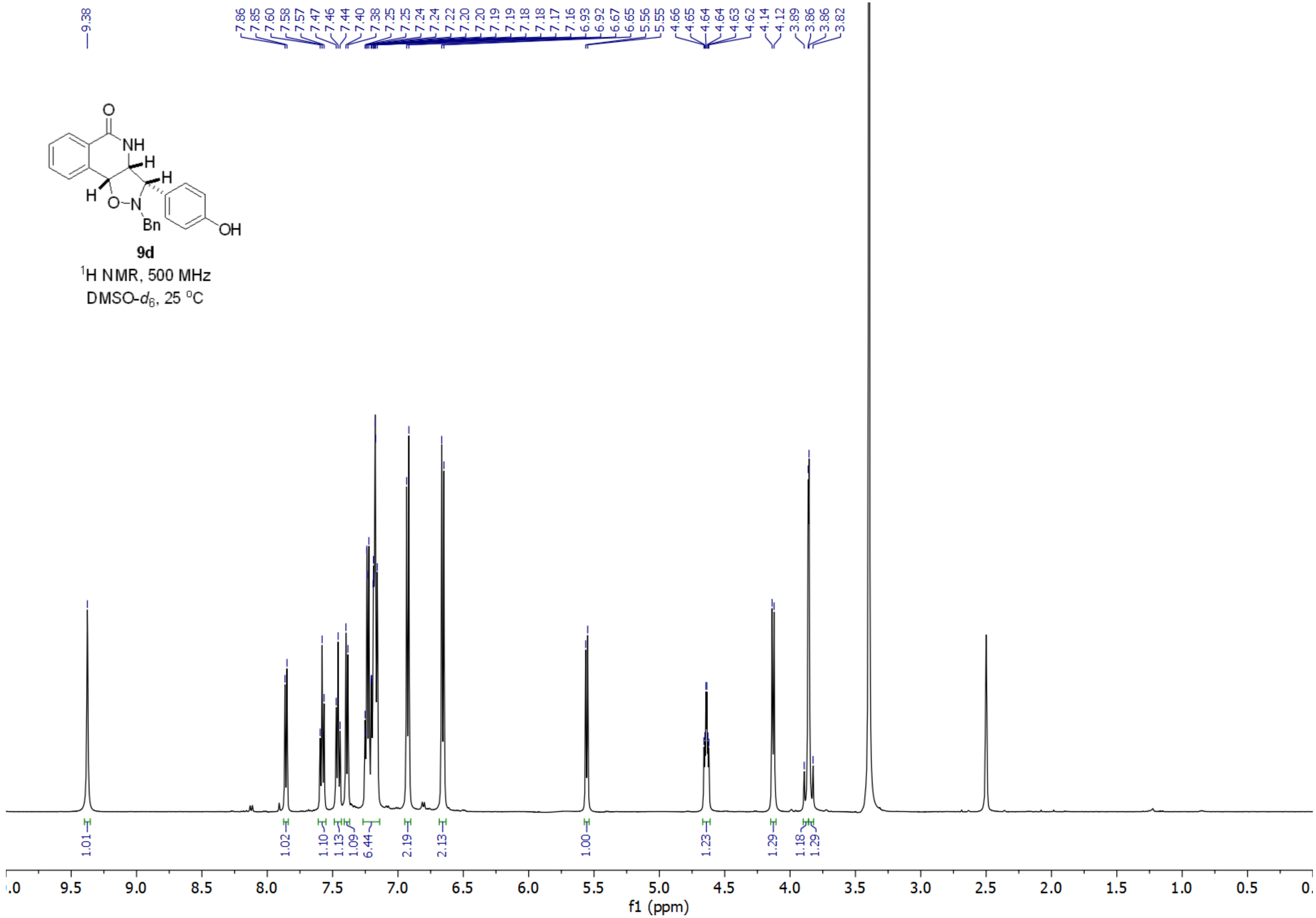
9d
¹H NMR, 500 MHz
DMSO-d6, 25 °C
f1 (ppm)

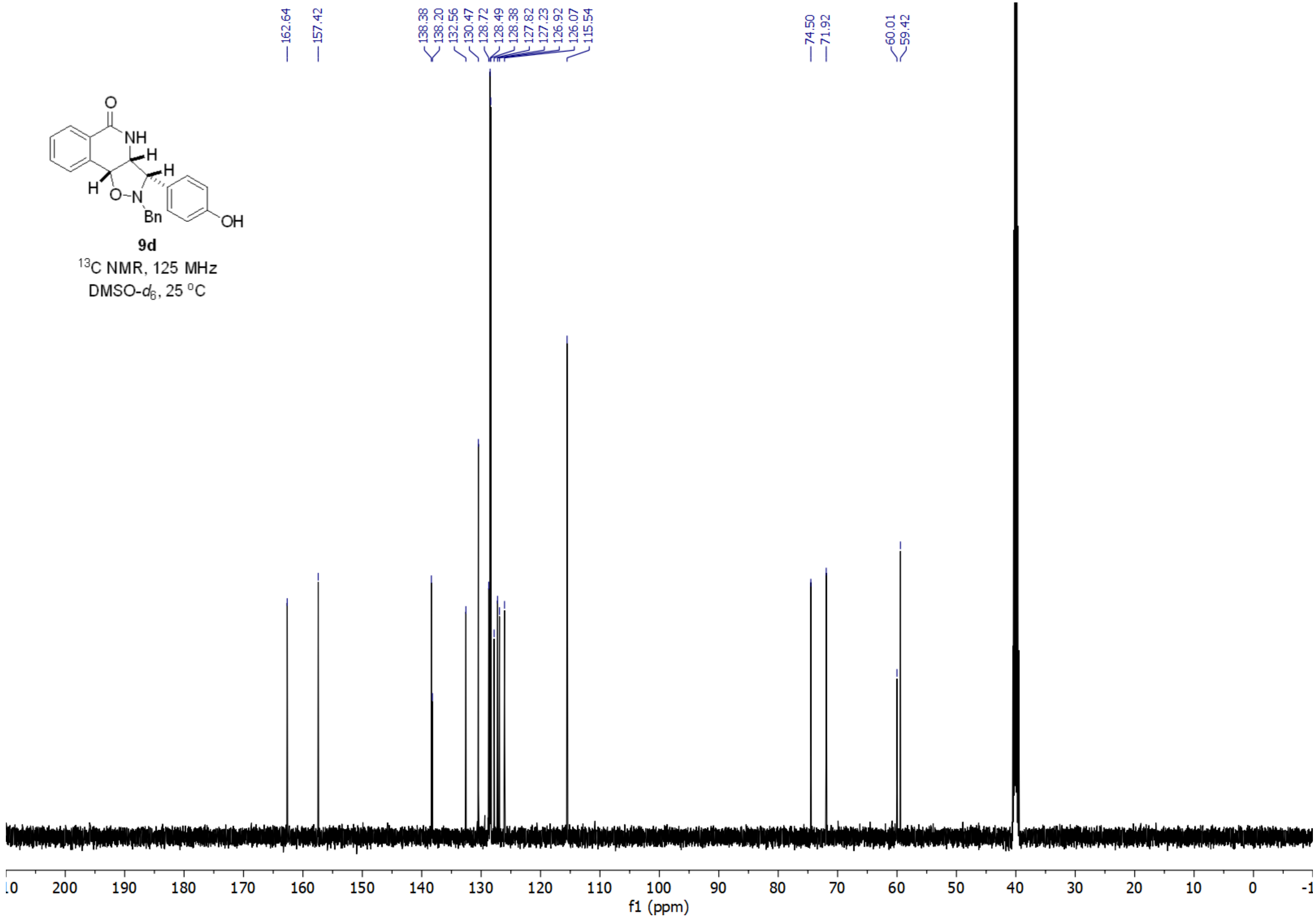
162.64
157.42
138.38
138.20
132.56
130.47
128.72
128.49
128.38
127.82
127.23
126.92
126.07
115.54
74.50
71.92
60.01
59.42
O
NH
H
H
H
O
N
Bn
OH
9d
^{13}C NMR, 125 MHz
DMSO-d_6, 25 °C
f1 (ppm)

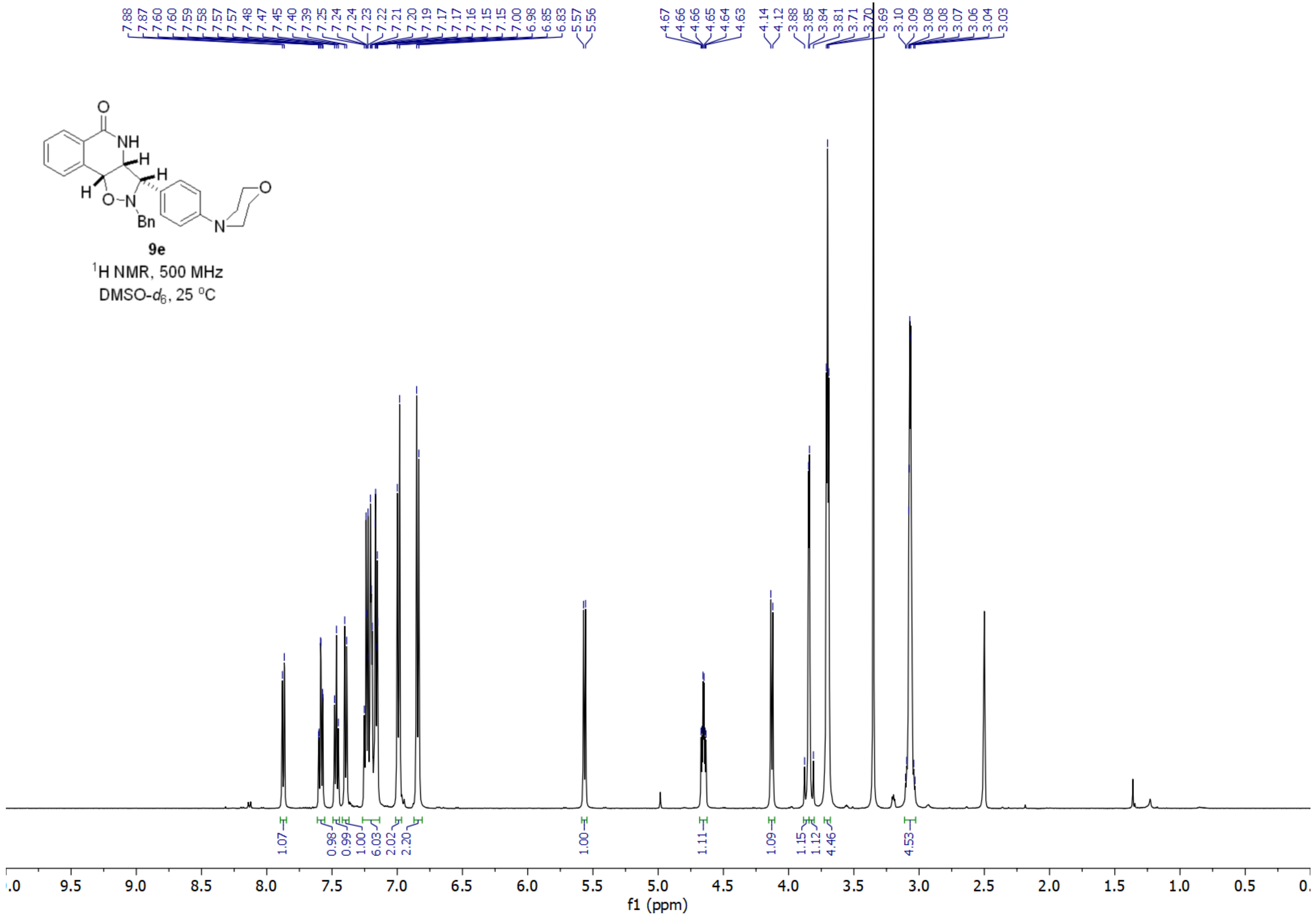
9e
¹H NMR, 500 MHz
DMSO-d6, 25 °C
f1 (ppm)

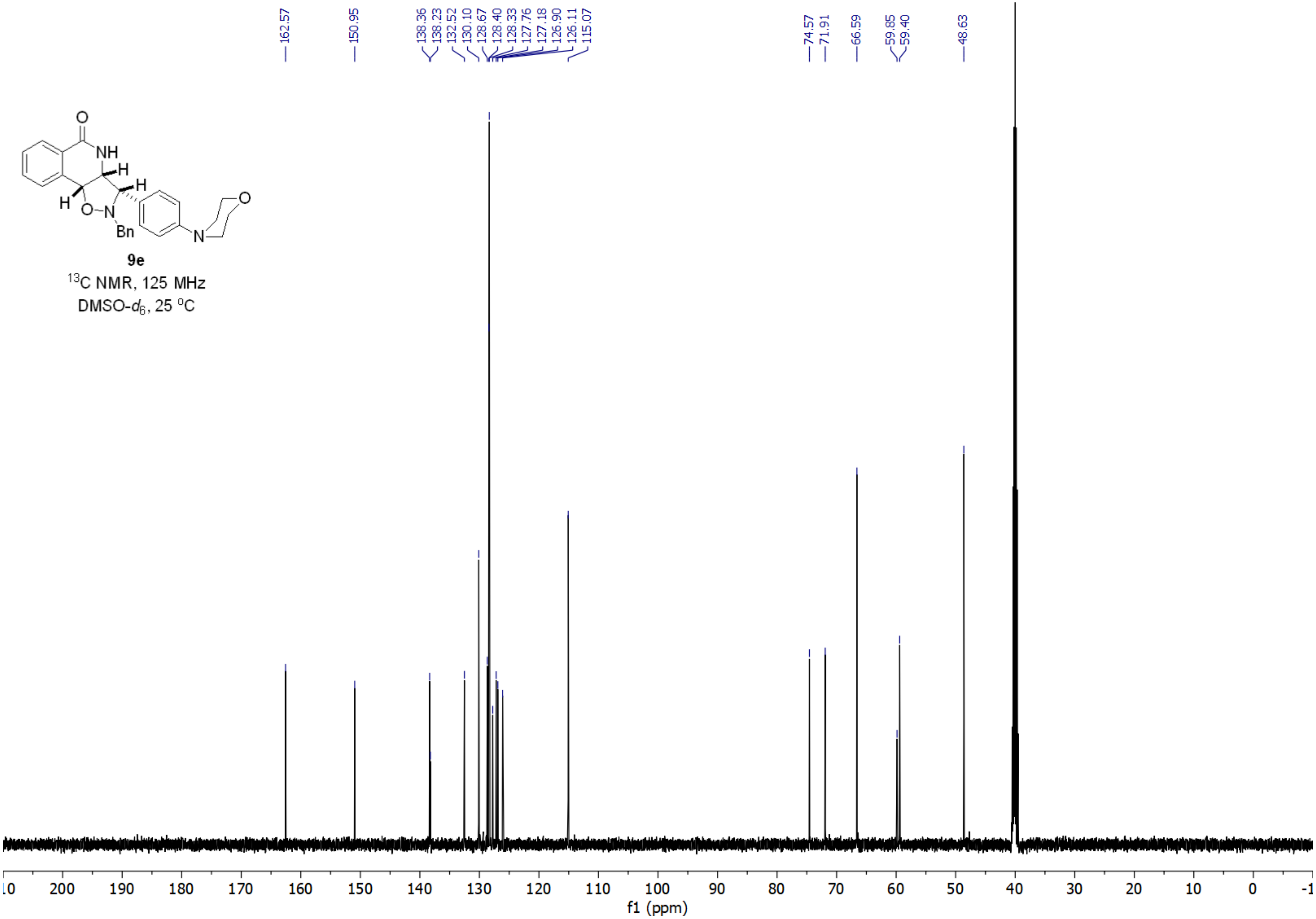
162.57
150.95
138.36
138.23
132.52
130.10
128.67
128.40
128.33
127.76
127.18
126.90
126.11
115.07
74.57
71.91
66.59
59.85
59.40
48.63
O
NH
H
H
H
O–N
Bn
N
O
9e
^{13}C NMR, 125 MHz
DMSO-d_6, 25 °C
f1 (ppm)

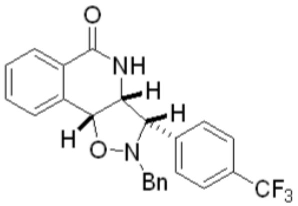

9f

^{1}H NMR, 500 MHz

DMSO-d_6, 25 °C

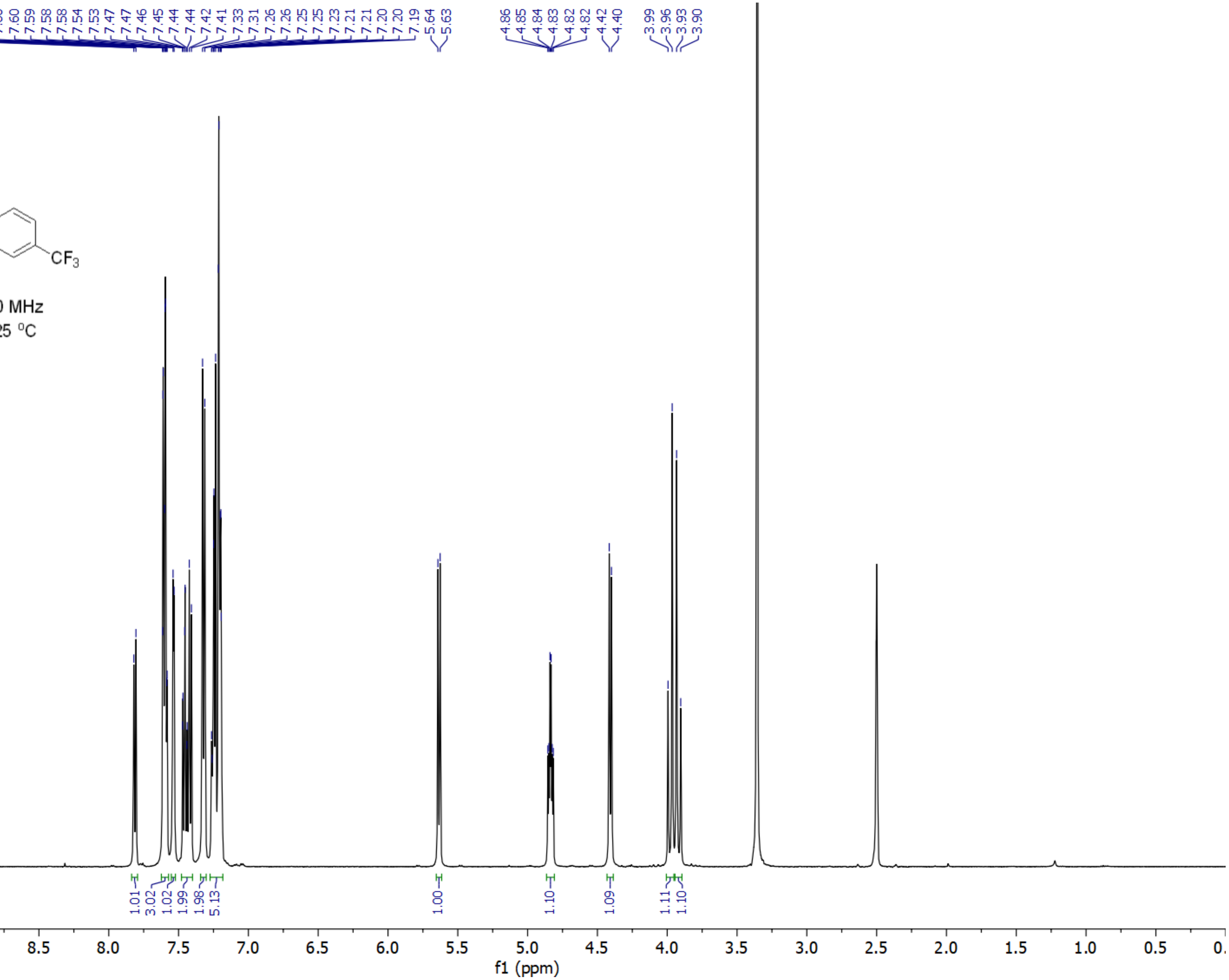

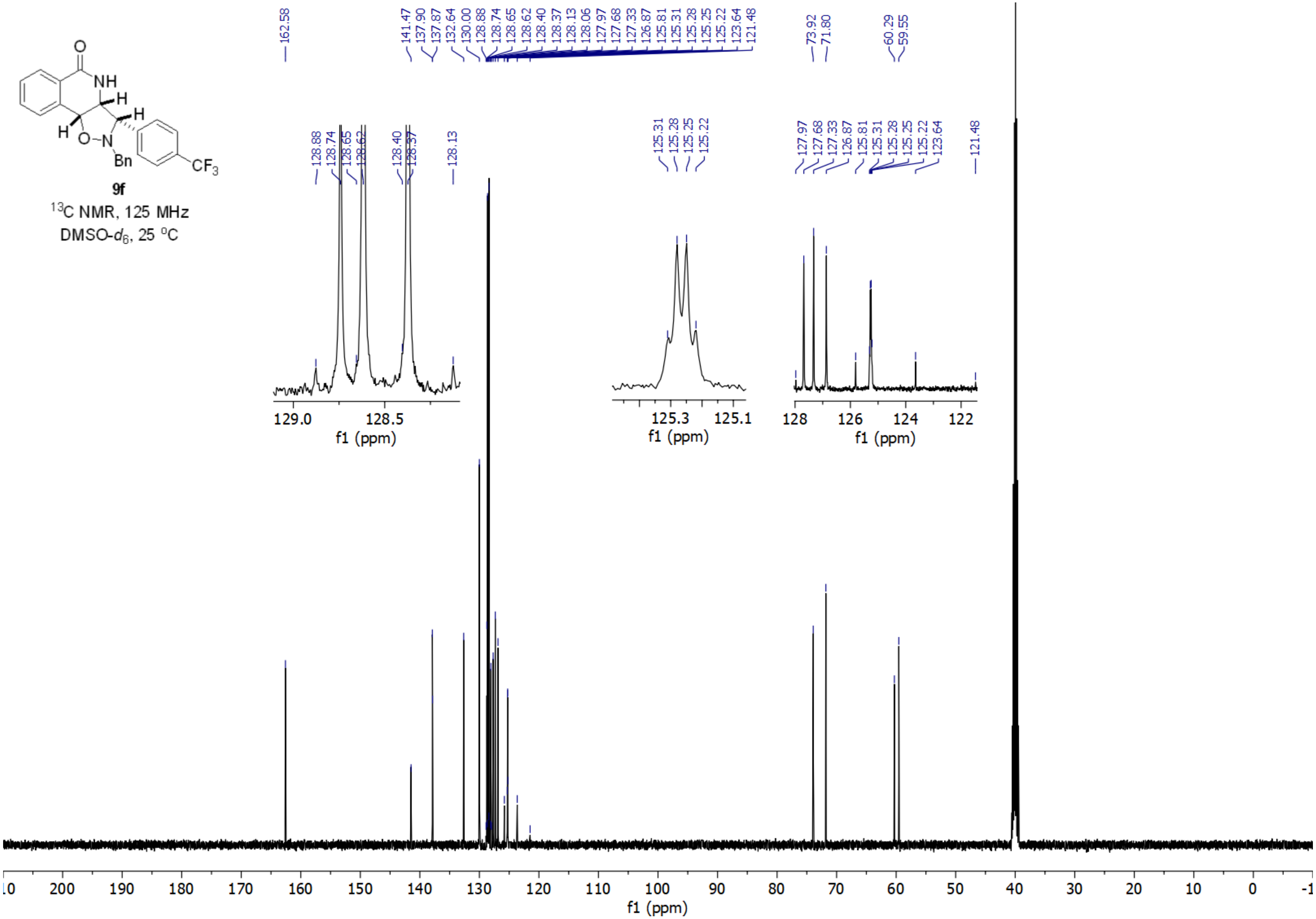
9f
13C NMR, 125 MHz
DMSO-d6, 25 °C
f1 (ppm)

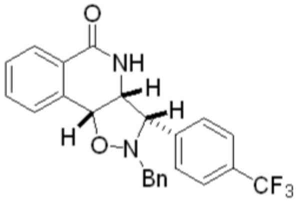

9f

^{19}F NMR, 470 MHz

DMSO-d_6, 25 ^{o}C

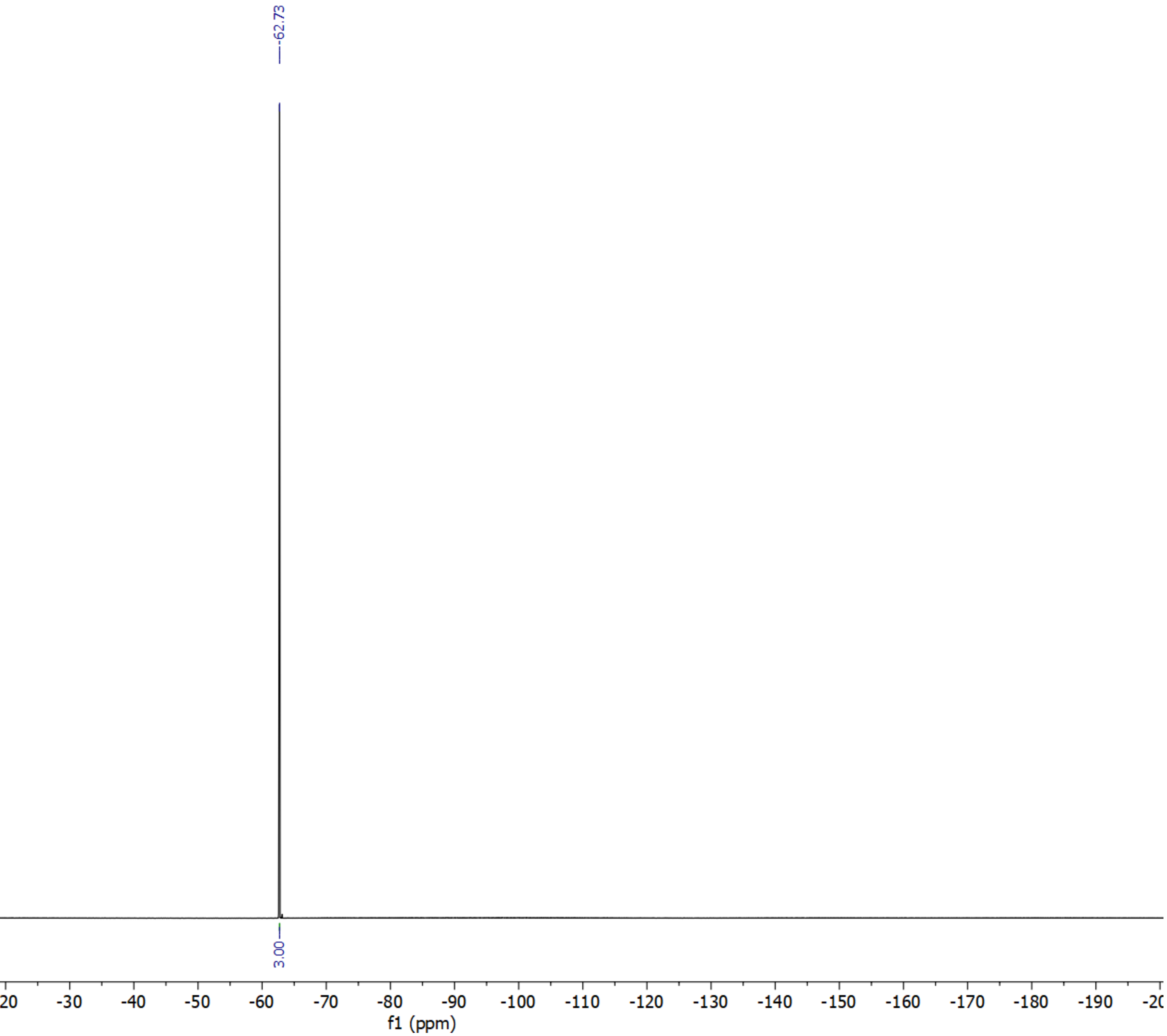

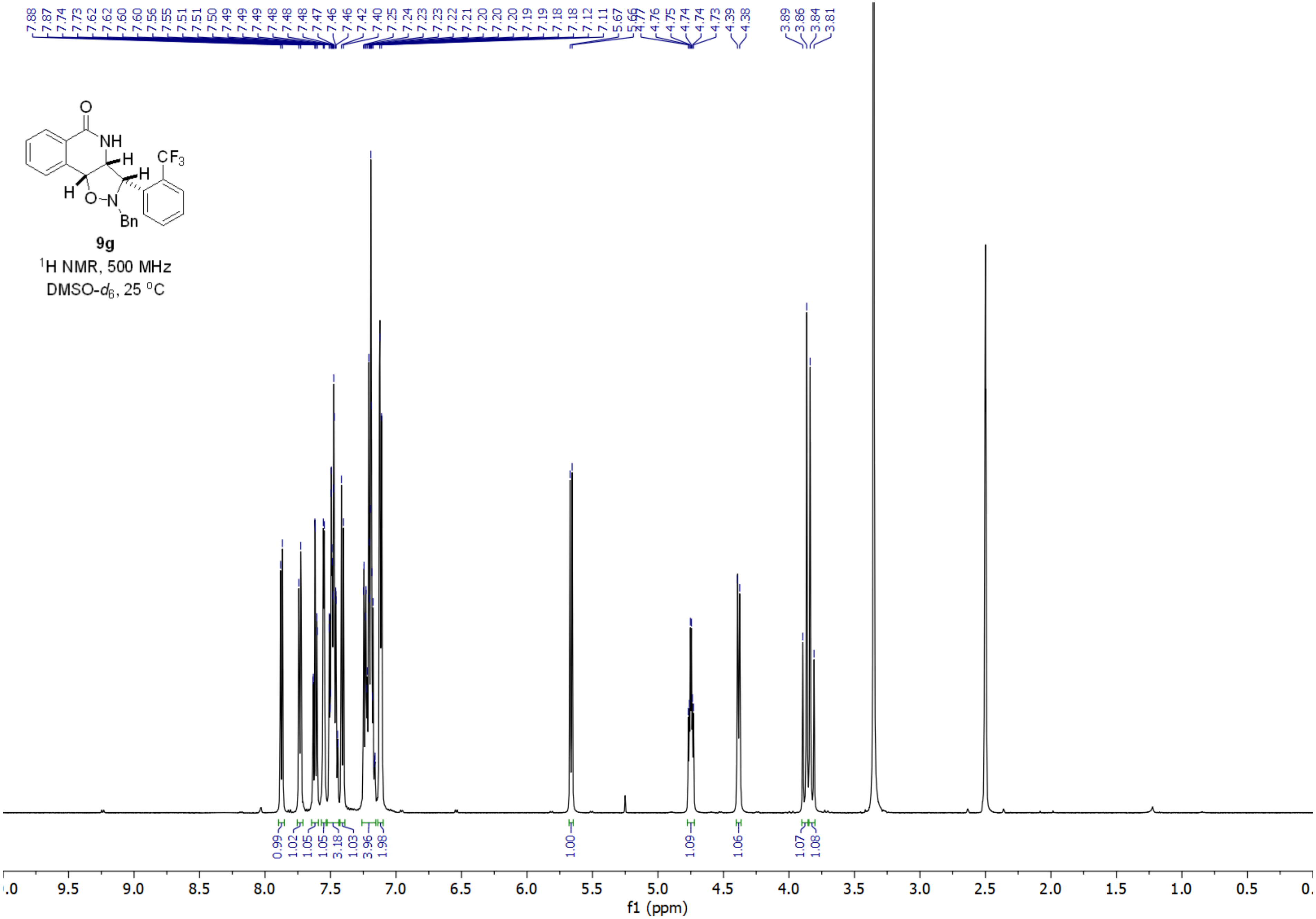
7.88 7.87 7.74 7.73 7.62 7.60 7.60 7.56 7.55 7.51 7.50 7.49 7.49 7.48 7.48 7.48 7.47 7.46 7.46 7.42 7.40 7.25 7.24 7.23 7.23 7.22 7.21 7.20 7.20 7.20 7.19 7.18 7.18 7.12 7.11 5.67 5.66 4.77 4.76 4.75 4.74 4.74 4.73 4.39 4.38 3.89 3.86 3.84 3.81
O
NH
H
CF3
H
H
O
N
Bn
9g
1H NMR, 500 MHz
DMSO-d6, 25 °C
0.99 1.02 1.05 1.05 3.18 1.03 3.96 1.98 1.00 1.09 1.06 1.07 1.08
9.5 9.0 8.5 8.0 7.5 7.0 6.5 6.0 5.5 5.0 4.5 4.0 3.5 3.0 2.5 2.0 1.5 1.0 0.5
f1 (ppm)

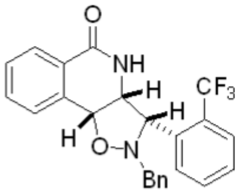

9g

^{13}C NMR, 125 MHz

DMSO-d_6, 25 °C

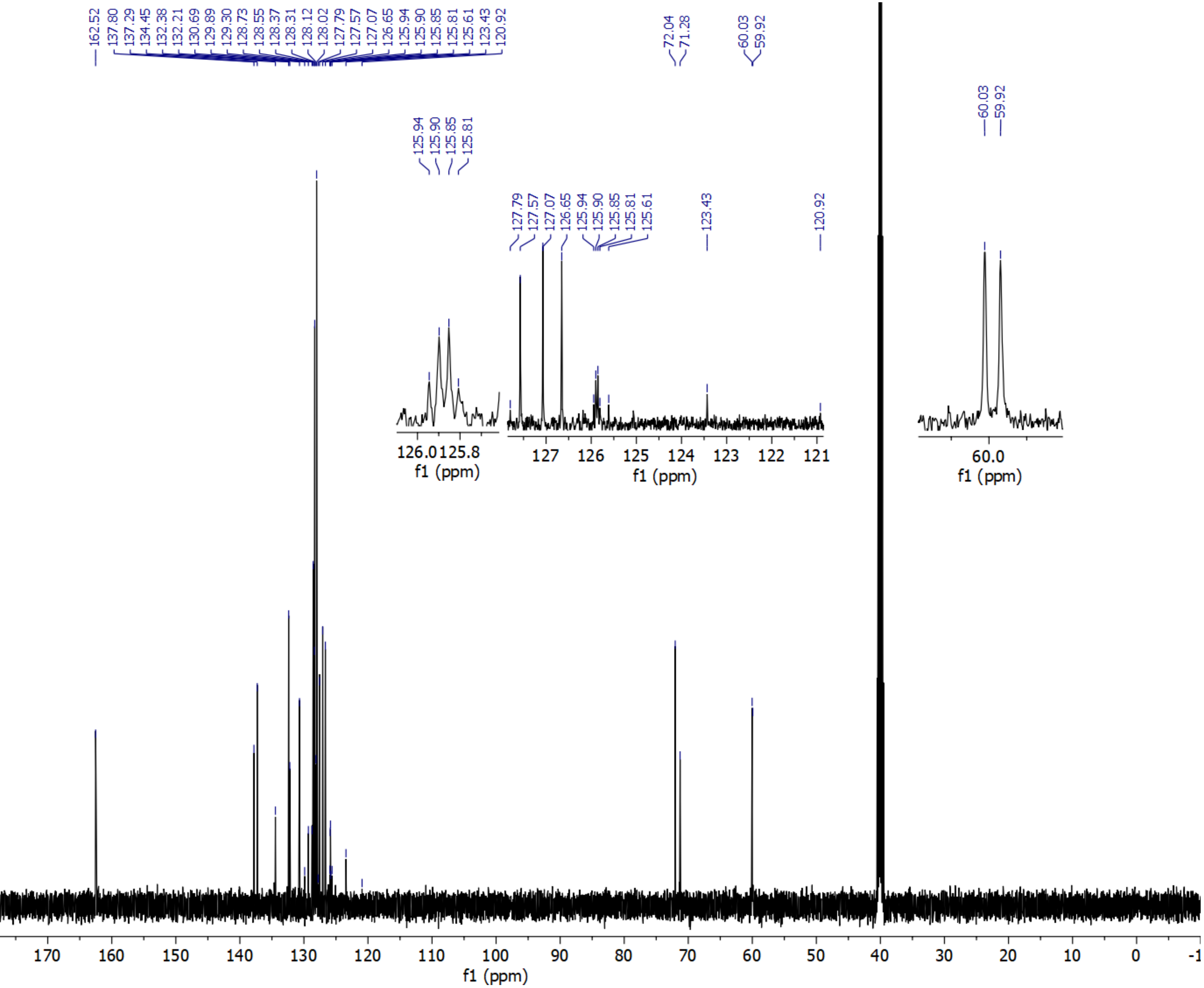

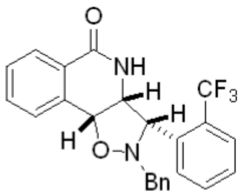

9g

^{19}F NMR, 470 MHz

DMSO-d_6, 25 ^{o}C

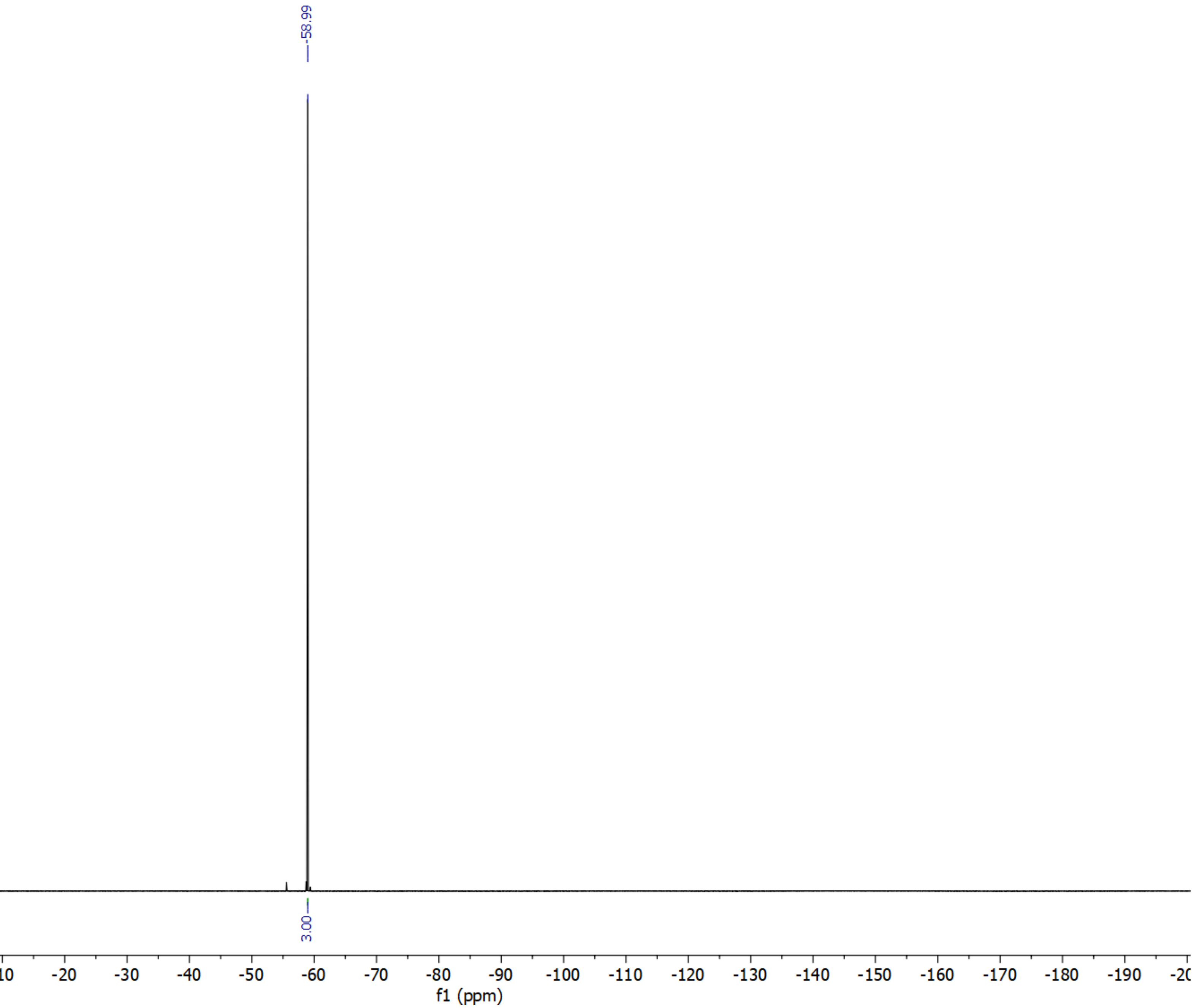

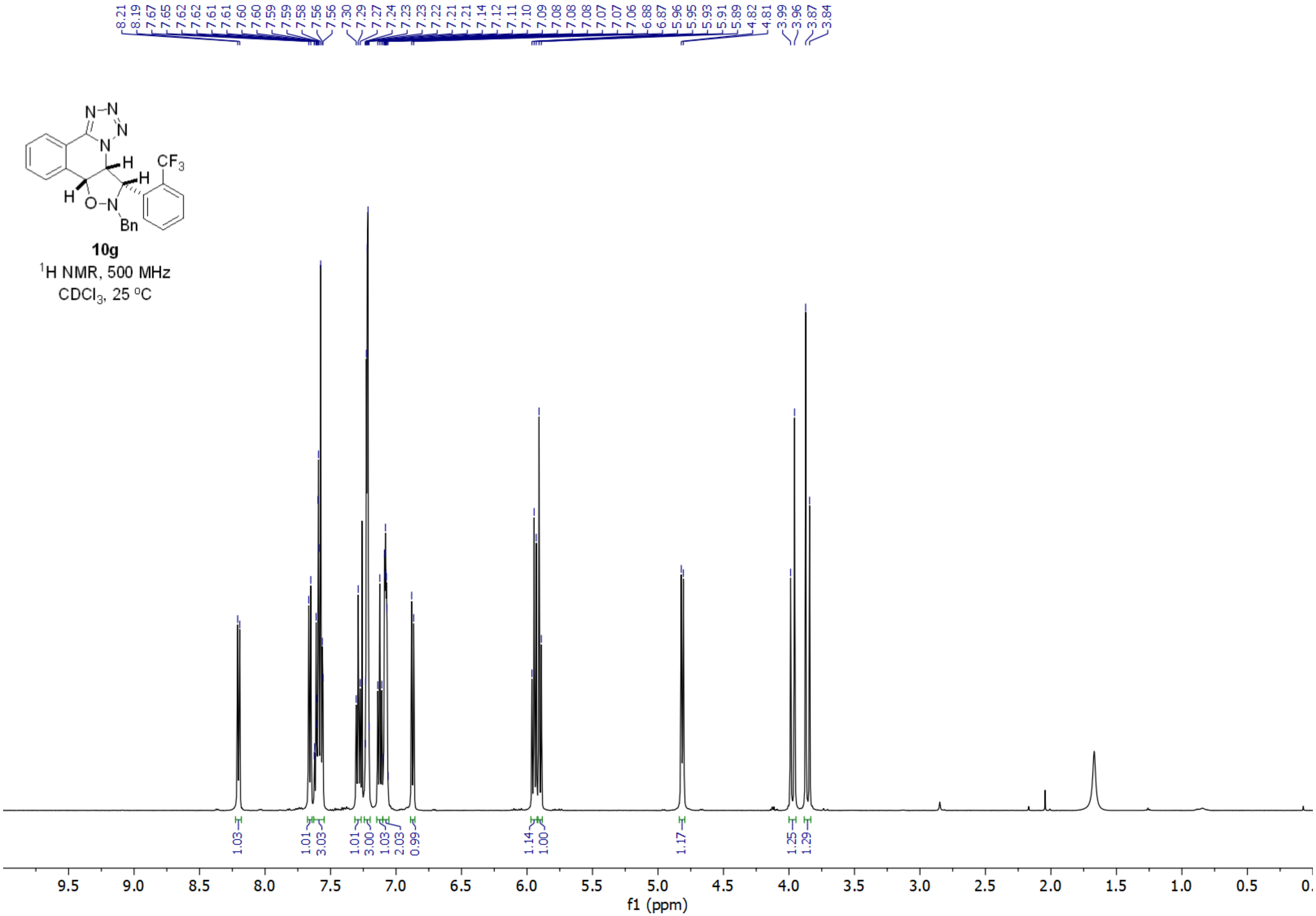

10g
¹H NMR, 500 MHz
CDCl3, 25 °C
8.21 8.19 7.67 7.65 7.62 7.61 7.61 7.60 7.60 7.59 7.59 7.58 7.56 7.56 7.30 7.29 7.27 7.24 7.23 7.23 7.22 7.21 7.21 7.14 7.12 7.11 7.10 7.09 7.08 7.08 7.08 7.07 7.07 7.06 6.88 6.87 5.96 5.95 5.93 5.91 5.89 4.82 4.81 3.99 3.96 3.87 3.84
1.03 1.01 3.03 1.01 3.00 1.03 2.03 0.99 1.14 1.00 1.17 1.25 1.29
9.5 9.0 8.5 8.0 7.5 7.0 6.5 6.0 5.5 5.0 4.5 4.0 3.5 3.0 2.5 2.0 1.5 1.0 0.5 0.
f1 (ppm)

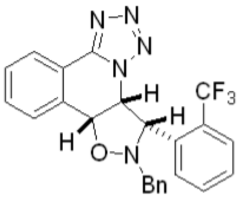

10g

^{13}C NMR, 125 MHz

$CDCl_3$, 25 °C

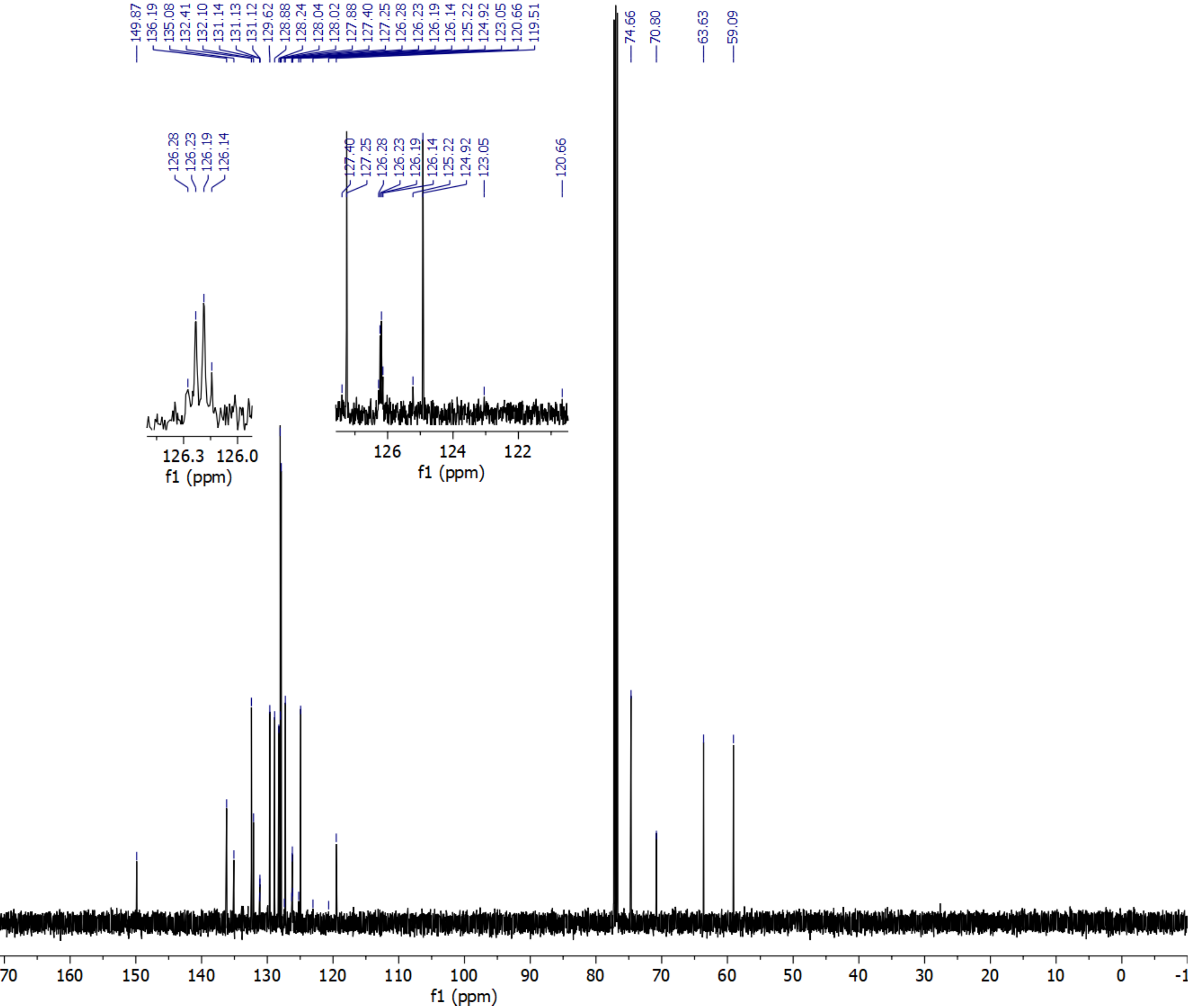

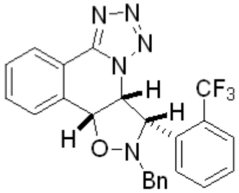

10g

^{19}F NMR, 470 MHz

$CDCl_3$, 25 °C

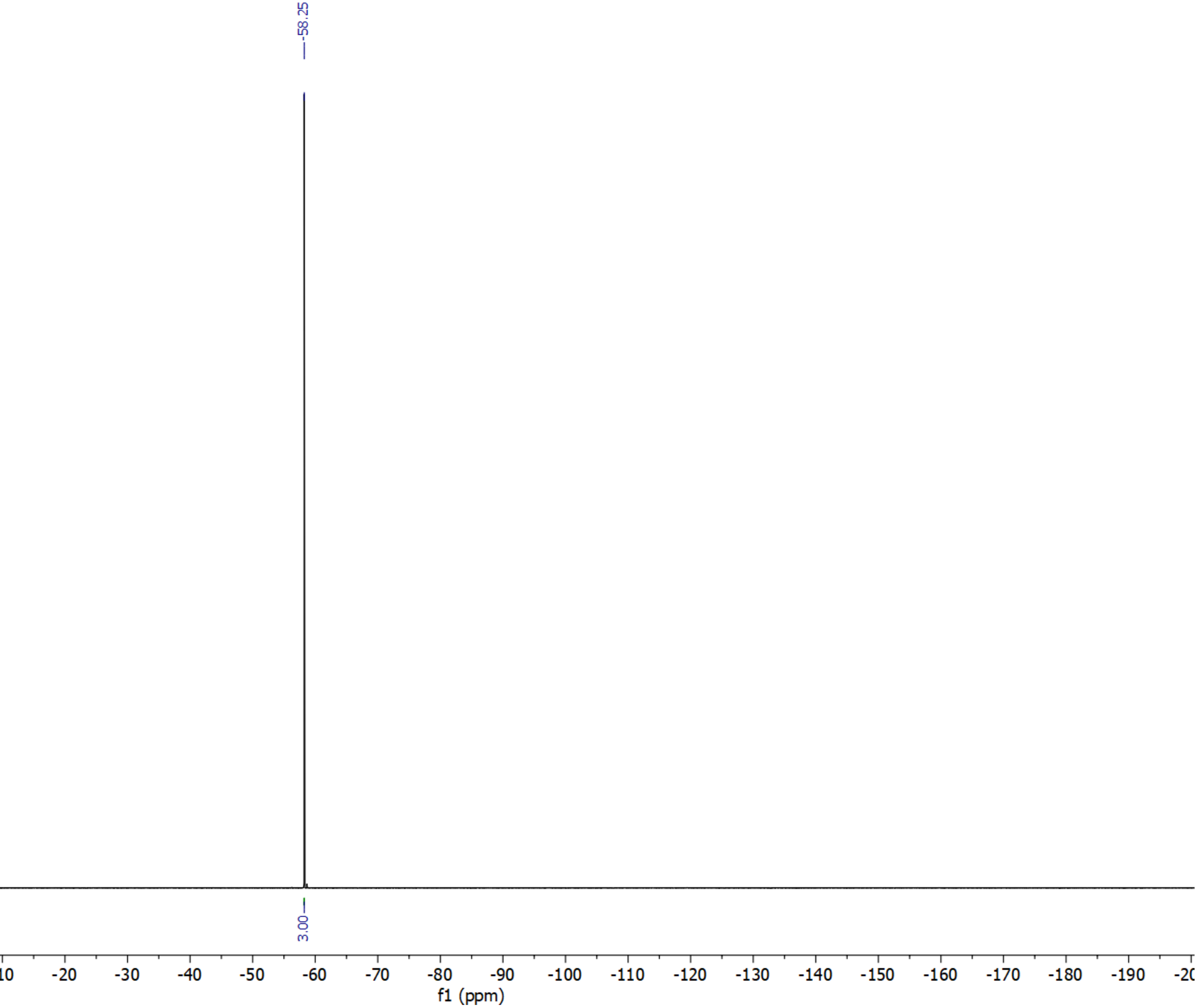

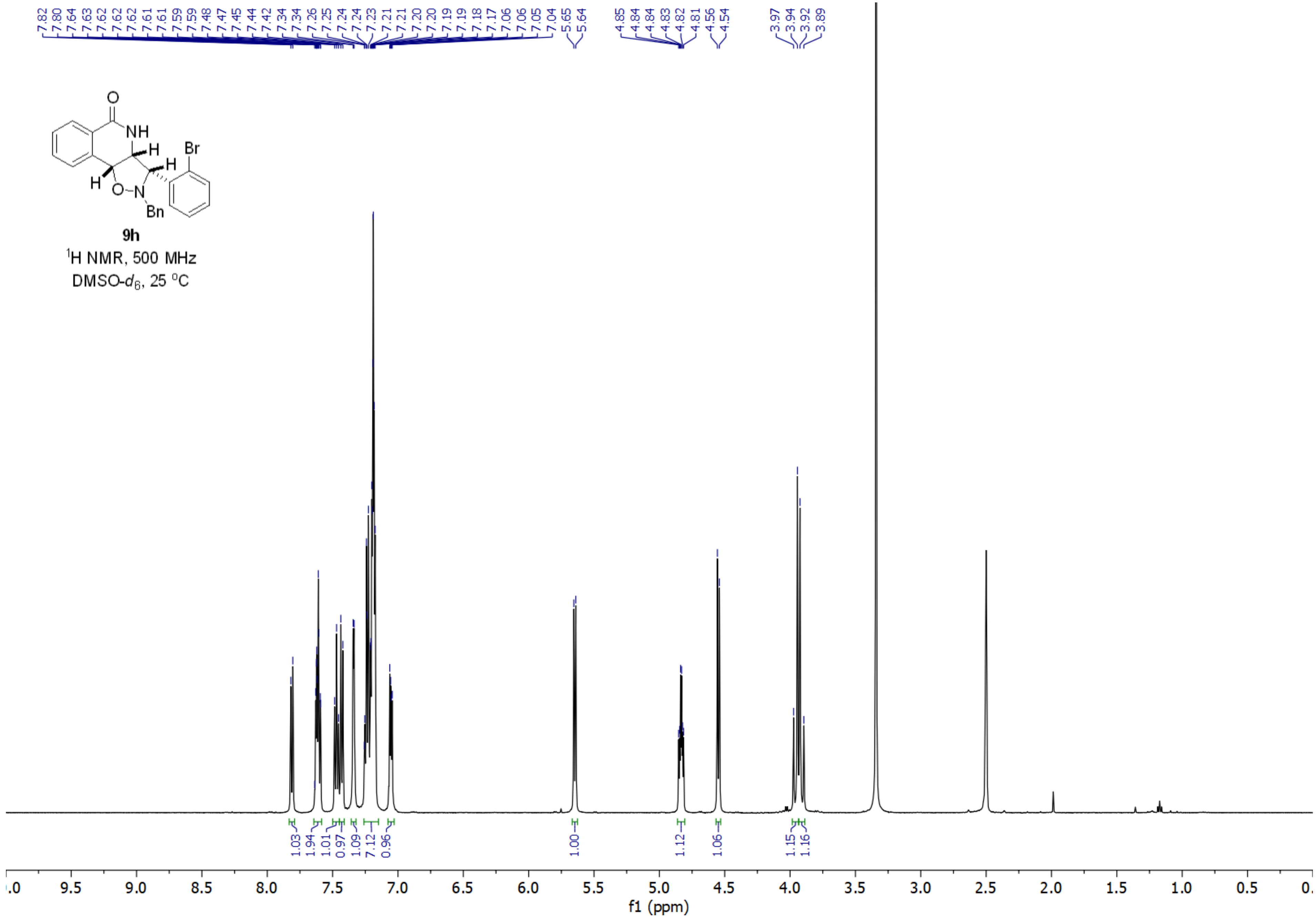
7.82
7.80
7.64
7.63
7.62
7.62
7.62
7.61
7.61
7.59
7.59
7.48
7.47
7.45
7.44
7.42
7.34
7.34
7.26
7.25
7.24
7.24
7.23
7.21
7.21
7.20
7.20
7.19
7.19
7.18
7.17
7.06
7.06
7.05
7.04
5.65
5.64
4.85
4.84
4.84
4.83
4.82
4.81
4.56
4.54
3.97
3.94
3.92
3.89
O
NH
H
Br
H
H
O
N
Bn
9h
^{1}H NMR, 500 MHz
DMSO-d_6, 25 ^{o}C
1.03
1.94
1.01
0.97
1.09
7.12
0.96
1.00
1.12
1.06
1.15
1.16
9.5
9.0
8.5
8.0
7.5
7.0
6.5
6.0
5.5
5.0
4.5
4.0
3.5
3.0
2.5
2.0
1.5
1.0
0.5
f1 (ppm)

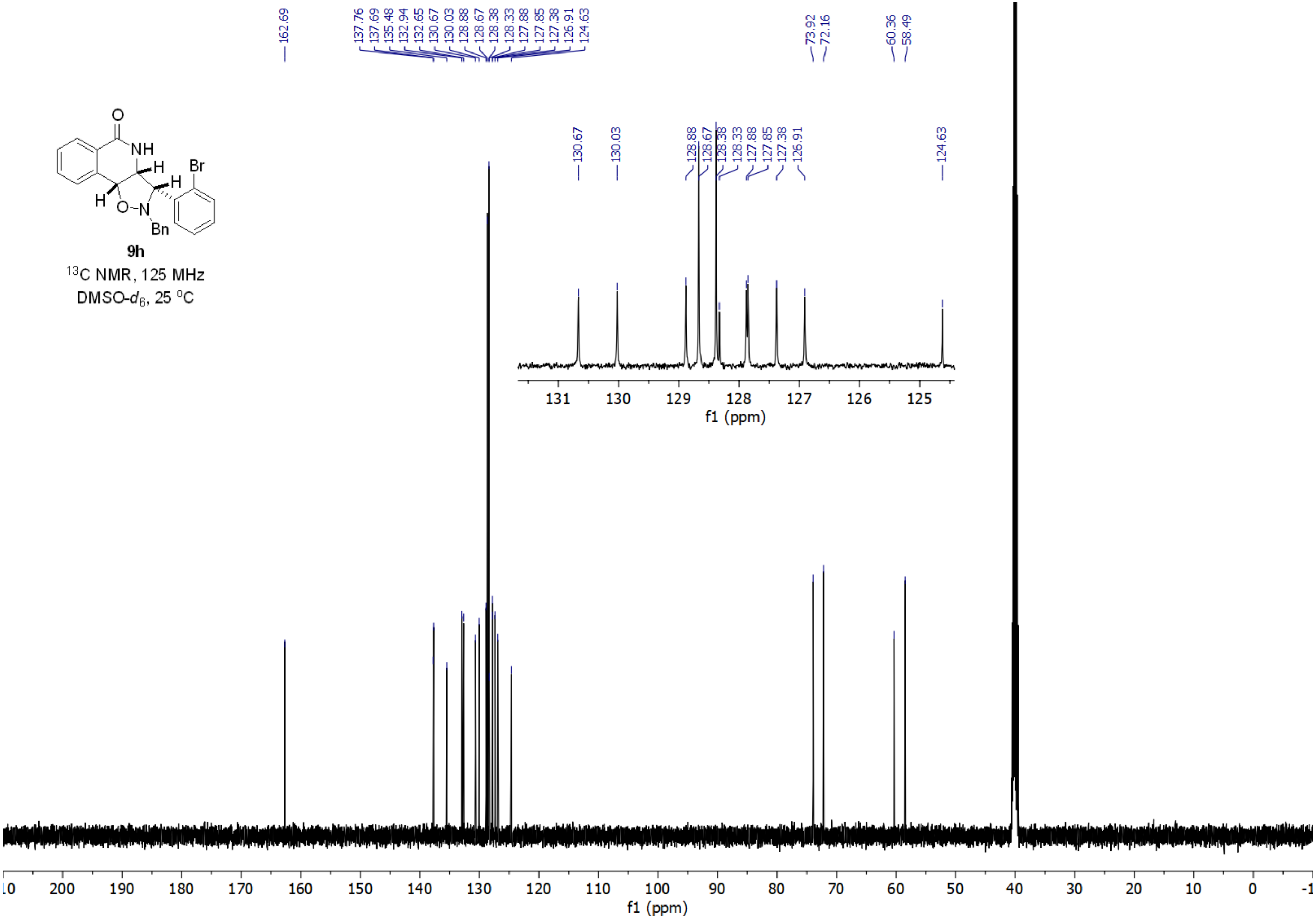

9h

^{13}C NMR, 125 MHz

DMSO-d_6, 25 °C

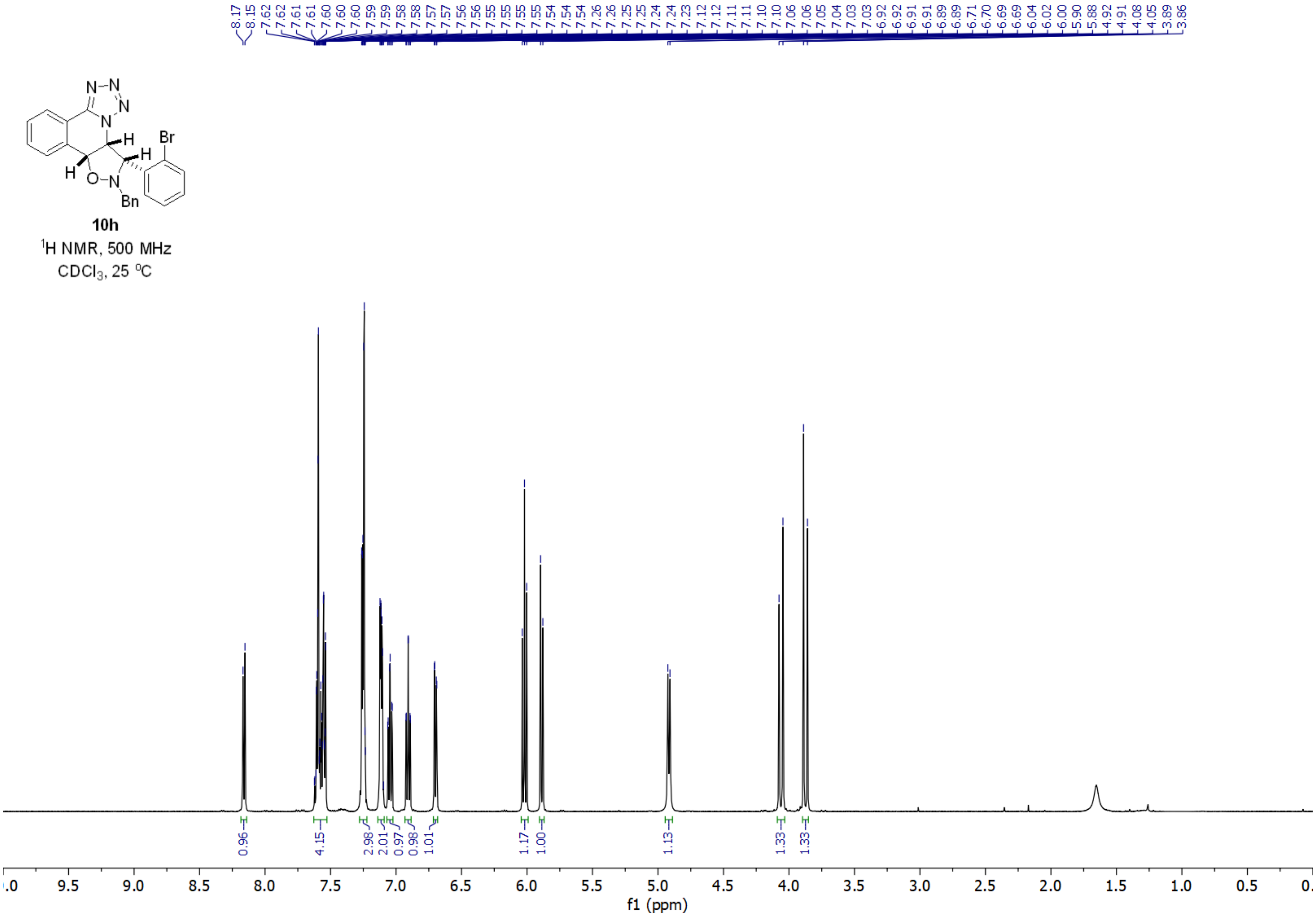
10h
^{1}H NMR, 500 MHz
CDCl3, 25 °C
f1 (ppm)

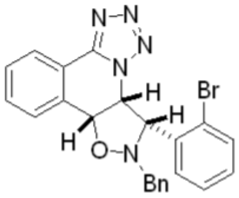

10h

^{13}C NMR, 125 MHz

$CDCl_3$, 25 °C

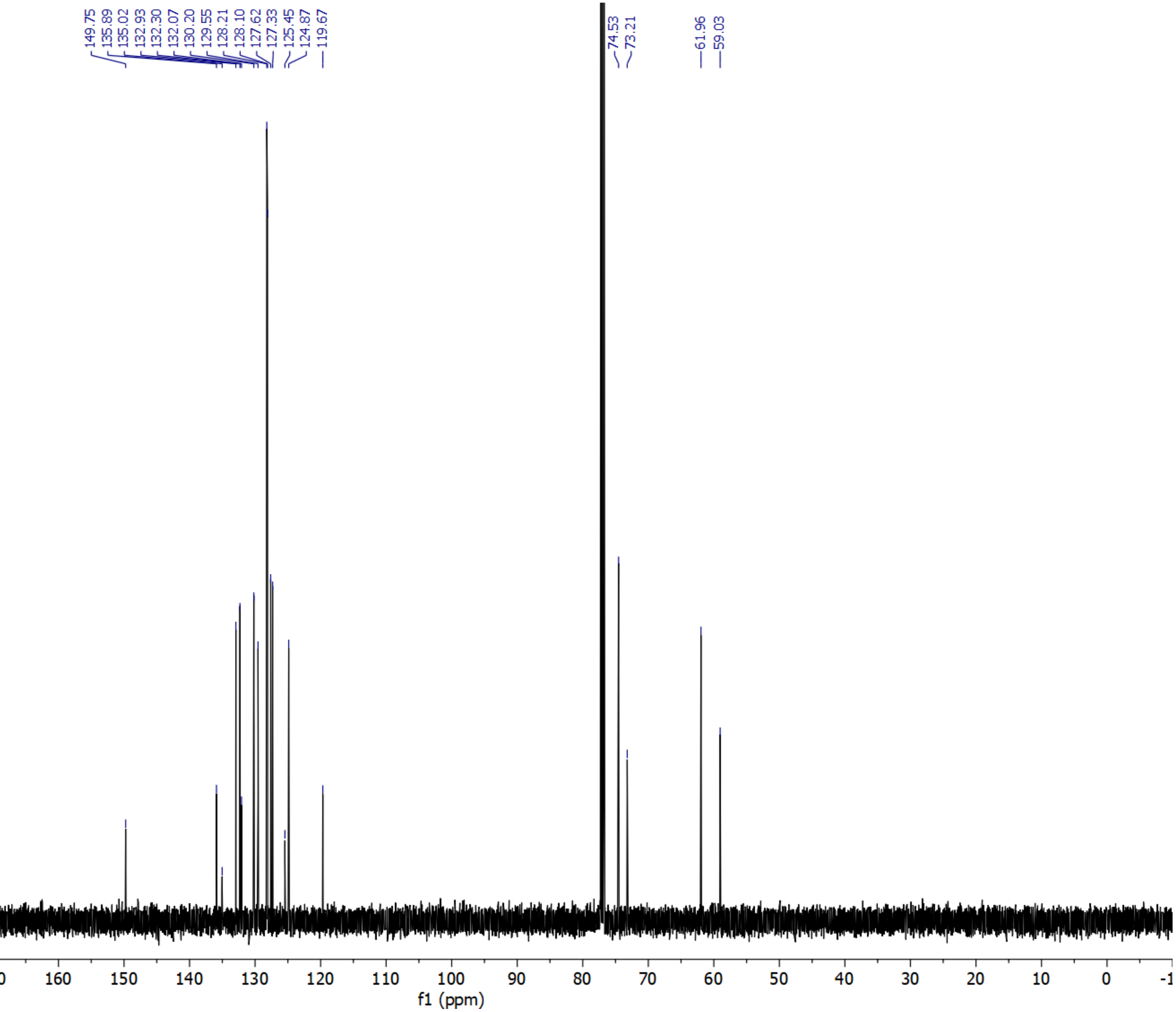

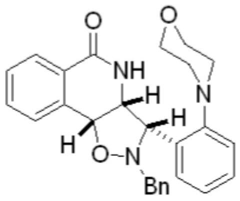

9i

^{1}H NMR, 500 MHz

DMSO-d_6, 25 ^{o}C

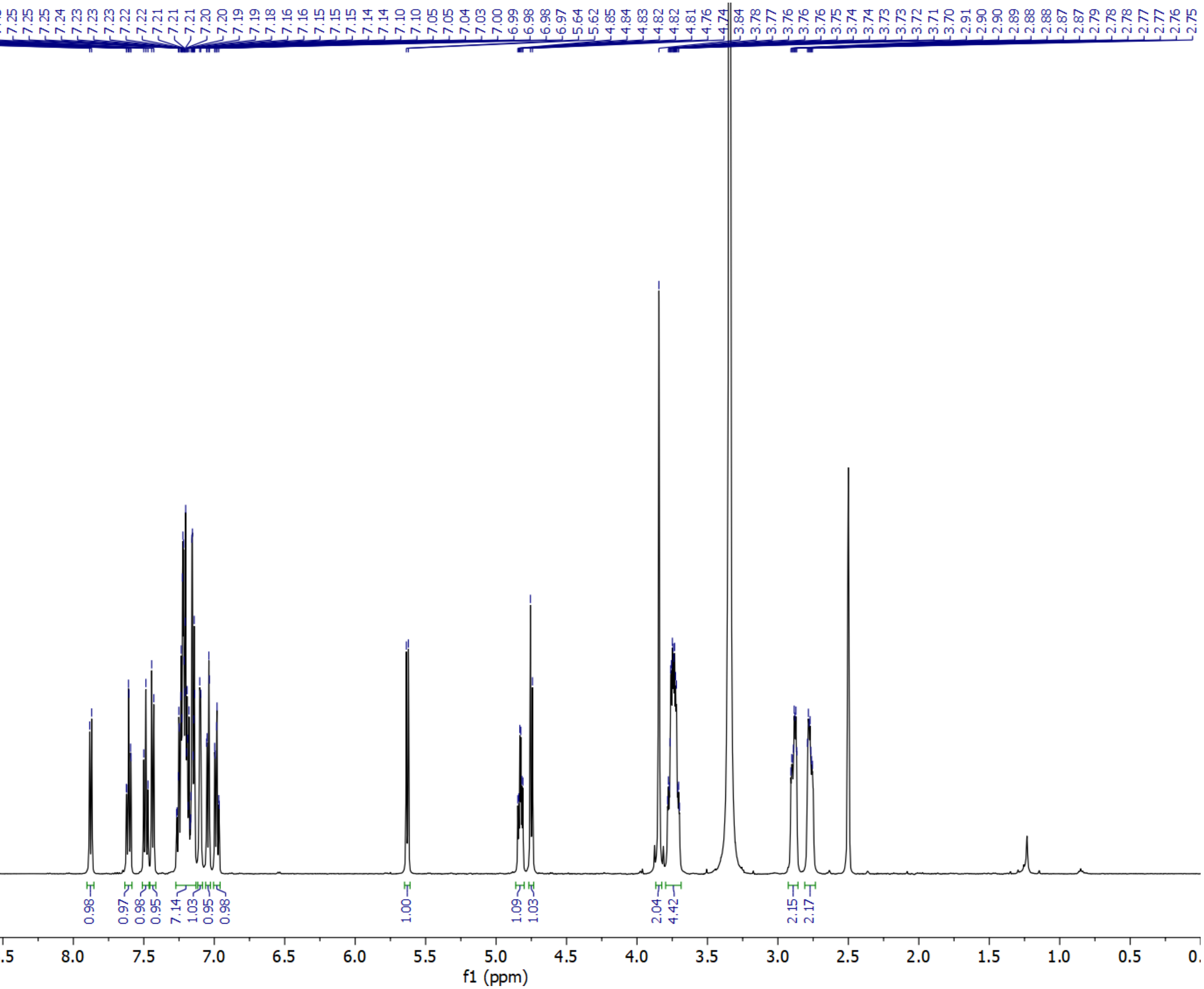

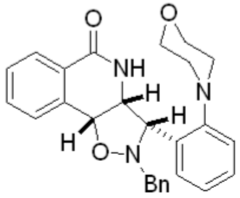

9i

^{1}H NMR, 500 MHz

DMSO-d_6, 25 °C

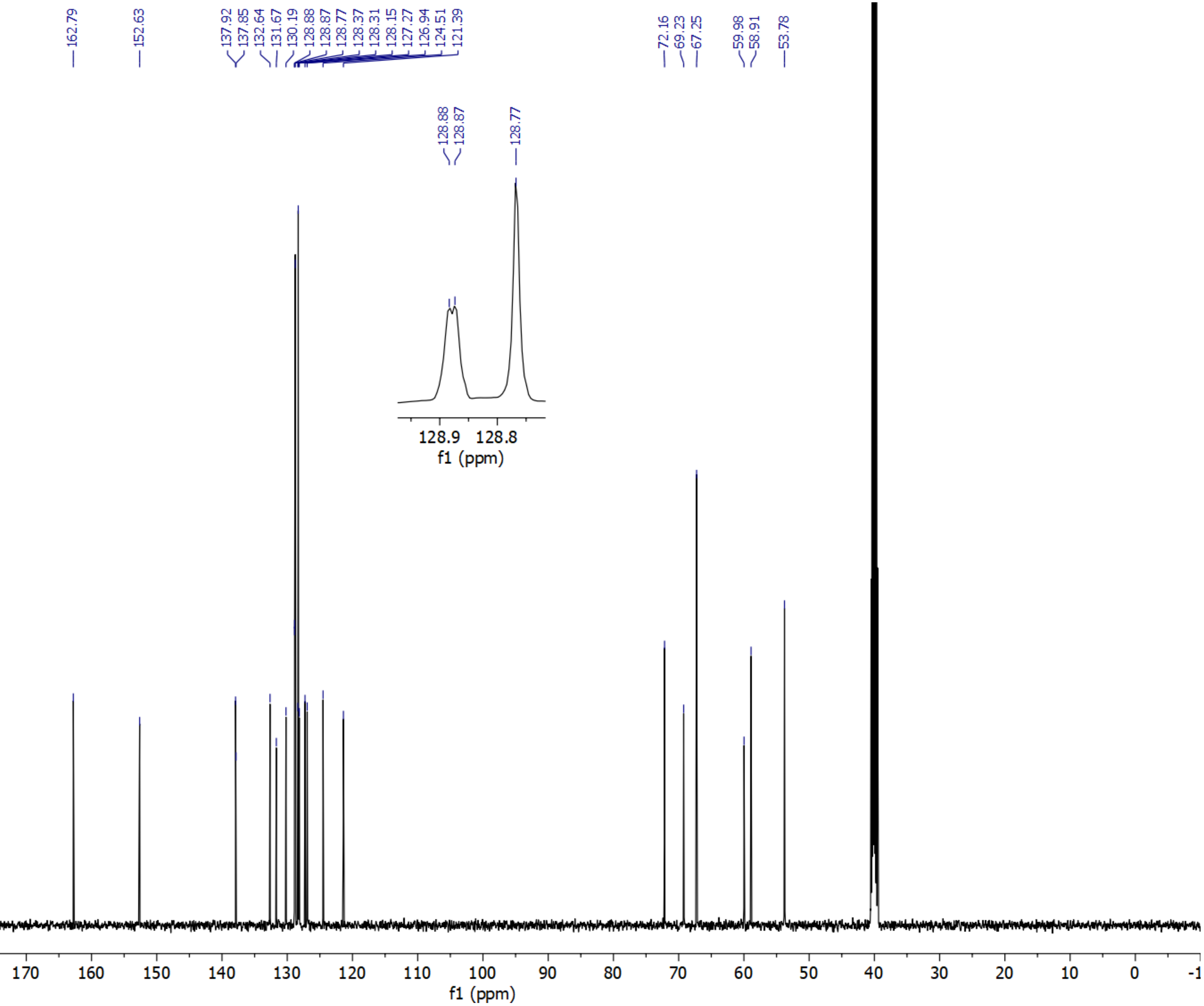

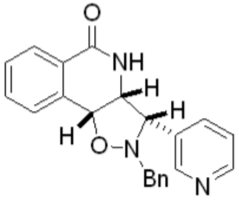

9j

^{1}H NMR, 500 MHz

DMSO-d_6, 25 ^{o}C

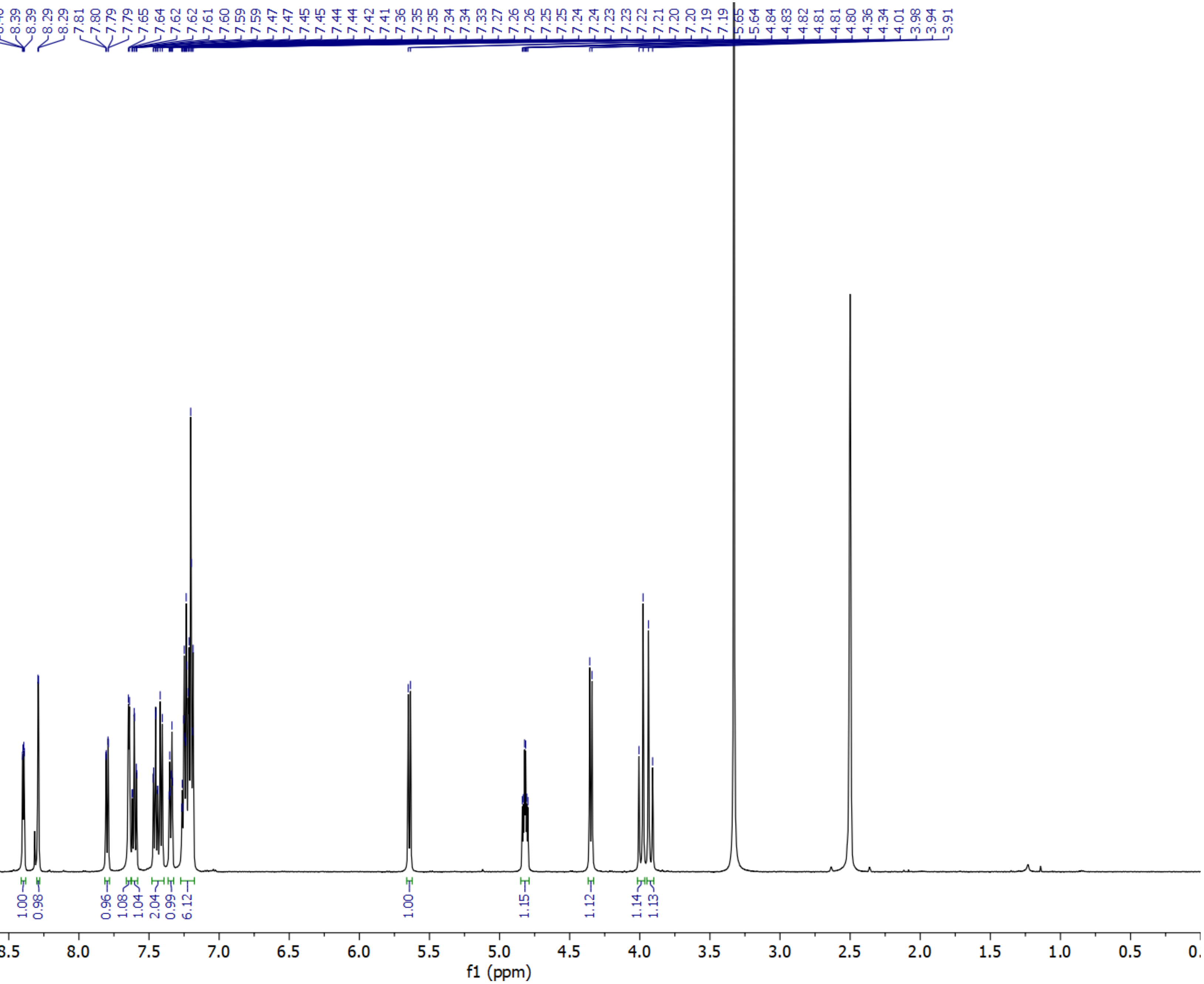

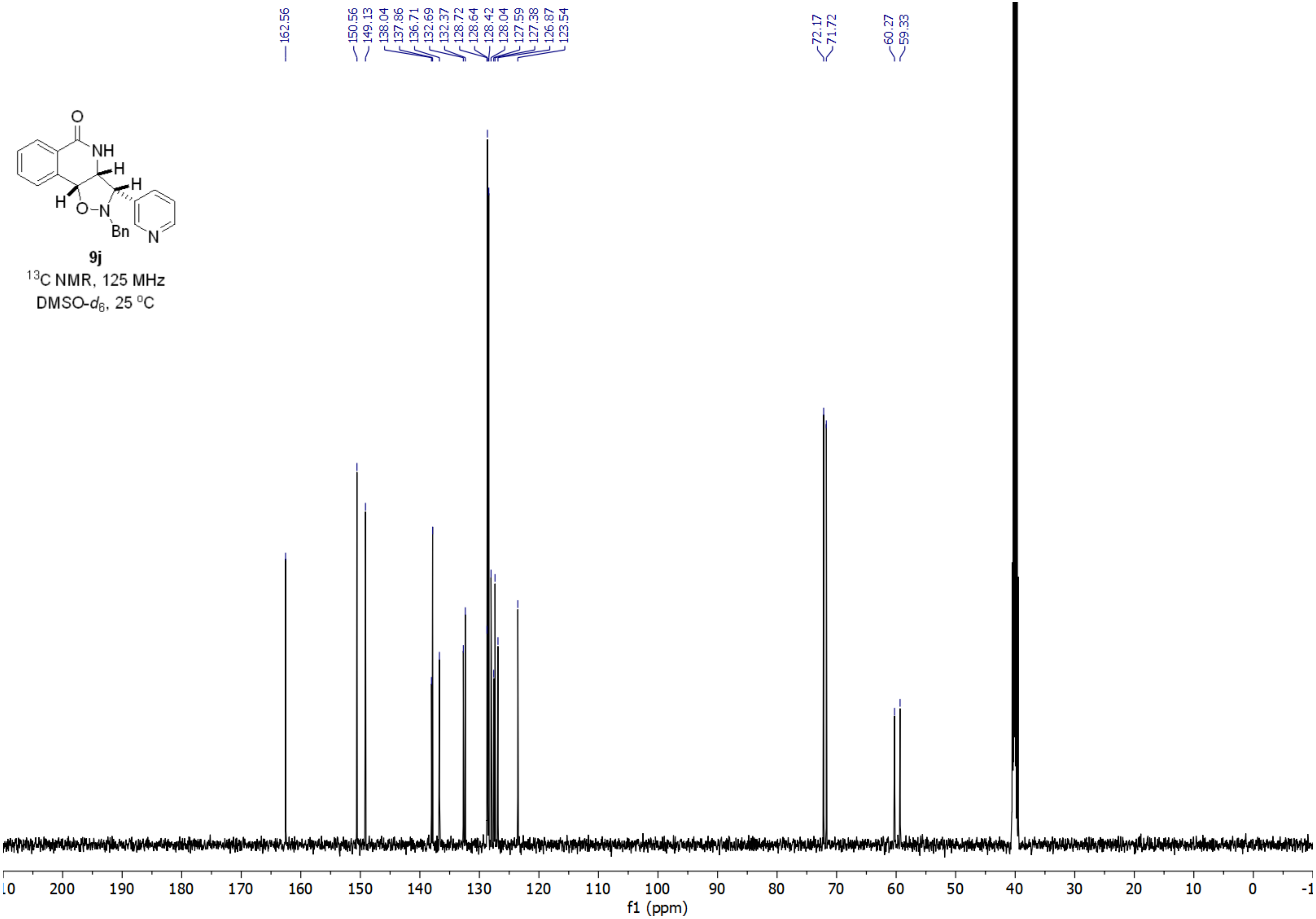

9j

^{13}C NMR, 125 MHz

DMSO-d_6, 25 °C

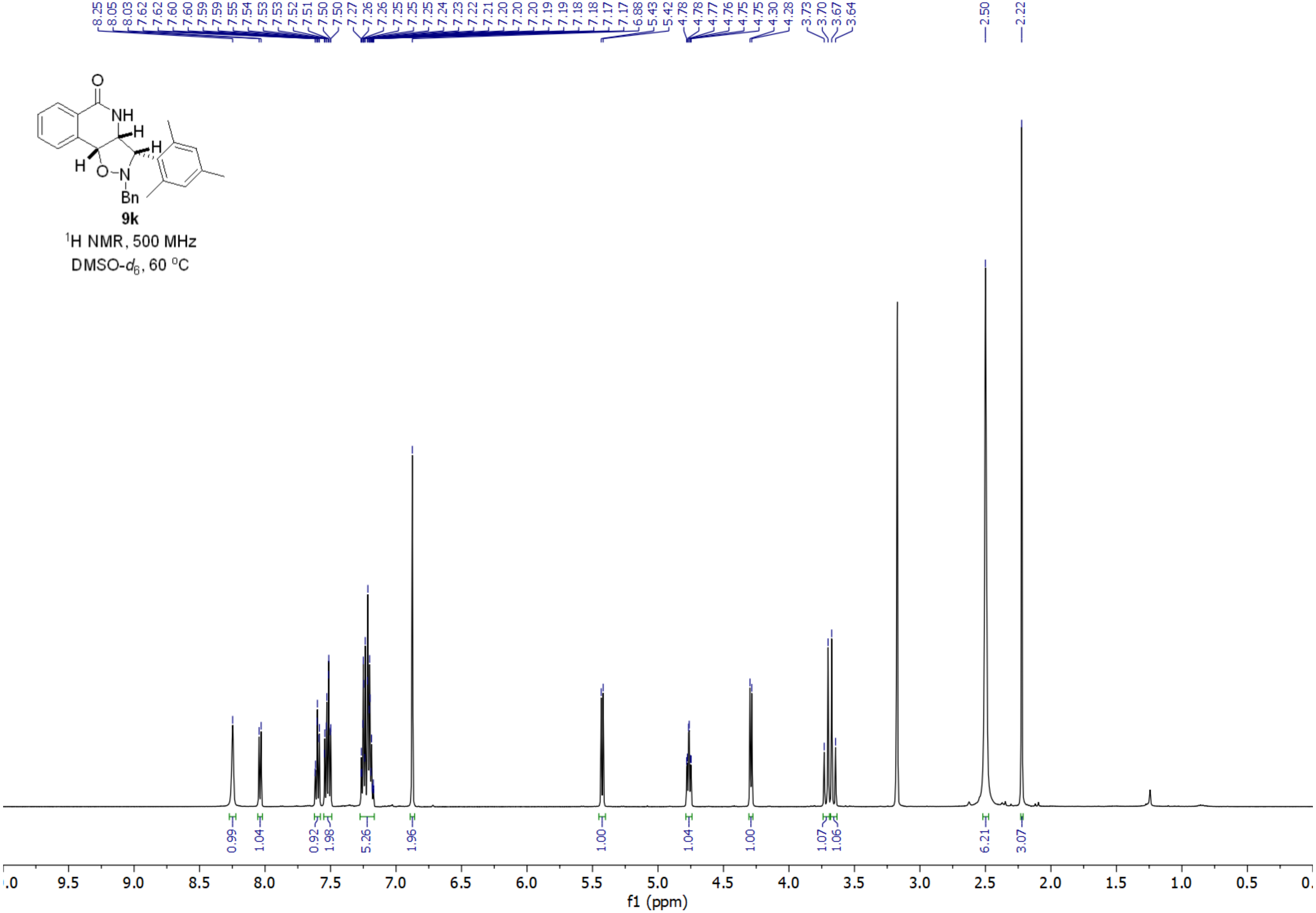

9k

^{1}H NMR, 500 MHz

DMSO-d_6, 60 ^{o}C

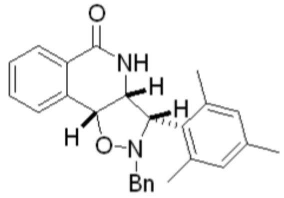

9k

^{13}C NMR, 125 MHz

DMSO-d_6, 60 °C

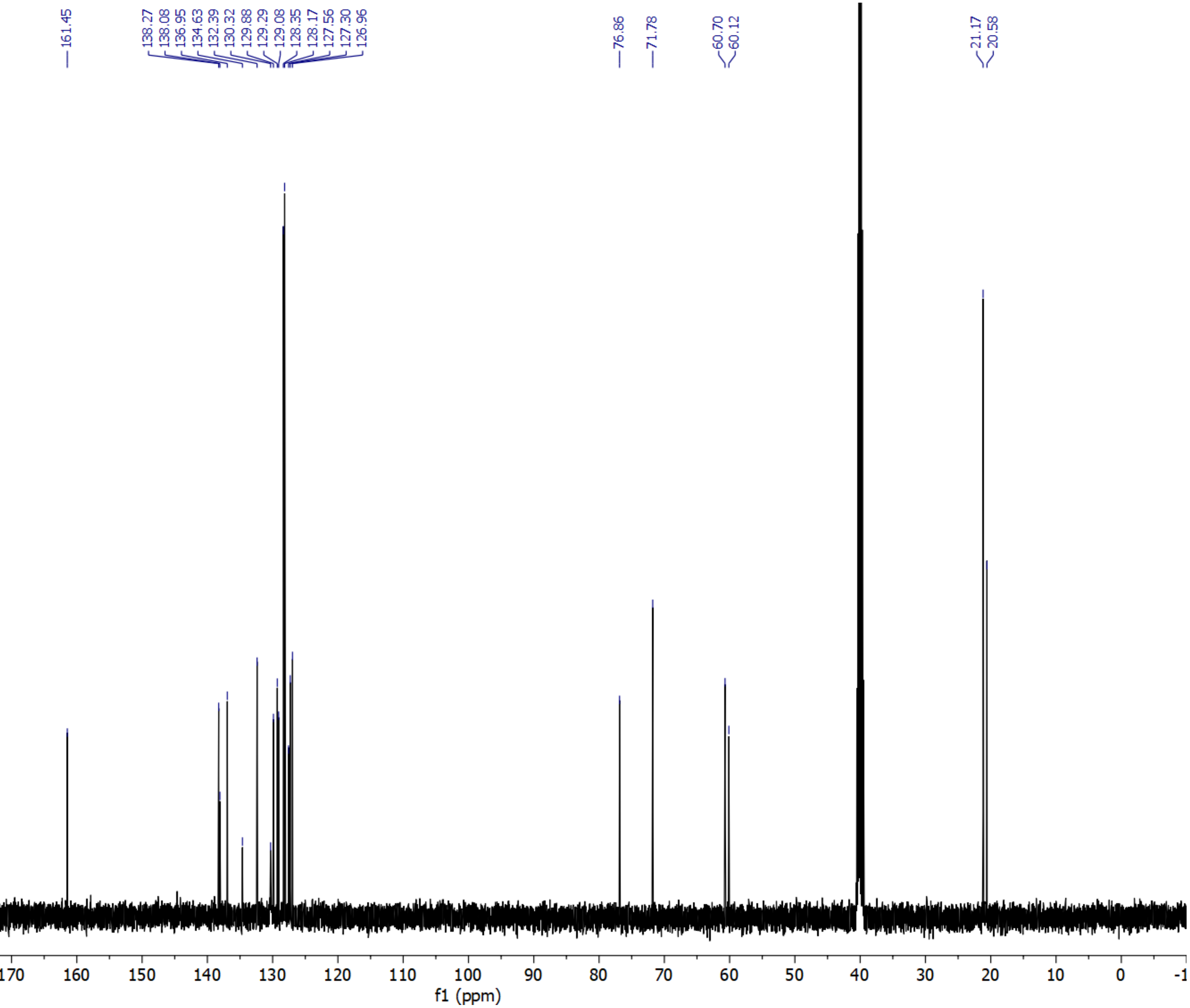

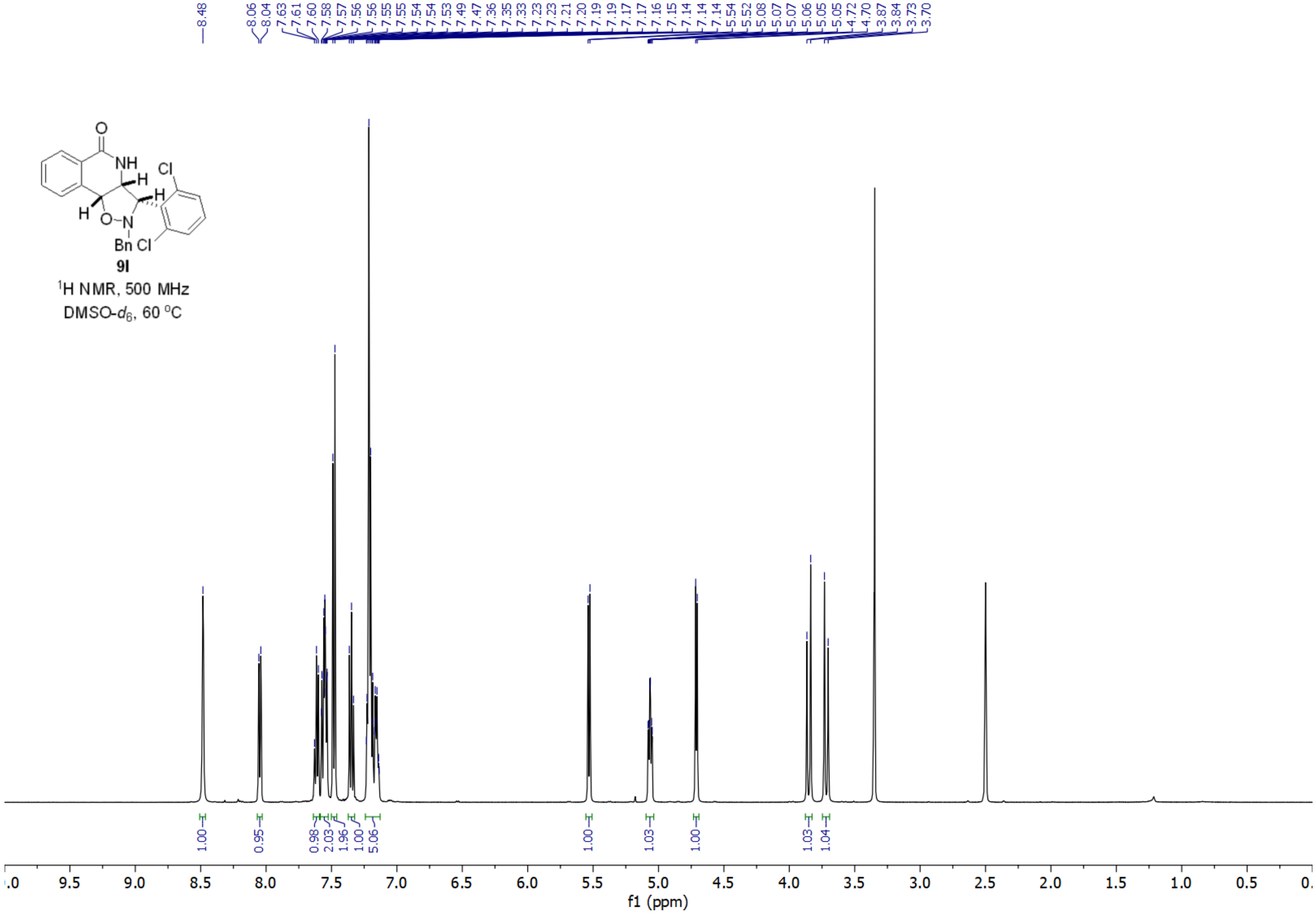

9l

^{1}H NMR, 500 MHz

DMSO-d_6, 60 °C

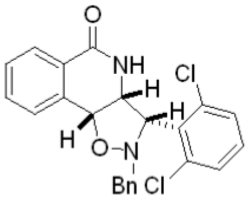

9l

^{13}C NMR, 125 MHz

DMSO-d_6, 60 °C

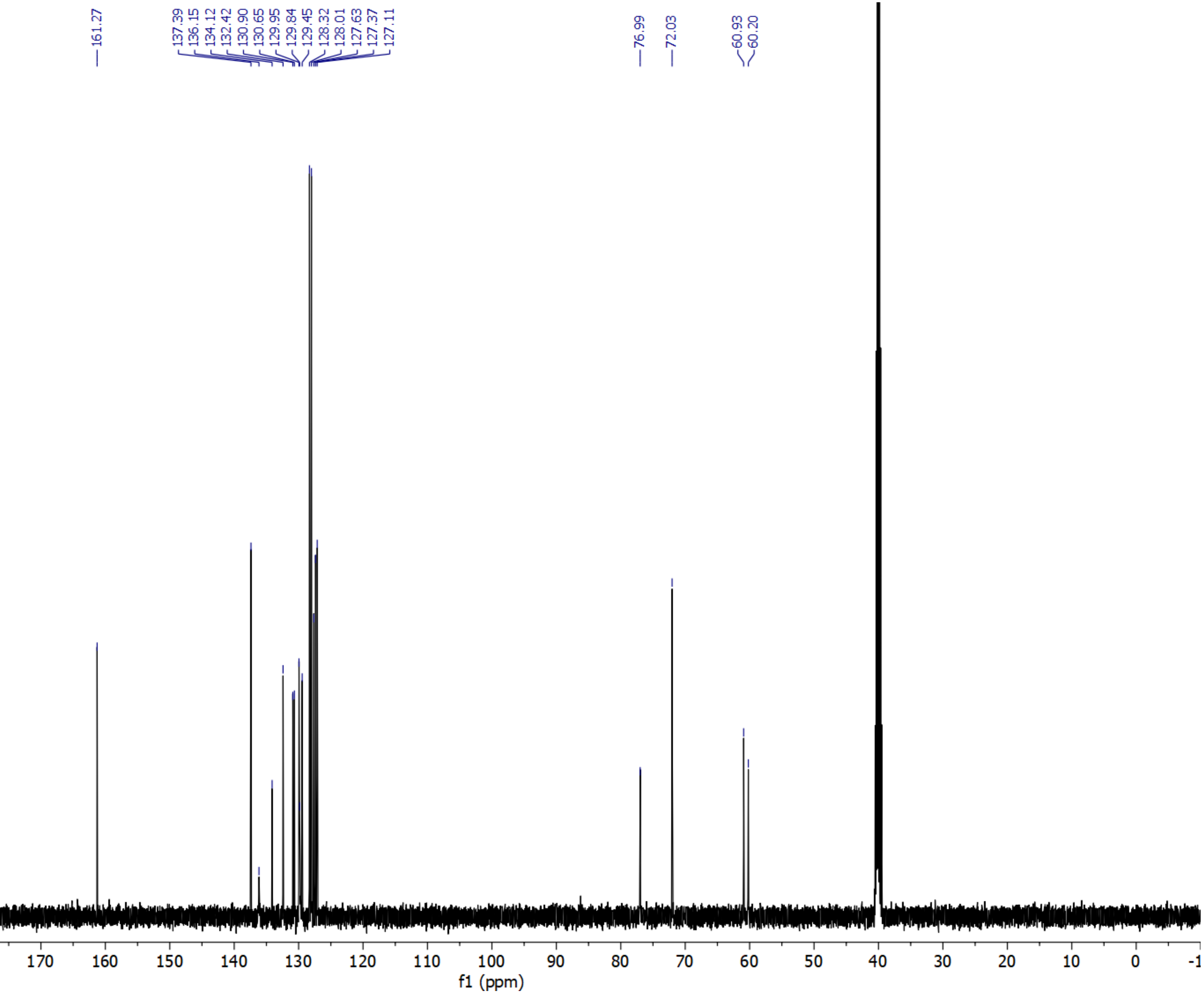

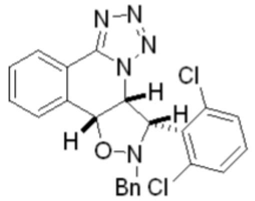

10l

^{1}H NMR, 500 MHz

$CDCl_3$, 25 °C

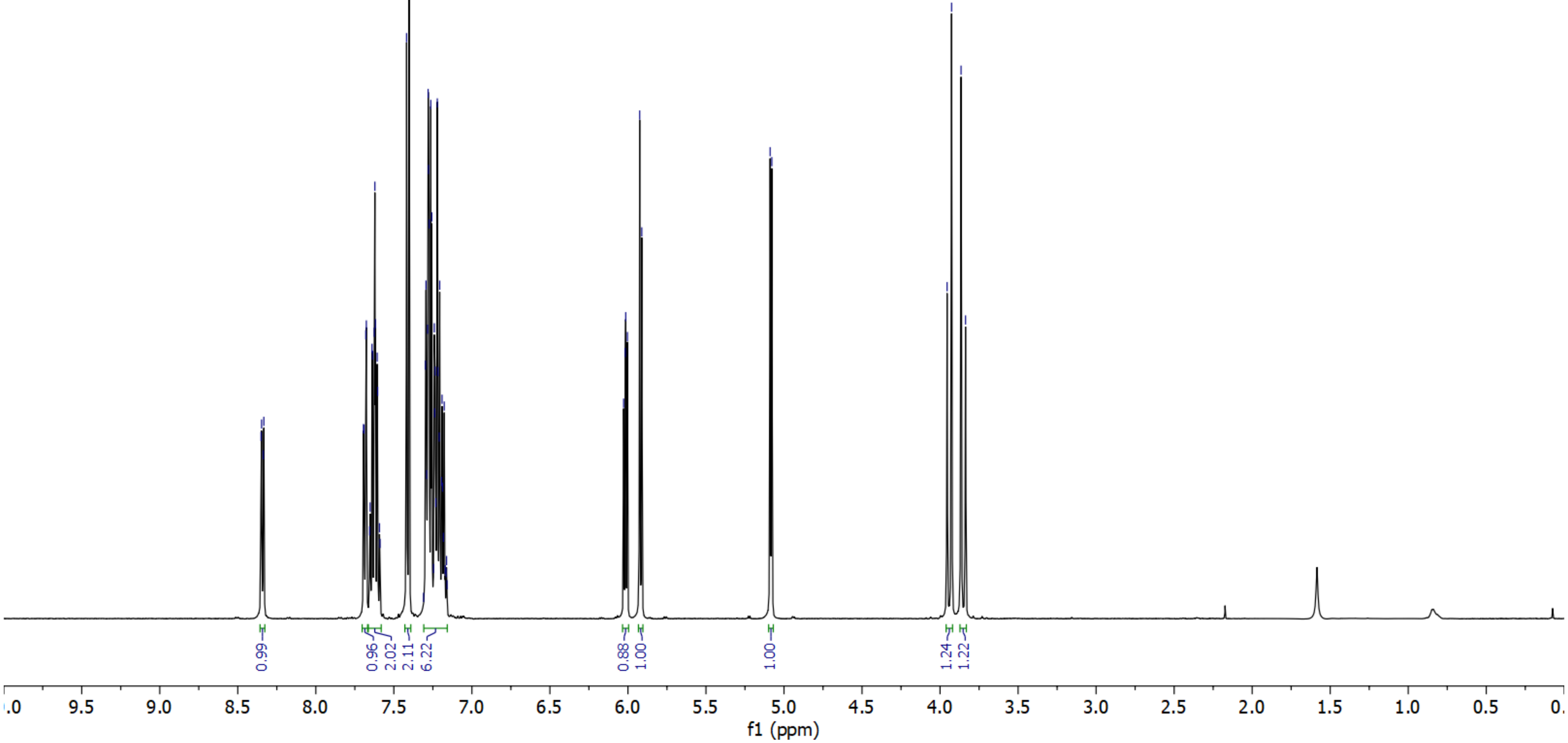

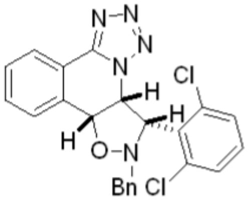

10l

^{13}C NMR, 125 MHz
$CDCl_3$, 25 °C

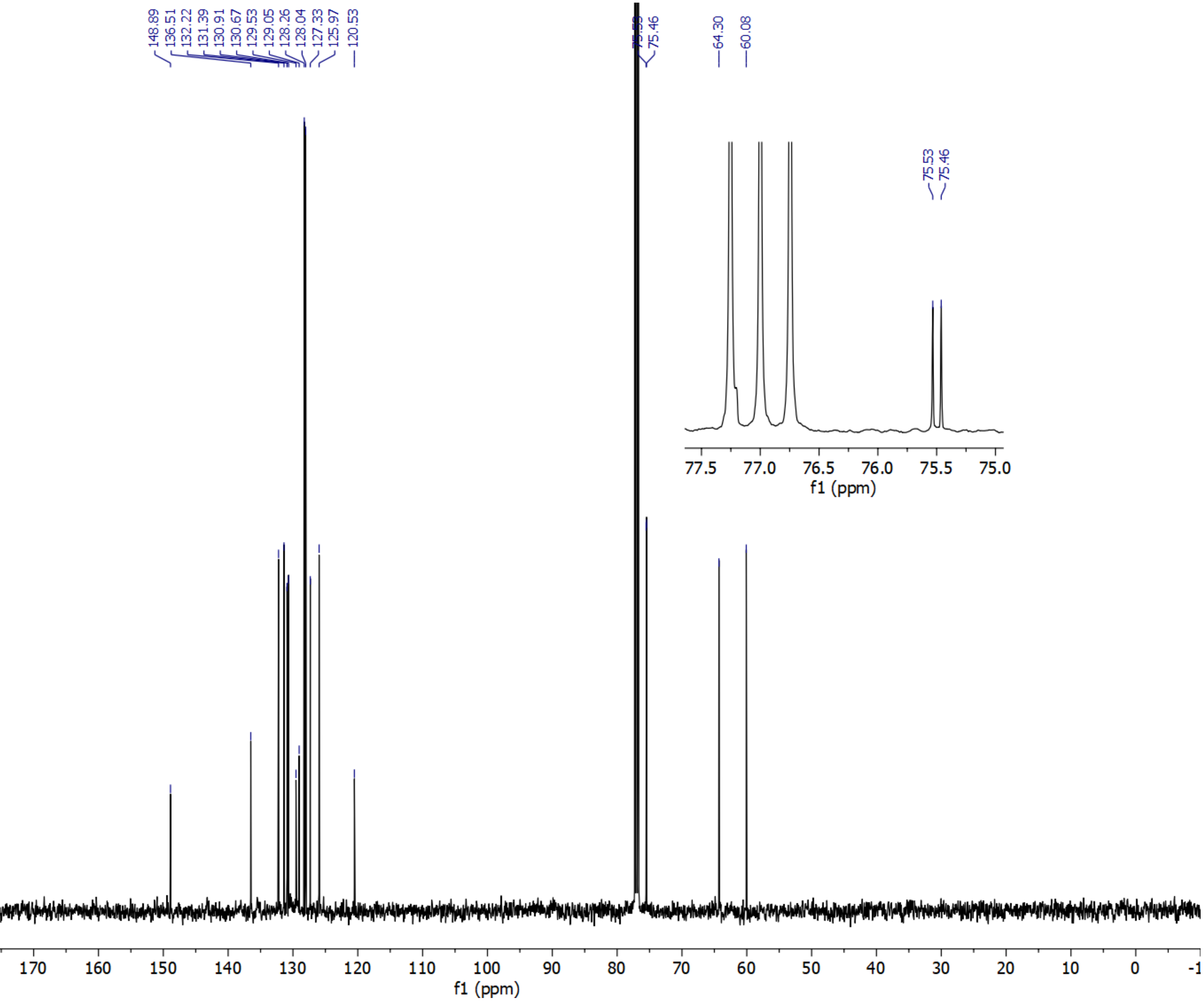

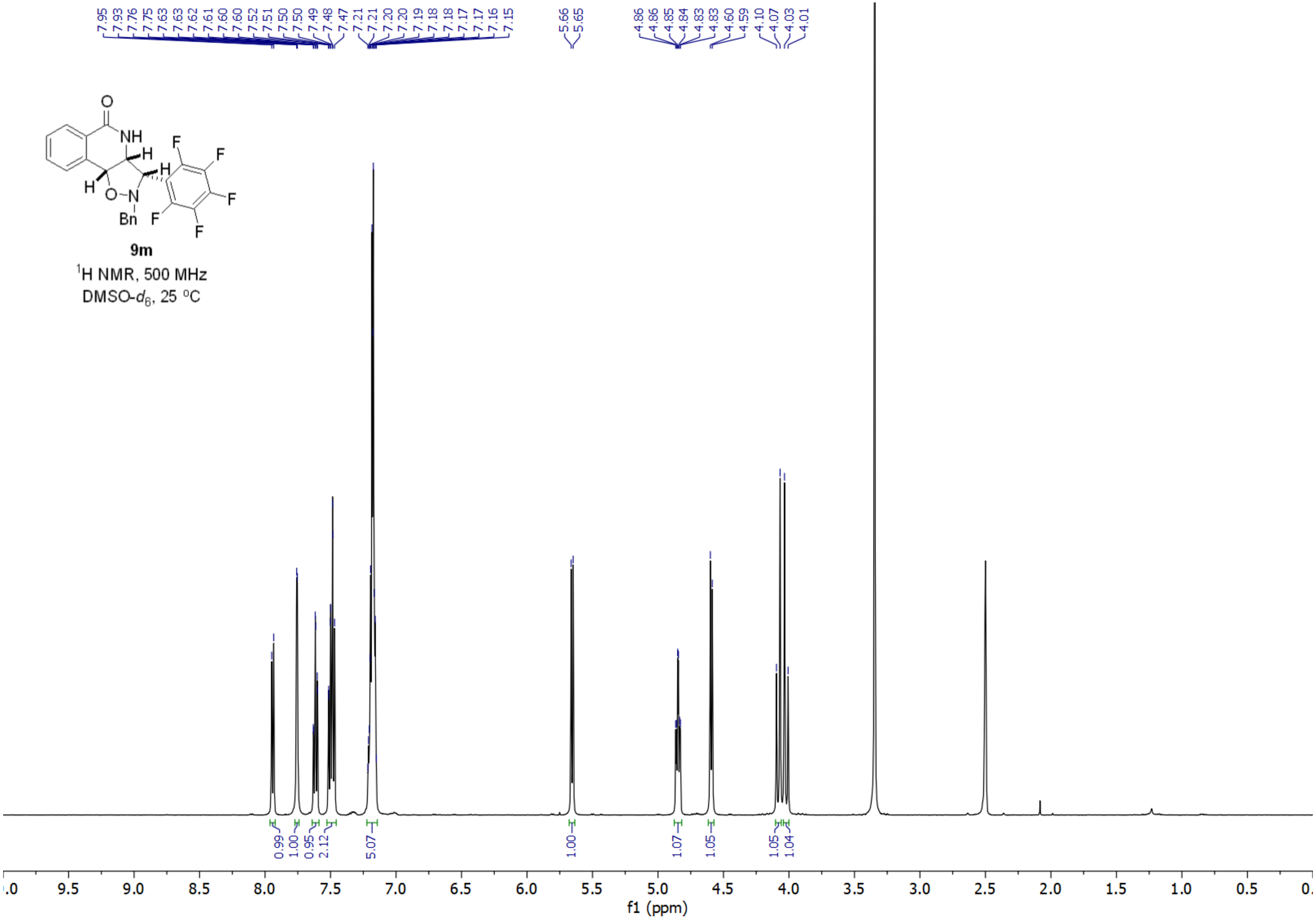

7.95 7.93 7.76 7.75 7.63 7.62 7.61 7.60 7.60 7.52 7.51 7.50 7.50 7.49 7.48 7.47 7.21 7.21 7.20 7.20 7.19 7.18 7.18 7.17 7.17 7.16 7.15
5.66 5.65
4.86 4.86 4.85 4.84 4.83 4.83 4.60 4.59 4.10 4.07 4.03 4.01
O
NH
H
H
H
O
N
Bn
F
F
F
F
F
9m
^{1}H NMR, 500 MHz
DMSO-d_6, 25 °C
0.99 1.00 0.95 2.12 5.07 1.00 1.07 1.05 1.05 1.04
9.5 9.0 8.5 8.0 7.5 7.0 6.5 6.0 5.5 5.0 4.5 4.0 3.5 3.0 2.5 2.0 1.5 1.0 0.5
f1 (ppm)

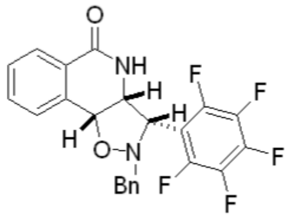

9m

^{13}C NMR, 125 MHz

DMSO-d_6, 25 °C

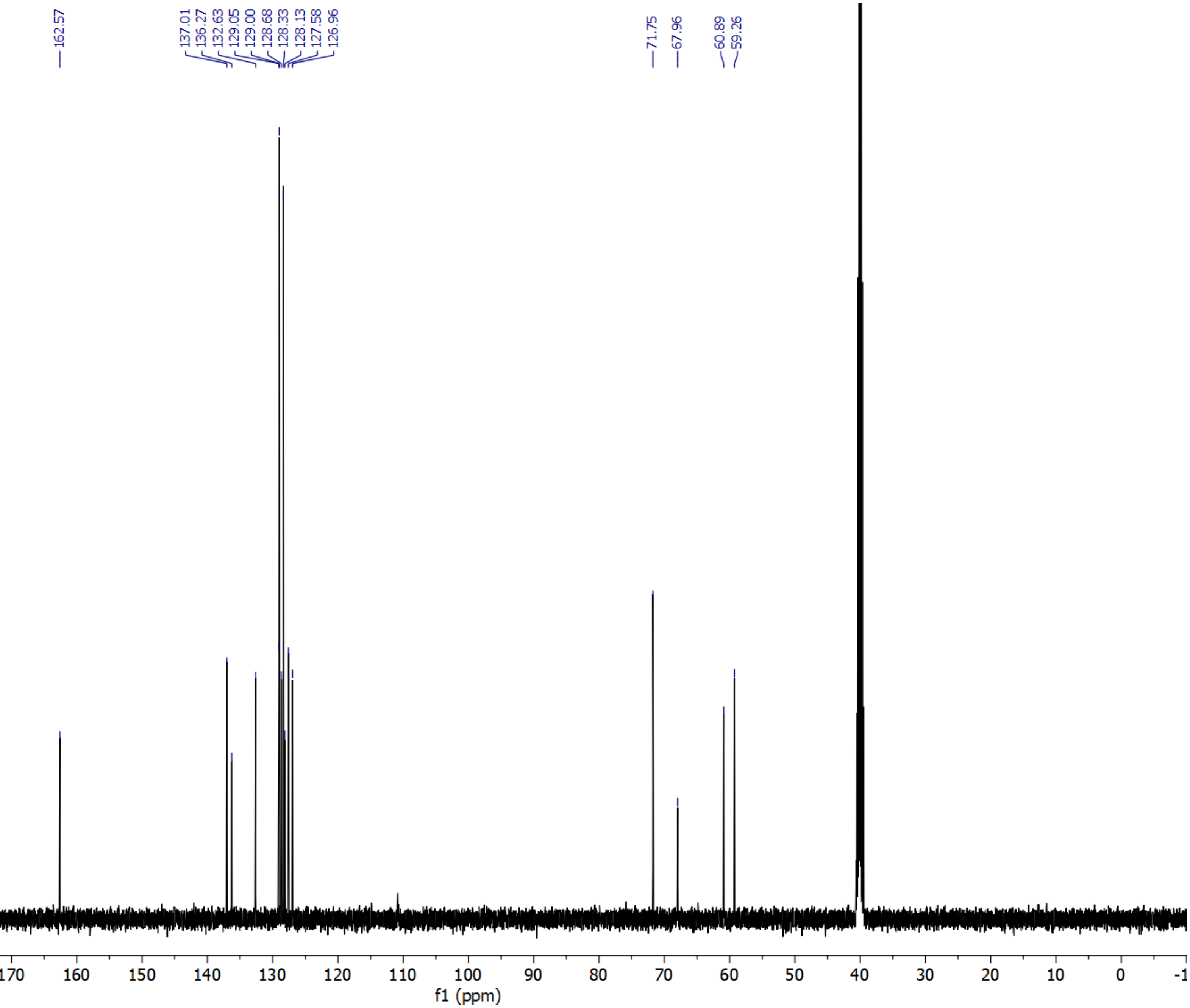

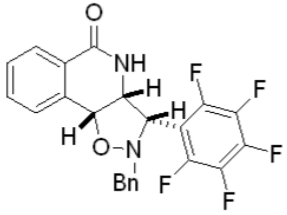

9m

^{19}F NMR, 470 MHz

DMSO-d_6, 25 °C

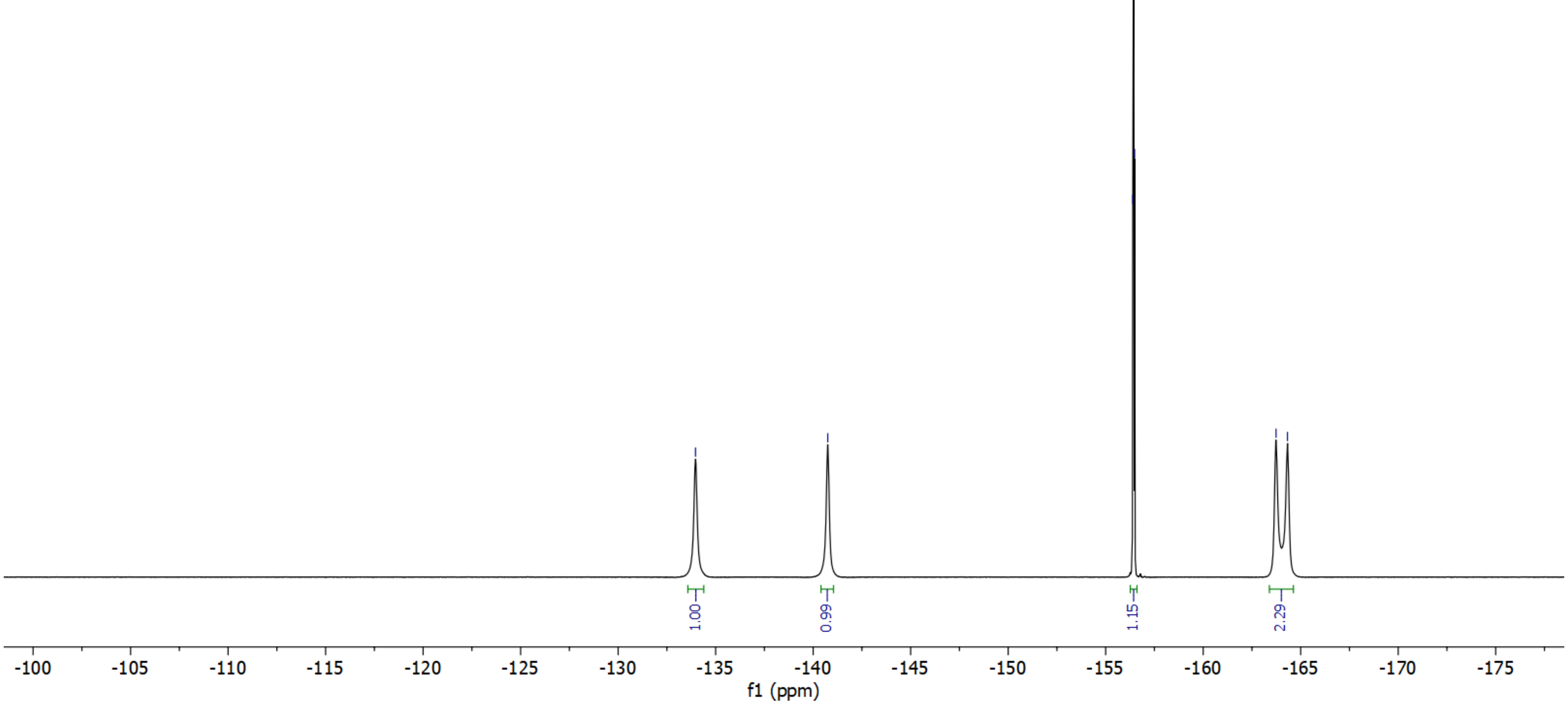

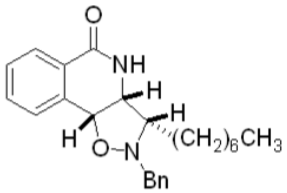

9n

^{1}H NMR, 500 MHz

DMSO-d_6, 25 °C

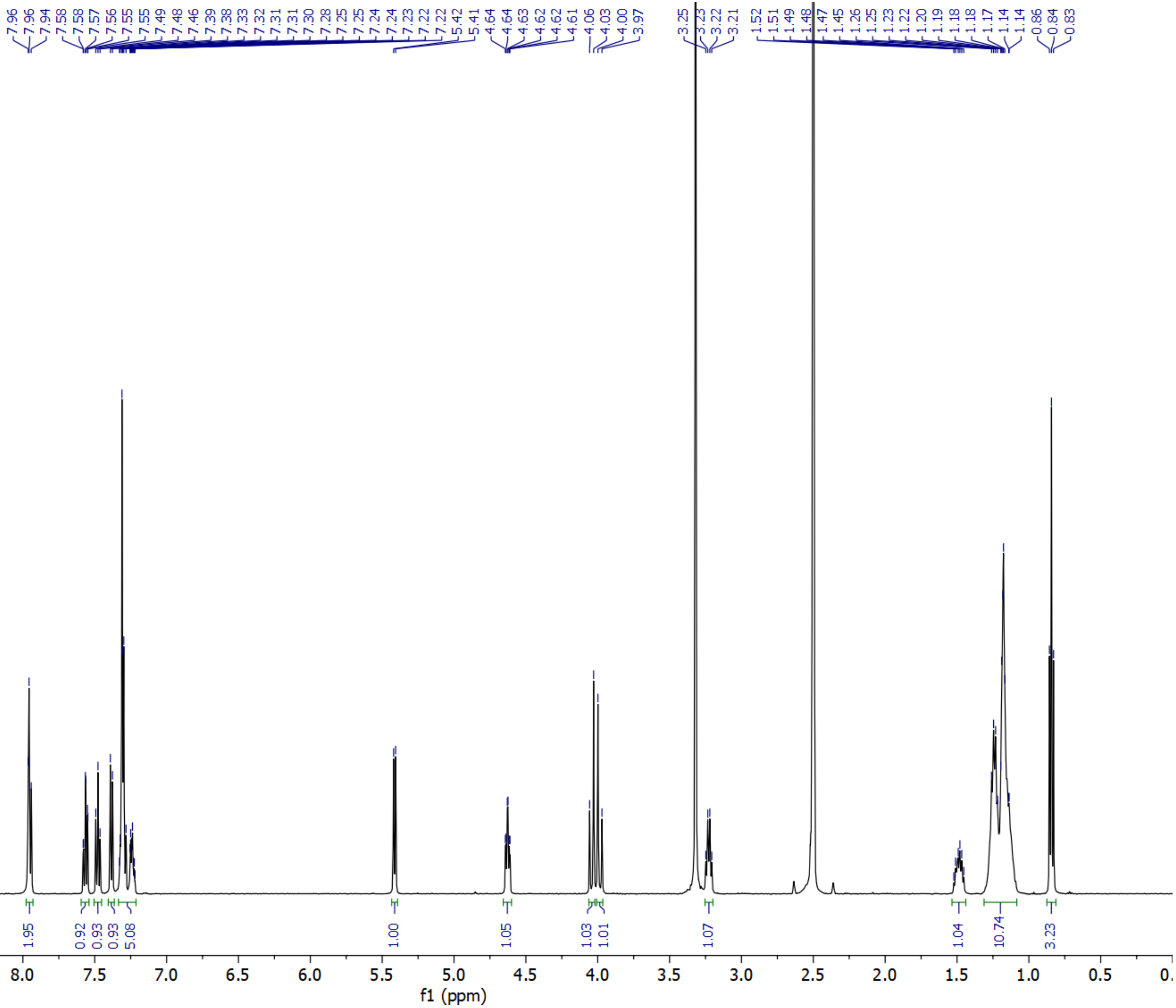

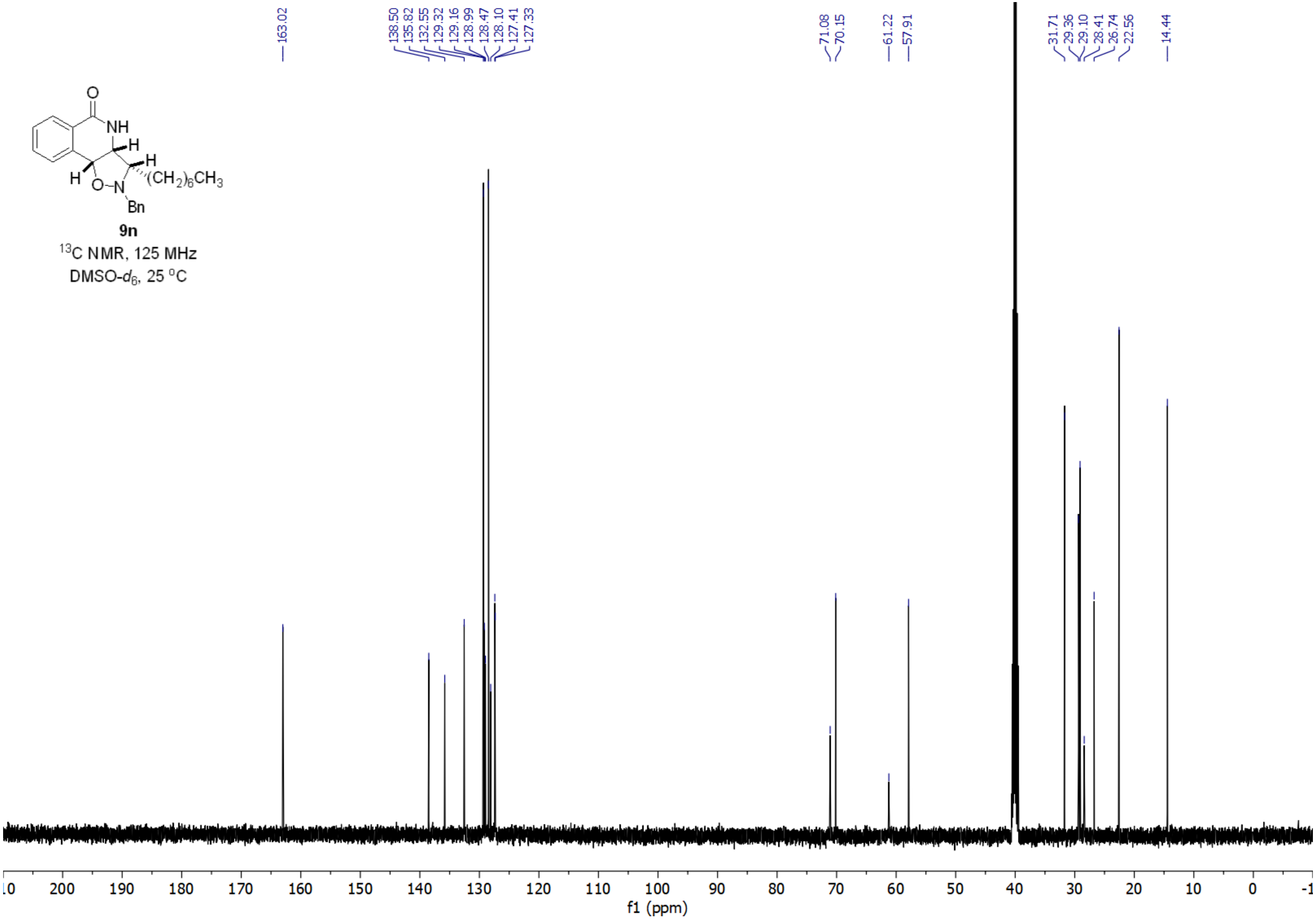

9n

^{13}C NMR, 125 MHz

DMSO-d_6, 25 °C

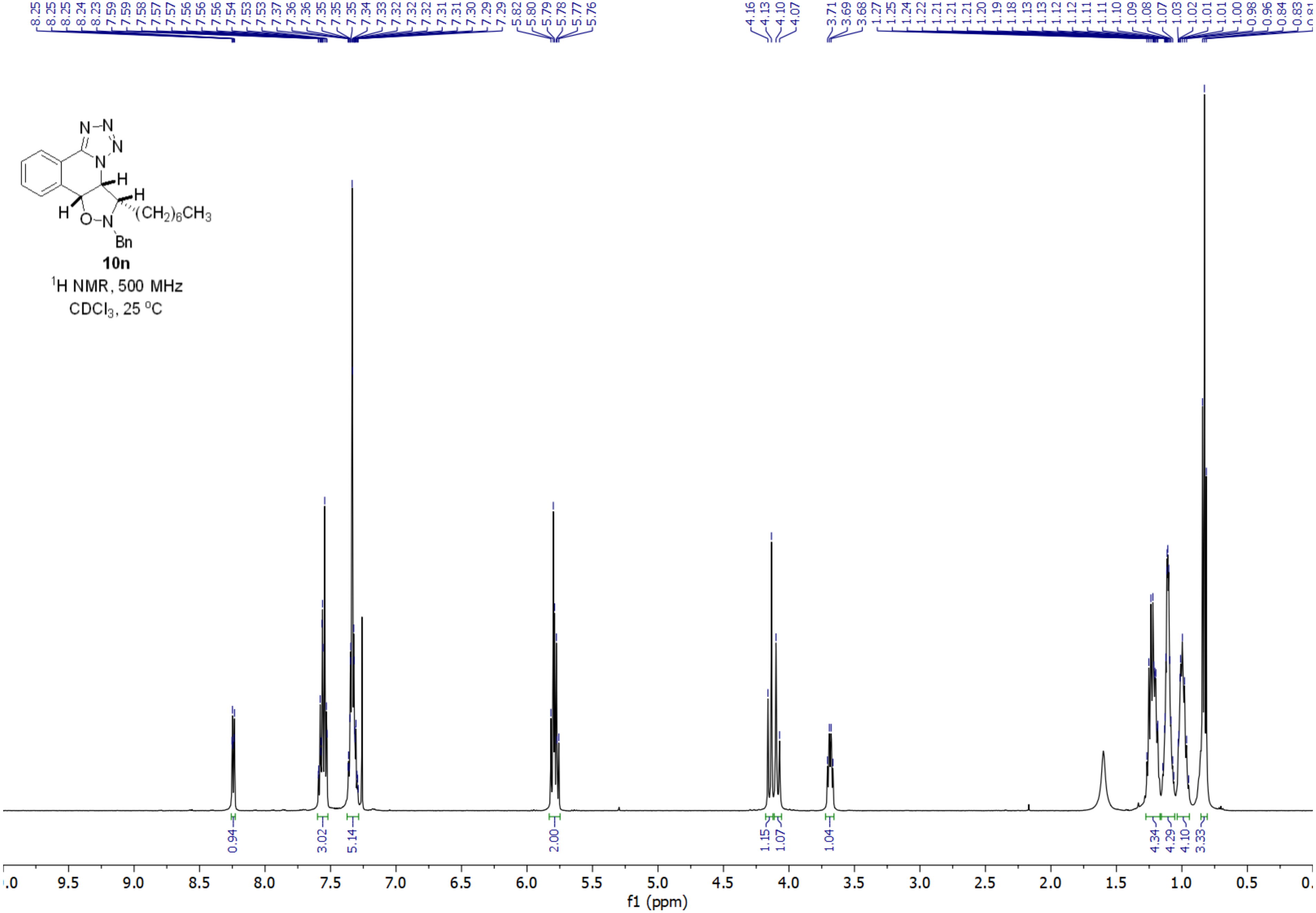
10n
1H NMR, 500 MHz
CDCl3, 25 °C
Bn
(CH2)6CH3
f1 (ppm)
0.94
3.02
5.14
2.00
1.15
1.07
1.04
4.34
4.29
4.10
3.33

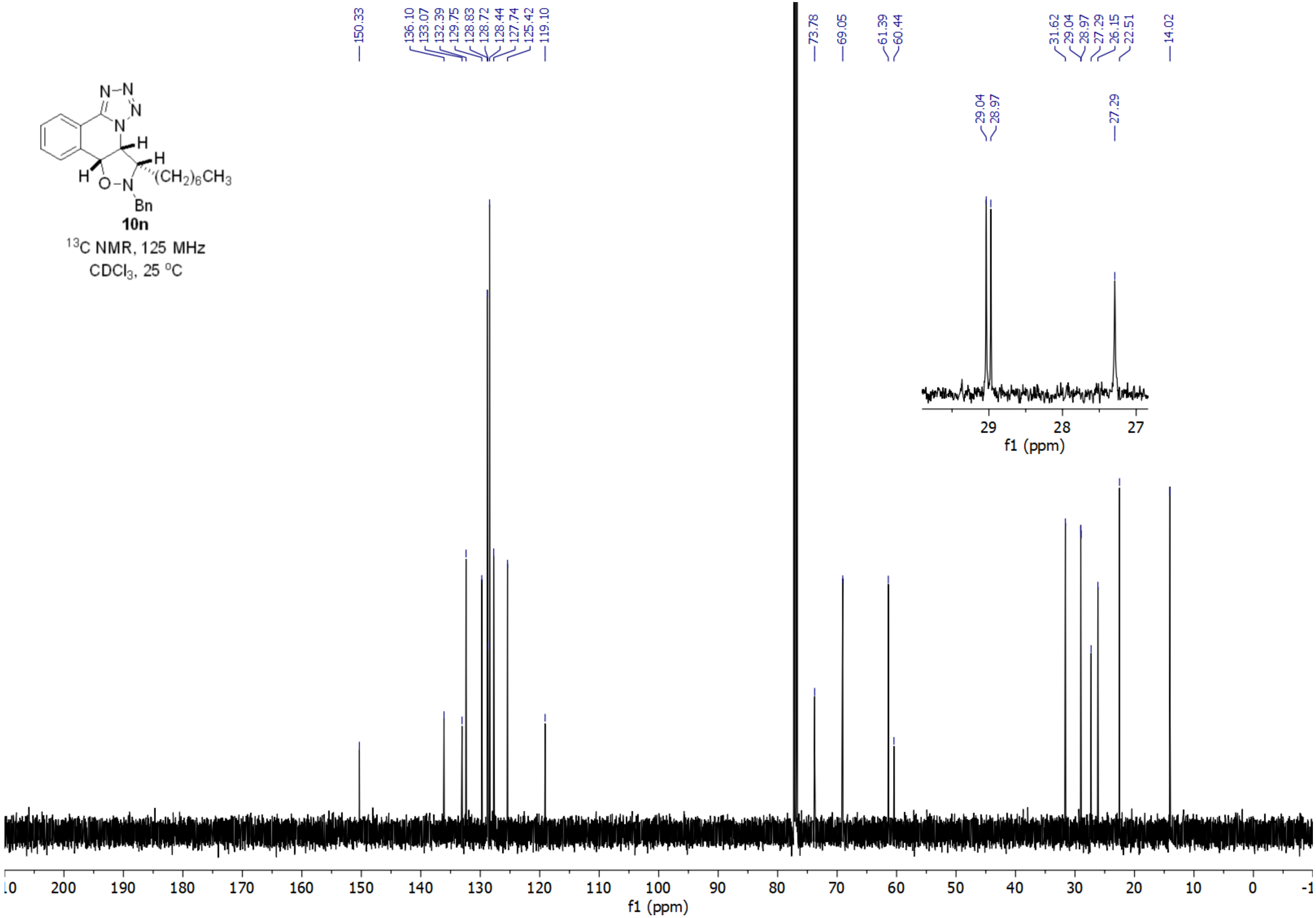

10n

^{13}C NMR, 125 MHz

$CDCl_3$, 25 °C

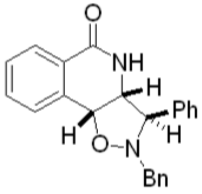

12a

^{1}H NMR, 500 MHz

DMSO-d_6, 60 ^{o}C

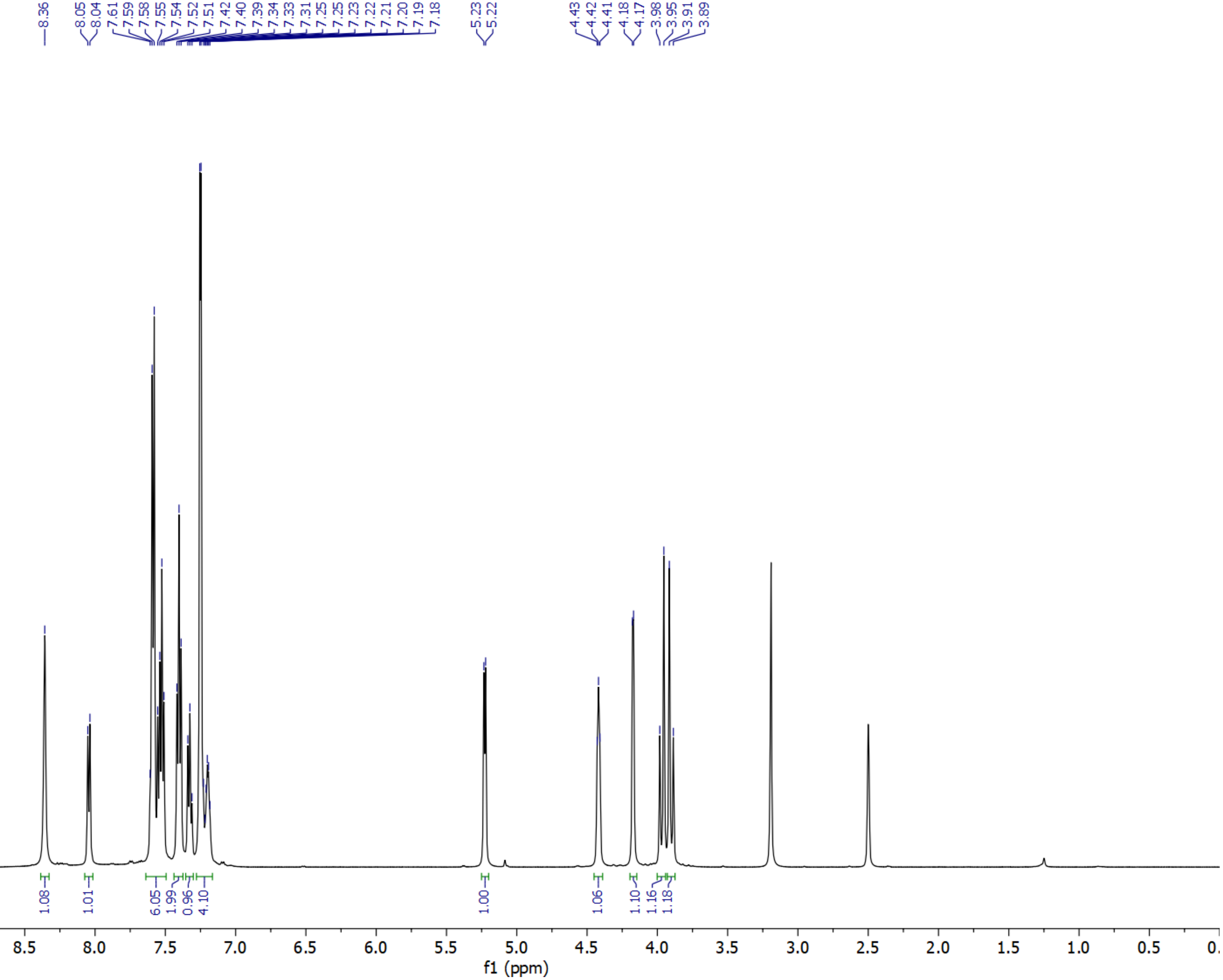

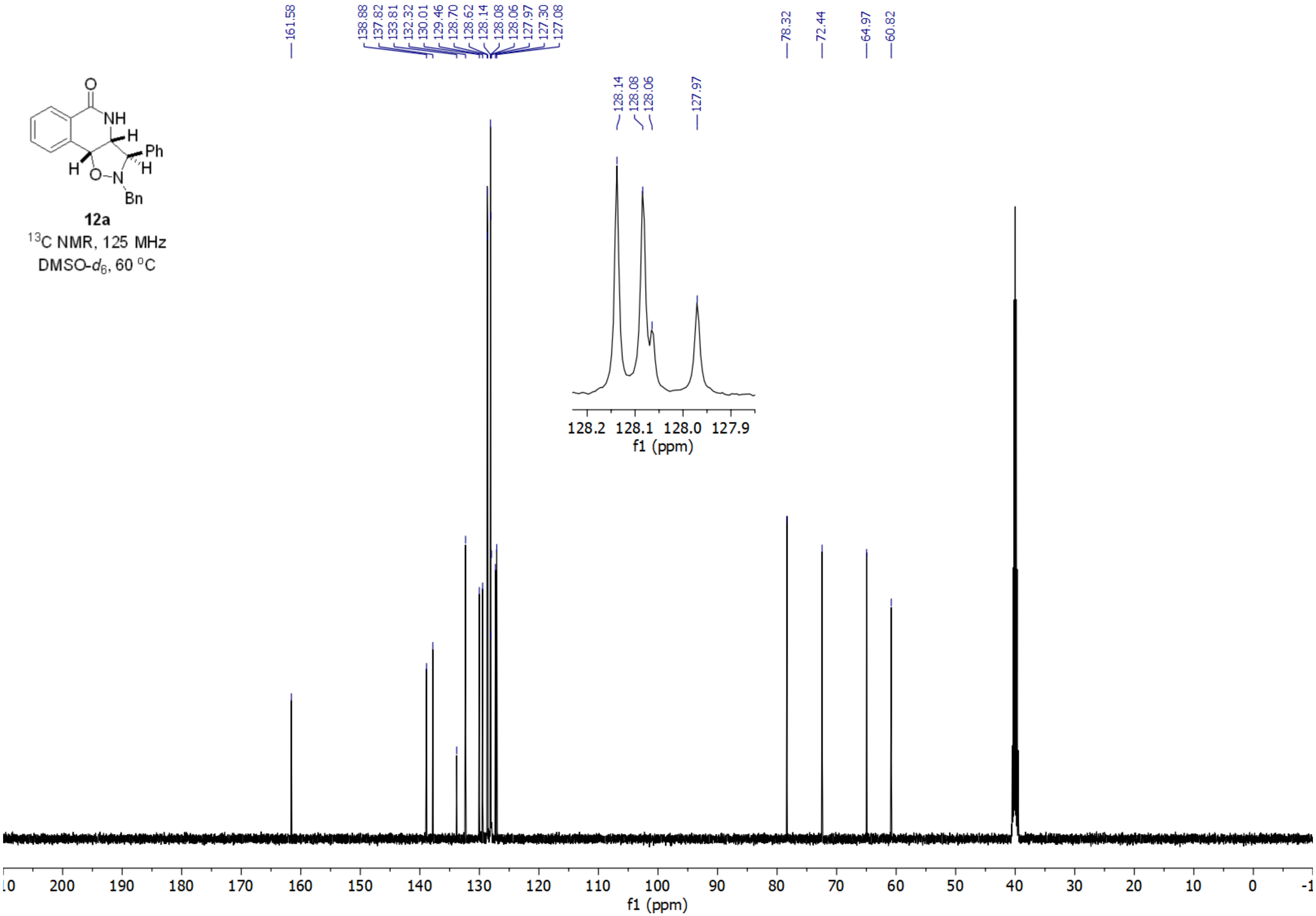
12a
^{13}C NMR, 125 MHz
DMSO-d_6, 60 °C
161.58
138.88
137.82
133.81
132.32
130.01
129.46
128.70
128.62
128.14
128.08
128.06
127.97
127.30
127.08
78.32
72.44
64.97
60.82
f1 (ppm)

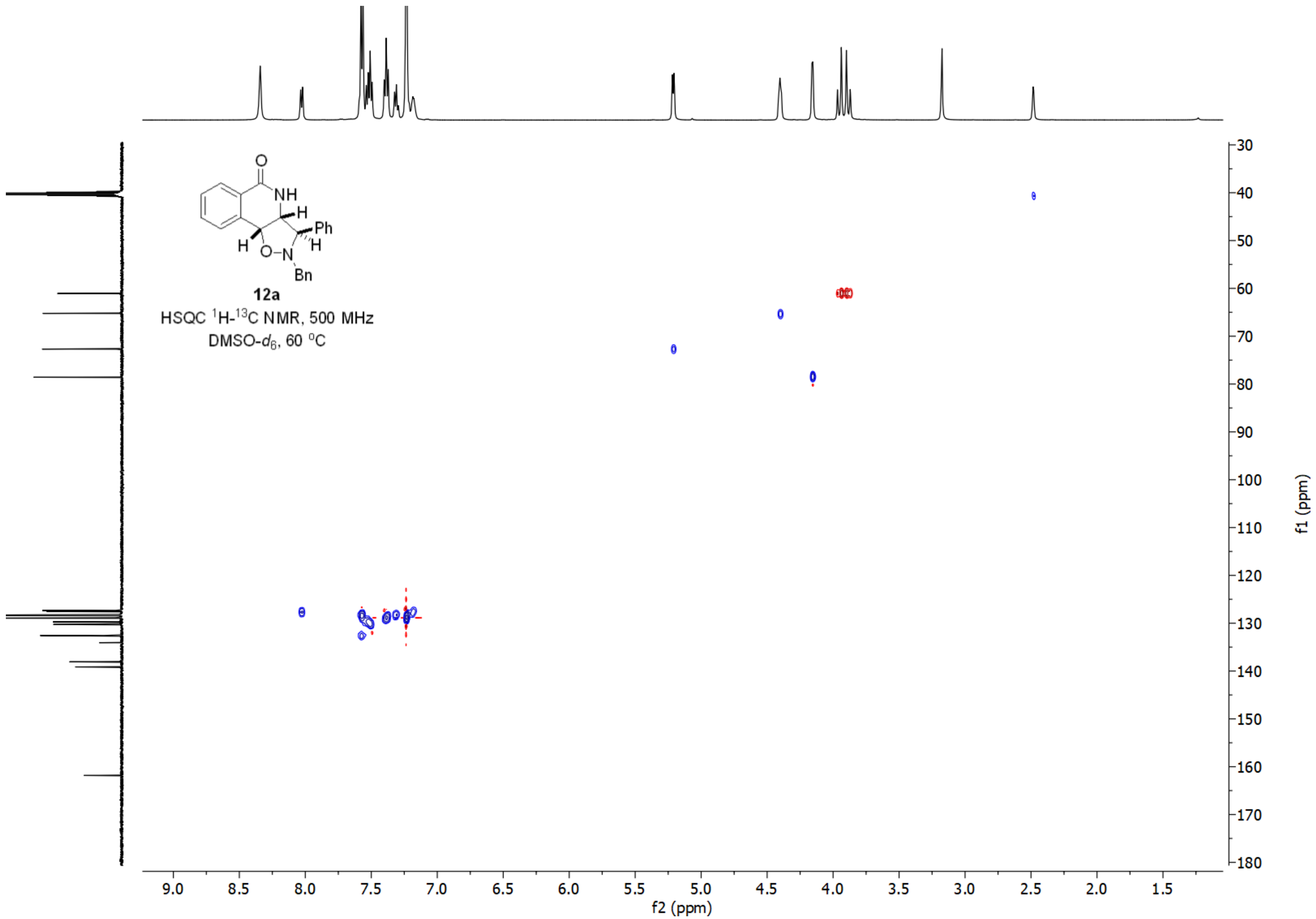
12a
HSQC ^{1}H-^{13}C NMR, 500 MHz
DMSO-d_6, 60 °C
f2 (ppm)
f1 (ppm)

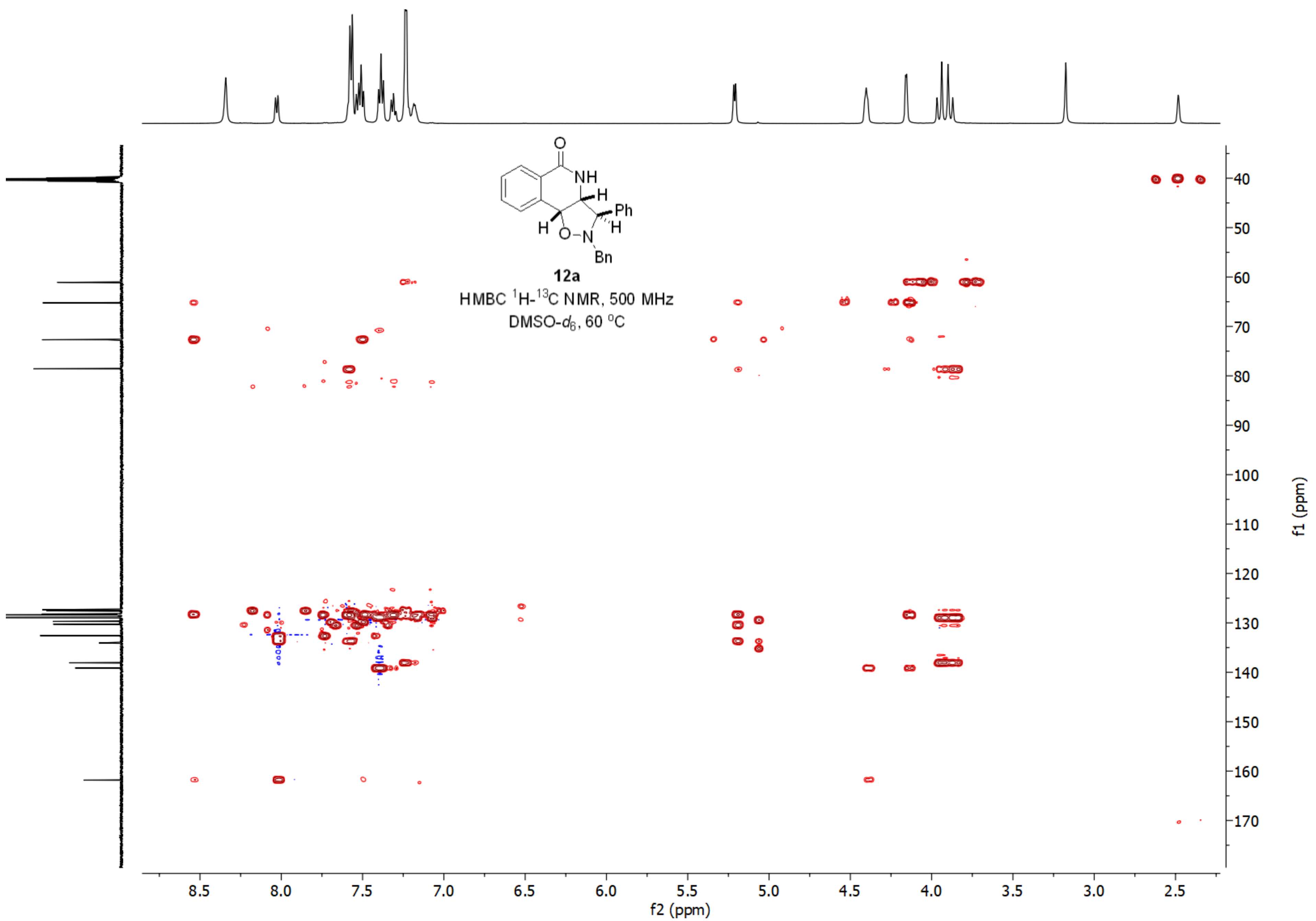

O
NH
H
Ph
H
O–N
H
Bn
12a
HMBC ^{1}H-^{13}C NMR, 500 MHz
DMSO-d_6, 60 °C
f2 (ppm)
f1 (ppm)

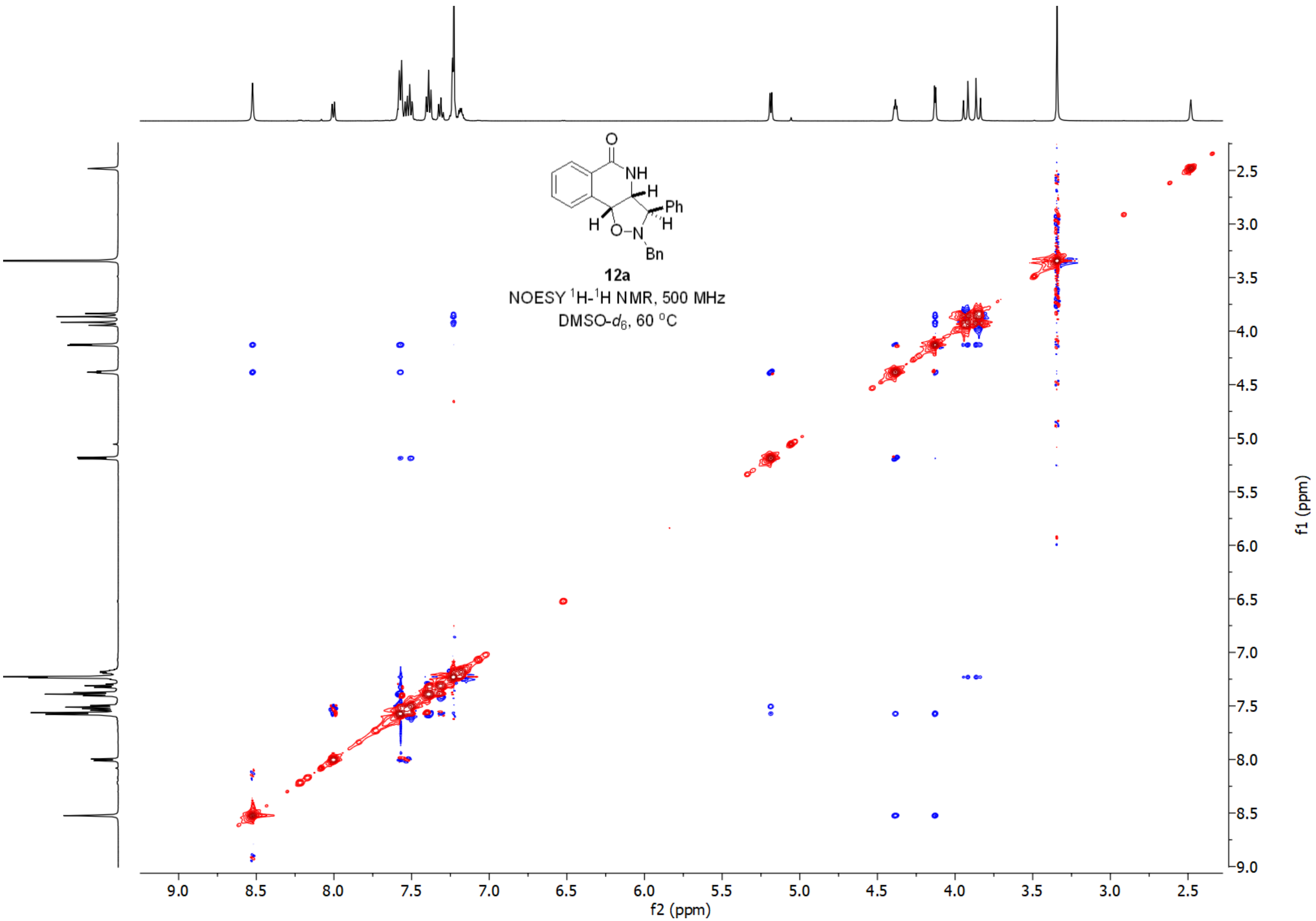
O
NH
H
Ph
H
H
O–N
Bn
12a
NOESY 1H-1H NMR, 500 MHz
DMSO-d6, 60 °C
f2 (ppm)
f1 (ppm)

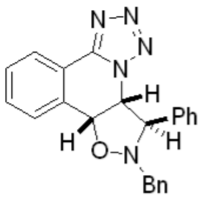

13a

^{1}H NMR, 500 MHz

$CDCl_3$, 25 ^{o}C

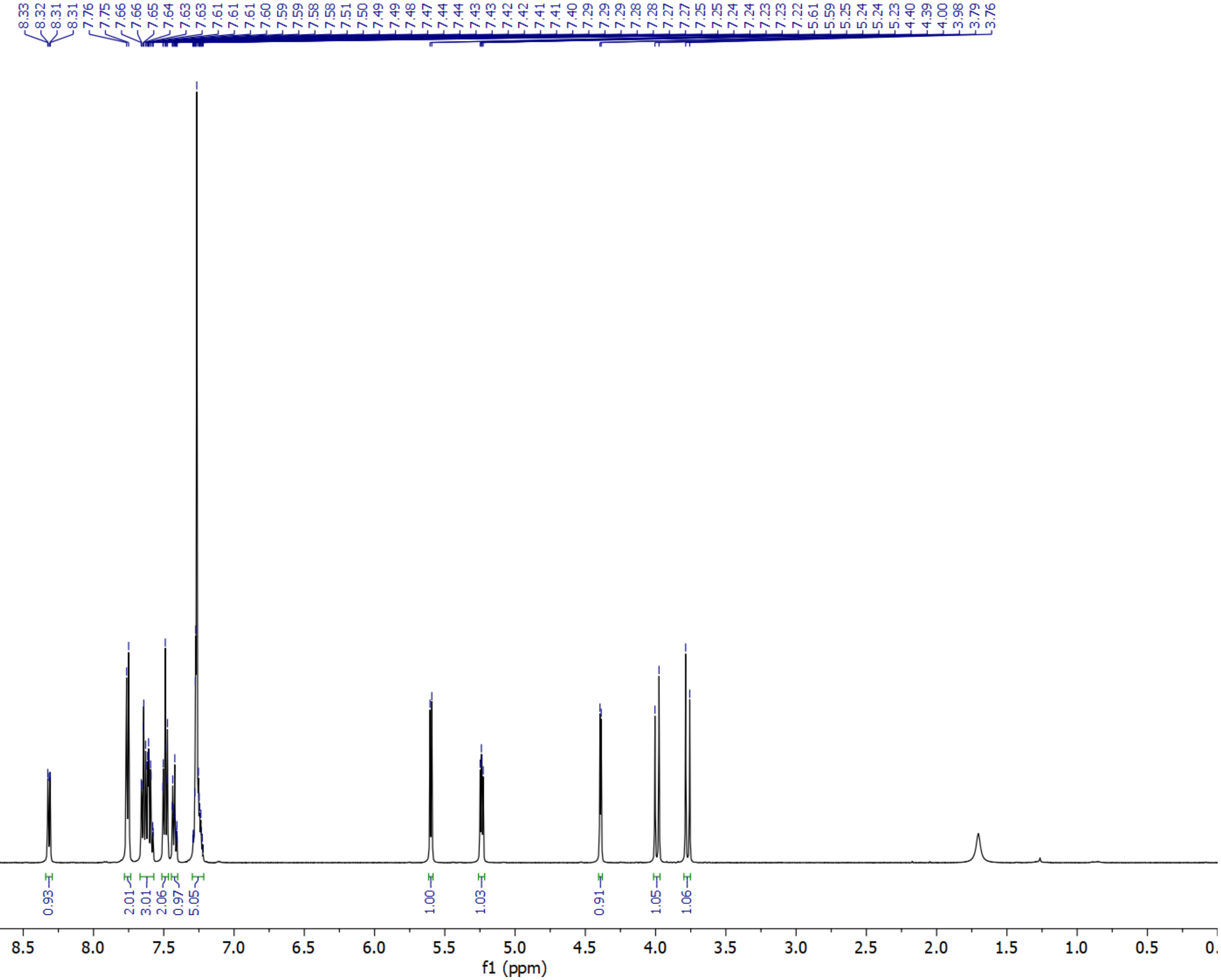

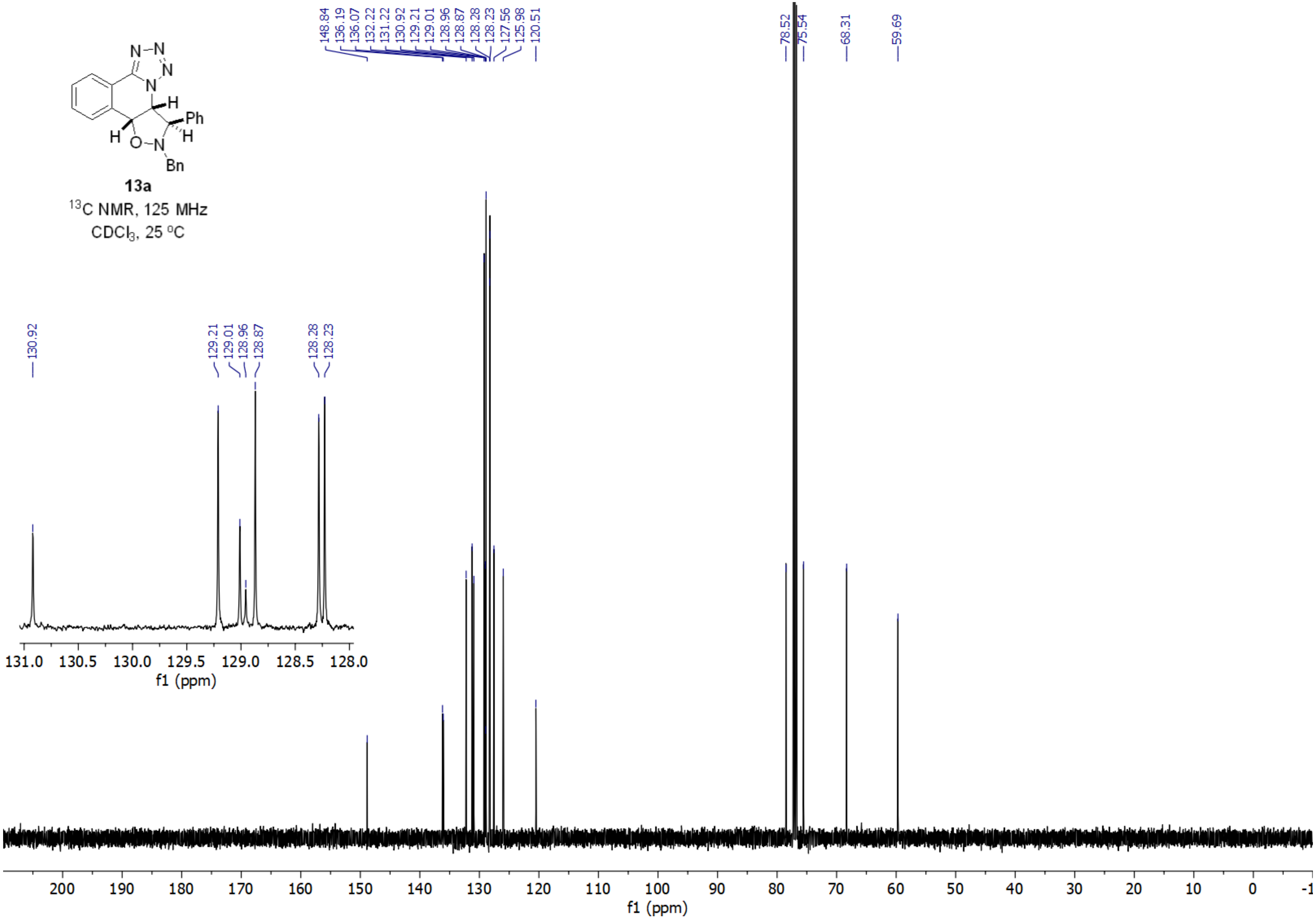

13a
^{13}C NMR, 125 MHz
$CDCl_3$, 25 °C
148.84
136.19
136.07
132.22
131.22
130.92
129.21
129.01
128.96
128.87
128.28
128.23
127.56
125.98
120.51
78.52
75.54
68.31
59.69
f1 (ppm)

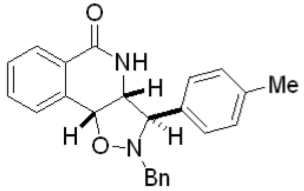

12b

^{1}H NMR, 500 MHz

DMSO-d_6, 60 °C

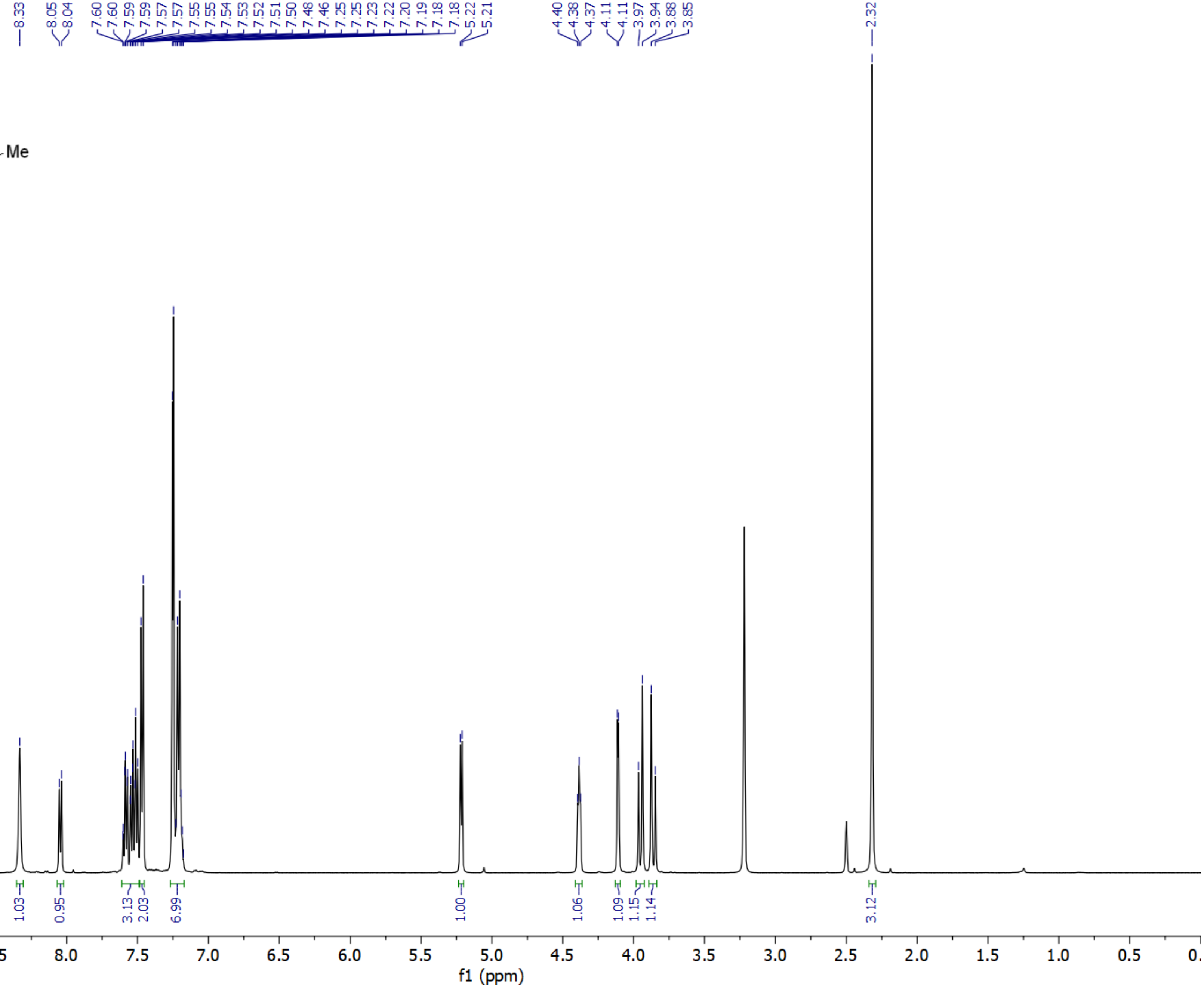

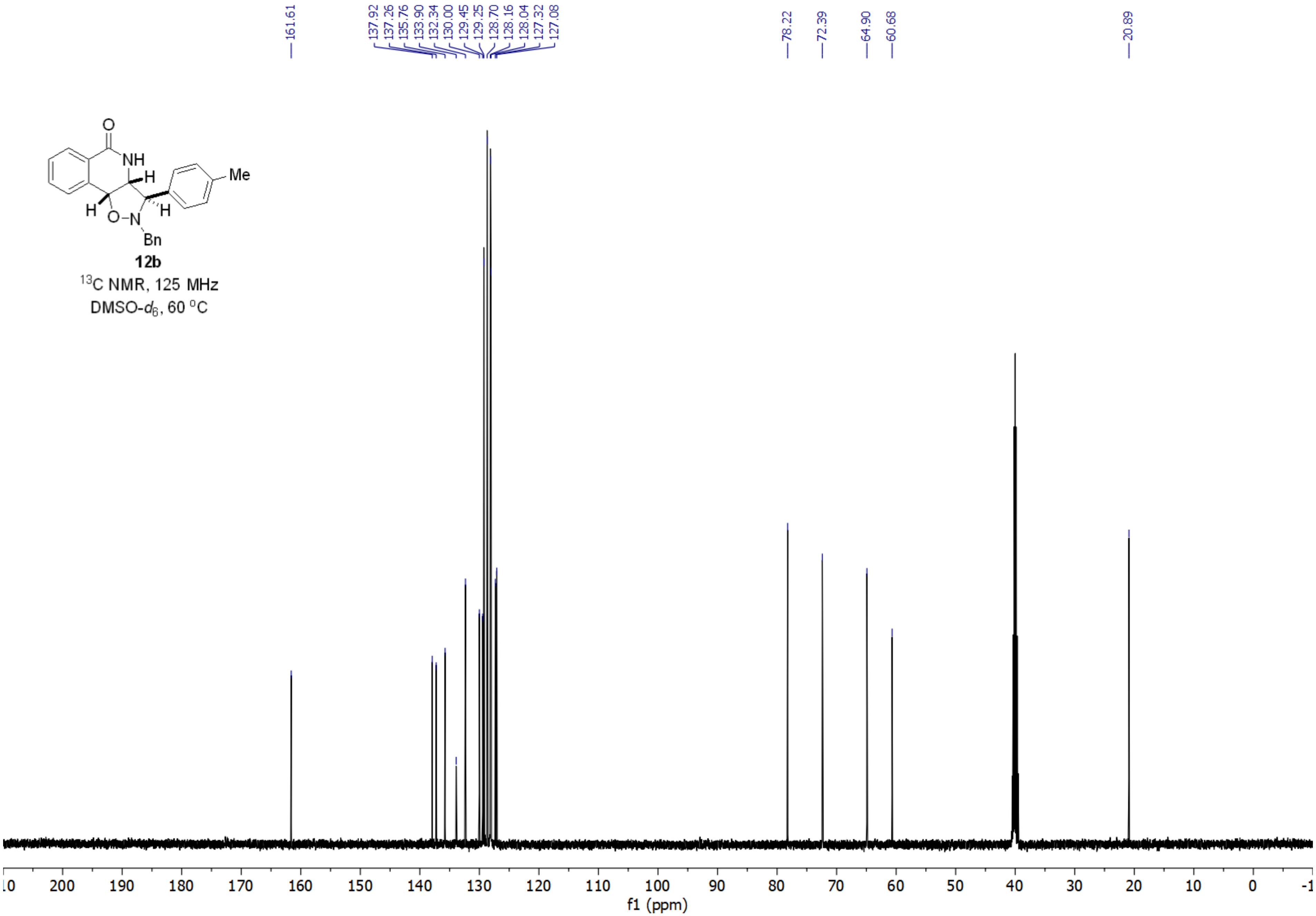
161.61
137.92
137.26
135.76
133.90
132.34
130.00
129.45
129.25
128.70
128.16
128.04
127.32
127.08
78.22
72.39
64.90
60.68
20.89
O
NH
H
Me
H
H
O–N
Bn
12b
^{13}C NMR, 125 MHz
DMSO-d_6, 60 °C
f1 (ppm)

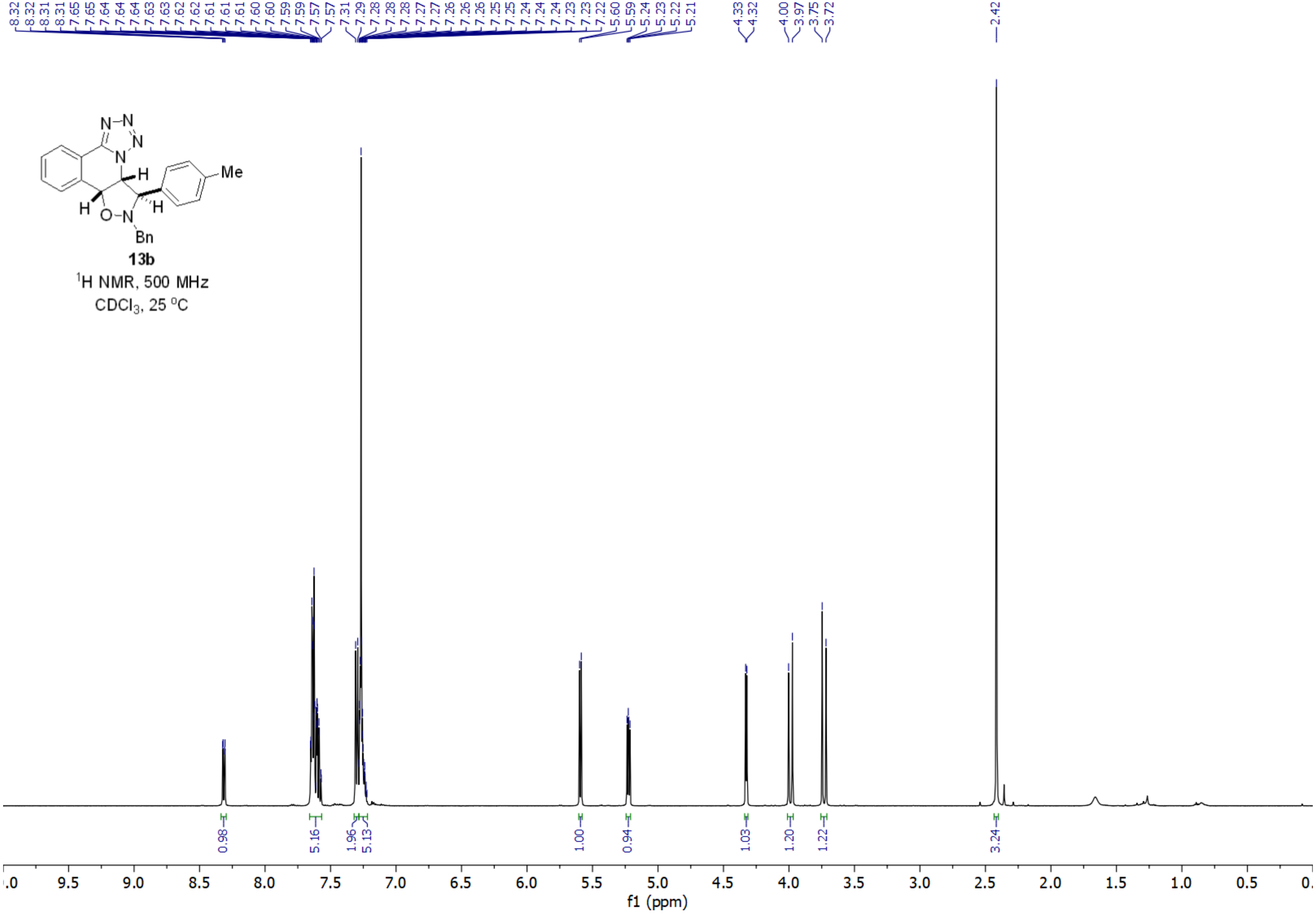

13b
¹H NMR, 500 MHz
CDCl3, 25 °C
8.32 8.32 8.31 8.31 7.65 7.65 7.64 7.64 7.64 7.63 7.63 7.62 7.61 7.61 7.61 7.60 7.60 7.59 7.59 7.57 7.57 7.31 7.29 7.28 7.28 7.28 7.27 7.27 7.26 7.26 7.26 7.25 7.25 7.24 7.24 7.24 7.23 7.23 7.22 5.60 5.59 5.24 5.23 5.22 5.21
4.33 4.32
4.00 3.97 3.75 3.72
2.42
0.98 5.16 1.96 5.13 1.00 0.94 1.03 1.20 1.22 3.24
f1 (ppm)

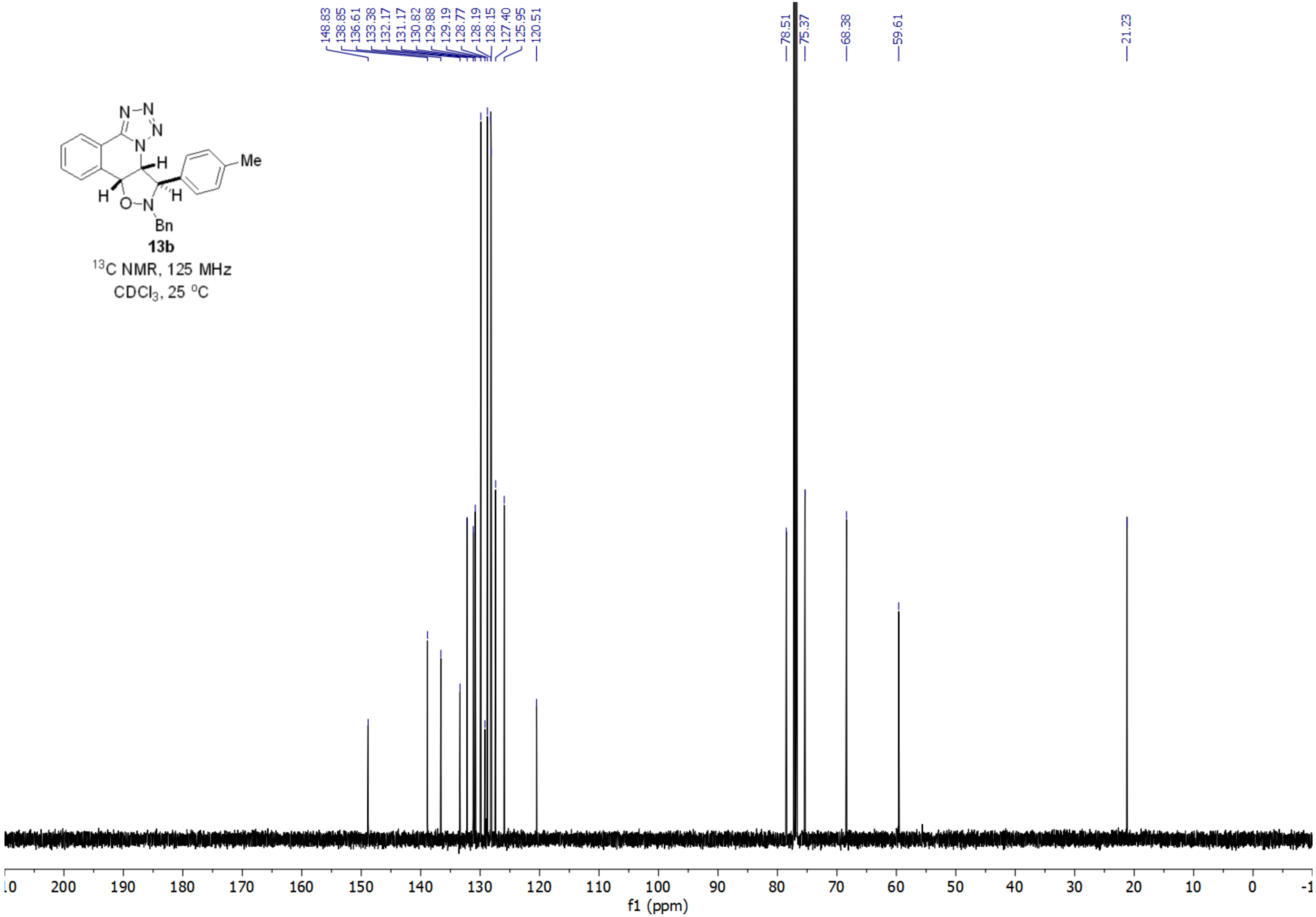

13b

^{13}C NMR, 125 MHz

$CDCl_3$, 25 °C

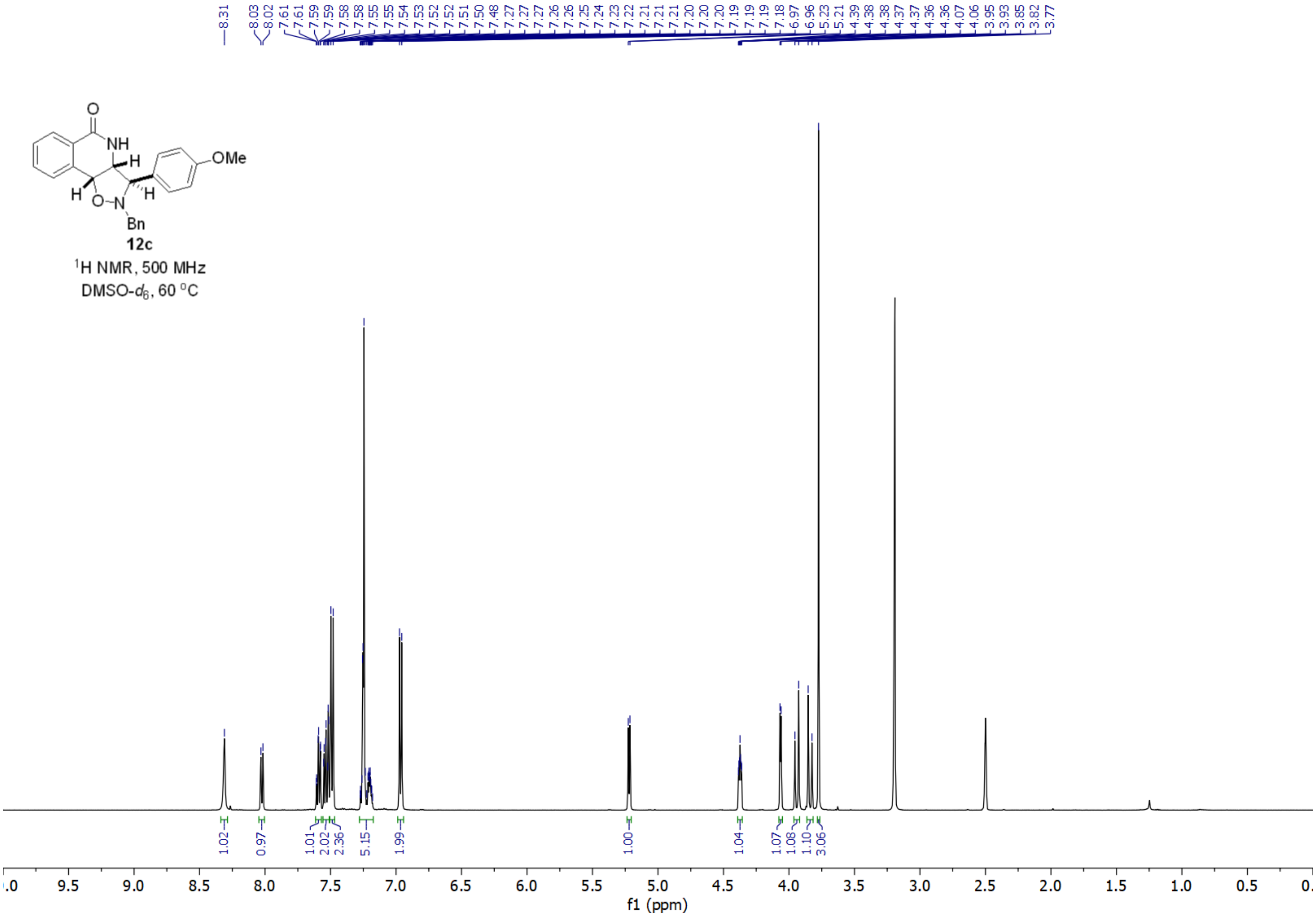

NH
H
OMe
H
H
O
N
Bn
12c
^{1}H NMR, 500 MHz
DMSO-d_6, 60 °C
f1 (ppm)

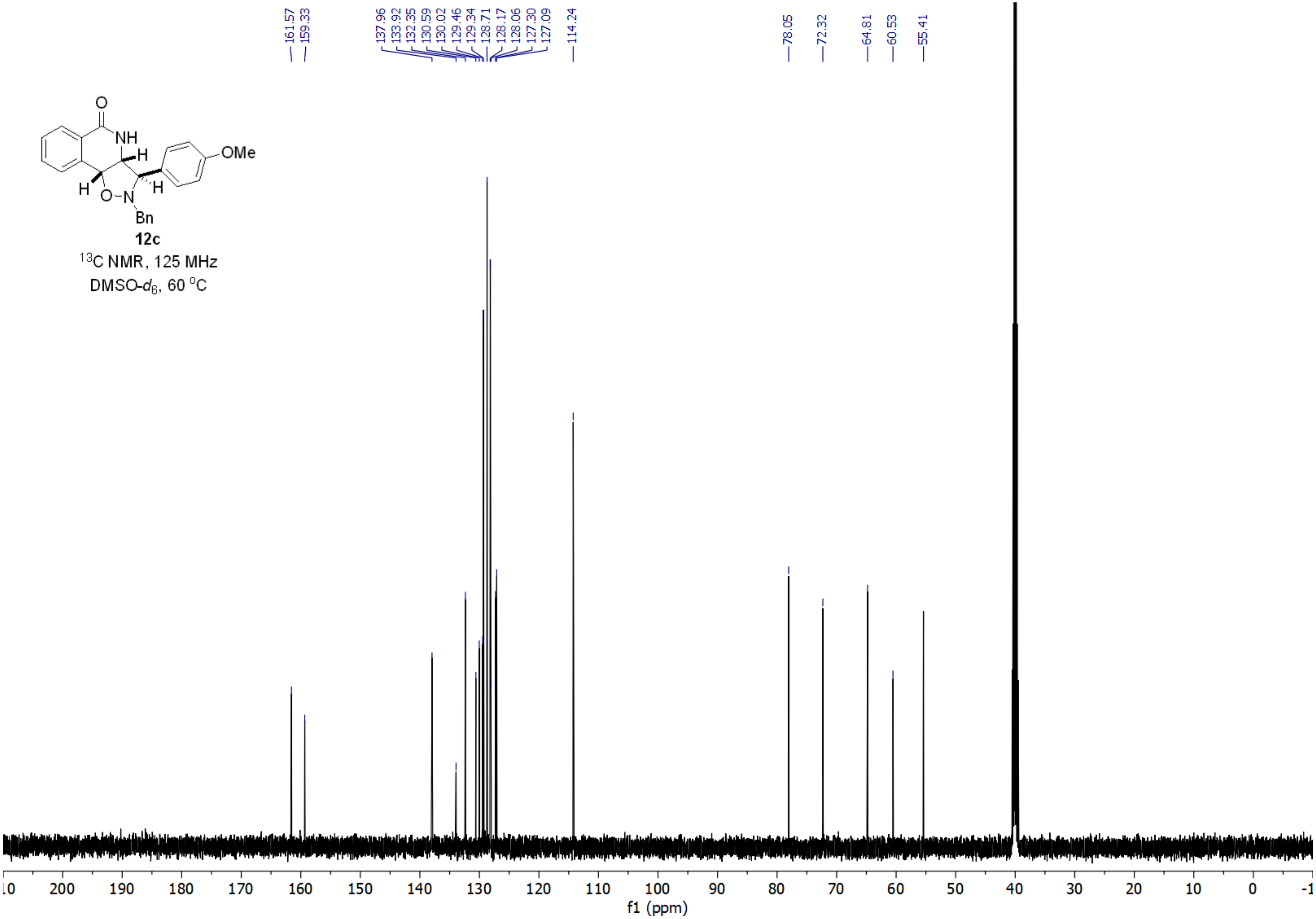

12c

^{13}C NMR, 125 MHz

DMSO-d_6, 60 °C

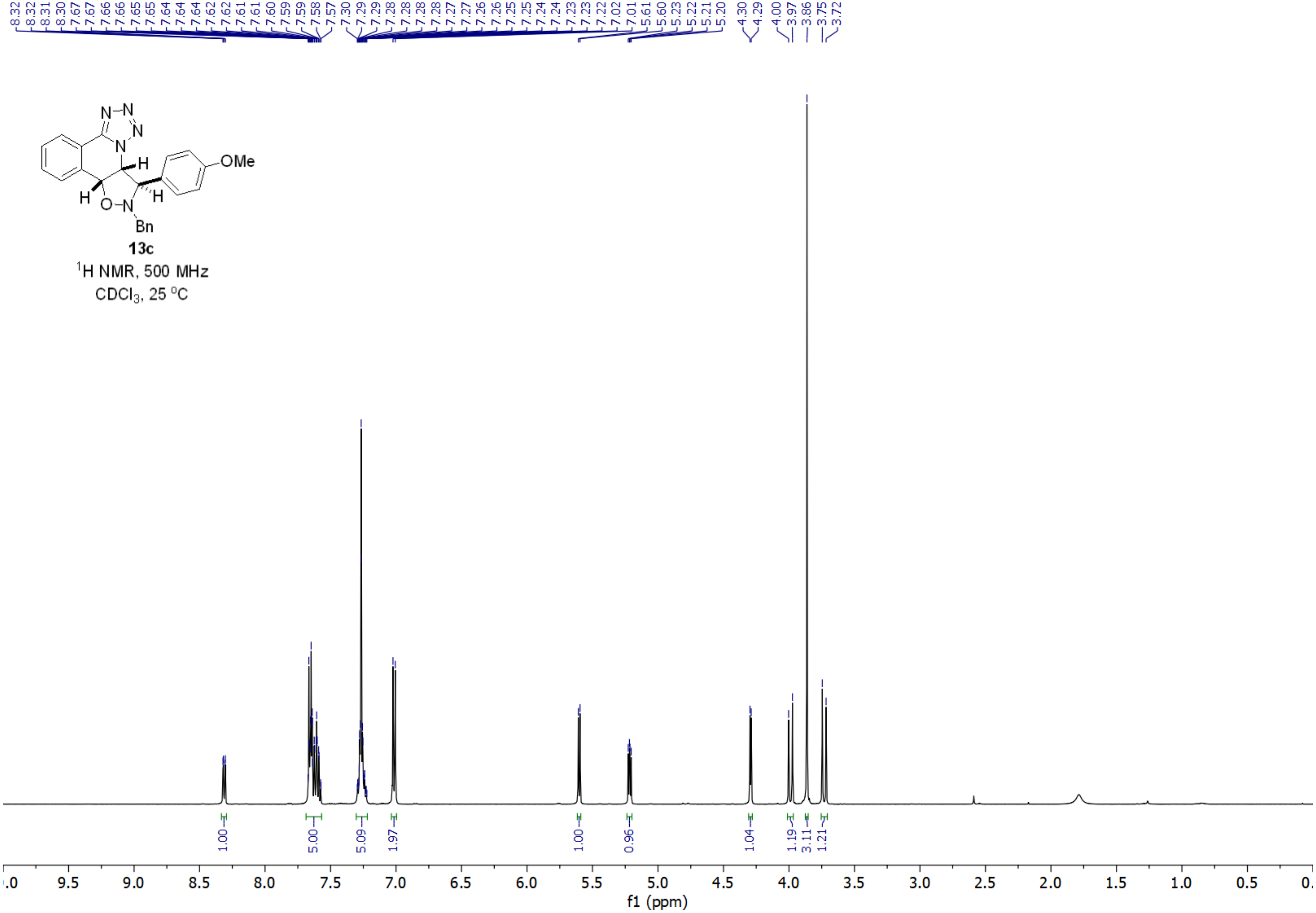
8.32 8.32 8.31 8.30 7.67 7.67 7.66 7.66 7.65 7.65 7.64 7.64 7.62 7.62 7.61 7.61 7.60 7.59 7.59 7.58 7.57 7.30 7.29 7.29 7.28 7.28 7.28 7.27 7.26 7.26 7.25 7.25 7.24 7.24 7.23 7.23 7.22 7.02 7.01 5.61 5.60 5.23 5.22 5.21 5.20 4.30 4.29 4.00 3.97 3.86 3.75 3.72
OMe
H
H
H
O
N
Bn
13c
^{1}H NMR, 500 MHz
$CDCl_3$, 25 °C
1.00
5.00
5.09
1.97
1.00
0.96
1.04
1.19
3.11
1.21
f1 (ppm)

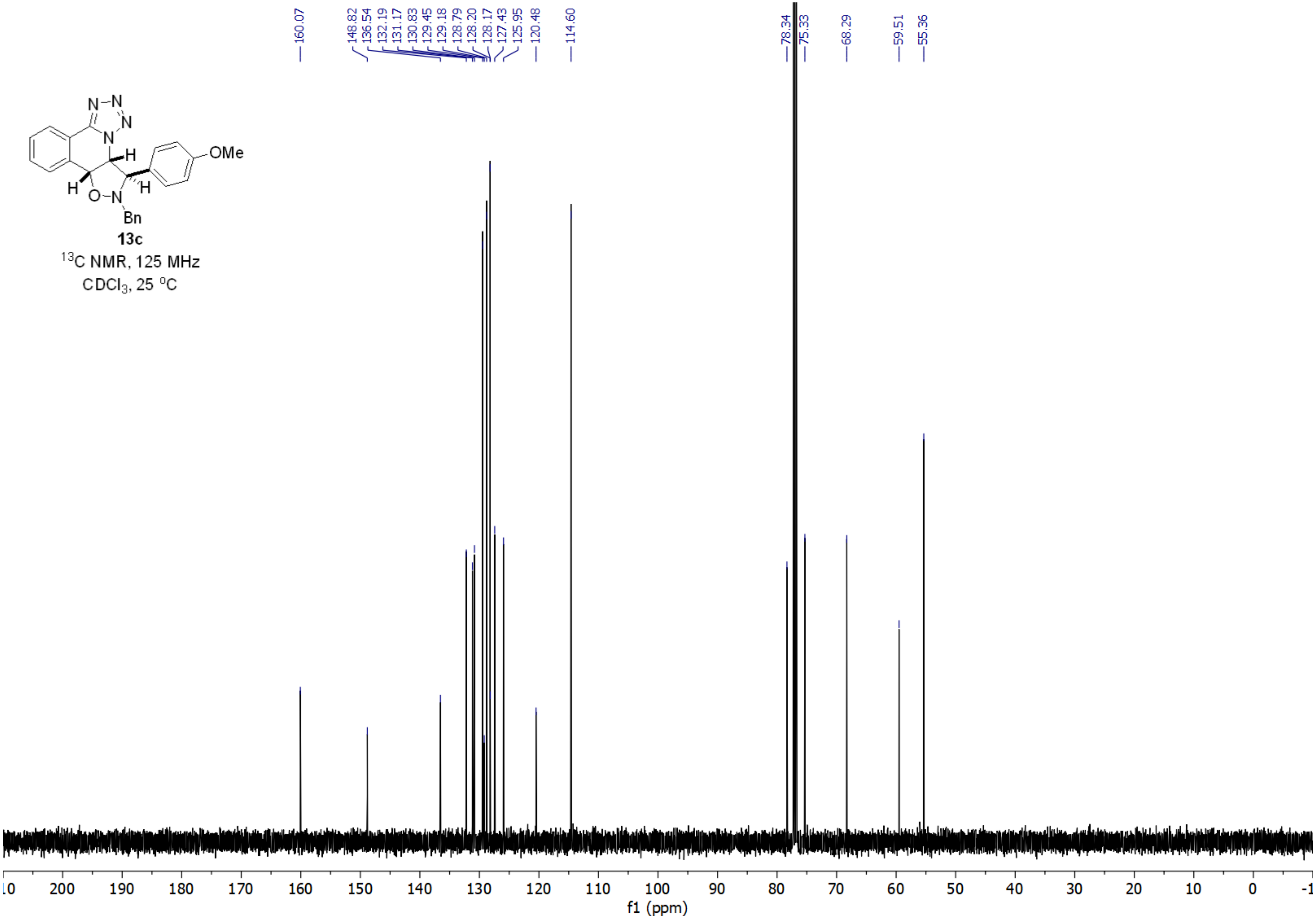

13c

^{13}C NMR, 125 MHz

$CDCl_3$, 25 °C

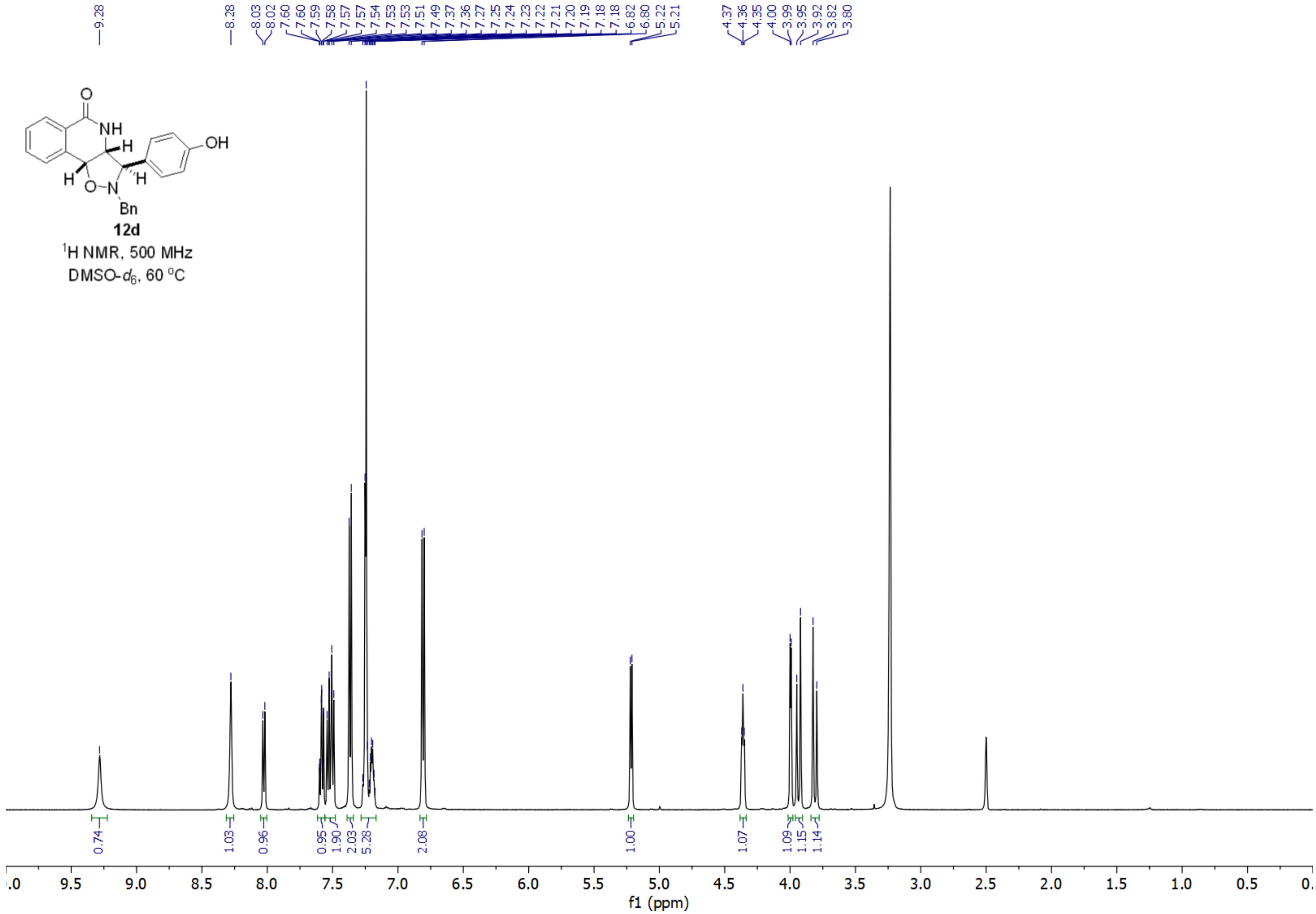

9.28
8.28
8.03
8.02
7.60
7.60
7.59
7.58
7.57
7.57
7.54
7.53
7.53
7.51
7.49
7.37
7.36
7.27
7.25
7.24
7.23
7.22
7.21
7.20
7.19
7.18
7.18
6.82
6.80
5.22
5.21
4.37
4.36
4.35
4.00
3.99
3.95
3.92
3.82
3.80
O
NH
H
OH
H
O
N
H
Bn
12d
^{1}H NMR, 500 MHz
DMSO-d_6, 60 °C
0.74
1.03
0.96
0.95
1.90
2.03
5.28
2.08
1.00
1.07
1.09
1.15
1.14
9.5
9.0
8.5
8.0
7.5
7.0
6.5
6.0
5.5
5.0
4.5
4.0
3.5
3.0
2.5
2.0
1.5
1.0
0.5
f1 (ppm)

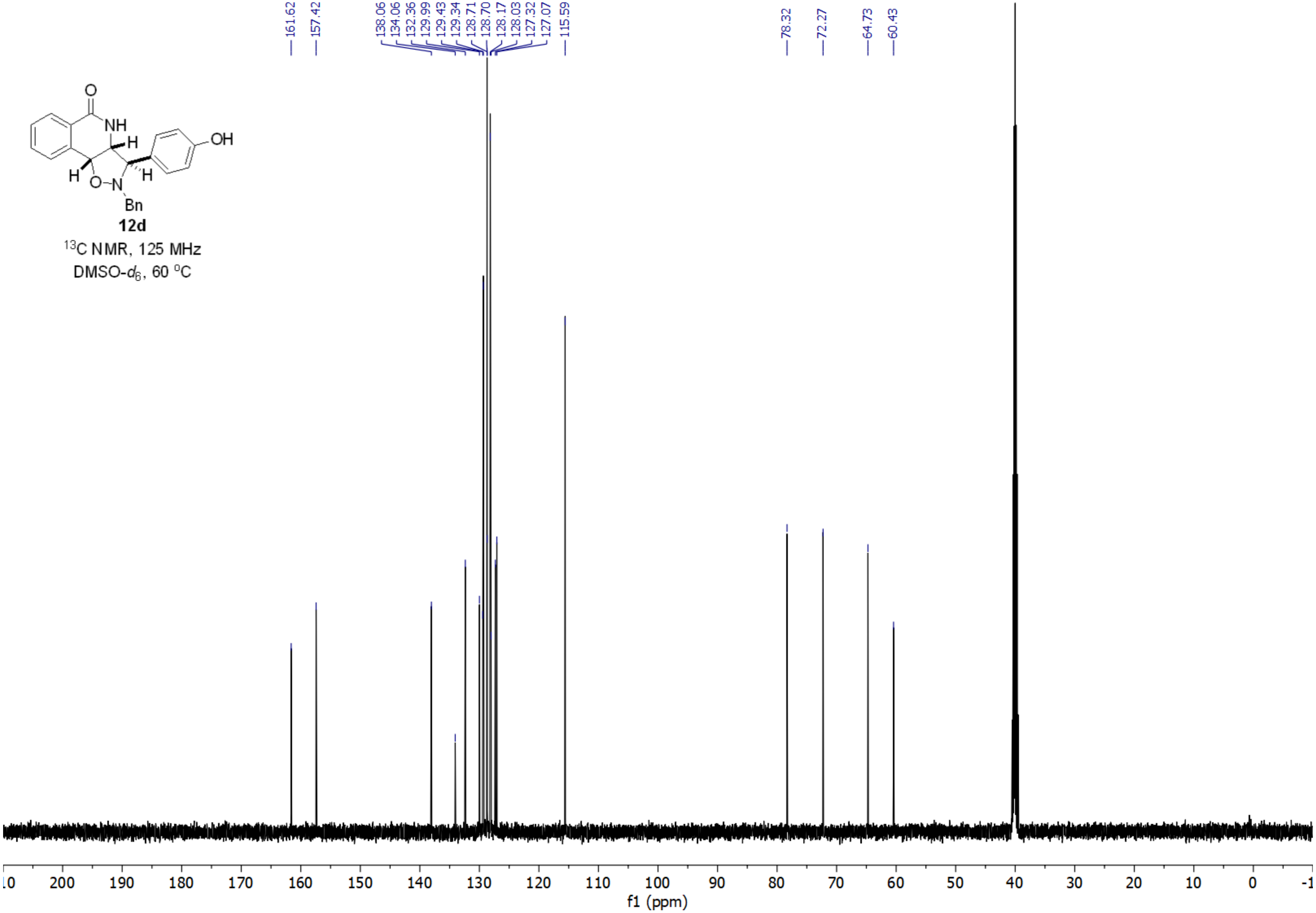

12d

^{13}C NMR, 125 MHz

DMSO-d_6, 60 ^{o}C

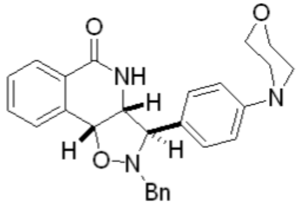

12e

^{1}H NMR, 500 MHz

DMSO-d_6, 60 °C

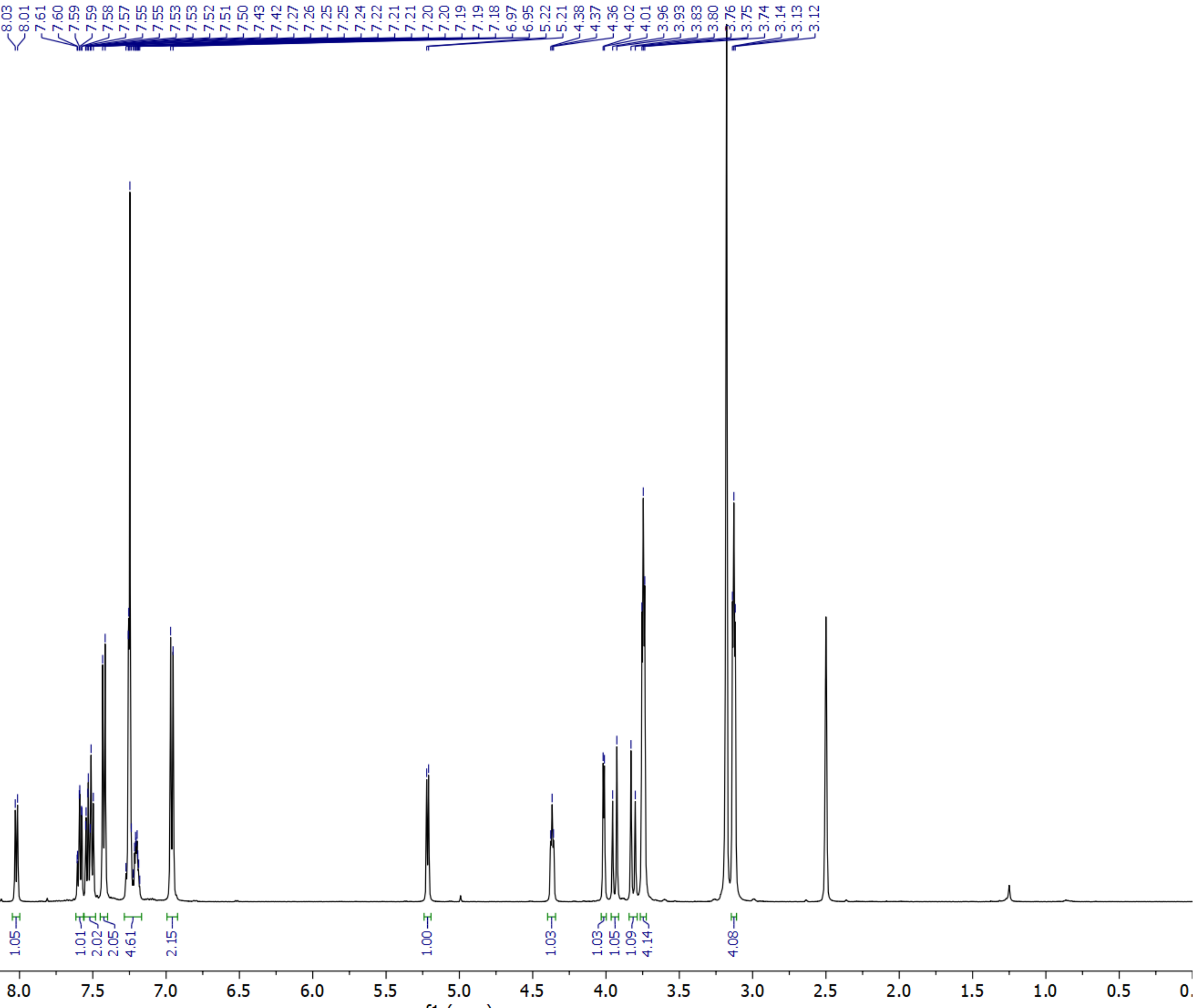

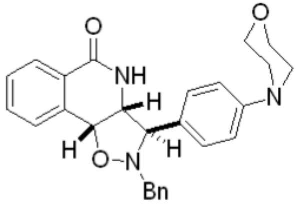

12e

^{13}C NMR, 125 MHz

DMSO-d_6, 60 °C

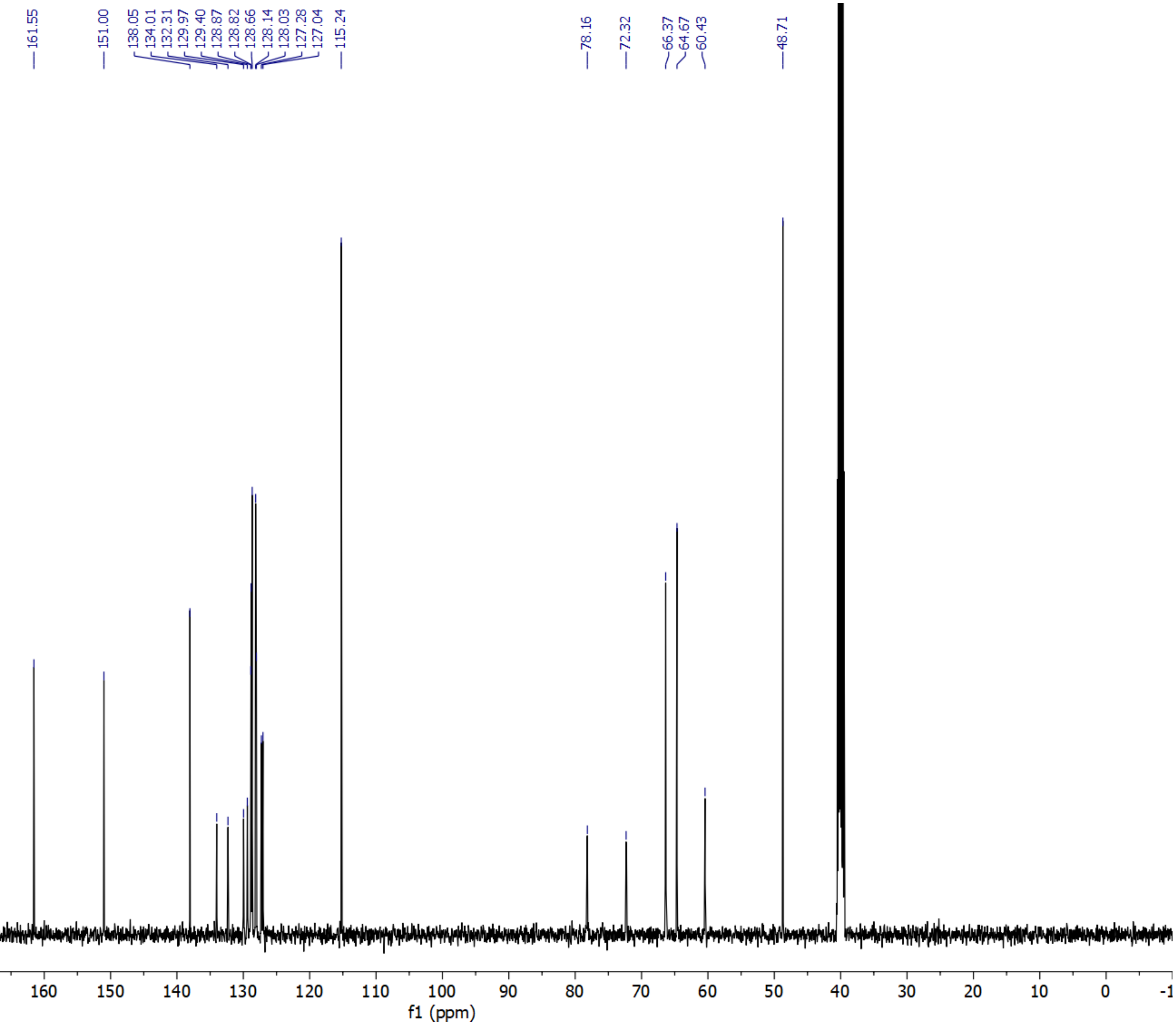

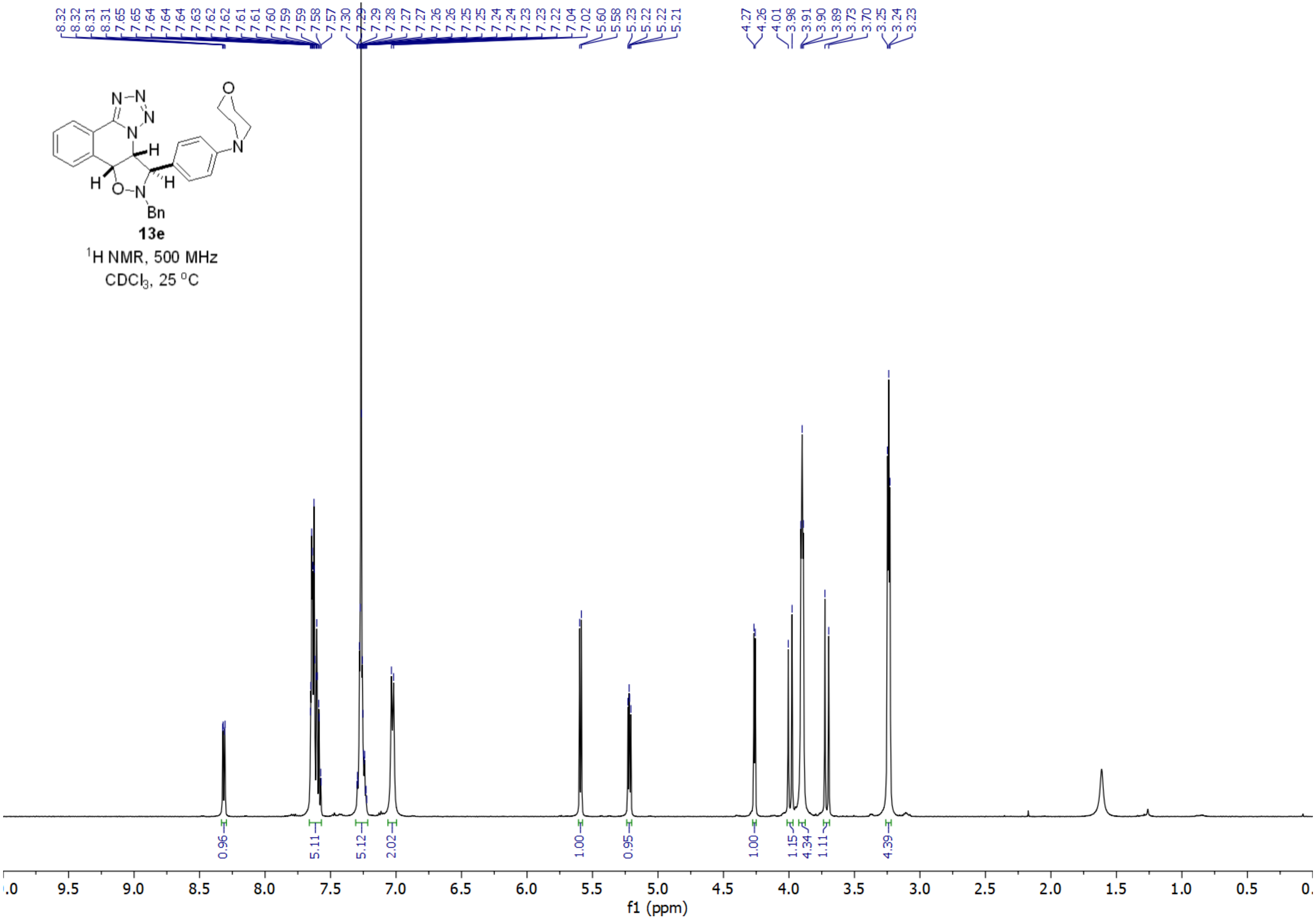

13e
1H NMR, 500 MHz
CDCl3, 25 °C
f1 (ppm)

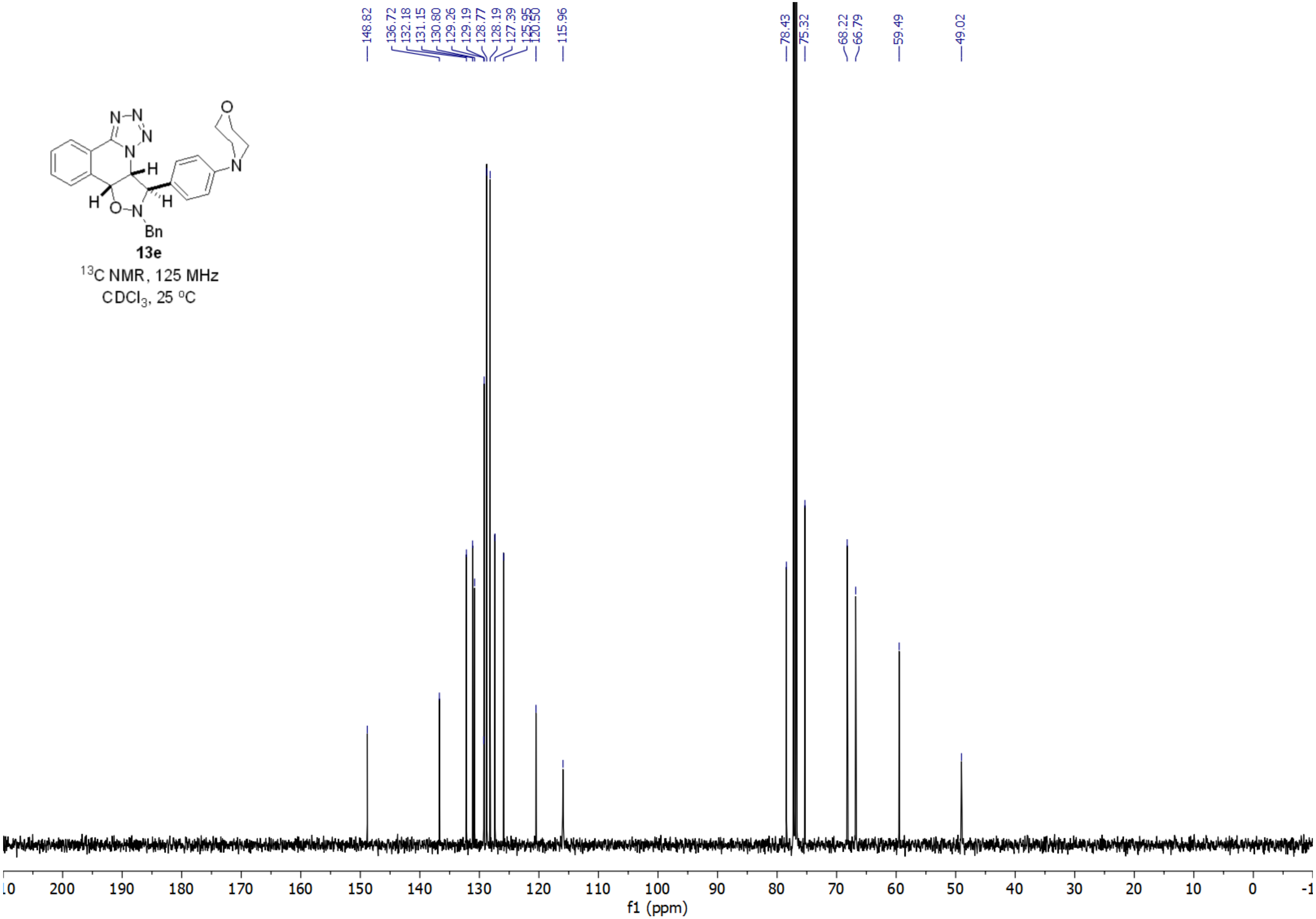

13e

^{13}C NMR, 125 MHz

$CDCl_3$, 25 °C

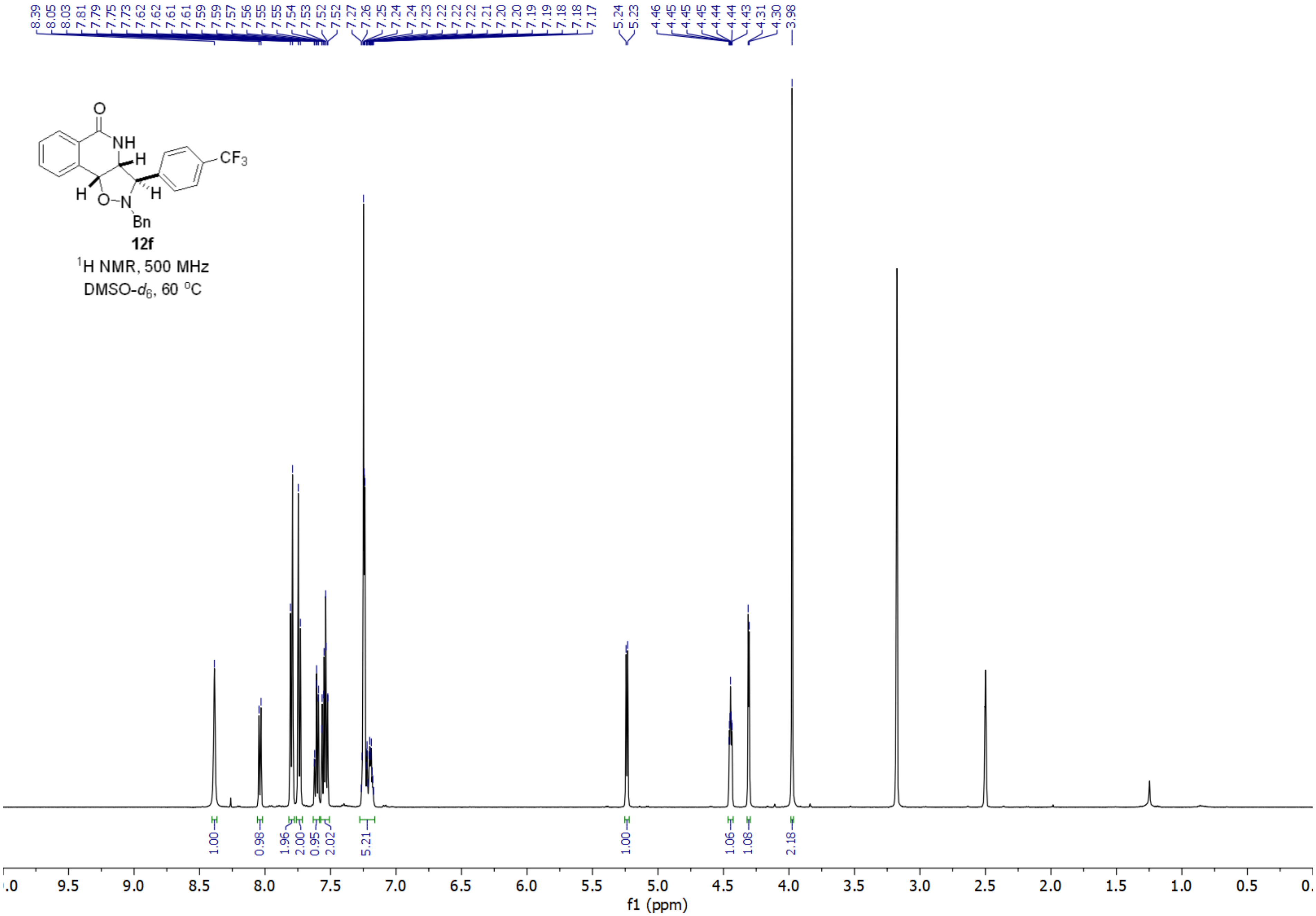

8.39
8.05
8.03
7.81
7.79
7.75
7.73
7.62
7.62
7.61
7.61
7.59
7.59
7.57
7.56
7.55
7.55
7.54
7.53
7.52
7.52
7.27
7.26
7.25
7.24
7.24
7.23
7.22
7.22
7.22
7.21
7.20
7.20
7.19
7.19
7.18
7.18
7.17
5.24
5.23
4.46
4.45
4.45
4.45
4.44
4.44
4.43
4.31
4.30
3.98
O
NH
H
CF3
H
O
N
H
Bn
12f
1H NMR, 500 MHz
DMSO-d6, 60 °C
1.00
0.98
1.96
2.00
0.95
2.02
5.21
1.00
1.06
1.08
2.18
9.5
9.0
8.5
8.0
7.5
7.0
6.5
6.0
5.5
5.0
4.5
4.0
3.5
3.0
2.5
2.0
1.5
1.0
0.5
f1 (ppm)

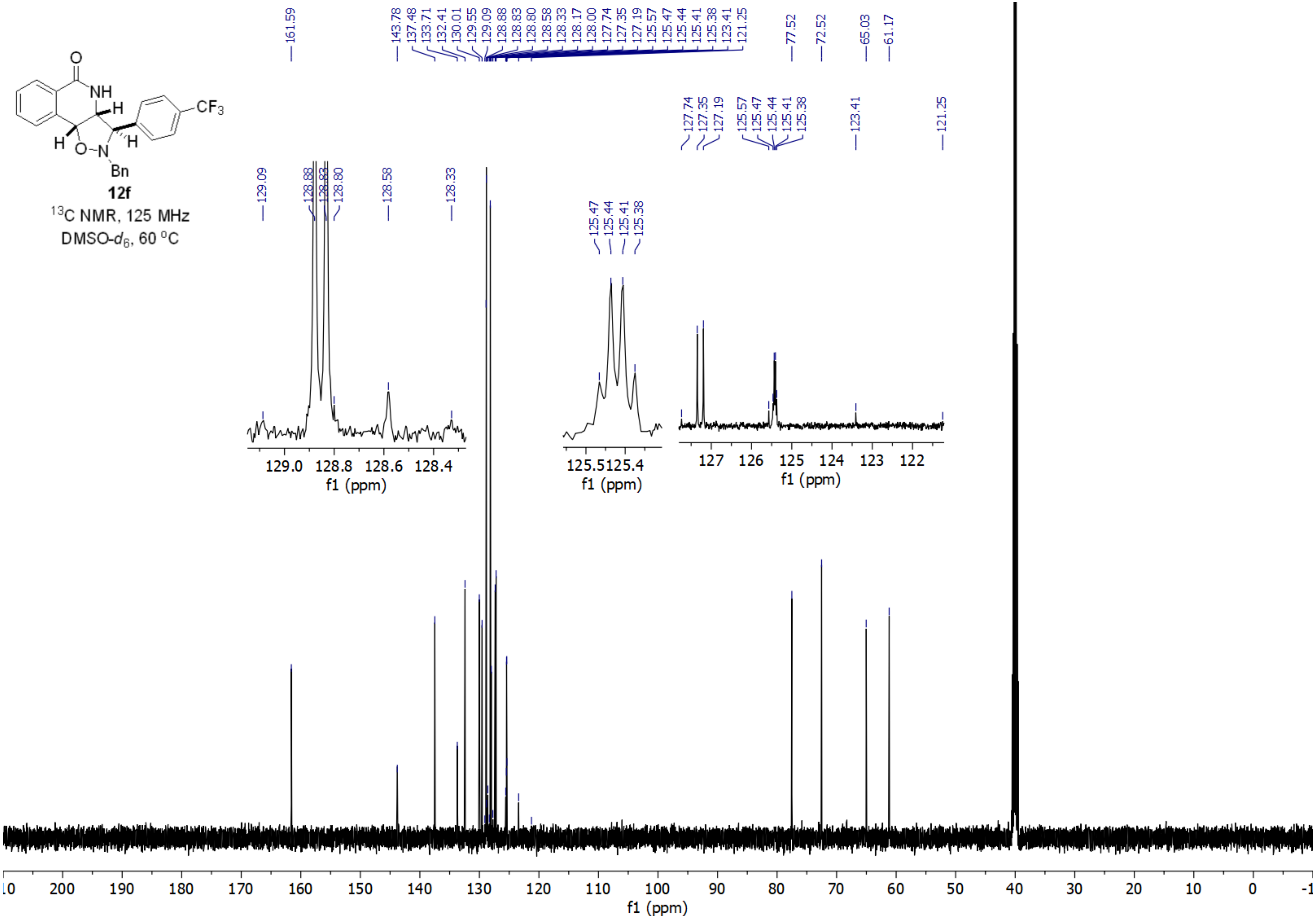

O
NH
H
CF3
H
H
O
N
Bn
12f
13C NMR, 125 MHz
DMSO-d6, 60 °C
161.59
143.78
137.48
133.71
132.41
130.01
129.55
129.09
128.88
128.83
128.80
128.58
128.33
128.17
128.00
127.74
127.35
127.19
125.57
125.47
125.44
125.41
125.38
123.41
121.25
77.52
72.52
65.03
61.17
129.09
128.88
128.83
128.80
128.58
128.33
129.0 128.8 128.6 128.4
f1 (ppm)
125.47
125.44
125.41
125.38
125.5125.4
f1 (ppm)
127.74
127.35
127.19
125.57
125.47
125.44
125.41
125.38
123.41
121.25
127 126 125 124 123 122
f1 (ppm)
10 200 190 180 170 160 150 140 130 120 110 100 90 80 70 60 50 40 30 20 10 0 -1
f1 (ppm)

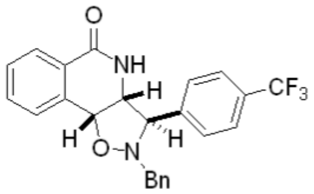

12f

^{19}F NMR, 470 MHz

DMSO-d_6, 25 °C

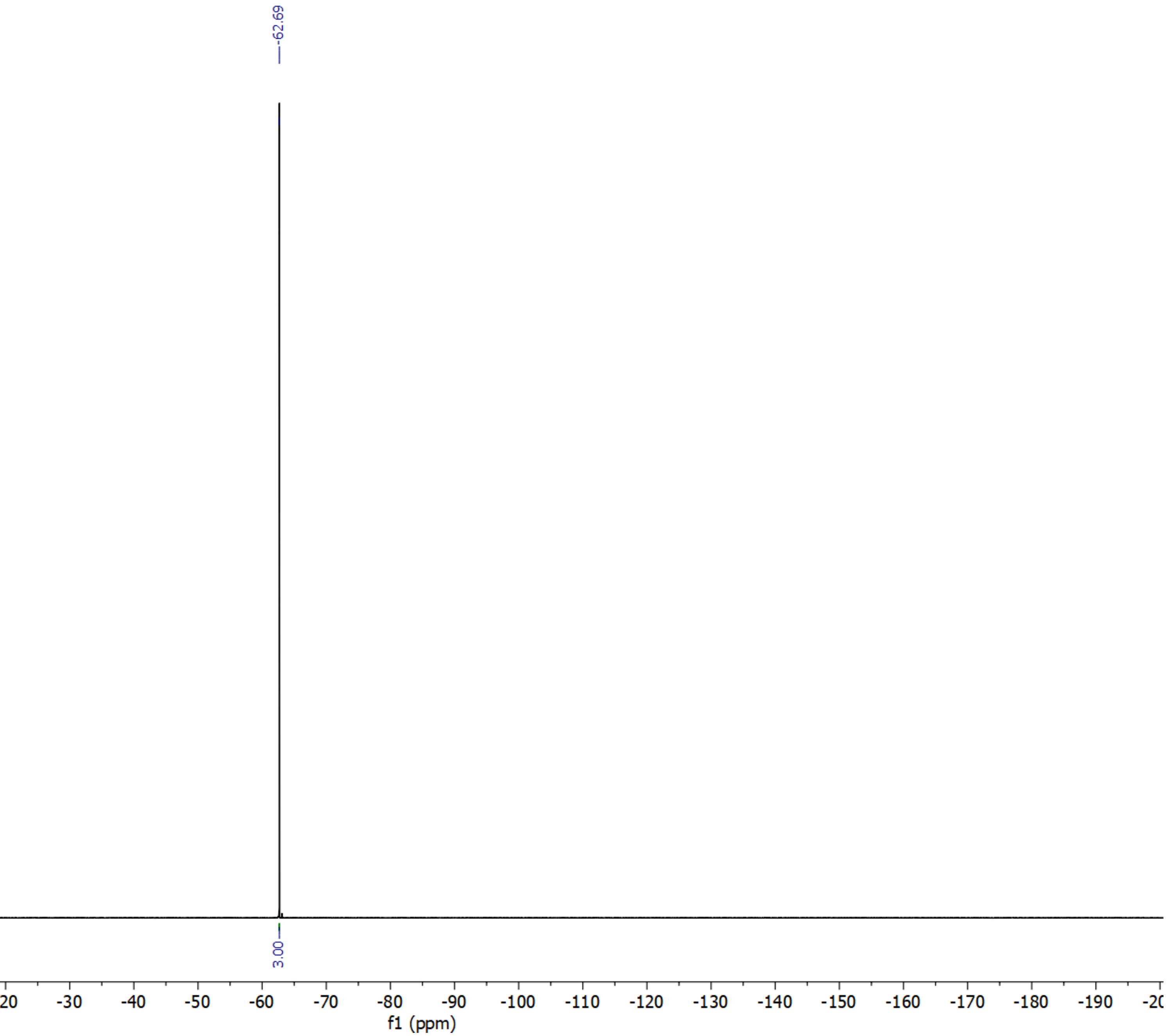

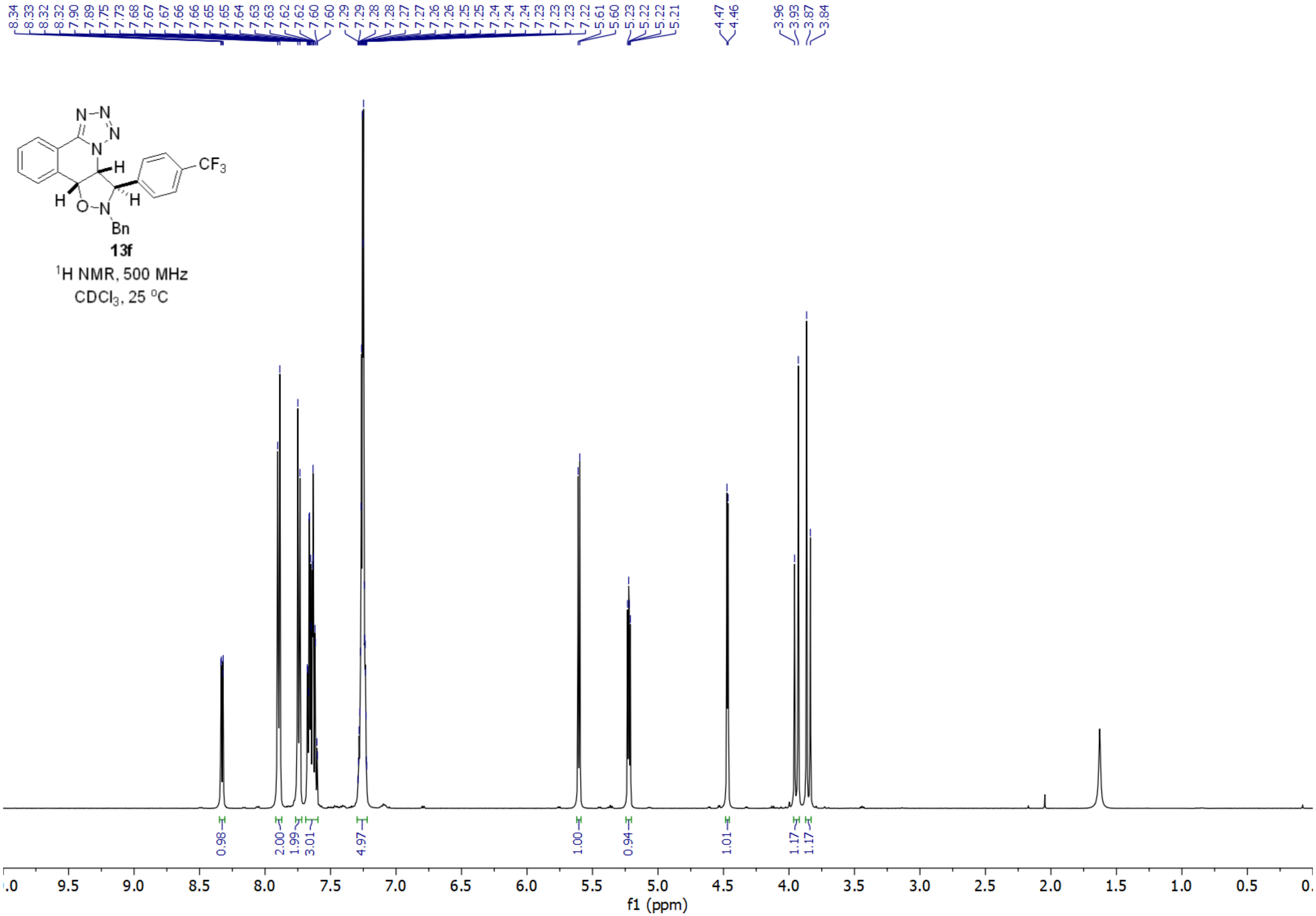

8.34
8.33
8.32
8.32
7.90
7.89
7.75
7.73
7.68
7.67
7.67
7.66
7.66
7.65
7.65
7.64
7.63
7.63
7.62
7.62
7.60
7.60
7.29
7.29
7.28
7.28
7.27
7.27
7.26
7.26
7.25
7.25
7.24
7.24
7.24
7.23
7.23
7.23
7.22
5.61
5.60
5.23
5.22
5.22
5.21
4.47
4.46
3.96
3.93
3.87
3.84
13f
^{1}H NMR, 500 MHz
$CDCl_3$, 25 °C
0.98
2.00
1.99
3.01
4.97
1.00
0.94
1.01
1.17
1.17
9.5
9.0
8.5
8.0
7.5
7.0
6.5
6.0
5.5
5.0
4.5
4.0
3.5
3.0
2.5
2.0
1.5
1.0
0.5
f1 (ppm)

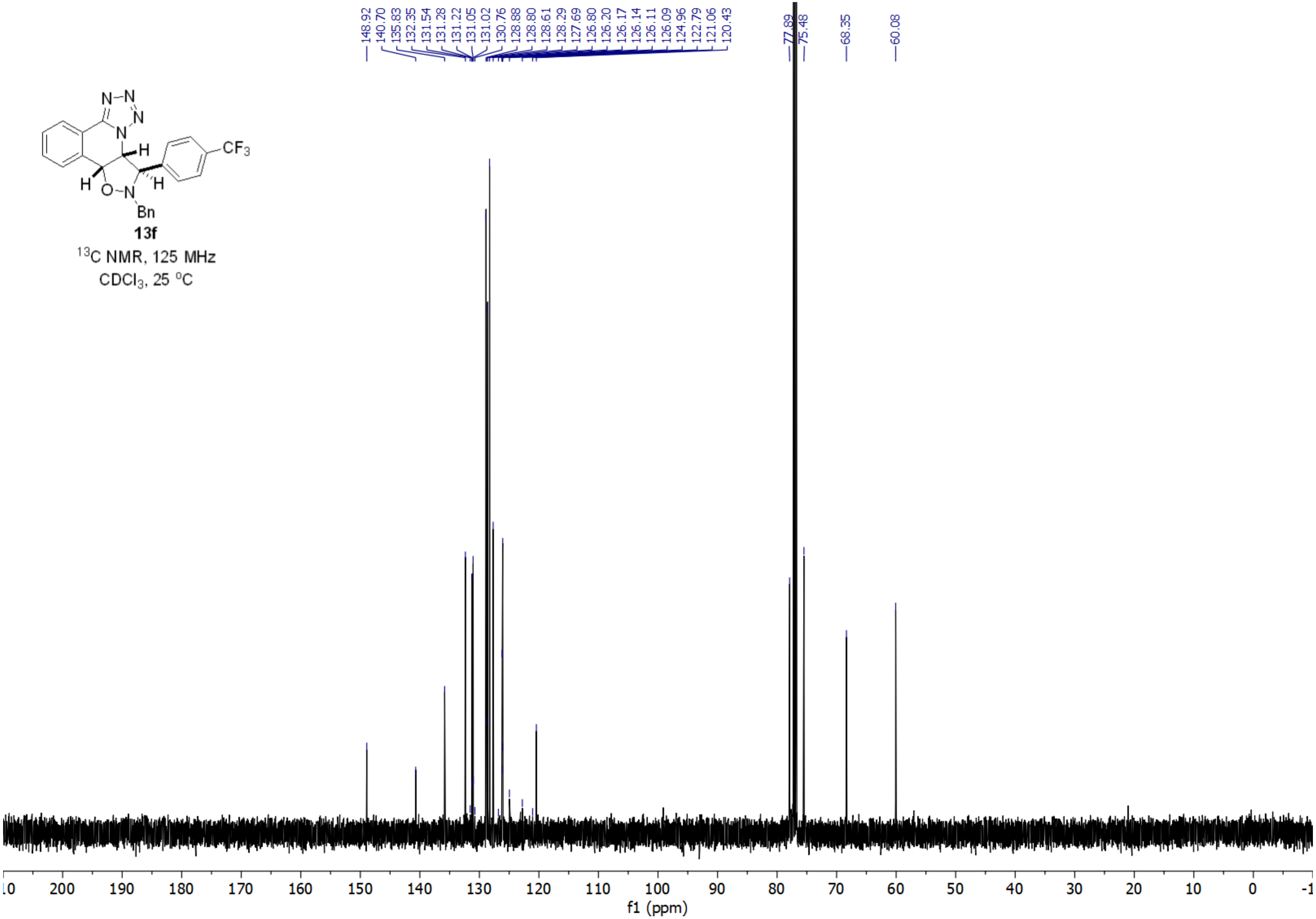
148.92
140.70
135.83
132.35
131.54
131.28
131.22
131.05
131.02
130.76
128.88
128.80
128.61
128.29
127.69
126.80
126.20
126.17
126.14
126.11
126.09
124.96
122.79
121.06
120.43
77.89
75.48
68.35
60.08
N
N
N
N
H
H
H
O
N
Bn
CF3
13f
13C NMR, 125 MHz
CDCl3, 25 °C
f1 (ppm)

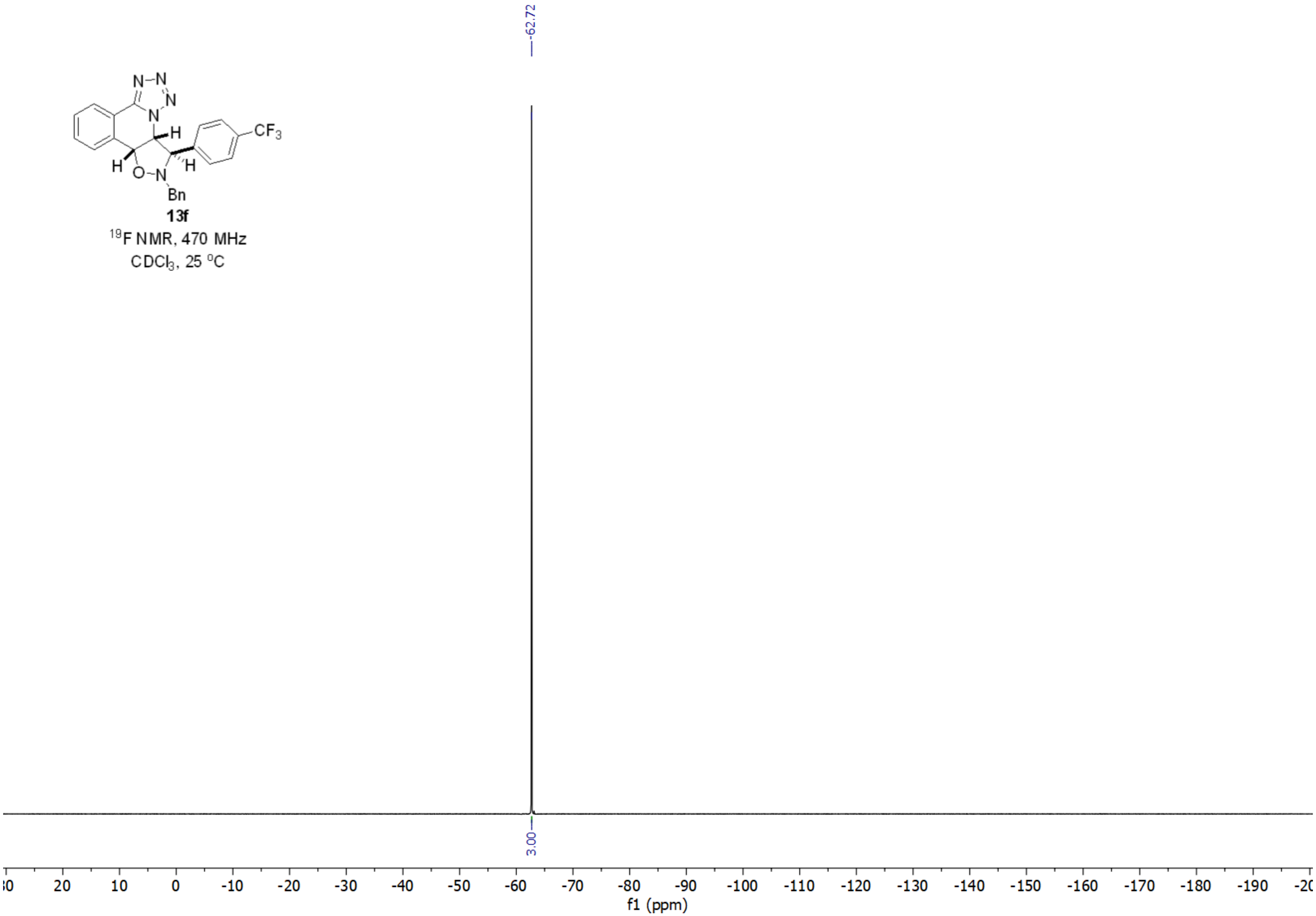

13f

^{19}F NMR, 470 MHz

$CDCl_3$, 25 ^{o}C

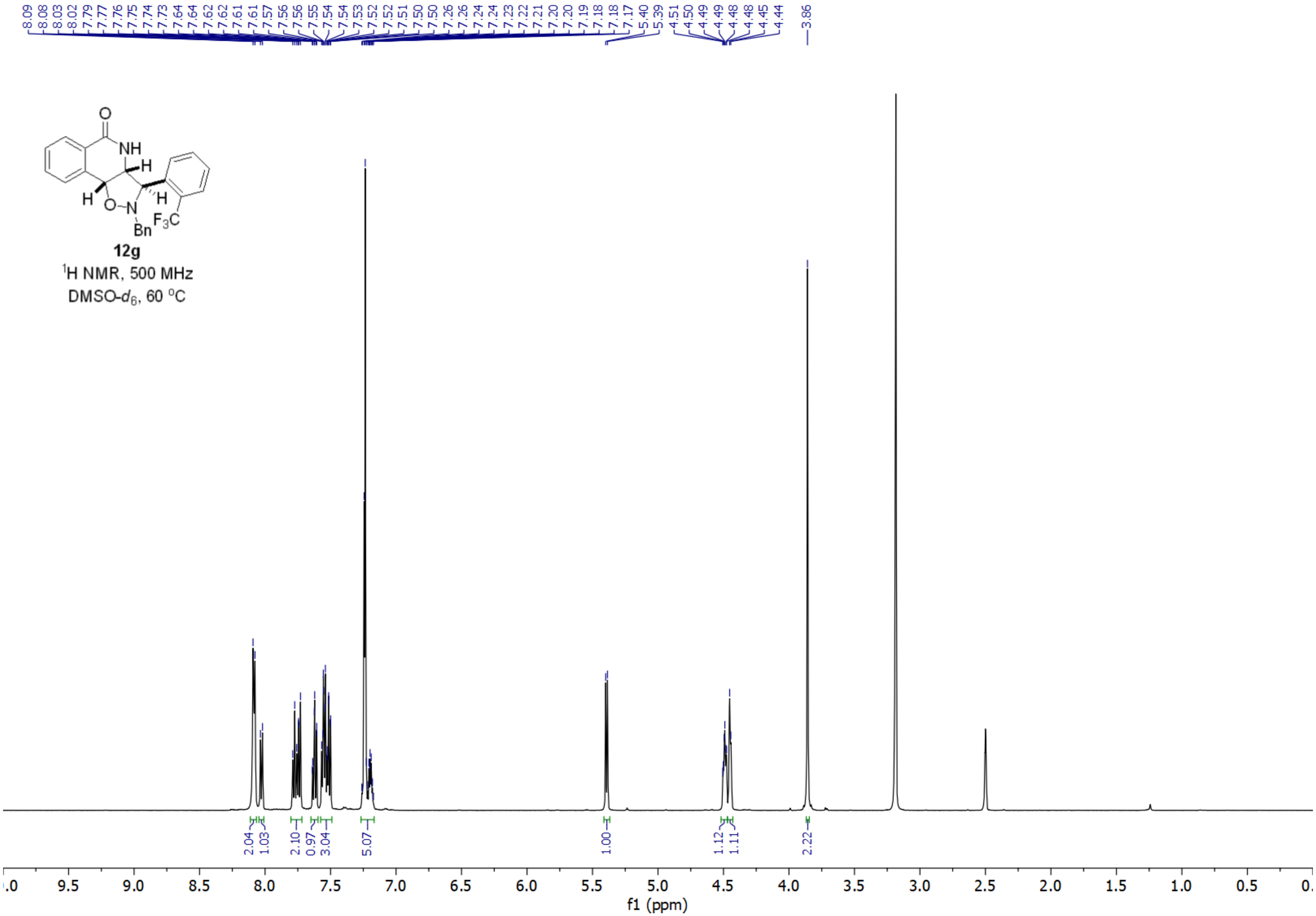
8.09 8.08 8.03 8.02 7.79 7.77 7.76 7.75 7.74 7.73 7.64 7.64 7.62 7.62 7.61 7.61 7.57 7.56 7.56 7.55 7.54 7.54 7.53 7.52 7.52 7.51 7.50 7.50 7.26 7.26 7.24 7.24 7.23 7.22 7.21 7.20 7.20 7.19 7.18 7.18 7.17 5.40 5.39 4.51 4.50 4.49 4.49 4.48 4.45 4.44 3.86
O
NH
H
H
H
O
N
Bn
F_3C
12g
^{1}H NMR, 500 MHz
DMSO-d_6, 60 °C
2.04
1.03
2.10
0.97
3.04
5.07
1.00
1.12
1.11
2.22
9.5 9.0 8.5 8.0 7.5 7.0 6.5 6.0 5.5 5.0 4.5 4.0 3.5 3.0 2.5 2.0 1.5 1.0 0.5
f1 (ppm)

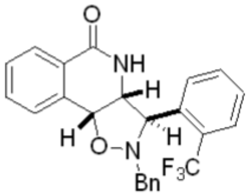

12g

^{13}C NMR, 125 MHz

DMSO-d_6, 60 °C

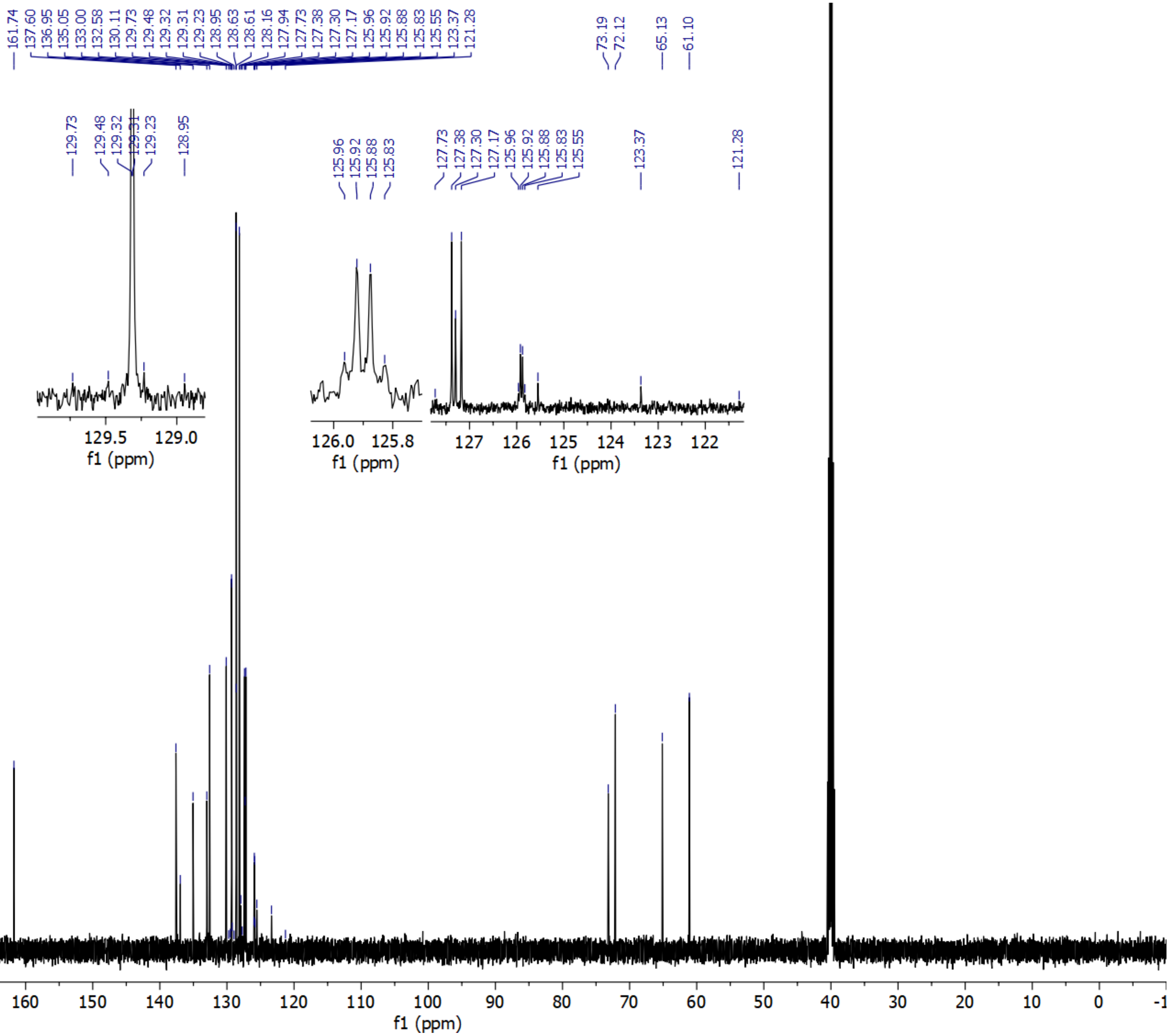

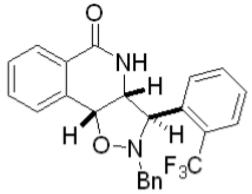

12g
^{19}F NMR, 470 MHz
DMSO-d_6, 25 °C

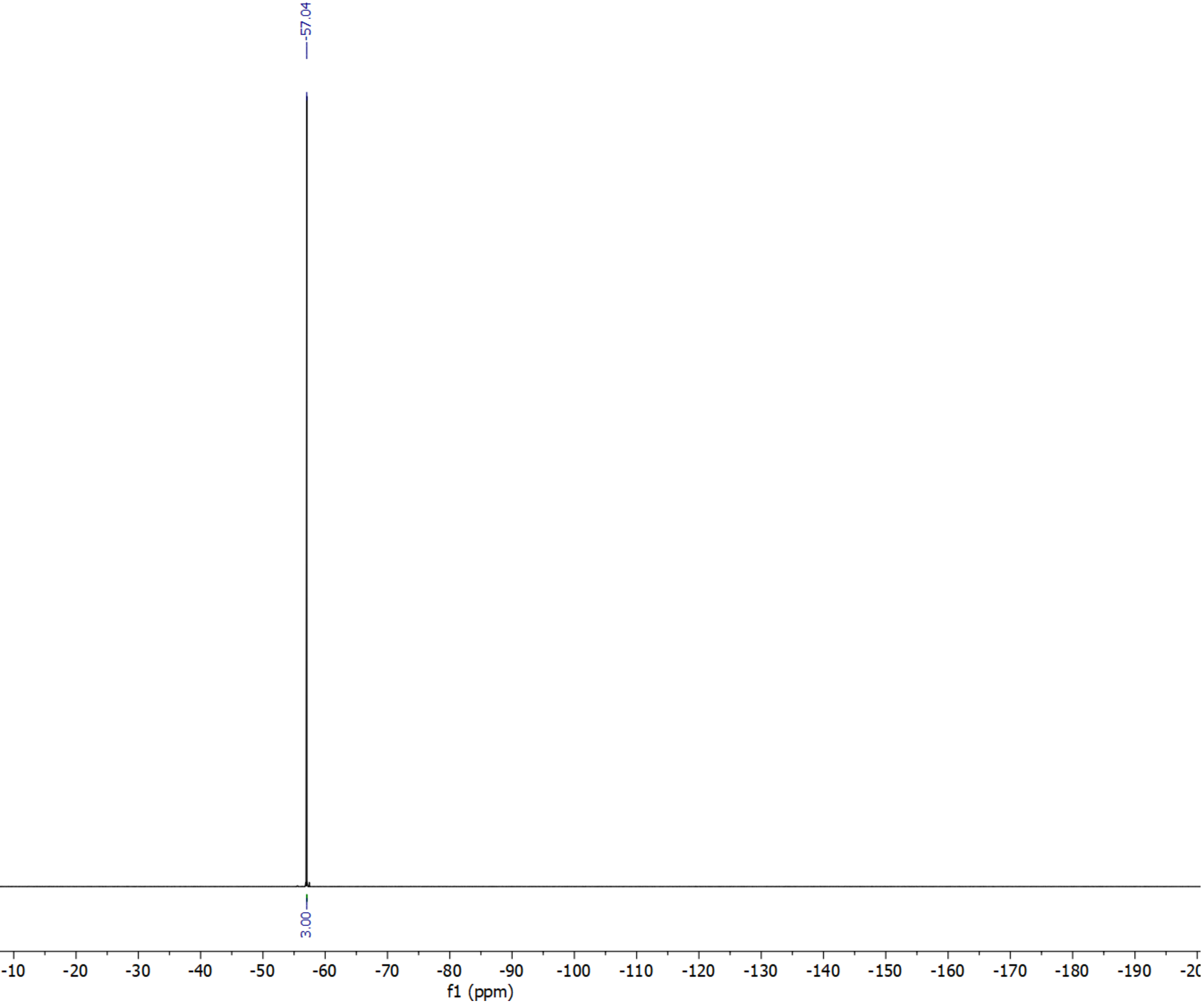

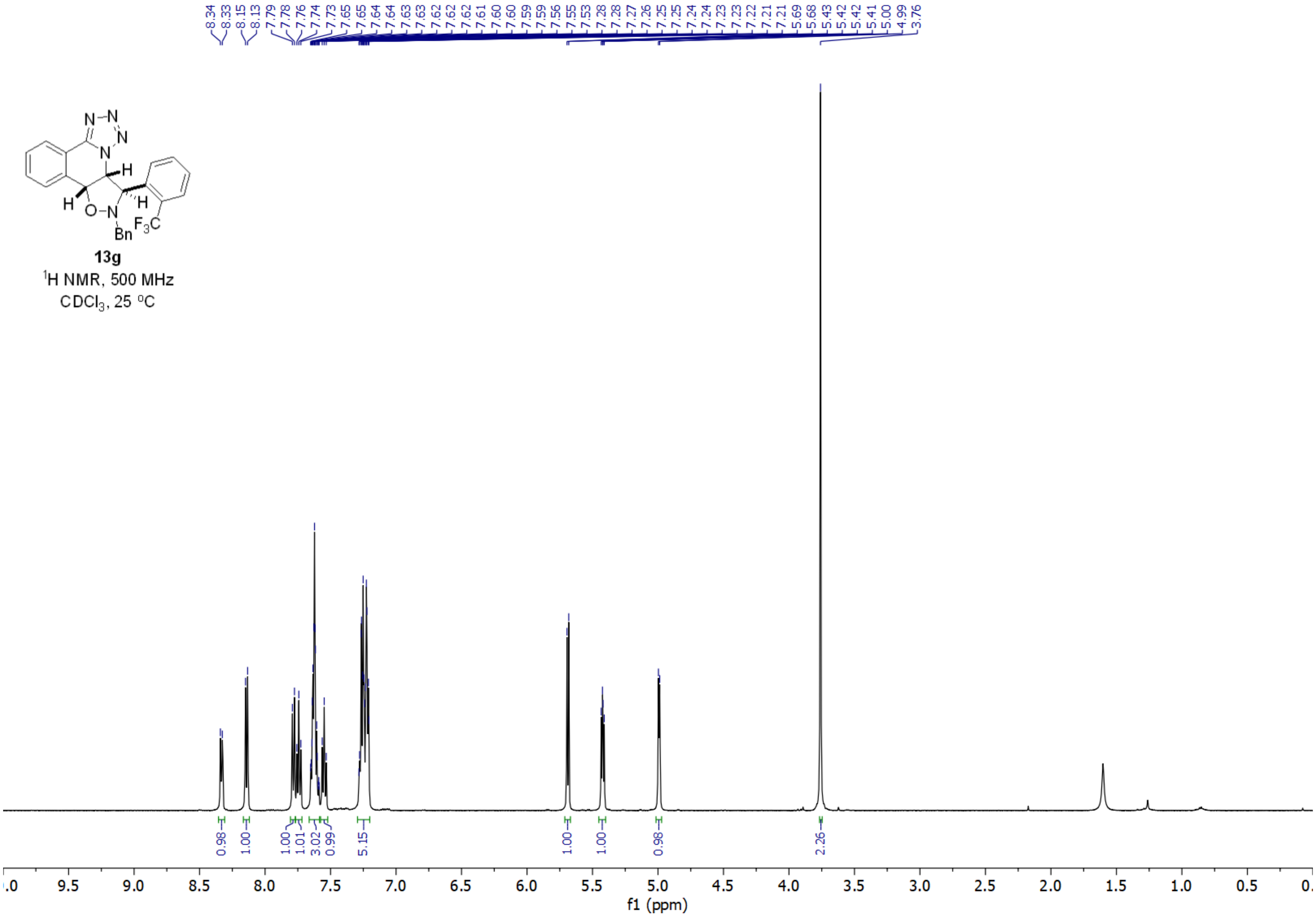

13g

^{1}H NMR, 500 MHz

$CDCl_3$, 25 °C

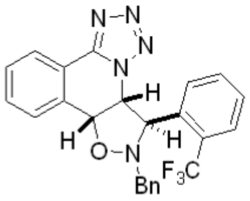

13g

^{13}C NMR, 125 MHz

$CDCl_3$, 25 °C

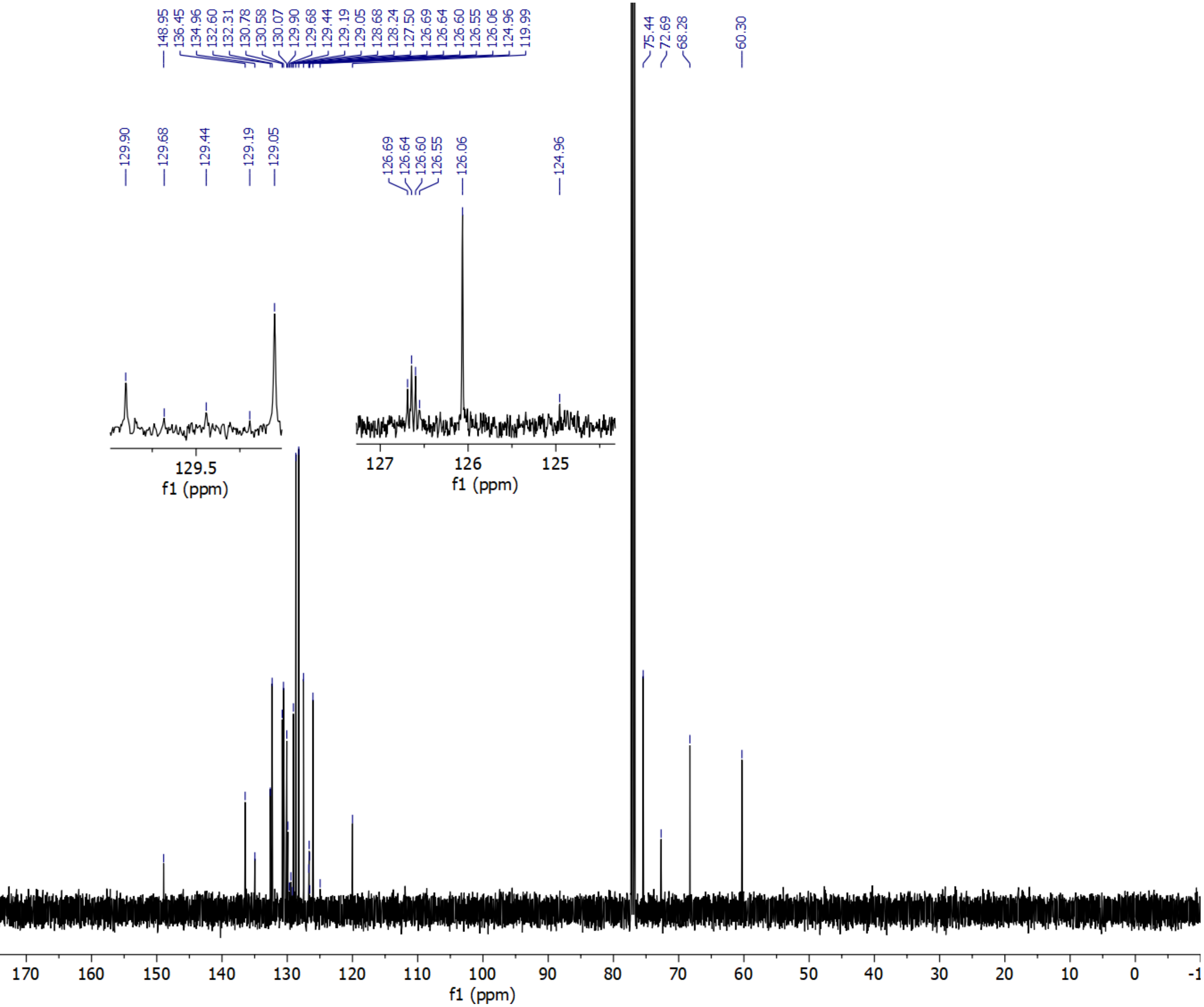

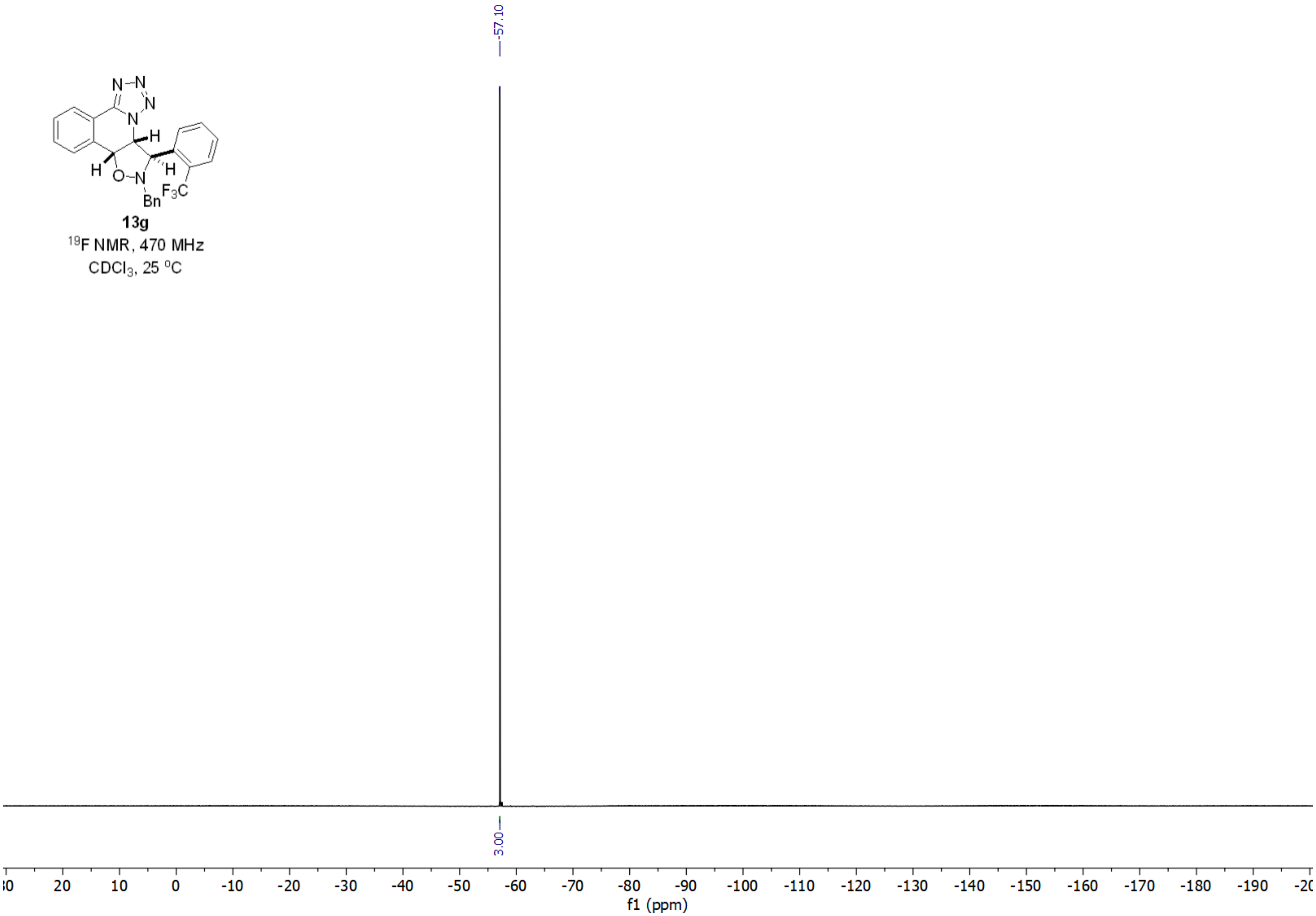
N–N
N
N
H
H
H
O–N
Bn
F3C
13g
19F NMR, 470 MHz
CDCl3, 25 °C
-57.10
3.00
30
20
10
0
-10
-20
-30
-40
-50
-60
-70
-80
-90
-100
-110
-120
-130
-140
-150
-160
-170
-180
-190
-200
f1 (ppm)

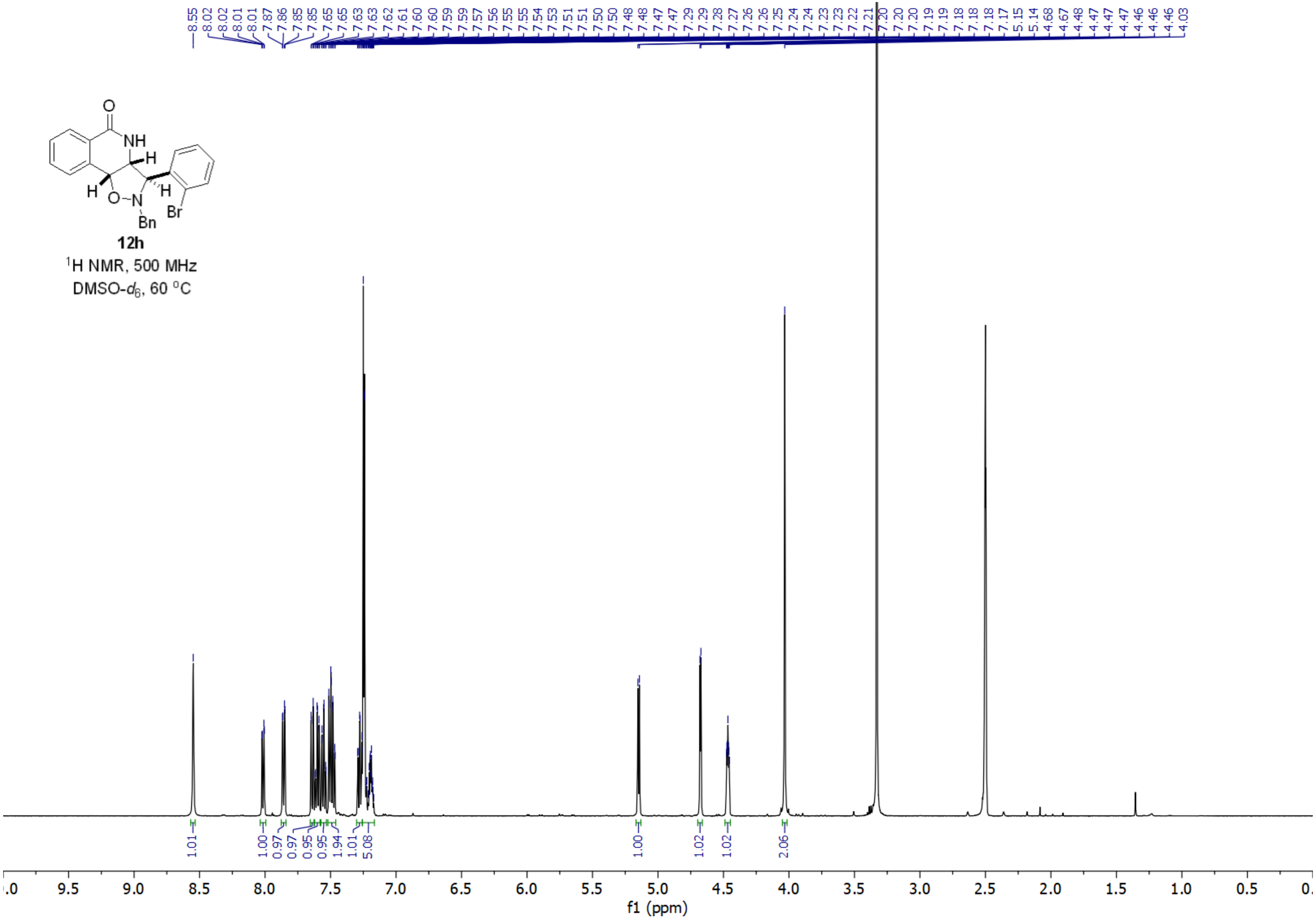
8.55
8.02
8.02
8.01
8.01
7.87
7.86
7.85
7.85
7.65
7.65
7.63
7.63
7.62
7.61
7.60
7.60
7.59
7.57
7.56
7.55
7.55
7.54
7.53
7.51
7.51
7.50
7.50
7.48
7.48
7.47
7.47
7.29
7.29
7.28
7.27
7.26
7.25
7.24
7.24
7.23
7.23
7.22
7.21
7.20
7.20
7.20
7.19
7.19
7.18
7.18
7.17
5.15
5.14
4.68
4.67
4.48
4.47
4.47
4.46
4.46
4.03
O
NH
H
H
H
O
N
Bn
Br
12h
^{1}H NMR, 500 MHz
DMSO-d_6, 60 °C
1.01
1.00
0.97
0.97
0.95
0.95
1.94
1.01
5.08
1.00
1.02
1.02
2.06
9.5
9.0
8.5
8.0
7.5
7.0
6.5
6.0
5.5
5.0
4.5
4.0
3.5
3.0
2.5
2.0
1.5
1.0
0.5
f1 (ppm)

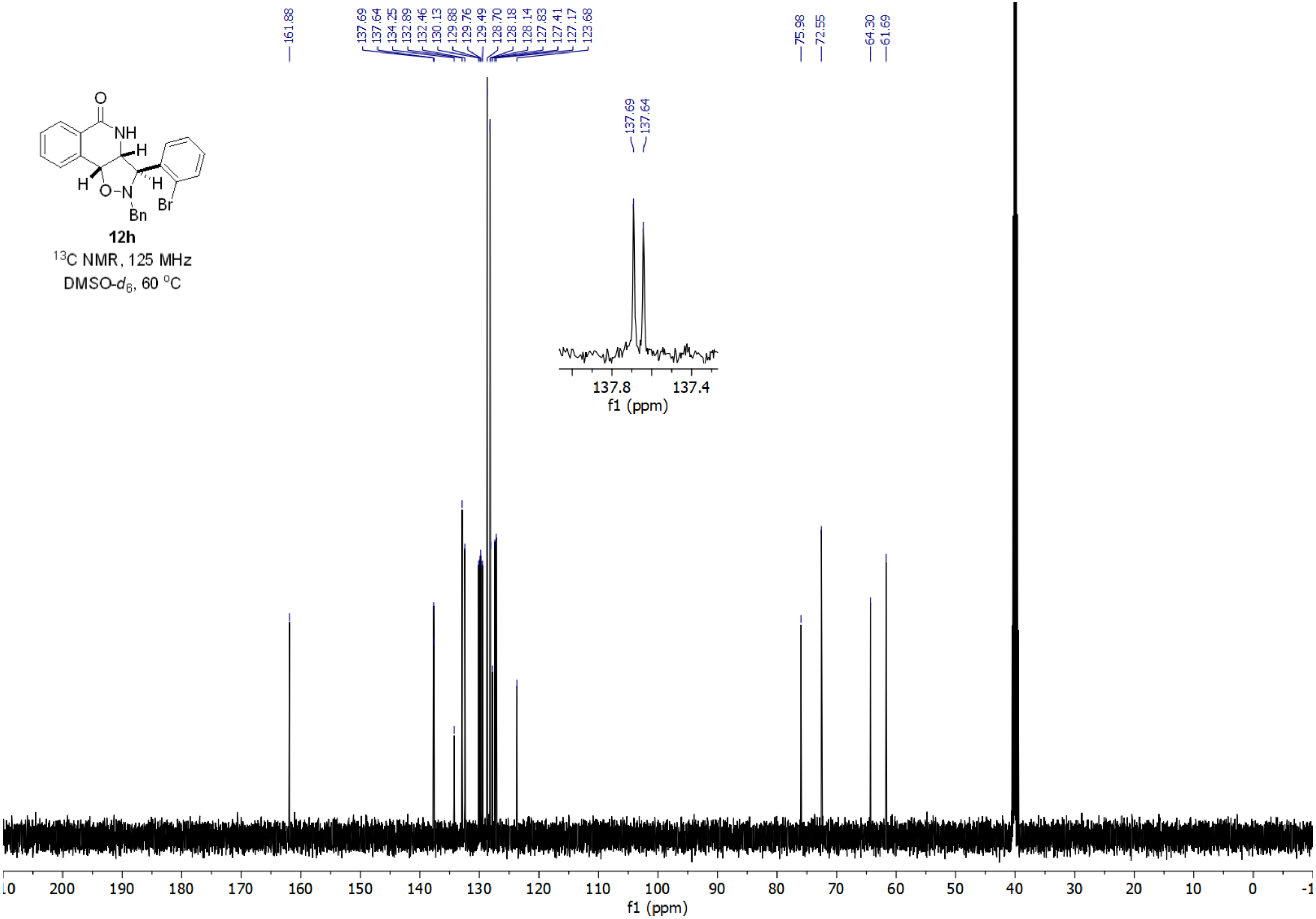

12h

^{13}C NMR, 125 MHz

DMSO-d_6, 60 ^{o}C

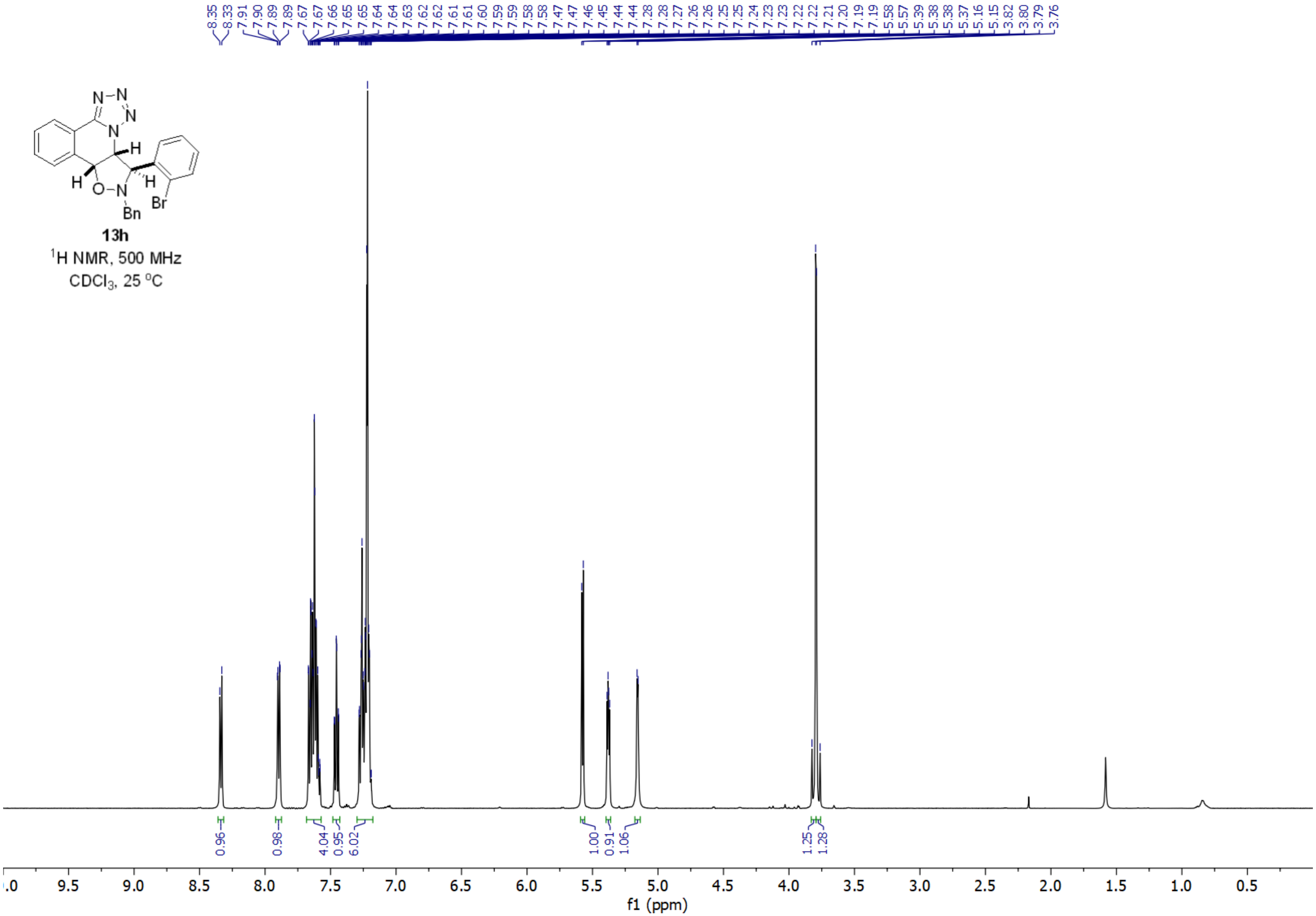

13h

^{1}H NMR, 500 MHz

$CDCl_3$, 25 °C

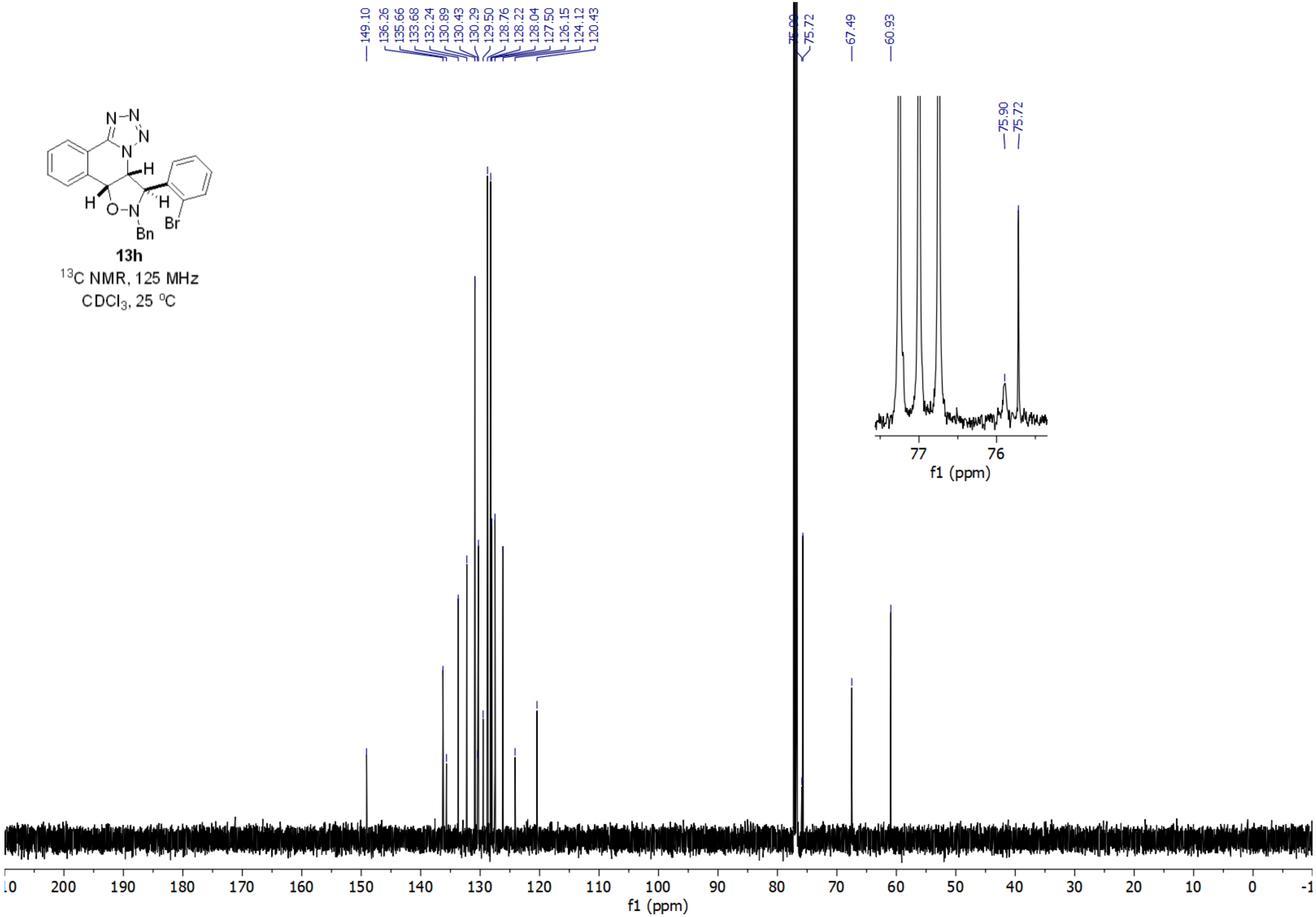

13h

^{13}C NMR, 125 MHz

$CDCl_3$, 25 °C

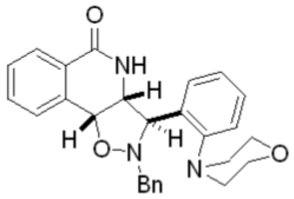

12i

^{1}H NMR, 500 MHz

DMSO-d_6, 60 ^{o}C

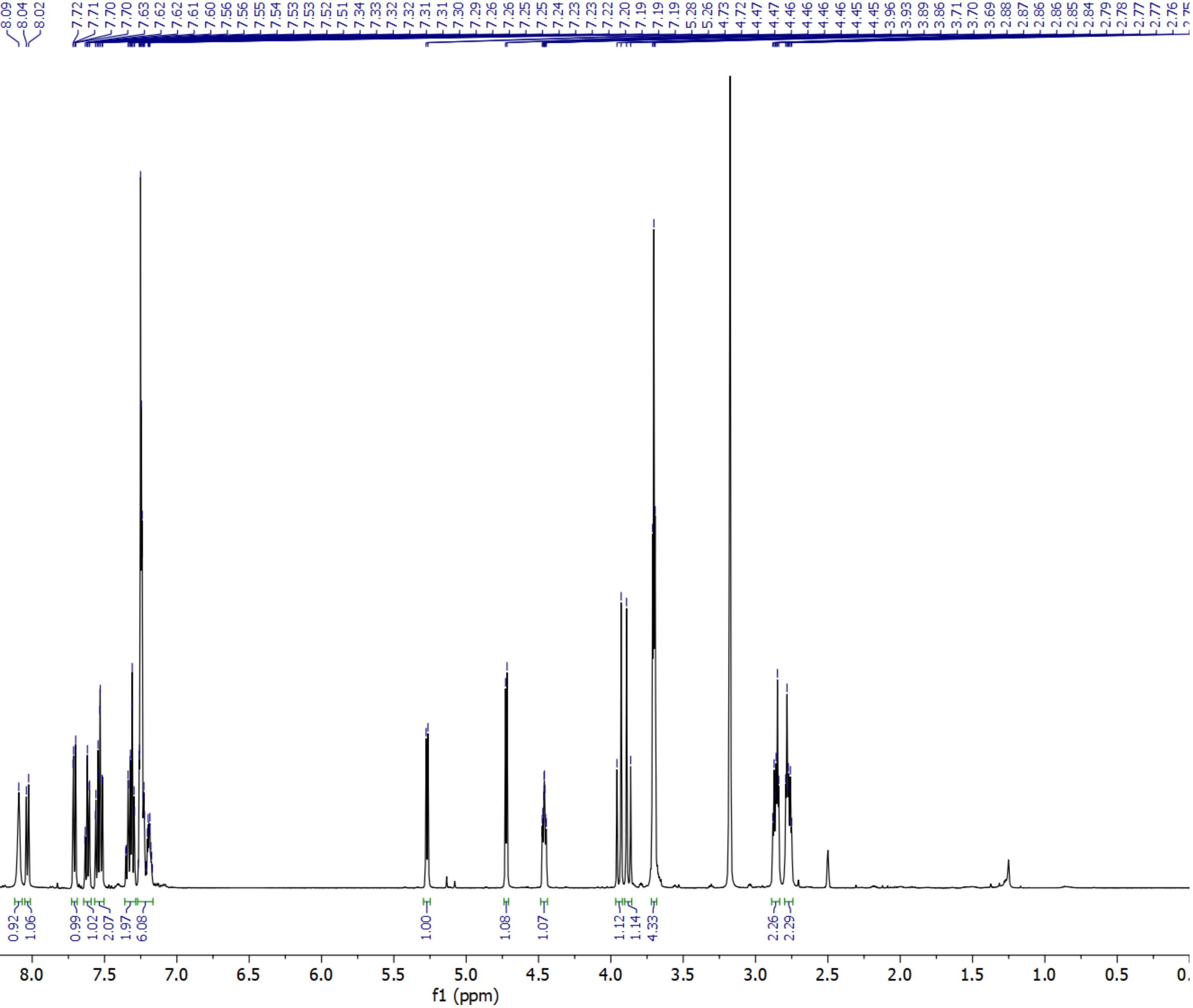

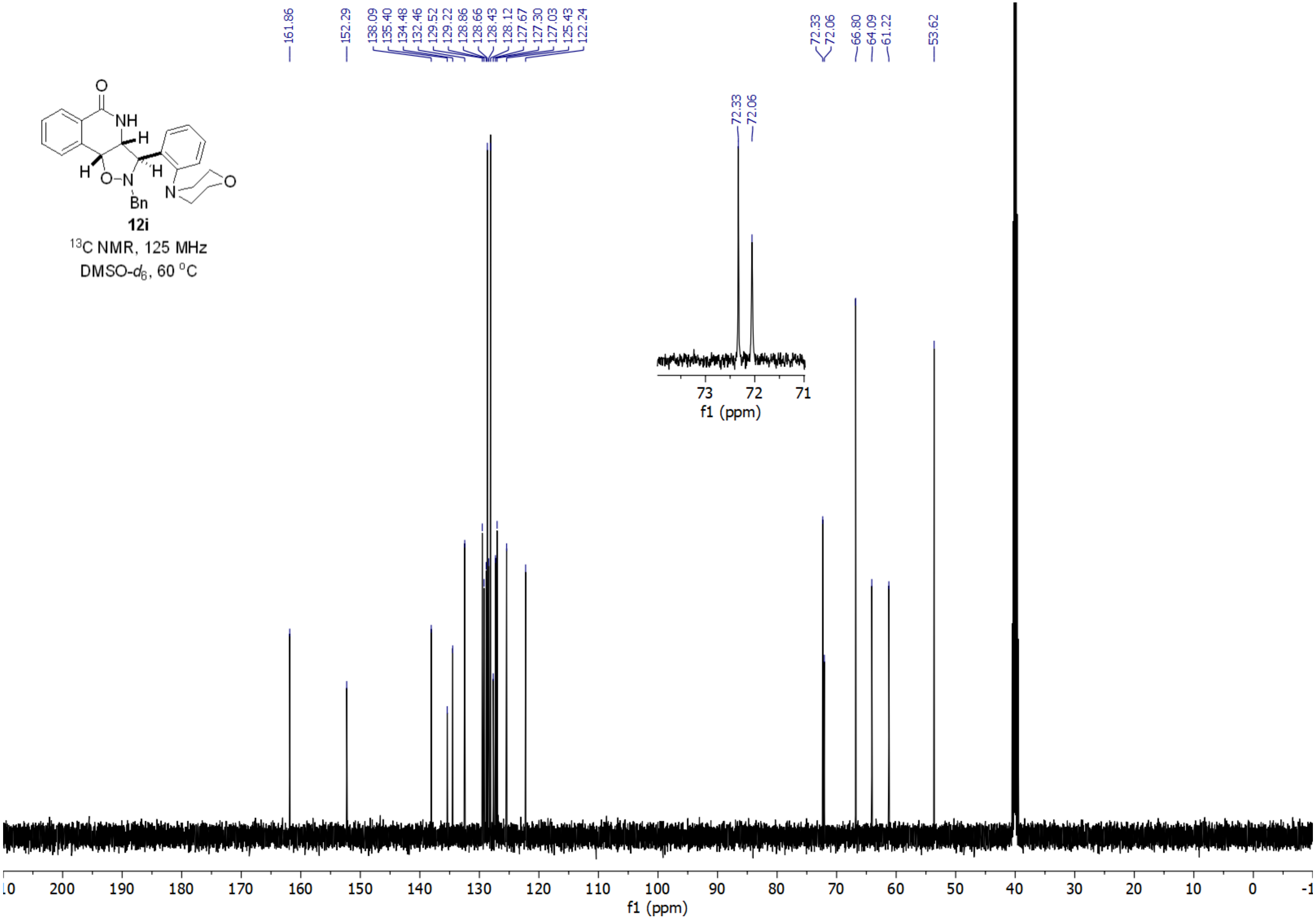
12i
^{13}C NMR, 125 MHz
DMSO-d_6, 60 °C
161.86
152.29
138.09
135.40
134.48
132.46
129.52
129.22
128.86
128.66
128.43
128.12
127.67
127.30
127.03
125.43
122.24
72.33
72.06
66.80
64.09
61.22
53.62
f1 (ppm)

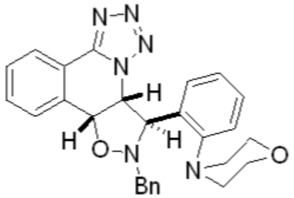

13i

^{1}H NMR, 500 MHz

$CDCl_3$, 25 °C

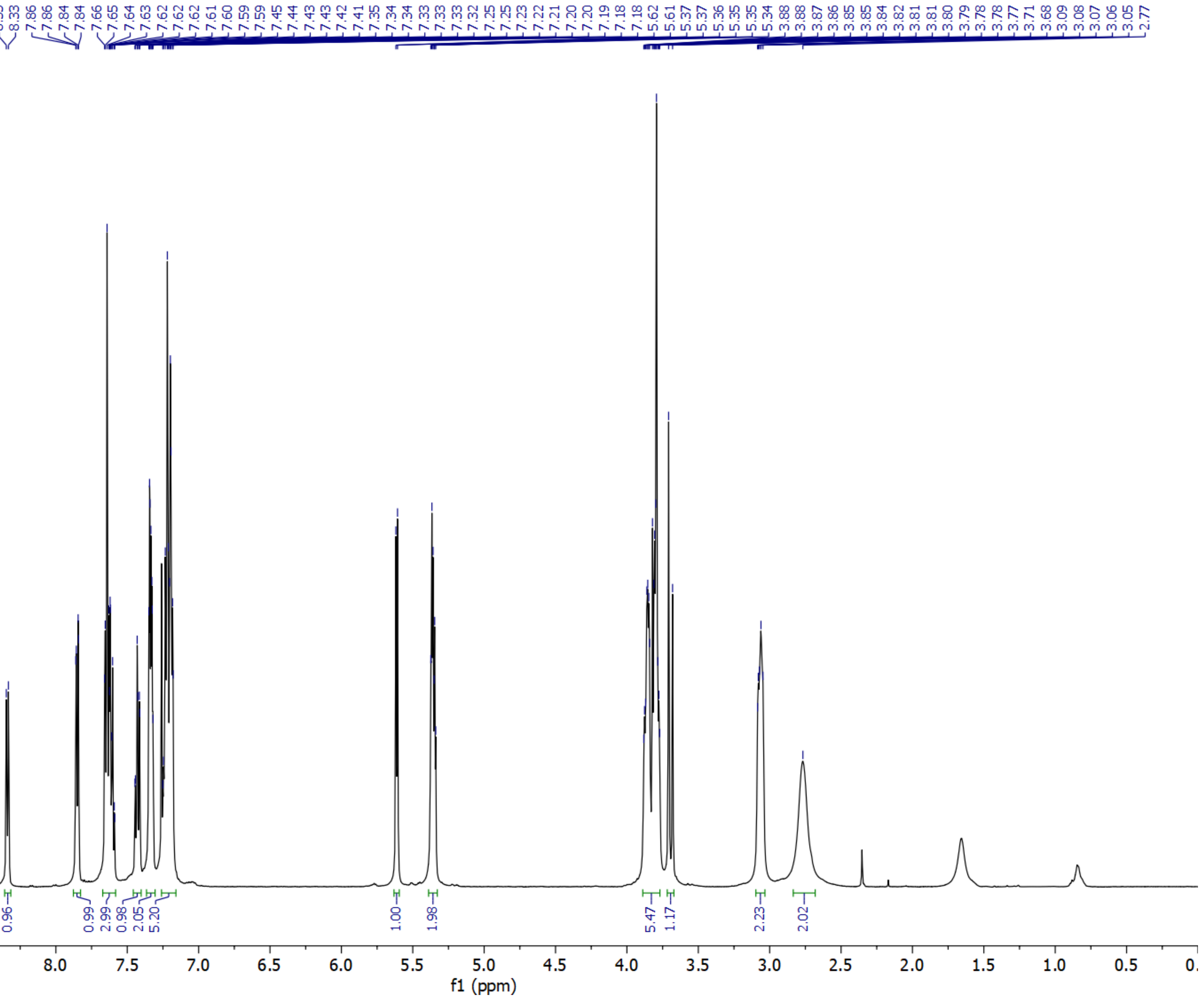

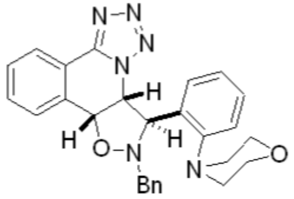

13i

^{13}C NMR, 125 MHz

$CDCl_3$, 25 °C

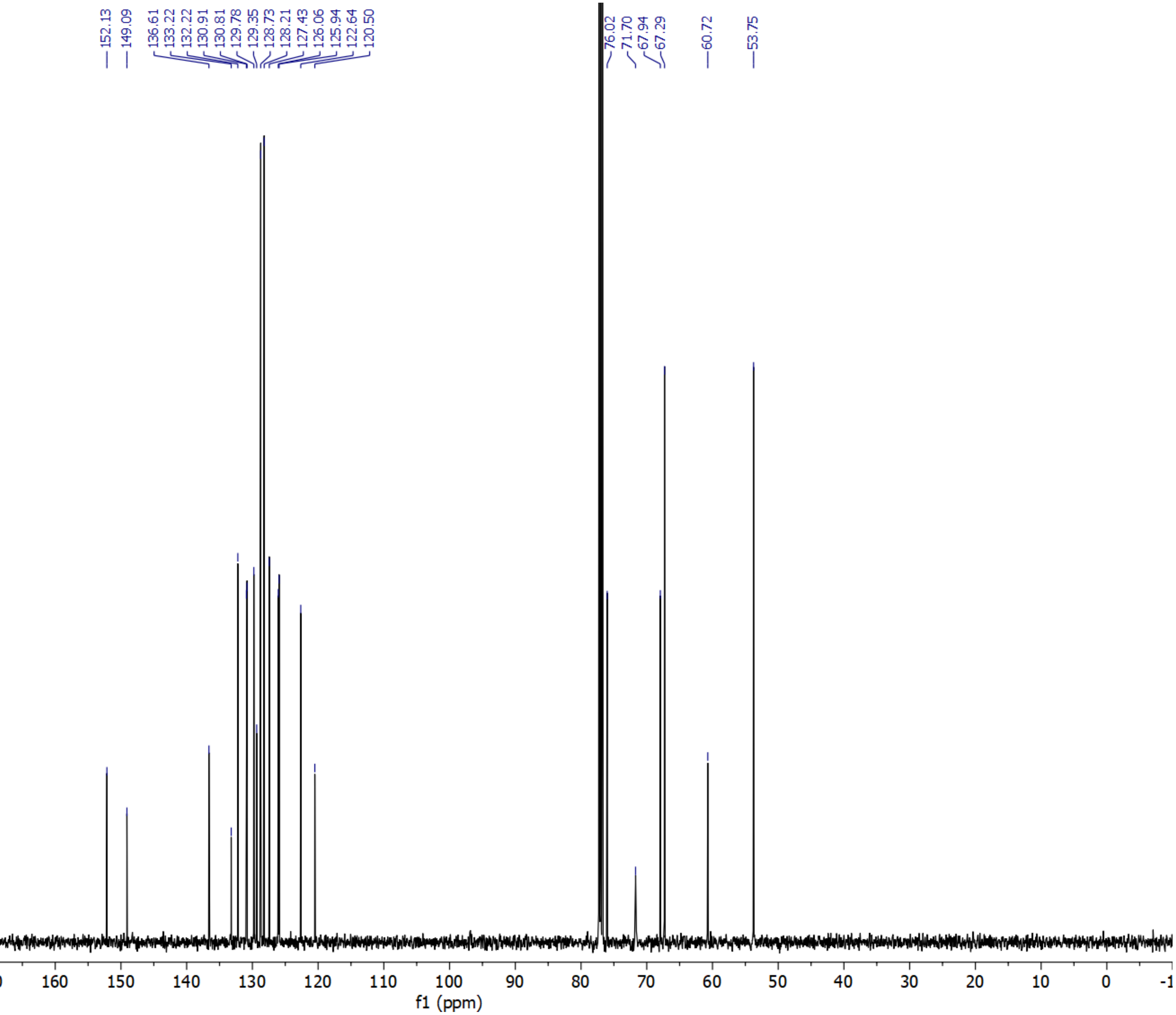

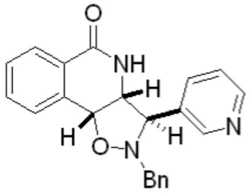

12j

^{1}H NMR, 500 MHz

DMSO-d_6, 60 °C

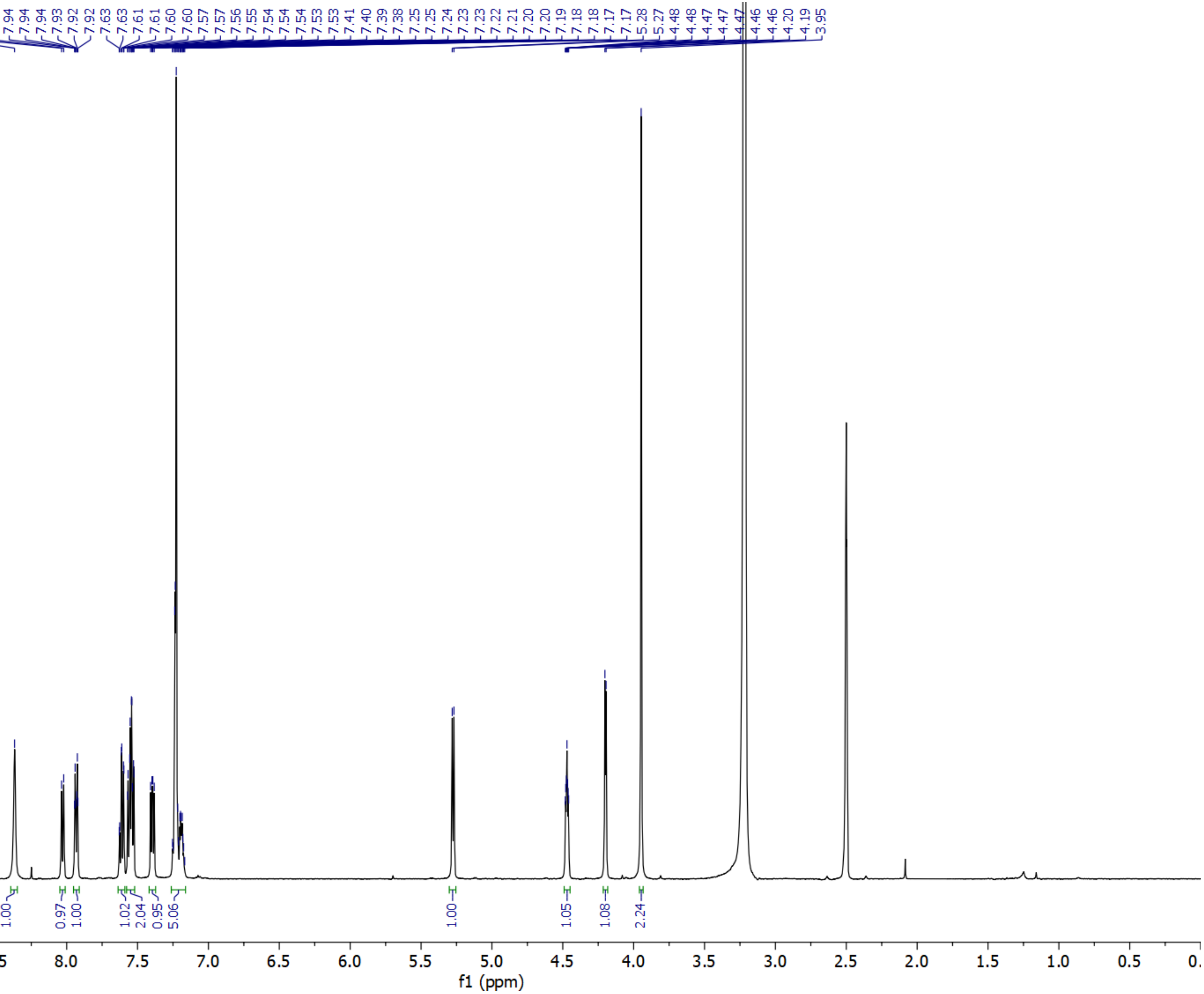

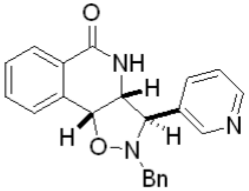

12j

^{13}C NMR, 125 MHz

DMSO-d_6, 60 °C

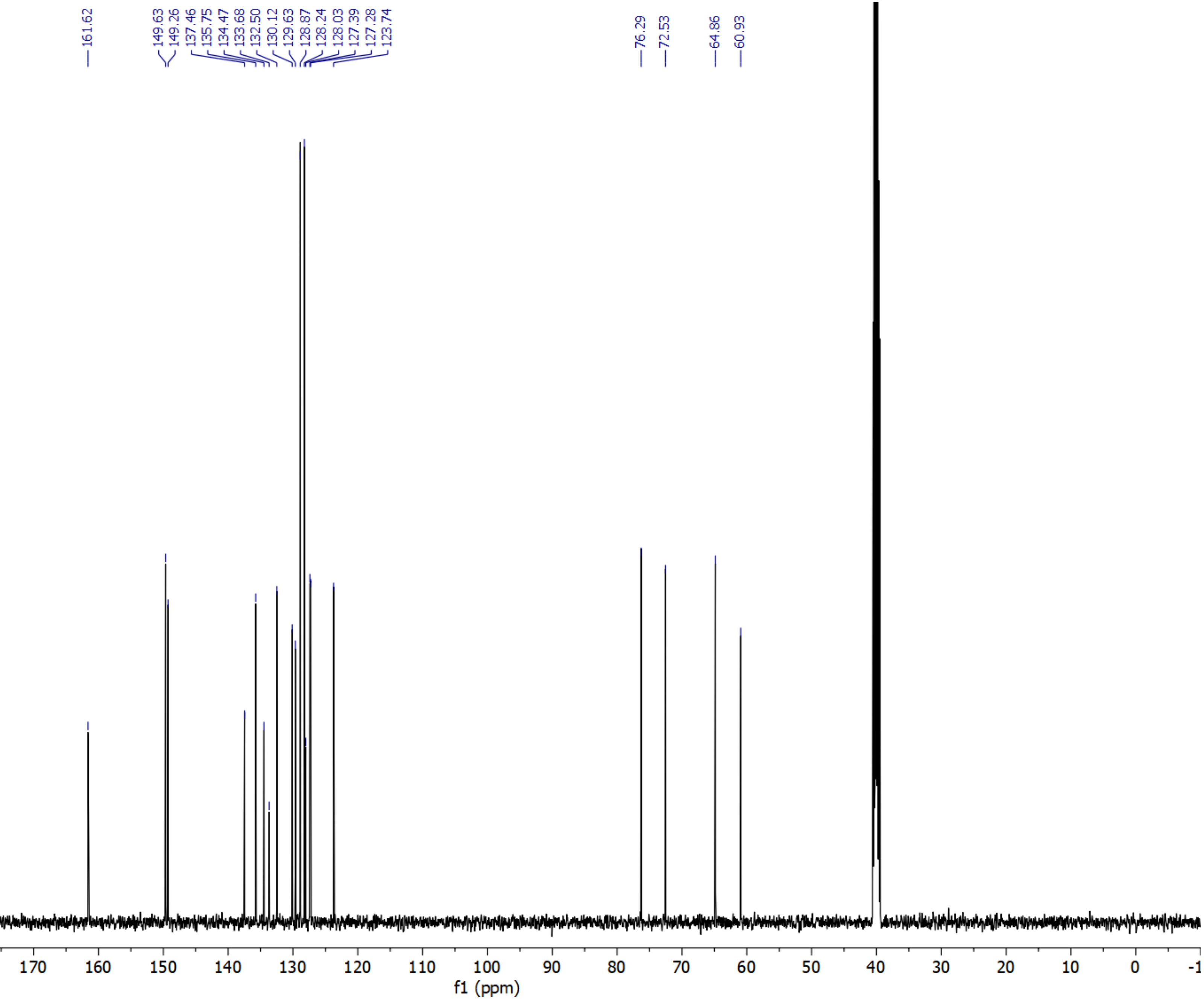

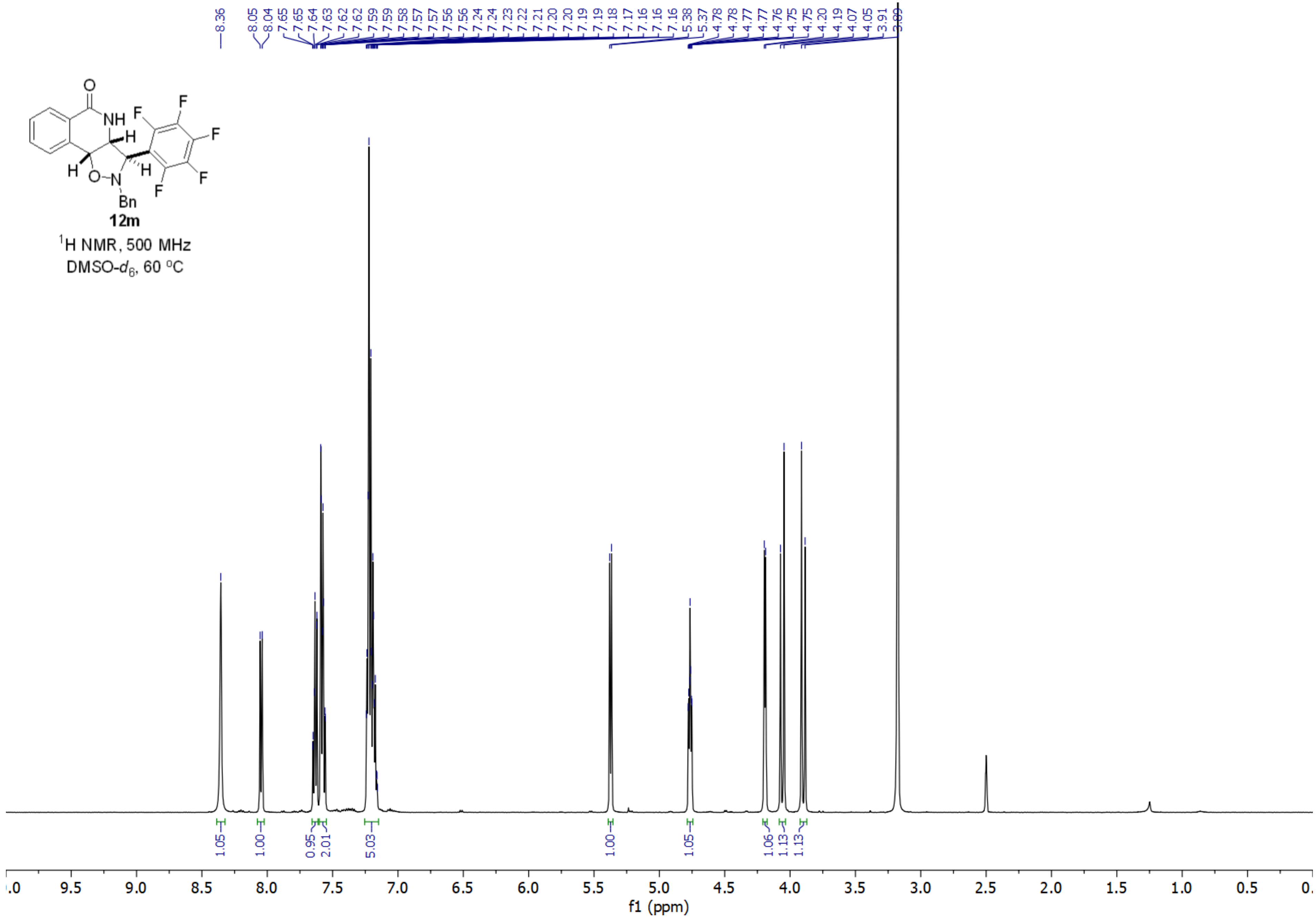

12m

^{1}H NMR, 500 MHz

DMSO-d_6, 60 °C

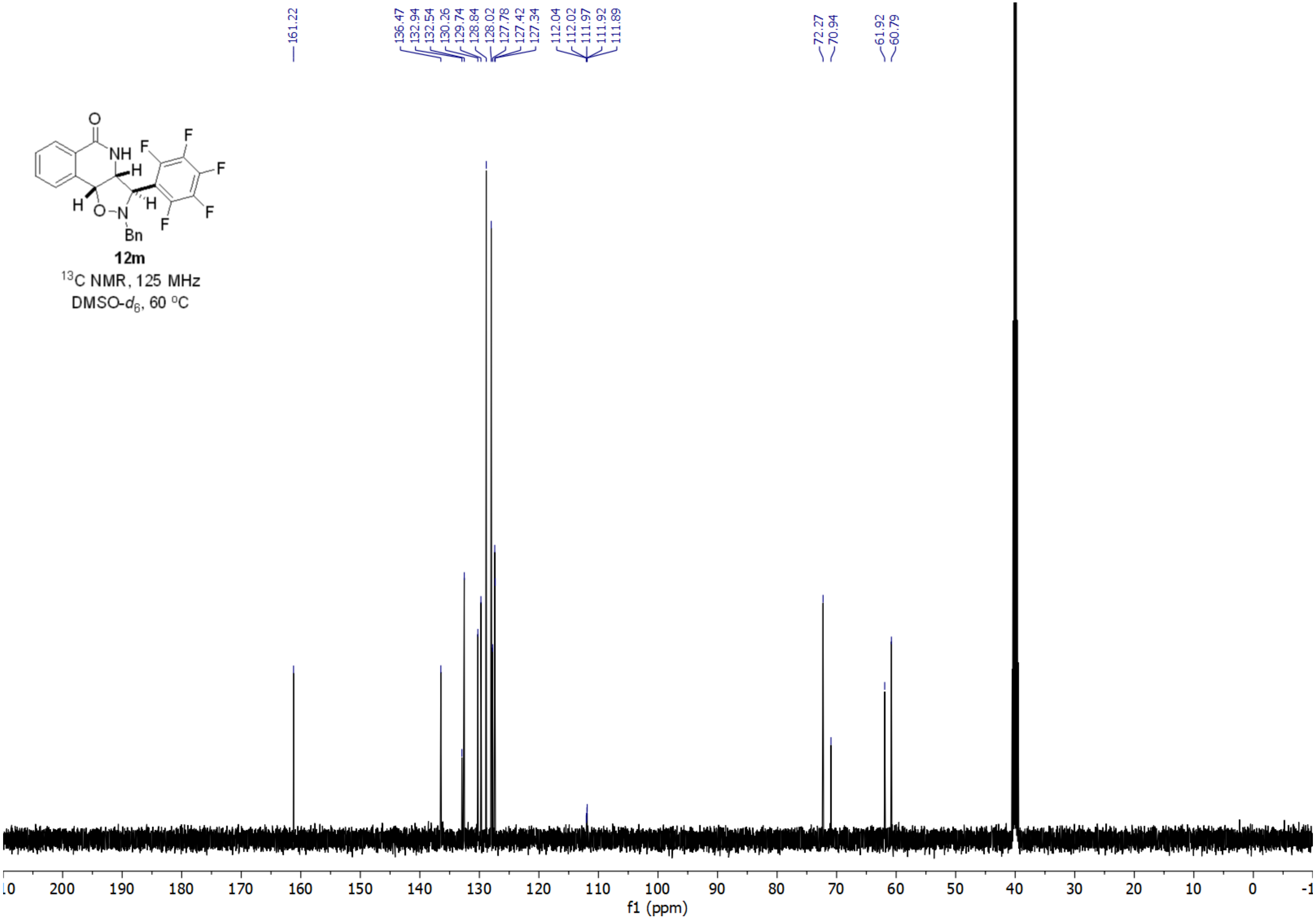

161.22
136.47
132.94
132.54
130.26
129.74
128.84
128.02
127.78
127.42
127.34
112.04
112.02
111.97
111.92
111.89
72.27
70.94
61.92
60.79
12m
^{13}C NMR, 125 MHz
DMSO-d_6, 60 °C
f1 (ppm)

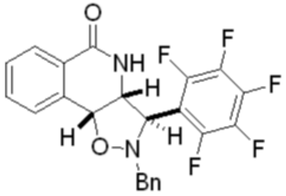

12m

^{19}F NMR, 470 MHz

DMSO-d_6, 25 °C

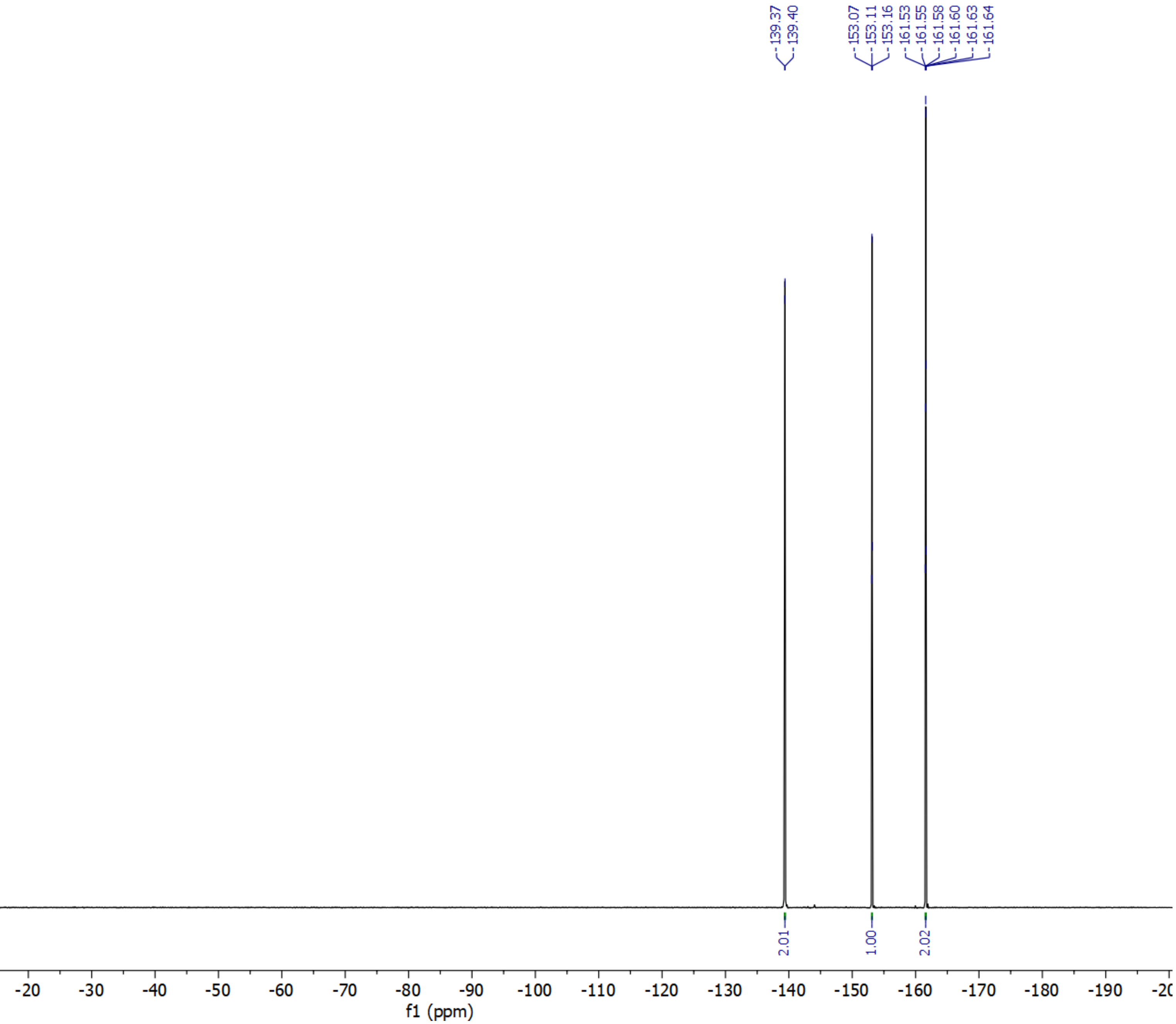

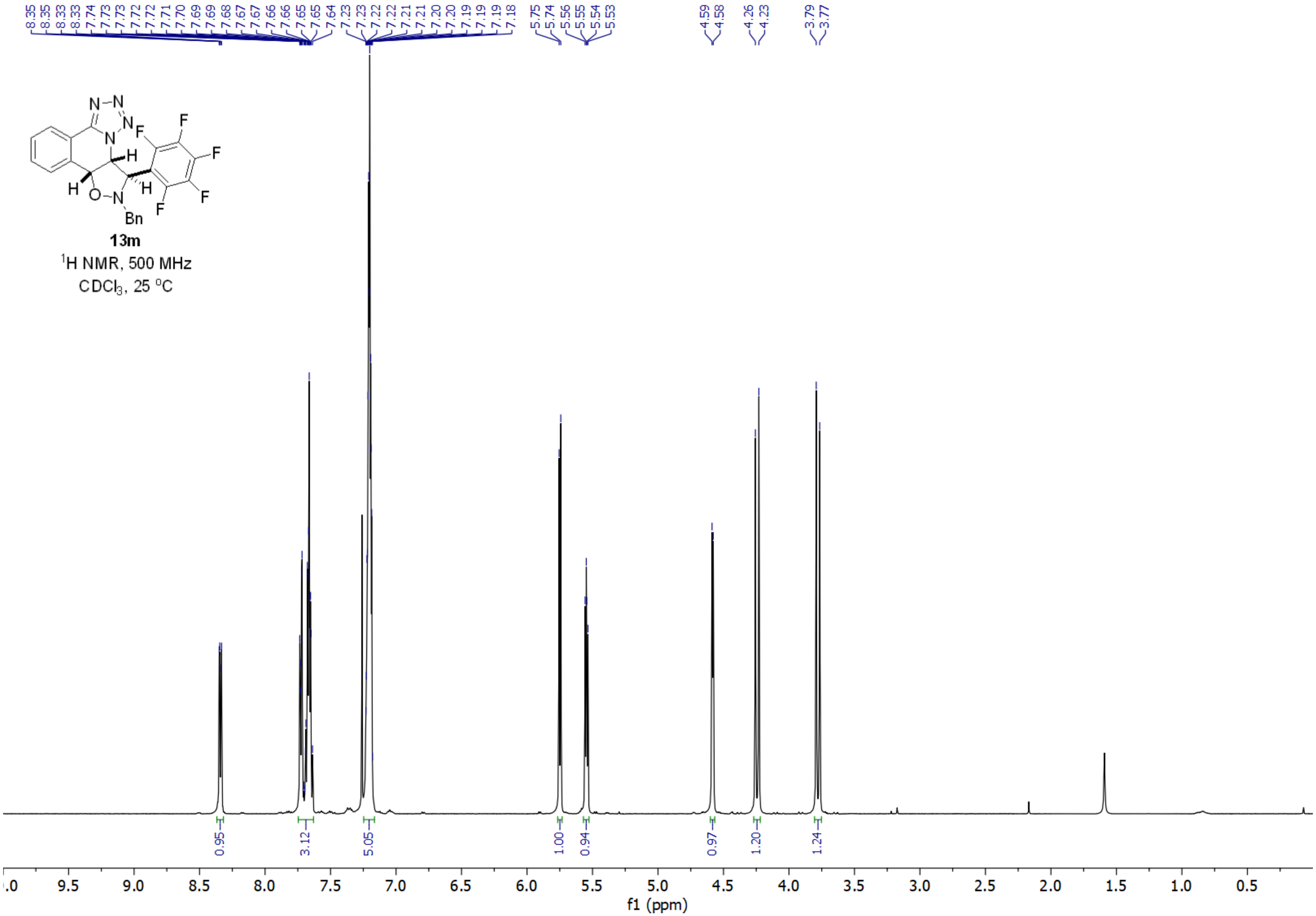

8.35 8.35 8.33 8.33 7.74 7.73 7.73 7.72 7.72 7.71 7.70 7.69 7.69 7.68 7.67 7.67 7.66 7.66 7.65 7.65 7.64 7.23 7.23 7.22 7.22 7.21 7.21 7.20 7.20 7.19 7.19 7.19 7.18 5.75 5.74 5.56 5.55 5.54 5.53 4.59 4.58 4.26 4.23 3.79 3.77
13m
^{1}H NMR, 500 MHz
$CDCl_3$, 25 °C
0.95 3.12 5.05 1.00 0.94 0.97 1.20 1.24
f1 (ppm)

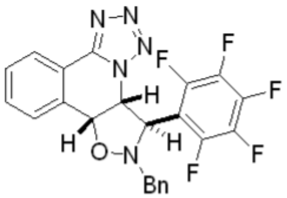

13m

^{13}C NMR, 125 MHz

$CDCl_3$, 25 ^oC

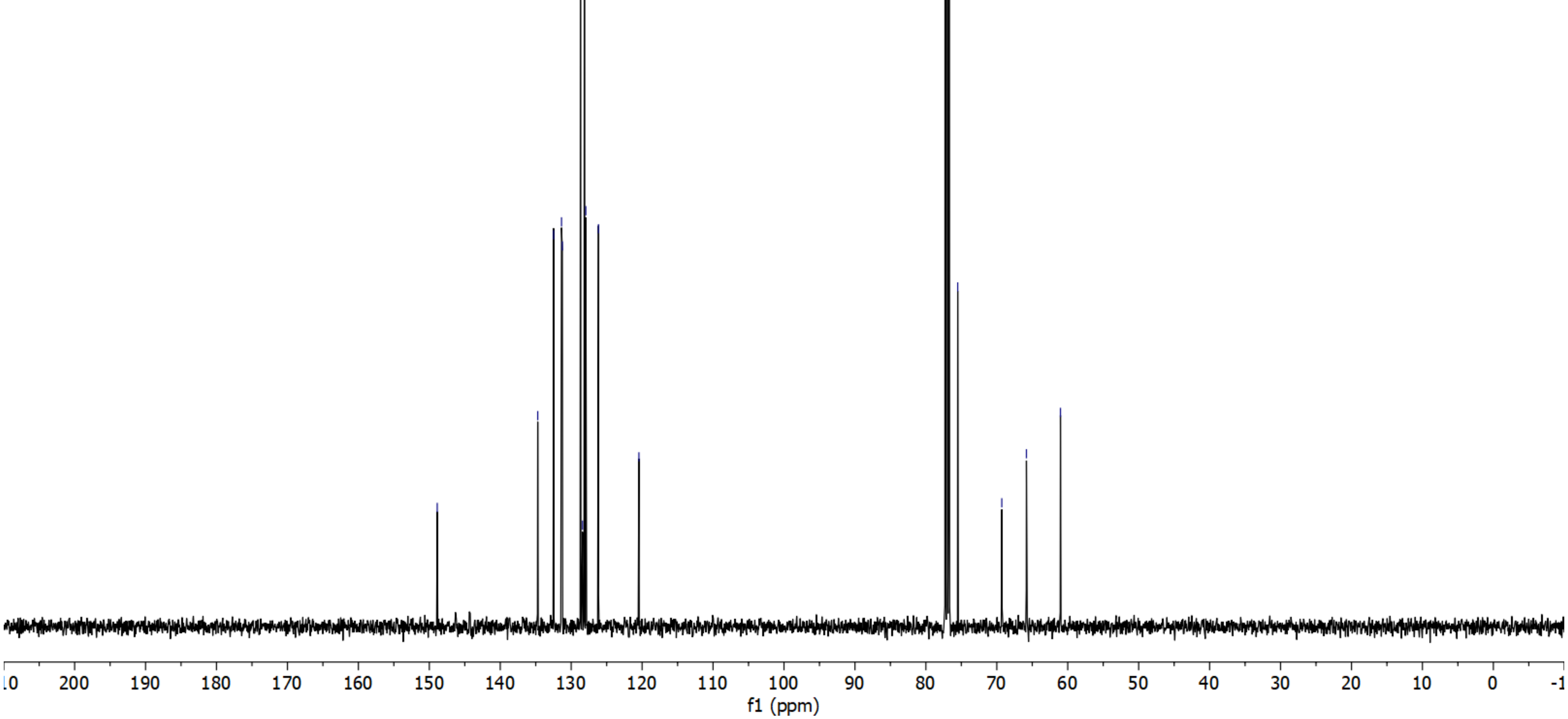

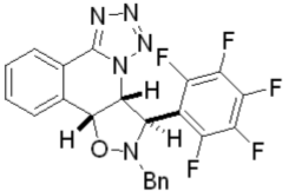

13m

^{19}F NMR, 470 MHz

$CDCl_3$, 25 °C

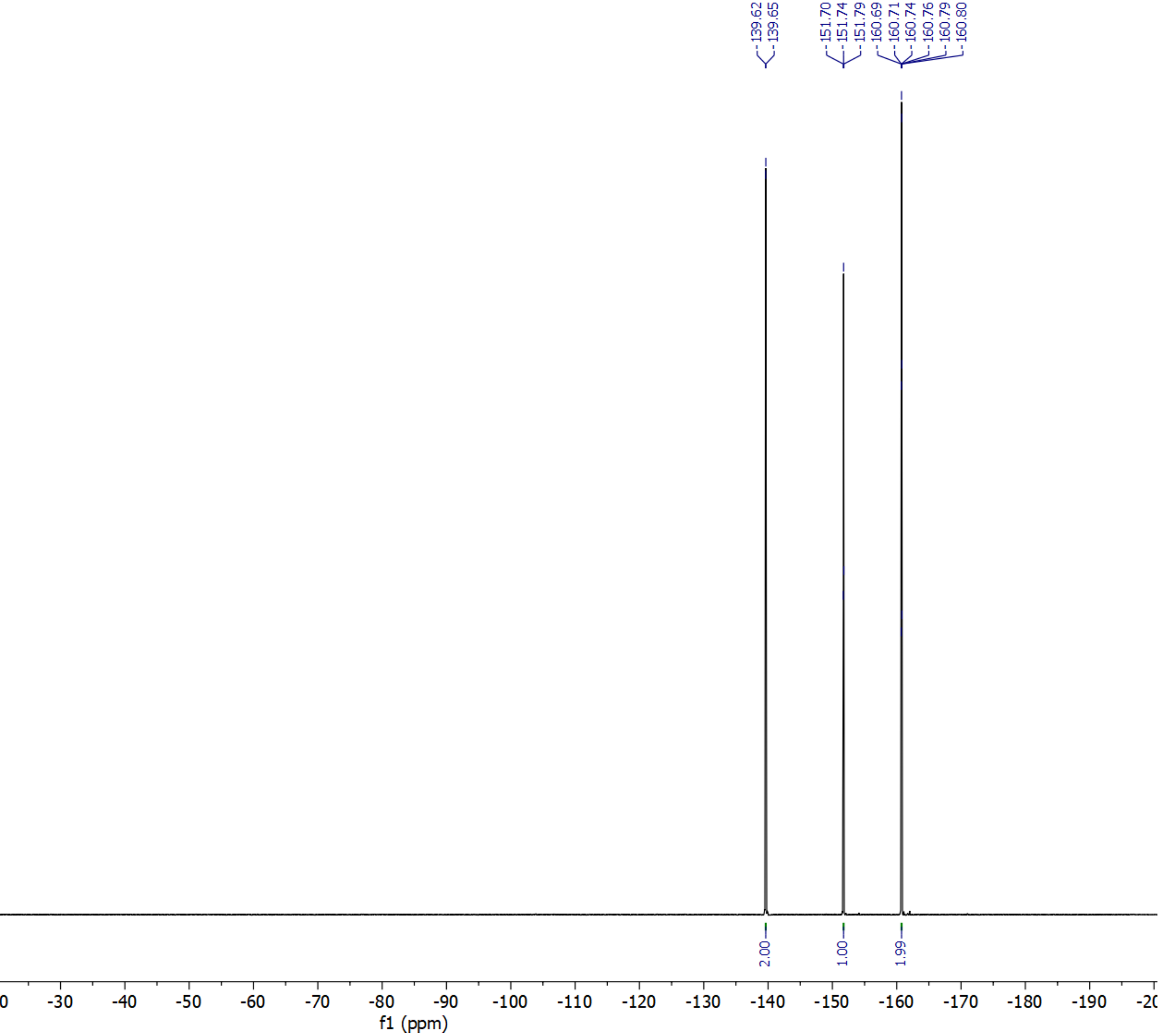

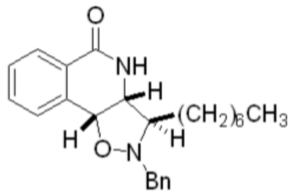

12n

^{1}H NMR, 500 MHz

DMSO-d_6, 60 °C

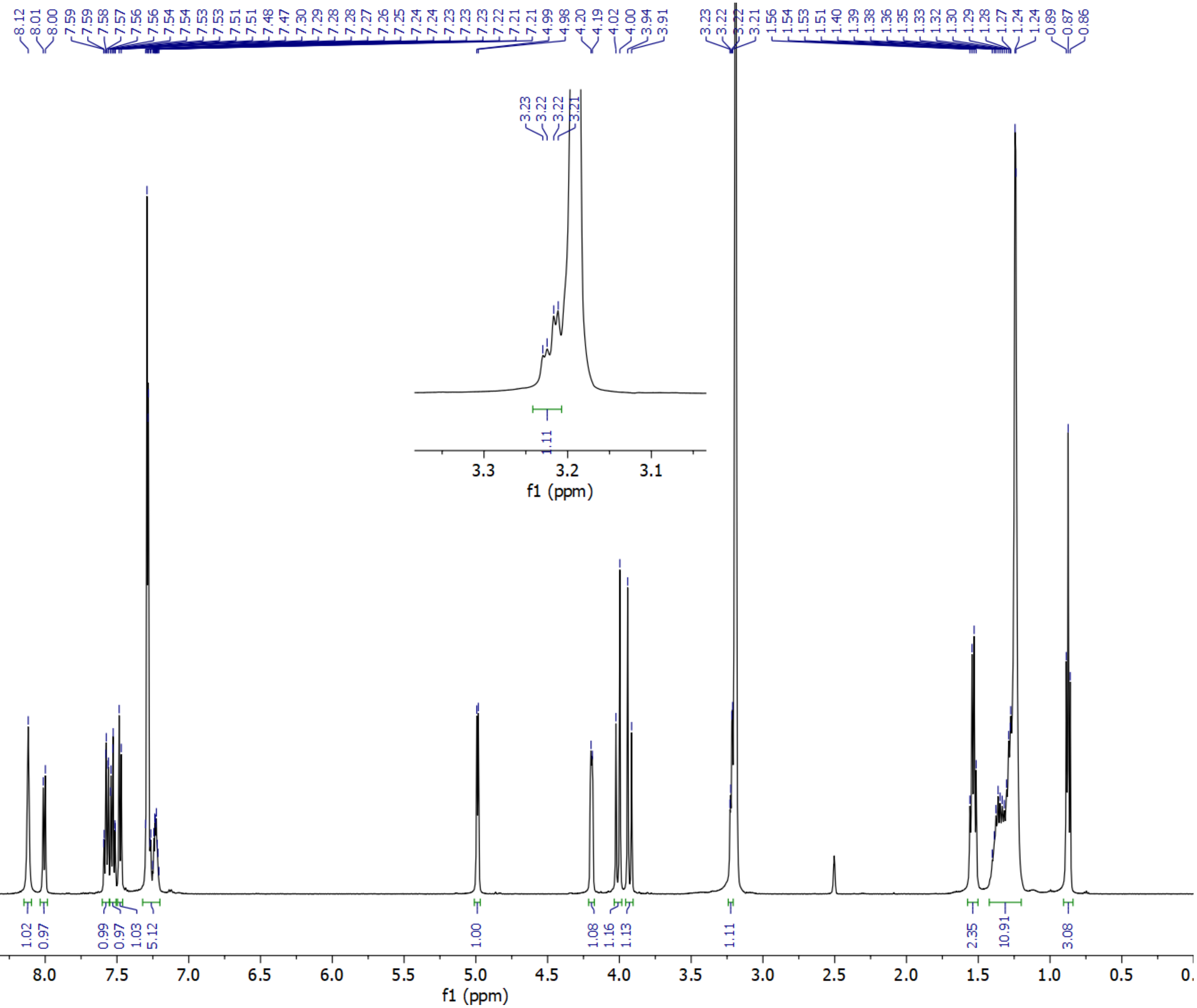

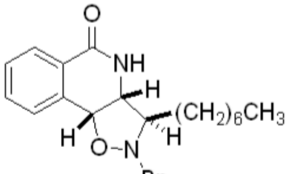

12n

^{13}C NMR, 125 MHz

DMSO-d_6, 60 °C

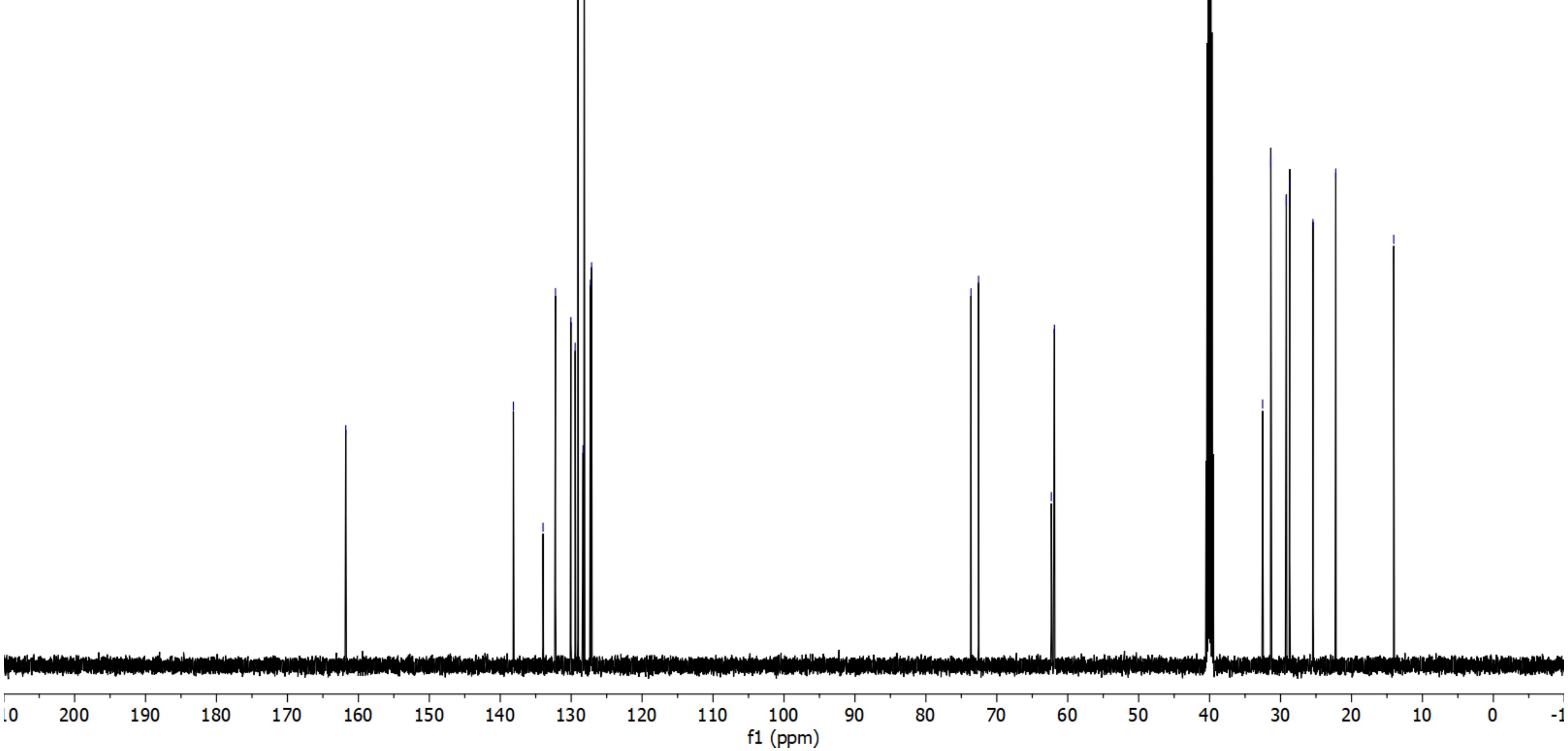

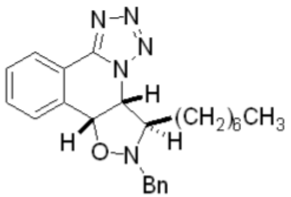

13n

^1H NMR, 500 MHz

$CDCl_3$, 25 °C

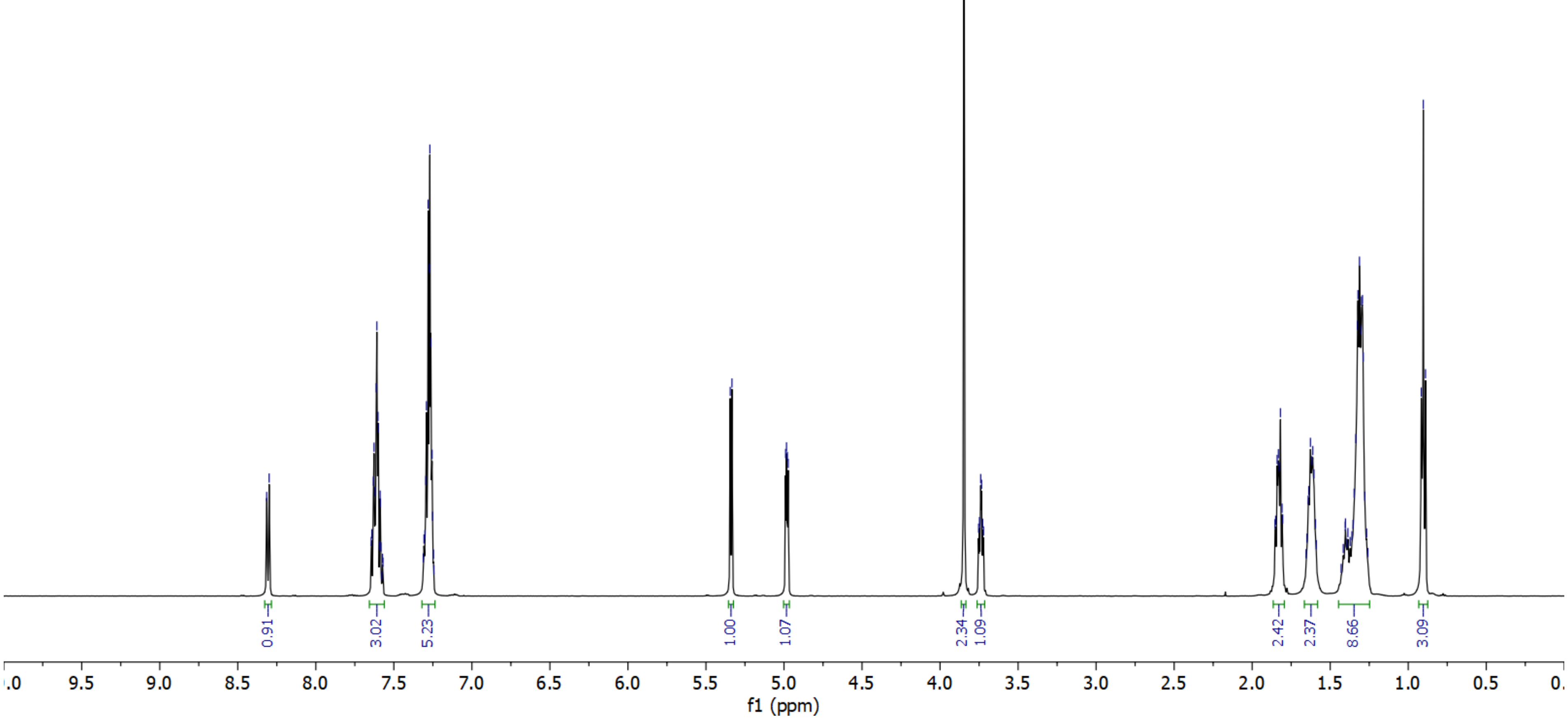

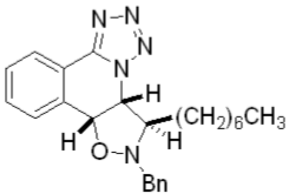

13n

^{13}C NMR, 125 MHz

$CDCl_3$, 25 °C

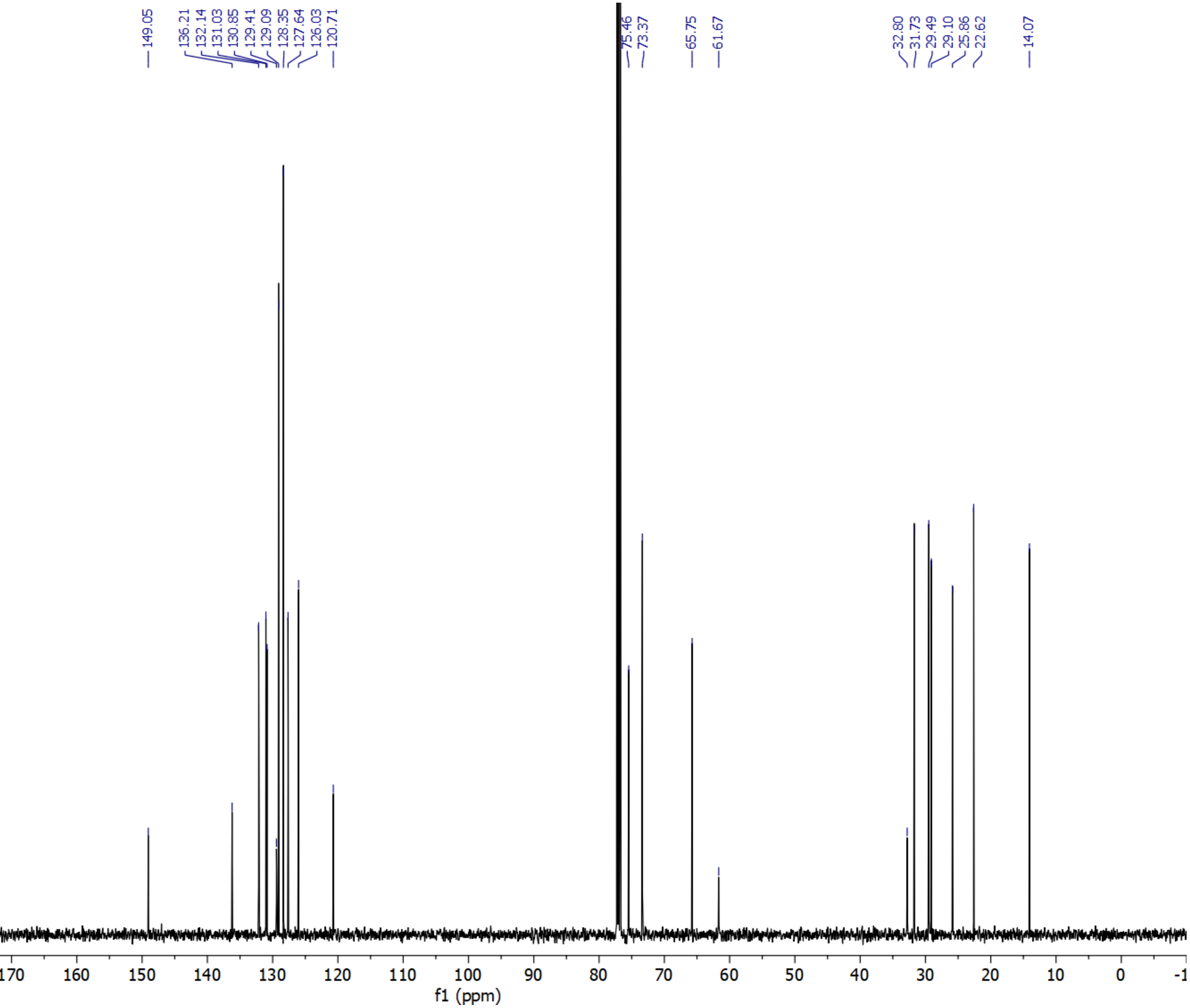

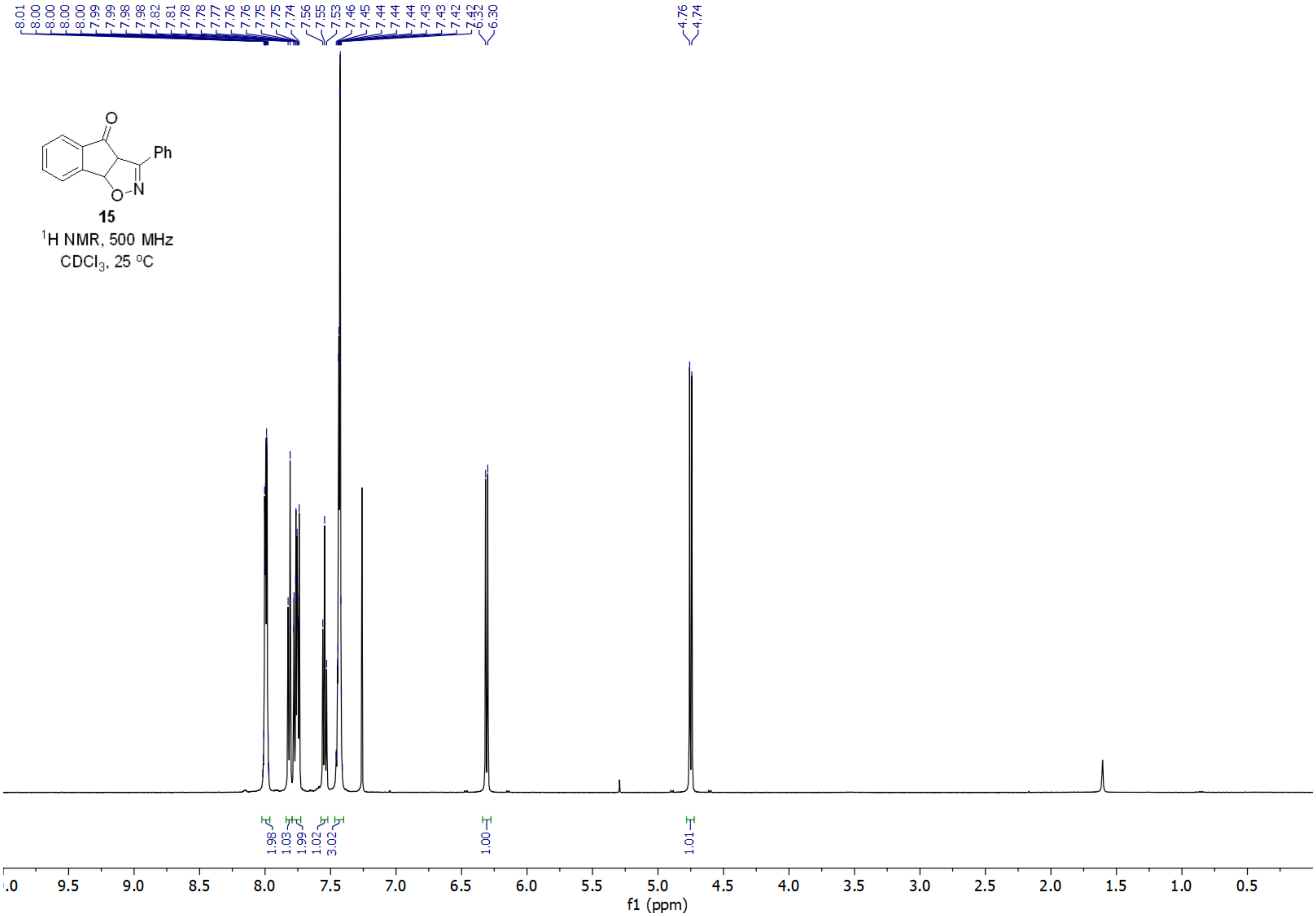

8.01 8.00 8.00 8.00 8.00 7.99 7.99 7.98 7.82 7.81 7.78 7.77 7.76 7.76 7.75 7.75 7.74 7.56 7.55 7.53 7.46 7.45 7.44 7.44 7.44 7.43 7.43 7.42 7.42 6.32 6.30
4.76 4.74
O
Ph
O
N
15
¹H NMR, 500 MHz
CDCl3, 25 °C
1.98
1.03
1.99
1.02
3.02
1.00
1.01
f1 (ppm)

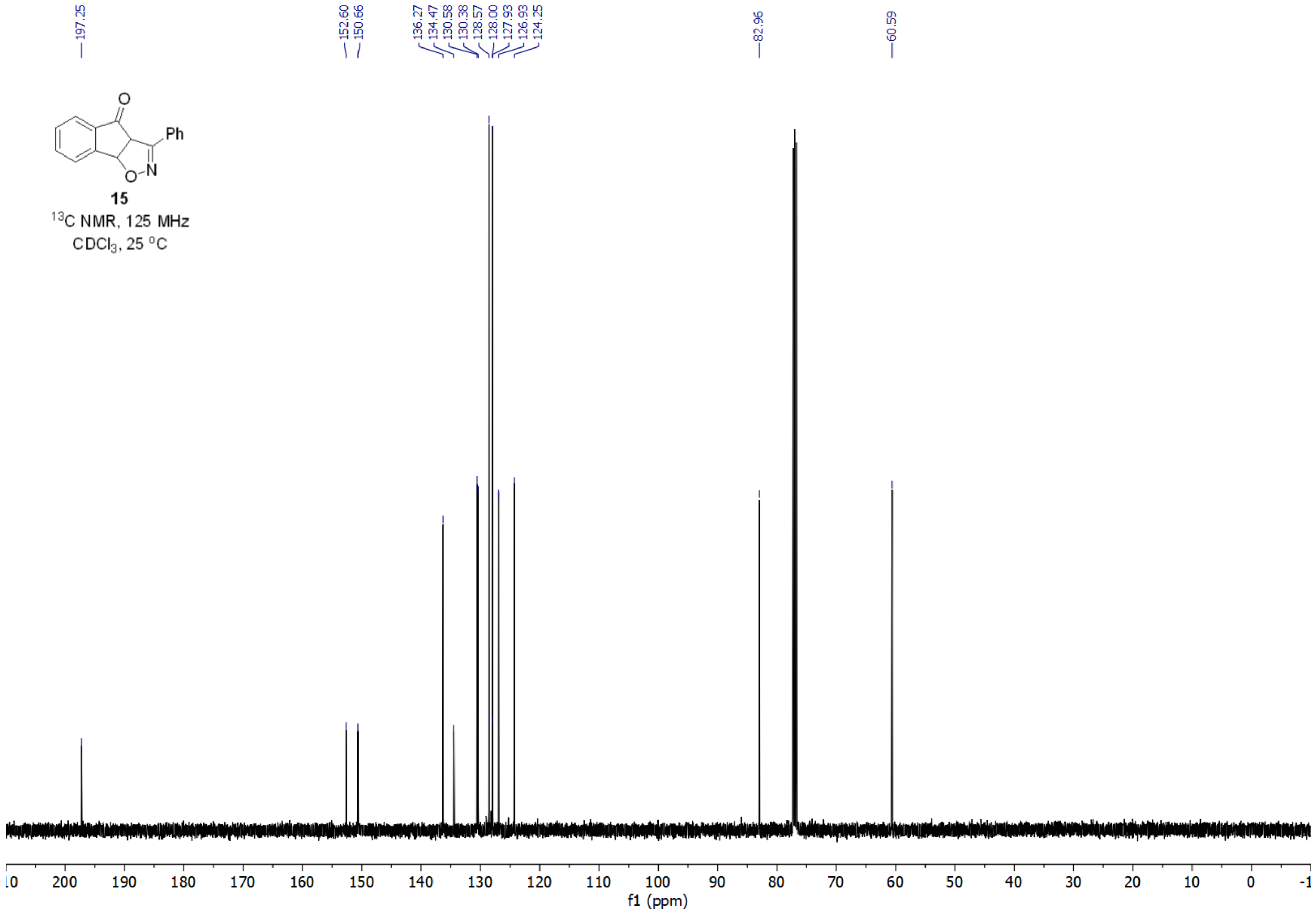
197.25
152.60
150.66
136.27
134.47
130.58
130.38
128.57
128.00
127.93
126.93
124.25
82.96
60.59
O
Ph
O
N
15
^{13}C NMR, 125 MHz
$CDCl_3$, 25 °C
f1 (ppm)
200
190
180
170
160
150
140
130
120
110
100
90
80
70
60
50
40
30
20
10
0

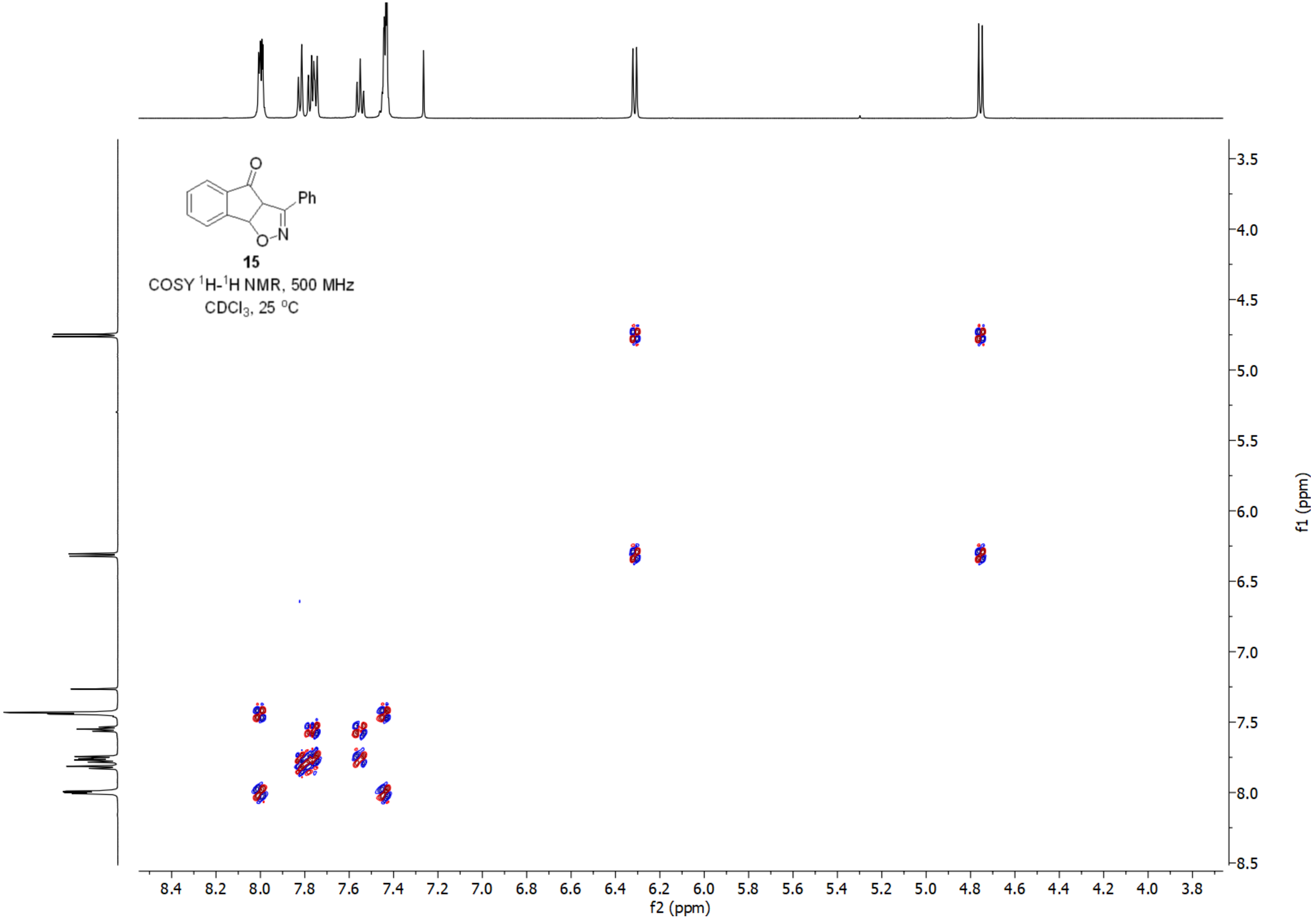
15
COSY 1H-1H NMR, 500 MHz
CDCl3, 25 °C
Ph
O
O–N
f2 (ppm)
f1 (ppm)

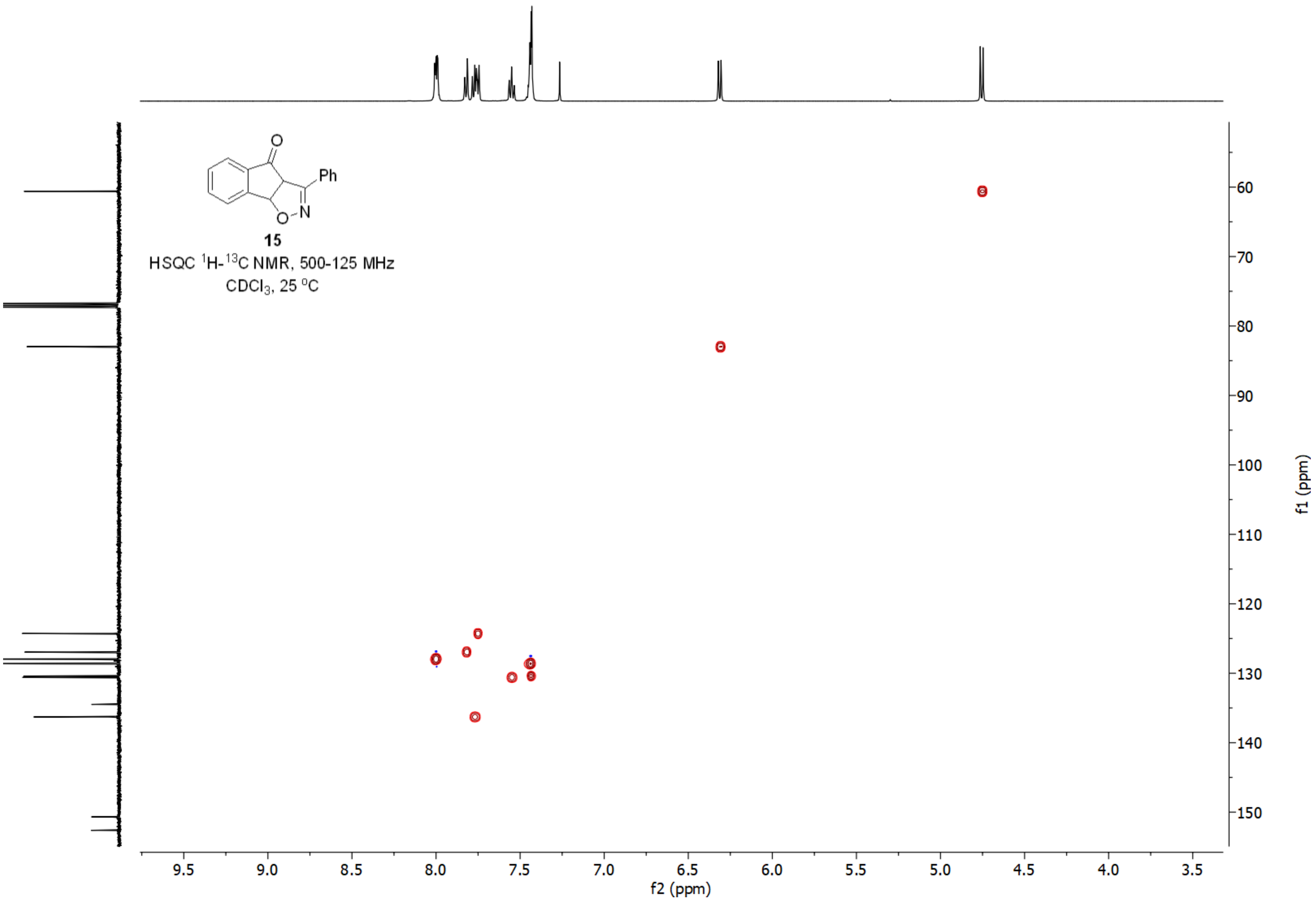

Ph
O
N
15
HSQC ¹H-¹³C NMR, 500-125 MHz
CDCl₃, 25 °C
f1 (ppm)
f2 (ppm)
9.5 9.0 8.5 8.0 7.5 7.0 6.5 6.0 5.5 5.0 4.5 4.0 3.5
60 70 80 90 100 110 120 130 140 150

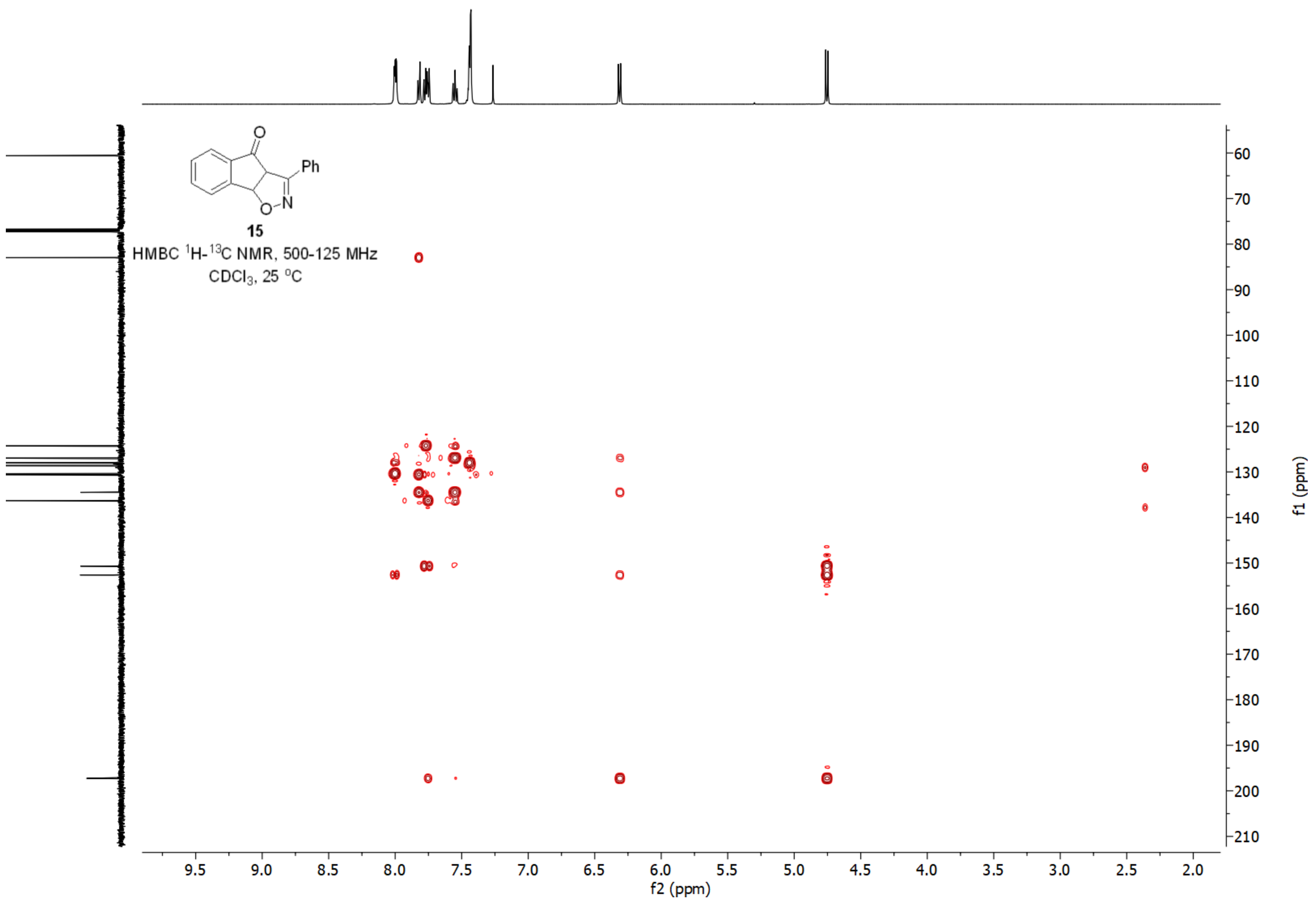

O
Ph
O
N
15
HMBC ^{1}H-^{13}C NMR, 500-125 MHz
$CDCl_3$, 25 °C
f2 (ppm)
f1 (ppm)

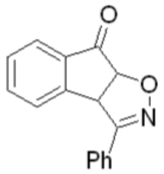

16

^{1}H NMR, 500 MHz

$CDCl_3$, 25 °C

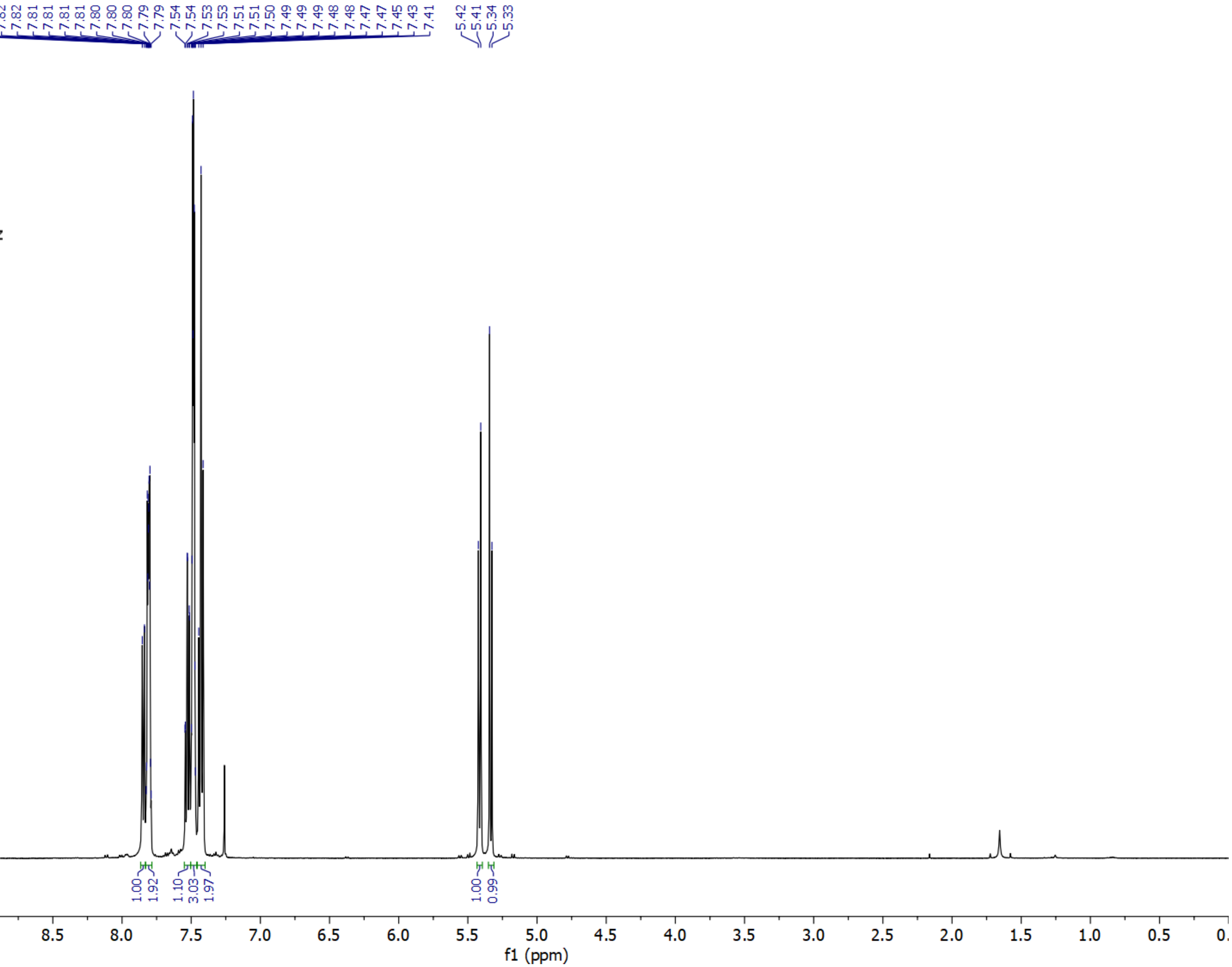

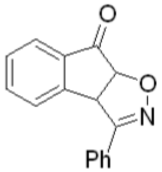

16

^{13}C NMR, 125 MHz
$CDCl_3$, 25 °C

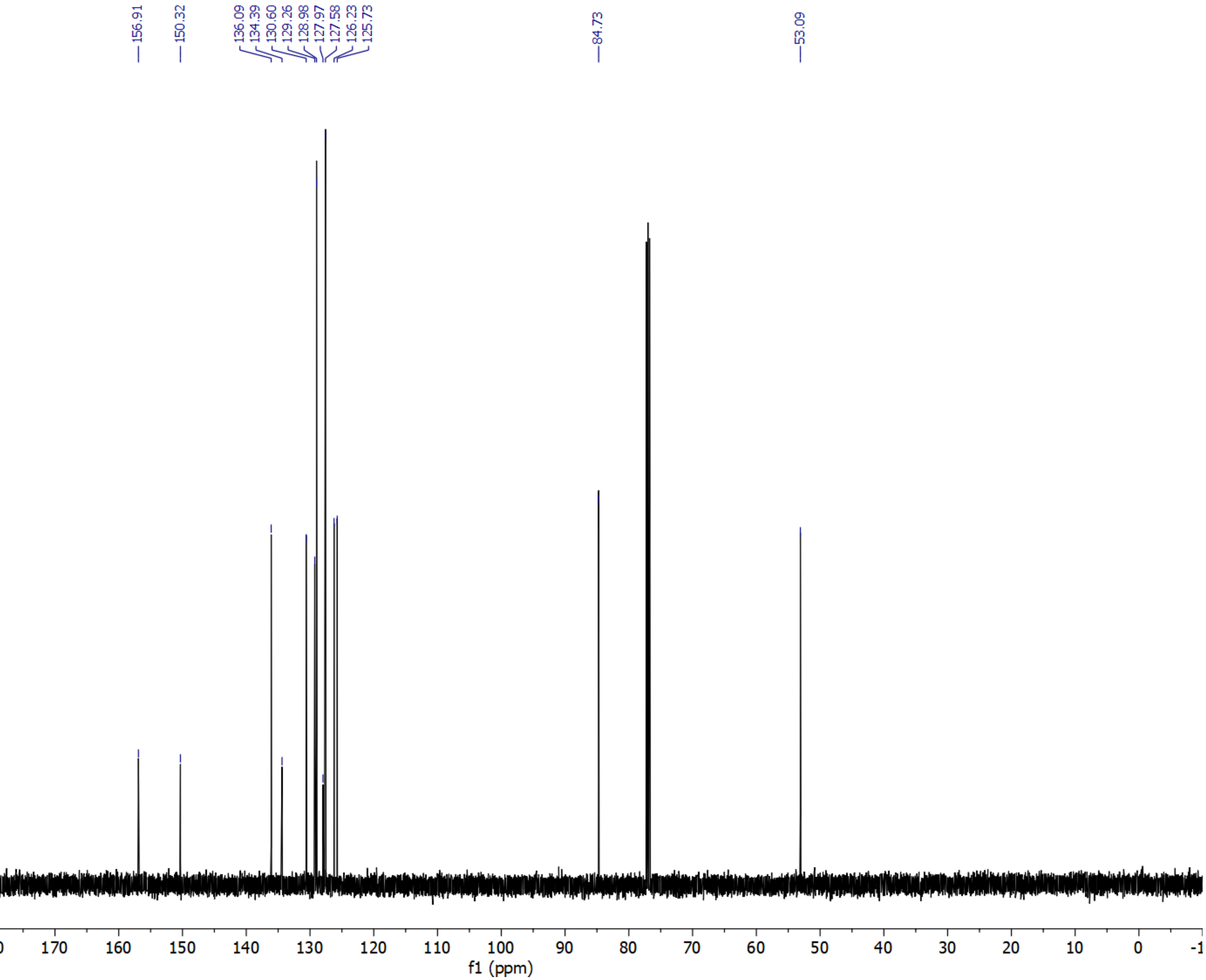

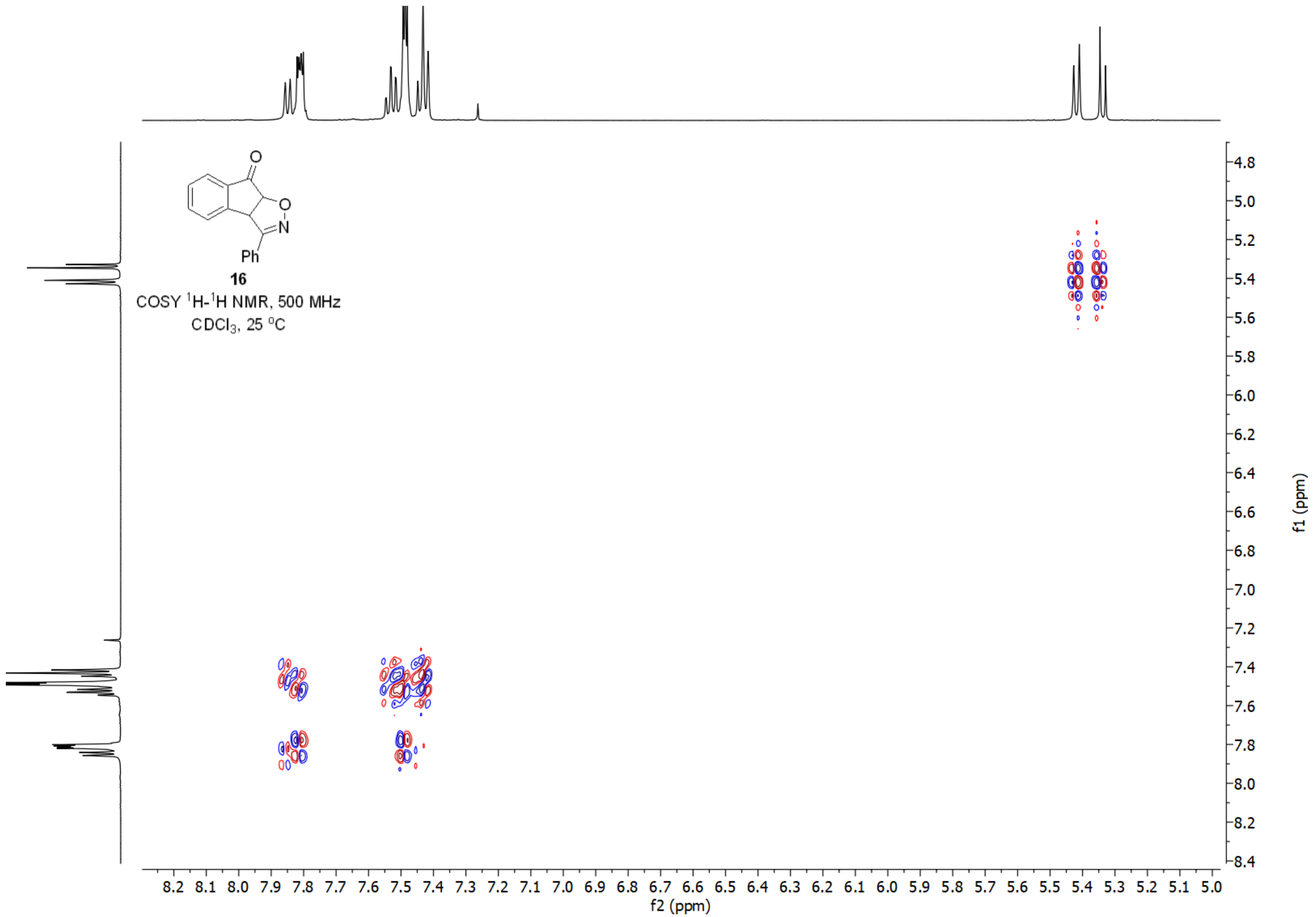

16

COSY ^{1}H-^{1}H NMR, 500 MHz

$CDCl_3$, 25 ^oC

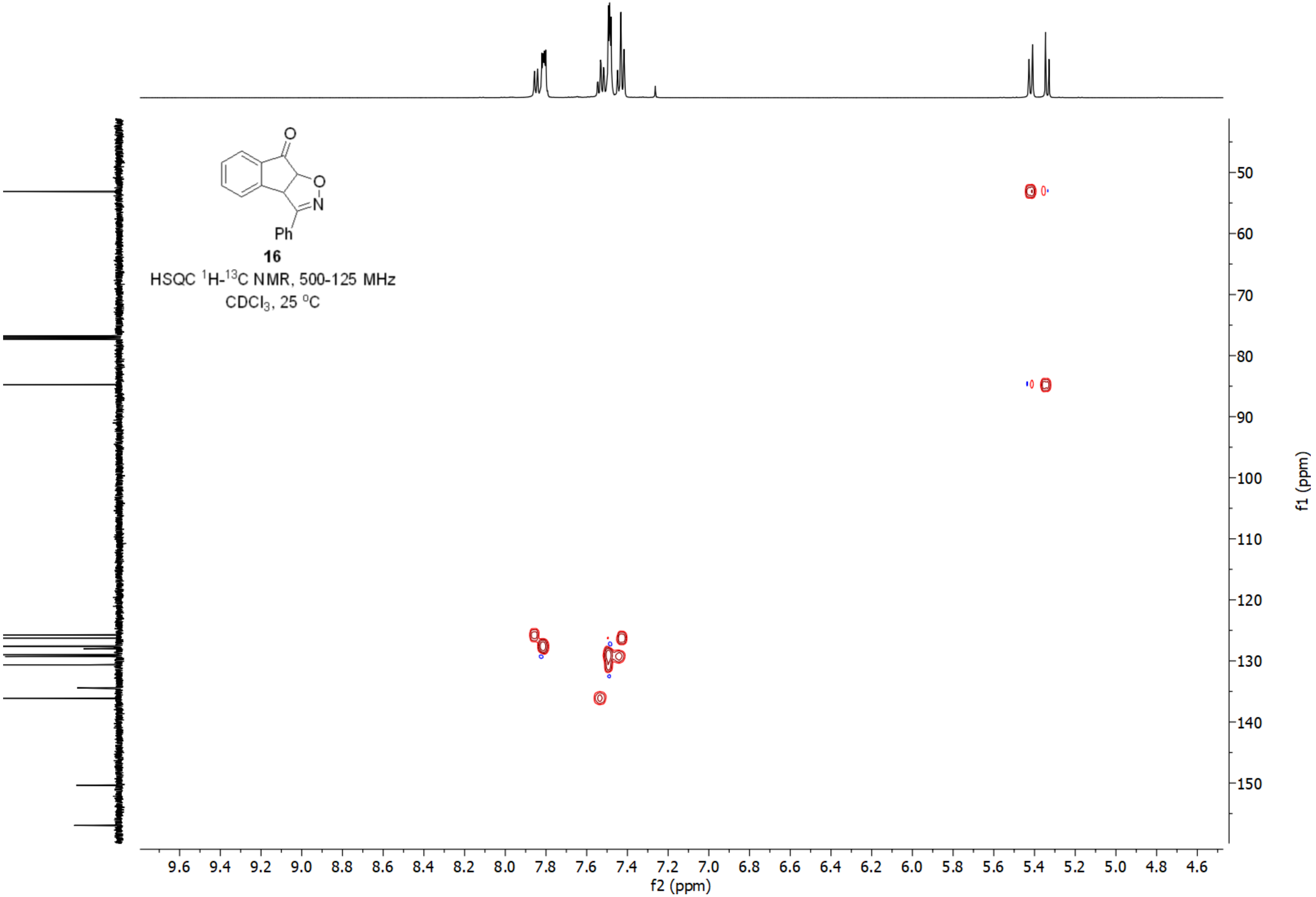
O
O
N
Ph
16
HSQC 1H-13C NMR, 500-125 MHz
CDCl3, 25 °C
f2 (ppm)
f1 (ppm)

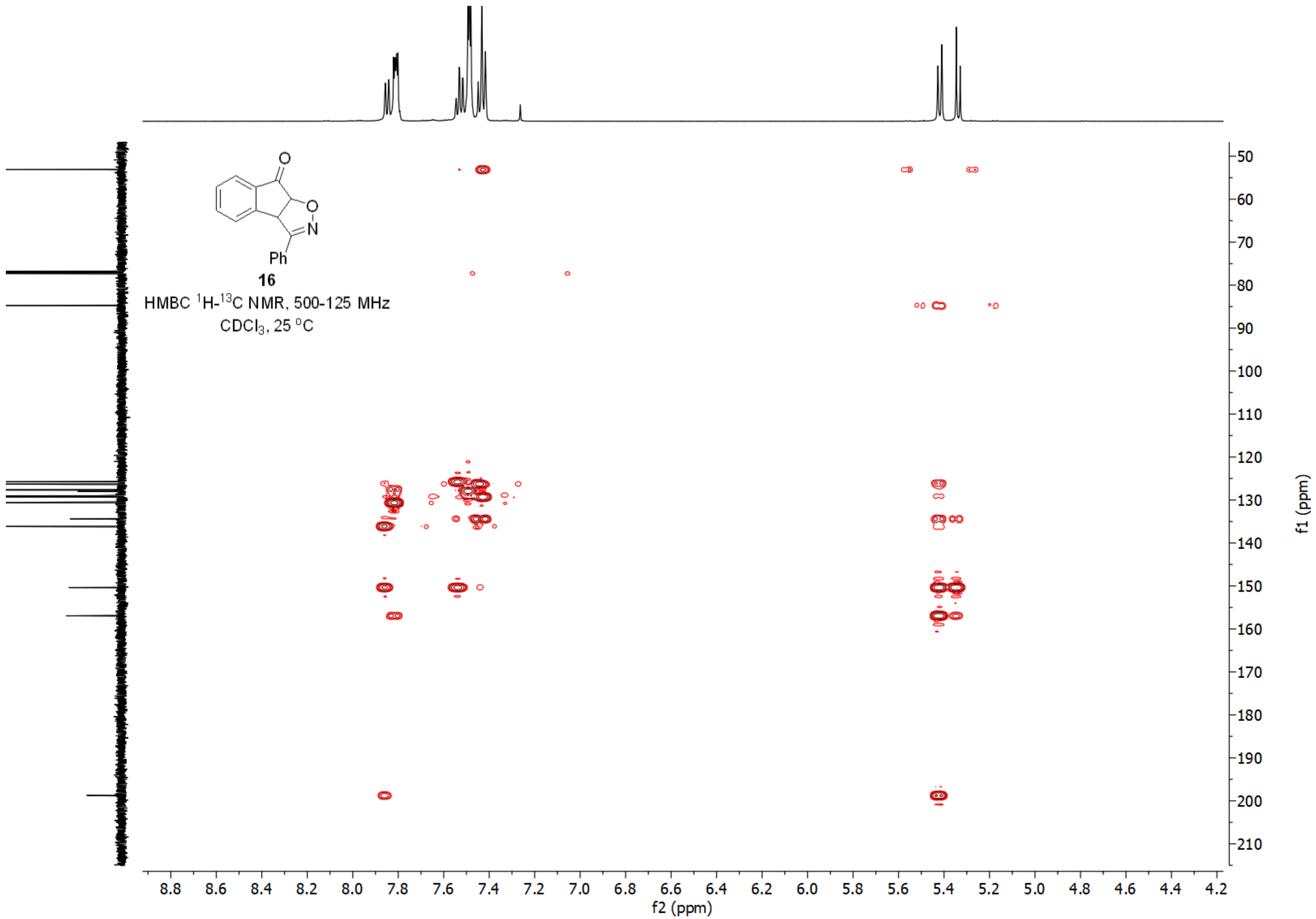

O
O
N
Ph
16
HMBC ^{1}H-^{13}C NMR, 500-125 MHz
$CDCl_3$, 25 °C
f2 (ppm)
f1 (ppm)

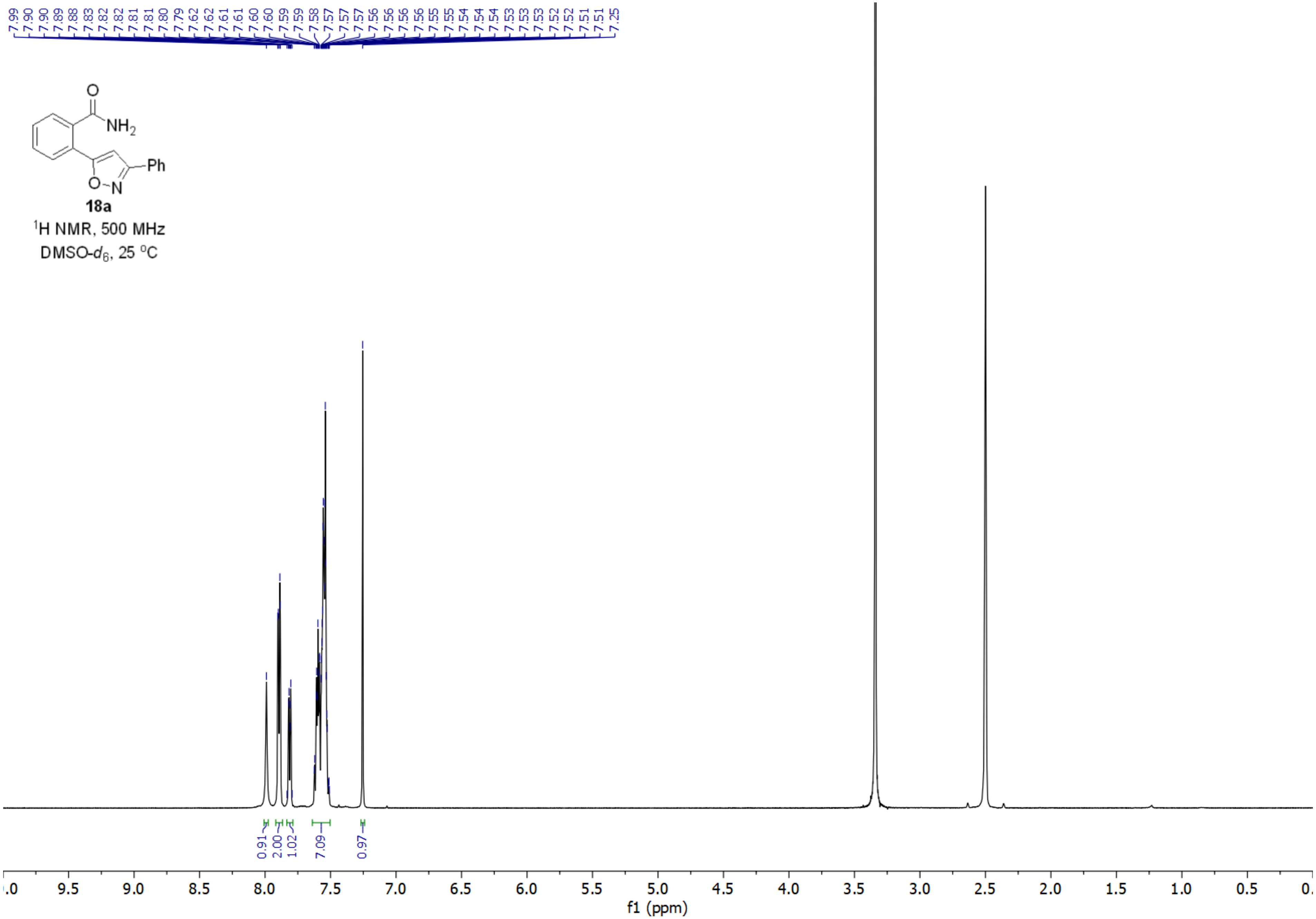
7.99
7.90
7.90
7.89
7.88
7.83
7.82
7.82
7.81
7.81
7.80
7.79
7.62
7.62
7.61
7.61
7.60
7.60
7.59
7.59
7.58
7.57
7.57
7.57
7.56
7.56
7.56
7.55
7.55
7.54
7.54
7.54
7.53
7.53
7.53
7.52
7.52
7.51
7.51
7.25
O
NH2
Ph
O
N
18a
1H NMR, 500 MHz
DMSO-d6, 25 °C
0.91
2.00
1.02
7.09
0.97
f1 (ppm)

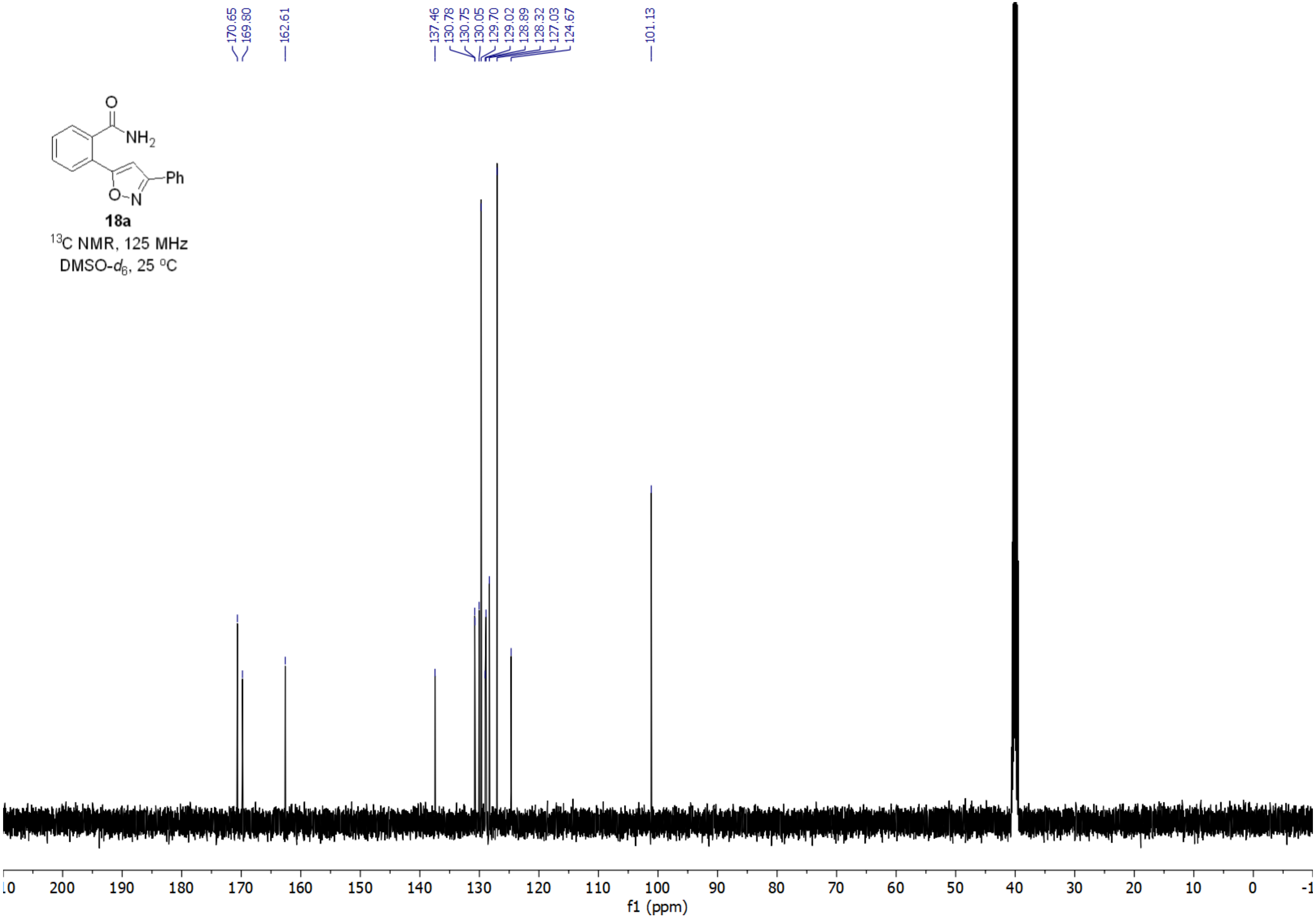
O
NH2
Ph
O
N
18a
13C NMR, 125 MHz
DMSO-d6, 25 °C
170.65
169.80
162.61
137.46
130.78
130.75
130.05
129.70
129.02
128.89
128.32
127.03
124.67
101.13
10
200
190
180
170
160
150
140
130
120
110
100
90
80
70
60
50
40
30
20
10
0
-1
f1 (ppm)

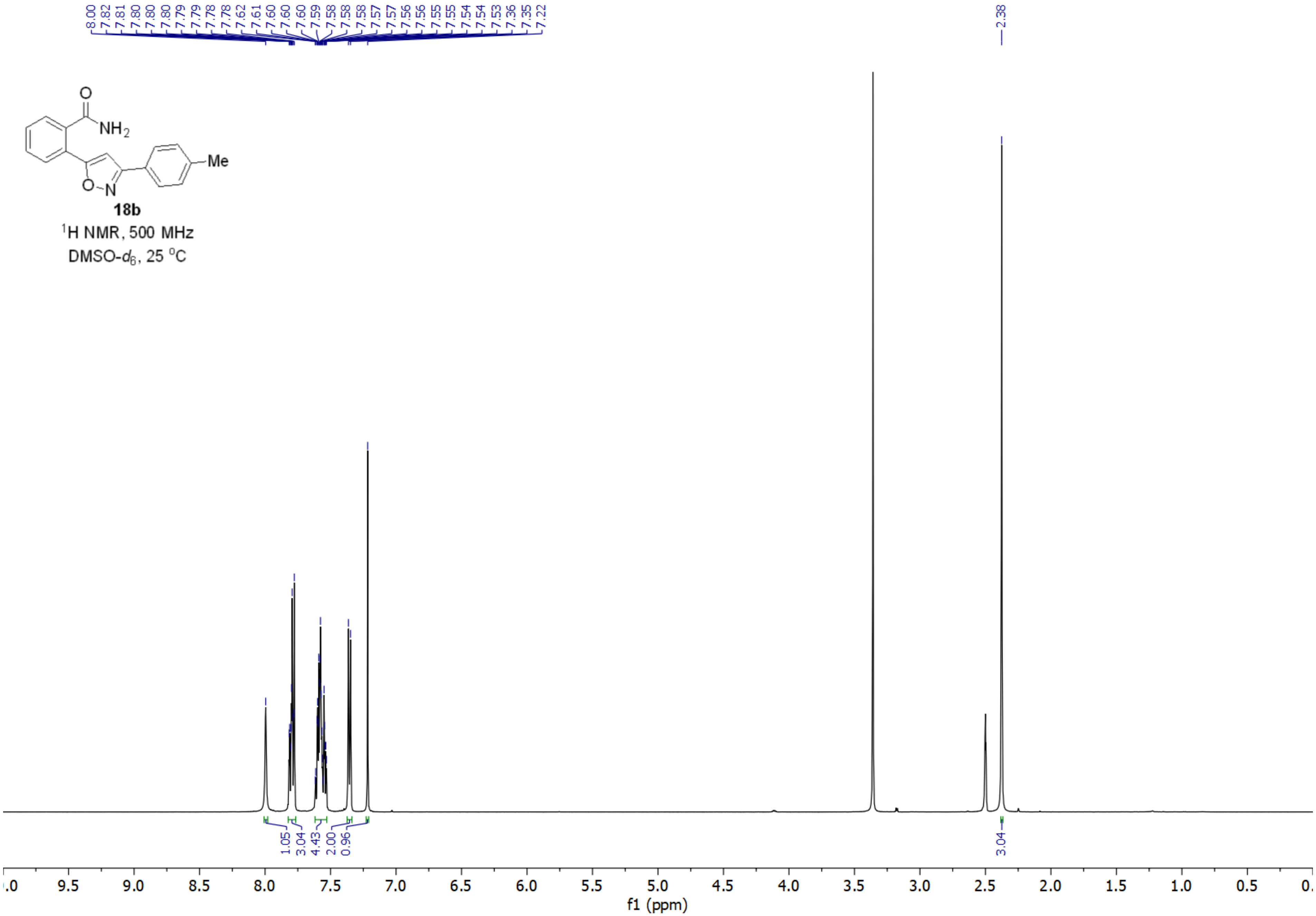

18b

^1H NMR, 500 MHz

DMSO-d_6, 25 °C

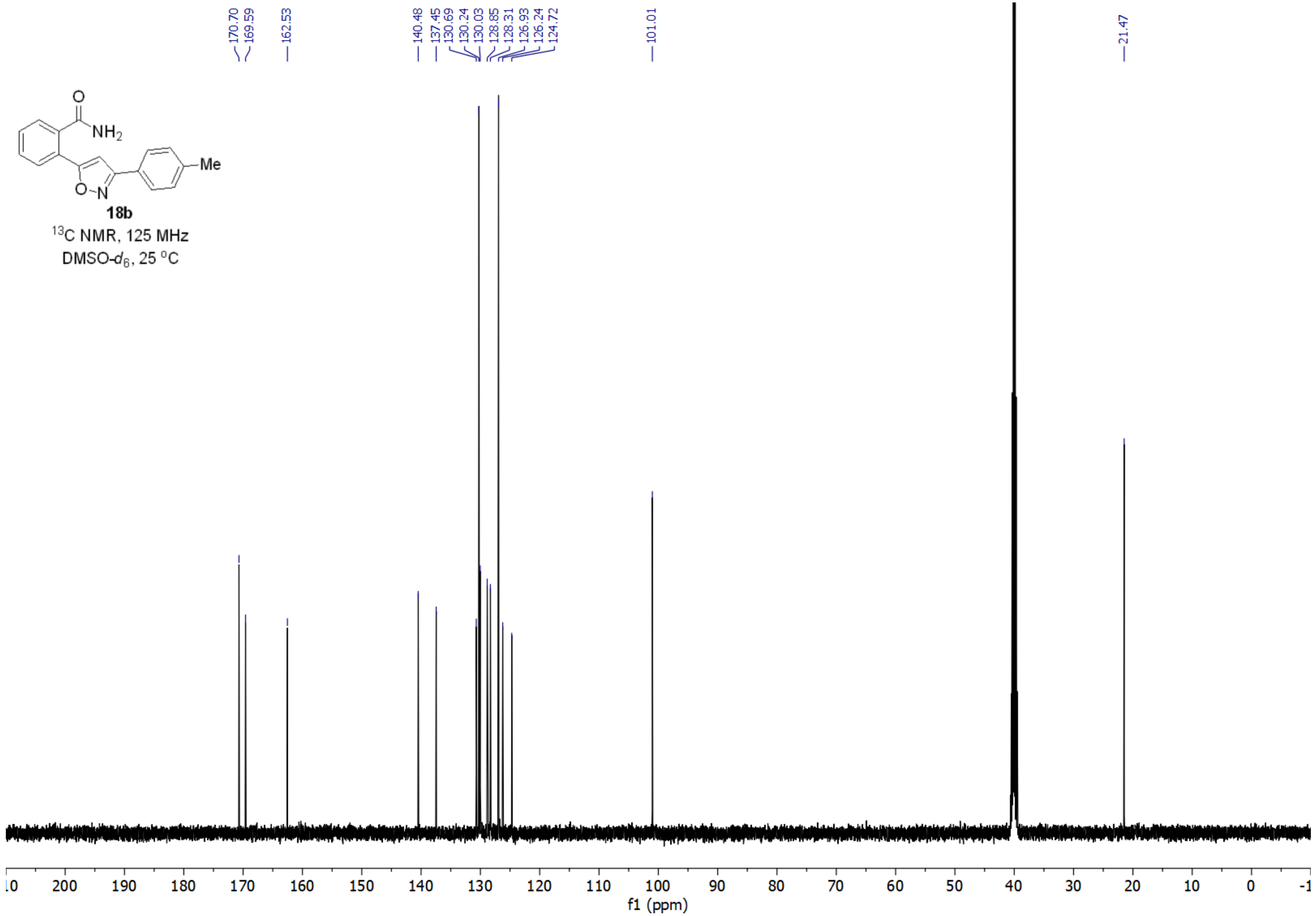
170.70
169.59
162.53
140.48
137.45
130.69
130.24
130.03
128.85
128.31
126.93
126.24
124.72
101.01
21.47
O
NH2
Me
O
N
18b
13C NMR, 125 MHz
DMSO-d6, 25 °C
f1 (ppm)

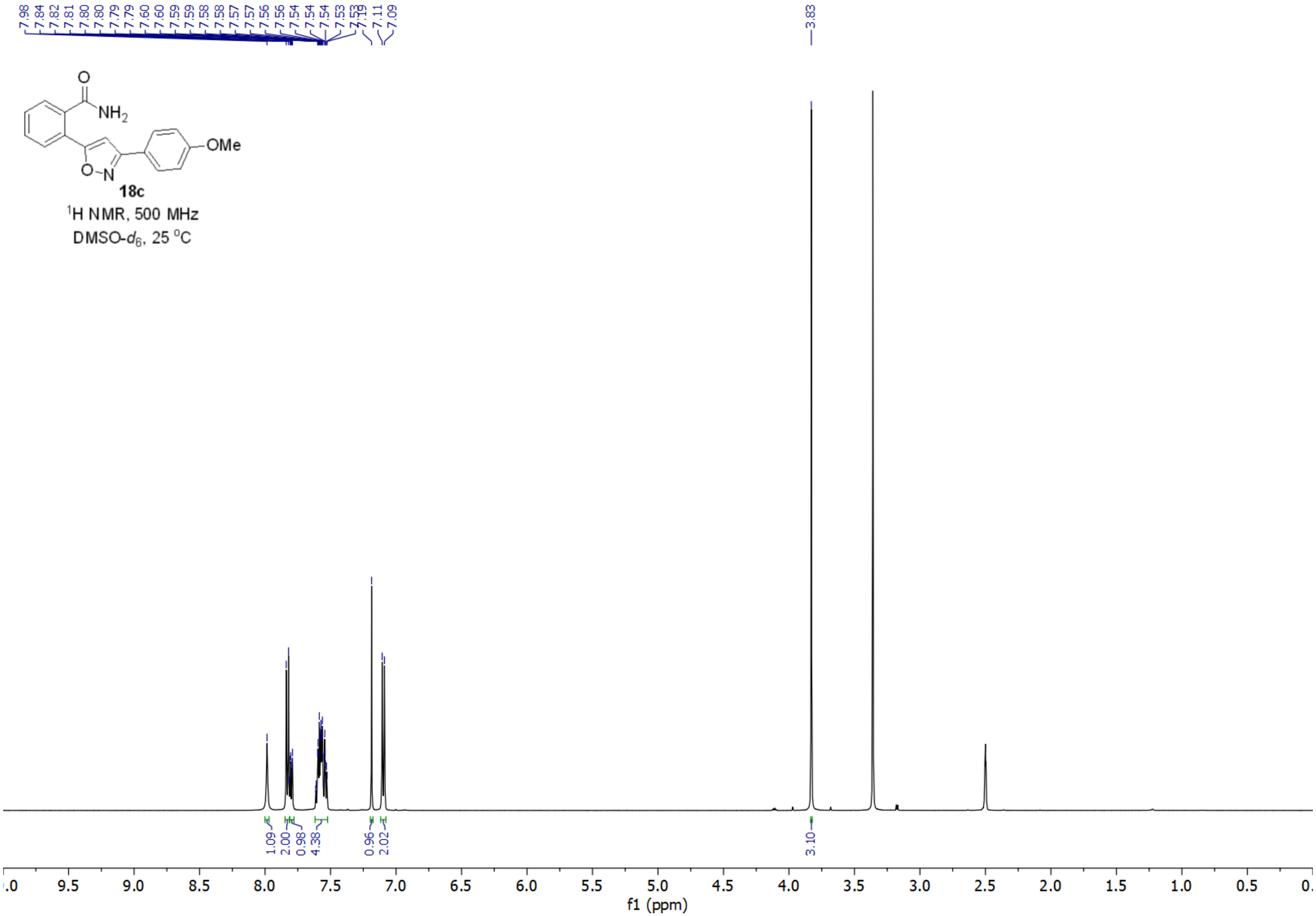

7.98
7.84
7.82
7.81
7.80
7.80
7.79
7.79
7.60
7.60
7.59
7.59
7.58
7.58
7.57
7.57
7.56
7.56
7.54
7.54
7.54
7.53
7.53
7.19
7.11
7.09
3.83
O
NH2
OMe
O
N
18c
1H NMR, 500 MHz
DMSO-d6, 25 °C
1.09
2.00
0.98
4.38
0.96
2.02
3.10
9.5
9.0
8.5
8.0
7.5
7.0
6.5
6.0
5.5
5.0
4.5
4.0
3.5
3.0
2.5
2.0
1.5
1.0
0.5
f1 (ppm)

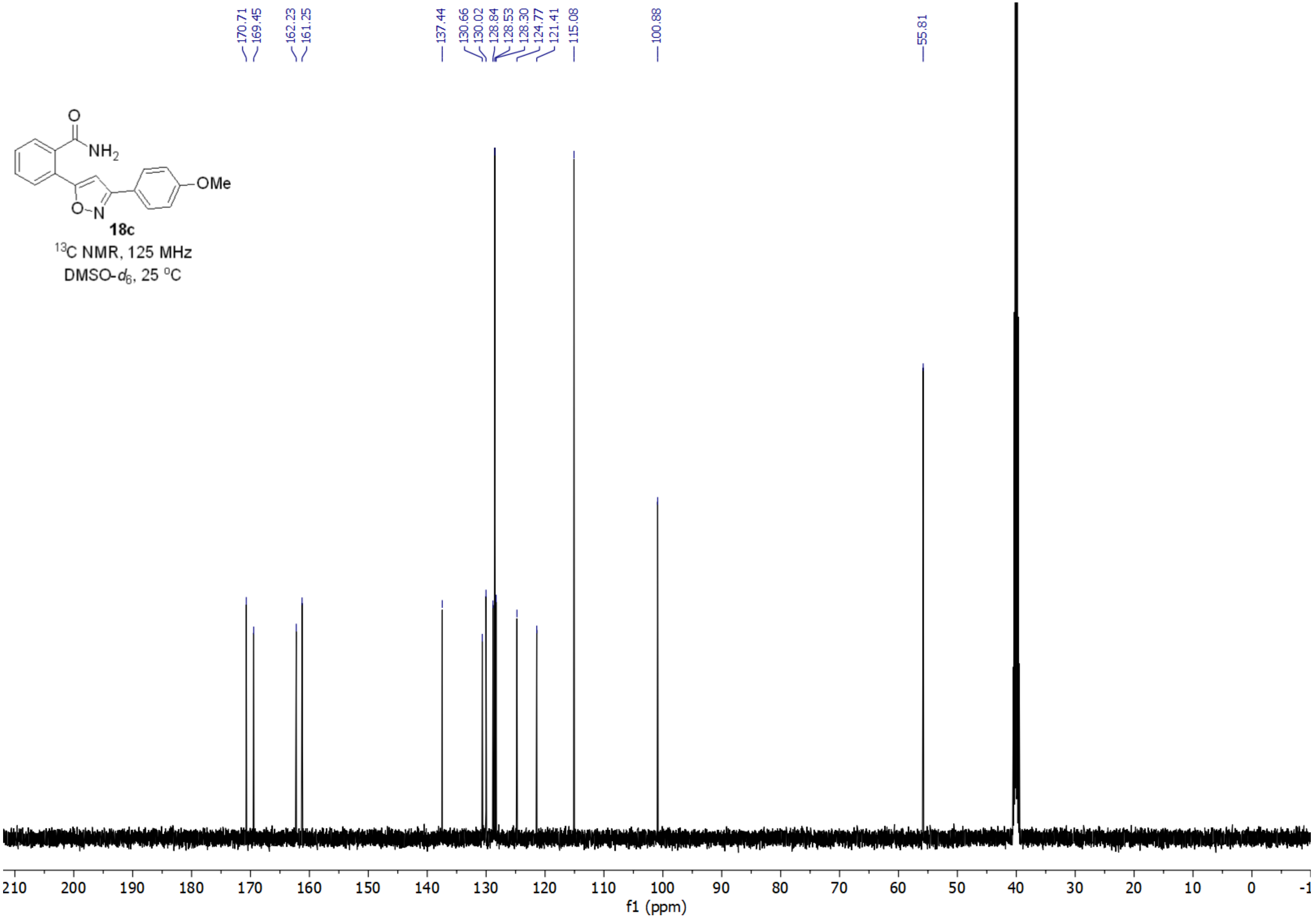
170.71
169.45
162.23
161.25
137.44
130.66
130.02
128.84
128.53
128.30
124.77
121.41
115.08
100.88
55.81
OMe
NH2
O
N
18c
13C NMR, 125 MHz
DMSO-d6, 25 °C
f1 (ppm)

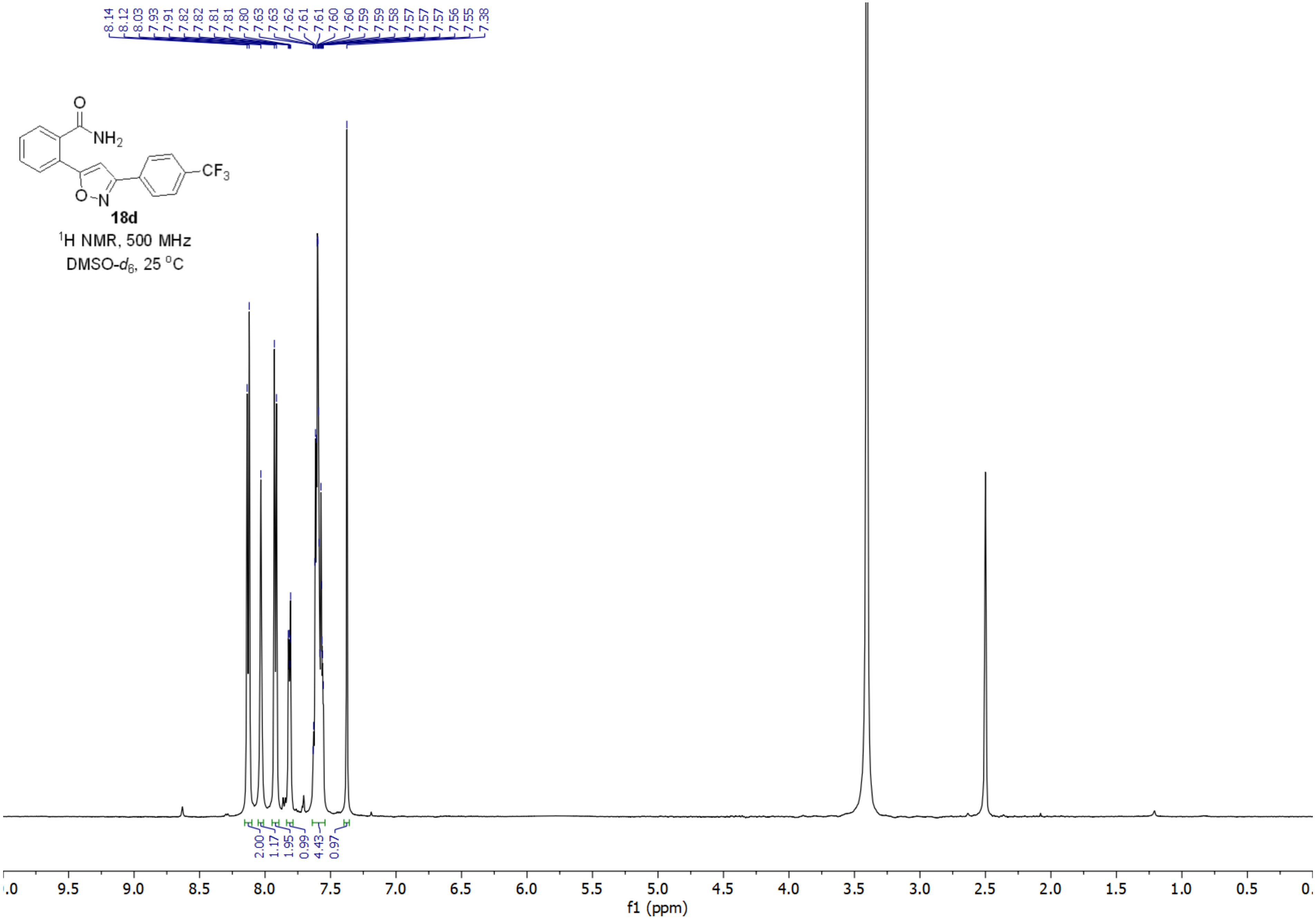
8.14
8.12
8.03
7.93
7.91
7.82
7.82
7.81
7.81
7.80
7.63
7.63
7.62
7.61
7.61
7.60
7.60
7.59
7.59
7.58
7.57
7.57
7.57
7.56
7.55
7.38
O
NH2
CF3
O
N
18d
1H NMR, 500 MHz
DMSO-d6, 25 °C
2.00
1.17
1.95
0.99
4.43
0.97
9.5
9.0
8.5
8.0
7.5
7.0
6.5
6.0
5.5
5.0
4.5
4.0
3.5
3.0
2.5
2.0
1.5
1.0
0.5
f1 (ppm)

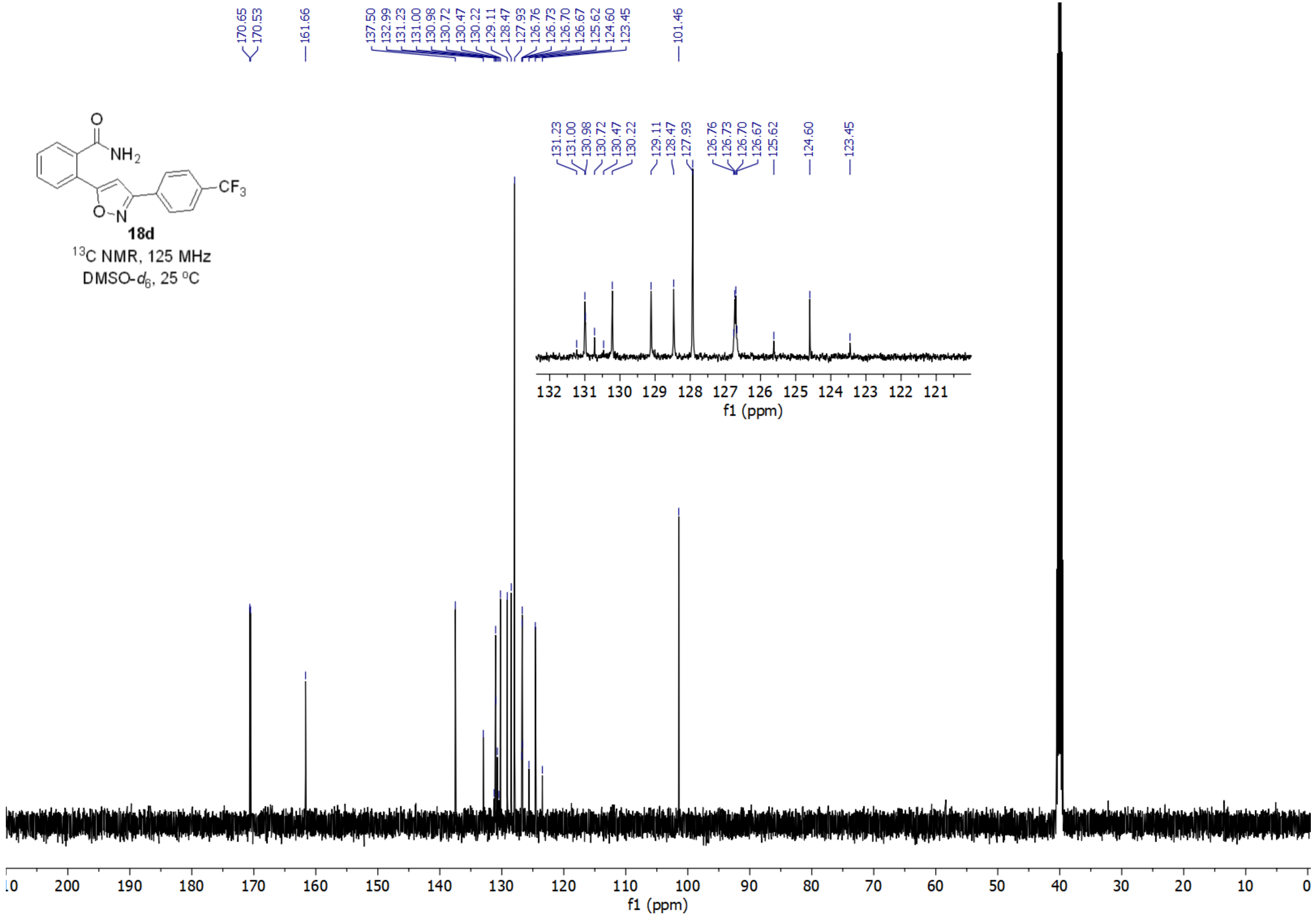

170.65
170.53
161.66
137.50
132.99
131.23
131.00
130.98
130.72
130.47
130.22
129.11
128.47
127.93
126.76
126.73
126.70
126.67
125.62
124.60
123.45
101.46
O
NH2
CF3
O
N
18d
13C NMR, 125 MHz
DMSO-d6, 25 °C
f1 (ppm)

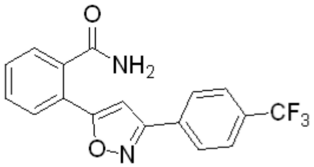

18d

^{19}F NMR, 470 MHz

DMSO-d_6, 25 °C

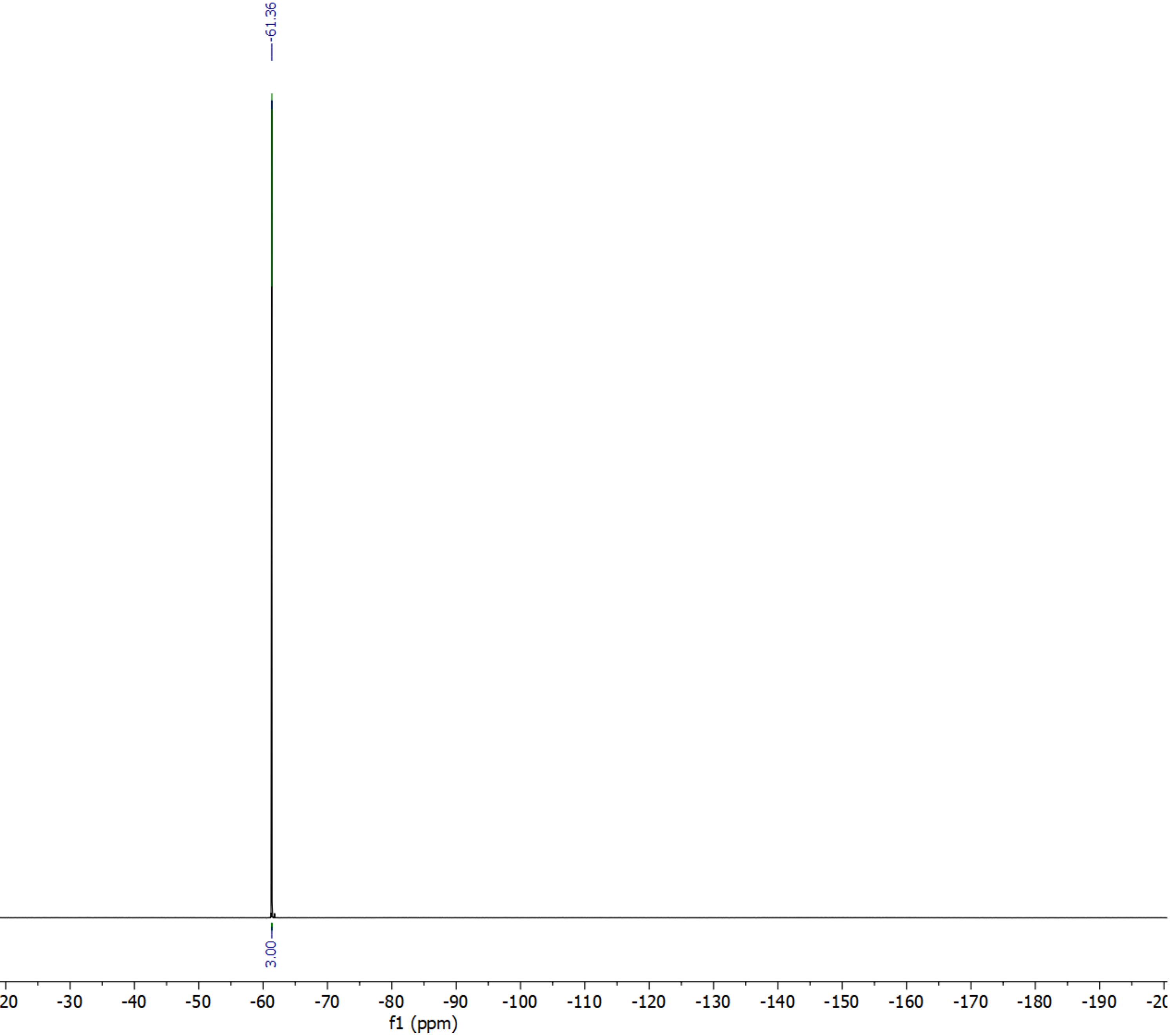

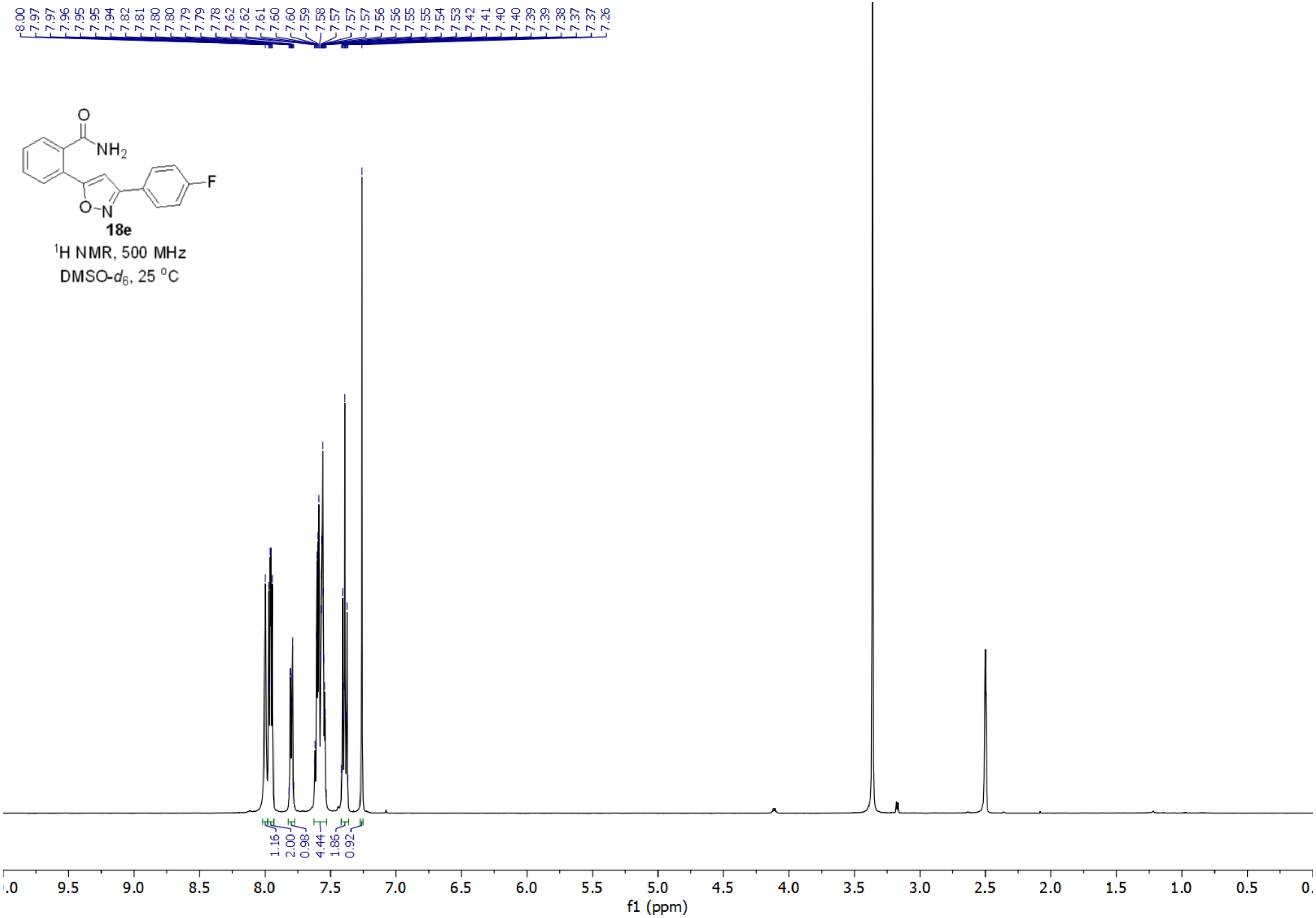

18e
¹H NMR, 500 MHz
DMSO-d6, 25 °C
f1 (ppm)

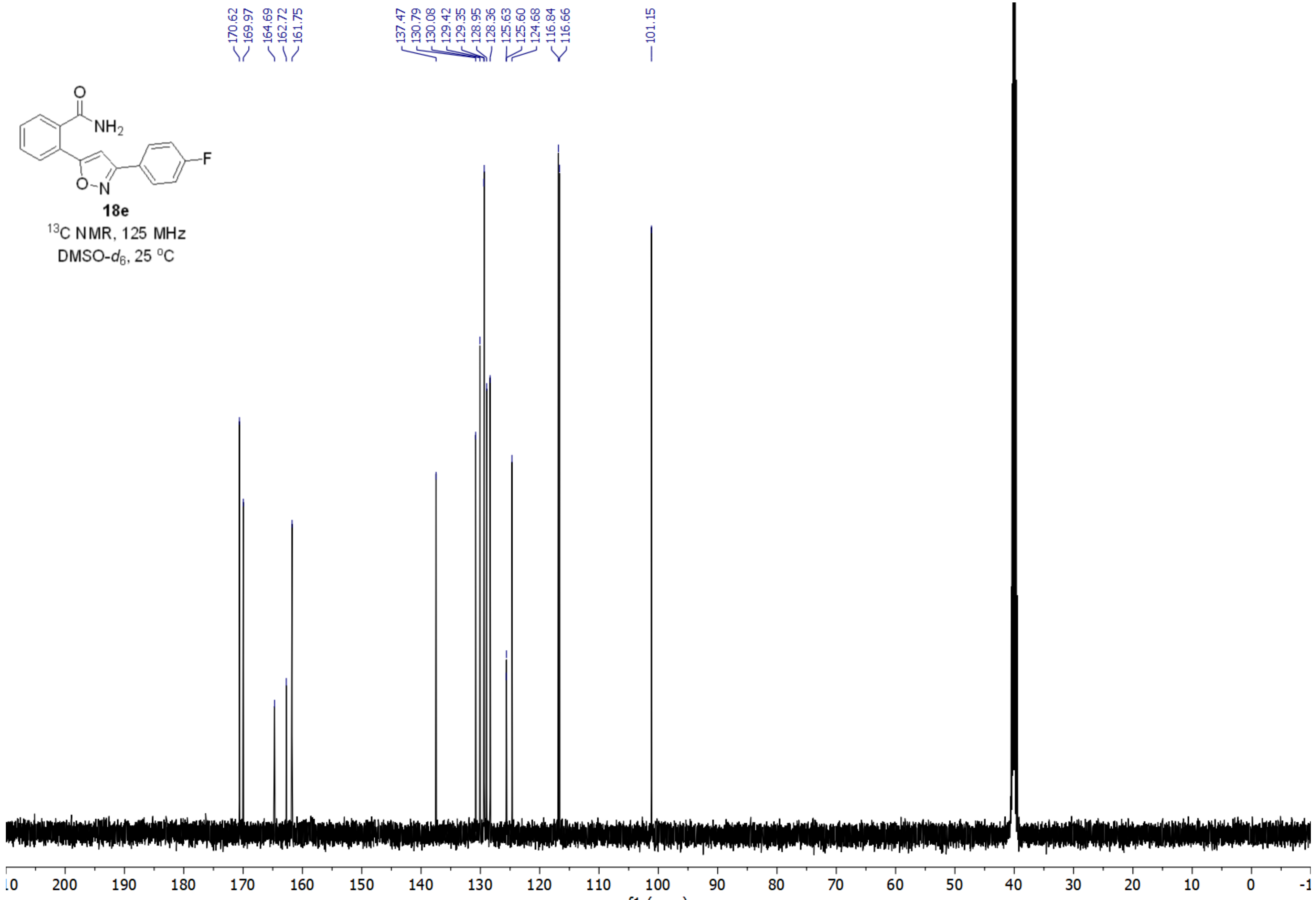

18e

^{13}C NMR, 125 MHz

DMSO-d_6, 25 °C

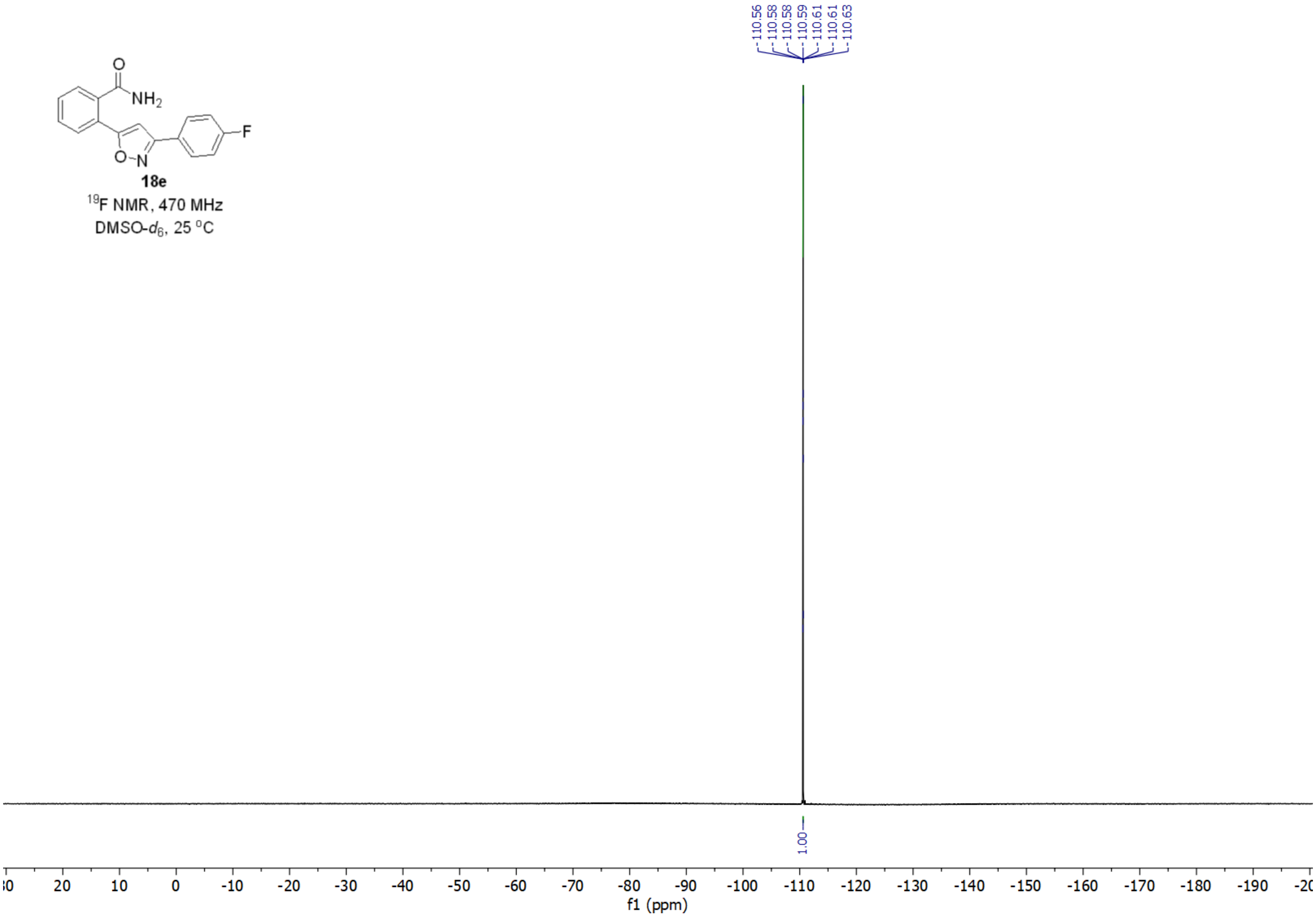
O
NH_2
F
O-N
18e
^{19}F NMR, 470 MHz
DMSO-d_6, 25 °C
-110.56
-110.58
-110.58
-110.59
-110.61
-110.61
-110.63
1.00
f1 (ppm)

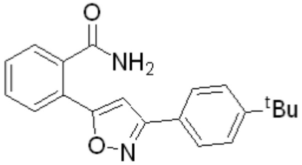

18f

^{1}H NMR, 500 MHz

DMSO-d_6, 25 °C

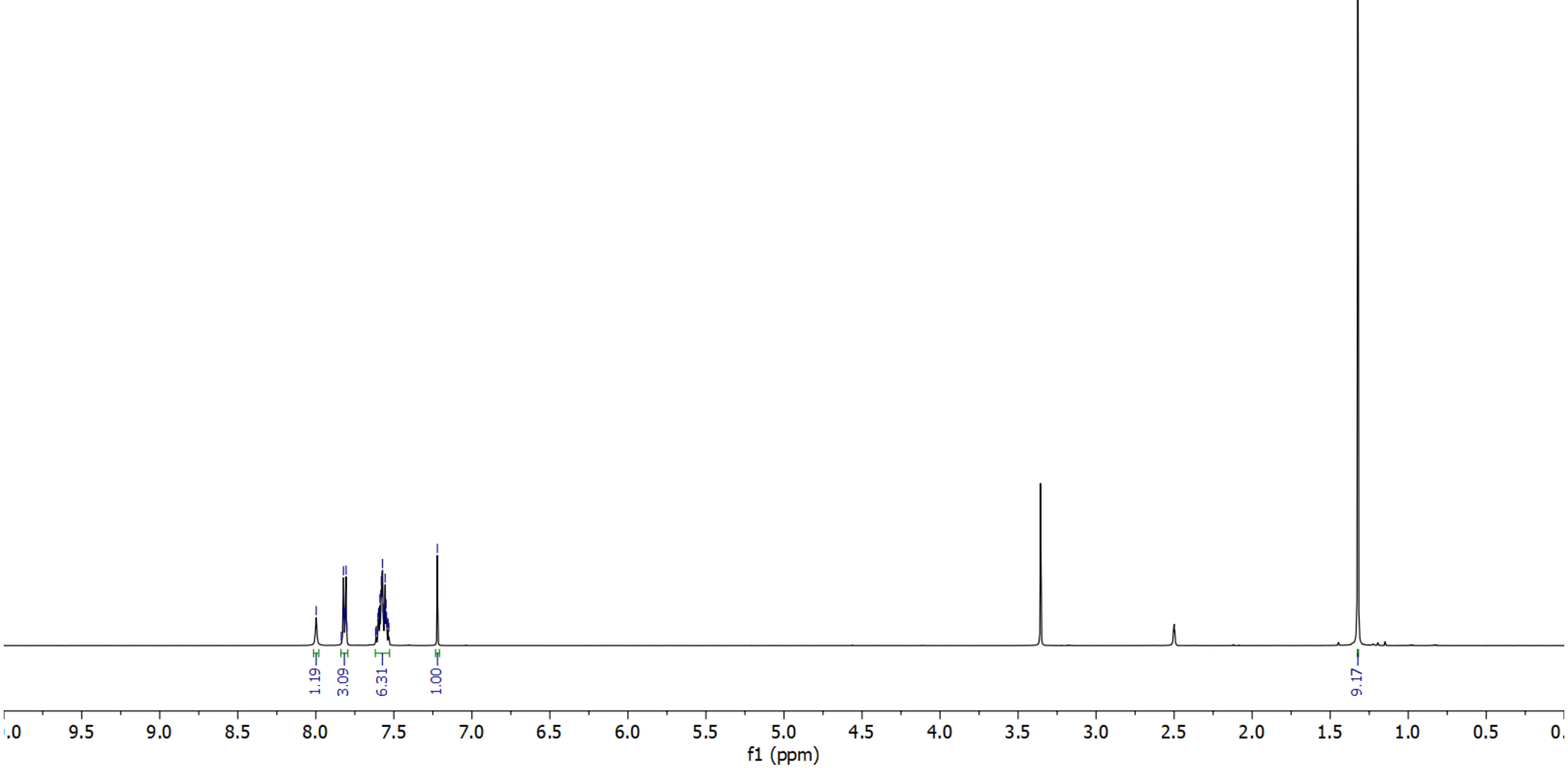

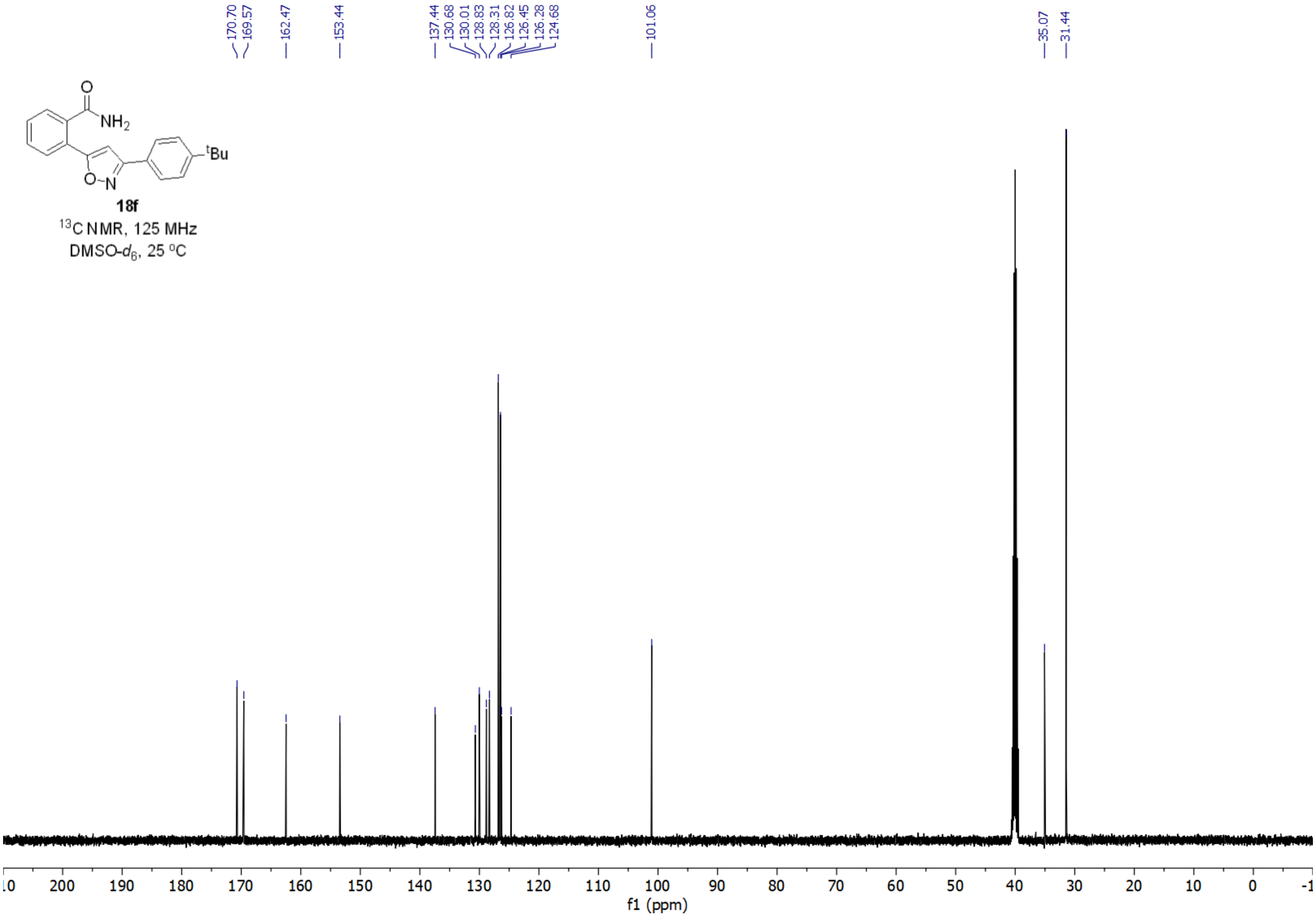

170.70
169.57
162.47
153.44
137.44
130.68
130.01
128.83
128.31
126.82
126.45
126.28
124.68
101.06
35.07
31.44
NH2
tBu
O
N
18f
13C NMR, 125 MHz
DMSO-d6, 25 °C
f1 (ppm)

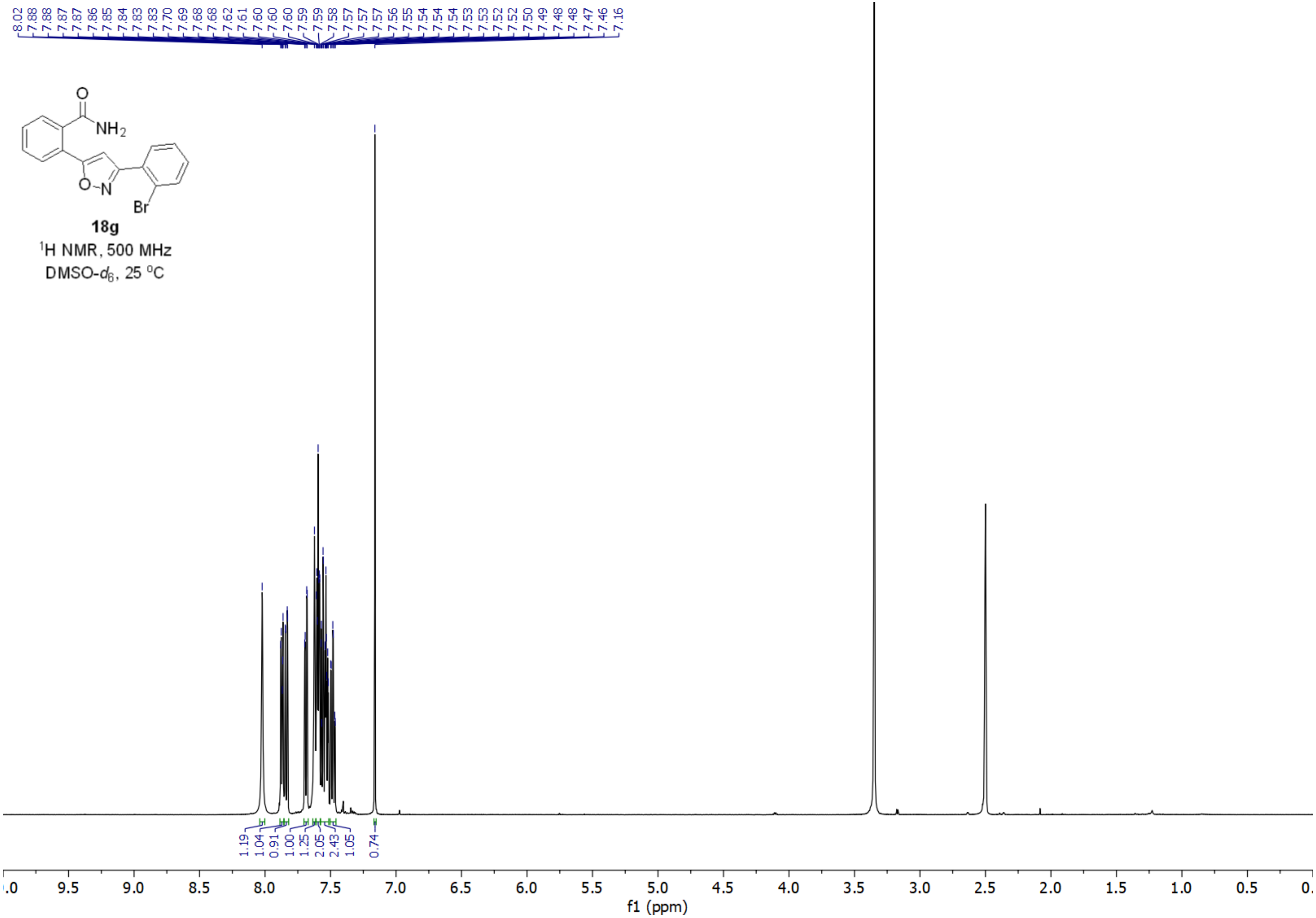

8.02 7.88 7.88 7.87 7.87 7.86 7.85 7.84 7.83 7.83 7.70 7.69 7.68 7.68 7.62 7.61 7.60 7.60 7.60 7.59 7.59 7.58 7.57 7.57 7.57 7.56 7.55 7.54 7.54 7.54 7.53 7.53 7.52 7.52 7.50 7.49 7.48 7.48 7.47 7.46 7.16
O
NH2
O-N
Br
18g
1H NMR, 500 MHz
DMSO-d6, 25 °C
1.19 1.04 0.91 1.00 1.25 2.05 2.43 1.05 0.74
9.5 9.0 8.5 8.0 7.5 7.0 6.5 6.0 5.5 5.0 4.5 4.0 3.5 3.0 2.5 2.0 1.5 1.0 0.5
f1 (ppm)

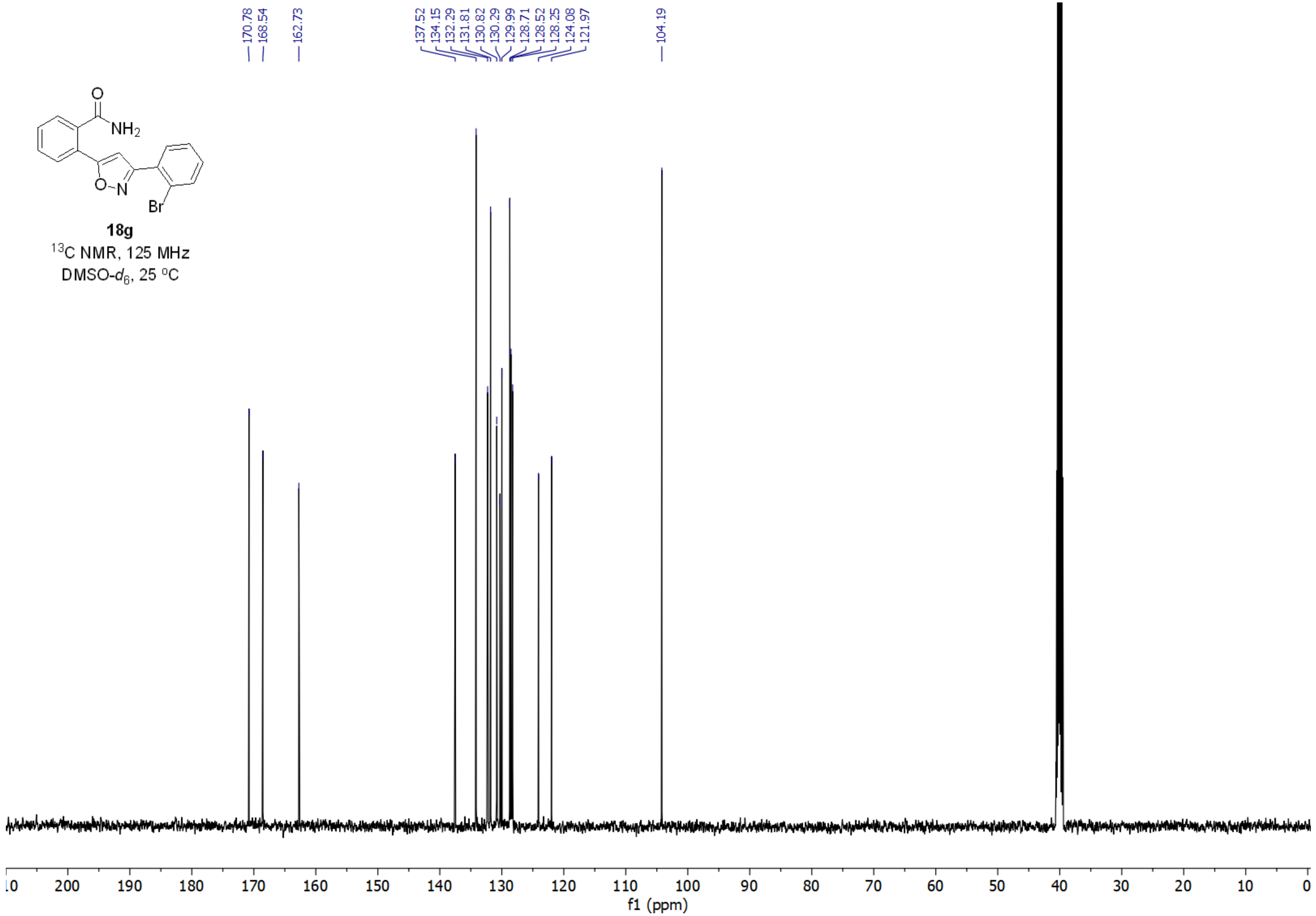
170.78
168.54
162.73
137.52
134.15
132.29
131.81
130.82
130.29
129.99
128.71
128.52
128.25
124.08
121.97
104.19
O
NH2
O
N
Br
18g
13C NMR, 125 MHz
DMSO-d6, 25 °C
f1 (ppm)

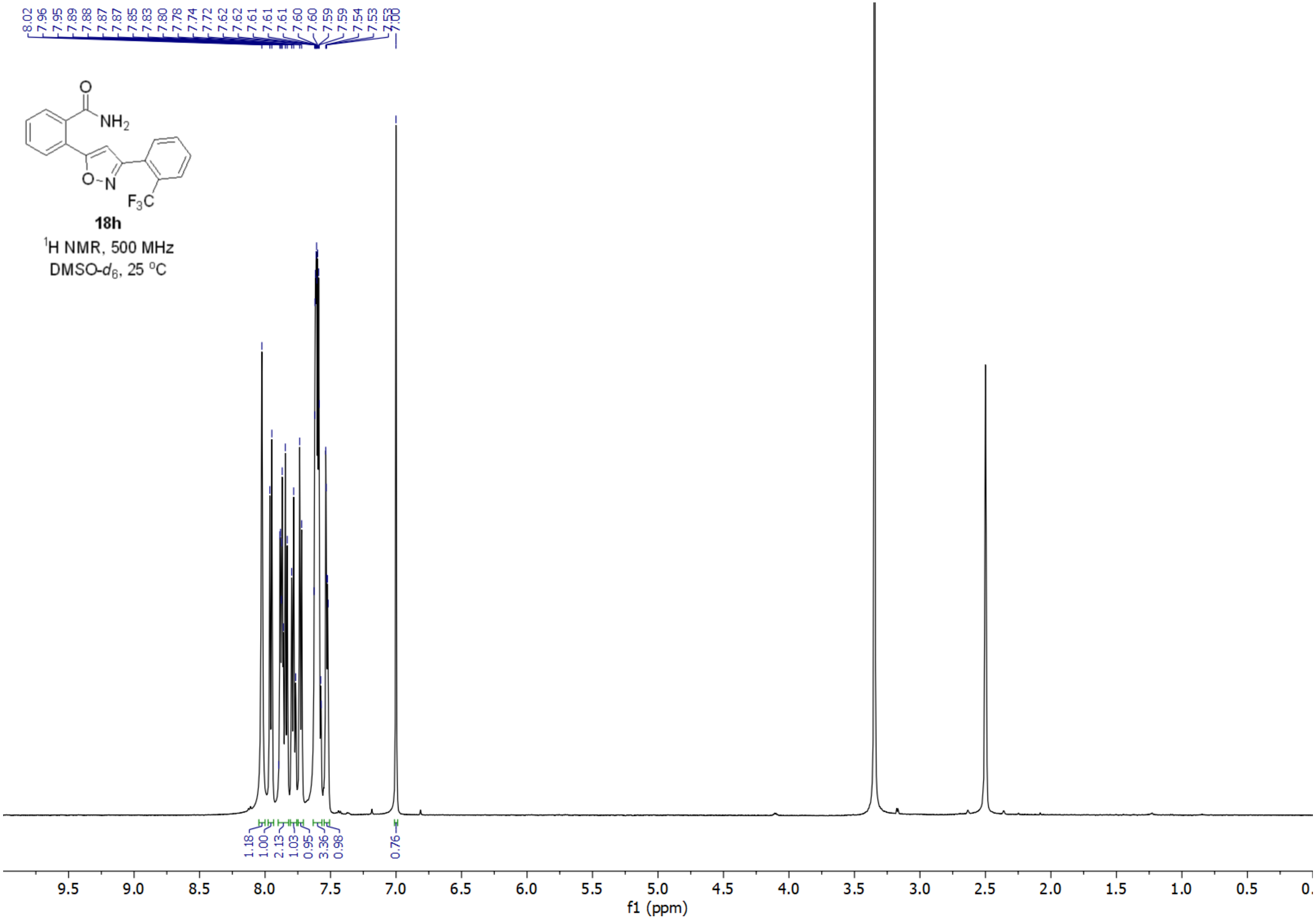
8.02 7.96 7.95 7.89 7.88 7.87 7.87 7.85 7.83 7.80 7.78 7.74 7.72 7.62 7.62 7.61 7.61 7.61 7.60 7.60 7.59 7.59 7.54 7.53 7.53 7.00
O
NH2
O-N
F3C
18h
1H NMR, 500 MHz
DMSO-d6, 25 °C
1.18 1.00 2.13 1.03 0.95 3.36 0.98 0.76
9.5 9.0 8.5 8.0 7.5 7.0 6.5 6.0 5.5 5.0 4.5 4.0 3.5 3.0 2.5 2.0 1.5 1.0 0.5 0.
f1 (ppm)

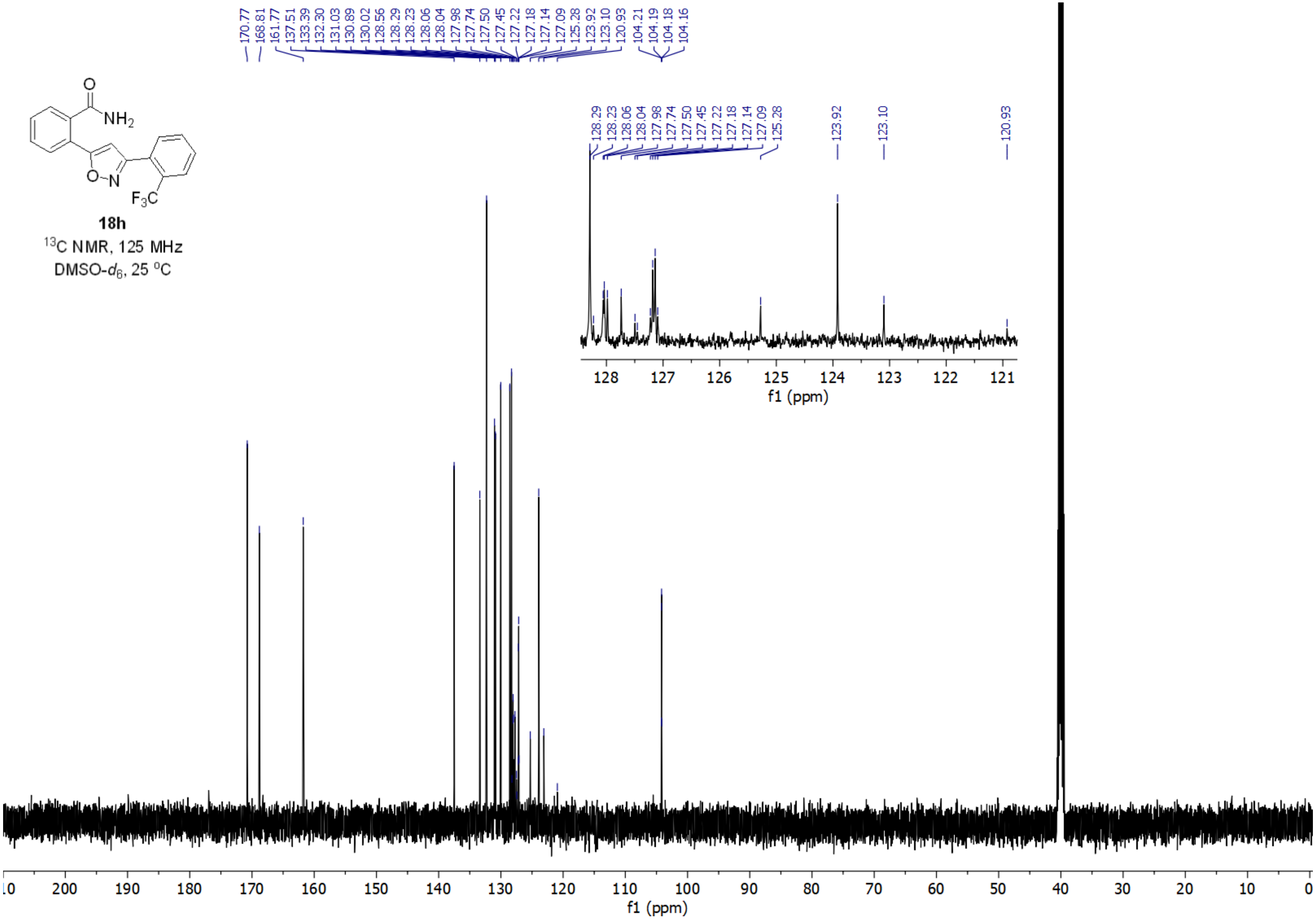

18h

^{13}C NMR, 125 MHz

DMSO-d_6, 25 °C

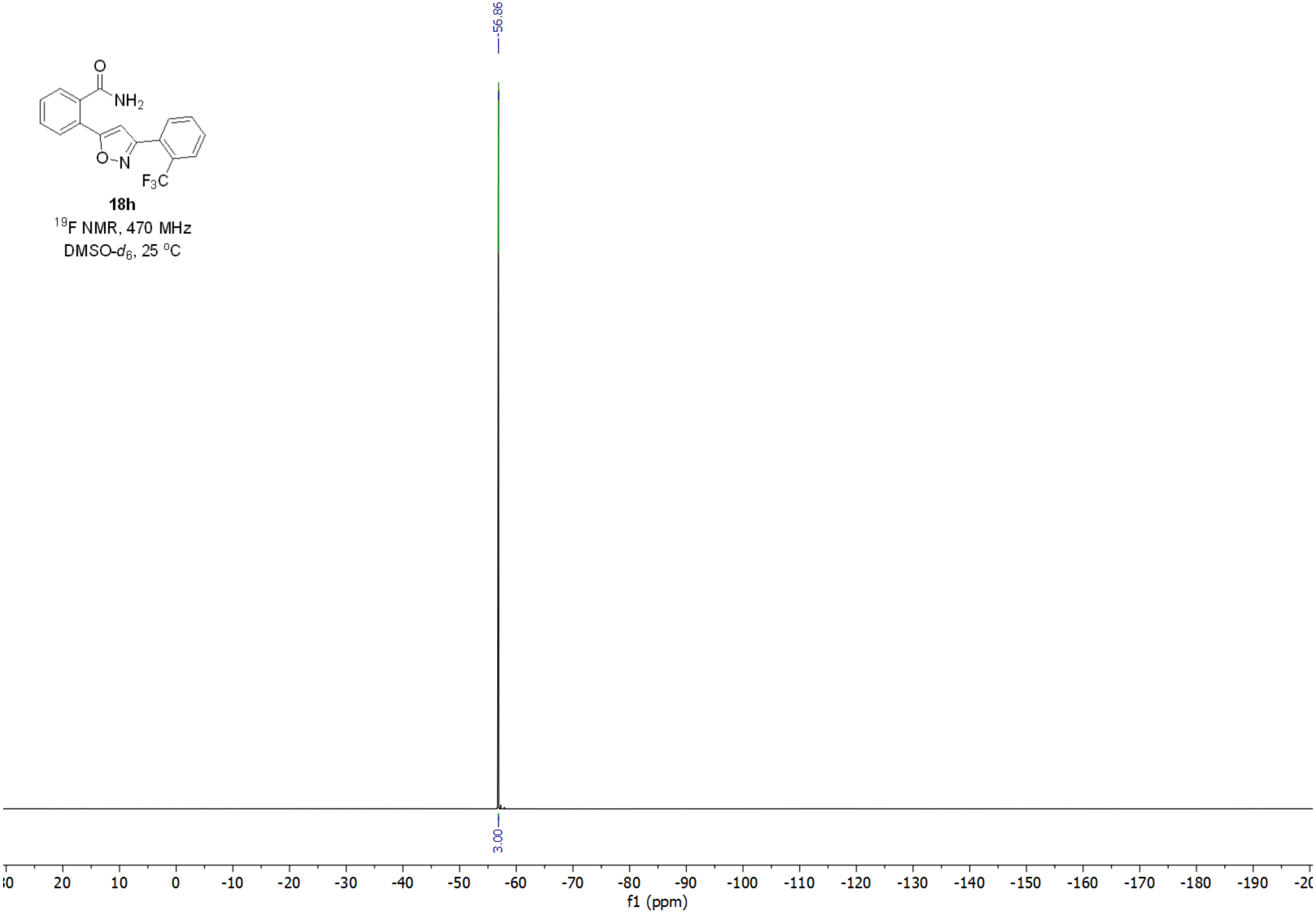
18h
19F NMR, 470 MHz
DMSO-d6, 25 °C
-56.86
3.00
f1 (ppm)

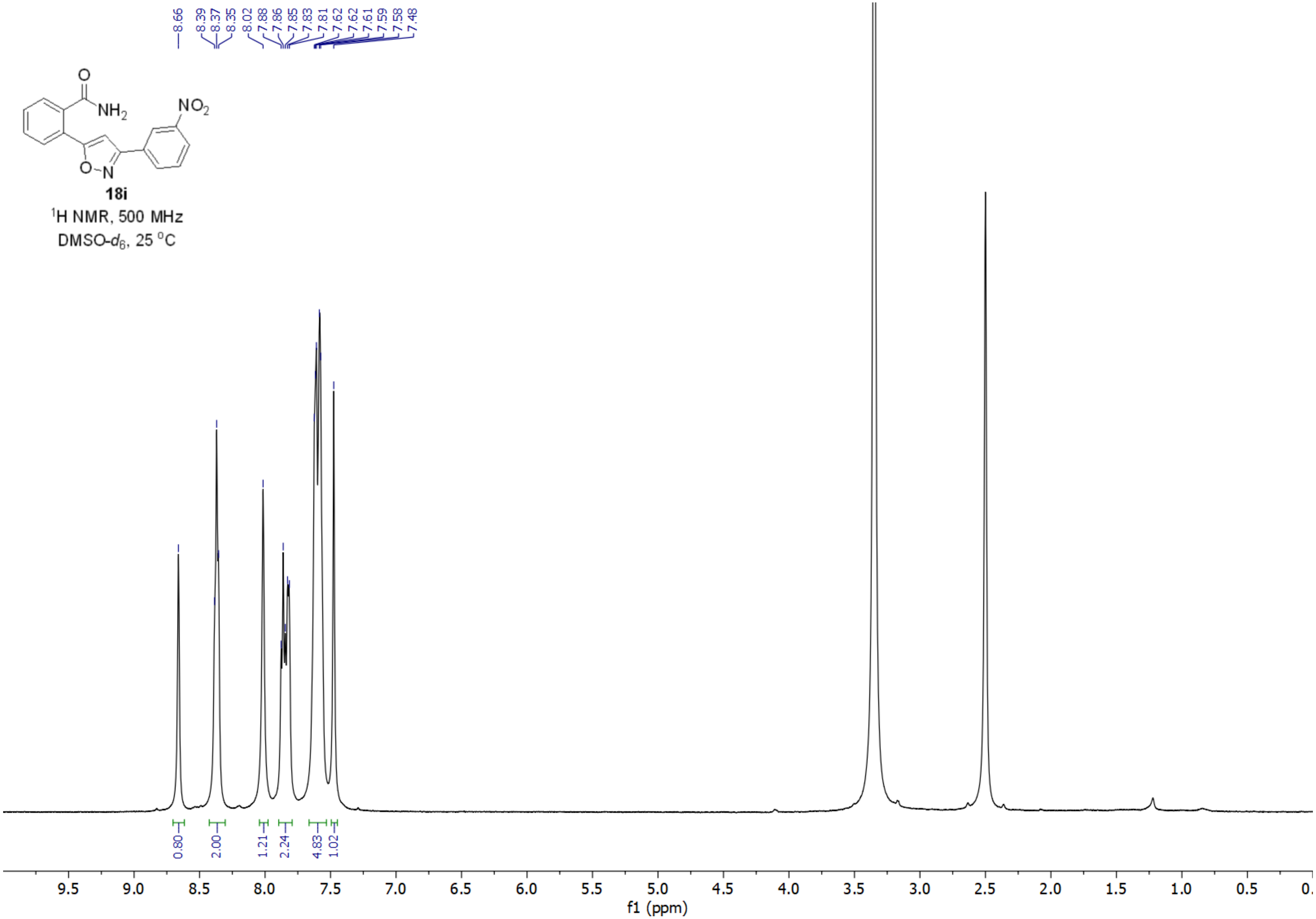
8.66
8.39
8.37
8.35
8.02
7.88
7.86
7.85
7.83
7.81
7.62
7.62
7.61
7.59
7.58
7.48
O
NH2
NO2
O
N
18i
1H NMR, 500 MHz
DMSO-d6, 25 °C
0.80
2.00
1.21
2.24
4.83
1.02
9.5
9.0
8.5
8.0
7.5
7.0
6.5
6.0
5.5
5.0
4.5
4.0
3.5
3.0
2.5
2.0
1.5
1.0
0.5
f1 (ppm)

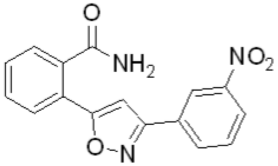

18i

^{13}C NMR, 125 MHz

DMSO-d_6, 25 ^{o}C

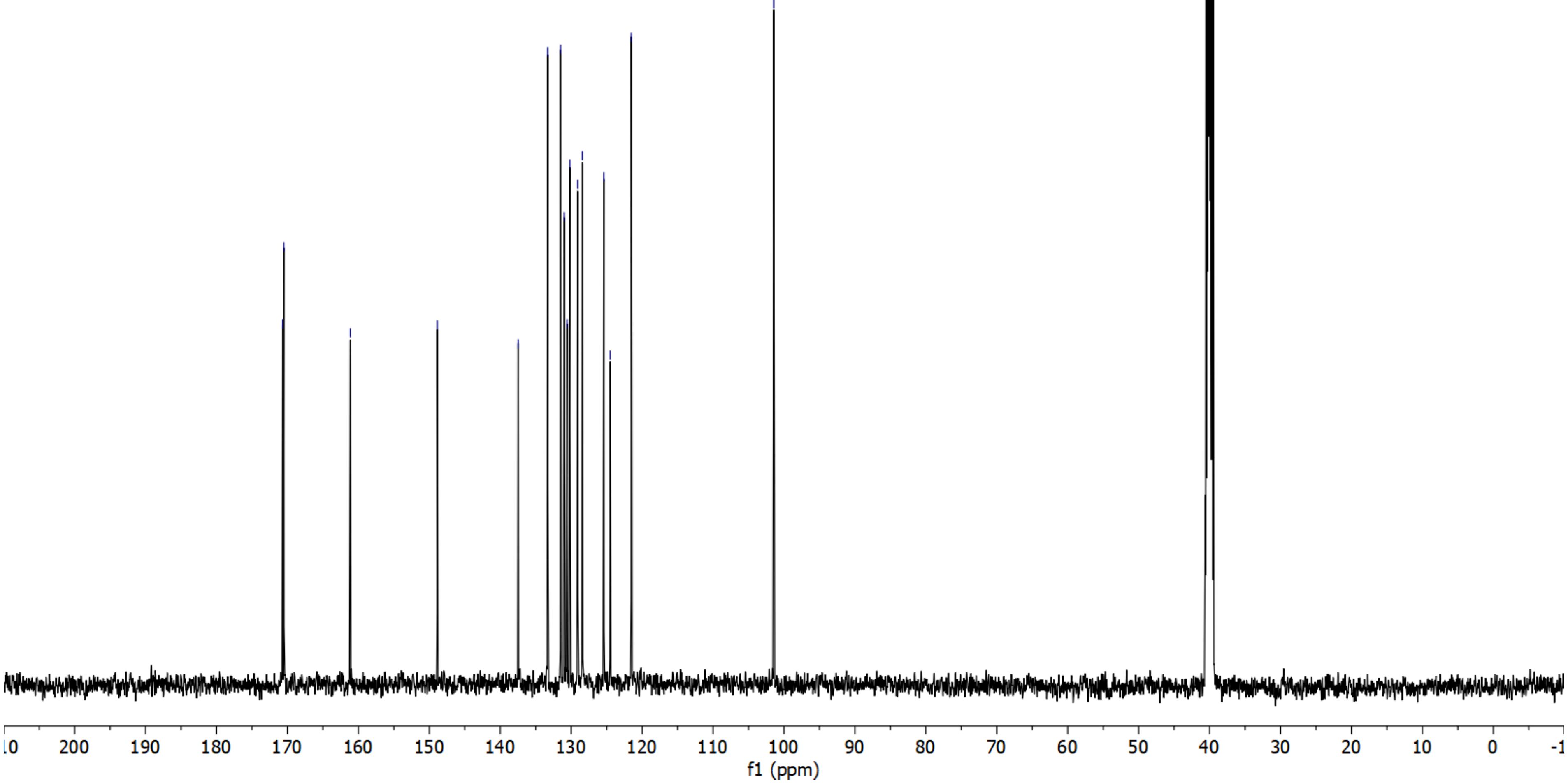

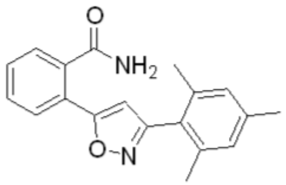

18j

^{1}H NMR, 500 MHz

$CDCl_3$, 25 ^{o}C

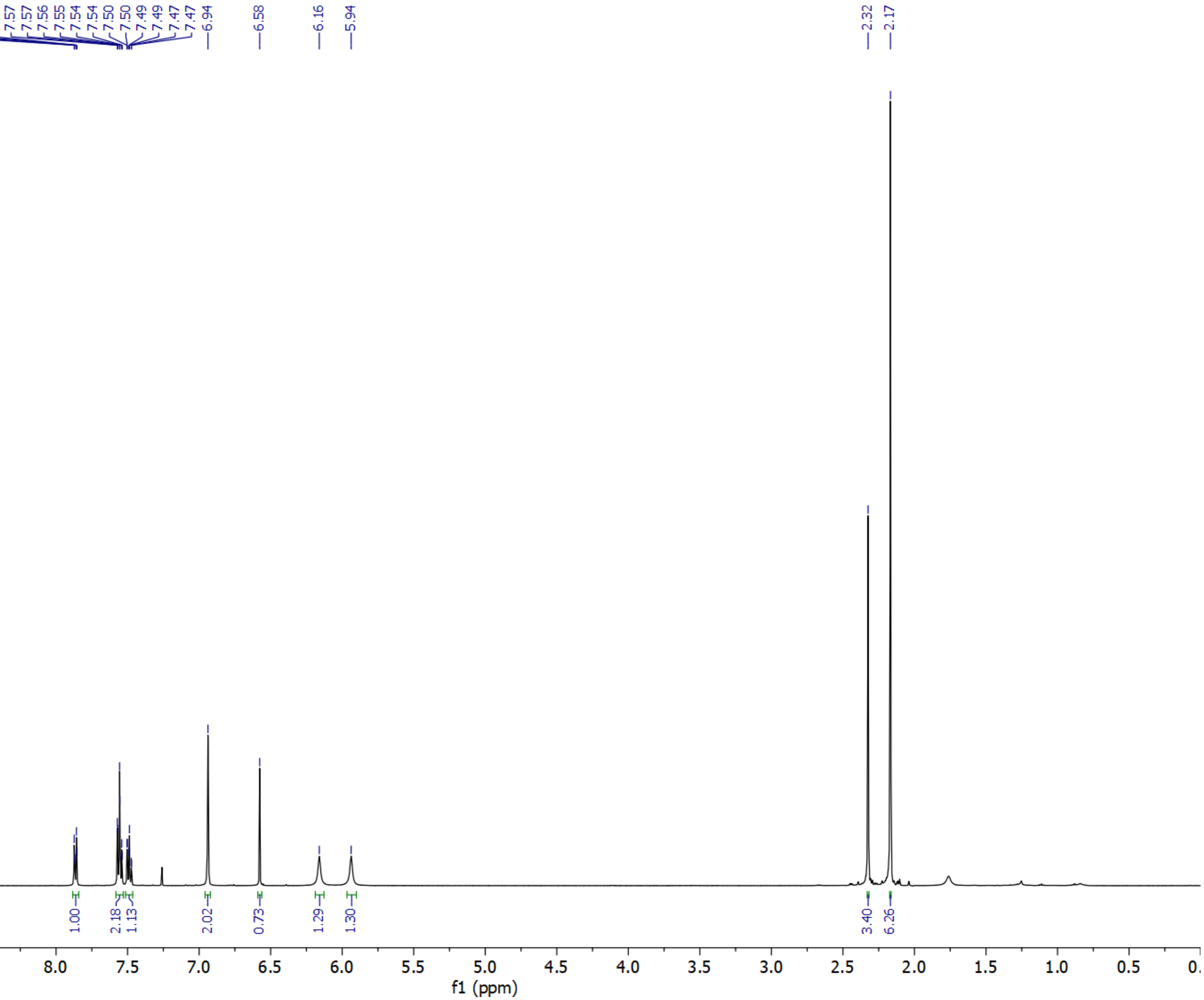

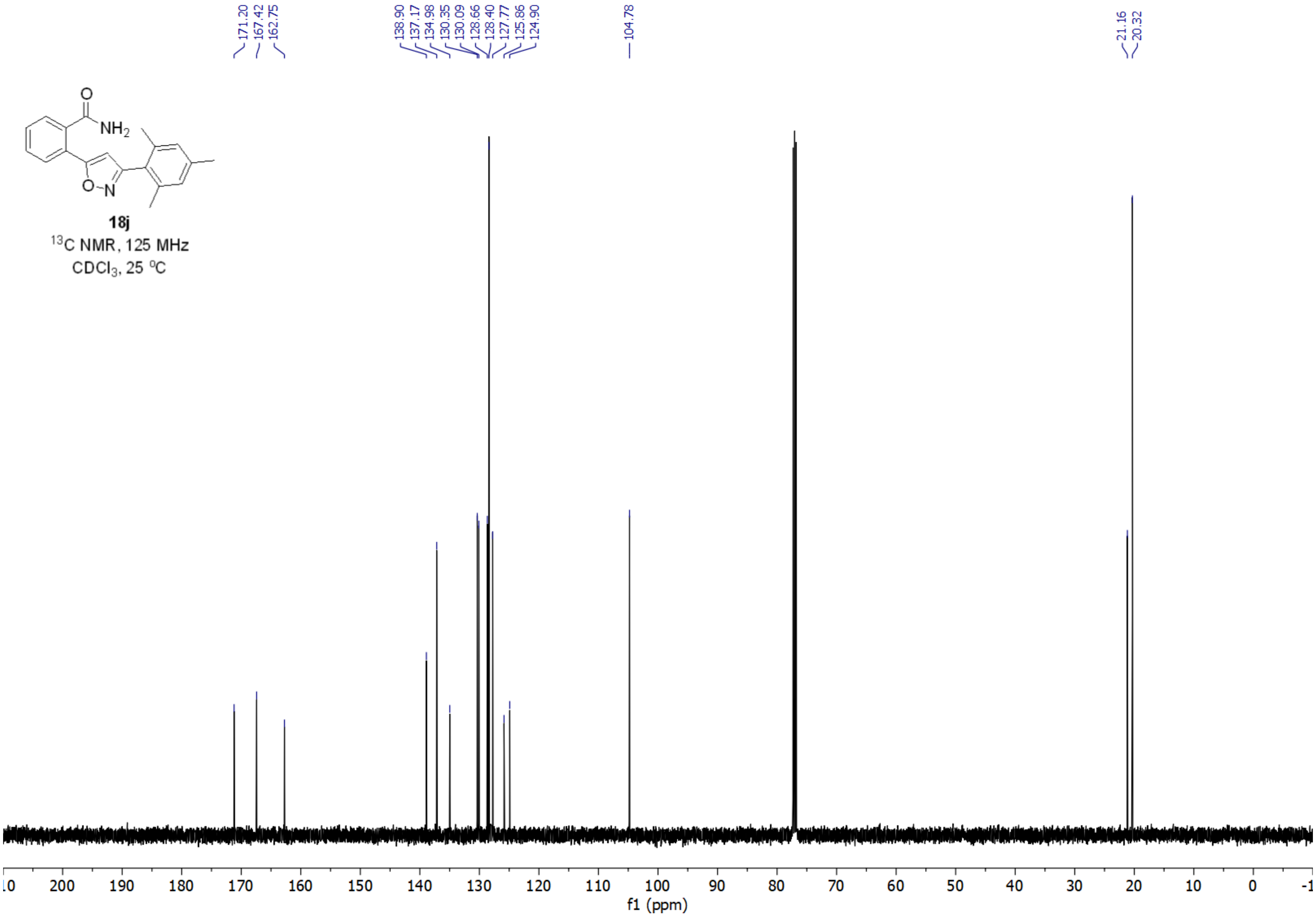

171.20
167.42
162.75
138.90
137.17
134.98
130.35
130.09
128.66
128.40
127.77
125.86
124.90
104.78
21.16
20.32
O
NH2
O
N
18j
13C NMR, 125 MHz
CDCl3, 25 °C
200
190
180
170
160
150
140
130
120
110
100
90
80
70
60
50
40
30
20
10
0
f1 (ppm)

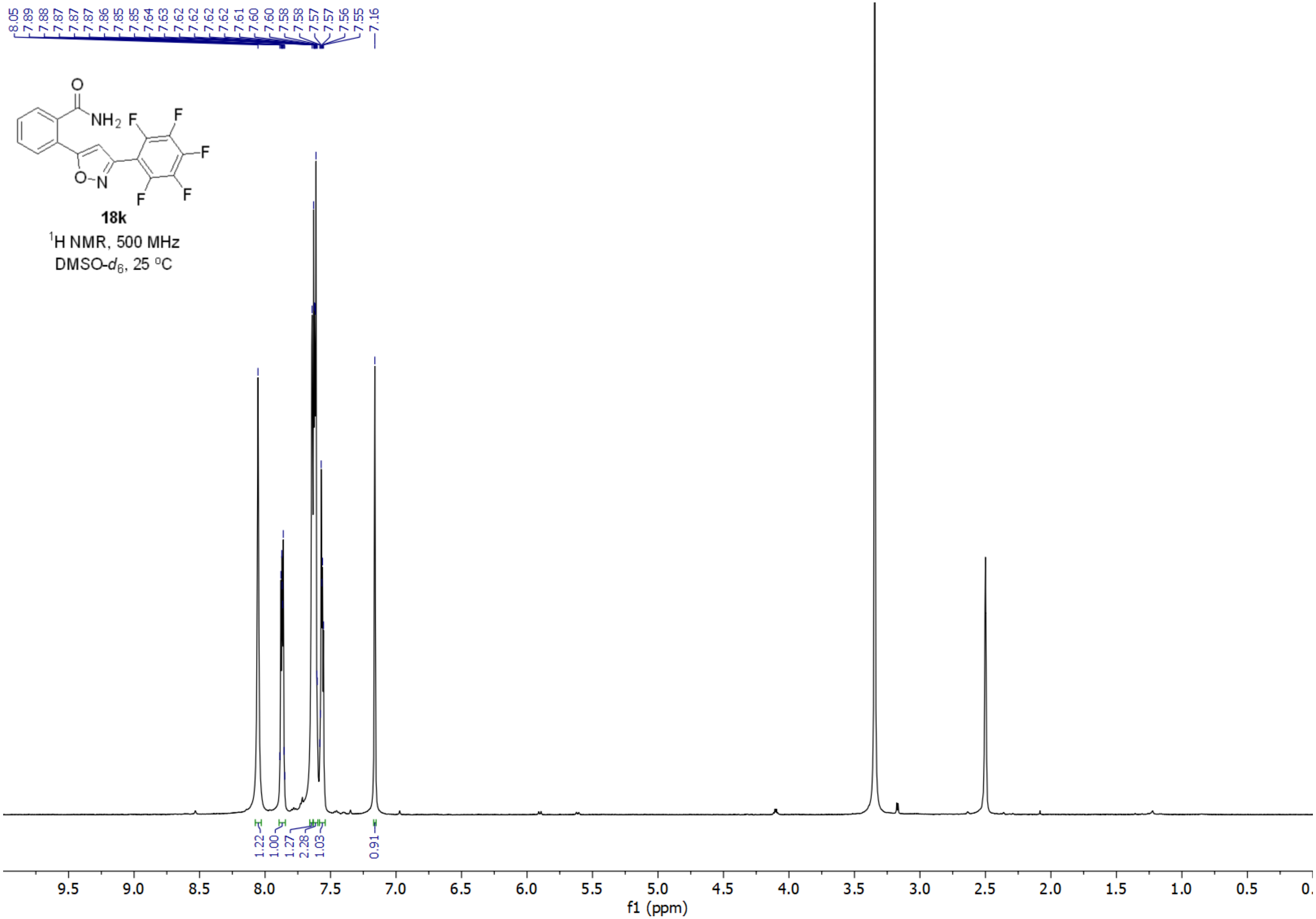
8.05
7.89
7.88
7.87
7.87
7.87
7.86
7.85
7.85
7.64
7.63
7.62
7.62
7.62
7.62
7.61
7.60
7.60
7.58
7.58
7.57
7.57
7.56
7.55
7.16
18k
^{1}H NMR, 500 MHz
DMSO-d_6, 25 °C
1.22
1.00
1.27
2.28
1.03
0.91
9.5
9.0
8.5
8.0
7.5
7.0
6.5
6.0
5.5
5.0
4.5
4.0
3.5
3.0
2.5
2.0
1.5
1.0
0.5
f1 (ppm)

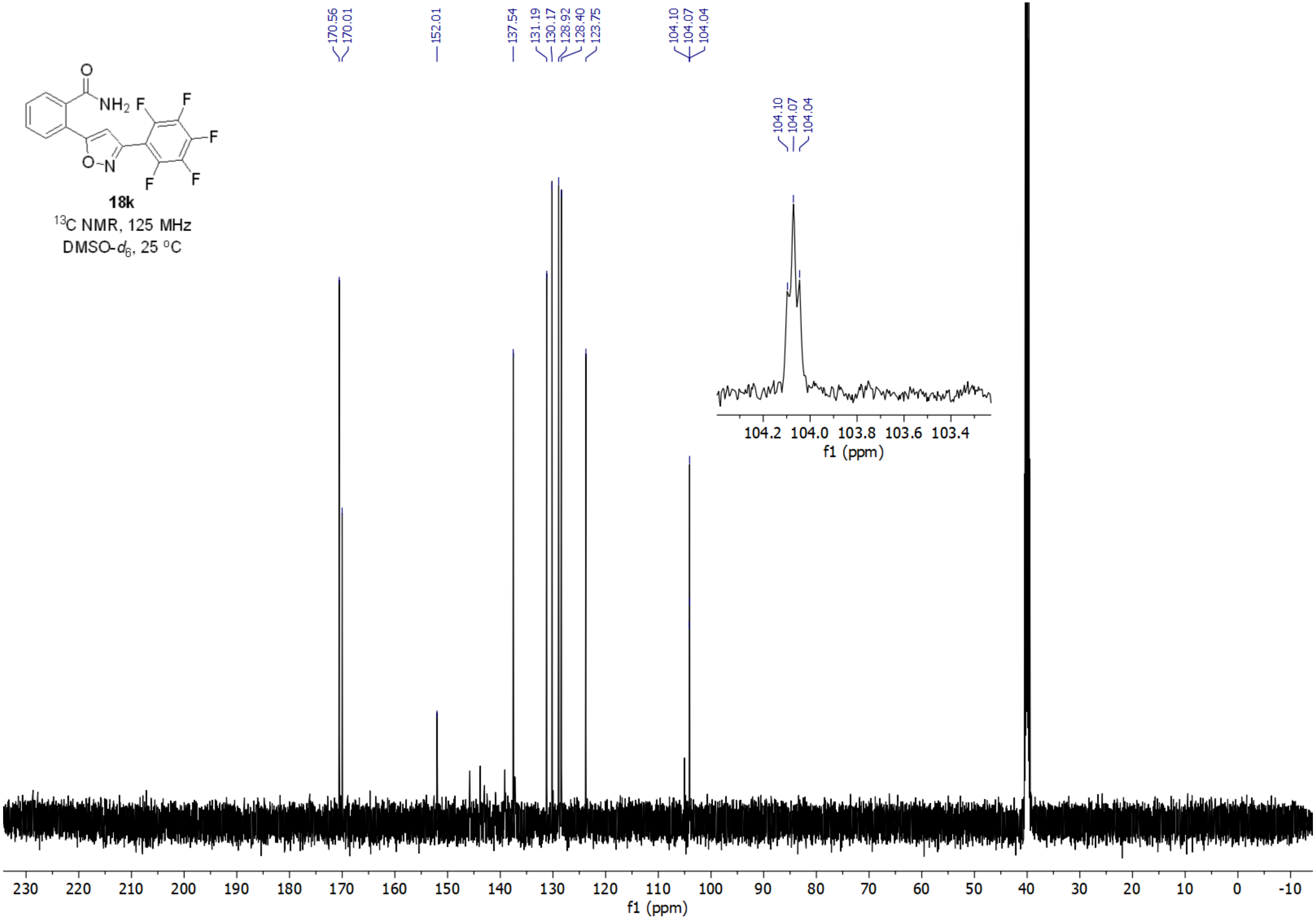
170.56
170.01
152.01
137.54
131.19
130.17
128.92
128.40
123.75
104.10
104.07
104.04
O
NH2
F
F
F
F
F
O
N
18k
13C NMR, 125 MHz
DMSO-d6, 25 °C
104.2 104.0 103.8 103.6 103.4
f1 (ppm)
230 220 210 200 190 180 170 160 150 140 130 120 110 100 90 80 70 60 50 40 30 20 10 0 -10
f1 (ppm)

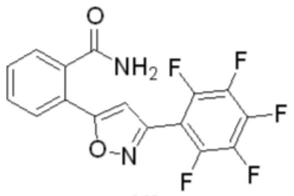

18k

^{19}F NMR, 470 MHz

DMSO-d_6, 25 °C

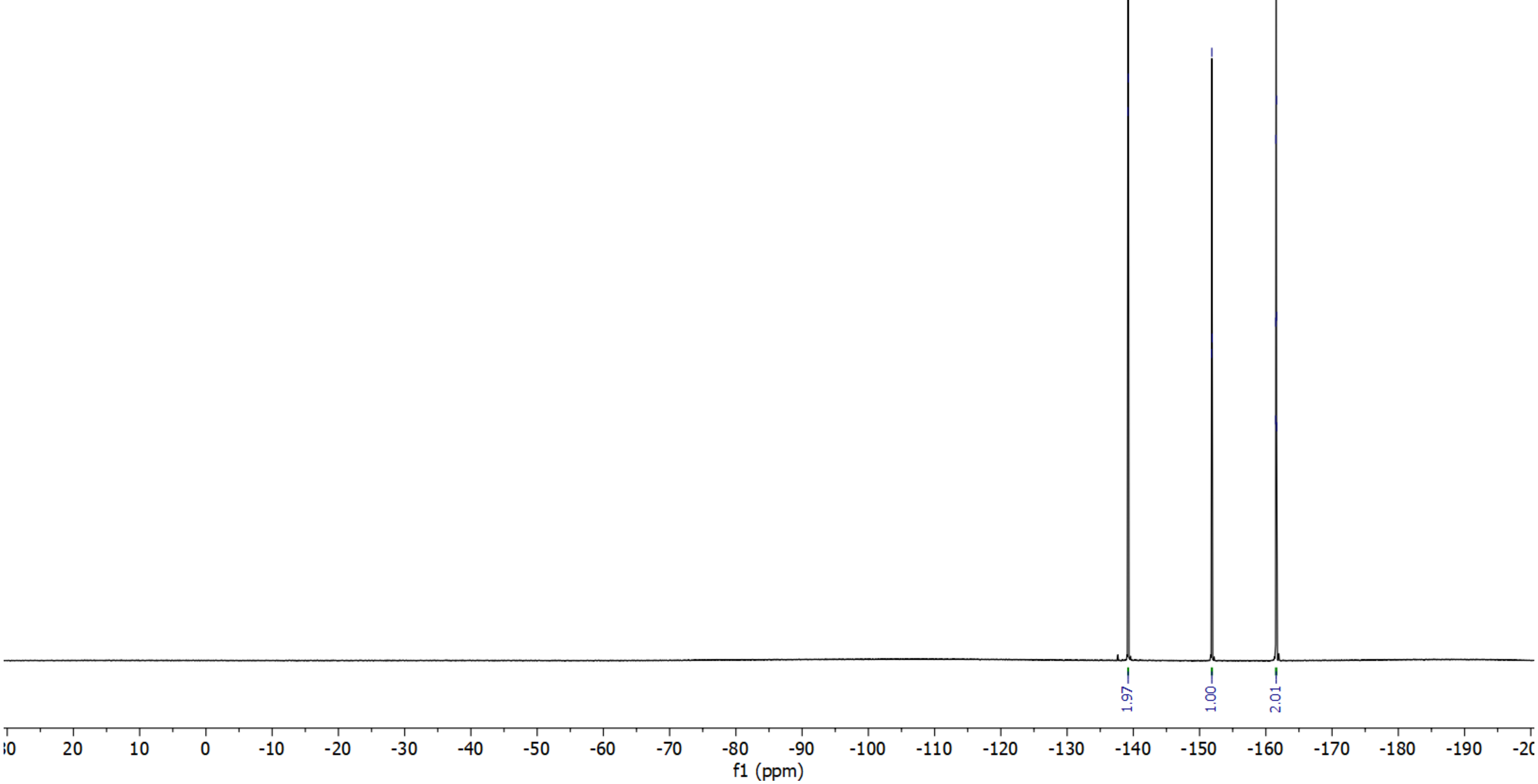

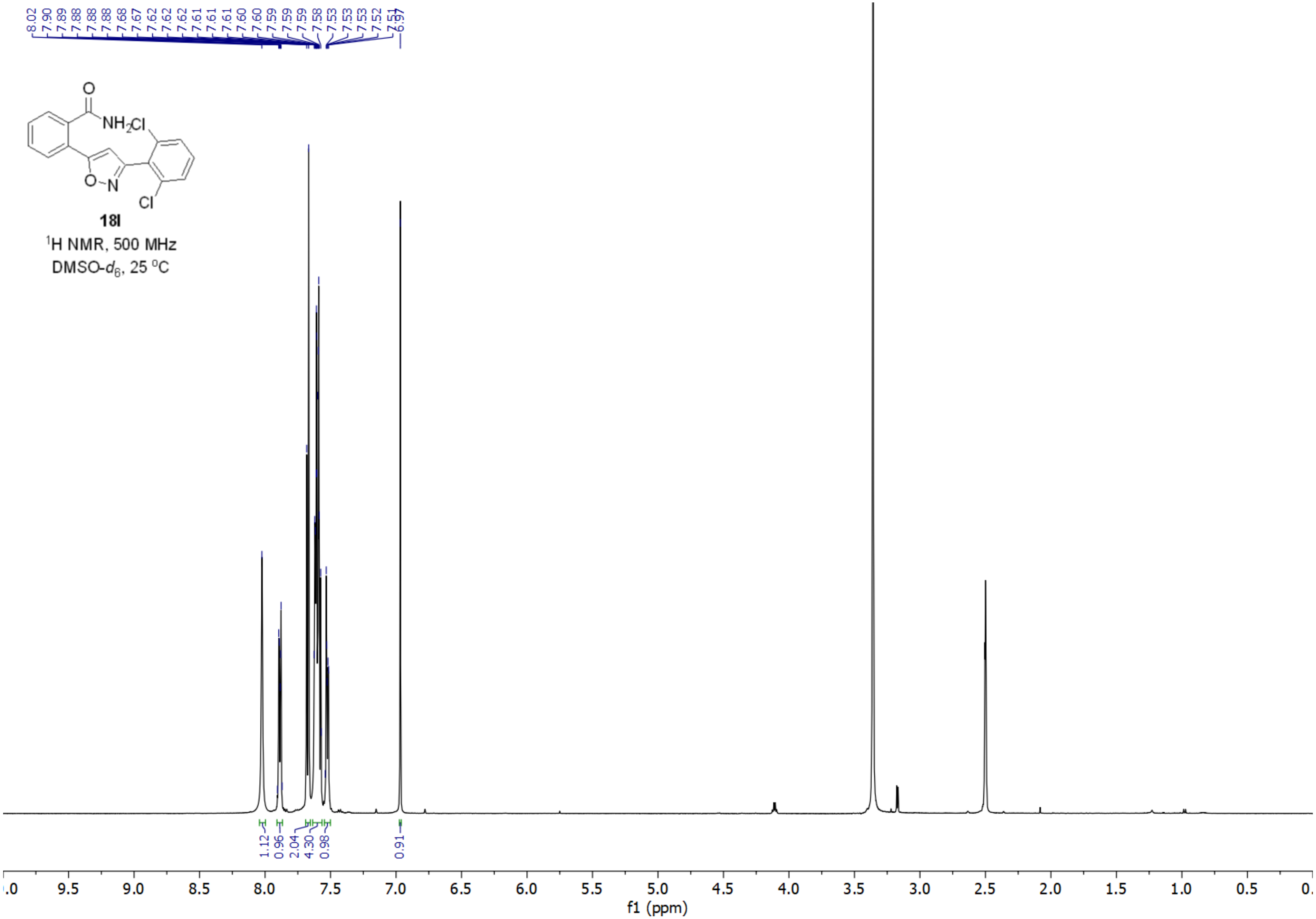

18l

^{1}H NMR, 500 MHz

DMSO-d_6, 25 ^{o}C

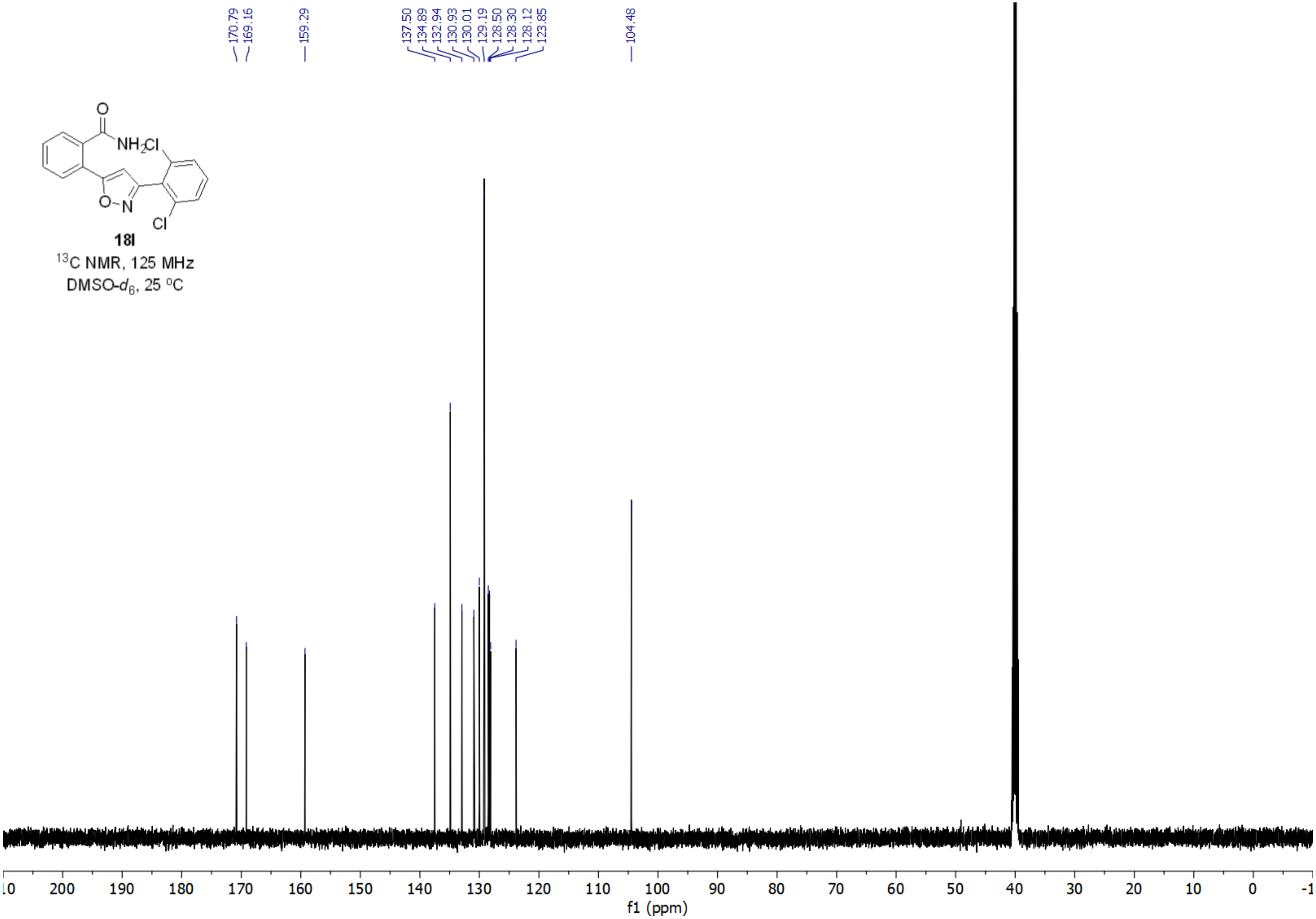
170.79
169.16
159.29
137.50
134.89
132.94
130.93
130.01
129.19
128.50
128.30
128.12
123.85
104.48
O
NH2
Cl
O
N
Cl
18l
13C NMR, 125 MHz
DMSO-d6, 25 °C
200 190 180 170 160 150 140 130 120 110 100 90 80 70 60 50 40 30 20 10 0
f1 (ppm)

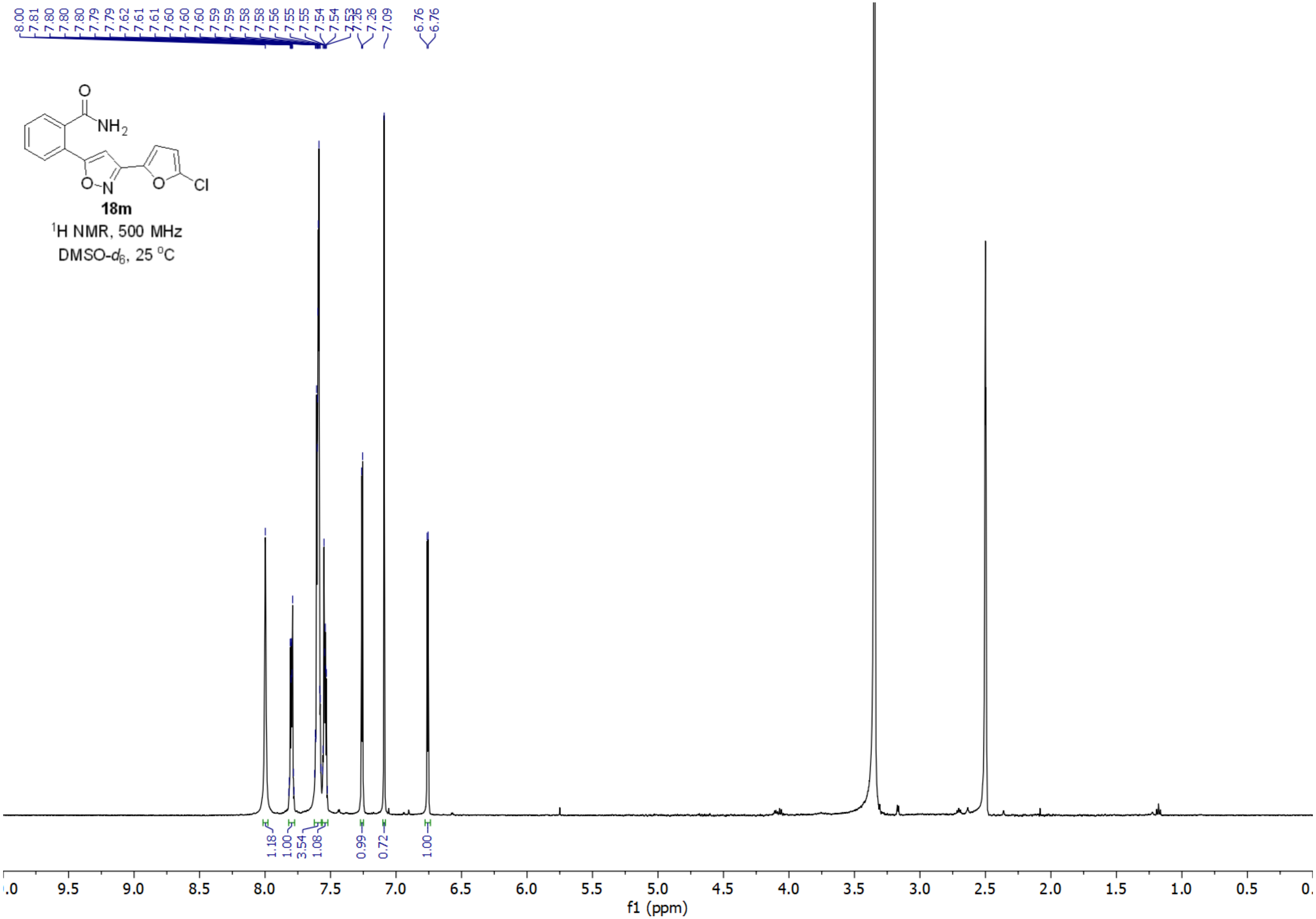

18m
¹H NMR, 500 MHz
DMSO-d6, 25 °C
f1 (ppm)

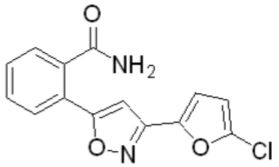

18m

^{13}C NMR, 125 MHz

DMSO-d_6, 25 °C

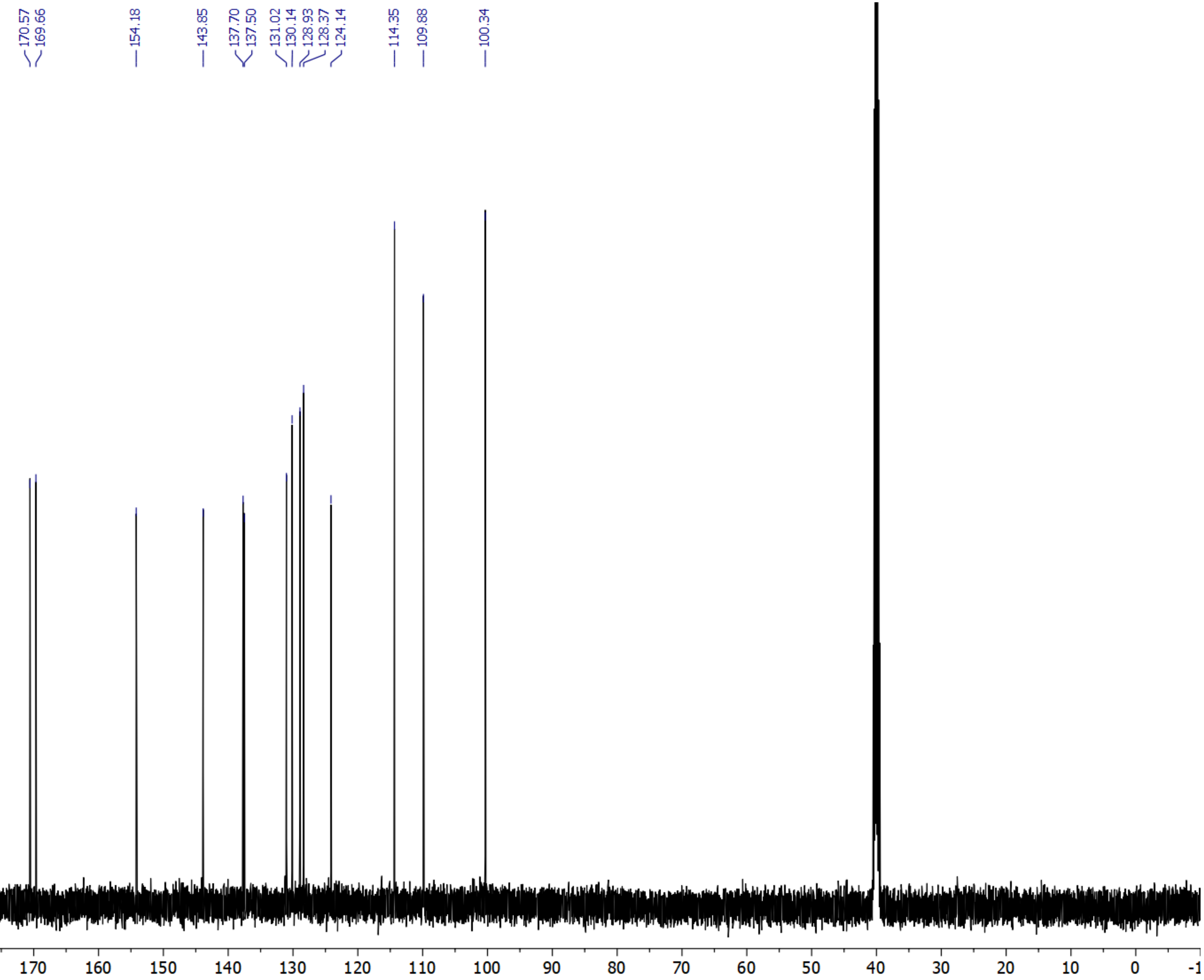

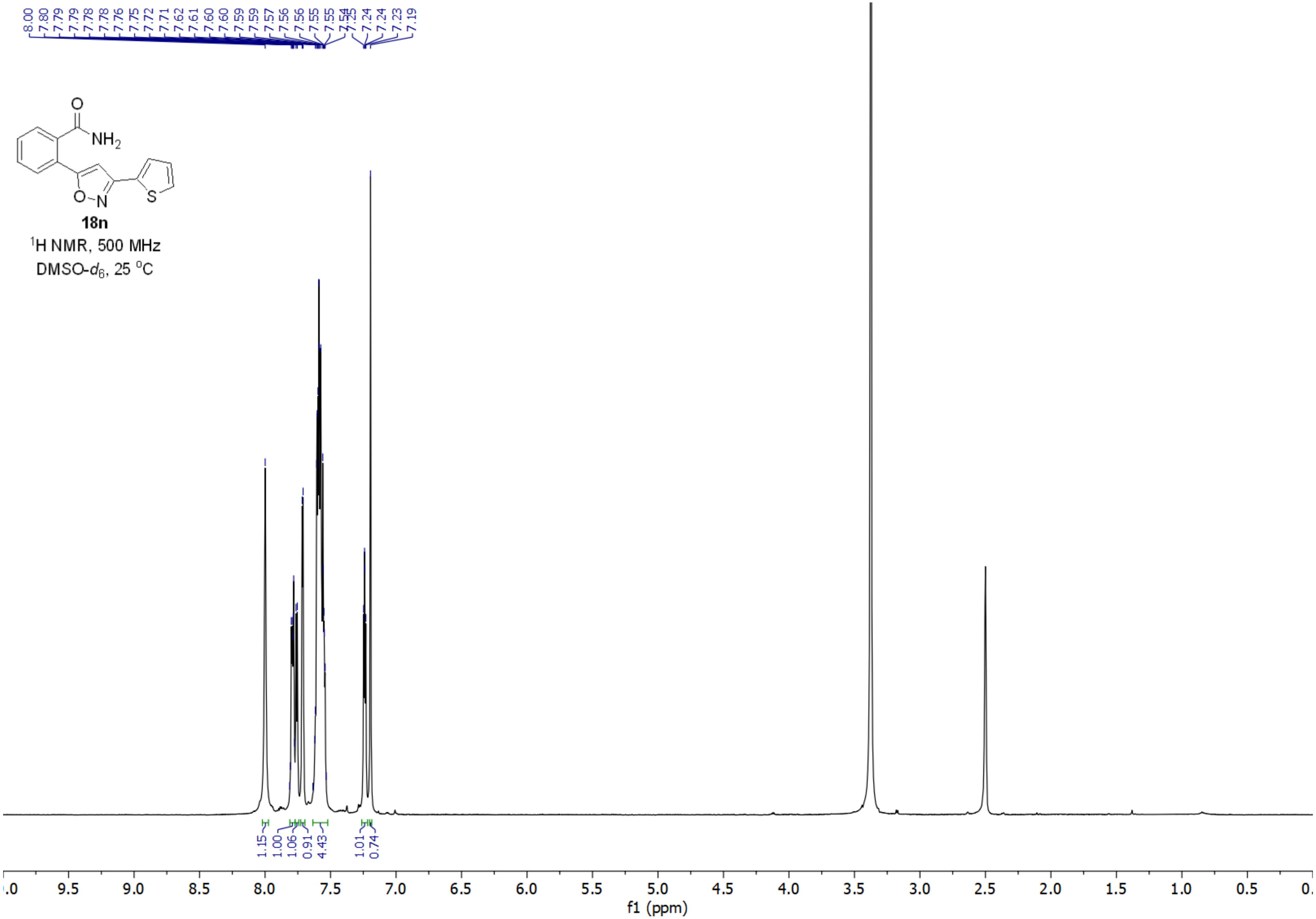

8.00 7.80 7.79 7.79 7.78 7.76 7.75 7.72 7.71 7.62 7.61 7.60 7.60 7.59 7.59 7.57 7.56 7.56 7.55 7.55 7.54 7.25 7.24 7.24 7.23 7.19
O
NH2
O
N
S
18n
1H NMR, 500 MHz
DMSO-d6, 25 °C
1.15 1.00 1.06 0.91 4.43 1.01 0.74
9.5 9.0 8.5 8.0 7.5 7.0 6.5 6.0 5.5 5.0 4.5 4.0 3.5 3.0 2.5 2.0 1.5 1.0 0.5
f1 (ppm)

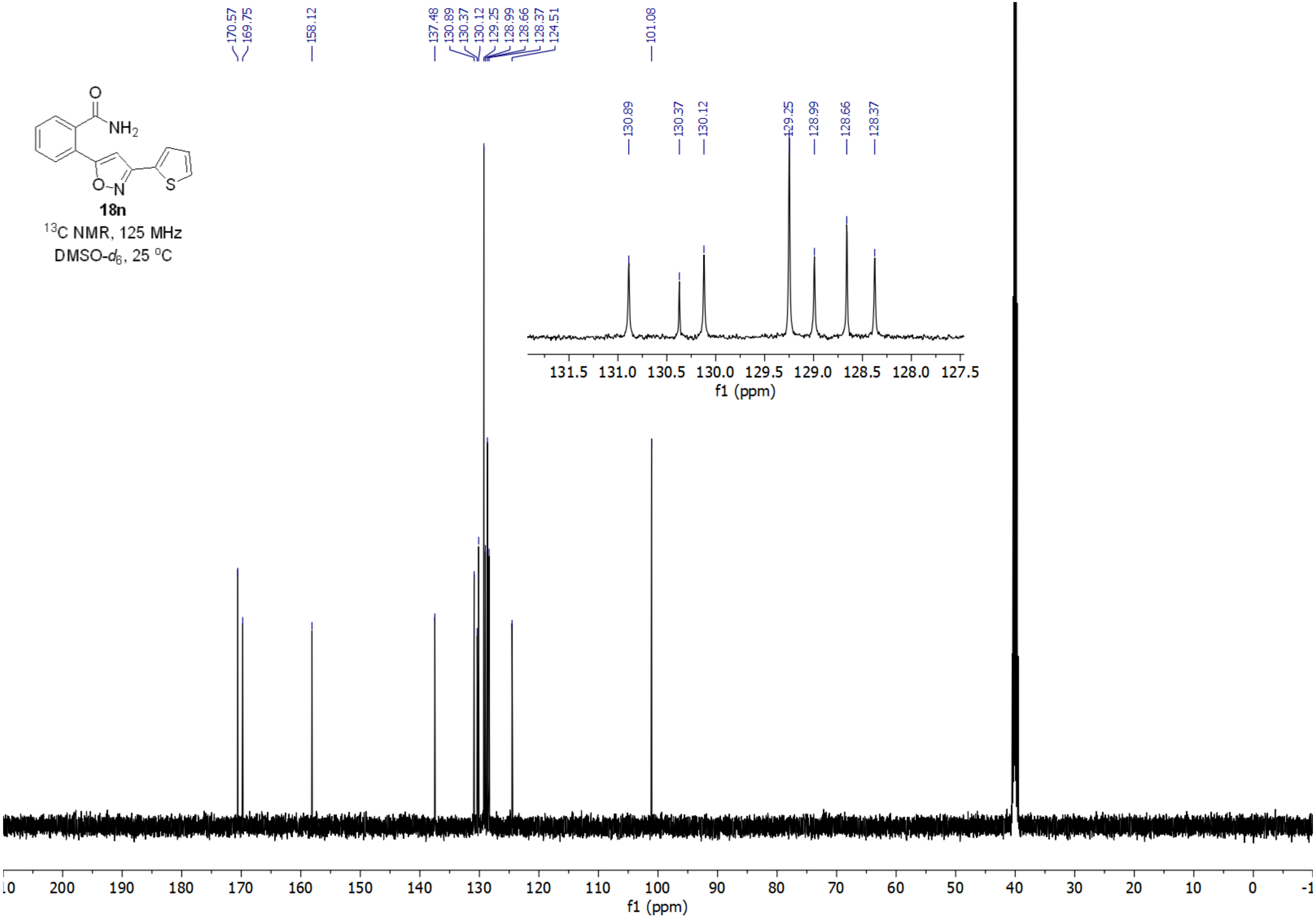

18n
^{13}C NMR, 125 MHz
DMSO-d_6, 25 °C
170.57
169.75
158.12
137.48
130.89
130.37
130.12
129.25
128.99
128.66
128.37
124.51
101.08
f1 (ppm)

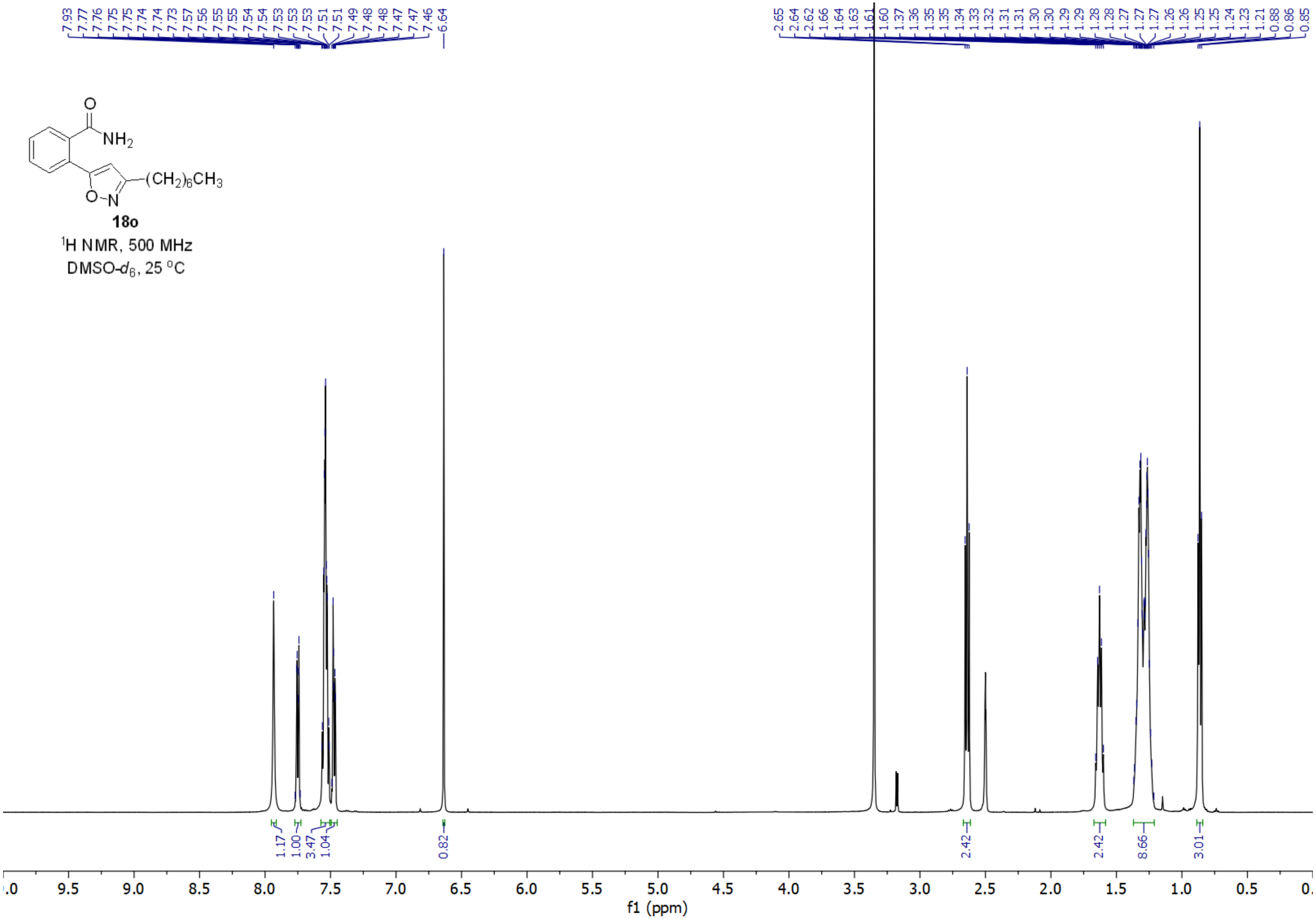
18o
^{1}H NMR, 500 MHz
DMSO-d6, 25 °C
f1 (ppm)

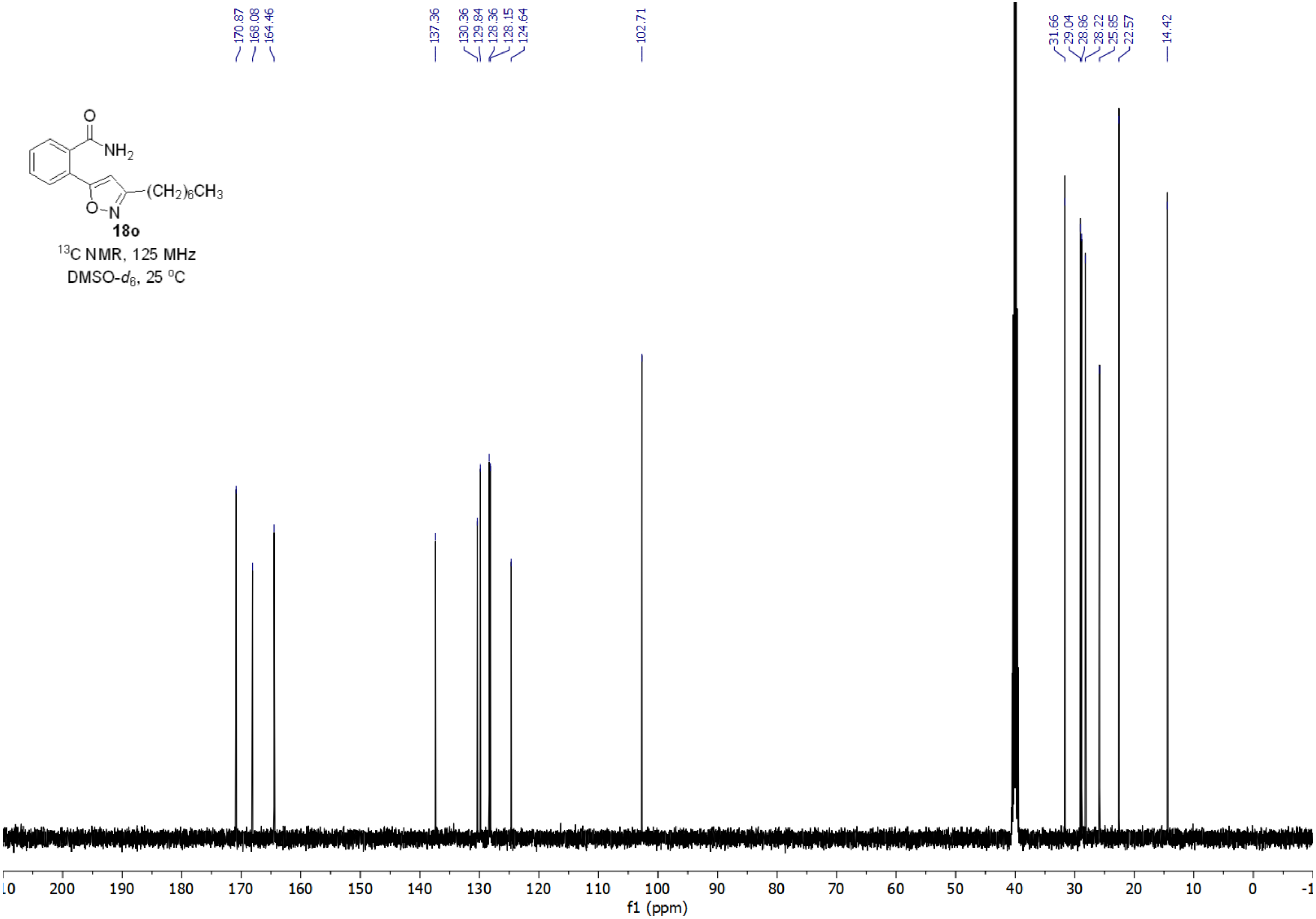

18o

^{13}C NMR, 125 MHz

DMSO-d_6, 25 °C

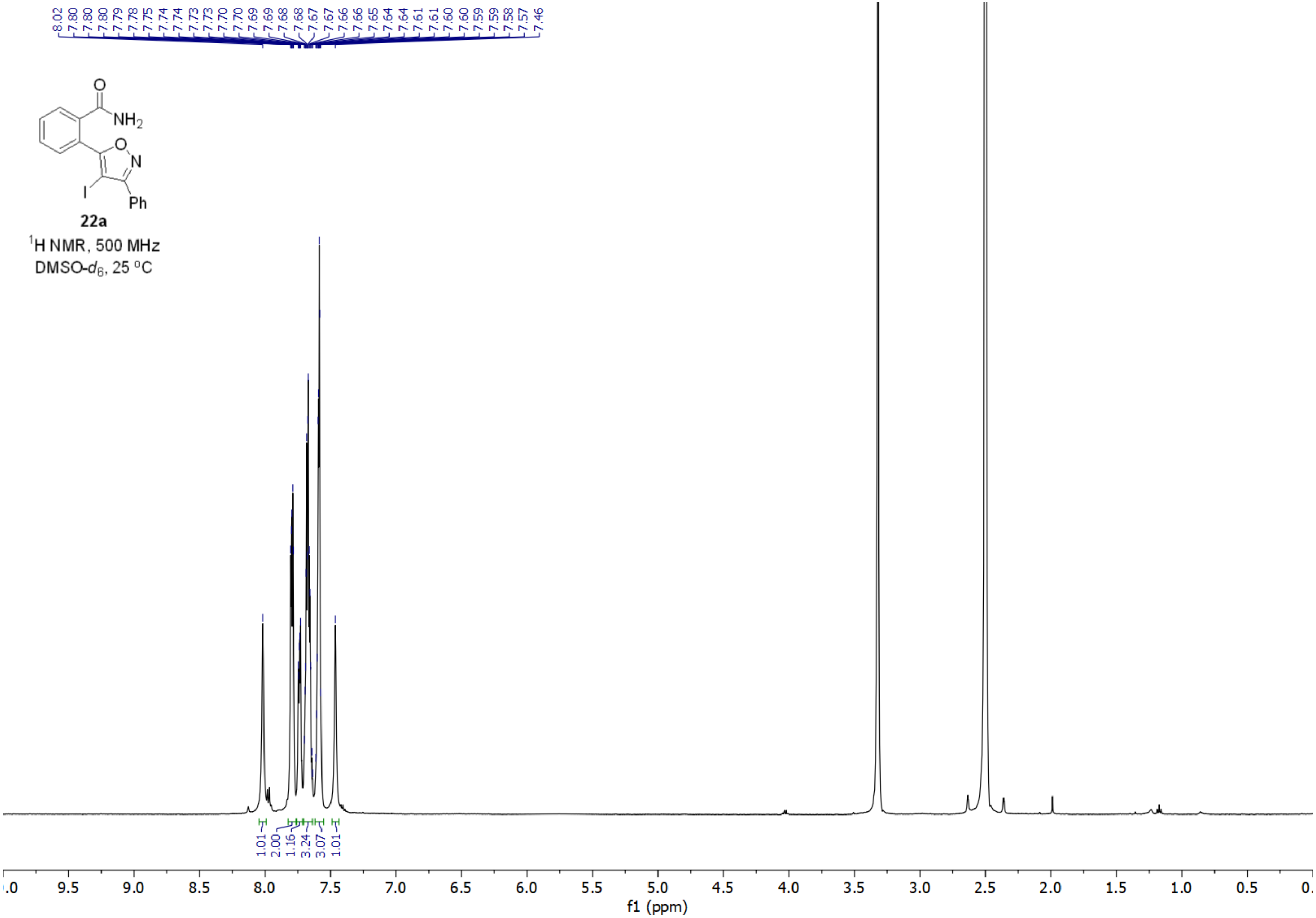
8.02
7.80
7.80
7.79
7.78
7.75
7.74
7.74
7.73
7.73
7.70
7.69
7.69
7.68
7.68
7.67
7.67
7.66
7.66
7.65
7.64
7.64
7.61
7.61
7.60
7.60
7.59
7.59
7.58
7.57
7.46
O
NH2
O
N
I
Ph
22a
1H NMR, 500 MHz
DMSO-d6, 25 °C
1.01
2.00
1.16
3.24
3.07
1.01
9.5
9.0
8.5
8.0
7.5
7.0
6.5
6.0
5.5
5.0
4.5
4.0
3.5
3.0
2.5
2.0
1.5
1.0
0.5
f1 (ppm)

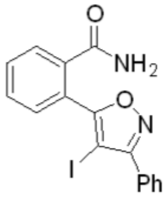

22a

^{13}C NMR, 125 MHz

DMSO-d_6, 25 °C

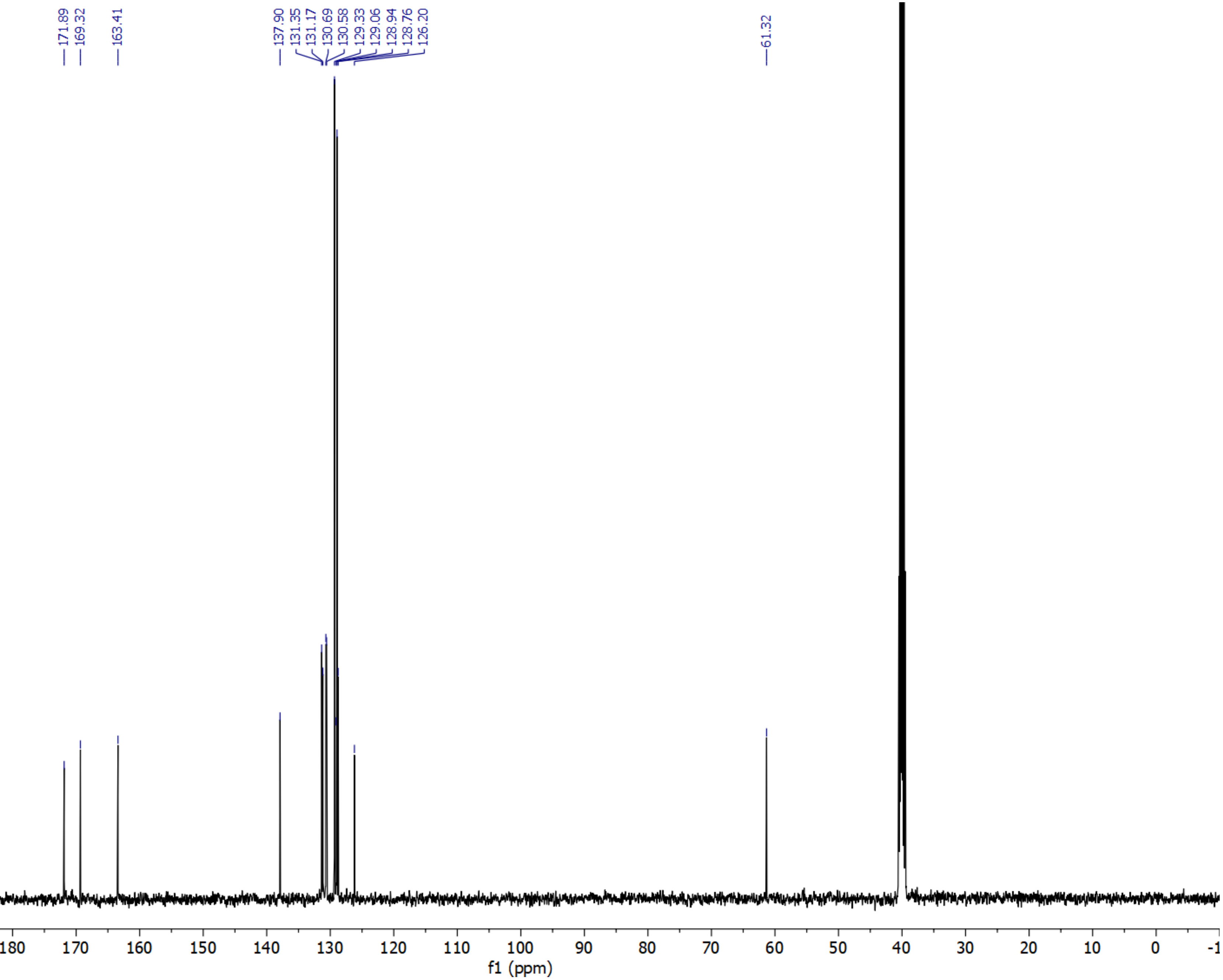

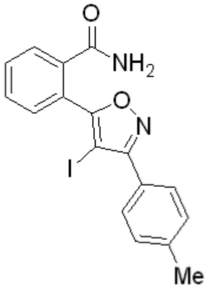

22b

^{1}H NMR, 500 MHz

$CDCl_3$, 25 °C

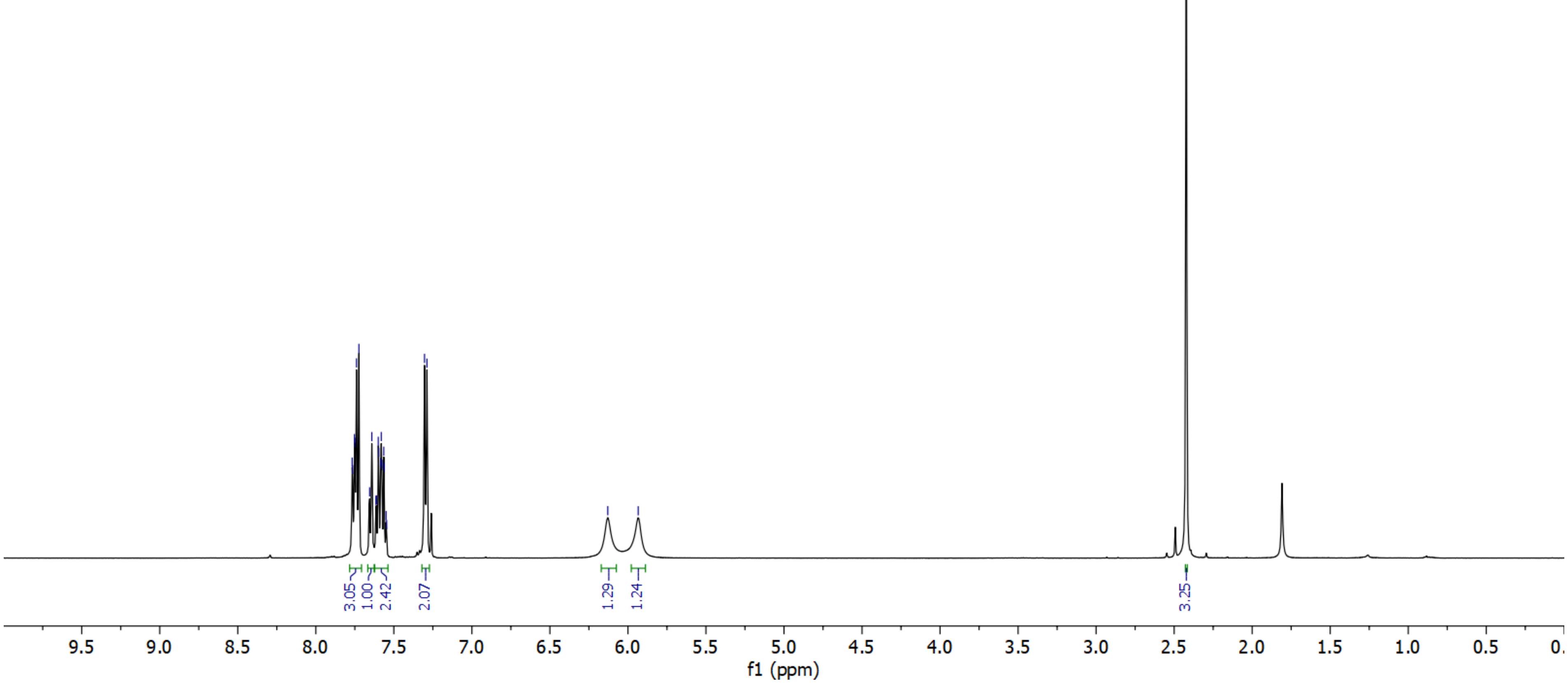

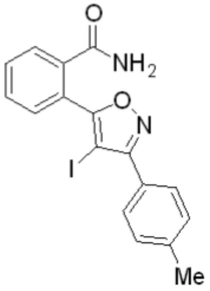

22b

^{13}C NMR, 125 MHz

$CDCl_3$, 25 °C

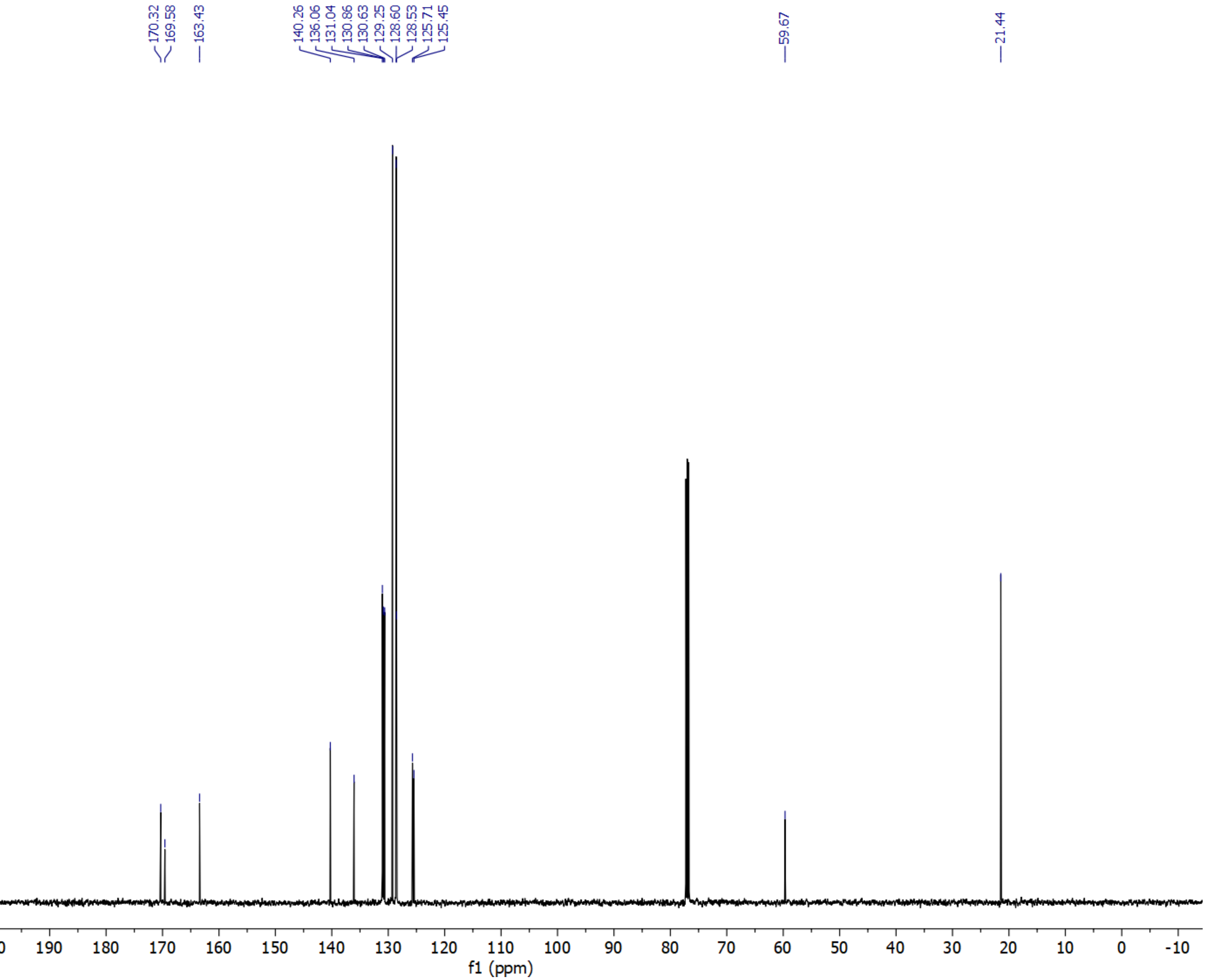

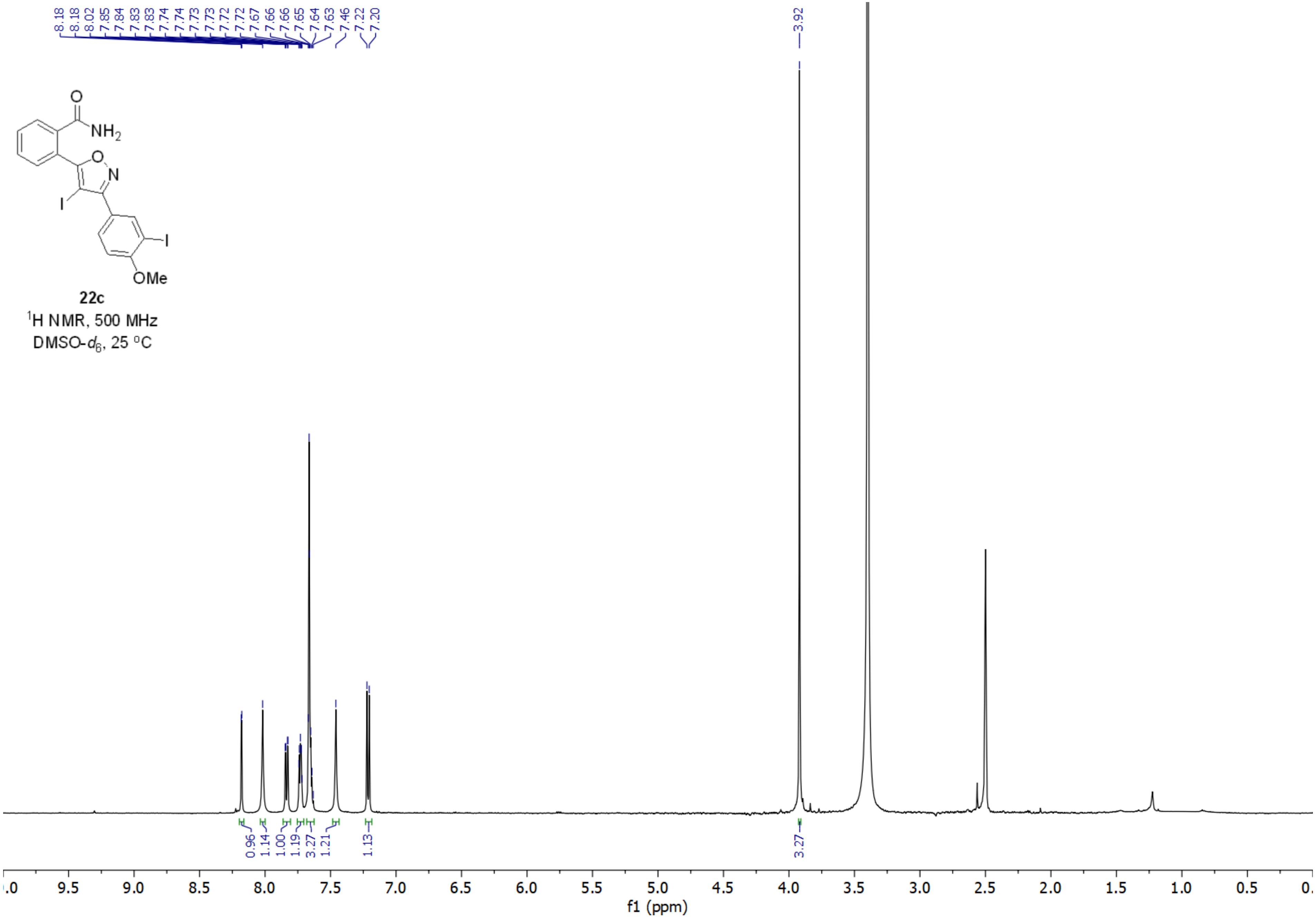
8.18 8.18 8.02 7.85 7.84 7.83 7.83 7.74 7.74 7.73 7.73 7.72 7.72 7.67 7.66 7.66 7.65 7.64 7.63 7.46 7.22 7.20
3.92
O
NH2
O
N
I
I
OMe
22c
1H NMR, 500 MHz
DMSO-d6, 25 °C
0.96 1.14 1.00 1.19 3.27 1.21 1.13
3.27
.0 9.5 9.0 8.5 8.0 7.5 7.0 6.5 6.0 5.5 5.0 4.5 4.0 3.5 3.0 2.5 2.0 1.5 1.0 0.5 0.
f1 (ppm)

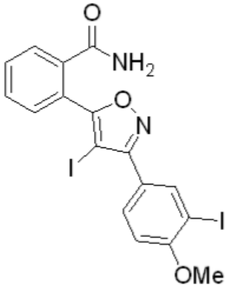

22c

^{13}C NMR, 125 MHz

DMSO-d_6, 25 °C

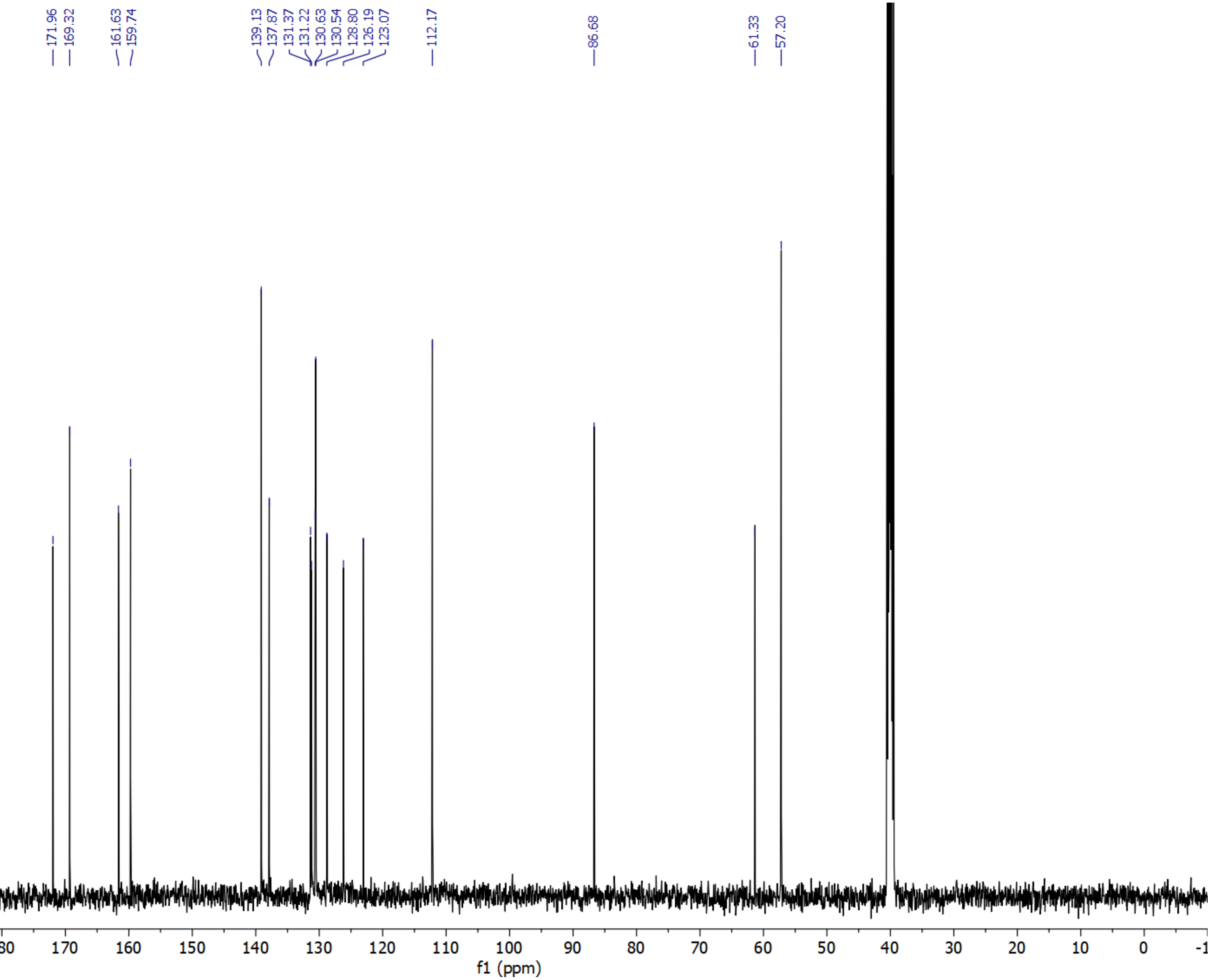

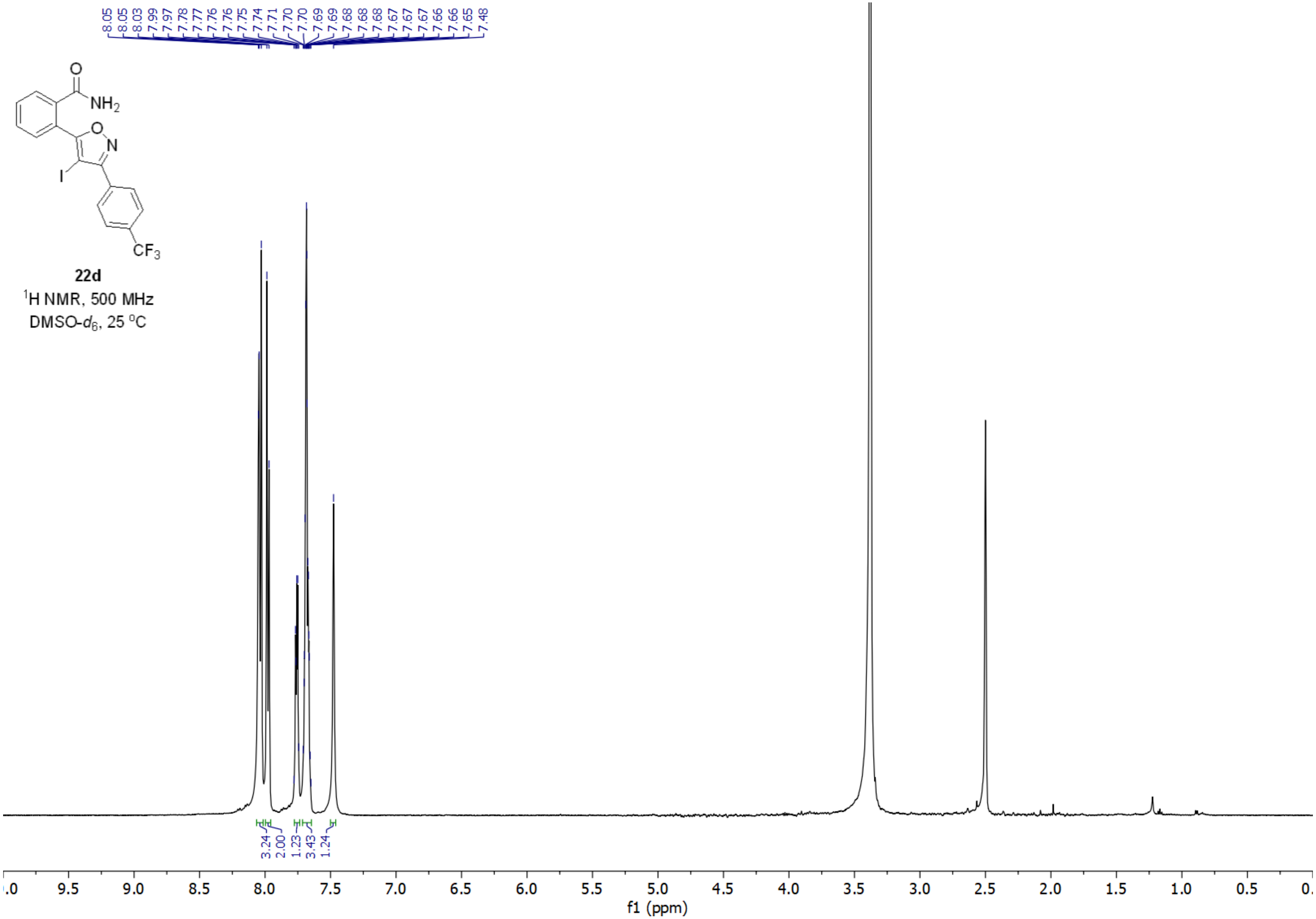

22d
^{1}H NMR, 500 MHz
DMSO-d_6, 25 °C

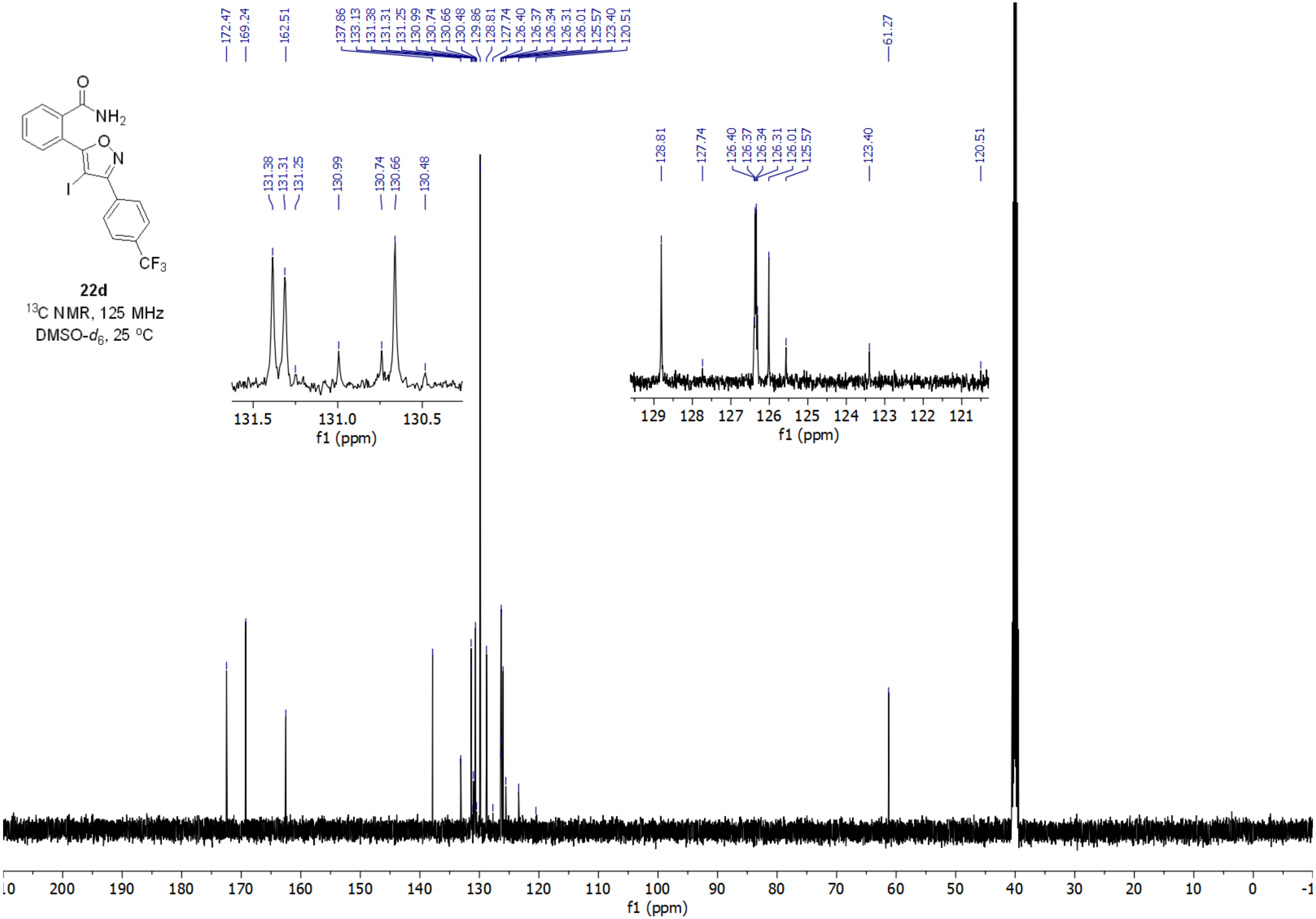

22d

^{13}C NMR, 125 MHz

DMSO-d_6, 25 °C

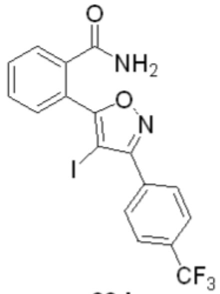

22d

^{19}F NMR, 470 MHz

DMSO-d_6, 25 °C

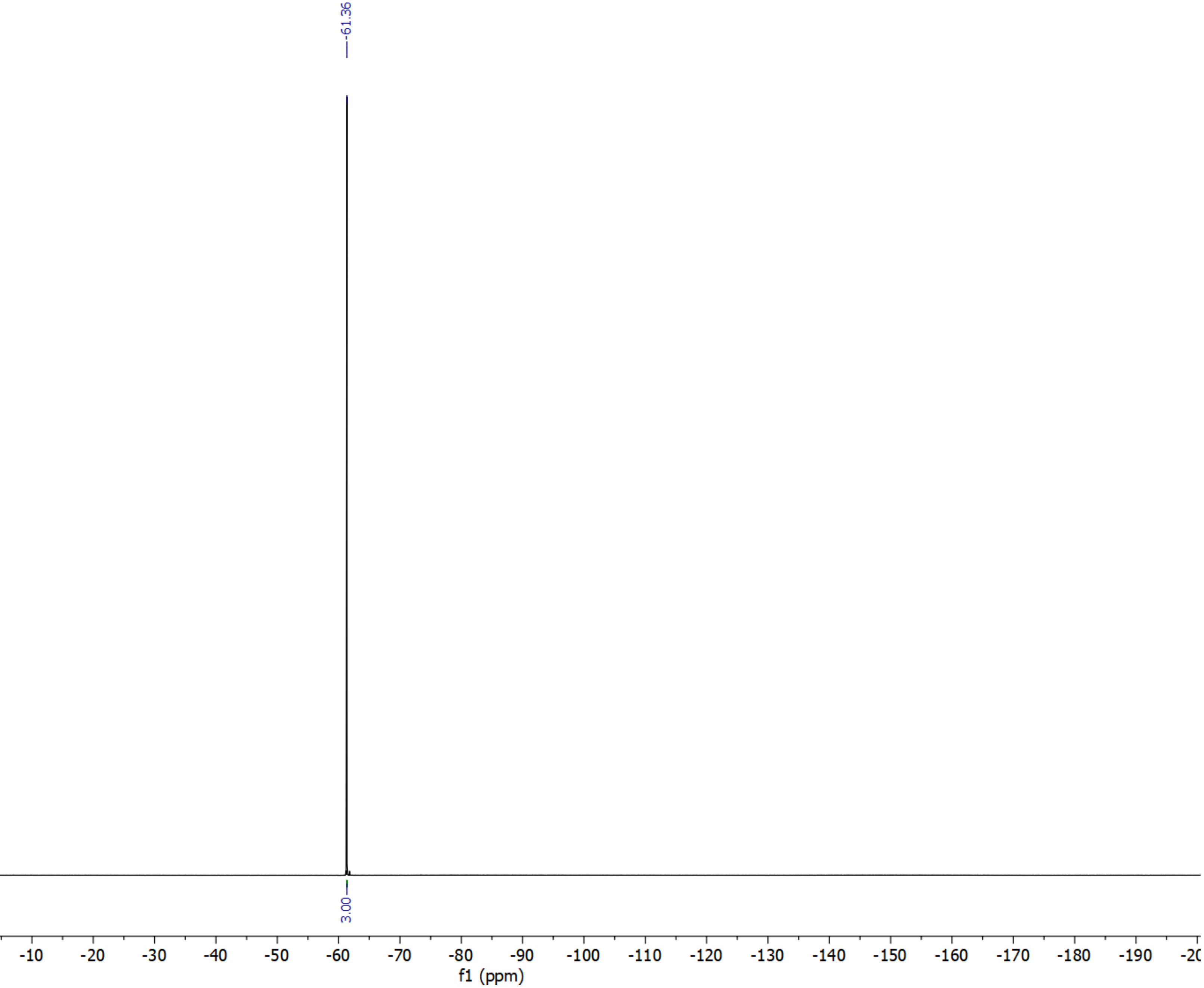

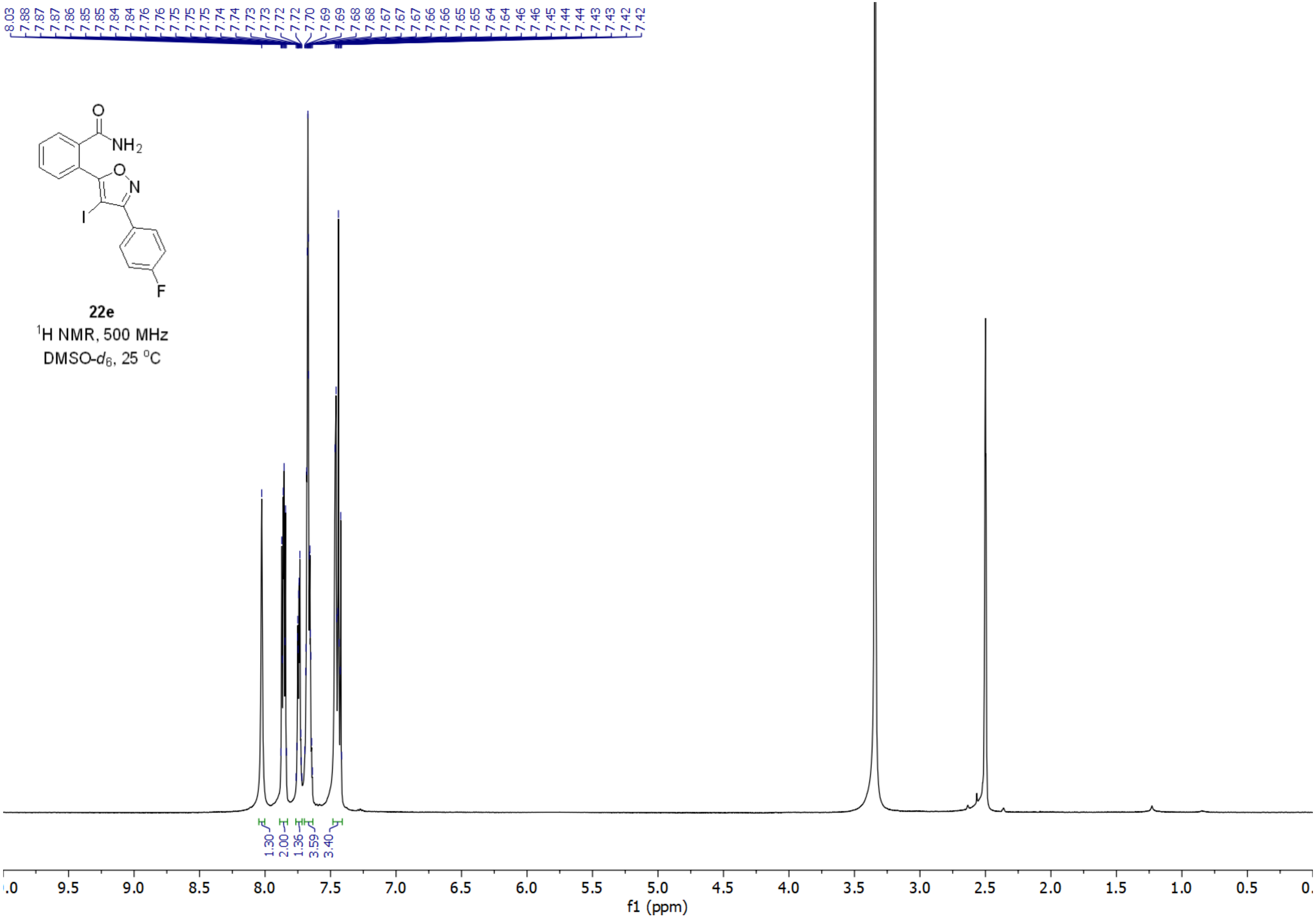
8.03 7.88 7.87 7.87 7.86 7.85 7.85 7.84 7.84 7.76 7.76 7.75 7.75 7.75 7.74 7.74 7.73 7.73 7.72 7.72 7.70 7.69 7.69 7.68 7.67 7.67 7.67 7.66 7.66 7.65 7.65 7.64 7.64 7.46 7.46 7.45 7.44 7.44 7.43 7.43 7.42 7.42
O
NH2
O
N
I
F
22e
1H NMR, 500 MHz
DMSO-d6, 25 °C
1.30 2.00 1.36 3.59 3.40
.0 9.5 9.0 8.5 8.0 7.5 7.0 6.5 6.0 5.5 5.0 4.5 4.0 3.5 3.0 2.5 2.0 1.5 1.0 0.5 0.
f1 (ppm)

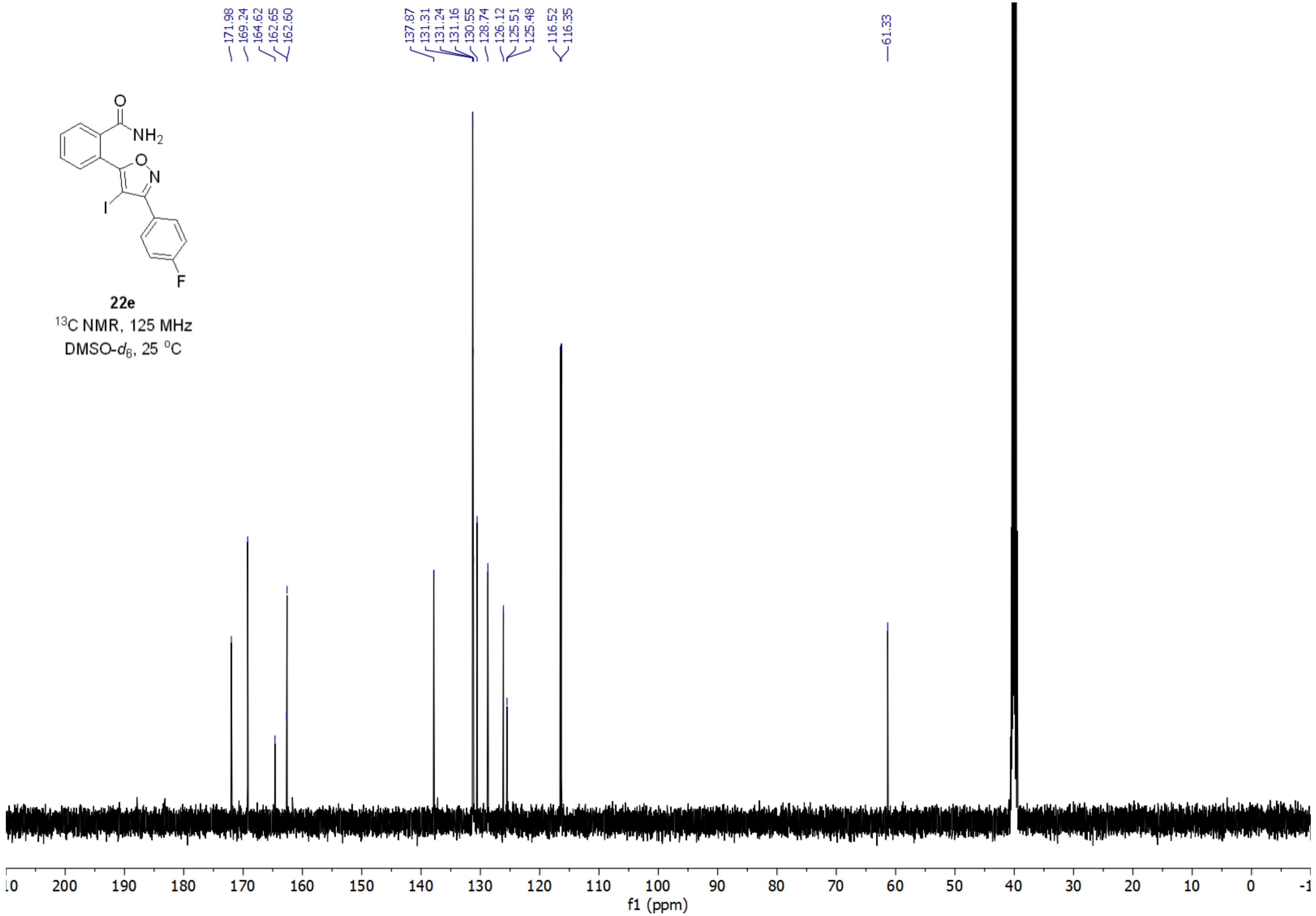

171.98
169.24
164.62
162.65
162.60
137.87
131.31
131.24
131.16
130.55
128.74
126.12
125.51
125.48
116.52
116.35
61.33
O
NH2
O
N
I
F
22e
13C NMR, 125 MHz
DMSO-d6, 25 °C
f1 (ppm)

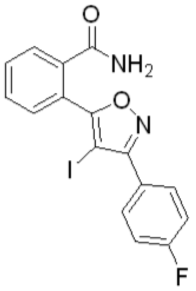

22e
^{19}F NMR, 470 MHz
DMSO-d_6, 25 °C

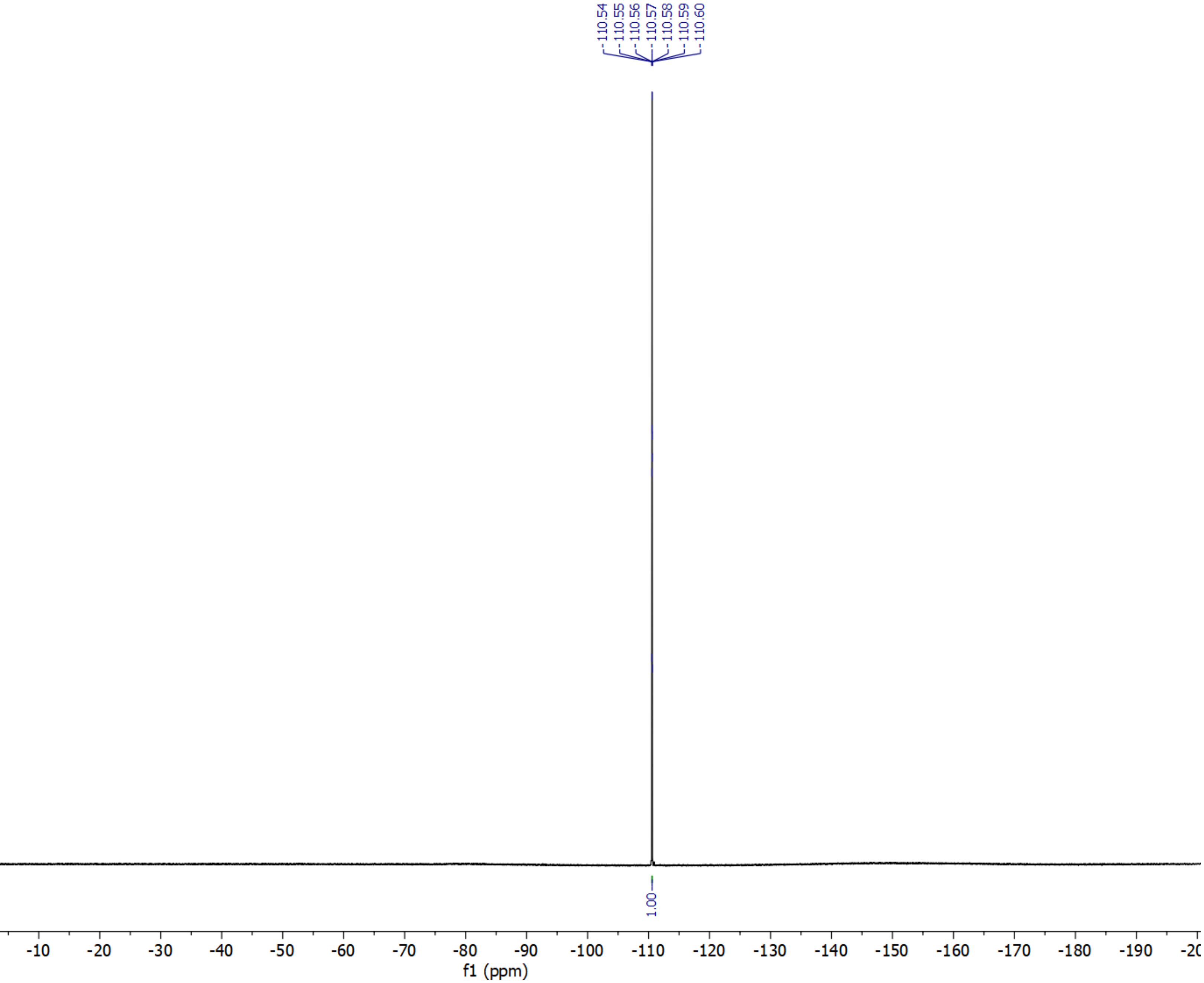

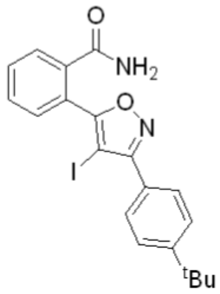

22f

^{1}H NMR, 500 MHz

DMSO-d_6, 25 ^{o}C

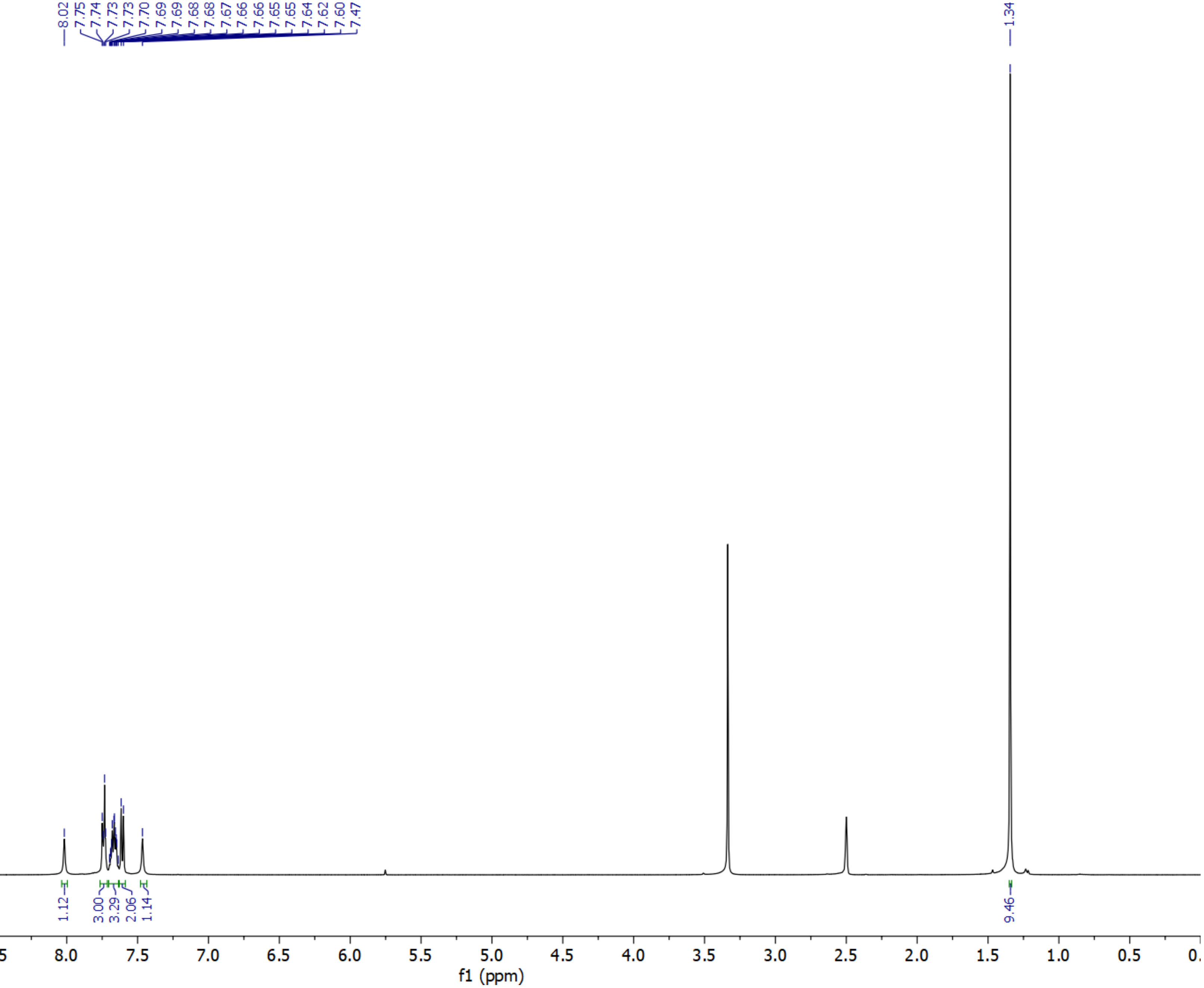

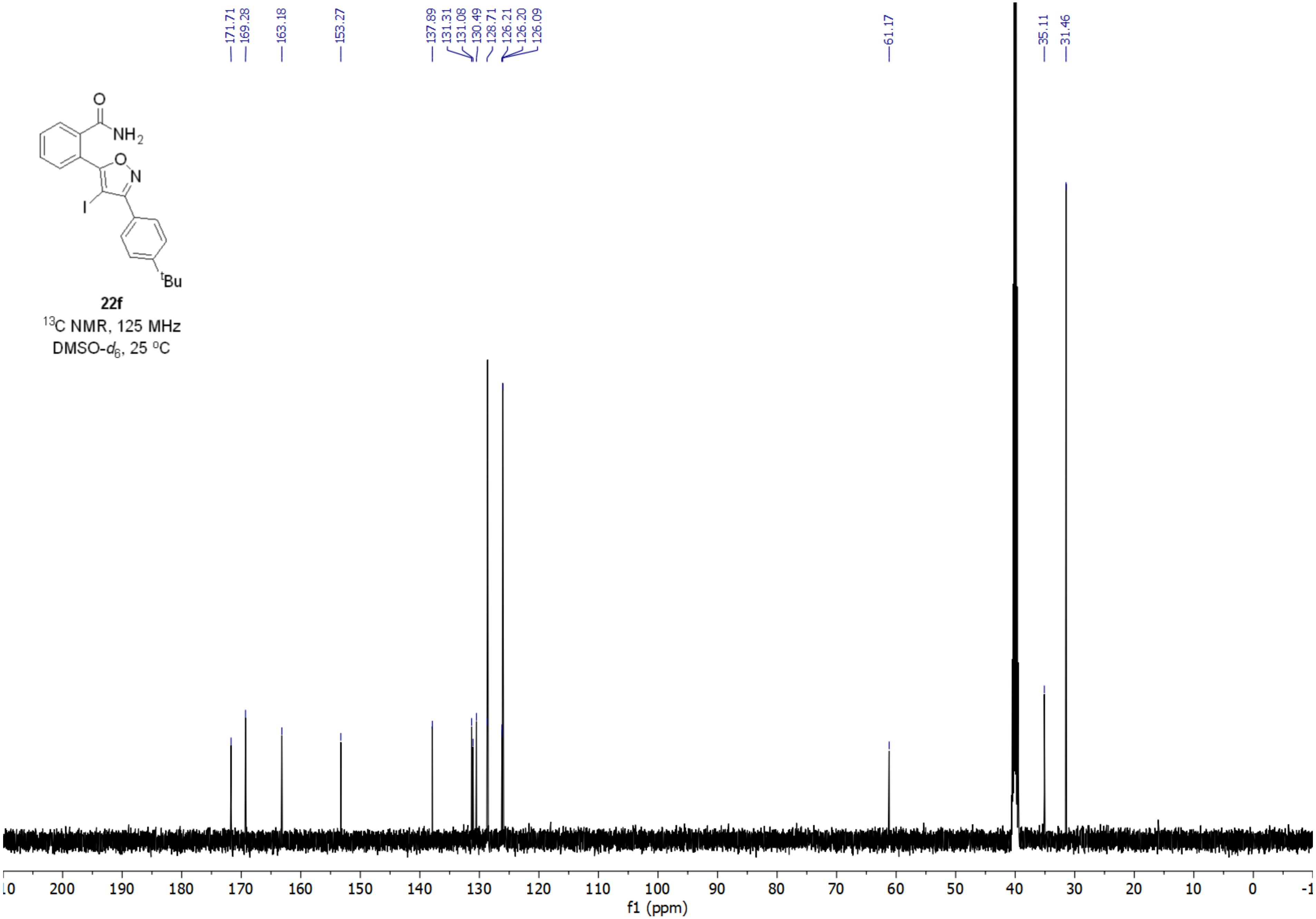

22f
^{13}C NMR, 125 MHz
DMSO-d_6, 25 °C

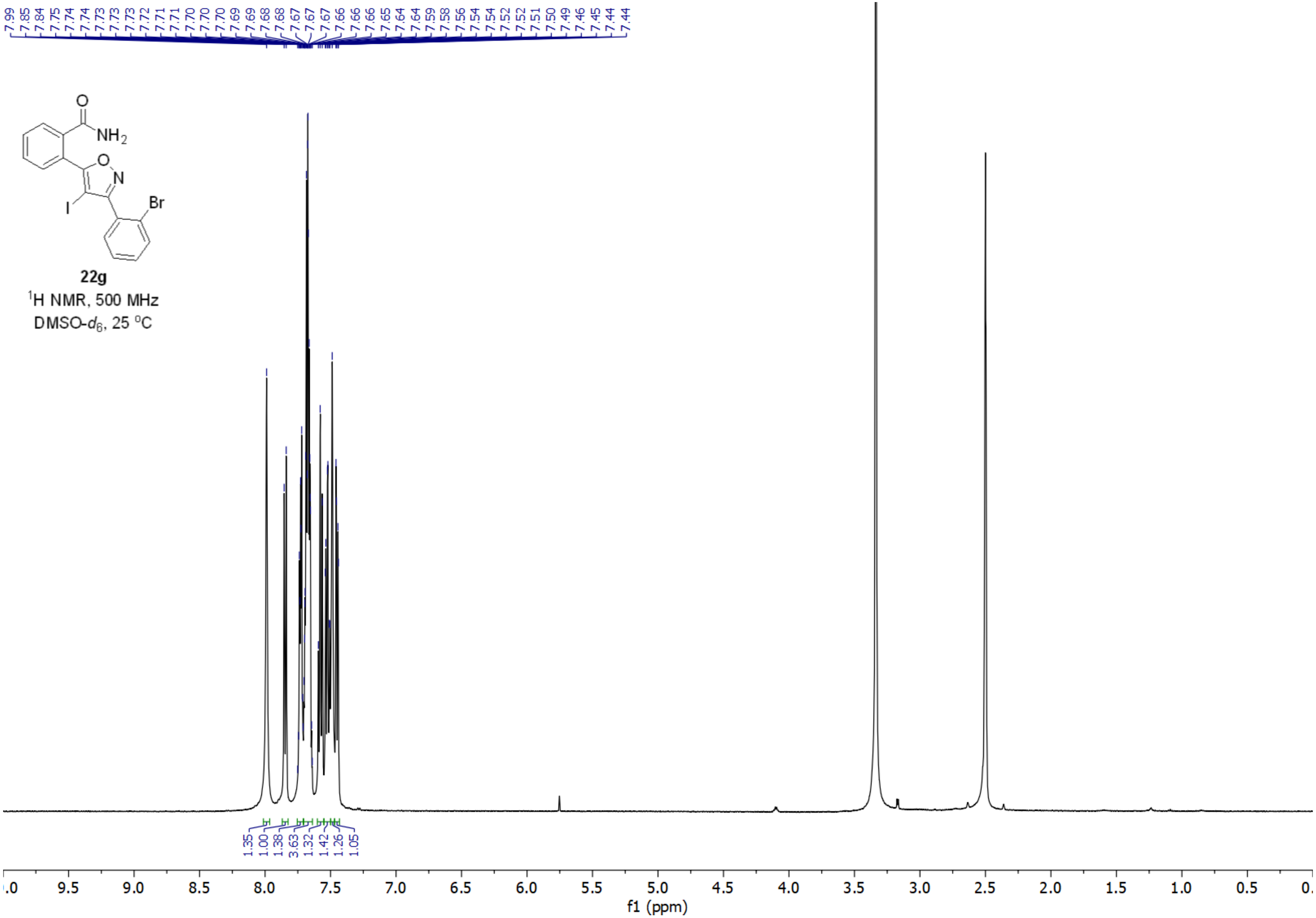
7.99
7.85
7.84
7.75
7.74
7.73
7.73
7.73
7.72
7.71
7.71
7.70
7.70
7.70
7.69
7.69
7.68
7.68
7.67
7.67
7.67
7.66
7.66
7.66
7.65
7.64
7.64
7.59
7.58
7.56
7.54
7.54
7.52
7.52
7.51
7.50
7.49
7.46
7.45
7.44
7.44
O
NH2
O
N
Br
I
22g
1H NMR, 500 MHz
DMSO-d6, 25 °C
1.35
1.00
1.38
3.63
1.32
1.42
1.26
1.05
9.5
9.0
8.5
8.0
7.5
7.0
6.5
6.0
5.5
5.0
4.5
4.0
3.5
3.0
2.5
2.0
1.5
1.0
0.5
f1 (ppm)

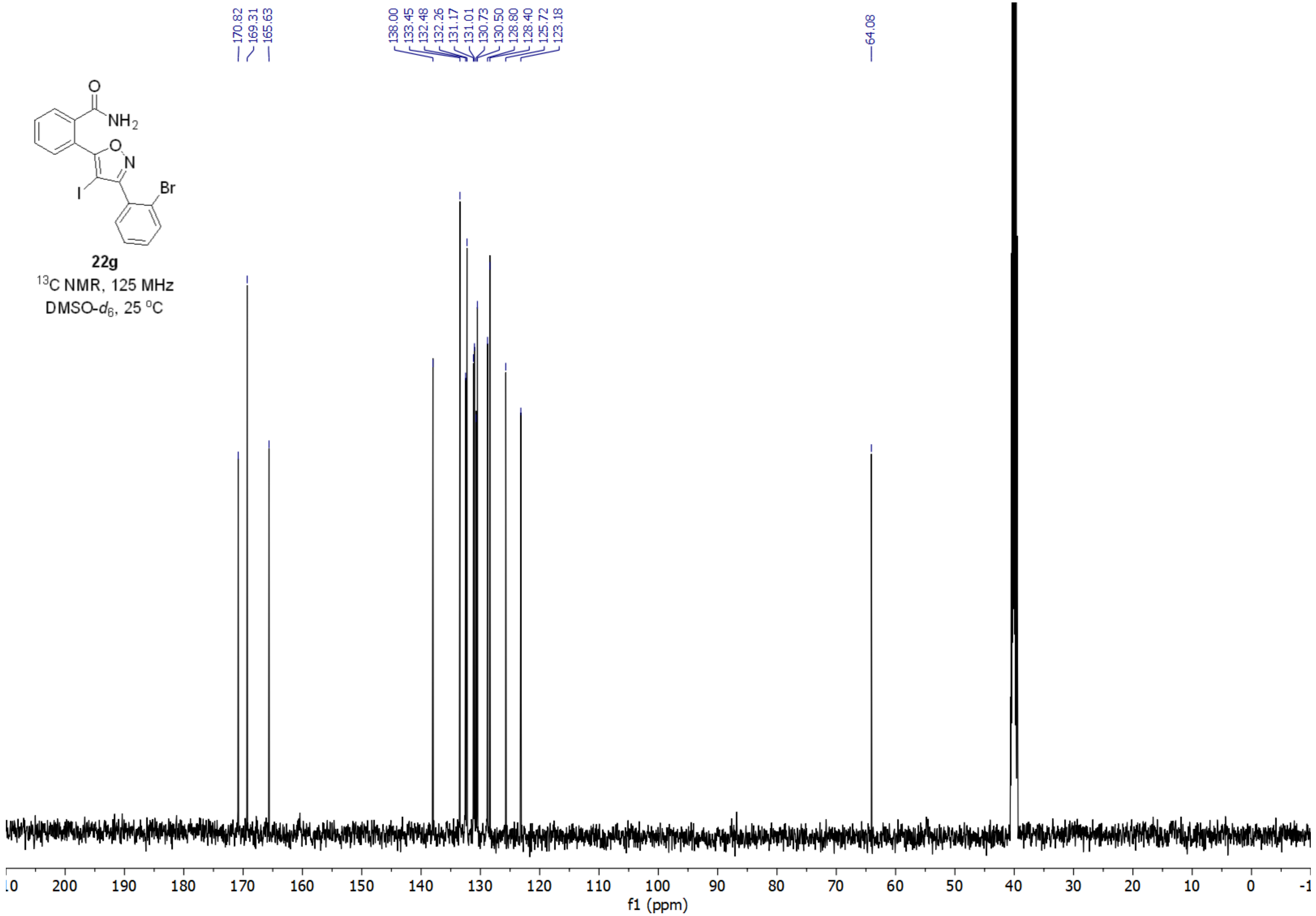

170.82
169.31
165.63
138.00
133.45
132.48
132.26
131.17
131.01
130.73
130.50
128.80
128.40
125.72
123.18
64.08
O
NH2
O
N
I
Br
22g
13C NMR, 125 MHz
DMSO-d6, 25 °C
200
190
180
170
160
150
140
130
120
110
100
90
80
70
60
50
40
30
20
10
0
f1 (ppm)

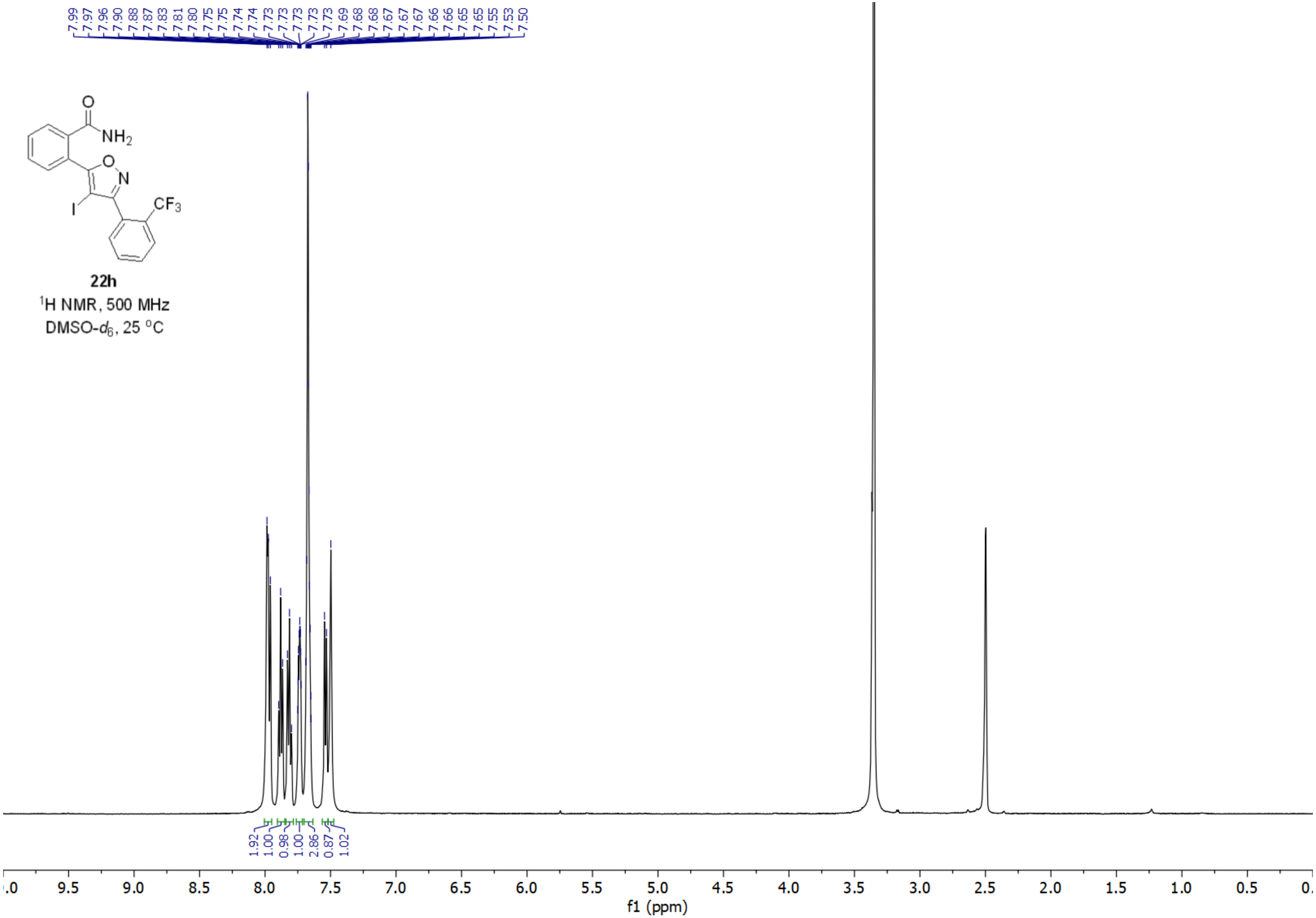
7.99 7.97 7.96 7.90 7.88 7.87 7.83 7.81 7.80 7.75 7.75 7.74 7.74 7.73 7.73 7.73 7.73 7.73 7.69 7.68 7.68 7.67 7.67 7.67 7.66 7.66 7.65 7.65 7.55 7.53 7.50
O
NH2
O
N
I
CF3
22h
1H NMR, 500 MHz
DMSO-d6, 25 °C
1.92 1.00 0.98 1.00 2.86 0.87 1.02
f1 (ppm)

22h

^{13}C NMR, 125 MHz

DMSO-d_6, 25 °C

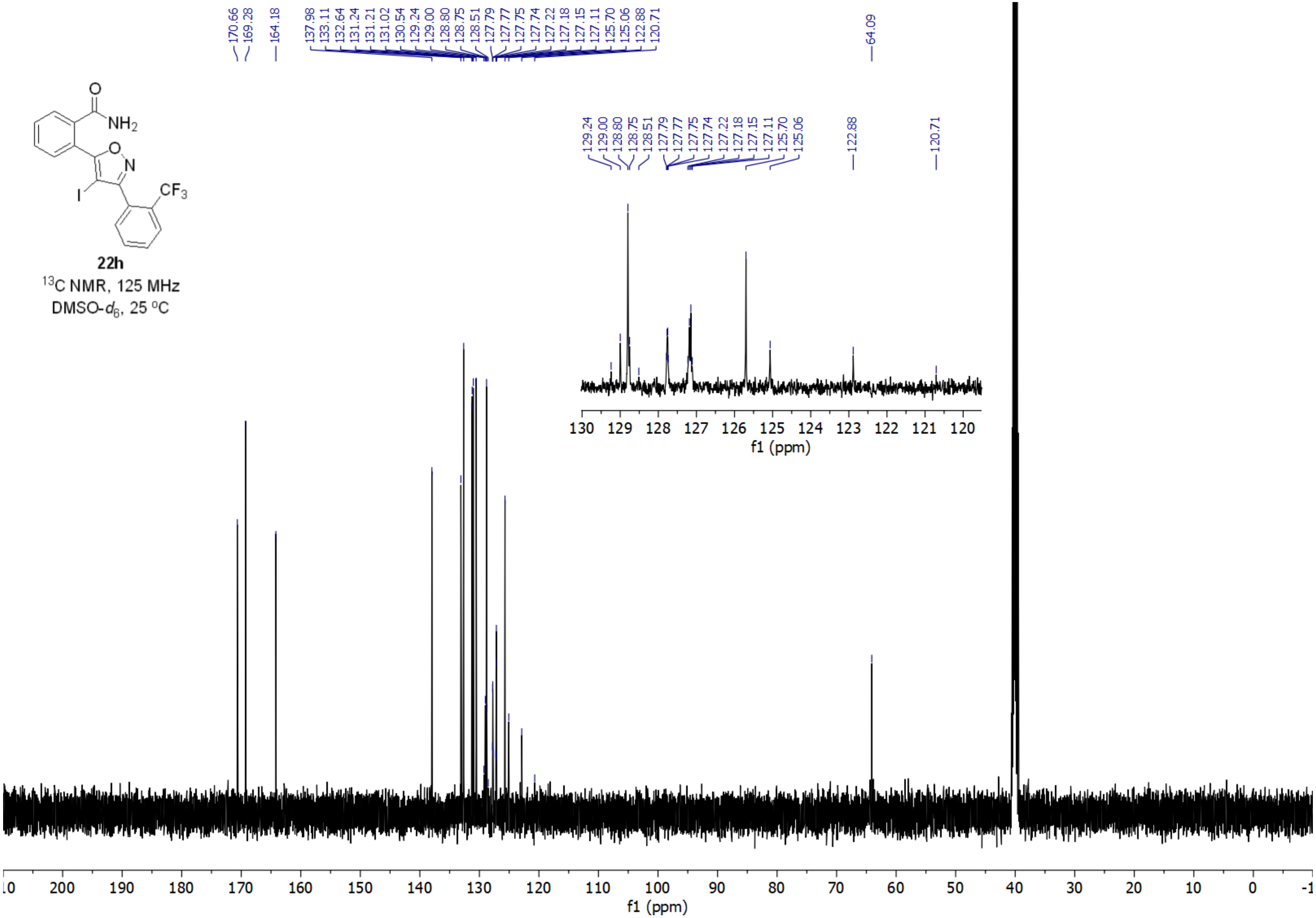

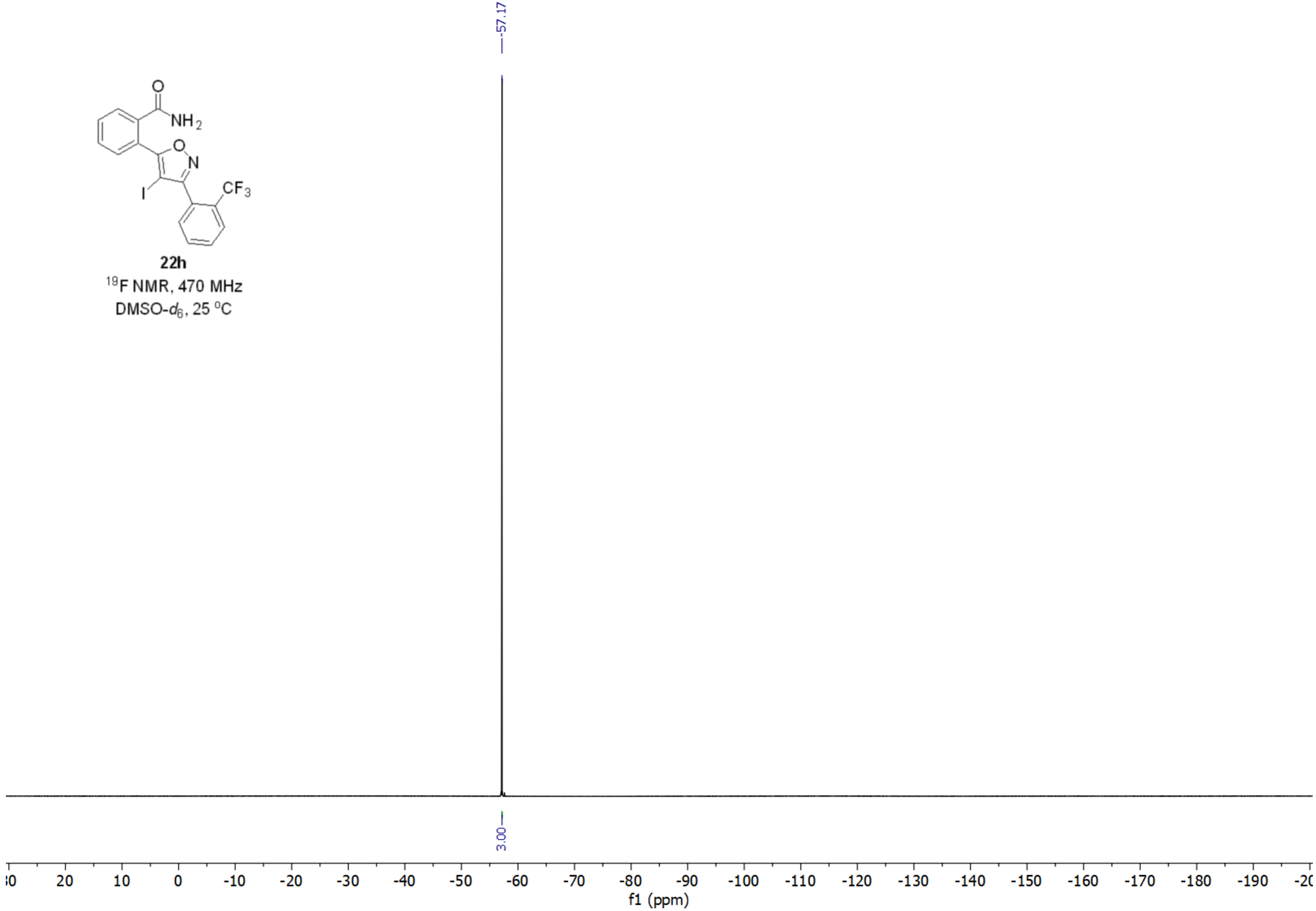
-57.17
22h
19F NMR, 470 MHz
DMSO-d6, 25 °C
3.00
f1 (ppm)

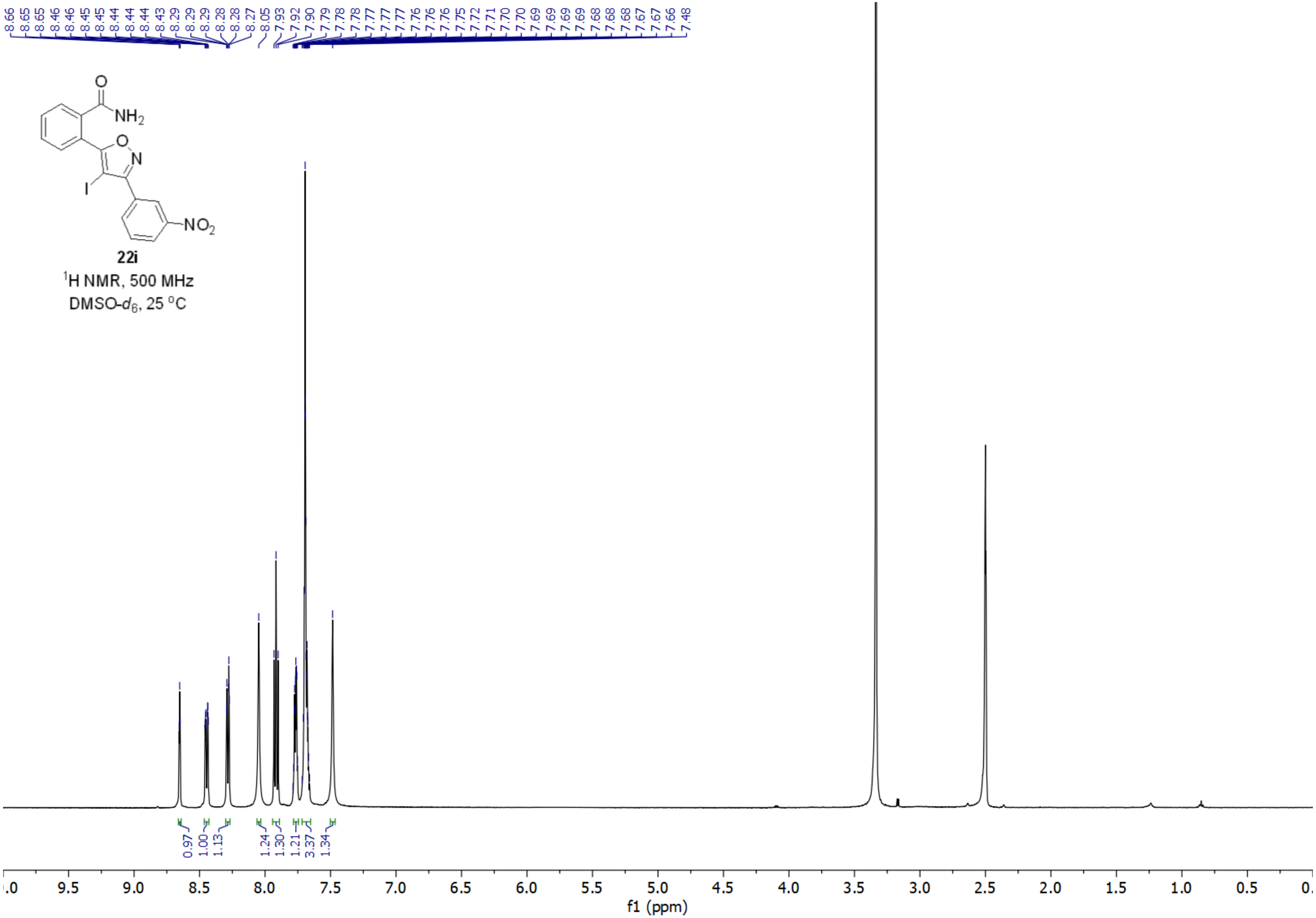
8.66 8.65 8.65 8.46 8.45 8.45 8.44 8.44 8.44 8.43 8.29 8.29 8.29 8.28 8.28 8.27 8.05 7.93 7.92 7.90 7.79 7.78 7.78 7.77 7.77 7.77 7.76 7.76 7.75 7.72 7.71 7.70 7.69 7.69 7.69 7.68 7.68 7.68 7.67 7.67 7.66 7.48
O
NH2
O
N
I
NO2
22i
1H NMR, 500 MHz
DMSO-d6, 25 °C
0.97 1.00 1.13 1.24 1.30 1.21 3.37 1.34
.0 9.5 9.0 8.5 8.0 7.5 7.0 6.5 6.0 5.5 5.0 4.5 4.0 3.5 3.0 2.5 2.0 1.5 1.0 0.5 0.
f1 (ppm)

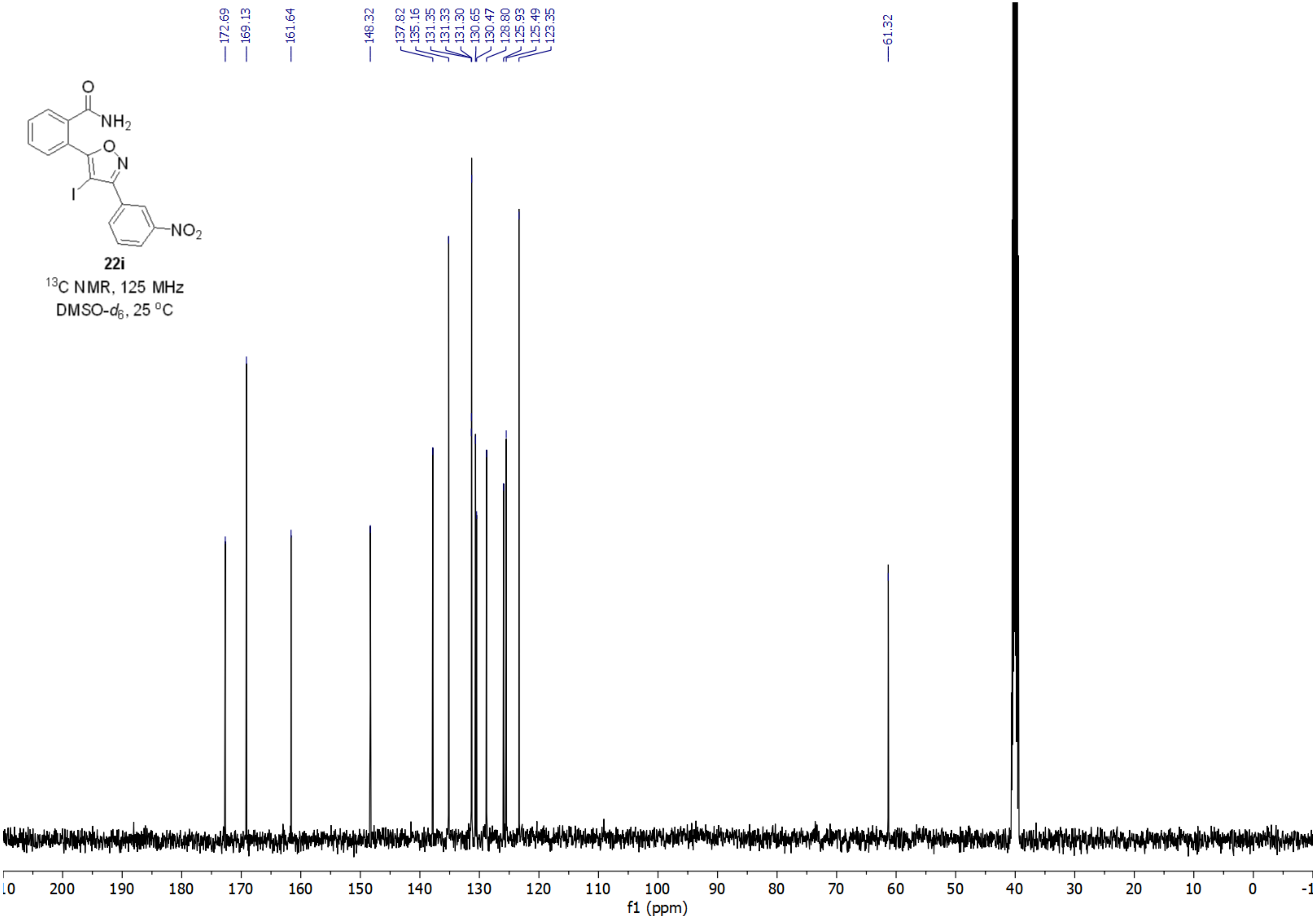
172.69
169.13
161.64
148.32
137.82
135.16
131.35
131.33
131.30
130.65
130.47
128.80
125.93
125.49
123.35
61.32
O
NH2
O
N
I
NO2
22i
13C NMR, 125 MHz
DMSO-d6, 25 °C
10 200 190 180 170 160 150 140 130 120 110 100 90 80 70 60 50 40 30 20 10 0 -1
f1 (ppm)

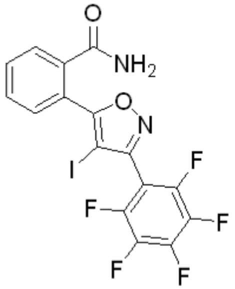

22k

^{1}H NMR, 500 MHz

DMSO-d_6, 25 °C

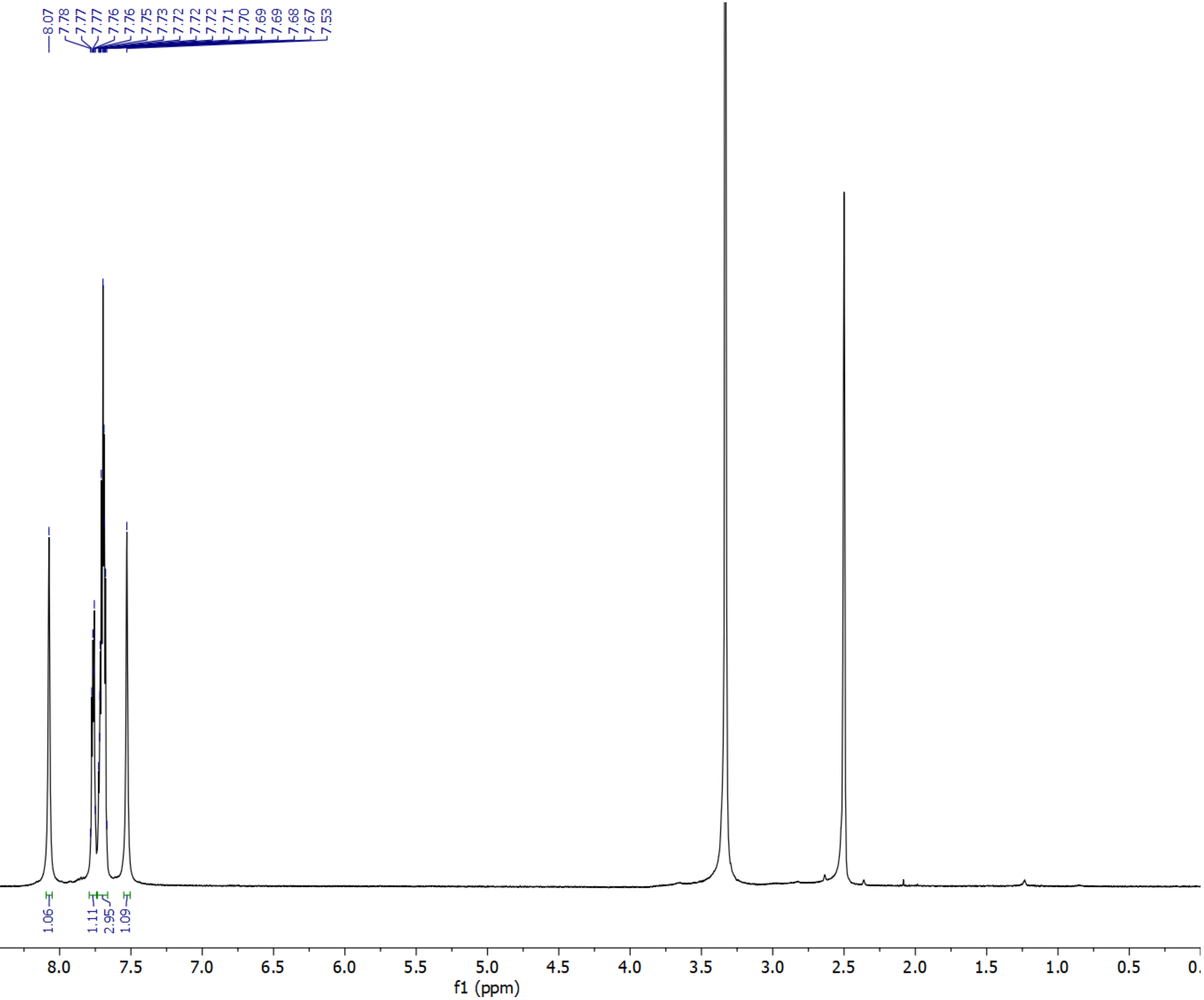

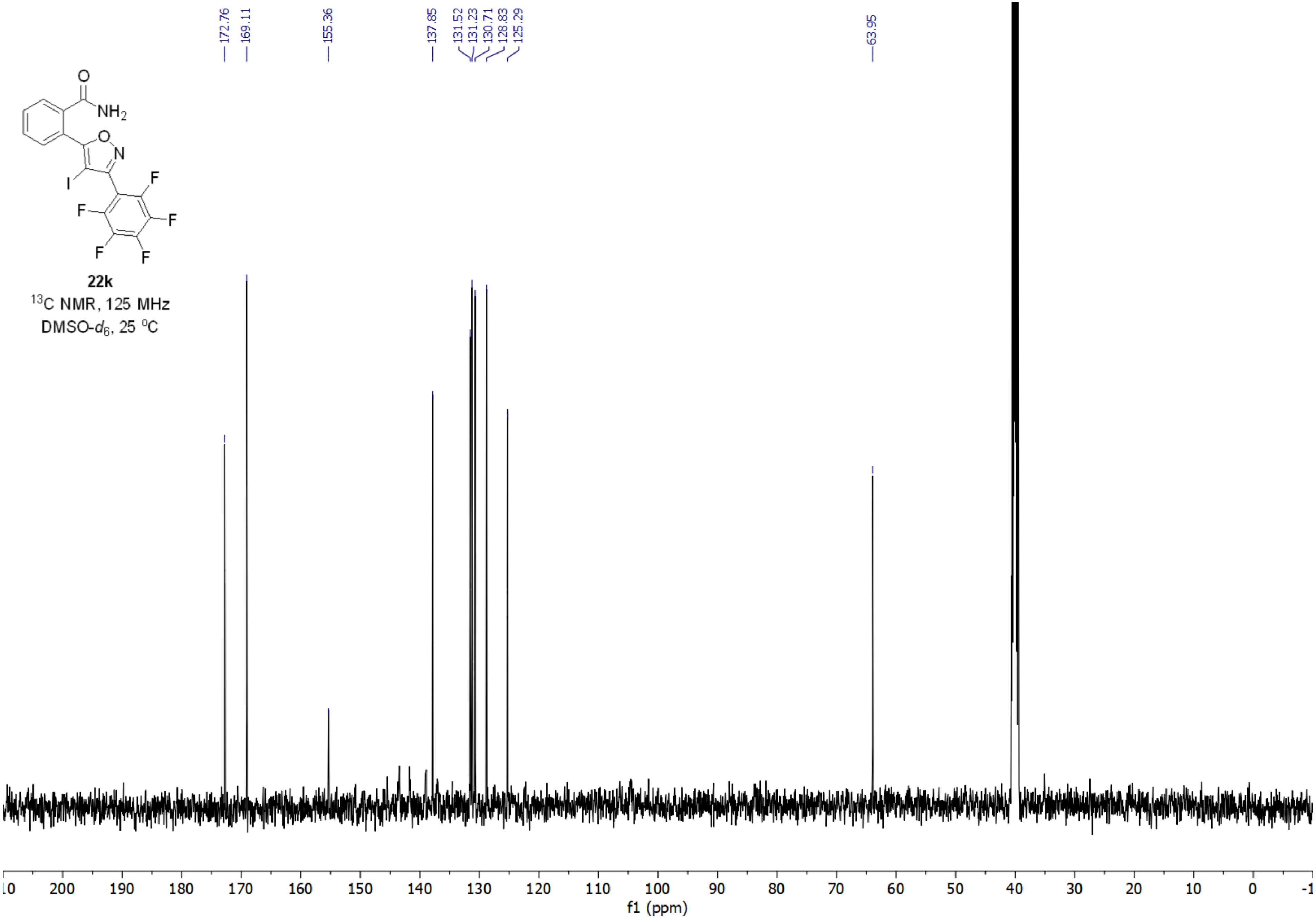
172.76
169.11
155.36
137.85
131.52
131.23
130.71
128.83
125.29
63.95
O
NH2
O
N
I
F
F
F
F
F
22k
13C NMR, 125 MHz
DMSO-d6, 25 °C
210
200
190
180
170
160
150
140
130
120
110
100
90
80
70
60
50
40
30
20
10
0
-10
f1 (ppm)

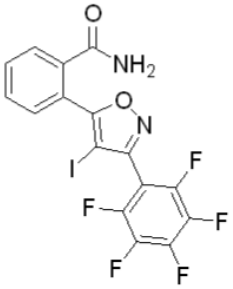

22k

^{19}F NMR, 470 MHz

DMSO-d_6, 25 ^{o}C

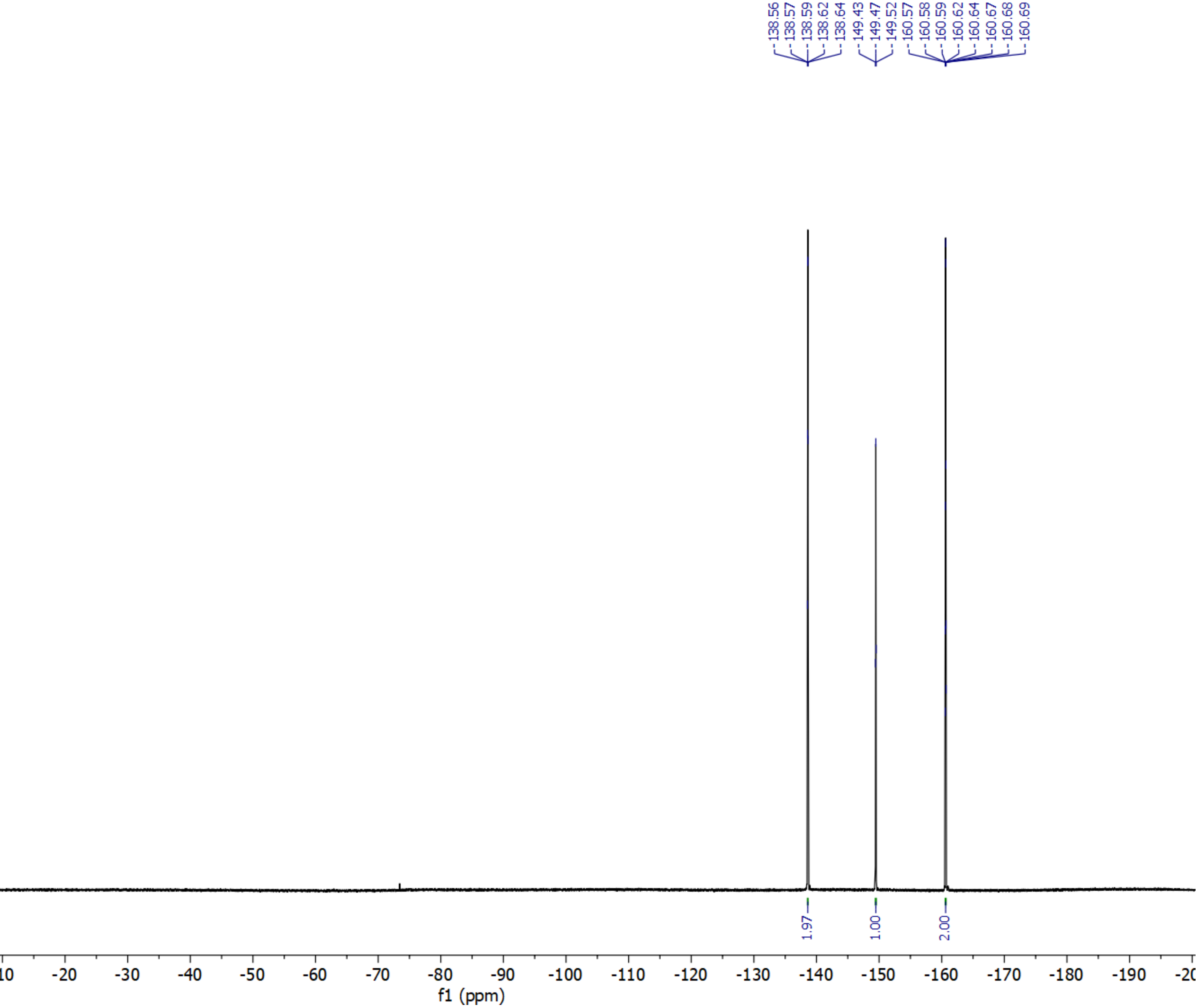

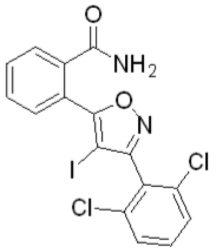

22l

^1H NMR, 500 MHz

DMSO-d_6, 25 °C

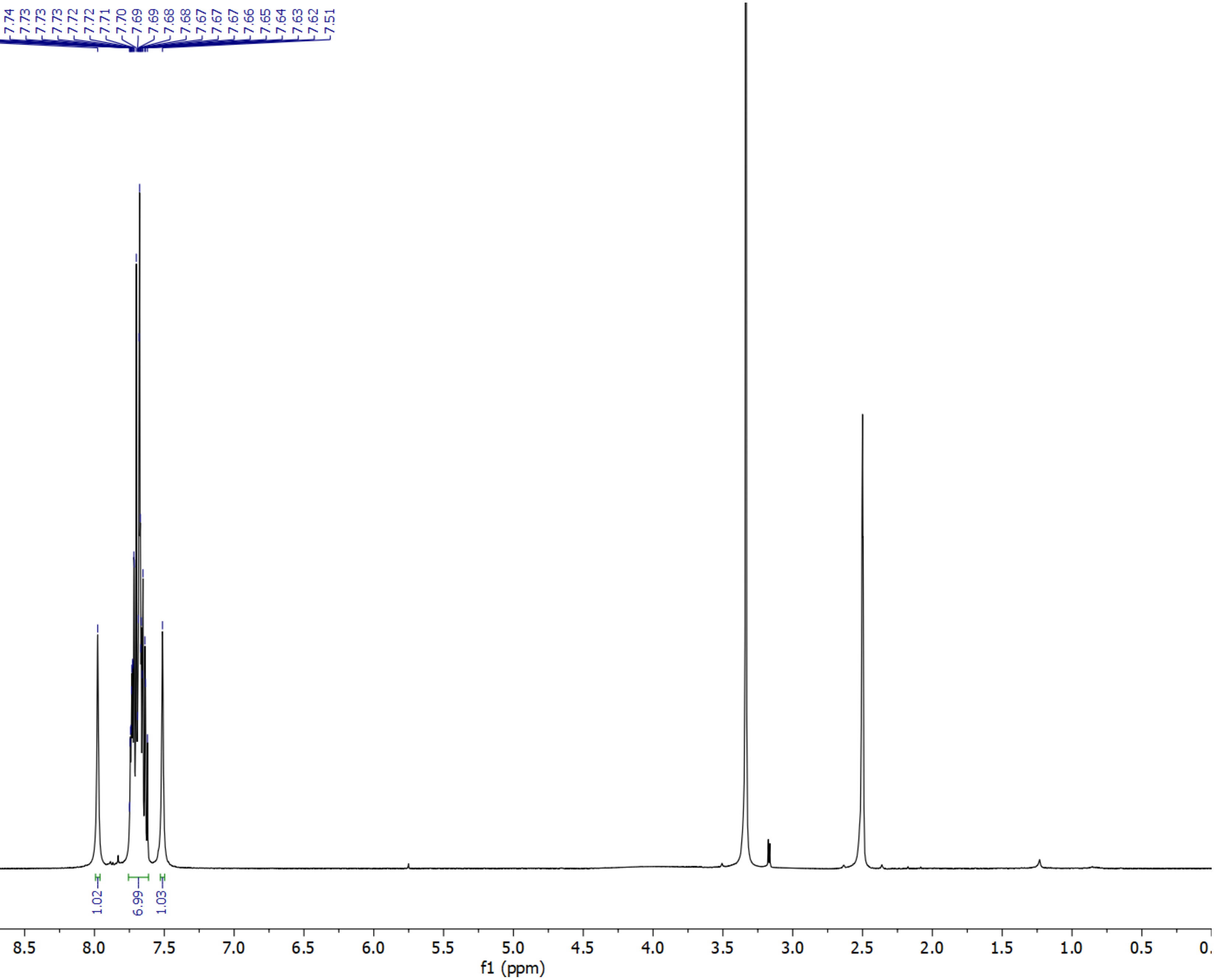

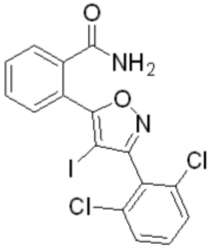

22l

^{13}C NMR, 125 MHz

DMSO-d_6, 25 °C

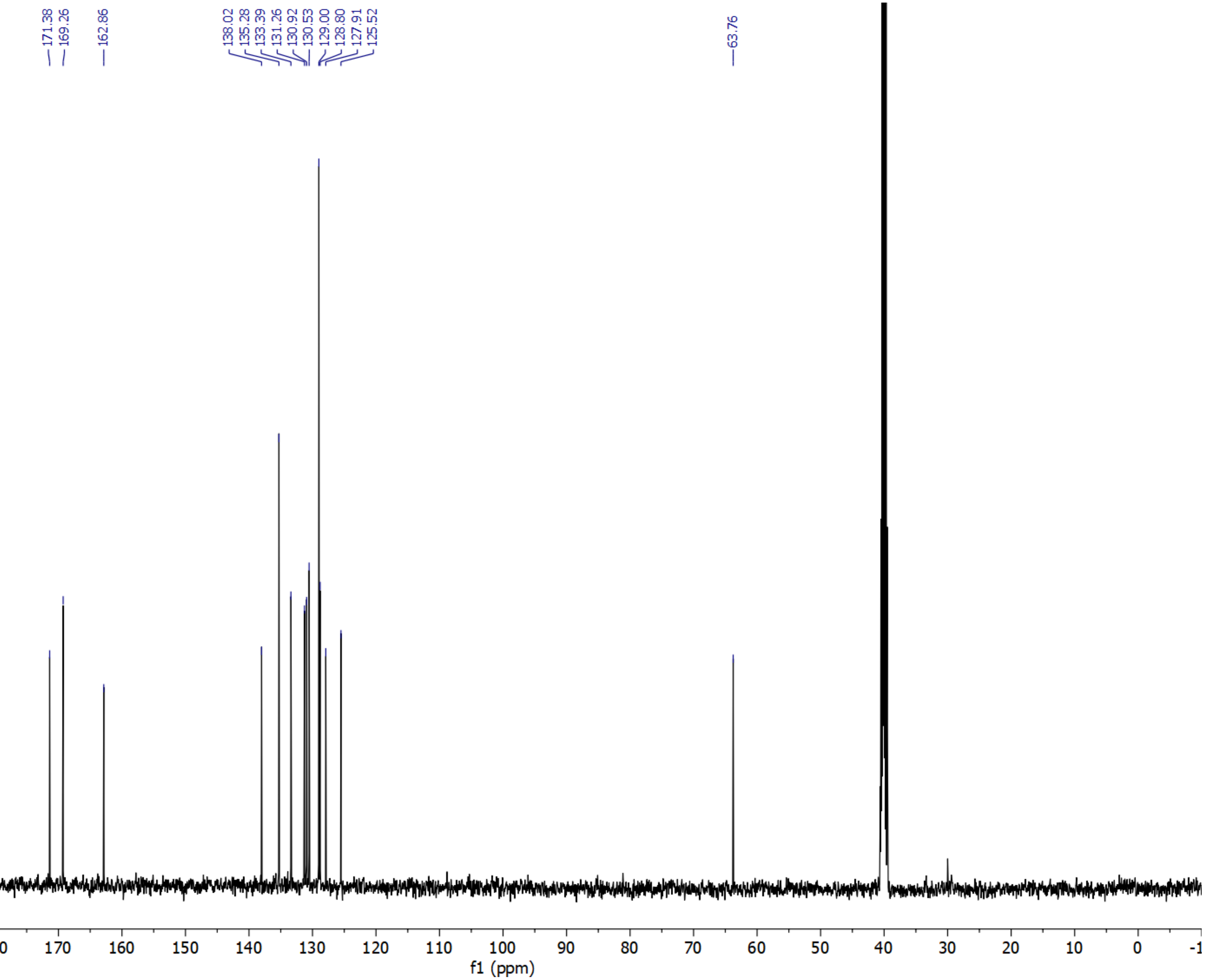

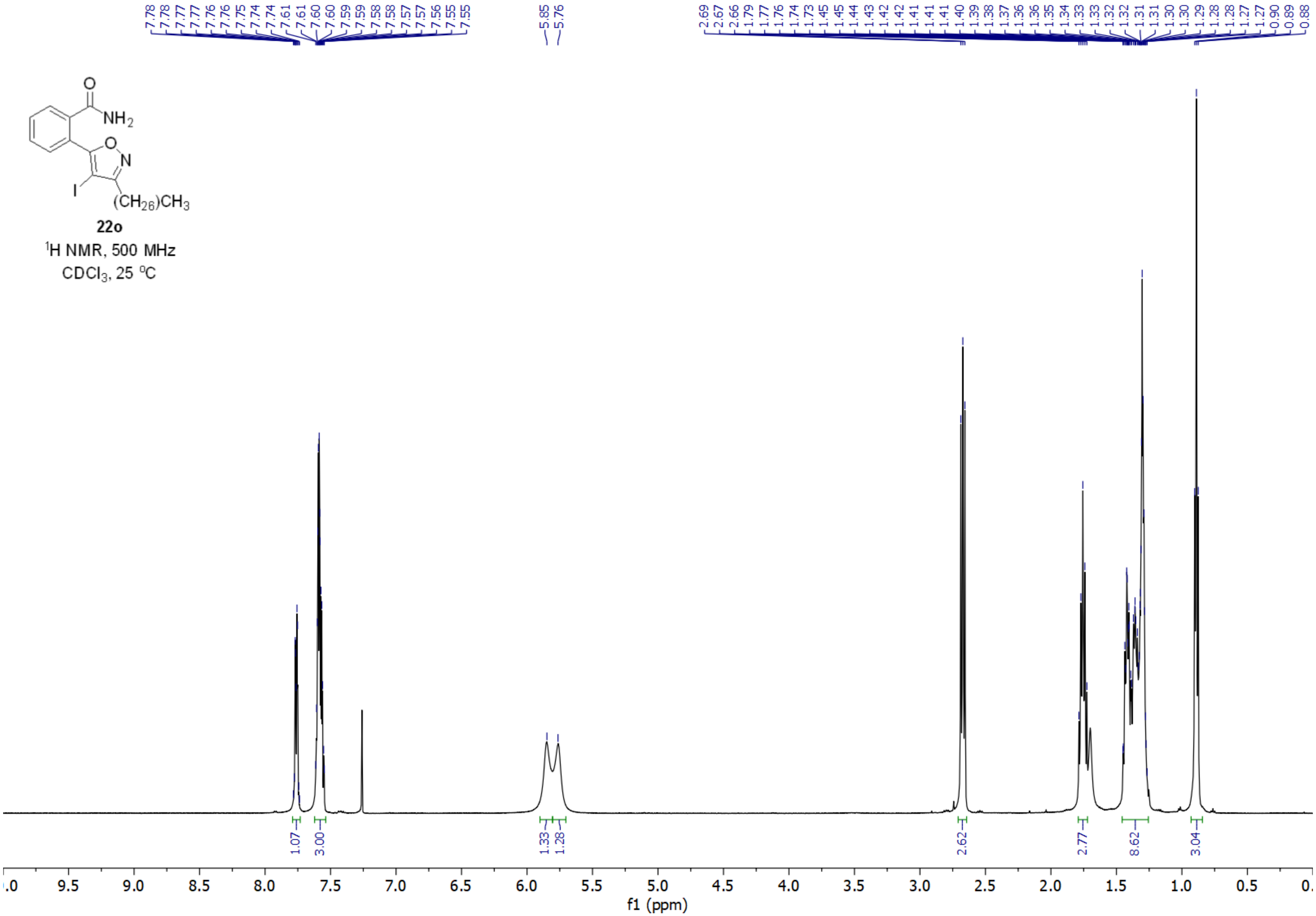

22o

^{1}H NMR, 500 MHz

$CDCl_3$, 25 °C

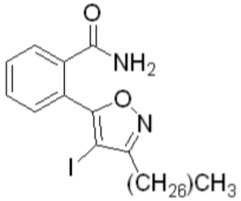

22o

^{13}C NMR, 125 MHz

$CDCl_3$, 25 °C

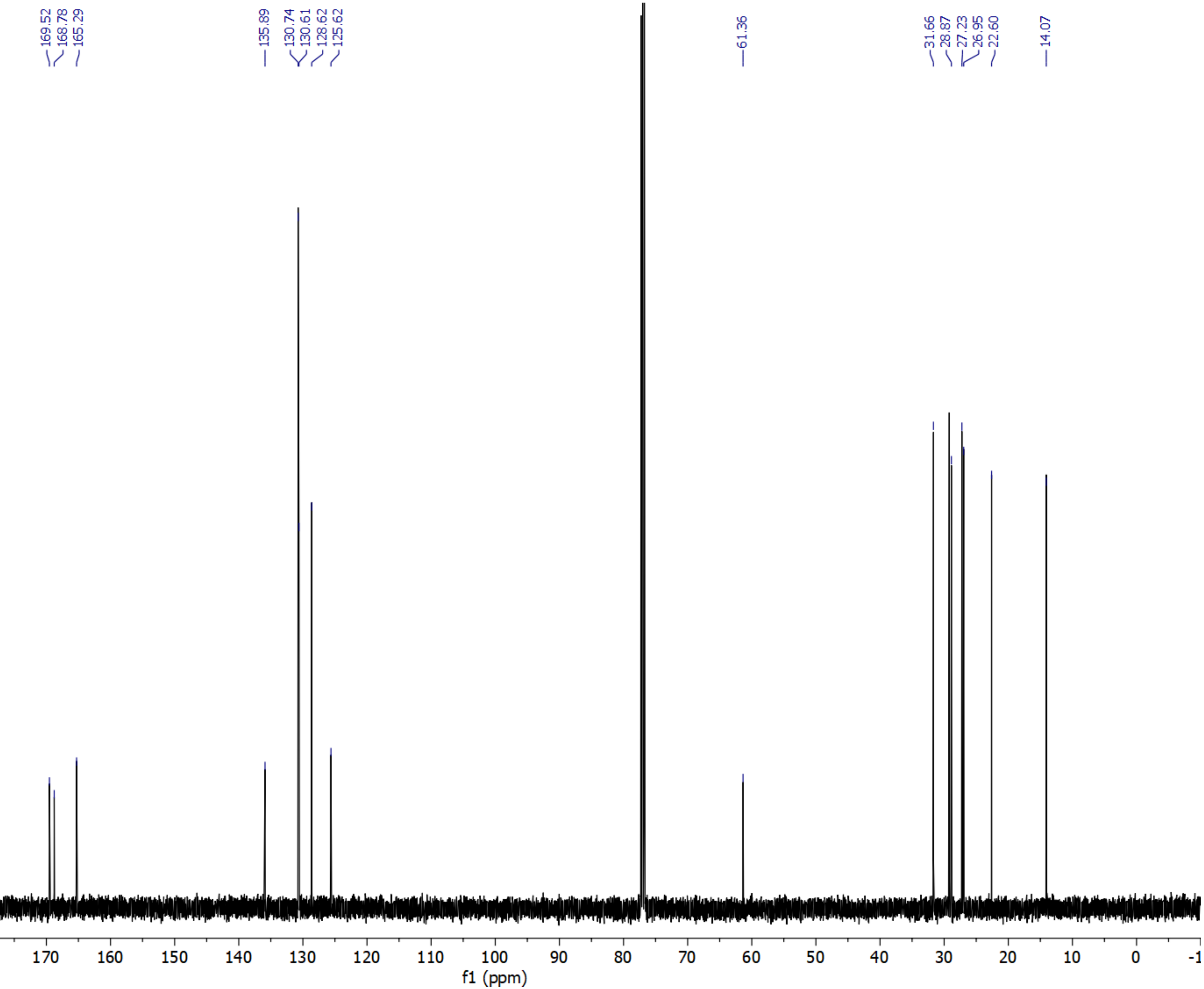

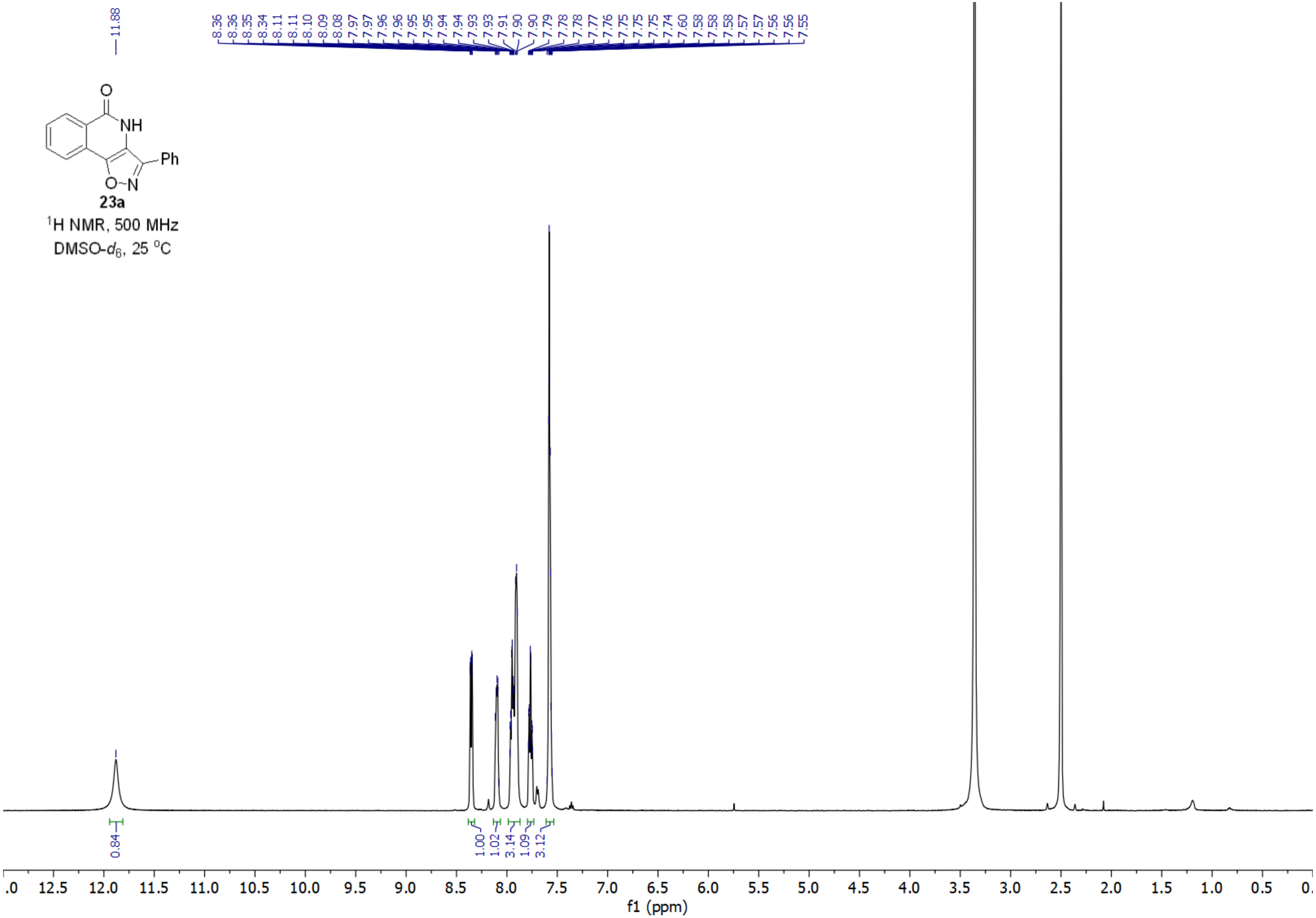
23a
^{1}H NMR, 500 MHz
DMSO-d_6, 25 °C
f1 (ppm)

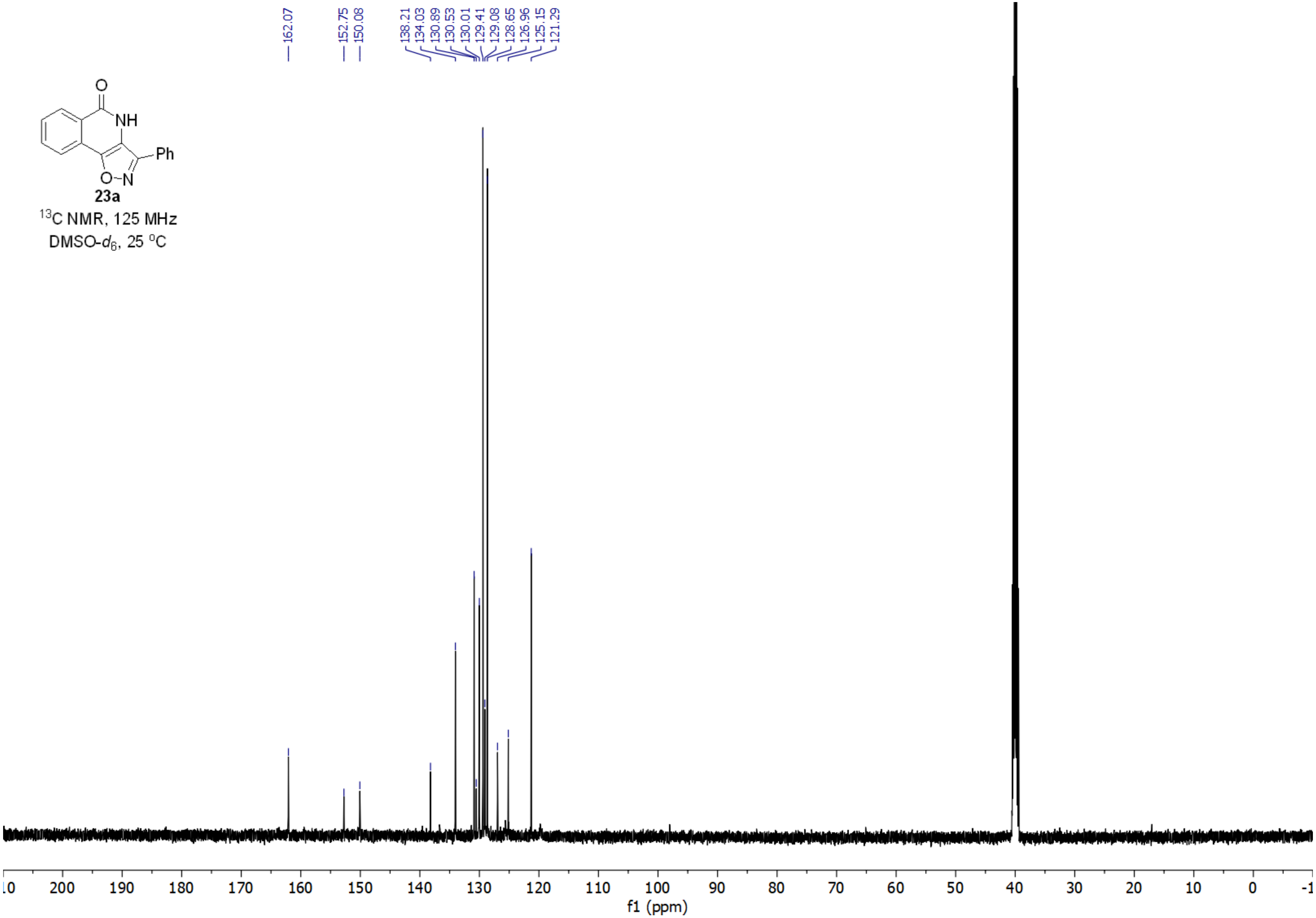
162.07
152.75
150.08
138.21
134.03
130.89
130.53
130.01
129.41
129.08
128.65
126.96
125.15
121.29
O
NH
Ph
O
N
23a
^{13}C NMR, 125 MHz
DMSO-d_6, 25 °C
200
190
180
170
160
150
140
130
120
110
100
90
80
70
60
50
40
30
20
10
0
f1 (ppm)

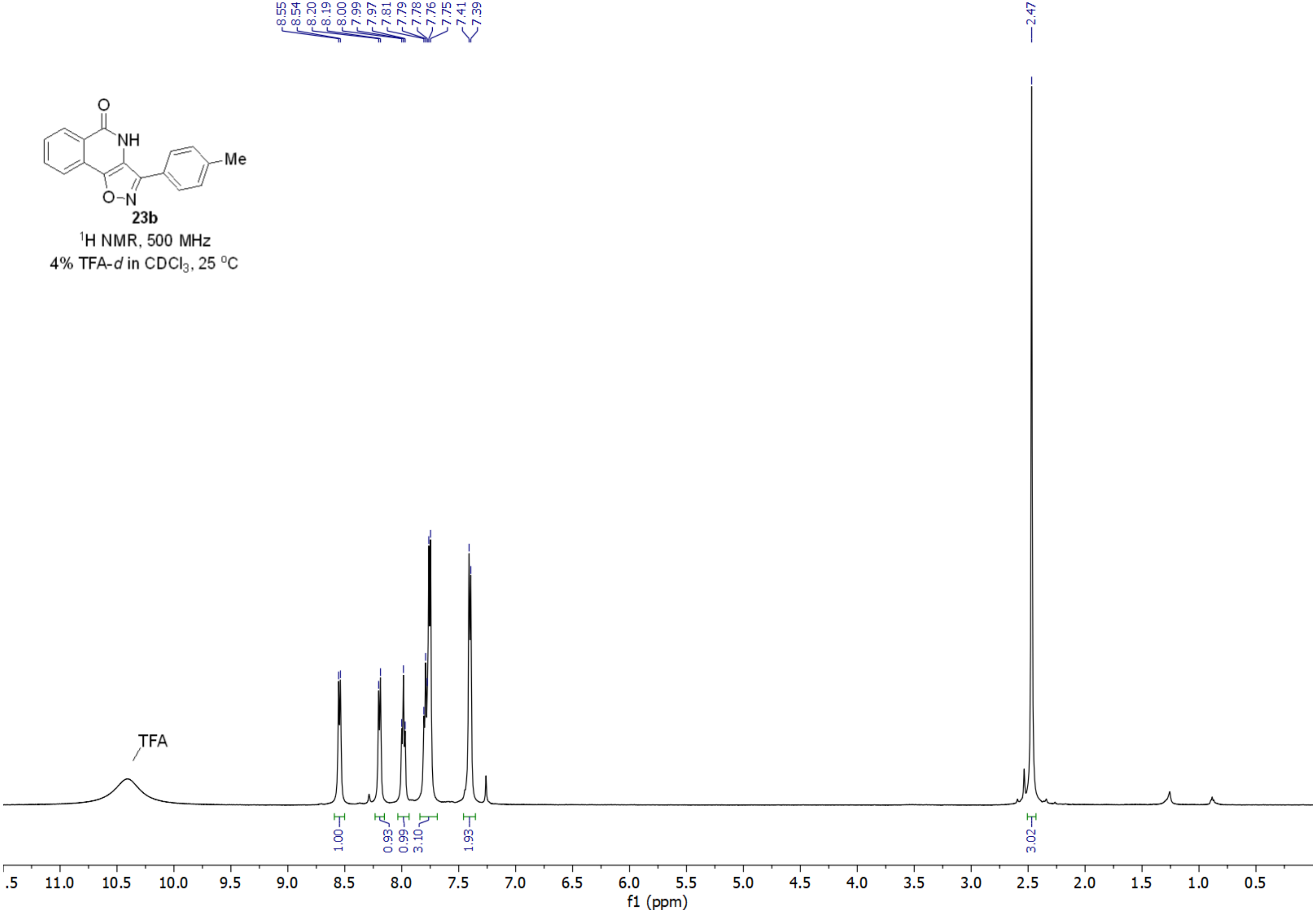

8.55
8.54
8.20
8.19
8.00
7.99
7.97
7.81
7.79
7.78
7.76
7.75
7.41
7.39
2.47
O
NH
Me
O–N
23b
^{1}H NMR, 500 MHz
4% TFA-*d* in $CDCl_3$, 25 °C
TFA
1.00
0.93
0.99
3.10
1.93
3.02
f1 (ppm)

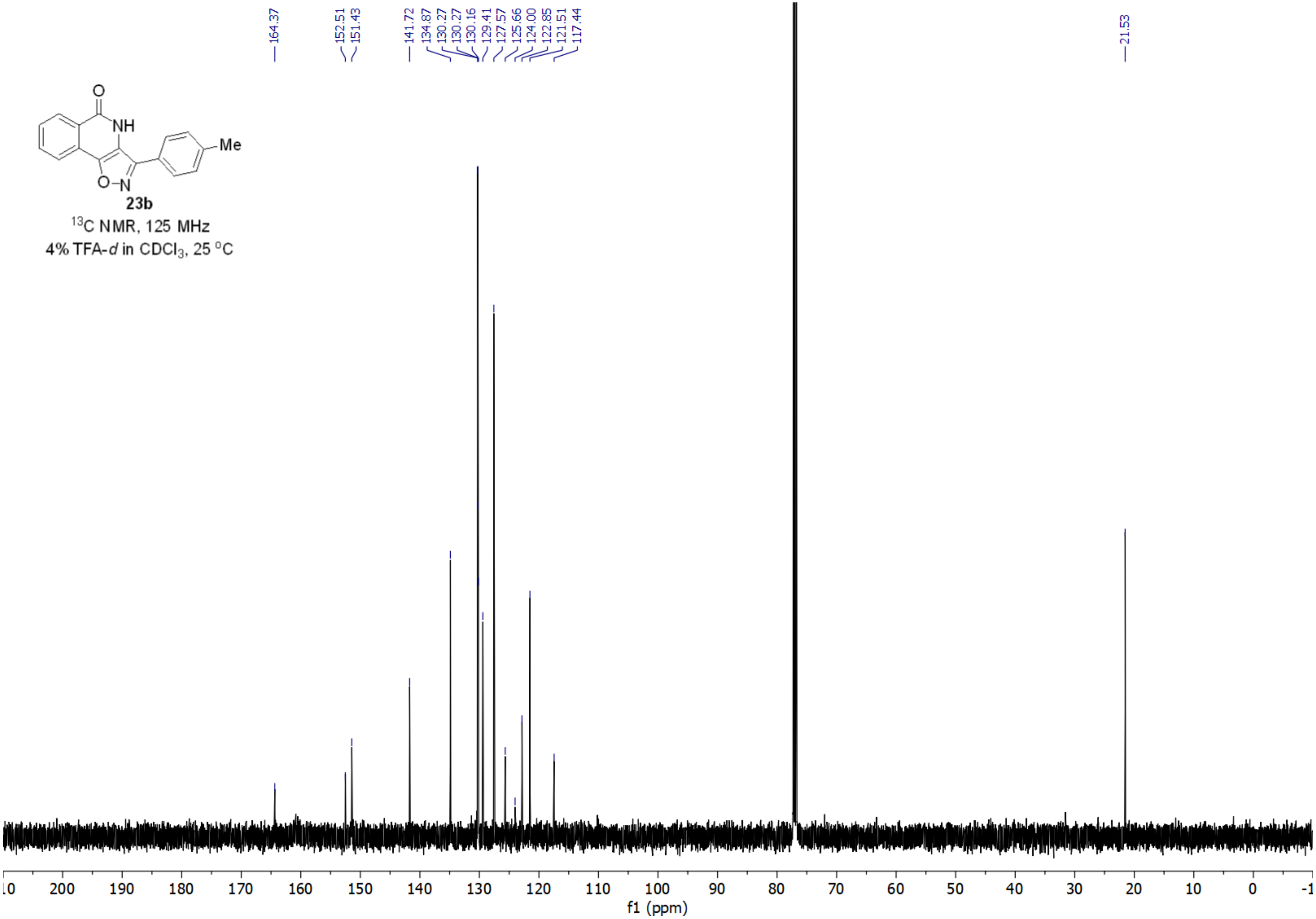

164.37
152.51
151.43
141.72
134.87
130.27
130.27
130.16
129.41
127.57
125.66
124.00
122.85
121.51
117.44
21.53
O
NH
Me
O
N
23b
^{13}C NMR, 125 MHz
4% TFA-*d* in $CDCl_3$, 25 °C
f1 (ppm)

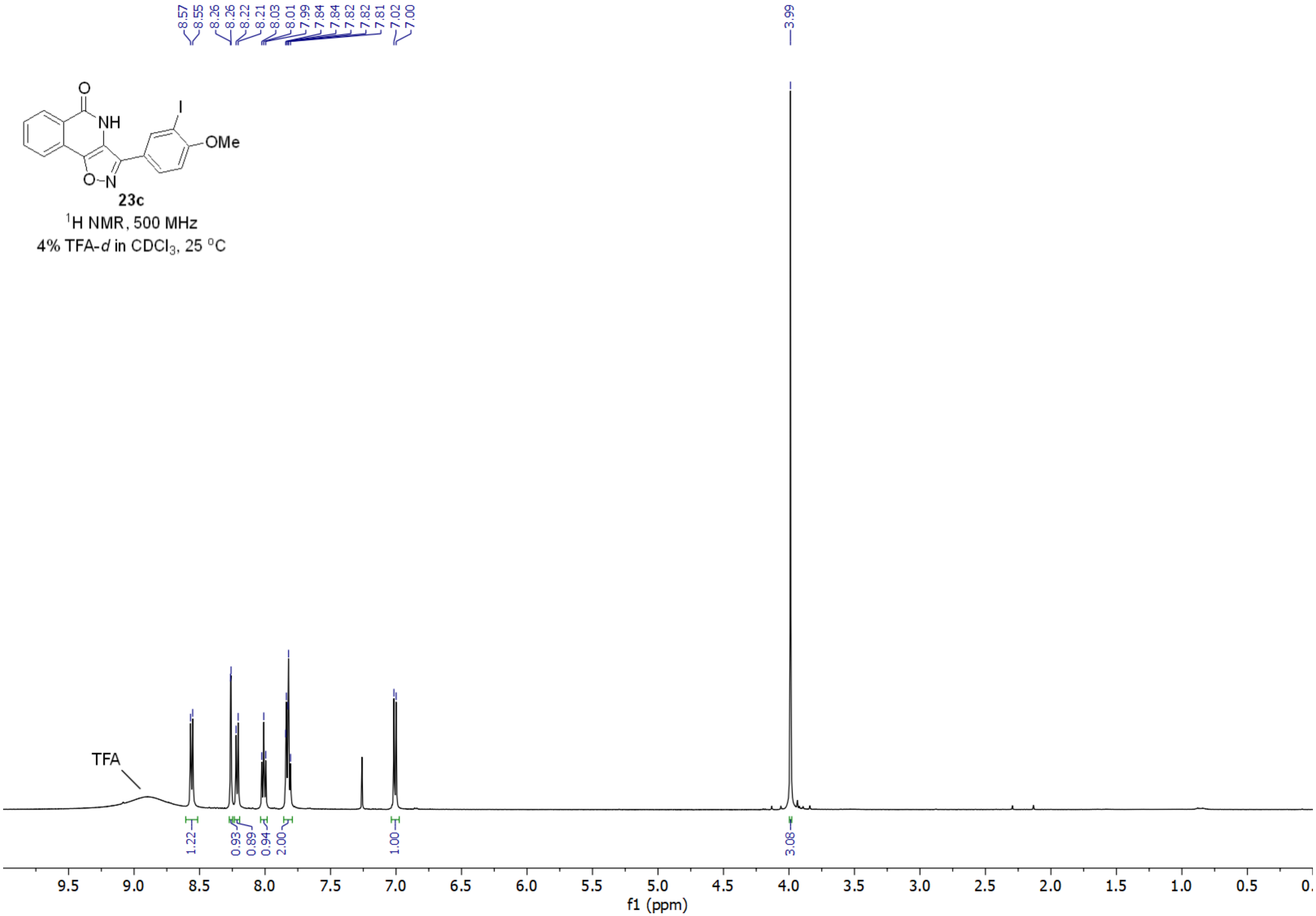

8.57
8.55
8.26
8.26
8.22
8.21
8.03
8.01
7.99
7.84
7.84
7.82
7.82
7.81
7.02
7.00
3.99
O
NH
I
OMe
O
N
23c
^{1}H NMR, 500 MHz
4% TFA-*d* in $CDCl_3$, 25 °C
TFA
1.22
0.93
0.89
0.94
2.00
1.00
3.08
9.5
9.0
8.5
8.0
7.5
7.0
6.5
6.0
5.5
5.0
4.5
4.0
3.5
3.0
2.5
2.0
1.5
1.0
0.5
0.
f1 (ppm)

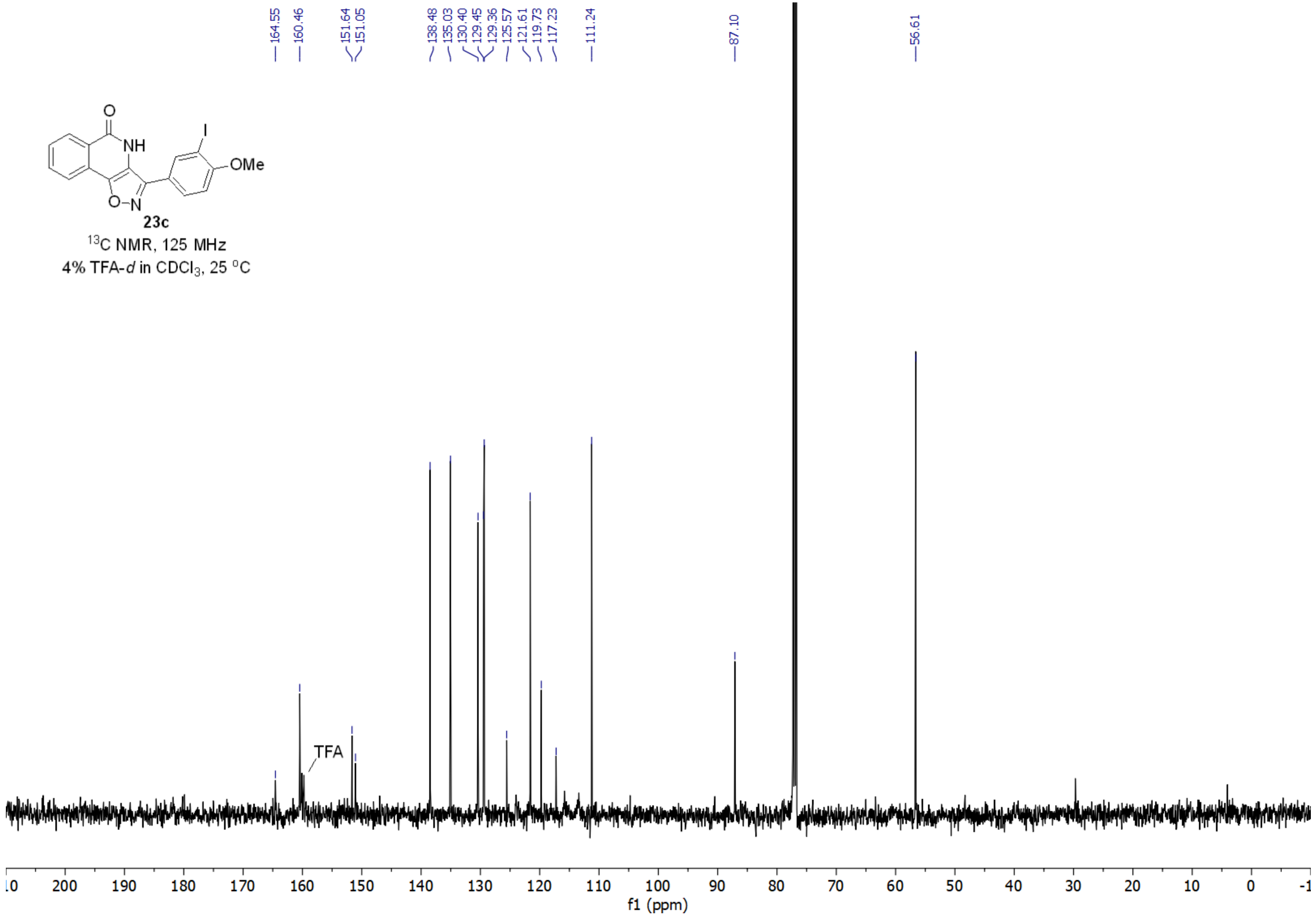
164.55
160.46
151.64
151.05
138.48
135.03
130.40
129.45
129.36
125.57
121.61
119.73
117.23
111.24
87.10
56.61
O
NH
I
OMe
O
N
23c
^{13}C NMR, 125 MHz
4% TFA-*d* in $CDCl_3$, 25 °C
TFA
f1 (ppm)

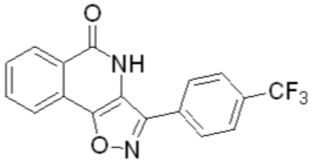

23d

^{1}H NMR, 500 MHz

4% TFA-*d* in $CDCl_3$, 25 °C

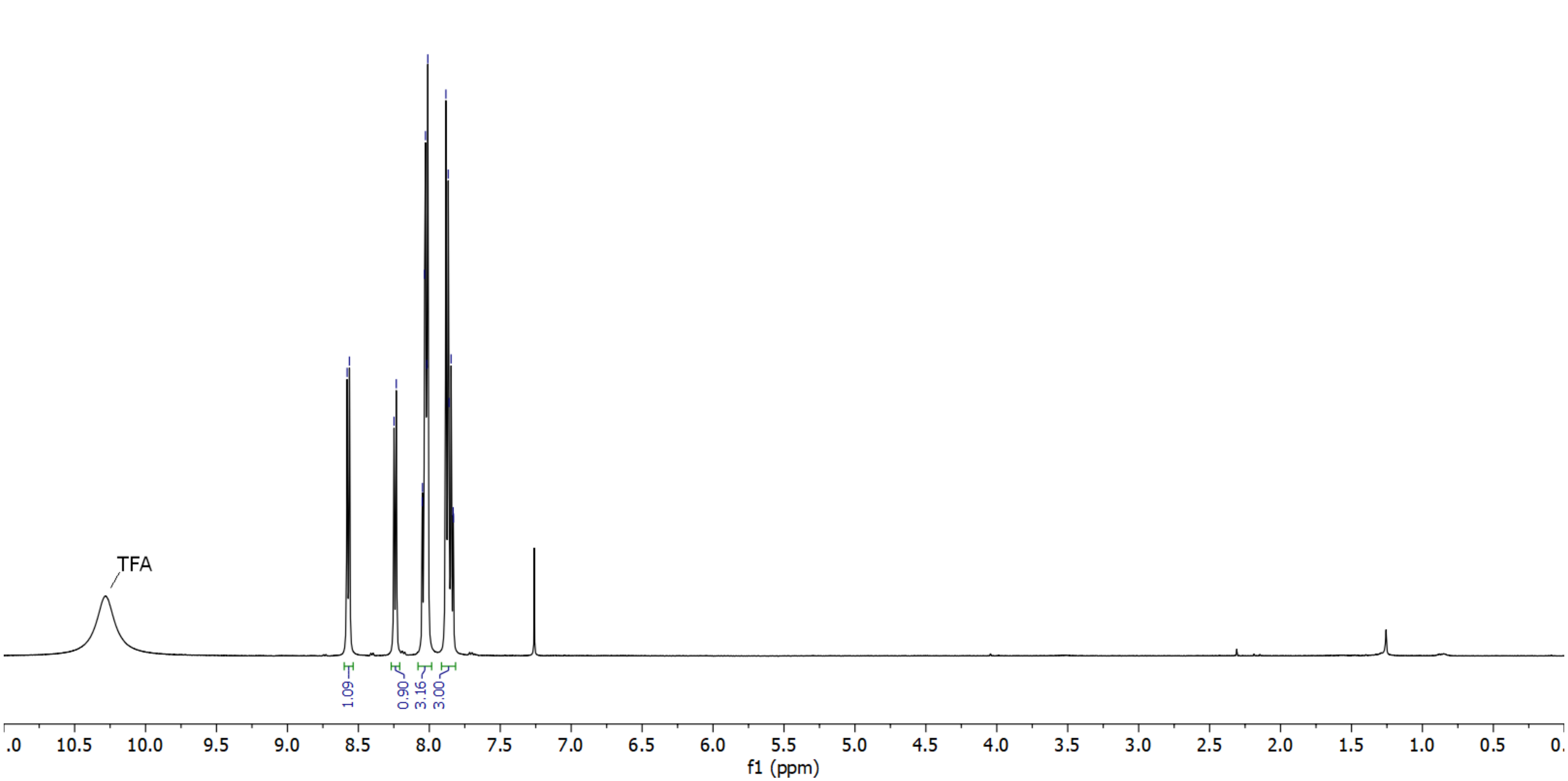

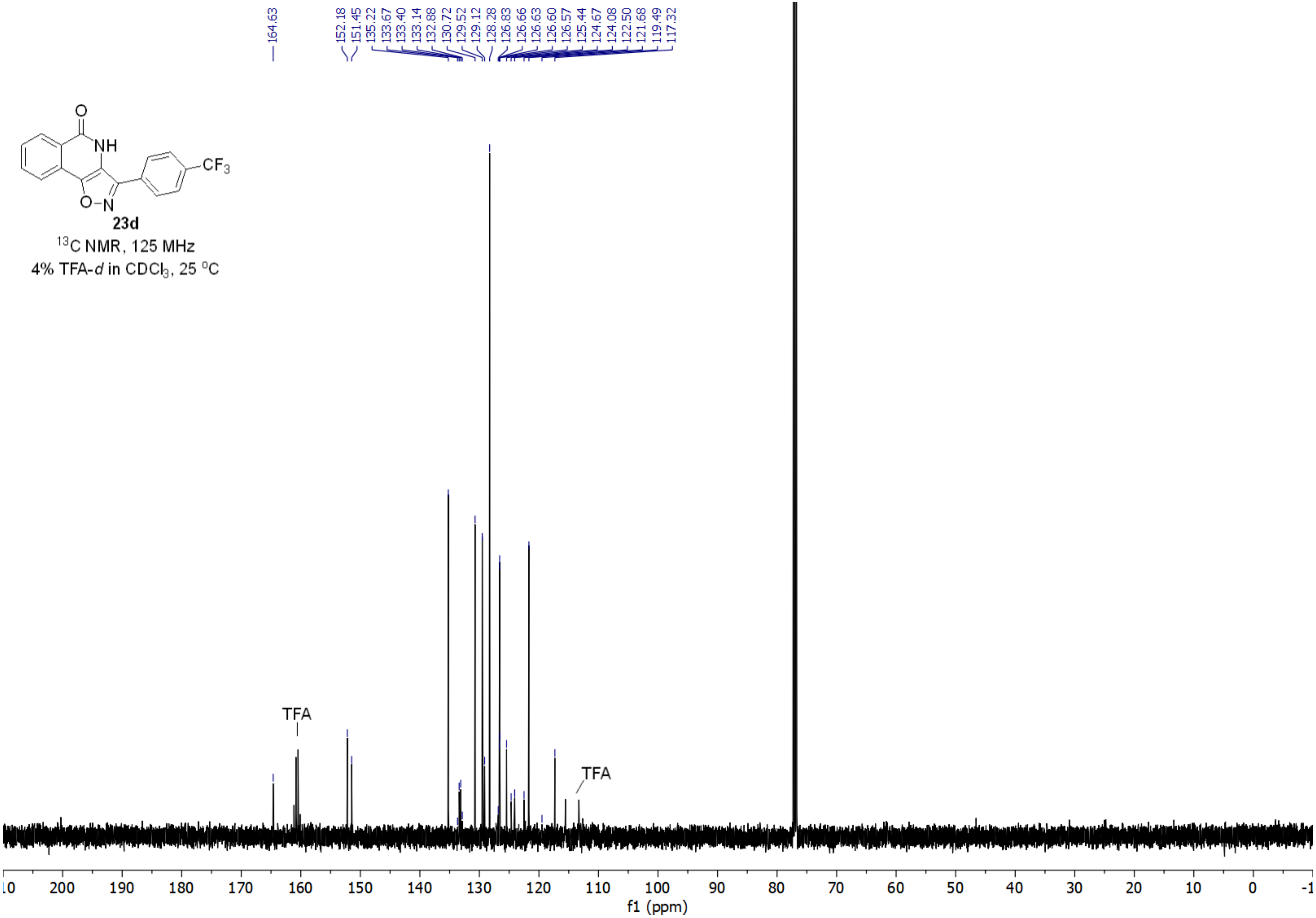
164.63
152.18
151.45
135.22
133.67
133.40
133.14
132.88
130.72
129.52
129.12
128.28
126.83
126.66
126.63
126.60
126.57
125.44
124.67
124.08
122.50
121.68
119.49
117.32
23d
^{13}C NMR, 125 MHz
4% TFA-*d* in $CDCl_3$, 25 °C
TFA
TFA
f1 (ppm)

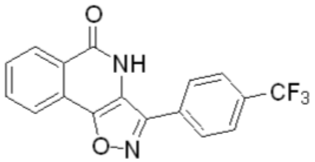

23d

^{19}F NMR, 470 MHz

4% TFA-*d* in $CDCl_3$, 25 °C

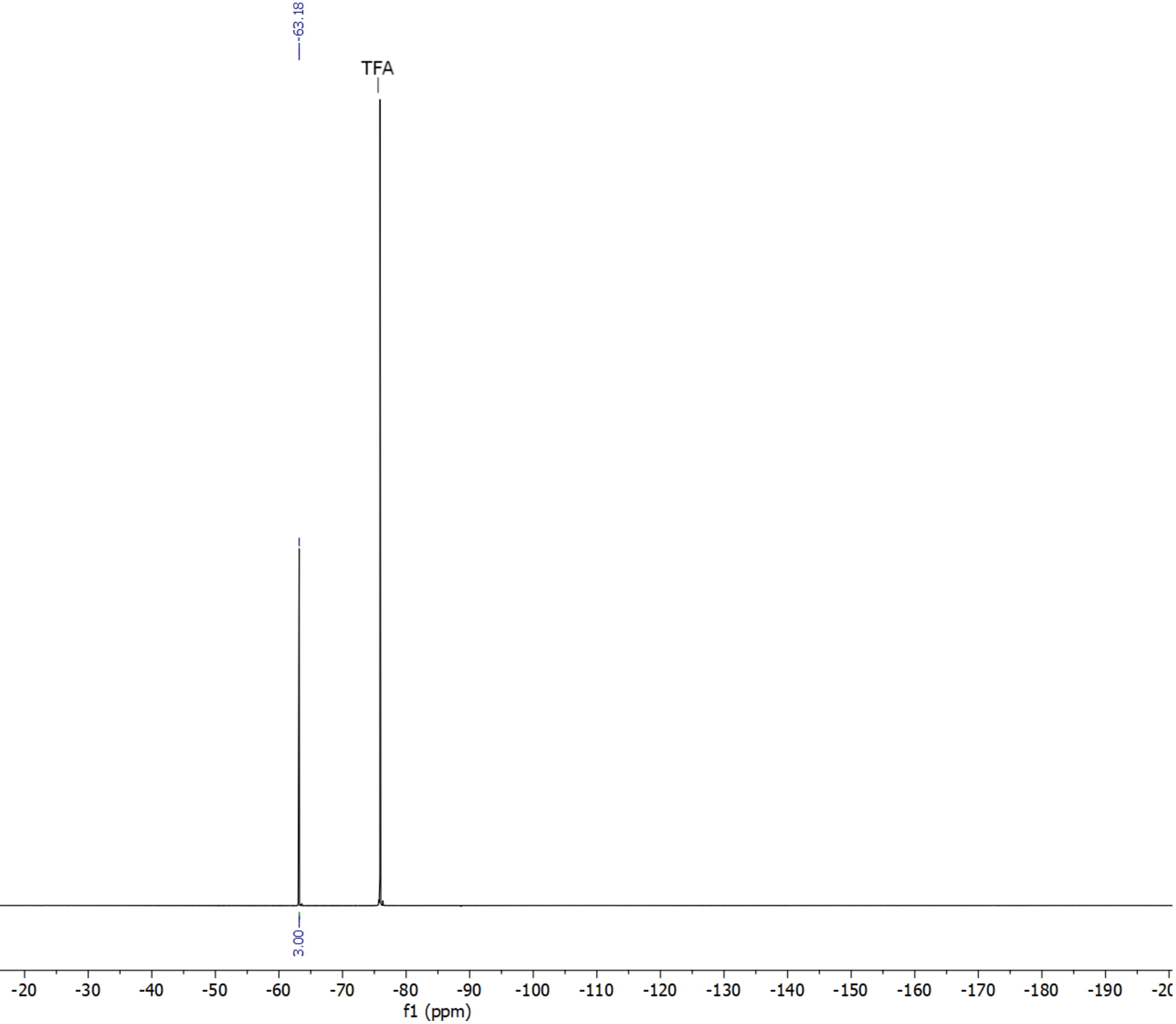

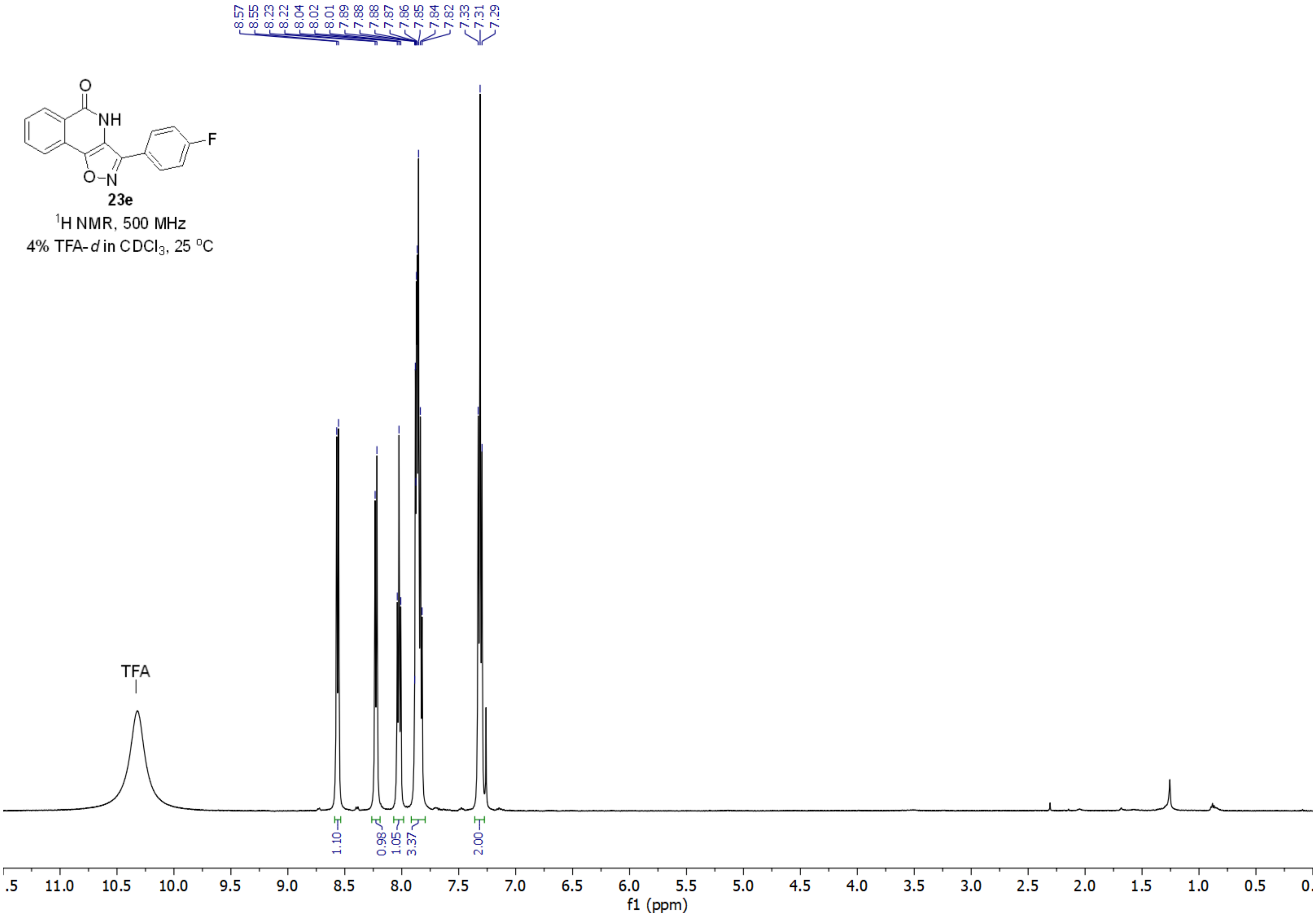

8.57
8.55
8.23
8.22
8.04
8.02
8.01
7.89
7.88
7.88
7.87
7.86
7.85
7.84
7.82
7.33
7.31
7.29
O
NH
F
O–N
23e
^{1}H NMR, 500 MHz
4% TFA-d in CDCl$_3$, 25 °C
TFA
1.10
0.98
1.05
3.37
2.00
.5
11.0
10.5
10.0
9.5
9.0
8.5
8.0
7.5
7.0
6.5
6.0
5.5
5.0
4.5
4.0
3.5
3.0
2.5
2.0
1.5
1.0
0.5
0.
f1 (ppm)

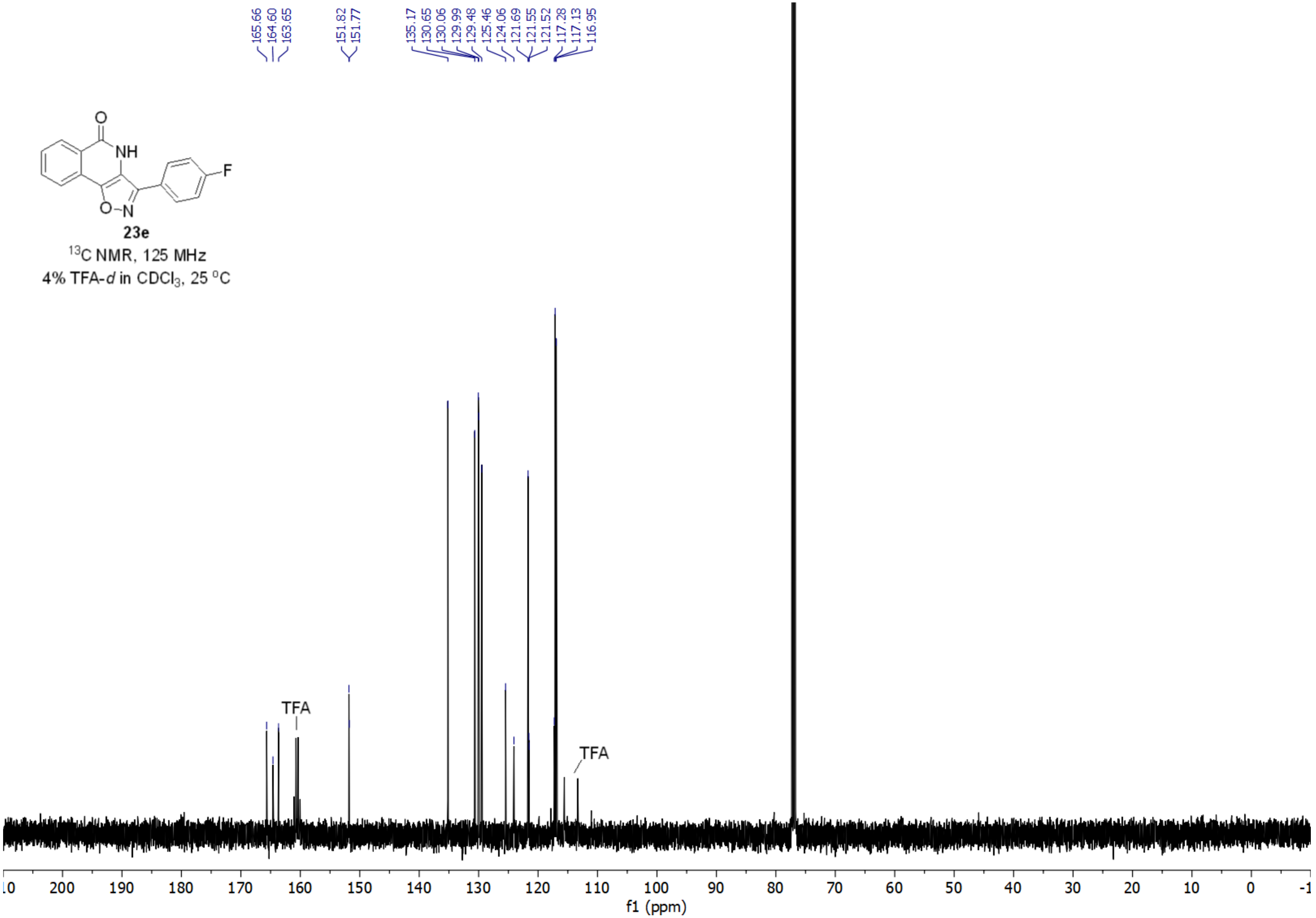
165.66
164.60
163.65
151.82
151.77
135.17
130.65
130.06
129.99
129.48
125.46
124.06
121.69
121.55
121.52
117.28
117.13
116.95
O
NH
F
O
N
23e
^{13}C NMR, 125 MHz
4% TFA-*d* in $CDCl_3$, 25 °C
TFA
TFA
200
190
180
170
160
150
140
130
120
110
100
90
80
70
60
50
40
30
20
10
0
f1 (ppm)

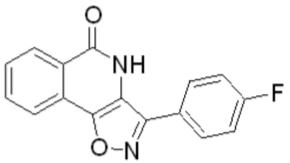

23e

^{19}F NMR, 470 MHz

4% TFA-*d* in $CDCl_3$, 25 °C

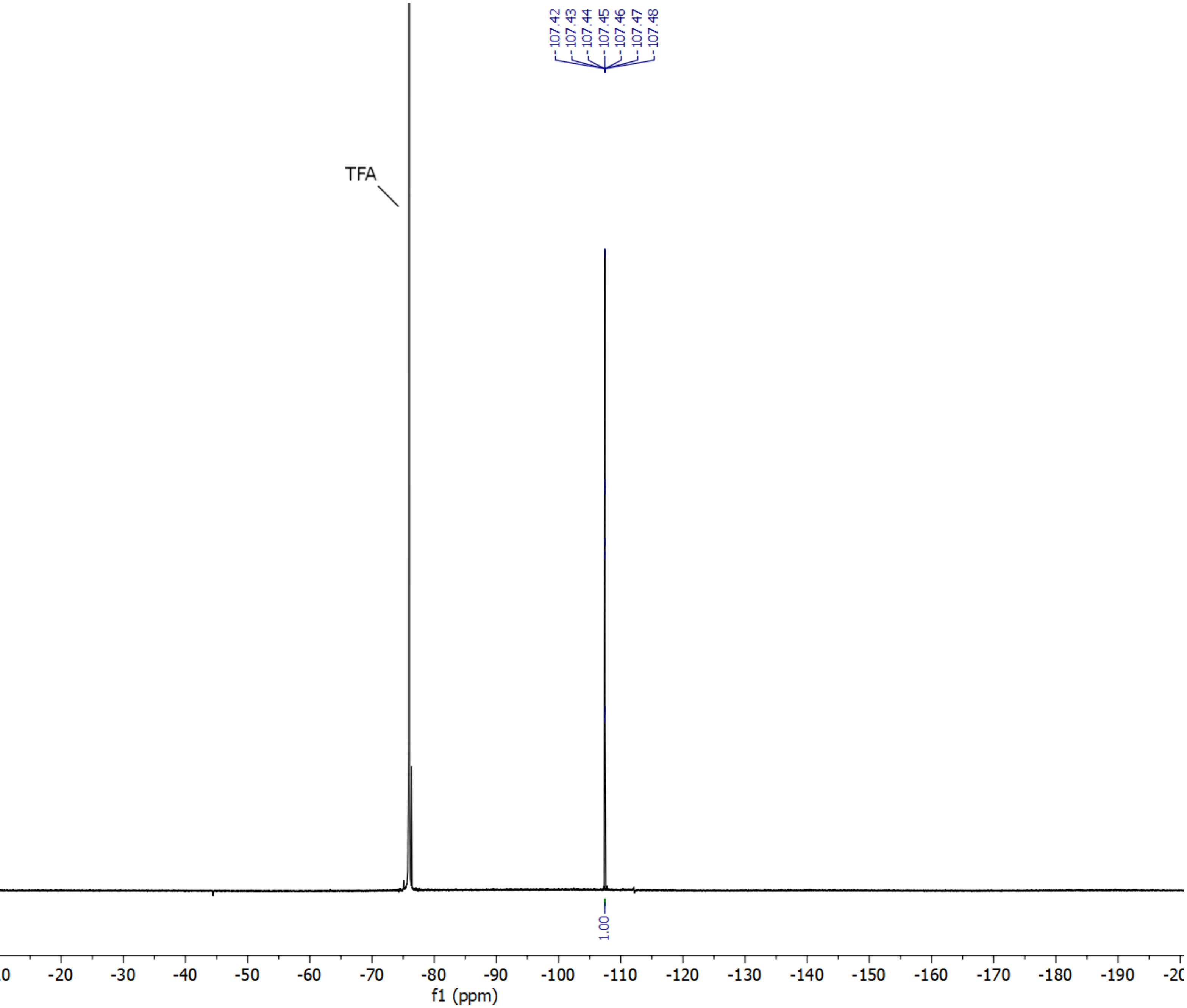

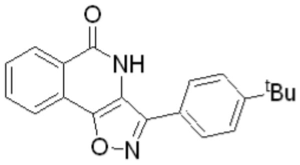

23f

^{1}H NMR, 500 MHz

4% TFA-*d* in $CDCl_3$, 25 °C

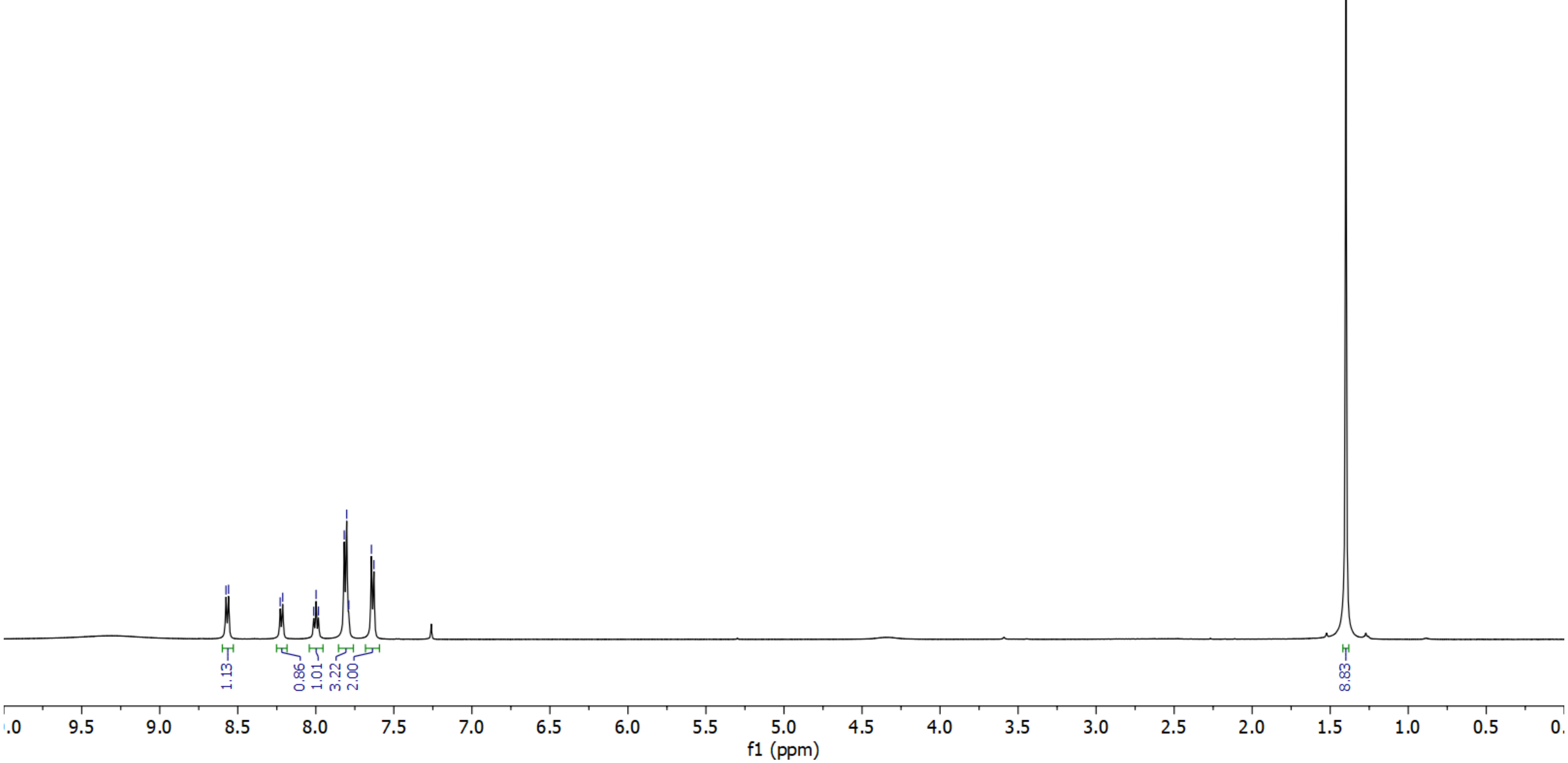

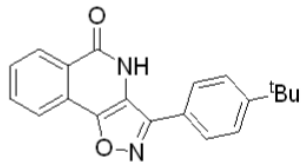

23f

^{13}C NMR, 125 MHz

4% TFA-*d* in $CDCl_3$, 25 °C

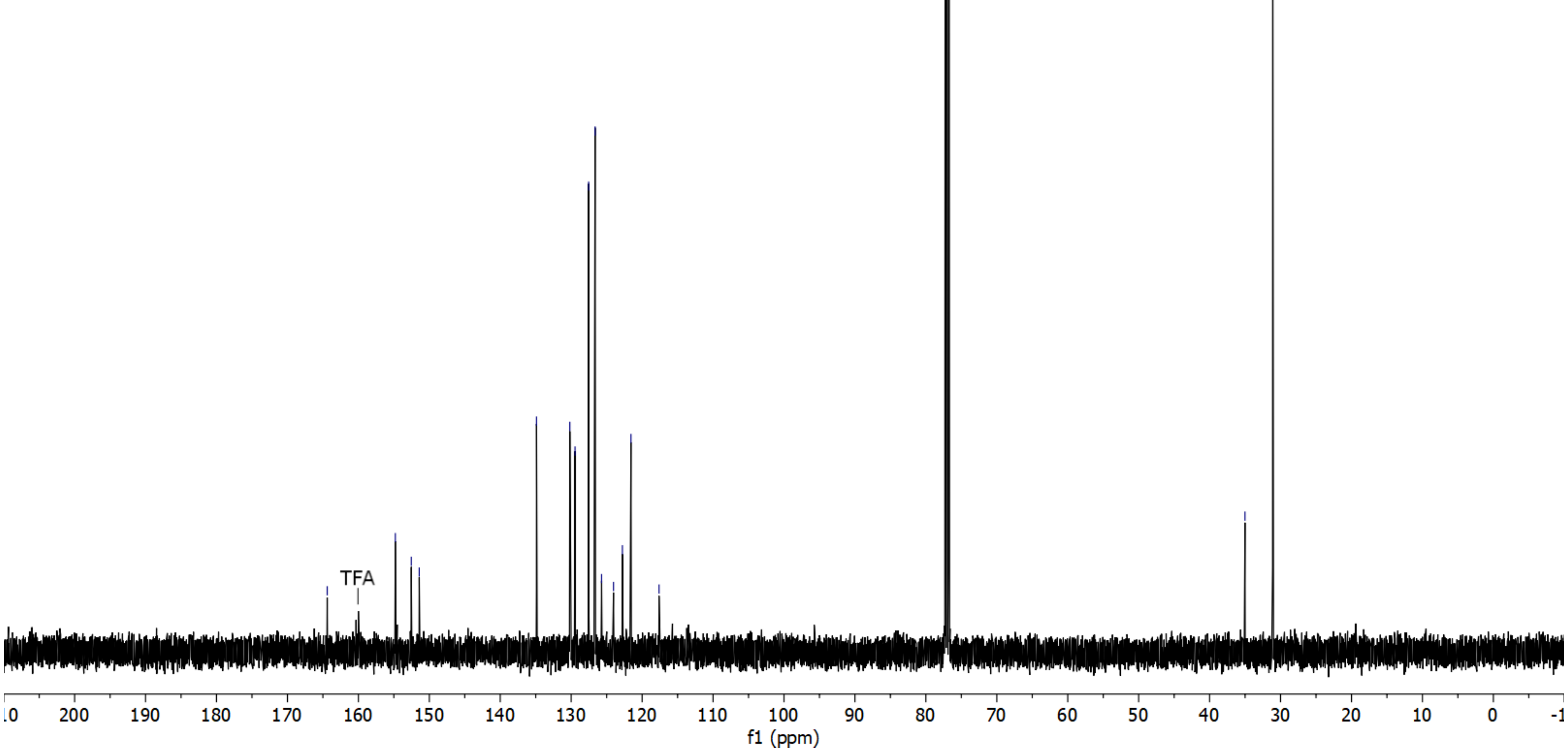

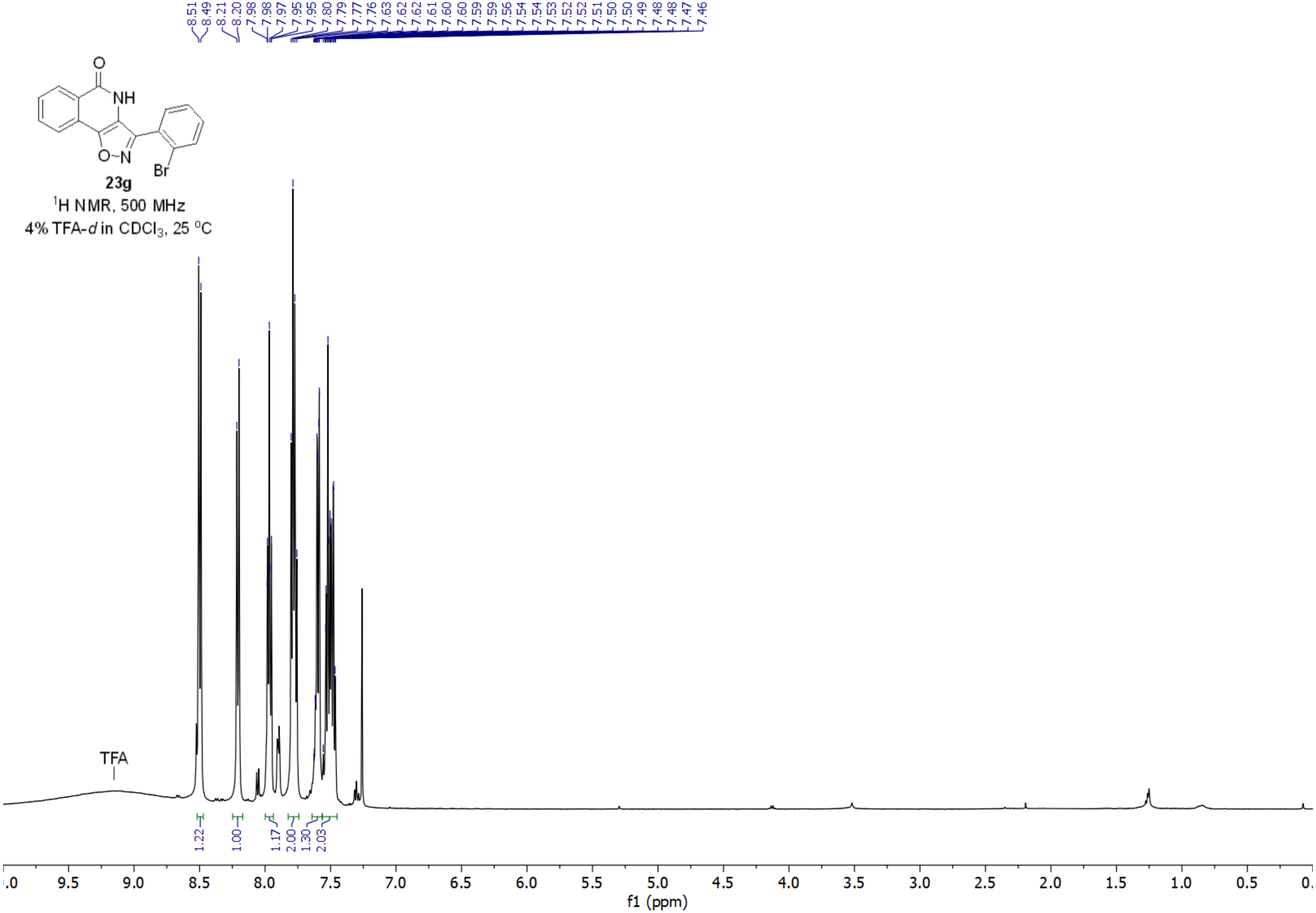

23g
^{1}H NMR, 500 MHz
4% TFA-d in $CDCl_3$, 25 °C
TFA
8.51
8.49
8.21
8.20
7.98
7.98
7.97
7.95
7.95
7.80
7.79
7.77
7.76
7.63
7.62
7.62
7.61
7.60
7.60
7.59
7.59
7.56
7.54
7.54
7.53
7.52
7.52
7.51
7.50
7.50
7.49
7.48
7.48
7.47
7.46
1.22
1.00
1.17
2.00
1.30
2.03
f1 (ppm)

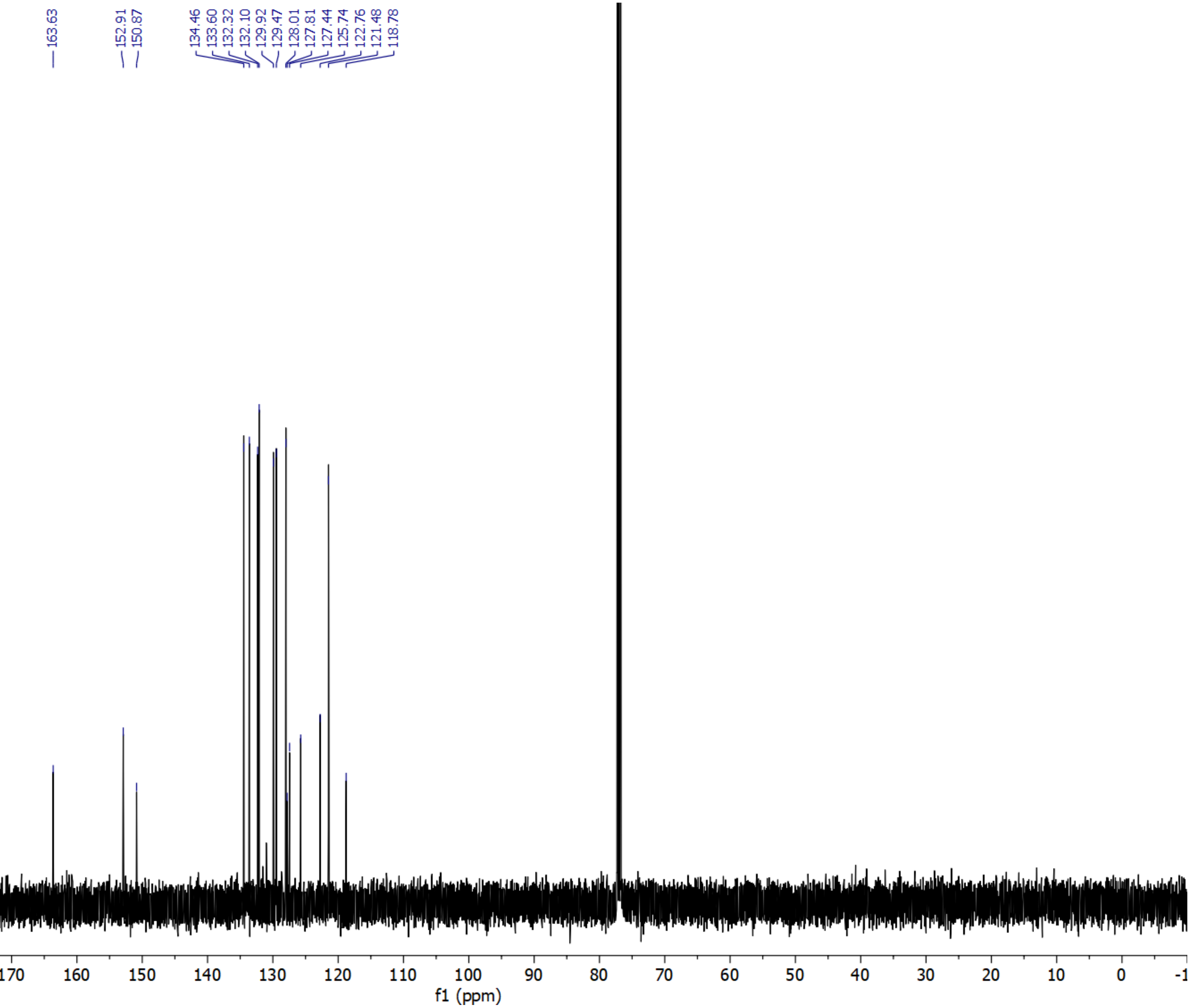

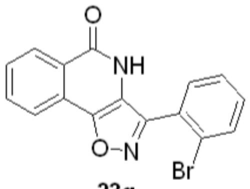

23g

^{13}C NMR, 125 MHz

4% TFA-*d* in $CDCl_3$, 25 °C

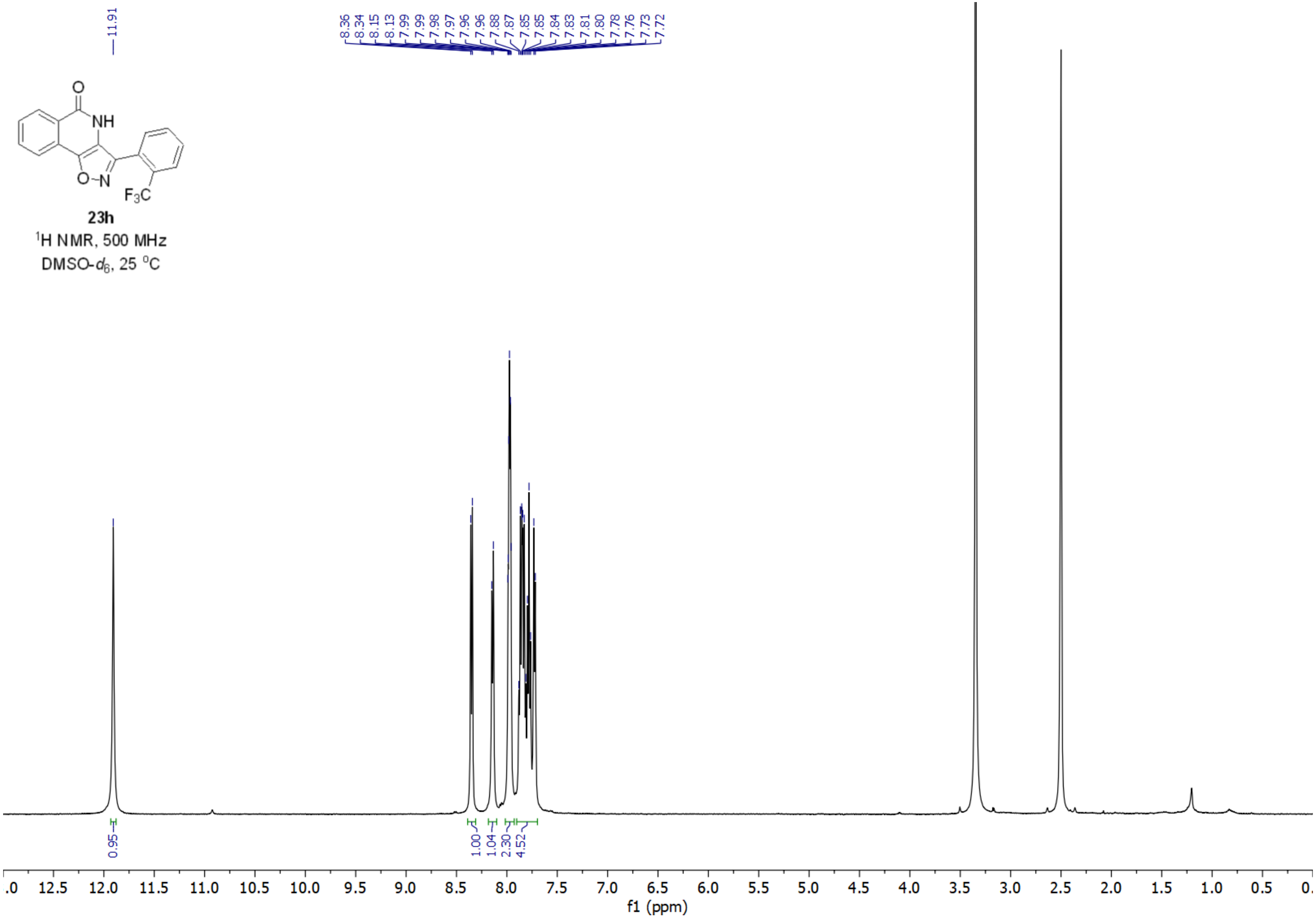
23h
¹H NMR, 500 MHz
DMSO-d6, 25 °C
11.91
8.36 8.34 8.15 8.13 7.99 7.99 7.98 7.97 7.96 7.96 7.88 7.87 7.85 7.85 7.84 7.83 7.81 7.80 7.78 7.76 7.73 7.72
0.95
1.00
1.04
2.30
4.52
f1 (ppm)

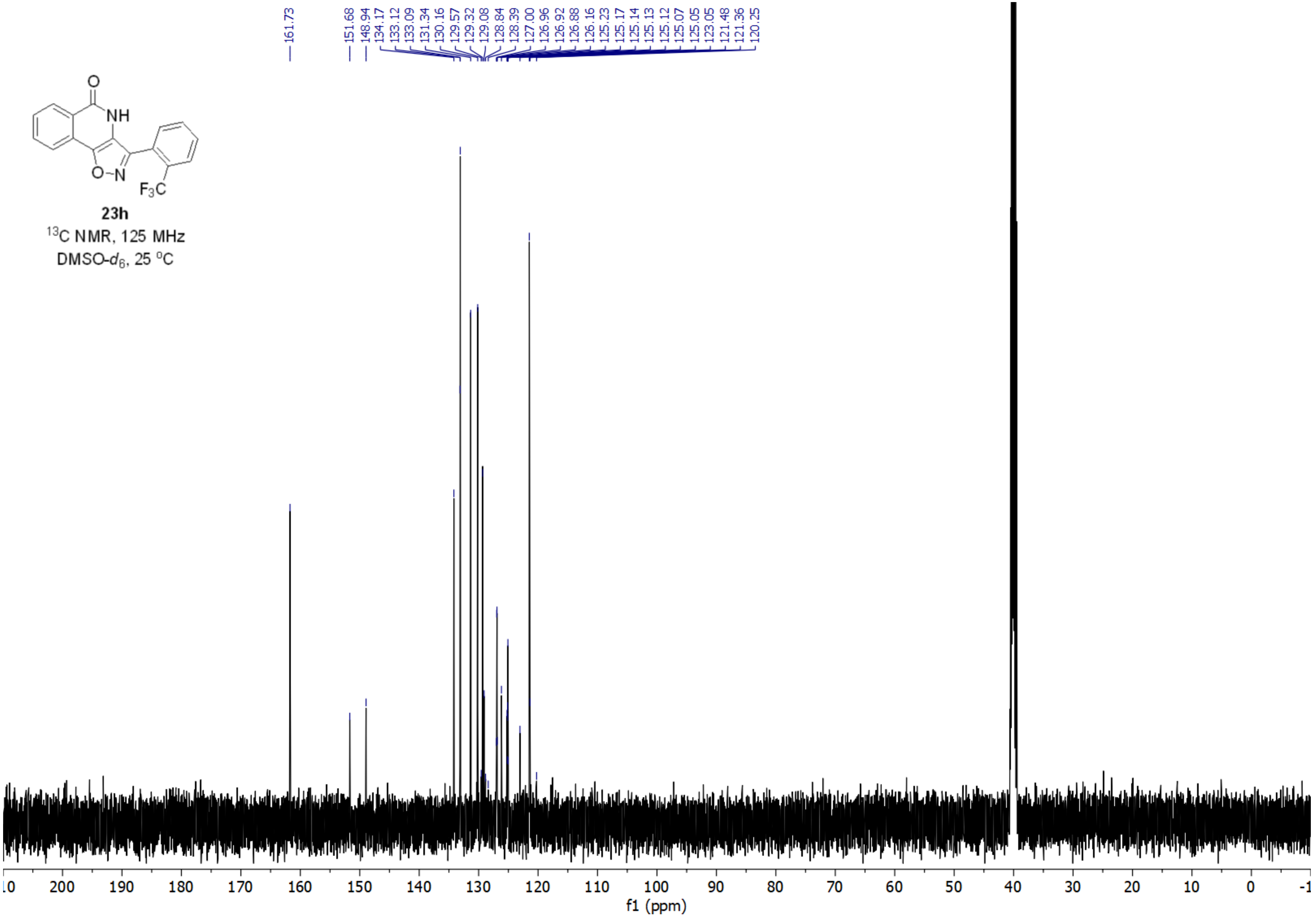
161.73
151.68
148.94
134.17
133.12
133.09
131.34
130.16
129.57
129.32
129.08
128.84
128.39
127.00
126.96
126.92
126.88
126.16
125.23
125.17
125.14
125.13
125.12
125.07
125.05
123.05
121.48
121.36
120.25
O
NH
O
N
F3C
23h
13C NMR, 125 MHz
DMSO-d6, 25 °C
f1 (ppm)

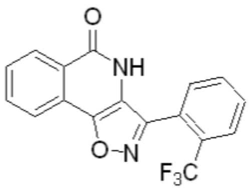

23h

^{19}F NMR, 470 MHz

DMSO-d_6, 25 °C

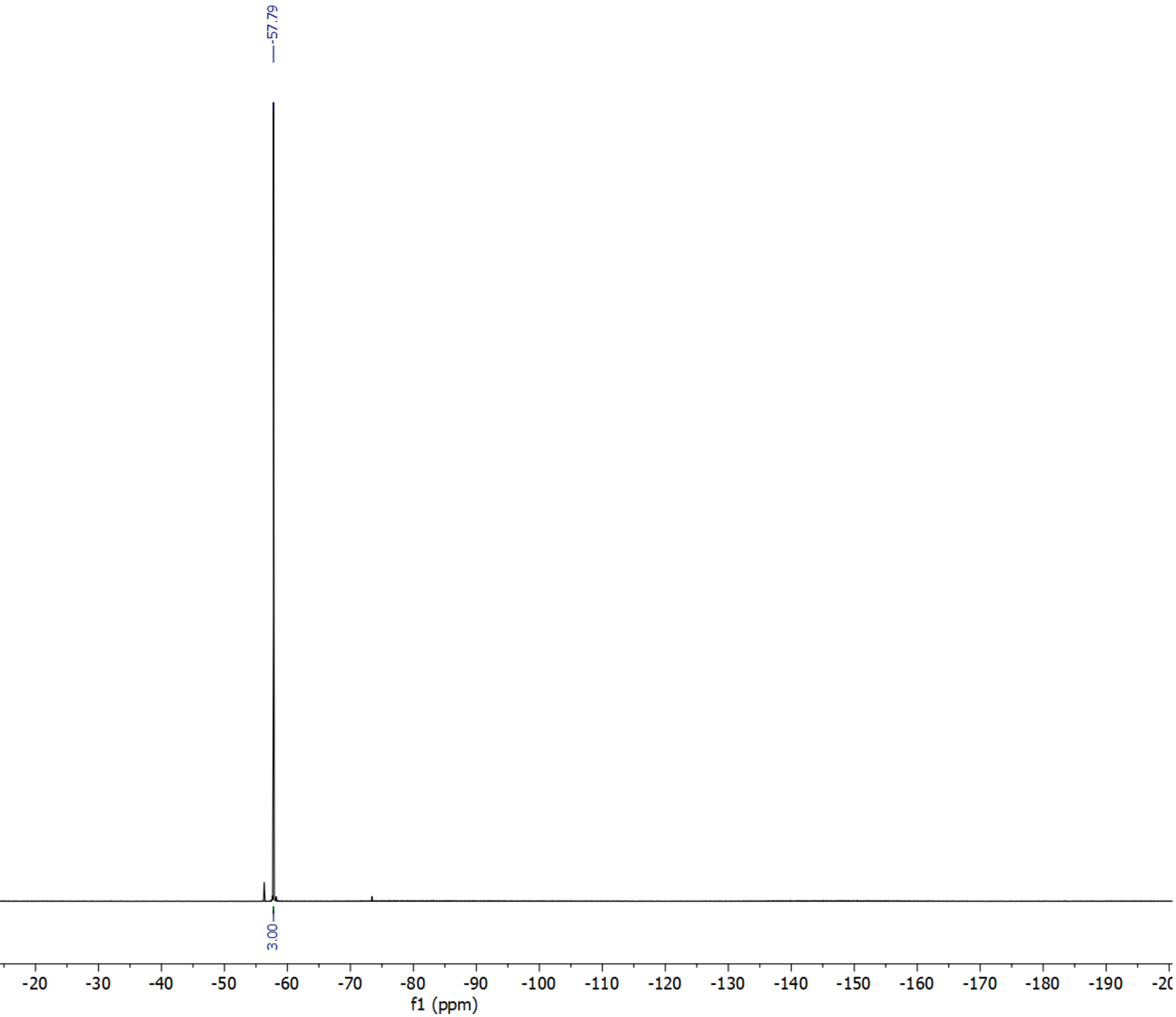

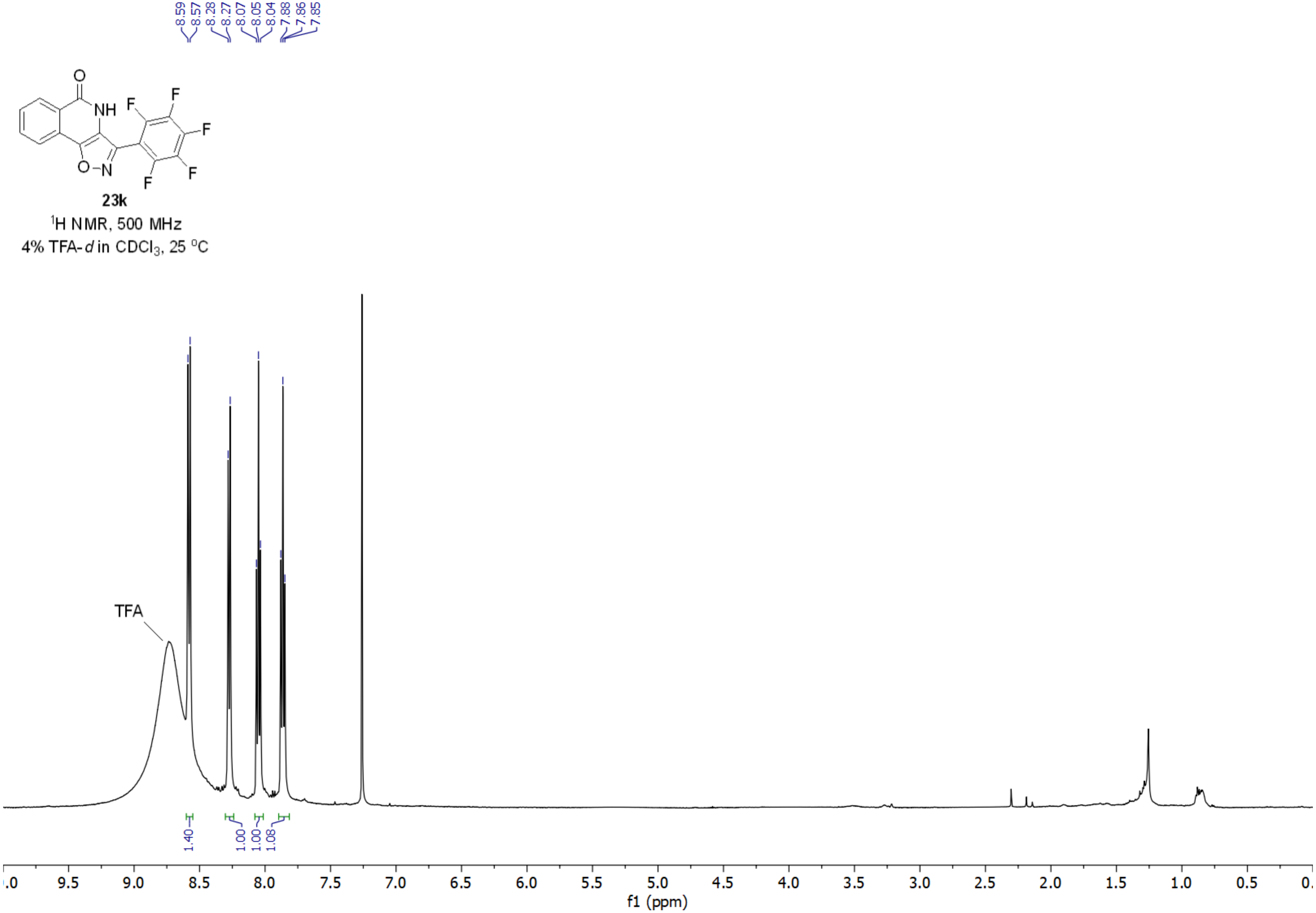

23k

^{1}H NMR, 500 MHz

4% TFA-*d* in $CDCl_3$, 25 ^oC

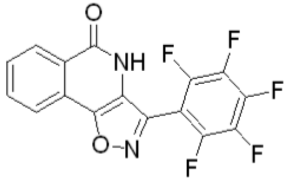

23k

^{13}C NMR, 125 MHz

4% TFA-*d* in $CDCl_3$, 25 °C

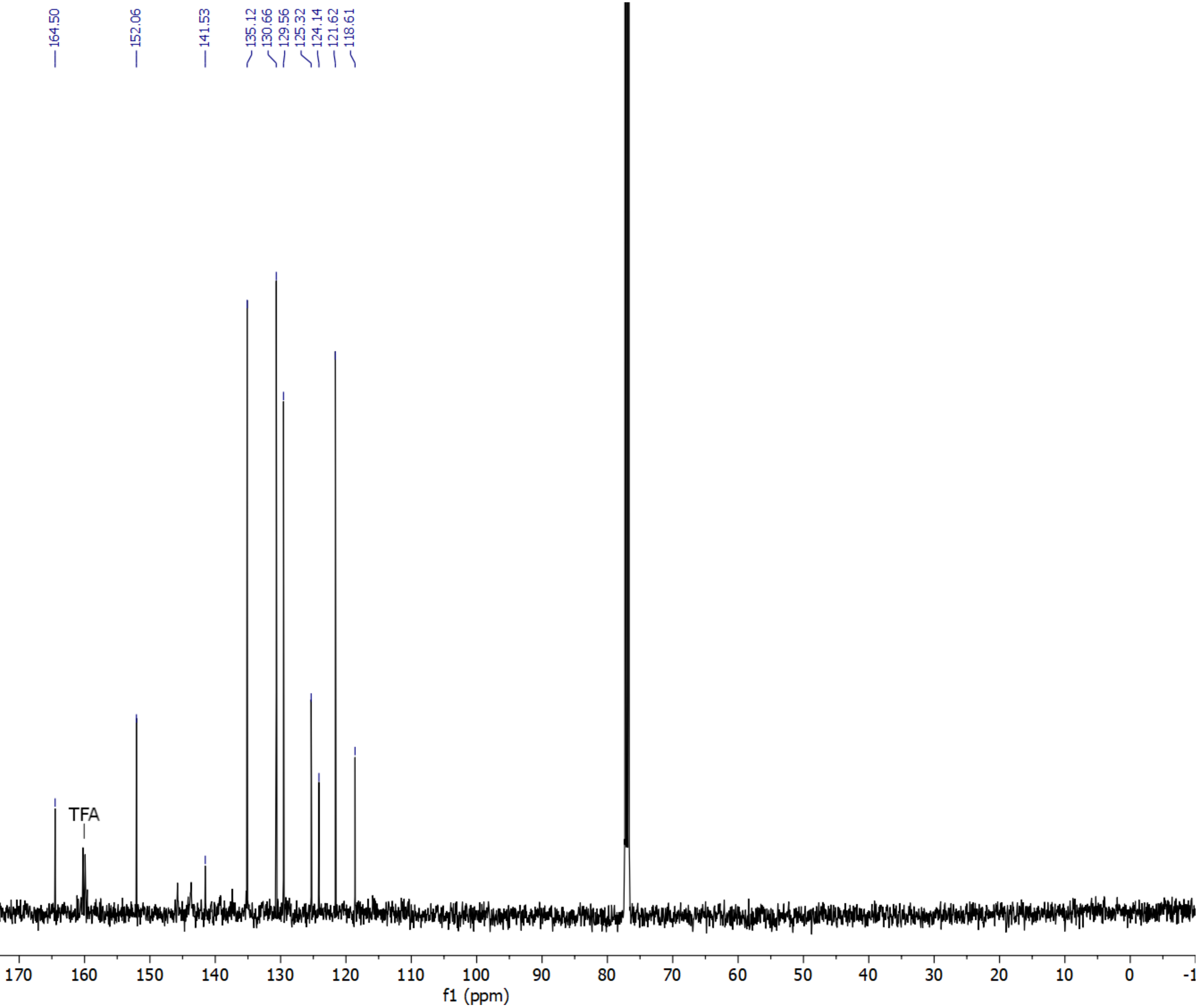

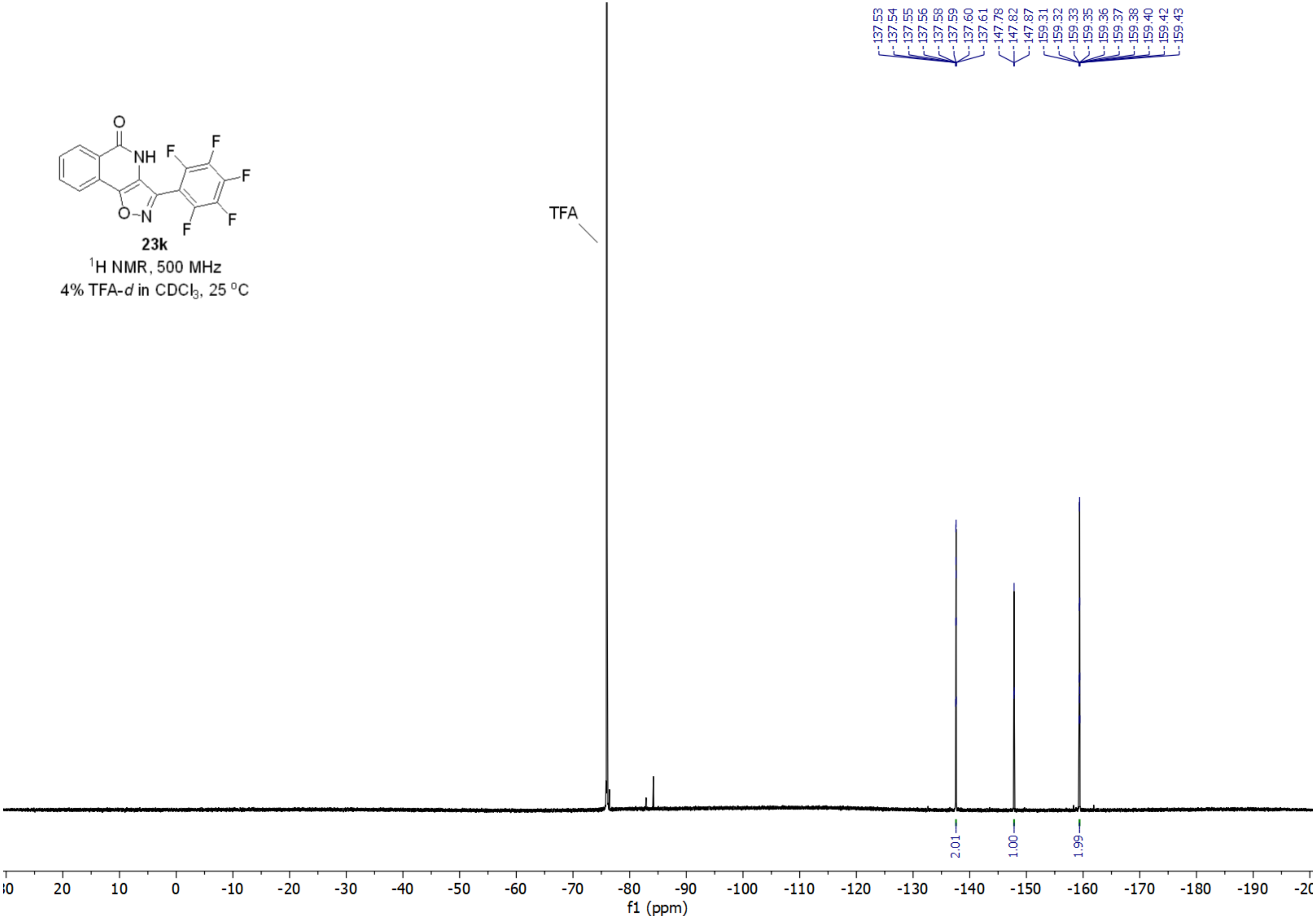

23k
^1H NMR, 500 MHz
4% TFA-*d* in $CDCl_3$, 25 °C
137.53
137.54
137.55
137.56
137.58
137.59
137.60
137.61
147.78
147.82
147.87
159.31
159.32
159.33
159.35
159.36
159.37
159.38
159.40
159.42
159.43
TFA
2.01
1.00
1.99
f1 (ppm)

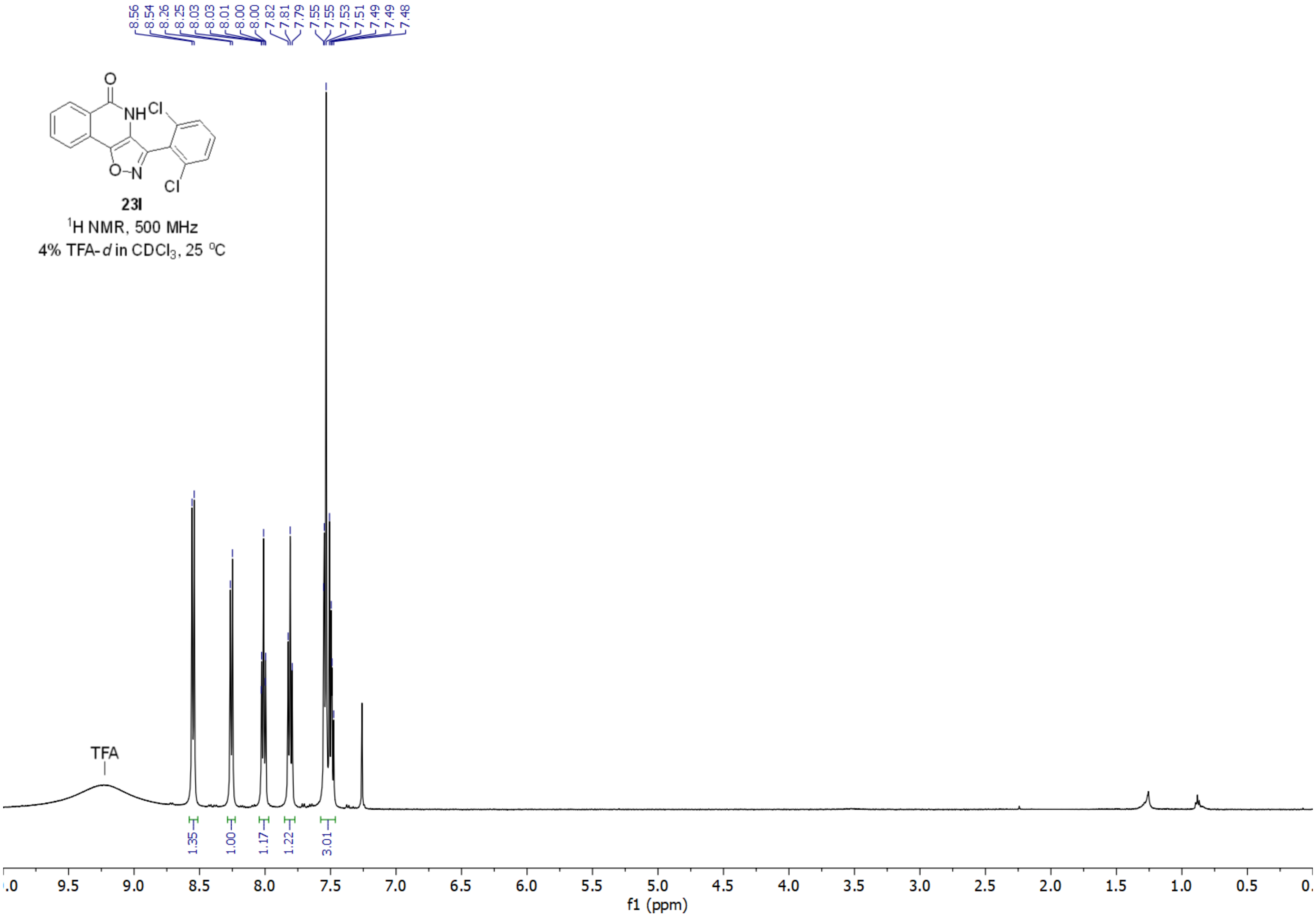
8.56
8.54
8.26
8.25
8.03
8.03
8.01
8.00
8.00
7.82
7.81
7.79
7.55
7.55
7.53
7.51
7.49
7.49
7.48
O
NH
Cl
O
N
Cl
23l
¹H NMR, 500 MHz
4% TFA-d in CDCl₃, 25 °C
TFA
1.35
1.00
1.17
1.22
3.01
9.5
9.0
8.5
8.0
7.5
7.0
6.5
6.0
5.5
5.0
4.5
4.0
3.5
3.0
2.5
2.0
1.5
1.0
0.5
f1 (ppm)

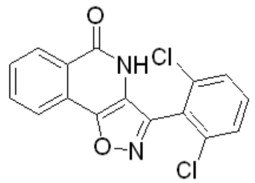

23l

^{13}C NMR, 125 MHz

4% TFA-*d* in $CDCl_3$, 25 °C

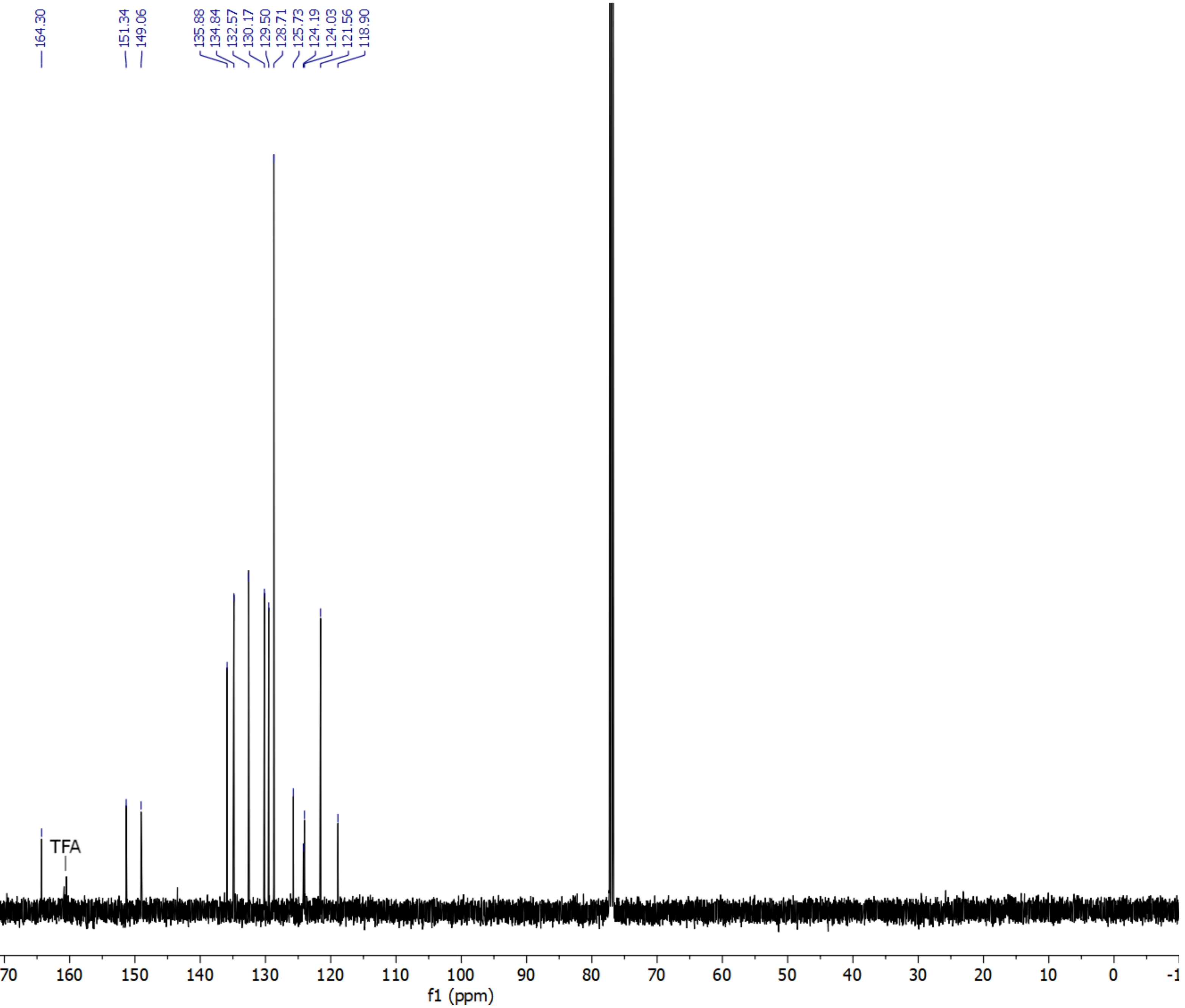

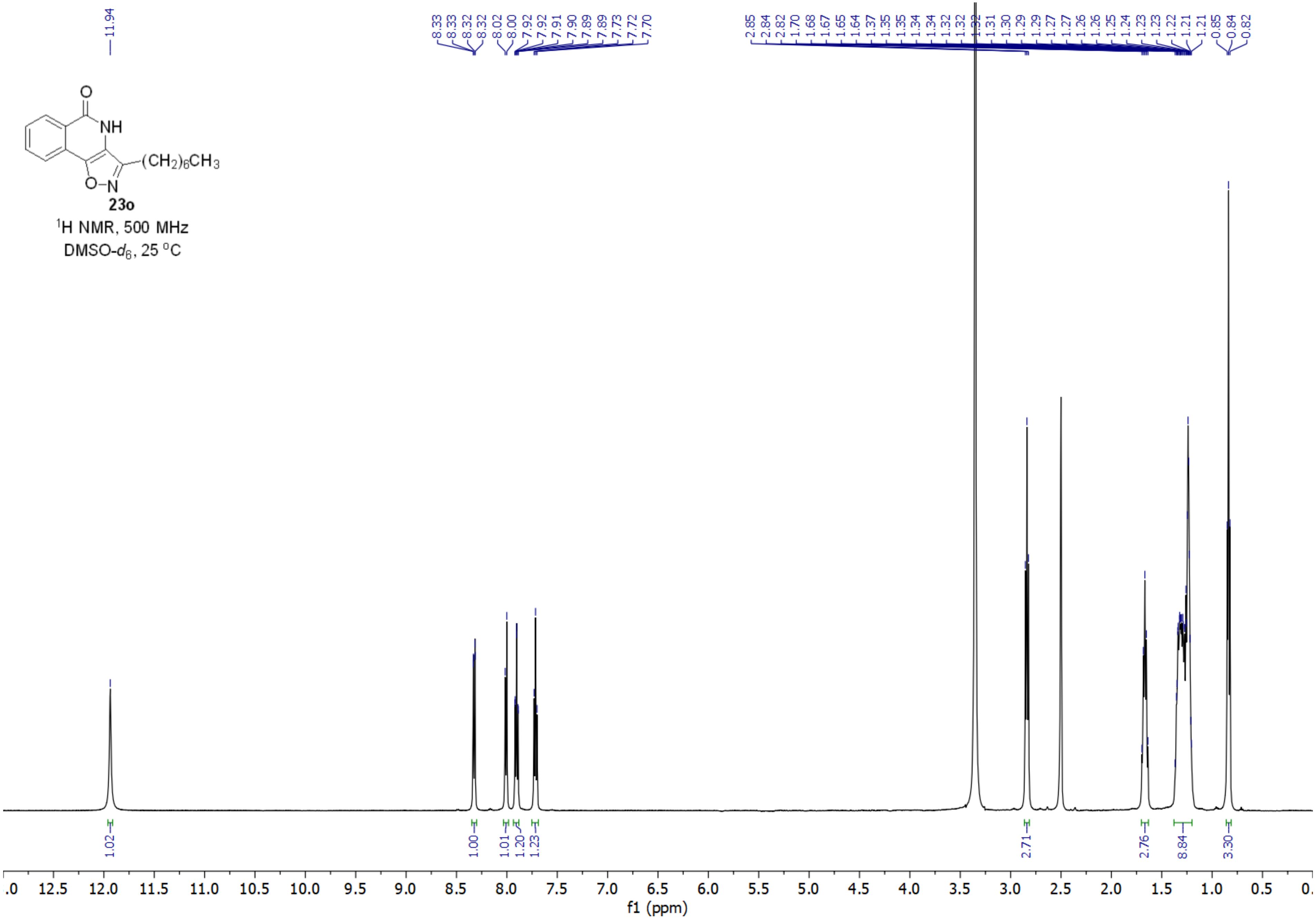

11.94
8.33
8.33
8.32
8.32
8.02
8.00
7.92
7.92
7.91
7.90
7.89
7.89
7.73
7.72
7.70
2.85
2.84
2.82
1.70
1.68
1.67
1.65
1.64
1.37
1.35
1.35
1.34
1.34
1.32
1.32
1.31
1.30
1.29
1.29
1.27
1.27
1.26
1.26
1.25
1.24
1.23
1.23
1.22
1.21
1.21
0.85
0.84
0.82
NH
O
(CH2)6CH3
O–N
23o
1H NMR, 500 MHz
DMSO-d6, 25 °C
1.02
1.00
1.01
1.20
1.23
2.71
2.76
8.84
3.30
f1 (ppm)

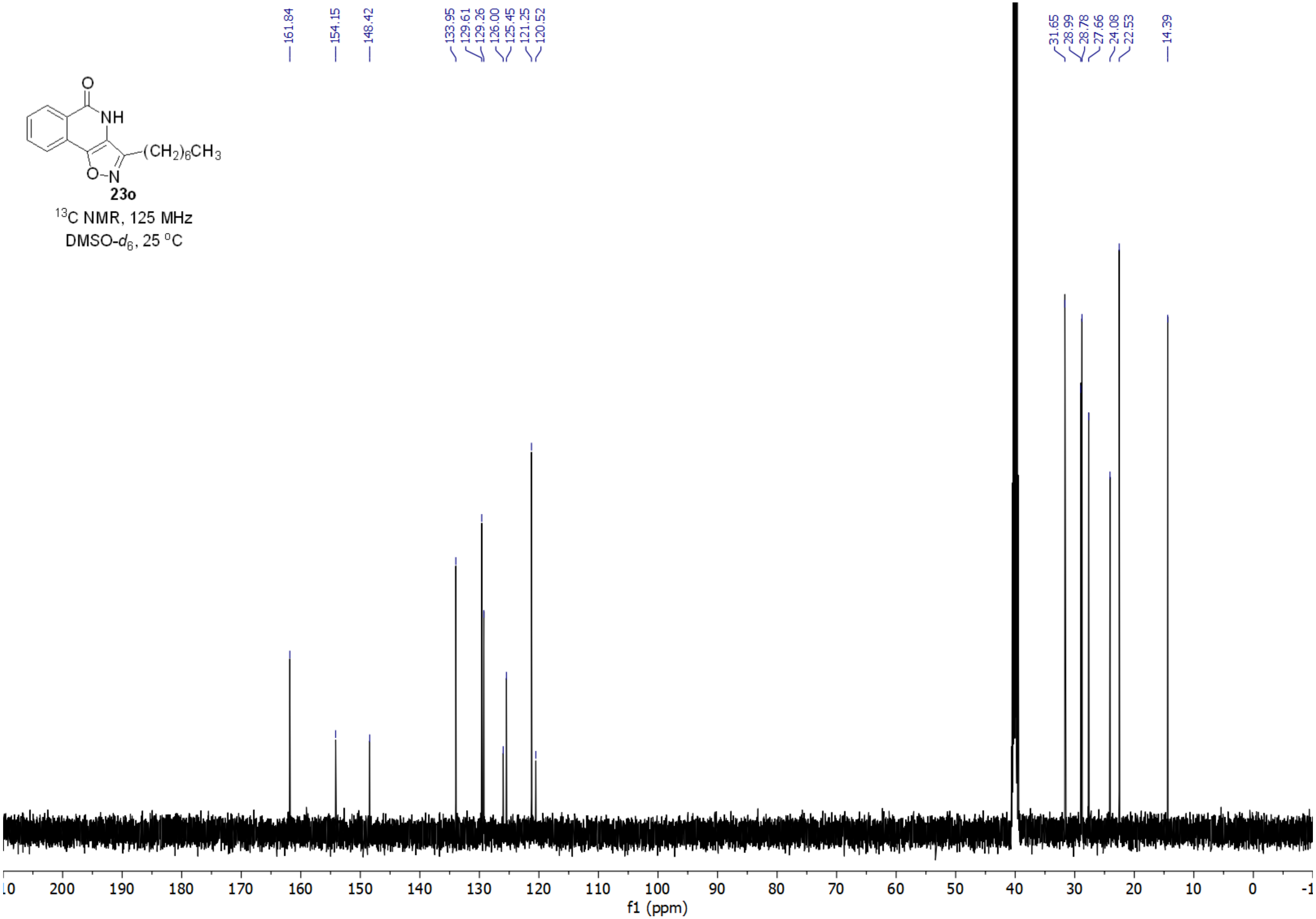

161.84
154.15
148.42
133.95
129.61
129.26
126.00
125.45
121.25
120.52
31.65
28.99
28.78
27.66
24.08
22.53
14.39
NH
(CH2)6CH3
O
N
23o
13C NMR, 125 MHz
DMSO-d6, 25 °C
f1 (ppm)

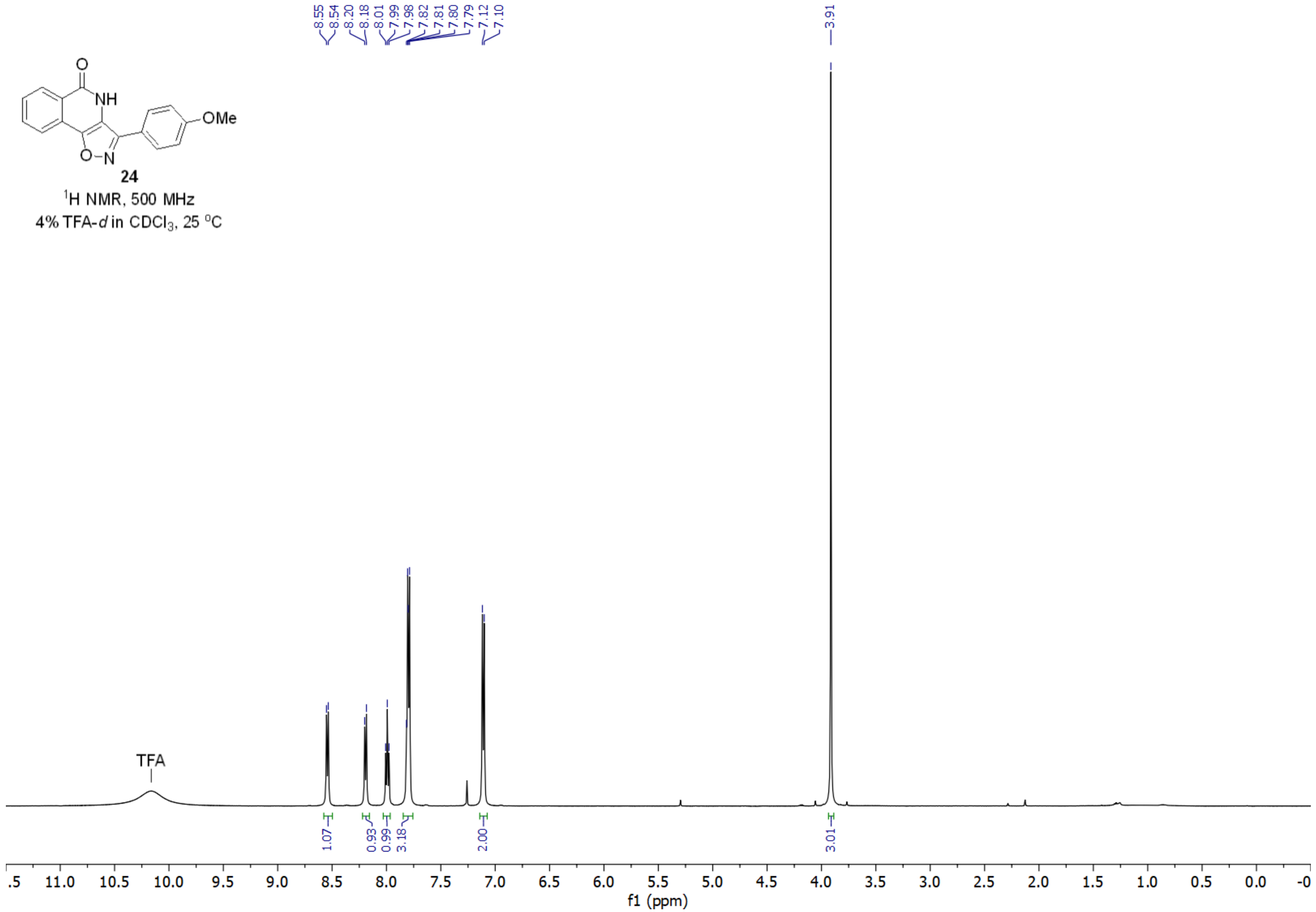

24
^{1}H NMR, 500 MHz
4% TFA-*d* in $CDCl_3$, 25 °C
TFA
f1 (ppm)

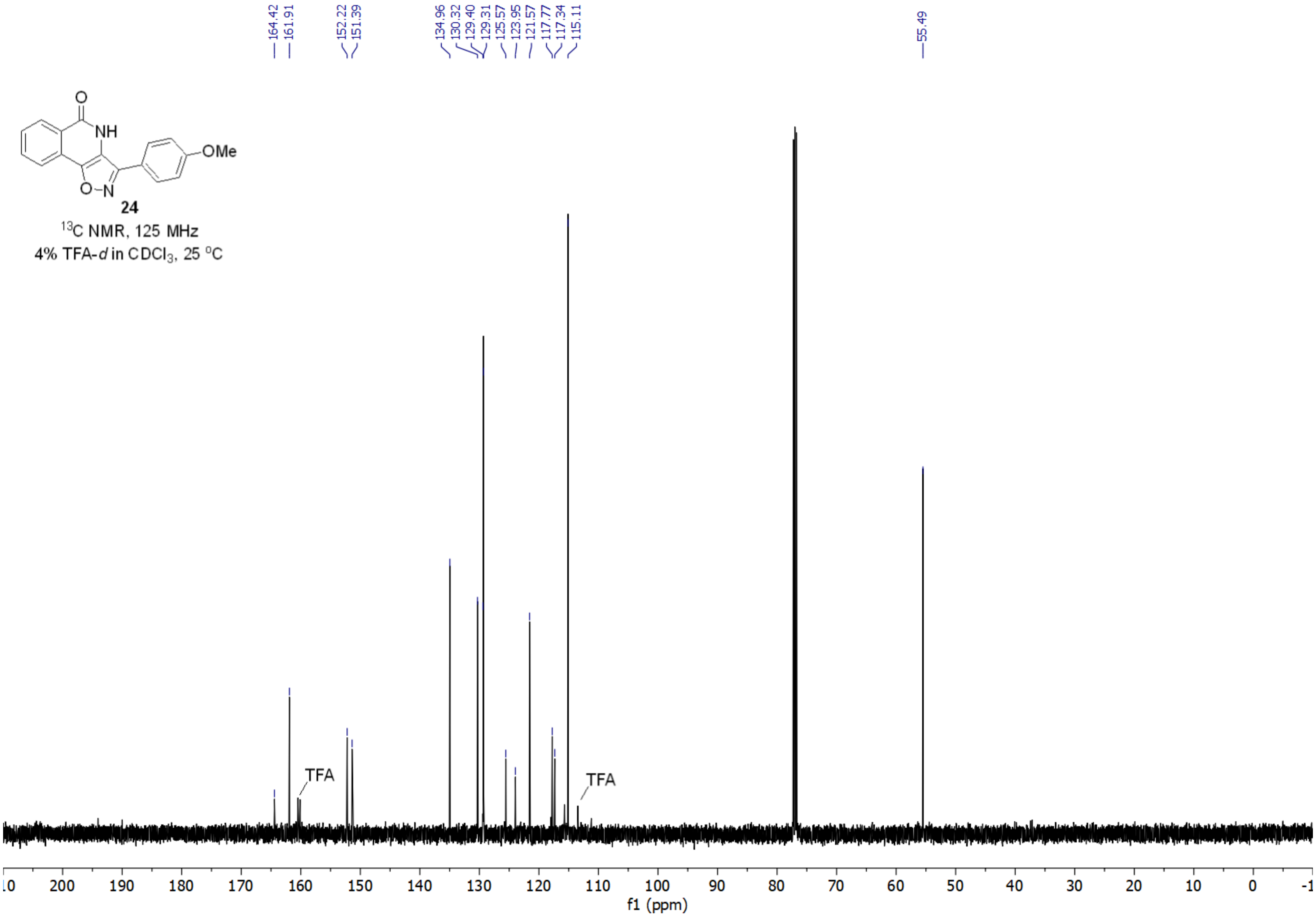
164.42
161.91
152.22
151.39
134.96
130.32
129.40
129.31
125.57
123.95
121.57
117.77
117.34
115.11
55.49
O
NH
OMe
O
N
24
^{13}C NMR, 125 MHz
4% TFA-*d* in $CDCl_3$, 25 °C
TFA
TFA
f1 (ppm)
